# Chemodivergent assembly of *ortho*-functionalized phenols with tunable selectivity via rhodium(III)-catalyzed and solvent-controlled C-H activation

**DOI:** 10.1038/s42004-021-00518-x

**Published:** 2021-06-03

**Authors:** Haiman Zhang, Shuang Lin, Hui Gao, Kaixin Zhang, Yi Wang, Zhi Zhou, Wei Yi

**Affiliations:** 1 Guangzhou Municipal and Guangdong Provincial Key Laboratory of Protein Modification and Degradation & Molecular Target and Clinical Pharmacology, State Key Laboratory of Respiratory Disease, School of Pharmaceutical Sciences & the Fifth Affiliated Hospital, Guangzhou Medical University, Guangzhou, Guangdong China; 2 School of Chemical Engineering and Technology, Sun Yat-Sen University, Guangzhou, Guangdong China

**Keywords:** Homogeneous catalysis, Diversity-oriented synthesis

## Abstract

*Ortho*-functionalized phenols and their derivatives represent prominent structural motifs and building blocks in medicinal and synthetic chemistry. While numerous synthetic approaches exist, the development of atom-/step-economic and practical methods for the chemodivergent assembly of diverse *ortho*-functionalized phenols based on fixed catalyst/substrates remains challenging. Here, by selectively controlling the reactivities of different sites in methylenecyclopropane core, Rh(III)-catalyzed redox-neutral and tunable C-H functionalizations of *N*-phenoxyacetamides are realized, providing access to both *ortho*-functionalized phenols bearing linear dienyl, cyclopropyl or allyl ether groups, and cyclic 3-ethylidene 2,3-dihydrobenzofuran frameworks under mild cross-coupling conditions. These divergent transformations feature broad substrate compatibility, synthetic applications and excellent site-/regio-/chemoselectivity. Experimental and computational mechanistic studies reveal that distinct catalytic modes involving selective β-C/β-H elimination, π-allylation, inter-/intramolecular nucleophilic substitution cascade and β-H’ elimination processes enabled by different solvent-mediated and coupling partner-controlled reaction conditions are crucial for achieving chemodivergence, among which a structurally distinct Rh(V) species derived from a five-membered rhodacycle is proposed as the corresponding active intermediates.

## Abstract (web summary)

Ortho functionalisation of phenols can be achieved using N-phenoxy amide directing groups. Here a method for chemodivergent C-H alkenylation, alkylation, carboetherification, or [3 + 2] annulation is presented, with product selectivity determined by the choice of solvent.

## Introduction

**Fig. 1:**
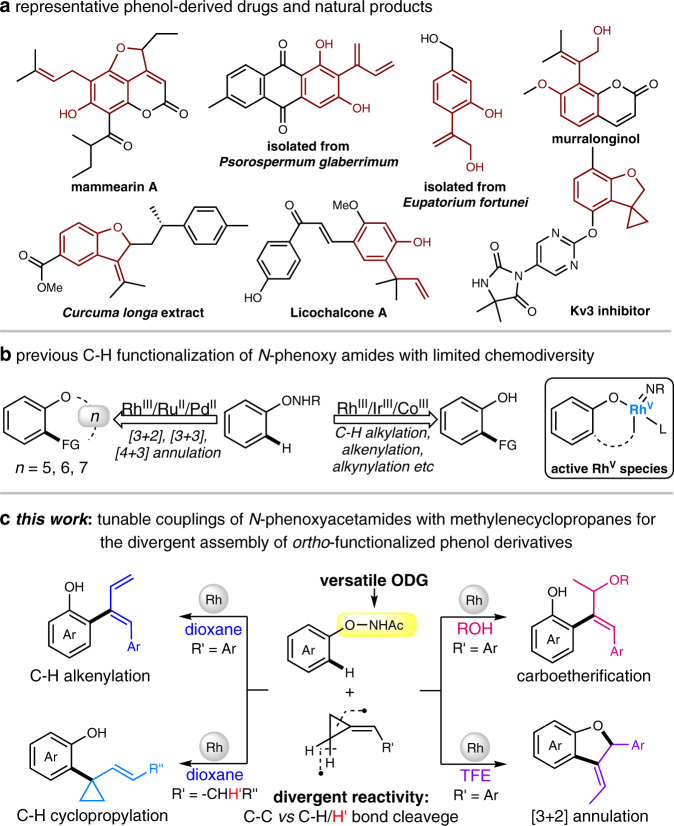
Chemodivergent assembly of *ortho*-functionalized phenols. **a** Representative molecules containing the privileged *ortho*-functionalized phenol core. **b**, **c** TM-catalyzed C–H functionalization for the construction of *ortho*-functionalized phenols.

Functionalized phenols and their derivatives have drawn intensive efforts from the community of synthetic organic chemistry due to their prevalent bioactivity and broad utility, such as using prominent building blocks (Fig. 1a)^1–4^. Consequently, a diverse range of synthetic approaches is developed for the efficient assembly of these motifs, among which the transition metal (TM)-catalyzed C–H functionalization represents a powerful and straightforward method toward their construction^5–9^. Indeed, various masked phenols equipped with different directing groups including silanol, carbonyl, phosphite, or carboxylic acid are employed so far for the site-selective C–H functionalization on the phenyl ring^10–13^. Most recently, the free hydroxyl group-directed specific *ortho*- or *para*-selective C–H alkylation has also been disclosed^14–18^. Despite the notable advances, further innovation to develop chemodivergent and highly regioselective C–H transformations for the rapid construction of privileged functionalized phenols is still desirable to meet the demand of green chemistry considering the reaction efficiency and diversity.

*N*-phenoxy amides bearing the -ONHR oxidizing directing groups (ODGs) have represented versatile substrates in Rh(III)-^19–33^, Pd(II)-^34^, Ir(III)-^35–37^, Co(III)-^38,39^, or Ru(II)-catalyzed^40^ C–H functionalization reactions, giving direct access to a variety of oxygen-containing heterocyclic compounds and *ortho*-functionalized phenols. Easily accessible C–H activation process of *N*-phenoxy amides mediated by an active Rh(III) catalyst and a putative, highly reactive Rh(V) species^41–43^ that was enabled by the post-insertion oxidative addition of O–N bond allow for the realization of diversified reactions under mild redox-neutral conditions with broad synthetic versatility (Fig. 1b). Nevertheless, in most cases almost only one main product could be formed with a fixed coupling partner (CP), which to some extent limits the chemodiversity and the synthetic efficiency. In searching for novel reaction modes of *N*-phenoxy amides, we envisioned that the rational design of a high reactive CP bearing divergent reactivity might endow with tunable transformations by appropriately adjusting different reaction pathways with the controllable selectivity, thus to achieve the chemodivergent C–H functionalization with the fixed substrates. Methylenecyclopropanes, with the presence of an alkene moiety and the strained cyclopropyl ring, are easily available building blocks that have been well-explored in diverse reactions including recently disclosed TM-catalyzed C–H functionalization^44–51^. Still, most of the developed C–H transformations with methylenecyclopropanes are basically limited to fixed products with given catalysts/substrates via classical C–H alkenylation or cyclopropylation. It is challenging but highly appealing to selectively control the different reactivity of the methylenecyclopropane core under mild and practical catalytic conditions to enable the novel reaction mode of the TM-catalyzed C–H functionalization.

Considering the potential divergent reactivities of methylenecyclopropanes and in combination with the highly reactive Rh(V) intermediates derived from *N*-phenoxy amides, we envisaged that the fine-tuning of different reaction parameters might stabilize particular rhodacycle intermediates, and thus switch the reaction pathway to afford chemodivergent products^52–55^. Herein, we report diverse synthesis of *ortho*-functionalized phenols and their derivatives via Rh(III)-catalyzed C–H couplings of *N*-phenoxyacetamides with methylenecyclopropanes in a tunable manner. By controlling the selective cleavage of different C–C and C–H bonds, a series of reaction modes including the classical C–H alkenylation and C–H cyclopropylation along with the highly attractive carboetherification and [3 + 2] annulation are realized, giving direct access to diene, 1-aryl-1-alkenyl disubstituted cyclopropane, allyl ether, as well as 3-ethylidene 2,3-dihydrobenzofuran frameworks, respectively, with broad substrate/ functional group compatibility (Fig. 1c). Experimental studies revealed that the reaction solvent is proven to play crucial roles in determining the observed chemoselectivity. DFT calculations defined the detailed solvent-dependent reaction manner and clarified the origin of the observed selectivity. Besides, synthetic applications in both the late-stage C-H modifications of bioactive compounds and the convenient derivations of the obtained diverse products have been demonstrated, which further strengthen the utility potential of these divergent transformations.

## Results

### Chemodivergent couplings of *N*-phenoxyacetamides and methylenecyclopropanes for the assembly of diversified phenols

At the outset of our investigation, *N*-phenoxyacetamide (**1a**) was treated with (cyclopropylidenemethyl)benzene (**2a**) under [Cp*RhCl_2_]_2_-catalyzed conditions in the presence of CsOAc, and the desired C-H activation/C-C cleavage of the cyclopropane moiety occurred smoothly, affording the desired diene product **3a** with specific stereoselectivity bearing a 1,2-diphenylethene moiety. Further screening of the related reaction parameters including the TM catalyst, solvent, reaction temperature, additives, and the ratio of the starting materials revealed that **3a** could be formed in a good yield with the following optimized conditions: **1a** (1 equiv), **2a** (2 equiv), [Cp*RhCl_2_]_2_ (5 mol%), and CsOAc (1 equiv) in dioxane (0.2 M) at 40 °C for 24 h under an atmosphere of air (see Supplementary Table. 1 in the Supplementary Information for details).

**Fig. 2:**
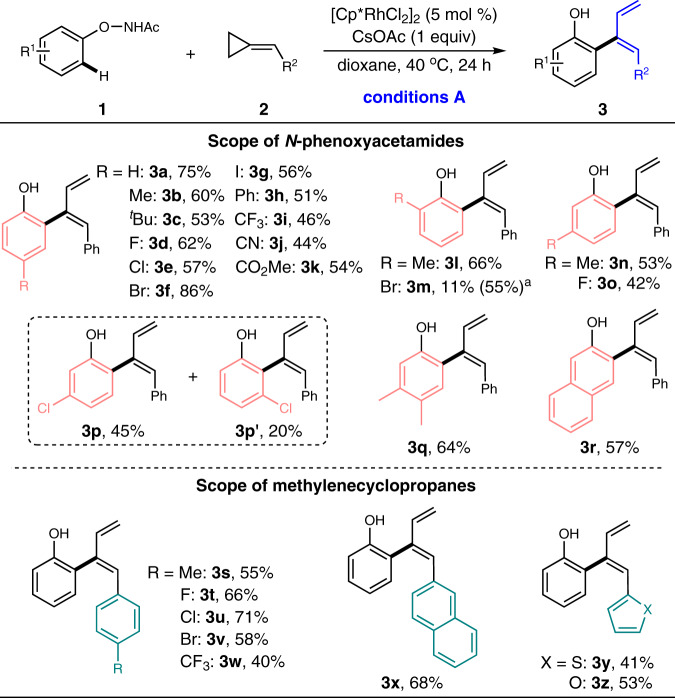
Substrate scope for the synthesis of dienes. Reaction conditions A: **1** (0.2 mmol), **2** (0.4 mmol), [Cp*RhCl_2_]_2_ (5 mol%), and CsOAc (1 equiv) in dioxane (0.2 M) at 40 °C for 24 h under air; isolated yields were reported. ^a^The reaction was conducted in MeOH (0.2 M).

Having established the optimal conditions, we then explored the versatility of the developed protocol in assembling diene skeletons. As shown in Fig. 2, a range of *N*-phenoxyacetamides bearing various commonly encountered functional groups including alkyl (**3b**–**c**), halogens (**3d**–**g**), phenyl (**3h**), trifluoromethyl (**3i**), cyano (**3j**) and ester (**3k**) were verified as good reactants for this transformation, affording the desired diene products in moderate to good yields. Various substituents at either *para*-, *ortho*-, or *meta*-position were all tolerated (**3l**–**q**), suggesting the substituted position had no obvious influence on the reactivity. Nevertheless, two regioisomers (**3p** and **3p’**) were obtained when *meta*-chloro substituted *N*-phenoxyacetamide was employed, while other *meta*-substituted substrates resulted in exclusive regioselectivity toward the less-hindered site, illustrating the nature of the substituent had an effect on determining the regioselectivity. Moreover, the naphthalene substrate also participated in this reaction, delivering the corresponding diene derivative **3r** in a synthetically useful yield. Subsequent examination of a diverse array of methylenecyclopropanes further extended the scope of this transformation, revealing that the reaction was compatible with various aryl-substituted methylenecyclopropanes regardless of the electronic properties of the substituent on the phenyl ring (**3s–w**). Gratefully, the methylenecyclopropanes bearing naphthyl, thienyl, and furyl groups were also viable substrates to provide the desired dienes (**3x**–**z**) smoothly.

**Fig. 3:**
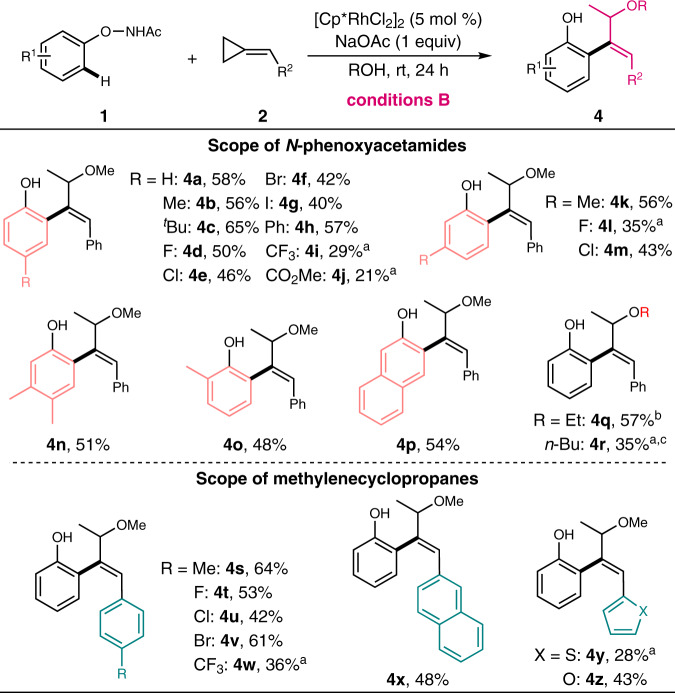
Substrate scope for the synthesis of allyl ethers. Reaction conditions B: **1** (0.2 mmol), **2** (0.4 mmol), [Cp*RhCl_2_]_2_ (5 mol%), and NaOAc (1 equiv) in MeOH (0.2 M) at room temperature for 24 h under air; isolated yields were reported. ^a^Diene derivative was obtained as the main product. ^b^EtOH was used instead of MeOH. ^c^*n*-BuOH was used instead of MeOH.

To further define the innovative reactivity of the versatile *N*-phenoxyacetamide and methylenecyclopropane substrates, especially to develop chemodivergent synthetic approaches for the diversified assembly of *ortho*-functionalized phenols by virtue of a tunable strategy, we next intrigued to screen the reaction parameters systematically. Interestingly, the results revealed that the reaction solvent exerted a crucial effect on determining the reaction outcome since alcoholic solvent led to the formation of unexpected allyl ether derivatives via regioselective carboetherification while other solvents led to the diene products. Enlightened by the intriguing chemoselectivity and the novel reactivity, we systematically defined the optimal reaction conditions (see Supplementary Table 2 in the Supplementary Information for details) and tested the substrate scope for the solvent-controllable C-H activation/etherification cascades. As shown in Fig. 3, various *N*-phenoxyacetamides were well tolerated to generate the desired allyl methyl ethers regardless of the substituted position on the phenyl ring (**4a**–**h**, **4k**, and **4m**–**p**). However, the electron-deficient *N*-phenoxyacetamides seemed to be less effective in delivering the corresponding allyl ether framework, in which diene derivatives were obtained instead (**4i**–**j** and **4l**). Notably, when *meta*-substituted *N*-phenoxyacetamides were employed, the C–H functionalization occurred at the less hindered site with specific regioselectivity. Other alcohols (e.g., EtOH and *n*-BuOH) were also good reaction media for such transformation, furnishing the desired allyl alkyl ethers smoothly (**4q** and **4r**). Inspired by these results, several methylenecyclopropanes were then examined to further probe the compatibility of this protocol, and the desired products were furnished smoothly under the standard conditions (**4s**–**z**), indicating that the developed synthetic route was broadly applicable in constructing the allyl alkyl ether skeletons.

**Fig. 4:**
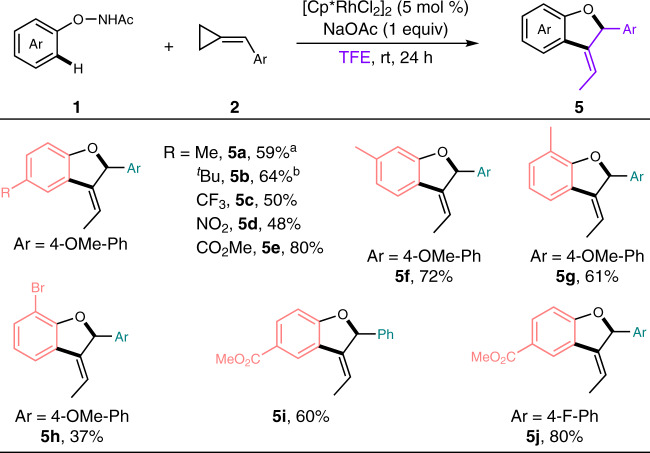
TFE-tuned [3 + 2] annulation for the selective synthesis of 3-ethylidene 2,3-dihydrobenzofurans. Reaction conditions: **1** (0.2 mmol), **2** (0.4 mmol), [Cp*RhCl_2_]_2_ (5 mol%) and NaOAc (1 equiv) in TFE (0.2 M) at room temperature for 24 h under air, isolated yields were reported. ^a^An inseparable mixture of two isomers was obtained, the ratio was determined to be 7.5/1 by ^1^H-NMR analysis. ^b^An inseparable mixture of two isomers was obtained, the ratio was determined to be 12/1 by ^1^H-NMR analysis.

Of note, an alternative [3 + 2] annulation was realized by simply switching the solvent to TFE instead of MeOH under conditions B, giving access to the 2,3-dihydrobenzofuran derivatives selectively (Fig. 4). This reaction featured specific regioselectivity and afforded the desired 2,3-dihydrobenzofuran core along with the equipment of an *E*-selective ethylidene moiety at the 3-position, which was contrary to the observed regioselectivity in MeOH that the methoxyl group connected at the less hindered site. It was noteworthy that the electronic property of the substituents on the phenyl ring had an effect on the regioselectivity of the desired product. Alkyl-substituted *N*-phenoxyacetamides led to an inseparable mixture of two isomers (**5a** and **5b**), while CF_3_−, NO_2_− and CO_2_Me-substituted substrates resulted in relatively high regioselectivity (**5c**–**e**). Further expansion of this [3 + 2] annulation to other *N*-phenoxyacetamides bearing either *meta*- or *ortho*-substituents as well as different methylenecyclopropanes furnished the desired products smoothly (**5f**–**j**), illustrating a good substrates compatibility. The observed chemodiversity as well as the distinct regioselectivity from fixed catalyst/substrates gave a strong hint that different reaction paths might be involved and could be handily tuned by adjusting the appropriate solvent.

**Fig. 5:**
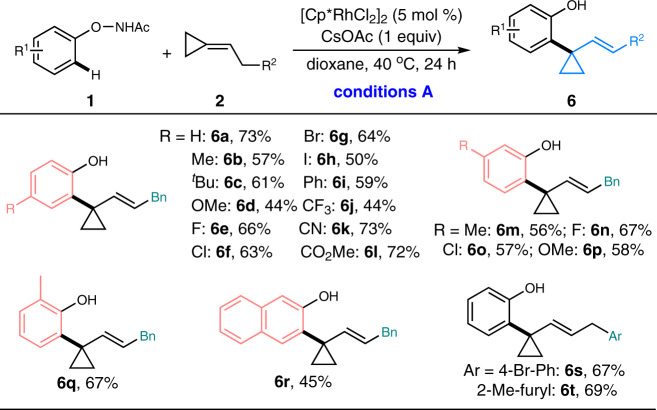
Substrate scope for C–H cyclopropylation. Reaction conditions A: **1** (0.2 mmol), **2** (0.4 mmol), [Cp*RhCl_2_]_2_ (5 mol%), and CsOAc (1 equiv) in dioxane (0.2 M) at 40 °C for 24 h under air, isolated yields were reported.

Considering the diverse reactivity of the methylenecyclopropane framework and encouraged by the developed divergent transformations of *N*-phenoxyacetamides with aryl-substituted methylenecyclopropanes via rhodium(III)-catalyzed C–H activation/ring-opening process, we envisaged that the C–H cyclopropylation might be realized by selectively tuning the β-H elimination process instead of the ring-opening of cyclopropane moiety. To our delight, the proposed reaction mode was successfully achieved in the presence of alkyl-substituted methylenecyclopropanes under standard conditions A, generating a variety of 1-aryl-1-alkenyl disubstituted cyclopropanes in decent yields (Fig. 5). The generality of this transformation was investigated with different *N*-phenoxyacetamides bearing both electron-donating and electron-withdrawing groups at either *para*-, *meta*-, or *ortho*-positions. Gratefully, these substrates were fully tolerated to afford the corresponding cyclopropanes in moderate to good yields (**6b**–**q**). Naphthalene substrate showed relatively lower efficiency, giving the desired product **6r** in 45% yield. Further examination demonstrated that the methylenecyclopropanes tethered with naphthalene moiety and substituted phenyl or furyl groups were also good reactants, forming the corresponding products (**6r–t**) in 45–69% yields. In all cases, the above-observed β-C elimination/ring-opening process was not detected, suggesting the β-H elimination was a more facile reaction path in these reaction conditions.

**Fig. 6:**
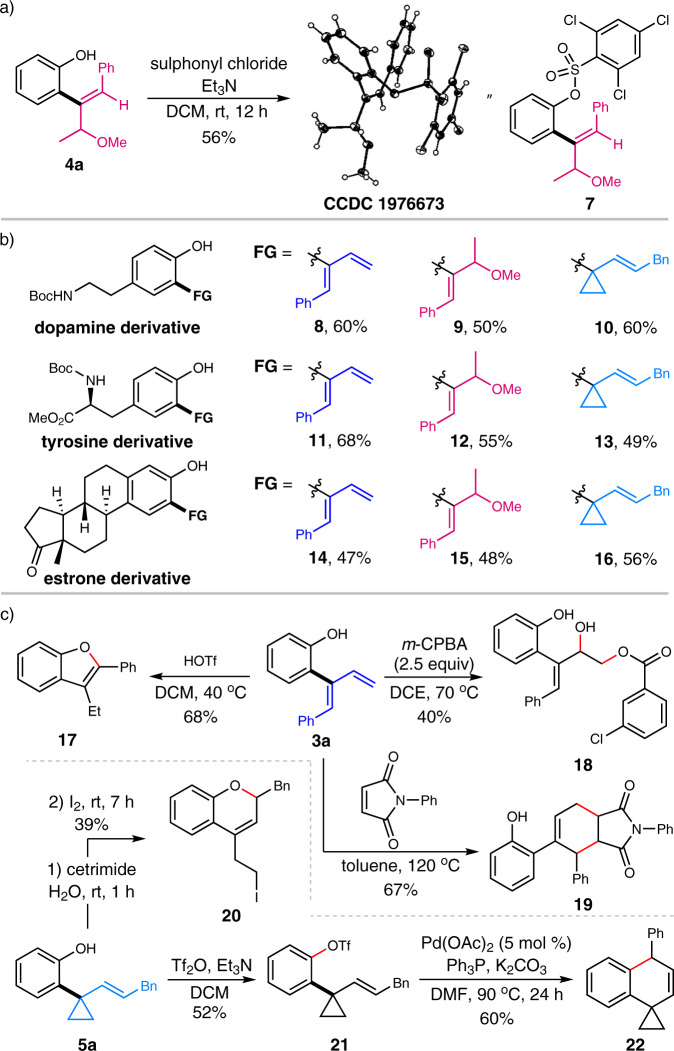
Synthetic applications. **a** Determination of the configuration. **b** Late-stage C–H modification of natural products. **c** Derivatizations of the product.

With the versatile protocols being established for the divergent construction of both diene, allyl ether, and 1,1-disubstituted cyclopropane skeletons, the synthetic applications of these products were next demonstrated. Protection of the hydroxyl group in allyl ether **4a** with sulphonyl chloride resulted in the formation of product **7**, which was confirmed by X-ray crystallography (CCDC 1976673) to uncover the absolute configuration of **4a** (Fig. 6a). The following application of the developed protocols for the late-stage C–H modification of complex bioactive compounds resulted in good compatibility since several substrates derived from natural product including dopamine, tyrosine, and estrone were fully tolerated with the above-established conditions to deliver the corresponding dienes, allyl ethers and cyclopropane derivatives smoothly (Fig. 6b). Evidently, the obtained products were useful synthetic intermediates due to the presence of the transformable alkenyl moieties and the hydroxyl group. For example, the treatment of the diene product **3a** with HOTf led to the formation of benzofuran derivative **17** with specific regioselectivity. In addition, the exposure of **3a** to *m*-CPBA resulted in the formation of an oxidative product **18**. Diels–Alder reaction of **3a** with *N*-phenylmaleimide in toluene gave facile access to the [4 + 2] adduct **19** in decent yield. Intriguingly, treatment of **5a** with I_2_ in the presence of cetrimide afforded 2*H*-chromene derivative **20** bearing an iodo-tethered alkyl chain. Further protection of the -OH in **5a** with Tf_2_O followed by Pd-catalyzed intramolecular annulation led to the formation of dihydronaphthalene derivative **22**. Taken together, these results further illustrated the profound synthetic potential for the rapid construction of various linear or cyclic skeletons via the conventionally chemical transformations (Fig. 6c).

### Experimental mechanistic studies

**Fig. 7:**
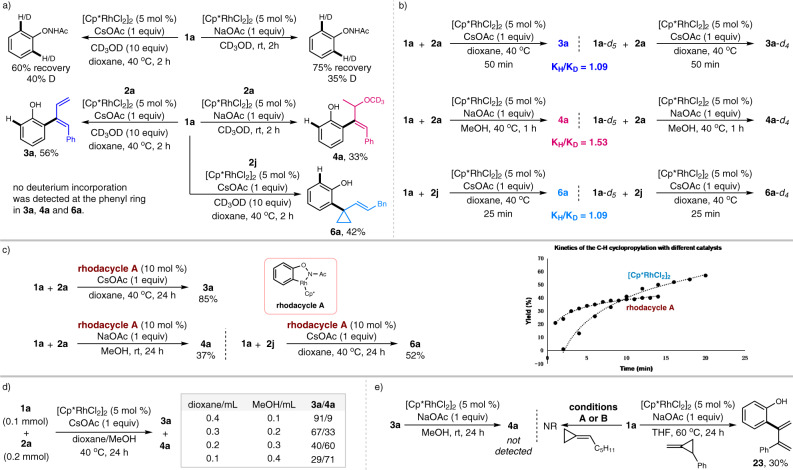
Experimental mechanistic studies. **a** Deuterium-labeling experiments. **b** Kinetic isotope studies. **c** Defining the five-membered rhodacycle as the active intermediate. **d** Studies on the solvent effect. **e** Control experiment.

Inspired by the abovementioned chemodivergent results, further investigations were next conducted to probe the reaction mechanism of these transformations. With the aid of CD_3_OD, deuterium-labeling experiments were first carried out to clarify the reversibility of the C–H bond cleavage process. The results showed approximately 40 and 35% deuteration at the *ortho*-positions of the recovered **1a** under both conditions A and B, respectively, suggesting a reversible C–H metalation. Another deuterium-labeling experiment of **1a** with methylenecyclopropanes in the presence of CD_3_OD gave the corresponding products **3a**, **4a**, and **6a** smoothly with no obvious deuterium incorporation at the *ortho*-position of the DG, while **4a** bearing -OCD_3_ was detected, proving that the methanol solvent was involved in delivering the allyl ether product (Fig. 7a). The kinetic isotope studies were next performed using **1a** and **1a**-*d*_*5*_ as the substrates by measuring the initial rates under different standard conditions for the formation of **3a**, **4a**, and **6a**. Primary KIE values were determined to be 1.09, 1.53, and 1.09, respectively, which revealed that the C–H bond cleavage might not be related to the rate-determining step (Fig. 7b).

To gain more insight into the mechanism, the rhodacycle **A** was readily prepared and proved to be an effective catalyst for the formation of diene, allyl ether and cyclopropane derivatives. The kinetics of the C–H cyclopropylation with either [Cp^*^RhCl_2_]_2_ or rhodacycle **A** was measured by ^1^H-NMR spectroscopy. The results demonstrated that relatively higher yields were obtained with the rhodacycle **A** as the catalyst at the initial stage of the reaction (within 10 min), thus defining that the rhodium(III)-catalyzed C–H activation process of *N*-phenoxyacetamide was initially involved to yield the five-membered rhodacycle **A** as an active intermediate for these reactions (Fig. 7c). Further studies on the solvent effect revealed that the selectivity between a diene and allyl ether products could be switched gradually with the addition of methanol under conditions A (Fig. 7d), which provided adequate evidence that the solvent played a crucial role in determining the chemoselectivity for the developed protocols. Control experiment revealed that the diene product **3a** could not be converted to allyl ether **4a** in the presence of methanol, supporting that **3a** and **4a** were formed via different reaction pathways. Furthermore, no reaction occurs when **1a** was treated with hexylidenecyclopropane under either condition A or B, implying that the developed protocols were suitable for aryl or heteroaryl tethered methylenecyclopropane substrates. Remarkably, in a search for other methylenecyclopropane skeletons that exhibit good reactivity with *N*-phenoxyacetamide under rhodium(III) catalysis, we turned our attention to (2-methylenecyclopropyl)benzene. Accordingly, the intriguing product **23** was formed smoothly, thus further verifying the versatility of the developed rhodium(III)-catalyzed C-H functionalization for constructing diverse diene fragments (Fig. 7e).

### Computational studies to further disclose the reaction mechanism

**Fig. 8:**
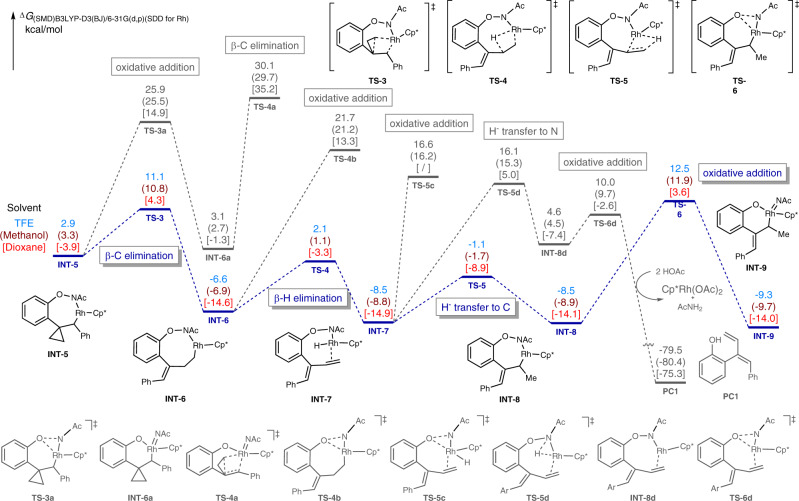
Computed Gibbs free energy changes of the reaction pathways for β-C elimination, β-H elimination, Rh-H reinsertion, and oxidative addition in the reaction solvents (TFE, MeOH, and dioxane). The corresponding free energy profiles of different solvents were presented as follows: blue values for TFE, brown values in the parentheses for methanol, and red values in the square brackets for dioxane.

To further figure out the reaction pathways for the developed chemodivergent C–H couplings, in particular, to clarify the origin of the distinctive chemoselectivity, DFT calculations were performed with Gaussian 09 by selecting the rhodacycle **INT-1** as the starting point. Computed Gibbs free energy changes of the reaction pathways were performed in different solvents including TFE, MeOH, and dioxane on the basis of our experimental results. Initially, the C–H bond cleavage and the migratory insertion of alkene processes were calculated (see Supplementary Fig. 1 in the Supplementary Information for details). With TFE being the solvent, the C–H activation occurred via a concerted-metalation-deprotonation transition state **TS-1** (*Δ*G^≠^ = 14.5 kcal/mol), followed by a regioselective alkene insertion via **TS-2** bearing a relatively higher free energy of 25.6 kcal/mol to afford **INT-5**. Similar results proceeded with a lower energy profile (values in the brackets) in MeOH or dioxane, indicating that the C–H activation was not the turnover limiting step, which was consistent with the experimentally observed low KIE values for these transformations. Subsequently, the β-C elimination from **INT-5** resulted in the formation of the ring-opening intermediate **INT-6** with an energy barrier of 8.2 kcal/mol (from **INT-5** to **TS-3**) in TFE, followed by the β-H elimination via **TS-4** (*∆*G^≠^ = 8.7 kcal/mol) to yield the diene intermediate **INT-7** (Fig. 8). From **INT-7**, different reaction paths were calculated to elucidate the proposed mechanism. The reinsertion of the alkene moiety into Rh-H bond occurred smoothly via **TS-5** (*∆*G^≠^ = 7.4 kcal/mol), delivering the seven-membered rhodacycle **INT-8** with a free energy of −8.5 kcal/mol. The following oxidative addition from **INT-8** led to the formation of Rh(V) species **INT-9** with an energy barrier of 21.0 kcal/mol. Alternatively, a Rh(III)-Rh(I)-Rh(III) reaction path involving the H^−^ transfer to N underwent via **TS-5d** with a relatively high energy barrier of 24.6 kcal/mol (from **INT-7** to **TS-5d**) to afford Rh(I) intermediate **INT-8d**, which proceeded the oxidative addition for the regeneration of Rh(III) species via **TS-6d** along with the release of the diene product **PC1** in the presence of HOAc. Taken together, the reaction pathway involving the Rh(V) intermediate was proved to be more reasonable due to the low energy barrier compared to the Rh(III)–Rh(I)–Rh(III) processes (21.0 vs. 24.6 kcal/mol).

Moreover, the comparisons of the oxidative addition in early stages were also performed. For example, the oxidative addition of **INT-5** delivered the six-membered Rh(V) species **INT-6a** via **TS-3a** (*∆*G^≠^ = 25.9 kcal/mol), the following β-C elimination of **INT-6a** ruled out this path due to the relatively high energy barrier of 30.1 kcal/mol (from **INT-1** to **TS-4a**). Other oxidative addition processes from **INT-6** or **INT-7** were also ruled out due to high energy barriers (e.g., 28.3 kcal/mol from **INT-6** to **TS-4b**, 25.1 kcal/mol from **INT-7** to **TS-5c**), thus provided adequate evidence to support the β-C elimination/β-H elimination/Rh-H reinsertion/oxidative addition cascade in TFE. Moreover, DFT calculations with MeOH or dioxane being the solvent resulted in a similar reaction path involving lower free energy barriers (20.8 kcal/mol from **INT-8** to **TS-6** in MeOH, 18.5 kcal/mol from **INT-7** to **TS-6** in dioxane) compared with TFE, demonstrating the Rh(V) species **INT-9** was involved as a common intermediate for these conditions.

**Fig. 9:**
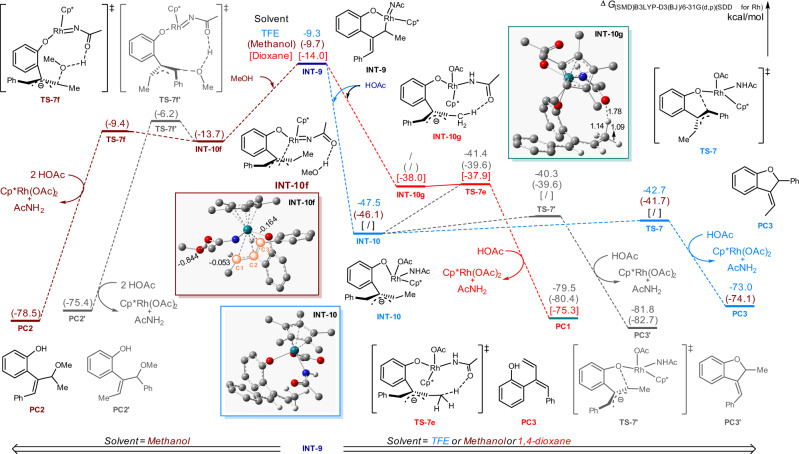
Computed Gibbs free energy changes for the divergent assembly of diene, allyl ether, and dihydrobenzofuran derivatives from INT-5 tuned by the reaction solvent. The corresponding free energy profiles of different solvents were presented as follows: blue values for TFE, brown values in the parentheses for methanol and red values in the square brackets for dioxane.

The origin of the observed chemoselectivity enabled by different reaction solvents was next clarified by calculating the possible reaction pathways from **INT-9**. By employing MeOH as the solvent, **INT-9** facilely converted into the more stable π-allyl rhodium species **INT-10f** with a free energy of −13.7 kcal/mol, in which the coordination of one molecular of MeOH via the hydrogen bond was proposed (Fig. 9). Further nucleophilic attack of MeOH to the π-allylrhodium moiety on either C_1_ or benzylic C_3_ position resulted in the formation of **PC2** or **PC2’**, among which **PC2** was both dynamically and thermodynamically favorable since a mild energy barrier of 4.3 kcal/mol (from **INT-10f** to **TS-7f**) was involved. NBO analysis showed that the C_1_ position of **INT-10f** was relatively more electrophilic compared with C_3_ position (−0.053 e vs. −0.164 e), thus accounted for the observed regioselectivity that the carboetherification occurred at the C_1_ position specifically.

In addition, with the aid of HOAc, the π-allylrhodium species **INT-10** was formed with a free energy of −47.5 kcal/mol in TFE, subsequent intramolecular nucleophilic attack of phenolate oxygen to the η^3^-allyl Rh(V) complex on benzylic C_3_ position resulted in the generation of 3-ethylidene 2,3-dihydrobenzofuran product **PC3** (G^≠^ = −79.5 kcal/mol) via **TS-7** (G^≠^ = −42.7 kcal/mol). As a comparison, the nucleophilic attack of phenolate oxygen on the C_1_ position via **TS-7’** possessed a relatively higher energy barrier (7.2 kcal/mol *vs* 4.8 kcal/mol), delivering a more stable 3-benzylidene 2,3-dihydrobenzofuran product **PC3’** with a free energy of −81.8 kcal/mol. These results demonstrated that the experimental observation of 3-ethylidene 2,3-dihydrobenzofurans under TFE-involved conditions was a dynamic control process. Similar results were obtained with MeOH being the solvent, in which an energy barrier of 4.4 kcal/mol (from **INT-10** to **TS-7**) was involved to form the [3 + 2] annulation product **PC3**. Interestingly, further investigation on the solvent effect in dioxane failed to afford the **INT-10**, while the distinctive intermediate **INT-10g** bearing an atypical hydrogen bond between the methyl and acyl group (1.78 Ǻ) was formed instead. The transformation of **INT-9** to **INT-10**, **INT-10f**, or **INT-10g** was investigated by geometry analysis, revealing that the release of the ring strain in **INT-9** and the facile conversion from η^1^ to η^3^ allyl rhodium species via π-allylation accounted for the exothermic process (see Supplementary Fig. 3 in the Supplementary Information for details). The following conversion from **INT-10g** via **TS-7e** (G^≠^ = −37.9 kcal/mol) gave facile access to the diene product **PC1**. Alternatively, a similar hydrogen transfer process from **INT-10** via **TS-7e** involved relatively higher energy barriers in the alcoholic solvents (6.1 kcal/mol in TFE, 6.5 kcal/mol in MeOH), which were in good agreement with the solvent-tunable experimental results.

Finally, with (3-cyclopropylidenepropyl)benzene being the CP, the subsequent β-H’ elimination, β-C elimination and oxidative addition process from **INT-5h** were compared (see Supplementary Fig. 2 in the Supplementary Information for details). The results revealed that the β-H’ elimination via **TS-3h** was more favorable with an energy barrier of 1.5 kcal/mol (from **INT-5h** to **TS-3h**), while the other two paths involved relatively higher energy barriers (24.4 kcal/mol for **TS-3i**, 4.4 kcal/mol for **TS-3j**), suggesting a facile β-H’ elimination process for this type of substrates.

### Mechanistic proposal

**Fig. 10:**
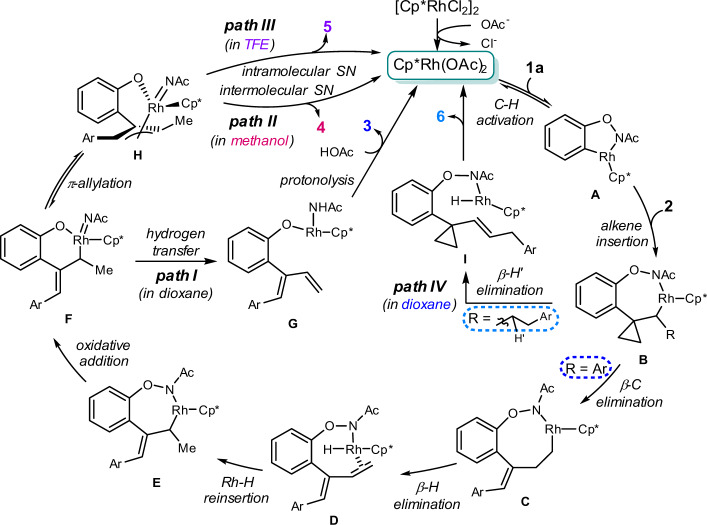
Proposed reaction pathways. Solvent-controlled diversified C–H activation cascades involving selective β-C/β-H elimination, π-allylation, inter-/intramolecular nucleophilic substitution, and β-H’ elimination processes were proposed.

On the basis of the combined experimental and computational mechanistic studies, we proposed the reaction pathways for generating these obtained frameworks (Fig. 10). Initially, the active Cp^*^Rh(OAc)_2_ is formed via anion exchange with the aid of acetate, followed by the reversible C–H activation to afford the five-membered rhodacycle **A**, which has been proved as the active intermediate for the following transformations. The regioselective migratory insertion of the double bond of methylenecyclopropane substrate into the C-Rh bond delivers intermediate **B**. Thereafter, distinct reaction pathways are involved tuned by the nature of the substituent on the methylenecyclopropane moiety and different reaction solvents. For aryl-substituted methylenecyclopropanes, **B** undergoes the ring-opening process of the cyclopropyl moiety via selective β-C/β-H eliminations to afford intermediate **D**, which undergoes the reinsertion of the double bond into the Rh-H bond to give the ƞ^1^-allylic rhodium species **E**. Further oxidative addition of **E** delivers the active Rh(V) species **F**, from which the solvent-tunable chemodivergent transformations are realized via different reaction paths. In the path I, the hydrogen transfer followed by protonolysis yields product **3** along with the regeneration of active Rh(III) catalyst. Alternatively, the facile conversion of **F** into more stable π-allyl rhodium intermediate **H** proceeds smoothly, further selective inter- (path II) or intramolecular (path III) nucleophilic substitution^56–58^ of the π-allyl rhodium in **H** by external alcohol or internal phenolate oxygen provides the product **4** or **5** in a controllable manner. On the other hand, with alkyl-substituted methylenecyclopropanes, the facile β-H’ elimination occurs to furnish intermediate **I** with the retention of the strained cyclopropane skeleton, generating the 1,1-disubstituted cyclopropane product **6** accordingly (path IV). It was worth noting that the generation of the active Rh(V) species seems to be reasonable due to the facile oxidative addition process with the -ONHAc ODG^24,41,43,59–62^, which was crucial for achieving the observed diversified chemoselectivities.

## Discussion

In summary, by virtue of the versatile -ONHAc as the ODG and methylenecyclopropane as the CP, diversified rhodium(III)-catalyzed C–H functionalization has been realized for the chemodivergent and controllable synthesis of diene, allyl ether, 1,1-disubstituted cyclopropane, and 3-ethylidene 2,3-dihydrobenzofuran derivatives with tunable chemoselectivity, good functional group compatibility, and potential synthetic utility. Integrated experimental and computational mechanistic studies revealed that the solvent-mediated and CP-controlled switches between different reaction paths were involved by employing unique and structurally different Rh(V) species as the corresponding active intermediates. Further investigation to probe a more detailed reaction mechanism, realize the stereoselective synthesis, and highlight the divergent construction of other heterocycles via tunable C-H functionalization strategy are in progress in our laboratory.

## Methods

### General information

NMR spectra were recorded on JEOL 400 NMR spectrometer (^1^H 400 MHz; ^13^C 100 MHz). The residual solvent signals were used as references and the chemical shifts converted to the TMS scale. Mass spectra and high-resolution mass spectra were measured on an Agilent TOF-G6230B mass spectrometer and Thermo-DFS mass spectrometer. Thin-layer chromatographies were done on pre-coated silica gel 60 F254 plates (Merck). Silica gel 60H (200–300 mesh) and preparative TLC (200 × 200 mm, 0.2–0.25 mm in thickness) manufactured by Qingdao Haiyang Chemical Group Co. (China) were used for general chromatography. X-ray crystallography of compound **7** was performed on an XtaLAB Synergy R, DW system, HyPix diffractometer using CuKα radiation.

### General procedure for the synthesis of dienes 3a–z and cyclopropane products 6a–t

The mixture of *N*-phenoxyacetamides **1** (0.2 mmol, 1.0 equiv), methylenecyclopropanes **2** (0.4 mmol, 2.0 equiv), [Cp*RhCl_2_]_2_ (5 mol%), and CsOAc (0.2 mmol, 1.0 equiv) in dioxane (1.0 mL) was stirred at 4 °C for 24 h without exclusion of air or moisture. Afterward, the solvent was removed under reduced pressure, and the resulted mixture was purified by preparative TLC to afford the corresponding derivatives.

### General procedure for the synthesis of allyl ethers 4a–z

The mixture of *N*-phenoxyacetamides **1** (0.2 mmol, 1.0 equiv), methylenecyclopropanes **2** (0.4 mmol, 2.0 equiv), [Cp*RhCl_2_]_2_ (5 mol%) and NaOAc (0.2 mmol, 1.0 equiv) in an alcoholic solvent (1.0 mL) was stirred at room temperature for 24 h without exclusion of air or moisture. Afterward, the solvent was removed under reduced pressure, and the resulted mixture was purified by preparative TLC to afford the corresponding derivatives.

### General procedure for the synthesis of 2,3-dihydrobenzofuran 5

The mixture of *N*-phenoxyacetamides **1** (0.2 mmol, 1.0 equiv), methylenecyclopropanes **2** (0.4 mmol, 2.0 equiv), [Cp*RhCl_2_]_2_ (5 mol%), and NaOAc (0.2 mmol, 1.0 equiv) in TFE (1.0 mL) was stirred at room temperature for 24 h without exclusion of air or moisture. Afterward, the solvent was removed under reduced pressure, and the resulted mixture was purified by preparative TLC to afford the corresponding 2,3-dihydrobenzofuran derivatives.

### Methods of the DFT calculations

DFT calculations were carried out using Gaussian 09. Geometry optimizations and frequency analyses were calculated using the SMD solvation model (solvent = TFE, methanol, and 1,4-dioxane) at the level of the B3LYP functional with Grimme’s DFT-D3 method and a mixed basis set of SDD for Rh and 6–31 G(d,p) for other atoms.

## Supplementary information

Supplementary Information

Description of Additional Supplementary Files

Supplementary Data 1

Supplementary Data 2

Supplementary Data 3

## Data Availability

The authors declare that the data supporting the findings of this study are available within the article and Supplementary Information file or from the corresponding author upon reasonable request. The X-ray crystallographic coordinates for the structure (Supplementary Data. 1) reported in this study has been deposited at the Cambridge Crystallographic Data Centre (CCDC) under deposition numbers CCDC 1976673. These data can be obtained free of charge from The Cambridge Crystallographic Data Centre via www.ccdc.cam.ac.uk/data_request/cif. The computed energy values and coordinates are available in Supplementary Data. 2. Original ^1^H, ^13^C, and ^19^F NMR spectra of the compounds obtained in this manuscript are available in Supplementary Data 3 (Supplementary Figs. 4–227).
