# Supplementary material for: Chemodivergent assembly of ortho-functionalized phenols with tunable selectivity via rhodium(III)-catalyzed and solvent-controlled C-H activation
Source: Commun Chem. 2021 Jun 3;4:81. doi: 10.1038/s42004-021-00518-x (PMC9814747; doi:10.1038/s42004-021-00518-x)
Supplement: Supplementary file 1 — Supplementary Information [file 42004_2021_518_MOESM1_ESM.pdf]

Supplementary Information

Chemodivergent Assembly of *ortho*-Functionalized Phenols with Tunable Selectivity via Rhodium(III)-Catalyzed and Solvent-Controlled C-H Activation

Haiman Zhang,[†,§] Shuang Lin,[†,§] Hui Gao,[†,§] Kaixin Zhang,[†,‡] Yi Wang,[†]

Zhi Zhou,[†,*] and Wei Yi[†,*]

[†]Guangzhou Municipal and Guangdong Provincial Key Laboratory of Protein Modification and Degradation & Molecular Target and Clinical Pharmacology, State Key Laboratory of Respiratory Disease, School of Pharmaceutical Sciences & the Fifth Affiliated Hospital, Guangzhou Medical University, Guangzhou, Guangdong 511436, China.

[‡]School of Chemical Engineering and Technology, Sun Yat-sen University, Guangzhou, Guangdong 510275, China

E-mail: yiwei@gzhmu.edu.cn; zhouzhi@gzhmu.edu.cn

[§]These authors contributed equally.

Tables of Contents

Supplementary Methods……………………………..…………………………S3

1. General…………………………………………………………….………S3

2. Experimental Information and Characterization Data..…………………...S3

3. Synthetic Applications……………………………………………….....S44

4. Mechanistic Studies….…………………………..................................….S54

5. X-Ray Crystallographic Data……………………………………………...S81

6. DFT Studies……………………………………………………..…...S82

7. References……………………….……………………….……………….......S84

Supplementary Methods

1. General

NMR spectra were recorded on JEOL 400 NMR (^{1}H 400 MHz; ^{13}C 100 MHz) in $CDCl_3$ orDMSO-d_6. Abbreviations for data quoted are s, singlet; brs, broad singlet; d, doublet; t, triplet; dd, doublet of doublets; m, multiplet. The residual solvent signals were used as references and the chemical shifts converted to the TMS scale ($CDCl_3$: δ_H = 7.26 ppm, δ_C = 77.16 ppm; DMSO-d_6: δ_H = 2.50 ppm, δ_C = 39.52 ppm; CD_3OD: δ_H = 3.31 ppm, δ_C = 49.00 ppm). Mass spectra and high-resolution mass spectra were measured on an agilent TOF-G6230B mass spectrometer and Thermo-DFS mass spectrometer. Thin-layer chromatographies were done on pre-coated silica gel 60 F254 plates (Merck). Silica gel 60H (200-300 mesh) and preparative TLC (200x200 mm, 0.2-0.25 mm in thickness) manufactured by Qingdao Haiyang Chemical Group Co. (China) were used for general chromatography. $[Cp^*RhCl_2]_2$, [Ru(*p*-cymene)$Cl_2]_2$ and $[Cp^*IrCl_2]_2$ were purchased from Aldrich and used without further purification. *N*-phenoxyacetamides[S1] were synthesized according to published procedures. Other chemicals were purchased from commercial suppliers and were dried and purified when necessary. No attempts were made to optimize yields for substrate synthesis.

2. Experimental Information and Characterization Data

General procedure for the synthesis of methylenecyclopropane substrates 2:

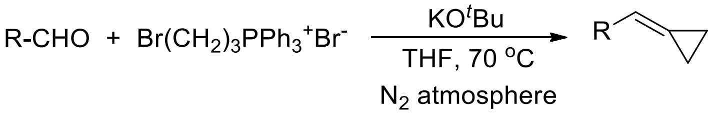

Methylenecyclopropanes **2** were prepared following a modifiedpublished procedure[S2]: A solution of KO^{t}Bu (3.0 equiv) in THF (3 M) was slowly added to a solution of (3-bromopropyl)triphenylphosphonium bromide (1.5 equiv) in dry THF (1.5 M) and stirred at 70 °C for 30min. Then a THF solution of aldehyde (1.0 equiv, 2 M) was added dropwise and the mixture was refluxed overnight under N_2 atmosphere. After cooling, the suspensionwas filtered and the filtrate was concentrated under

vacuum at 0 °C, the products were purified by column chromatography on silica gel, eluting with petroleum to afford methylenecyclopropanes.

2-(cyclopropylidenemethyl)furan(2i)

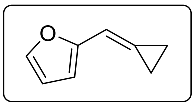

^{1}H NMR (400 MHz, $CDCl_3$): δ 7.31 (s, 1H), 6.70 (dd, *J* = 3.9, 1.9 Hz, 1H), 6.41 (dd, *J* = 3.3, 1.8 Hz, 1H), 6.33 (t, *J* = 3.2 Hz, 1H), 1.40-1.34 (m, 2H), 1.28-1.21 (m, 2H); ^{13}C NMR (100 MHz, $CDCl_3$): δ 153.7, 141.2, 123.7, 111.1, 108.2, 105.7, 3.6, 1.7; HRMS (ESI) calcd. for C_8H_9O ($[M+H]^+$): 121.0648; found: 121.0649.

1-bromo-4-(3-cyclopropylidenepropyl)benzene(2k)

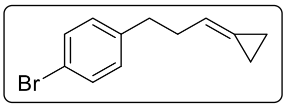

^{1}H NMR (400 MHz, $CDCl_3$): δ7.38 (d, *J* = 8.3 Hz, 2H), 7.07 (d, *J* = 8.3 Hz, 2H), 5.79-5.73 (m, 1H), 2.74-2.68 (m, 2H), 2.49-2.43 (m, 2H), 1.04-0.99 (m, 2H), 0.97-0.91 (m, 2H); ^{13}C NMR (100 MHz, $CDCl_3$): δ 141.2, 131.3, 130.3, 122.4, 119.6, 117.0, 35.2, 33.6, 2.4, 2.0; HRMS (ESI) calcd. for $C_{12}H_{12}Br$ ($[M-H]^-$): 235.0128; found: 235.0129.

2-(3-cyclopropylidenepropyl)-5-methylfuran (2l)

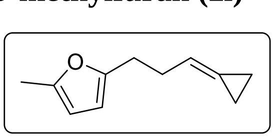

^{1}H NMR (400 MHz, $CDCl_3$): δ 5.81-5.77 (m, 3H), 2.75-2.64 (m, 2H), 2.53-2.44 (m, 2H), 2.20 (s, 3H), 1.01-0.97 (m, 4H); ^{13}C NMR (100 MHz, $CDCl_3$): δ 154.1, 150.0, 121.9, 117.2, 105.9, 105.4, 30.5, 28.0, 13.3, 2.1, 1.8; HRMS (ESI) calcd. for $C_{11}H_{15}O$ ($[M+H]^+$): 163.1118; found: 163.1114.

Optimization studies:

The mixture of *N*-phenoxyacetamide **1a** (0.1 mmol, 1.0 equiv),

methylenecyclopropanes **2a** (0.2 mmol, 2.0 equiv), catalyst (5 mol %), base (1.0 equiv) and additive in the solvent (0.2 M) was stirred at corresponding temperature for 24 h without exclusion of air or moisture. Afterwards, it was diluted with EtOAc and filtered through a short silica gel column to remove the metal residues. Then, the reaction mixture was concentrated and purified by preparative TLC (eluent: PE/EA = 10/1) to give the desired diene product **3a**.

Supplementary Table 1. Conditions Screening for the Synthesis of Diene Derivative **3a**[a]

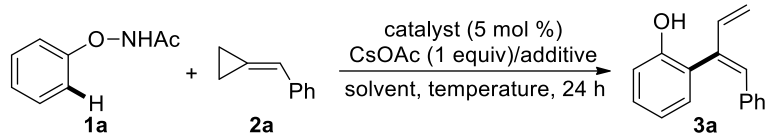

Entry	Catalyst	Solvent	Additive (equiv)	Yields (%)
1	$[Cp^*RhCl_2]_2$	THF	/	65
2	$[Cp^*IrCl_2]_2$	THF	/	trace
3	[Ru(*p*-cymene)$Cl_2]_2$	THF	/	trace
4	$Cp^*Co(CO)I_2$	THF	/	trace
5	[Os(*p*-cymene)$Cl_2]_2$	THF	/	nd
6	$Mn(CO)_5Br$	THF	/	nd
7	$[Cp^*RhCl_2]_2$	DCE	/	58
8	$[Cp^*RhCl_2]_2$	acetone	/	65
9	$[Cp^*RhCl_2]_2$	MeCN	/	41
10	$[Cp^*RhCl_2]_2$	toluene	/	61
11	$[Cp^*RhCl_2]_2$	DMF	/	41
12	$[Cp^*RhCl_2]_2$	DCM	/	23
13	$[Cp^*RhCl_2]_2$	dioxane	/	70
14	$[Cp^*RhCl_2]_2$	DMSO	/	trace
15	$[Cp^*RhCl_2]_2$	dioxane	HOAc (1)	66
16	$[Cp^*RhCl_2]_2$	dioxane	PivOH (1)	59
17	$[Cp^*RhCl_2]_2$	dioxane	4Å MS (20 mg)	50
18	$[Cp^*RhCl_2]_2$	dioxane	Amberlite IRA-400 (20 mg)	63
19	$[Cp^*RhCl_2]_2$	dioxane	Amberlite IR-120 (20 mg)	62
20[b]	$[Cp^*RhCl_2]_2$	dioxane	/	39
21[c]	$[Cp^*RhCl_2]_2$	dioxane	/	73

[a]Reaction conditions: **1a** (0.1 mmol, 1 equiv), **2a** (0.2 mmol, 2 equiv), catalyst (5 mol %) and base (1 equiv) in solvent (0.2 M) at room temperature for 24 h under air, isolated yields were reported.[b]NaOAc (1 equiv) was used as the base. [c]The reaction was conducted at 40 °C.

The mixture of *N*-phenoxyacetamide**1a**, methylenecyclopropane **2a**,catalyst(x mol %), base (1 equiv) andadditive in MeOH was stirred at the corresponding temperature for 24 h without exclusion of air or moisture. Afterwards, the solvent was removed under reduced pressure, and theresulted mixture was purified by preparative TLC(eluent: PE/EA = 10/1) to afford the desired allyl ether product**4a**.

Supplementary Table 2. Conditions Screening for the Synthesis of Allyl Ether **4a**[a]

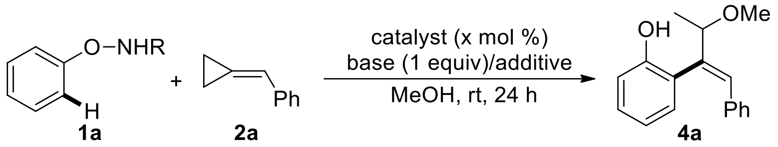

Entry	R	**1a**:**2a**	Catalyst (xmol %)	Base	Additive (equiv)	Yields (%)
1[b]	Ac	1:1	$[Cp^*RhCl_2]_2$ (2.5)	CsOAc	/	22
2[c]	Ac	1:1	$[Cp^*RhCl_2]_2$ (2.5)	CsOAc	/	24
3	Ac	1:1	$[Cp^*RhCl_2]_2$ (2.5)	CsOAc	/	30
4	Ac	1:1	$[Cp^*IrCl_2]_2$ (2.5)	CsOAc	/	trace
5	Ac	1:1	$Cp^*Co(CO)I_2$ (2.5)	CsOAc	/	NR
6	Ac	1:1	$Mn(CO)_5Br$ (2.5)	CsOAc	/	NR
7	Ac	1:1.5	$[Cp^*RhCl_2]_2$ (2.5)	CsOAc	/	38
8	Ac	1:2	$[Cp^*RhCl_2]_2$ (2.5)	CsOAc	/	43
9	Ac	1:2	$[Cp^*RhCl_2]_2$ (2.5)	KOAc	/	32
10	Ac	1:2	$[Cp^*RhCl_2]_2$ (2.5)	$Zn(OAc)_2$	/	29
11	Ac	1:2	$[Cp^*RhCl_2]_2$ (2.5)	K_2CO_3	/	trace
12	Ac	1:2	$[Cp^*RhCl_2]_2$ (2.5)	K_3PO_4	/	trace
13	Ac	1:2	$[Cp^*RhCl_2]_2$ (2.5)	KH_2PO_4	/	trace
14	Ac	1:2	$[Cp^*RhCl_2]_2$ (2.5)	Cs_2CO_3	/	trace
15	Ac	1:2	$[Cp^*RhCl_2]_2$ (2.5)	$Mn(OAc)_2$	/	18
16	Ac	1:2	$[Cp^*RhCl_2]_2$ (2.5)	KOPiv	/	17
17	Ac	1:2	$[Cp^*RhCl_2]_2$ (2.5)	NaOAc	/	47
18	Ac	1:2	$[Cp^*RhCl_2]_2$ (2.5)	NaOAc	HOAc (1)	38

19	Ac	1:2	$[Cp^*RhCl_2]_2$ (2.5)	NaOAc	TsOH (1)	NR
20	Ac	1:2	$[Cp^*RhCl_2]_2$ (2.5)	NaOAc	PivOH (1)	35
21	Ac	1:2	$[Cp^*RhCl_2]_2$ (2.5)	NaOAc	TFA (1)	NR
22	Ac	1:2	$[Cp^*RhCl_2]_2$ (2.5)	NaOAc	4Å MS (20 mg)	10
23[d]	Ac	1:2	$[Cp^*RhCl_2]_2$ (2.5)	NaOAc	Amberlite IRA-400	24
24[d]	Ac	1:2	$[Cp^*RhCl_2]_2$ (2.5)	NaOAc	Amberlite IR-120	46
25	Ac	1:2	$[Cp^*RhCl_2]_2$ (2.5)	NaOAc	$AgSbF_6$ (0.2)	30
26[e]	Ac	1:2	$[Cp^*RhCl_2]_2$ (5)	NaOAc	$AgNTf_2$ (0.2)	<10
27[e]	Ac	1:2	$[Cp^*RhCl_2]_2$ (5)	/	$AgNTf_2$ (0.2)	<10
28[e]	Ac	1:2	$[Cp^*RhCl_2]_2$ (5)	/	$AgSbF_6$ (0.2)	<10
29	Ac	1:2	$[Cp^*RhCl_2]_2$ (5)	NaOAc	/	44
30[e]	Ac	1:2	$[Cp^*RhCl_2]_2$ (5)	NaOAc	/	58
31[e]	Ac	1:2	$[Cp^*RhCl_2]_2$ (10)	NaOAc	/	49
32[e]	Ac	1:2	$[Cp^*Rh(OAc)_2]$ (5)	NaOAc	/	40
33[e]	Ac	1:2	$[Cp^*Rh(OAc)_2]$ (10)	NaOAc	/	38
34[e]	Ac	1:2	$[Cp^*Rh(OAc)_2]$ (10)	/	/	48
35[e]	Ac	1:2	$[Cp^{*Cy}RhCl_2]_2$(5)	NaOAc	/	50
36[e]	Ac	1:2	$[Cp^{*i\text{-}Pr}RhCl_2]_2$ (5)	NaOAc	/	44
37[e]	Ac	1:2	$[Cp^{*Ind}RhCl_2]_2$ (5)	NaOAc	/	trace
38[e]	Ac	1:2	$[Cp^{*2Ph}RhCl_2]_2$ (5)	NaOAc	/	trace
39[e]	Ac	1:2	$[Cp^{*Bn}RhCl_2]_2$(5)	NaOAc	/	37
40[e,f]	Ac	1:2	$[Cp^*RhCl_2]_2$ (5)	NaOAc	/	43
41[e]	Ac	1:2	$[Cp^*RhCl_2]_2$ (5)	NaOAc	AgOAc (1)	trace
42[e]	Ac	1:2	$[Cp^*RhCl_2]_2$ (5)	NaOAc	Ag_2O (1)	trace
43[e]	Ac	1:2	$[Cp^*RhCl_2]_2$ (5)	NaOAc	Ag_2CO_3(1)	<10
44[e]	Ac	1:2	$[Cp^*RhCl_2]_2$ (5)	NaOAc	$Cu(OAc)_2$(1)	<10
45[e]	Ac	1:2	$[Cp^*RhCl_2]_2$ (5)	NaOAc	$Na_2S_2O_8$ (1)	<10
46[e]	Ac	1:2.5	$[Cp^*RhCl_2]_2$ (5)	NaOAc	/	44
47[e]	Ac	1:3	$[Cp^*RhCl_2]_2$ (5)	NaOAc	/	39
48[e]	EtCO	1:2	$[Cp^*RhCl_2]_2$ (5)	NaOAc	/	35
49[e]	Bz	1:2	$[Cp^*RhCl_2]_2$ (5)	NaOAc	/	NR
50[e]	Ts	1:2	$[Cp^*RhCl_2]_2$ (5)	NaOAc	/	NR
51[e]	Piv	1:2	$[Cp^*RhCl_2]_2$ (5)	NaOAc	/	20

[a]Reaction conditions: **1a** (0.1 mmol, 1 equiv), **2** (0.1 mmol, 1 equiv), catalyst (2.5 mol %) and

base (1 equiv) in MeOH (0.1 M) at room temperature for 24 h under air. [b]The reaction was conducted at 60 °C. [c]The reaction was conducted at 40 °C. [d]20 mg ion exchange resin was added as the additive. [e]MeOH (0.2 M). [f]The reaction was conducted under an atmosphere of N_2.

General procedure for the synthesis of dienes 3a-z:

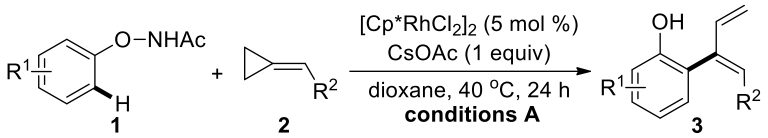

The mixture of *N*-phenoxyacetamides **1** (0.2 mmol, 1.0 equiv), methylenecyclopropanes **2** (0.4 mmol, 2.0 equiv), $[Cp^*RhCl_2]_2$ (5 mol %) and CsOAc (0.2 mmol, 1.0 equiv) in dioxane (1.0 mL) was stirred at 40 °C for 24 h without exclusion of air or moisture. Afterwards, the solvent was removed under reduced pressure, and theresulted mixture was purified by preparative TLCto afford the corresponding derivatives **3a-z.**

Characterization of products 3:

2-(1-phenylbuta-1,3-dien-2-yl)phenol (3a)

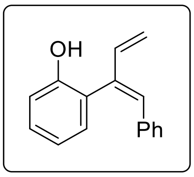

This compound was obtained in 75% yield (33.6 mg) as light yellow oil. Eluent: PE/EA = 10/1. R_f = 0.5; ^{1}H NMR (400 MHz, DMSO-*d6*): δ 9.20 (s, 1H), 7.24-7.18 (m, 1H), 7.15-7.08 (m, 3H), 6.99 (d,*J* = 7.4 Hz, 2H), 6.93 (d, *J* = 8.0 Hz, 1H), 6.88-6.81 (m, 2H), 6.72 (dd, *J* = 17.6, 9.7 Hz, 2H), 6.71 (s, 1H), 5.08 (d, *J* = 10.9 Hz, 1H), 4.66 (d, *J* = 17.2 Hz, 1H); ^{13}C NMR (100 MHz, DMSO-*d6*): δ 154.8, 141.3, 138.6, 136.9, 131.8, 130.4, 128.9, 128.7, 128.1, 127.0, 124.1, 119.4, 115.8, 115.1; HRMS (ESI) calcd. for $C_{16}H_{14}O$ ($[M+Na]^+$): 245.0937; found: 245.0937.

4-methyl-2-(1-phenylbuta-1,3-dien-2-yl)pheno (3b)

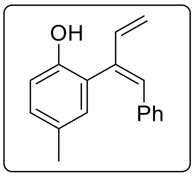

This compound was obtained in 60% yield (28.3 mg) as light yellow oil. Eluent: PE/EA = 10/1. R_f = 0.6; ^{1}H NMR (400 MHz, DMSO-d_6): δ 8.91 (s, 1H), 7.16-7.06 (m, 3H), 7.03-6.98 (m, 3H), 6.81 (d, *J* = 8.2 Hz, 1H), 6.74-6.65 (m, 3H), 5.07 (dd, *J* = 10.1, 1.4 Hz, 1H), 4.66 (d, *J* = 17.2 Hz, 1H), 2.17 (s, 3H); ^{13}C NMR (100 MHz, DMSO-d_6): δ 152.5, 141.3, 138.6, 136.8, 131.6, 130.4, 129.2, 128.7, 128.0, 127.7, 126.9, 123.8, 115.6, 115.1, 20.1; HRMS (ESI) calcd. for $C_{17}H_{15}O$ ([M-H]$^-$): 235.1128; found: 235.1121.

4-(*tert*-butyl)-2-(1-phenylbuta-1,3-dien-2-yl)phenol (3c)

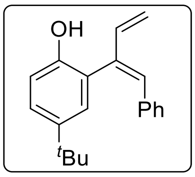

This compound was obtained in 53% yield (29.4 mg) as light yellow oil. Eluent: PE/EA = 10/1. R_f = 0.5; ^{1}H NMR (400 MHz, DMSO-d_6): δ 8.86 (s, 1H), 7.17-7.14 (m, 1H), 7.07-7.03 (m, 3H), 6.93-6.90 (m, 2H), 6.77-6.80 (m, 2H), 6.70-6.61 (m, 2H), 5.03 (dd, *J* = 10.2, 1.3 Hz, 1H), 4.65 (dd, *J* = 17.0, 1.4 Hz, 1H), 1.14 (s, 9H); ^{13}C NMR (100 MHz, DMSO-d_6): δ 152.3, 141.4, 141.2, 139.1, 136.8, 131.7, 128.7, 127.9, 127.2, 126.9, 125.2, 123.2, 115.3, 115.1, 33.6, 31.3; HRMS (ESI) calcd. for $C_{20}H_{21}O$ ([M-H]$^-$): 277.1598; found: 277.1594.

4-fluoro-2-(1-phenylbuta-1,3-dien-2-yl)phenol (3d)

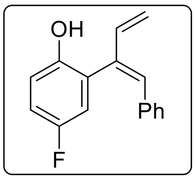

This compound was obtained in 62% yield (29.8 mg) as light yellow oil. Eluent: PE/EA = 10/1. R_f = 0.4; ^{1}H NMR (400 MHz, $CDCl_3$): δ7.19-7.13 (m, 3H), 7.02-6.97 (m, 3H), 6.91 (dd, *J* = 8.9, 4.7 Hz, 1H), 6.83 (s, 1H), 6.79 (dd, *J* = 8.6, 3.1 Hz, 1H), 6.72 (dd, *J* = 17.2, 10.4 Hz, 1H), 5.23 (d, *J* = 10.4 Hz, 1H), 4.94 (d, *J* = 17.1 Hz, 1H),

4.82 (s, 1H); ^{13}C NMR (100 MHz, $CDCl_3$): δ 157.3 (d, J = 239.0 Hz), 148.9, 139.6, 135.4, 134.9, 134.6, 129.2, 128.4, 128.3, 124.5, 124.5, 117.4, 117.1 (d, J = 8.1 Hz), 116.7 (d, J = 23.1 Hz), 116.3 (d, J = 22.9 Hz); ^{19}F NMR (376 MHz, $CDCl_3$): δ -123.42; HRMS (ESI) calcd. for $C_{16}H_{12}FO$ ($[M-H]^-$): 239.0877; found: 239.0870.

4-chloro-2-(1-phenylbuta-1,3-dien-2-yl)phenol (3e)

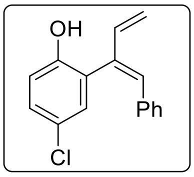

This compound was obtained in 57% yield (29.0 mg) as light yellow oil. Eluent: PE/EA = 10/1. R_f = 0.5; ^{1}H NMR (400 MHz, DMSO-d_6): δ9.55 (s, 1H), 7.25 (dd, J = 8.7, 2.7 Hz, 1H), 7.17-7.12 (m, 3H), 7.02-6.99 (m, 2H), 6.93 (d, J = 8.7 Hz, 1H), 6.85 (d, J = 2.7 Hz, 1H), 6.75-6.67 (m, 2H), 5.10 (d, J = 10.8 Hz, 1H), 4.64 (dd, J = 17.1, 0.8 Hz, 1H); ^{13}C NMR (100 MHz, DMSO-d_6): δ 154.0, 140.7, 137.1, 136.4, 132.3, 129.6, 128.6, 128.6, 128.2, 127.2, 126.1, 122.5, 117.4, 115.2; HRMS (ESI) calcd. for $C_{16}H_{12}ClO$ ($[M-H]^-$): 255.0582; found: 255.0579.

4-bromo-2-(1-phenylbuta-1,3-dien-2-yl)phenol (3f)

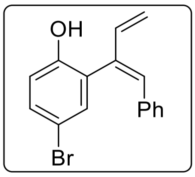

This compound was obtained in 86% yield (51.6 mg) as light yellow oil. Eluent: PE/EA = 10/1. R_f = 0.5; ^{1}H NMR (400 MHz, DMSO-d_6): δ 9.59 (s, 1H), 7.37 (dd, J = 8.7, 2.6 Hz, 1H), 7.20-7.10 (m, 3H), 7.02-6.96 (m, 3H), 6.89 (d, J = 8.7 Hz, 1H), 6.75-6.67(m, 2H), 5.10 (d, J = 10.7 Hz, 1H), 4.65 (d, J = 17.1 Hz, 1H); ^{13}C NMR (100 MHz, DMSO-d_6): δ 154.5, 140.7, 137.0, 136.4, 132.32, 132.26, 131.5, 128.6, 128.2, 127.3, 126.7, 117.9, 115.2, 110.1; HRMS (ESI) calcd. for $C_{16}H_{12}BrO$ ($[M-H]^-$): 299.0077; found: 299.0078.

4-iodo-2-(1-phenylbuta-1,3-dien-2-yl)phenol (3g)

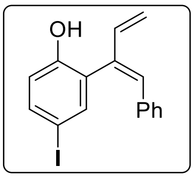

This compound was obtained in 56% yield (39.0 mg) as light yellow oil. Eluent: PE/EA = 10/1. R_f = 0.4; ^{1}H NMR (400 MHz, DMSO-d_6): δ 9.57 (s, 1H), 7.51 (dd, *J* = 8.5, 2.3 Hz, 1H), 7.20-7.11 (m, 4H), 7.00 (d, *J* = 6.9 Hz, 2H), 6.78 (d, *J* = 8.6 Hz, 1H), 6.74-6.65 (m, 2H), 5.09 (d, *J* = 10.1 Hz, 1H), 4.64 (d, *J* = 17.0 Hz, 1H); ^{13}C NMR (100 MHz, DMSO-d_6): δ 155.1, 140.8, 138.0, 137.4, 137.0, 136.4, 132.2, 128.7, 128.2, 127.3, 127.2, 118.5, 115.3, 80.9; HRMS (ESI) calcd. for $C_{16}H_{12}IO$ ([M-H]$^-$): 346.9938; found: 346.9938.

3-(1-phenylbuta-1,3-dien-2-yl)-[1,1'-biphenyl]-4-ol (3h)

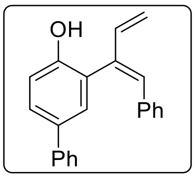

This compound was obtained in 51% yield (30.4 mg) as light yellow oil. Eluent: PE/EA = 10/1. R_f = 0.4; ^{1}H NMR (400 MHz, DMSO-d_6): δ 9.39 (s, 1H), 7.58-7.50 (m, 3H), 7.41-7.34 (m, 2H), 7.28-7.22 (m, 1H), 7.17-7.10 (m, 4H), 7.09-7.04 (m, 2H), 7.01 (d, *J* = 8.4 Hz, 1H), 6.81-6.71 (m, 2H), 5.11 (d, *J* = 11.0 Hz,1H), 4.74 (dd, *J* = 17.1, 1.4 Hz, 1H); ^{13}C NMR (100 MHz, DMSO-d_6): δ 154.7, 141.2, 139.7, 138.3, 136.8, 132.0, 131.2, 128.8, 128.7, 128.3, 128.1, 127.1, 127.0, 126.5, 125.8, 124.6, 116.4, 115.2; HRMS (ESI) calcd. for $C_{22}H_{17}O$ ([M-H]$^-$): 297.1285; found: 297.1284.

2-(1-phenylbuta-1,3-dien-2-yl)-4-(trifluoromethyl)phenol (3i)

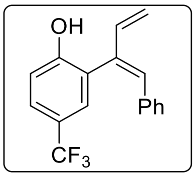

This compound was obtained in 46% yield (26.9 mg) as light yellow oil. Eluent: PE/EA = 10/1. R_f = 0.35; ^{1}H NMR (400 MHz, $CDCl_3$): δ 7.56 (dd, *J* = 8.3, 2.1 Hz, 1H), 7.37 (d, *J* = 1.9 Hz, 1H), 7.19-7.13 (m, 3H), 7.05 (d, *J* = 8.6 Hz, 1H), 6.98-6.95 (m, 2H), 6.88 (s, 1H), 6.74 (dd, *J* = 17.3, 10.5 Hz, 1H), 5.36 (s, 1H), 5.23 (d, *J* = 10.2

Hz, 1H), 4.87 (d, J = 17.2 Hz, 1H); ^{13}C NMR (100 MHz,$CDCl_3$): δ 155.5, 140.0, 135.5, 135.2, 133.9, 129.1, 128.7, 128.5, 128.1 (q, J = 3.7 Hz), 127.1 (q, J = 269.6 Hz), 127.0 (q, J = 3.7 Hz), 123.8, 123.6 (q, J = 35.5 Hz), 117.6, 116.3; ^{19}F NMR (376 MHz, DMSO-d_6): δ -61.33; HRMS (ESI) calcd. for $C_{17}H_{12}F_3O$ ([M-H]$^-$): 289.0845; found: 289.0846.

4-hydroxy-3-(1-phenylbuta-1,3-dien-2-yl)benzonitrile (3j)

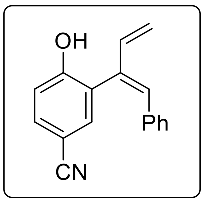

This compound was obtained in 44% yield (21.7 mg) as light yellow oil. Eluent: PE/EA = 10/1. R_f = 0.5; ^{1}H NMR (400 MHz, $CDCl_3$): δ 7.59 (dd, J = 8.5, 2.1 Hz, 1H), 7.40 (d, J = 2.0 Hz, 1H), 7.19-7.14 (m, 3H), 7.04 (d, J = 8.5 Hz, 1H), 6.97-6.93 (m, 2H), 6.90 (s, 1H), 6.73 (dd, J = 17.2, 10.5 Hz, 1H), 5.71 (brs, 1H), 5.24 (d, J = 10.4 Hz, 1H), 4.85 (d, J = 17.2 Hz, 1H); ^{13}C NMR (100 MHz, DMSO-d_6): δ 159.5, 140.5, 136.31, 136.27, 134.7, 133.7, 132.7, 128.6, 128.2, 127.4, 125.8, 119.3, 116.8, 115.2, 101.6; HRMS (ESI) calcd. for $C_{17}H_{12}NO$ ([M-H]$^-$): 246.0924; found: 246.0921.

methyl-4-hydroxy-3-(1-phenylbuta-1,3-dien-2-yl)benzoate (3k)

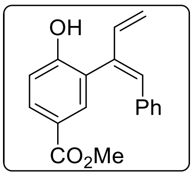

This compound was obtained in 54% yield (30.3 mg) as light yellow oil. Eluent: PE/EA = 10/1. R_f = 0.25; ^{1}H NMR (400 MHz, $CDCl_3$): δ 8.01 (dd, J = 8.6, 2.2 Hz, 1H), 7.82 (d, J = 2.0 Hz, 1H), 7.16-7.12 (m, 3H), 7.02-6.97 (m, 3H), 6.88 (s, 1H), 6.75 (dd, J = 17.2, 10.4 Hz, 1H), 5.44 (s, 1H), 5.22 (d, J = 10.5 Hz, 1H), 4.88 (d, J = 17.2 Hz, 1H), 3.87 (s, 3H); ^{13}C NMR (100 MHz, DMSO-d_6): δ 166.0, 159.8, 140.9, 137.4, 136.5, 132.4, 132.1, 130.9, 128.7, 128.3, 127.3, 124.5, 120.8, 115.9, 115.3, 51.7; HRMS (ESI) calcd. for $C_{18}H_{15}O_3$ ([M-H]$^-$): 279.1026; found: 279.1027.

2-methyl-6-(1-phenylbuta-1,3-dien-2-yl)phenol (3l)

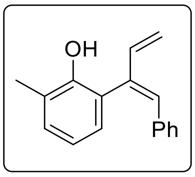

This compound was obtained in 66% yield (31.2 mg) as light yellow oil. Eluent: PE/EA = 10/1. R_f = 0.5; ^{1}H NMR (400 MHz, $CDCl_3$): δ 7.18-7.12 (m, 4H), 7.03-6.97 (m, 2H), 6.91-6.86 (m, 2H), 6.82 (s, 1H), 6.75 (dd, *J* = 17.0, 10.4 Hz, 1H), 5.21 (d, *J* = 10.4 Hz, 1H), 4.97 (s, 1H), 4.94 (d, *J* = 17.1 Hz, 1H), 2.26 (s, 3H); ^{13}C NMR (100 MHz, $CDCl_3$): δ 150.7, 140.2, 135.92, 135.89, 134.5, 130.9, 129.3, 128.4, 123.0, 127.9, 125.0, 122.8, 120.8, 117.3, 16.3; HRMS (ESI) calcd. for $C_{17}H_{17}O$ ($[M+H]^+$): 237.1274; found: 237.1271.

2-bromo-6-(1-phenylbuta-1,3-dien-2-yl)phenol (3m)

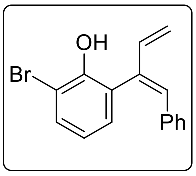

This compound was obtained in 11% yield (6.6 mg) as light yellow oil. Another batch of 3m was obtained in 55% yield (33.0 mg) when MeOH was used as the reaction solvent. Eluent: PE/EA = 10/1. R_f = 0.7; ^{1}H NMR (400 MHz, DMSO-d_6): δ 9.00 (s, 1H), 7.54 (d, *J* = 7.8 Hz, 1H), 7.17-7.13 (m, 3H), 6.97 (d, *J* = 7.6 Hz, 2H), 6.89 (d, *J* = 7.3 Hz, 1H), 6.84 (t, *J* = 7.7 Hz, 1H), 6.80 (s, 1H), 6.74 (dd, *J* = 17.2, 10.3 Hz, 1H), 5.11 (d, *J* = 10.3 Hz, 1H), 4.60 (d, *J* = 17.1 Hz, 1H); ^{13}C NMR (100 MHz, DMSO-d_6): δ 151.1, 140.9, 136.9, 136.3, 133.1, 132.4, 129.9, 128.8, 128.2, 127.4, 126.4, 121.4, 115.3, 111.0; HRMS (ESI) calcd. for $C_{16}H_{12}BrO$ ($[M-H]^-$): 299.0077; found: 299.0077.

5-methyl-2-(1-phenylbuta-1,3-dien-2-yl)phenol (3n)

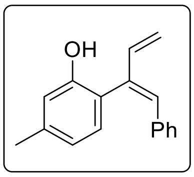

This compound was obtained in 53% yield (25.0 mg) as light yellow oil. Eluent: PE/EA = 10/1. R_f = 0.5; ^{1}H NMR (400 MHz, DMSO-d_6): δ 9.02 (s, 1H), 7.18-7.04 (m,

3H), 7.04-6.95 (m, 2H), 6.82-6.70 (m, 2H), 6.70-6.58 (m, 3H), 5.05 (dd, *J* = 10.2, 1.6 Hz, 1H), 4.65 (dd, *J* = 17.0, 1.4 Hz, 1H), 2.27 (s, 3H); ^{13}C NMR (100 MHz, DMSO-d_6): δ 154.6, 141.5, 138.6, 138.0, 136.9, 131.8, 130.1, 128.7, 128.0, 126.9, 121.1, 120.2, 116.4, 115.0, 21.0; HRMS (ESI) calcd. for $C_{17}H_{15}O$ ([M-H]$^-$): 235.1128; found: 235.1123.

5-fluoro-2-(1-phenylbuta-1,3-dien-2-yl)phenol (3o)

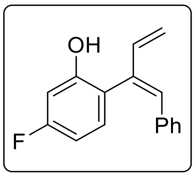

This compound was obtained in 42% yield (20.0 mg) as light yellow oil. Eluent: PE/EA = 10/1. R_f = 0.4; ^{1}H NMR (400 MHz, DMSO-d_6): δ 9.67 (s, 1H), 7.26-7.20 (m, 1H), 7.19-7.10 (m, 3H), 7.05-7.02 (m, 2H), 6.85 (s, 1H), 6.77-6.65 (m, 3H), 5.06 (d, *J* = 10.5 Hz, 1H), 4.67 (d, *J* = 17.1 Hz, 1H); ^{13}C NMR (100 MHz, DMSO-d_6): δ 159.9 (d, *J* = 241.2 Hz), 156.5 (d, *J* = 7.8 Hz), 140.1, 136.6, 133.4, 131.9, 129.6, 129.5, 128.2, 127.4, 114.3, 112.1 (d, *J* = 20.1 Hz), 111.6, 105.9 (d, *J* = 22.3 Hz); ^{19}F NMR (376 MHz, DMSO-*d6*): δ -114.85; HRMS (ESI) calcd. for $C_{16}H_{12}FO$ ([M-H]$^-$): 239.0877; found: 239.0872.

5-chloro-2-(1-phenylbuta-1,3-dien-2-yl)phenol (3p)

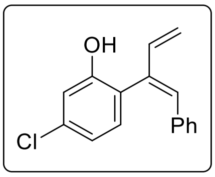

This compound was obtained in 45% yield (23.1 mg) as light yellow oil. Eluent: PE/EA = 10/1. R_f = 0.55; ^{1}H NMR (400 MHz, $CDCl_3$): δ 7.23 (dd, *J* = 8.2, 2.4 Hz, 1H), 7.19-7.16 (m, 3H), 7.06 (d, *J* = 2.4 Hz, 1H), 7.03-6.99 (m, 2H), 6.91 (d, *J* = 8.2Hz, 1H), 6.83 (s, 1H),6.72 (dd, *J* = 17.3, 10.2 Hz, 1H), 5.23 (d, *J* = 10.3 Hz, 1H), 4.97 (brs, 1H), 4.93 (d, *J* = 17.1 Hz, 1H); ^{13}C NMR (100 MHz, DMSO-d_6): δ 156.2, 139.5, 136.6, 135.3, 133.2, 132.7, 129.6, 128.24, 128.17, 127.3, 123.2, 119.8, 114.3, 114.0; HRMS (ESI) calcd. for $C_{16}H_{12}ClO$ ([M-H]$^-$): 255.0582; found: 255.0579.

3-chloro-2-(1-phenylbuta-1,3-dien-2-yl)phenol (3p')

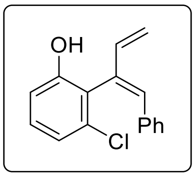

This compound was obtained in 20% yield (10.2 mg) as light yellow oil. Eluent: PE/EA = 10/1. R_f = 0.5; ^{1}H NMR (400 MHz, $CDCl_3$): δ7.23 (t, J = 8.2 Hz, 1H), 7.19-7.16 (m, 3H), 7.08 (dd, *J* = 8.0, 1.0 Hz, 1H), 7.03-6.99 (m, 2H), 6.95 (s, 1H), 6.89 (dd, *J* = 8.2, 0.9 Hz, 1H), 6.72 (dd, *J* = 17.2, 10.3 Hz, 1H), 5.19 (d, *J* = 10.3 Hz, 1H), 5.07 (brs, 1H), 4.87 (d, *J* = 17.0 Hz, 1H); ^{13}C NMR (100 MHz, DMSO-d_6): δ156.0, 140.9, 137.3, 136.5, 132.4, 132.3, 131.8, 128.6, 128.2, 127.2, 123.4, 119.3, 115.6, 115.1; HRMS (ESI) calcd. for $C_{16}H_{12}ClO$ ($[M-H]^-$): 255.0582; found: 255.0580.

4,5-dimethyl-2-(1-phenylbuta-1,3-dien-2-yl)phenol (3q)

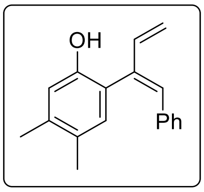

This compound was obtained in 64% yield (32.1 mg) as light yellow oil. Eluent: PE/EA = 10/1. R_f = 0.6; ^{1}H NMR (400 MHz, DMSO-d_6): δ 8.78 (s, 1H), 7.15-7.08 (m, 3H), 7.05-7.01 (m, 2H), 6.72-6.64 (m, 3H), 6.60 (s, 1H), 5.05 (dd, *J* = 10.2, 1.4 Hz, 1H), 4.66 (dd, *J* = 17.0, 1.4 Hz, 1H), 2.18 (s, 3H), 2.08 (s, 3H); ^{13}C NMR (100 MHz, DMSO-d_6): δ 152.6, 141.6, 138.5, 137.0, 136.3, 131.7, 130.8, 128.7, 128.0, 126.9, 126.5, 121.1, 117.0, 115.1, 19.5, 18.4; HRMS (ESI) calcd. for $C_{18}H_{19}O$ ($[M+H]^+$): 251.1431; found: 251.1425.

3-(1-phenylbuta-1,3-dien-2-yl)naphthalen-2-ol (3r)

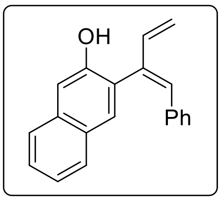

This compound was obtained in 57% yield (31.0 mg) as light yellow oil. Eluent: PE/EA = 10/1. R_f = 0.3; ^{1}H NMR (400 MHz, DMSO-d_6): δ 9.38 (s, 1H), 7.84-7.79 (m,

2H), 7.46-7.42 (m, 1H), 7.31-7.22 (m, 3H), 7.03-6.99 (m, 4H), 6.93-6.88 (m, 2H), 6.84 (dd, *J* = 17.4, 10.5 Hz, 1H), 4.98 (dd, *J* = 9.6, 1.6 Hz, 1H), 4.41 (dd, *J* = 16.6, 1.6 Hz, 1H); ^{13}C NMR (100 MHz, DMSO-*d6*): δ 151.9, 140.7, 136.7, 136.0, 133.3, 132.4, 129.0, 128.3, 128.04, 128.01, 127.96, 127.1, 126.3, 123.7, 122.6, 118.4, 116.1, 114.8; HRMS (ESI) calcd. for $C_{20}H_{15}O$ ([M-H]$^-$): 271.1128; found: 271.1125.

2-(1-(p-tolyl)buta-1,3-dien-2-yl)phenol (3s)

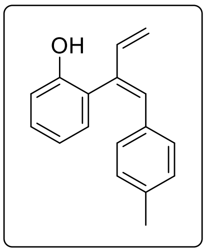

This compound was obtained in 55% yield (26.0 mg) as light yellow oil. Eluent: PE/EA = 10/1. R_f = 0.5; ^{1}H NMR (400 MHz, DMSO-*d6*): δ 9.12 (s, 1H), 7.19 (ddd, *J* = 8.2, 6.1, 3.1 Hz, 1H), 6.91 (dd, *J* = 8.1, 5.6 Hz, 3H), 6.88 (s, 1H), 6.86-6.81 (m, 3H), 6.73-6.65 (m, 2H), 5.04 (dd, *J* = 10.5, 1.3 Hz, 1H), 4.62 (dd, *J* = 17.2, 1.4 Hz, 1H), 2.18 (s, 3H); ^{13}C NMR (100 MHz, DMSO-*d6*): δ 154.8, 141.3, 137.7, 136.4, 134.0, 131.7, 130.4, 128.7, 128.6, 124.2, 119.4, 115.8, 114.5, 20.7; HRMS (ESI) calcd. for $C_{17}H_{15}O$ ([M-H]$^-$): 235.1128; found: 235.1123.

4-fluoro-2-(1-phenylbuta-1,3-dien-2-yl)phenol (3t)

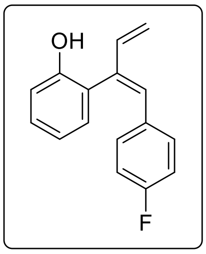

This compound was obtained in 66% yield (31.7 mg) as light yellow oil. Eluent: PE/EA = 10/1. R_f = 0.5; ^{1}H NMR (400 MHz, $CDCl_3$): δ 7.32-7.28 (m, 1H), 7.03 (dd, *J* = 7.7, 1.9 Hz, 1H), 7.00-6.94 (m, 4H), 6.85-6.79 (m, 2H), 6.78 (s, 1H), 6.72 (dd, *J* = 17.2, 10.4 Hz, 1H), 5.21 (d, *J* = 10.4 Hz, 1H), 5.02 (brs, 1H), 4.92 (d, *J* = 17.1 Hz, 1H); ^{13}C NMR (100 MHz, DMSO-*d6*): δ 163.6 (d, *J* = 246.0 Hz), 156.3, 142.6, 140.0, 135.3 (d, *J* = 3.5 Hz), 132.8, 132.5, 132.4, 132.3, 130.6, 126.1, 121.7, 117.5, 116.3,

116.0; ^{19}F NMR (376 MHz, $CDCl_3$): δ -117.61; HRMS (ESI) calcd. for $C_{16}H_{14}FO$ ([M+H]$^+$): 241.1023; found: 241.1023.

2-(1-(4-chlorophenyl)buta-1,3-dien-2-yl)phenol (3u)

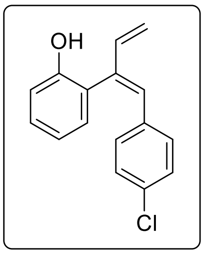

This compound was obtained in 71% yield (36.5 mg) as light yellow oil. Eluent: PE/EA = 10/1. R_f = 0.5; ^{1}H NMR (400 MHz, DMSO-d_6): δ 9.22 (s, 1H), 7.23-7.19 (m, 1H), 7.19-7.15 (m, 2H), 6.98 (d, J = 8.6 Hz, 2H), 6.91 (dd, J = 8.2, 2.5 Hz, 1H), 6.83 (dd, J = 6.9, 4.9 Hz, 2H), 6.75-6.66 (m, 2H), 5.14-5.06 (m, 1H), 4.67 (dd, J = 17.1, 1.5 Hz, 1H); ^{13}C NMR (100 MHz, DMSO-d_6): δ 154.7, 141.0, 139.4, 135.8, 131.4, 130.4, 130.3, 130.2, 129.0, 128.1, 123.6, 119.5, 115.9, 115.7; HRMS (ESI) calcd. for $C_{16}H_{12}ClO$ ([M-H]$^-$): 255.0582; found: 255.0580.

2-(1-(4-bromophenyl)buta-1,3-dien-2-yl)phenol (3v)

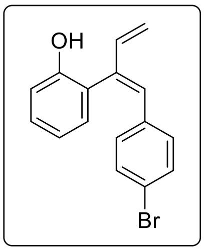

This compound was obtained in 58% yield (34.5 mg) as light yellow oil. Eluent: PE/EA = 10/1. R_f = 0.5; ^{1}H NMR (400 MHz, DMSO-d_6): δ 9.21 (s, 1H), 7.30 (d, J = 8.8 Hz, 2H), 7.24-7.16 (m, 1H), 6.94-6.87 (m, 3H), 6.85-6.82 (m, 2H), 6.70 (dd, J = 17.0, 10.2 Hz, 1H), 6.68 (s, 1H), 5.11 (dd, J = 10.2, 1.1 Hz, 1H), 4.70 (dd, J = 17.3, 1.3 Hz, 1H); ^{13}C NMR (100 MHz, DMSO-d_6): δ 154.7, 141.0, 139.5, 136.2, 131.0, 130.5, 130.4, 130.2, 129.0, 123.6, 120.1, 119.5, 115.9, 115.7; HRMS (ESI) calcd. for $C_{16}H_{12}BrO$ ([M-H]$^-$): 299.0077; found: 299.0076.

2-(1-(4-(trifluoromethyl)phenyl)buta-1,3-dien-2-yl)phenol (3w)

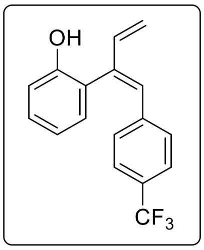

This compound was obtained in 40% yield (23.1 mg) as light yellow oil. Eluent: PE/EA = 10/1. R_f = 0.6; ^{1}H NMR (400 MHz, $CDCl_3$): δ 7.4 (d, *J* = 8.4 Hz, 2H), 7.33-7.28 (m, 1H), 7.08 (d, *J* = 8.2 Hz, 2H), 7.04-6.96 (m, 3H), 6.82 (s, 1H), 6.75 (dd, *J* = 17.1, 10.4 Hz, 1H), 5.29 (d, *J* = 10.4 Hz, 1H), 5.06 (brs, 1H), 5.00 (d, *J* = 17.1 Hz, 1H); ^{13}C NMR (100 MHz, DMSO-d_6): δ 154.7, 141.11, 141.06, 140.8, 130.21, 130.18, 129.2, 129.0, 126.9 (q, *J* = 31.6 Hz), 124.9 (q, *J* = 3.5 Hz), 124.2 (q, *J* = 270.8 Hz), 123.4, 119.5, 116.7, 115.9; ^{19}F NMR (376 MHz, $CDCl_3$): δ -62.62; HRMS (ESI) calcd. for $C_{17}H_{12}F_3O$ ($[M-H]^-$): 289.0845; found: 289.0839.

2-(1-(naphthalen-2-yl)buta-1,3-dien-2-yl)phenol (3x)

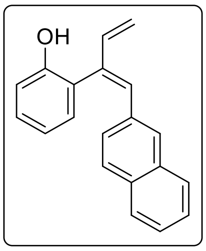

This compound was obtained in 68% yield (36.9 mg) as light yellow oil. Eluent: PE/EA = 10/1. R_f = 0.5; ^{1}H NMR (400 MHz, DMSO-d_6): δ 9.20 (s, 1H), 7.77-7.71 (m, 1H), 7.63-7.57 (m, 2H), 7.55 (s, 1H), 7.43-7.39 (m, 2H), 7.27-7.22 (m, 1H), 7.05 (dd, *J* = 8.7, 1.7 Hz, 1H), 6.95 (dd, *J* = 8.2, 0.8 Hz, 1H), 6.91 (dd, *J* = 7.5, 1.9 Hz, 1H), 6.89-6.83 (m, 2H), 6.78 (dd, *J* = 17.0, 10.4 Hz, 1H), 5.12 (dd, *J* = 10.2, 1.4 Hz,1H), 4.73 (dd, *J* = 17.0, 1.4 Hz, 1H); ^{13}C NMR (100 MHz, DMSO-d_6): δ 154.9, 141.2, 139.1, 134.6, 132.8, 131.9, 131.8, 130.5, 128.9, 128.2, 127.7, 127.3, 127.2, 126.2, 126.0, 124.1, 119.4, 115.8, 115.3; HRMS (ESI) calcd. for $C_{20}H_{15}O$ ($[M-H]^-$): 271.1128; found: 271.1138.

2-(1-(thiophen-2-yl)buta-1,3-dien-2-yl)phenol (3y)

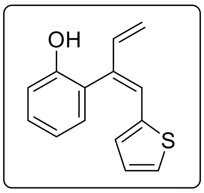

This compound was obtained in 41% yield (18.7 mg) as light yellow oil. Eluent: PE/EA = 10/1. R_f = 0.5; ^{1}H NMR (400 MHz, DMSO-d_6): δ 9.13 (s, 1H), 7.28-7.22 (m, 2H), 6.98 (d, J = 3.6 Hz, 1H), 6.96 (s, 1H), 6.90 (m, 3H), 6.88 (d, J = 1.5 Hz, 1H), 6.70 (dd, J = 17.0, 10.2 Hz, 1H), 5.04 (dd, J = 10.1, 1.3 Hz, 1H), 4.61 (dd, J = 17.0, 1.4 Hz, 1H); ^{13}C NMR (100 MHz, DMSO-d_6): δ 155.1, 140.5, 140.4, 136.4, 131.0, 129.5, 128.9, 127.7, 126.3, 125.4, 122.9, 119.7, 116.2, 114.8; HRMS (ESI) calcd. for $C_{14}H_{13}OS$ ([M+H]$^+$): 229.0682; found: 229.0679.

2-(1-(furan-2-yl)buta-1,3-dien-2-yl)phenol (3z)

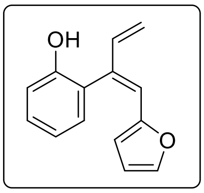

This compound was obtained in 53% yield (22.5 mg) as light yellow oil. Eluent: PE/EA = 10/1. R_f = 0.5; ^{1}H NMR (400 MHz, DMSO-d_6): δ 9.12 (s, 1H), 7.52 (d, J = 1.6 Hz, 1H), 7.22 (dt, J = 8.2, 4.5 Hz, 1H), 6.93 (d, J = 8.2 Hz, 1H), 6.89-6.86 (m, 2H), 6.71 (dd, J = 17.0, 10.3 Hz, 1H), 6.60 (s, 1H), 6.29 (dd, J = 3.4, 1.8 Hz, 1H), 5.34 (d, J = 3.2 Hz, 1H), 5.07 (dd, J = 10.2, 0.9 Hz, 1H), 4.67 (d, J = 17.0 Hz, 1H); ^{13}C NMR (100 MHz, DMSO-d_6): δ 154.4, 152.3, 142.2, 140.0, 137.0, 130.1, 128.9, 123.9, 120.0, 119.4, 115.9, 115.4, 111.9, 108.7; HRMS (ESI) calcd. for $C_{14}H_{11}O_2$ ([M-H]$^-$): 211.0764; found: 211.0757.

General procedure for the synthesis of allyl ethers 4a-z:

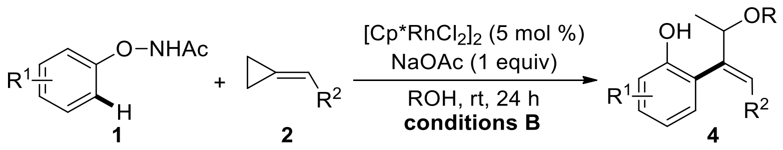

The mixture of *N*-phenoxyacetamides **1**(0.2 mmol, 1.0 equiv), methylenecyclopropanes **2** (0.4 mmol, 2.0 equiv), $[Cp^*RhCl_2]_2$ (5 mol %) and NaOAc (0.2 mmol, 1.0 equiv) in an alcoholic solvent (1.0 mL) was stirred at room

temperature for 24 h without exclusion of air or moisture. Afterwards, the solvent was removed under reduced pressure, and theresulted mixture was purified by preparative TLC to afford the corresponding derivatives 4**a-z.**

Characterization of products 4:

(*E*)-2-(3-methoxy-1-phenylbut-1-en-2-yl)phenol (4a)

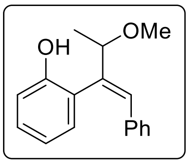

This compound was obtained in 58% yield (29.5 mg) as light yellow oil. Eluent: PE/EA = 10/1. R_f = 0.55; ^{1}H NMR (400 MHz, DMSO-d_6): δ 9.25 (s, 1H), 7.17-7.06 (m, 4H), 7.02-6.98 (m, 2H), 6.87 (dd, *J* = 8.1, 2.4 Hz, 2H), 6.77 (t, *J* = 7.4 Hz, 1H), 6.62 (s, 1H), 4.01 (q, *J* = 6.2 Hz, 1H), 3.36 (s, 3H), 1.15 (d, *J* = 6.3 Hz, 3H); ^{13}C NMR (100 MHz, DMSO-d_6): δ 154.8, 140.5, 136.8, 130.4, 128.6, 128.4, 127.9, 126.6, 126.1, 125.4, 119.2, 115.6, 81.0, 55.8, 20.2; HRMS (ESI) calcd. for $C_{17}H_{17}O_2$ ([M-H]$^-$): 253.1234; found: 253.1230.

(*E*)-2-(3-methoxy-1-phenylbut-1-en-2-yl)-4-methylphenol (4b)

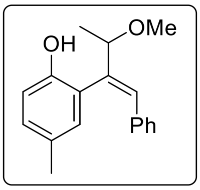

This compound was obtained in 56% yield (29.8 mg) as light yellow oil. Eluent: PE/EA = 10/1. R_f = 0.7; ^{1}H NMR (400 MHz, $CDCl_3$): δ 7.67 (brs, 1H), 7.13-7.11 (m, 3H), 7.00-6.97 (m, 3H), 6.86 (d, *J* = 8.2 Hz, 1H), 6.72 (s, 1H), 6.64 (d, *J* = 1.8 Hz, 1H), 4.09 (q, *J* = 6.4 Hz, 1H), 3.49 (s, 3H), 2.13 (s, 3H), 1.24 (d, *J* = 6.4 Hz, 3H); ^{13}C NMR (100 MHz, $CDCl_3$): δ 152.1, 138.0, 135.8, 132.7, 131.7, 130.2, 129.6, 129.5, 128.2, 127.6, 123.9, 116.9, 84.7, 56.3, 20.5, 18.9; HRMS (ESI) calcd. for $C_{18}H_{19}O_2$ ([M-H]$^-$): 267.1390; found: 267.1390.

(*E*)-4-(*tert*-butyl)-2-(3-methoxy-1-phenylbut-1-en-2-yl)phenol (4c)

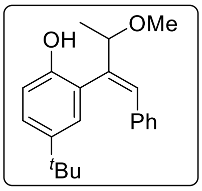

This compound was obtained in 65% yield (40.3 mg) as light yellow oil. Eluent: PE/EA = 10/1. R_f = 0.55; ^{1}H NMR (400 MHz, $CDCl_3$): δ 8.21 (brs, 1H), 7.18 (dd, *J* = 8.5, 2.5 Hz, 1H), 7.11-7.06 (m, 3H), 6.94 (dd, *J* = 6.8, 2.8 Hz, 2H), 6.88 (d, *J* = 8.4 Hz, 1H), 6.77 (d, *J* = 2.5 Hz, 1H), 6.72 (s, 1H), 4.12 (q, *J* = 6.4 Hz, 1H), 3.51 (s, 3H), 1.27 (d, *J* = 6.4 Hz, 3H), 1.06 (s, 9H); ^{13}C NMR (100 MHz, $CDCl_3$): δ 152.3, 142.8, 138.8, 136.1, 133.4, 129.61, 129.55, 128.0, 127.4, 126.3, 122.9, 116.5, 84.7, 56.2, 33.9, 31.4, 18.8; HRMS (ESI) calcd. for $C_{21}H_{25}O_2$ ($[M-H]^-$): 309.1860; found: 309.1857.

(*E*)-4-fluoro-2-(3-methoxy-1-phenylbut-1-en-2-yl)phenol (4d)

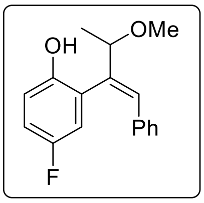

This compound was obtained in 50% yield (27.3 mg) as light yellow oil. Eluent: PE/EA = 10/1. R_f = 0.5; ^{1}H NMR (400 MHz, $CDCl_3$): δ 7.98 (brs, 1H), 7.17-7.13 (m, 3H), 7.02-6.96 (m, 2H), 6.91-6.90 (m, 1H), 6.89-6.87 (m, 1H), 6.76 (s, 1H), 6.55-6.50 (m, 1H), 4.11 (t, *J* = 6.4 Hz, 1H), 3.50 (s, 3H), 1.24 (d, *J* = 6.4 Hz, 3H); ^{13}C NMR (100 MHz, $CDCl_3$): δ 156.8 (d, *J* = 237.7 Hz), 155.7, 150.9, 136.8, 135.3, 133.9, 129.5, 128.3, 128.0, 125.1(d, *J* = 6.8 Hz), 118.1 (d, *J* = 8.4 Hz), 117.6 (d, *J* = 23.1 Hz), 116.1 (d, *J* = 22.9 Hz), 84.6, 56.3, 18.6; ^{19}F NMR (376 MHz, $CDCl_3$): δ-124.94; HRMS (ESI) calcd. for $C_{17}H_{16}FO_2$ ($[M-H]^-$): 271.1140; found: 271.1140.

(*E*)-4-chloro-2-(3-methoxy-1-phenylbut-1-en-2-yl)phenol (4e)

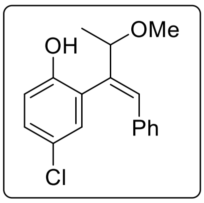

This compound was obtained in 46% yield (26.4 mg) as light yellow oil. Eluent: PE/EA = 10/1. R_f = 0.55; ^{1}H NMR (400 MHz, $CDCl_3$): δ 8.20 (brs, 1H), 7.19-7.13 (m, 4H), 7.02-6.94 (m, 2H), 6.91 (d, *J* = 8.7 Hz, 1H), 6.79 (d, *J* = 2.6 Hz, 1H), 6.76 (s,

1H), 4.11 (q, J = 6.4 Hz, 1H), 3.49 (s, 3H), 1.24 (d, J = 6.4 Hz, 3H); ^{13}C NMR (100 MHz, $CDCl_3$): δ 153.6, 136.5, 135.2, 134.1, 131.0, 129.5, 129.4, 128.3, 128.0, 125.7, 124.9, 118.7, 84.6, 56.3, 18.6; HRMS (ESI) calcd. for $C_{17}H_{16}ClO_2$ ($[M-H]^-$): 287.0844; found: 287.0845.

(*E*)-4-bromo-2-(3-methoxy-1-phenylbut-1-en-2-yl)phenol (4f)

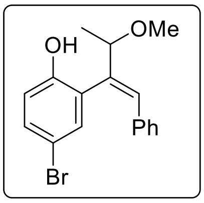

This compound was obtained in 42% yield (27.9 mg) as light yellow oil. Eluent: PE/EA = 10/1. R_f = 0.55; ^{1}H NMR (400 MHz, $CDCl_3$): δ 8.25 (brs, 1H), 7.28 (dd, J = 8.7, 2.5 Hz, 1H), 7.17-7.15 (m, 3H), 7.00-6.95 (m, 2H), 6.93 (d, J = 2.5 Hz, 1H), 6.86 (d, J = 8.7 Hz, 1H), 6.76 (s, 1H), 4.11 (q, J = 6.4 Hz, 1H), 3.49 (s, 3H), 1.24 (d, J = 6.4 Hz, 3H); ^{13}C NMR (100 MHz, $CDCl_3$): δ 154.1, 136.4, 135.2, 134.1, 133.9, 132.4, 129.5, 128.3, 128.0, 126.2, 119.2, 112.1, 84.6, 56.3, 18.6; HRMS (ESI) calcd. for $C_{17}H_{16}BrO_2$ ($[M-H]^-$): 331.0339; found: 331.0340.

(*E*)-4-iodo-2-(3-methoxy-1-phenylbut-1-en-2-yl)phenol (4g)

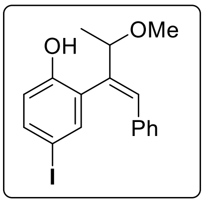

This compound was obtained in 40% yield (30.5 mg) as light yellow oil. Eluent: PE/EA = 10/1. R_f = 0.55; ^{1}H NMR (400 MHz, $CDCl_3$): δ 8.29 (brs, 1H), 7.46 (dd, J = 8.6, 2.3 Hz, 1H), 7.17-7.15 (m, 3H), 7.11 (d, J = 2.3 Hz, 1H), 6.99-6.95 (m, 2H), 6.76-6.73 (m, 2H), 4.10 (q, J = 6.4 Hz, 1H), 3.49 (s, 3H), 1.24 (d, J = 6.4 Hz, 3H); ^{13}C NMR (100 MHz, $CDCl_3$): δ 154.9, 139.8, 138.3, 136.3, 135.2, 134.1, 129.6, 128.3, 128.0, 126.8, 119.7, 84.6, 81.8, 56.3, 18.6; HRMS (ESI) calcd. for $C_{17}H_{16}IO_2$ ($[M-H]^-$): 379.0200; found: 379.0202.

(*E*)-3-(3-methoxy-1-phenylbut-1-en-2-yl)-[1,1'-biphenyl]-4-ol (4h)

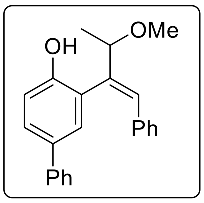

This compound was obtained in 57% yield (37.6 mg) as light yellow oil. Eluent: PE/EA = 10/1. R_f = 0.5; ^{1}H NMR (400 MHz, DMSO-d_6): δ 9.47 (s, 1H), 7.49-7.45 (m, 3H), 7.37 (t, J = 8.0 Hz, 2H), 7.24 (t, J = 7.6 Hz, 1H), 7.18-7.05 (m, 6H), 6.97 (d, J = 8.4 Hz, 1H), 6.68 (s, 1H), 4.08 (q, J = 6.3 Hz, 1H), 3.40 (s, 3H), 1.20 (d, J = 6.4 Hz, 3H); ^{13}C NMR (100 MHz, DMSO-d_6): δ 154.7, 140.2, 140.0, 136.8, 131.1, 128.8, 128.5, 128.4, 128.0, 126.9, 126.7, 126.5, 126.4, 125.9, 116.2, 81.0, 55.8, 20.2; HRMS (ESI) calcd. for $C_{23}H_{21}O_2$ ([M-H]$^-$): 329.1547; found: 329.1549.

(*E*)-2-(3-methoxy-1-phenylbut-1-en-2-yl)-4-(trifluoromethyl)phenol (4i)

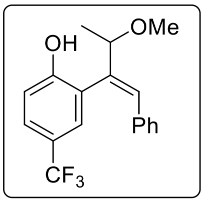

This compound was obtained in 29% yield (18.7 mg) as light yellow oil. Eluent: PE/EA = 10/1. R_f = 0.5; ^{1}H NMR (400 MHz, $CDCl_3$): δ 8.88 (brs, 1H), 7.44 (dd, J = 8.5, 2.2 Hz, 1H), 7.16-7.11 (m, 3H), 7.06 (d, J = 2.0 Hz, 1H), 7.03 (d, J = 8.4 Hz, 1H),6.94-6.91 (m, 2H), 6.81 (s, 1H), 4.15 (q, J = 6.4 Hz, 1H), 3.52 (s, 3H), 1.25 (d, J = 6.4 Hz, 3H); ^{13}C NMR (100 MHz, $CDCl_3$): δ 157.9, 136.5, 135.1, 134.9, 129.6, 129.5, 128.3, 128.0, 126.8(q, J = 3.7 Hz), 124.4 (q, J = 270.0 Hz), 122.6 (q, J = 32.5 Hz), 117.7, 84.5, 56.3, 18.6; ^{19}F NMR (376 MHz, $CDCl_3$): δ -61.49; HRMS (ESI) calcd. for $C_{18}H_{16}F_3O_2$ ([M-H]$^-$): 321.1108; found: 321.1109.

methyl (*E*)-4-hydroxy-3-(3-methoxy-1-phenylbut-1-en-2-yl)benzoate (4j)

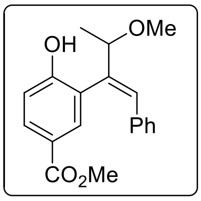

This compound was obtained in 21% yield (13.3 mg) as light yellow oil. Eluent: PE/EA = 10/1. R_f = 0.3; ^{1}H NMR (400 MHz, $CDCl_3$): δ 8.73 (brs, 1H), 7.90 (dd, J = 8.6, 2.2 Hz, 1H), 7.56 (d, J = 2.1 Hz, 1H), 7.14-7.10 (m, 3H), 6.99 (d, J = 8.5 Hz, 1H),

6.97-6.93 (m, 2H), 6.80 (s, 1H), 4.15 (q, J = 6.4 Hz, 1H), 3.78 (s, 3H), 3.52 (s, 3H), 1.24 (d, J = 6.4 Hz, 3H); ^{13}C NMR (100 MHz, $CDCl_3$): δ 166.9, 159.3, 136.7, 135.3, 134.3, 134.0, 131.5, 129.5, 128.3, 127.9, 124.1, 122.5, 117.3, 84.6, 56.3, 51.9, 18.5; HRMS (ESI) calcd. for $C_{19}H_{19}O_4$ ([M-H]$^-$): 311.1289; found: 311.1288.

(*E*)-2-(3-methoxy-1-phenylbut-1-en-2-yl)-5-methylphenol (4k)

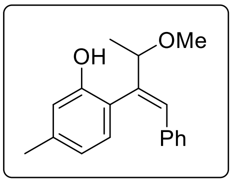

This compound was obtained in 56% yield (30.0 mg) as light yellow oil. Eluent: PE/EA = 10/1. R_f = 0.6; ^{1}H NMR (400 MHz, $CDCl_3$): δ 7.99 (brs, 1H), 7.16-7.10 (m, 3H), 7.01-6.98 (m, 2H), 6.79 (s, 1H), 6.72-6.69 (m, 2H), 6.56 (d, J = 7.7 Hz, 1H), 4.09 (q, J = 6.4 Hz, 1H), 3.49 (s, 3H), 2.30 (s, 3H), 1.23 (d, J = 6.4 Hz, 3H); ^{13}C NMR (100 MHz, $CDCl_3$): δ 154.3, 139.7, 137.9, 135.9, 132.9, 131.5, 129.5, 128.1, 127.5, 121.4, 121.0, 117.7, 84.8, 56.2, 21.4, 18.8; HRMS (ESI) calcd. for $C_{18}H_{19}O_2$ ([M-H]$^-$): 267.1390; found: 267.1391.

(*E*)-5-fluoro-2-(3-methoxy-1-phenylbut-1-en-2-yl)phenol (4l)

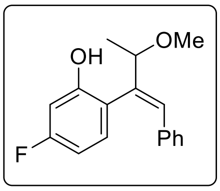

This compound was obtained in 35% yield (19.0 mg) as light yellow oil. Eluent: PE/EA = 10/1. R_f = 0.5; ^{1}H NMR (400 MHz, $CDCl_3$): δ 8.35 (s, 1H), 7.17-7.12 (m, 4H), 7.02-6.98 (m, 2H), 6.90 (s, 1H), 6.79 (dd, J = 8.3, 0.9 Hz, 1H), 6.53-6.48 (m, 1H), 4.21 (q, J = 6.4 Hz, 1H), 3.52 (s, 3H), 1.24 (d, J = 6.4 Hz, 3H); ^{13}C NMR (100 MHz, $CDCl_3$): δ 160.2 (d, J = 243.8 Hz), 156.3 (d, J = 5.1 Hz), 135.9, 135.7, 130.5, 129.9 (d, J = 10.6 Hz), 128.7, 128.3, 128.0, 112.9 (d, J = 2.7 Hz), 112.8 (d, J = 19.4 Hz), 107.3 (d, J = 23.2 Hz), 84.8, 56.2, 18.0; ^{19}F NMR (376 MHz, $CDCl_3$): δ -113.52; HRMS (ESI) calcd. for $C_{17}H_{16}FO_2$ ([M-H]$^-$): 271.1140; found: 271.1134.

(*E*)-5-chloro-2-(3-methoxy-1-phenylbut-1-en-2-yl)phenol (4m)

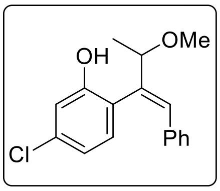

This compound was obtained in 43% yield (24.8 mg) as light yellow oil. Eluent: PE/EA = 10/1. R_f = 0.6; ^{1}H NMR (400 MHz, DMSO-d_6): δ 9.80 (s, 1H), 7.17-7.09 (m, 3H), 7.03-6.99 (m, 2H), 6.90-6.86 (m, 2H), 6.83 (dd, J = 8.1, 2.0 Hz, 1H), 6.65 (s, 1H), 3.99 (q, J = 6.3 Hz, 1H), 3.34 (s, 3H), 1.13 (d, J = 6.4 Hz, 3H); ^{13}C NMR (100 MHz, DMSO-d_6): δ 156.0, 139.0, 136.6, 132.2, 131.8, 128.3, 128.0, 127.2, 126.8, 124.5, 119.1, 115.3, 80.9, 55.8, 20.0; HRMS (ESI) calcd. for $C_{17}H_{16}ClO_2$ ([M-H]$^-$): 287.0844; found: 287.0844.

(*E*)-2-(3-methoxy-1-phenylbut-1-en-2-yl)-4,5-dimethylphenol (4n)

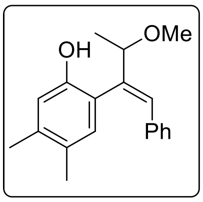

This compound was obtained in 51% yield (28.7 mg) as light yellow oil. Eluent: PE/EA = 10/1. R_f = 0.65; ^{1}H NMR (400 MHz, $CDCl_3$): δ 7.57 (brs, 1H), 7.15-7.10 (m, 3H), 7.04-6.99 (m, 2H), 6.76 (s, 1H), 6.69 (s, 1H), 6.59 (s, 1H), 4.07 (q, J = 6.4 Hz, 1H), 3.48 (s, 3H), 2.21 (s, 3H), 2.03 (s, 3H), 1.23 (d, J = 6.4 Hz, 3H); ^{13}C NMR (100 MHz, $CDCl_3$): δ 152.1, 138.0, 138.0, 136.0, 132.3, 132.1, 129.5, 128.3, 128.2, 127.5, 121.1, 118.2, 84.8, 56.2, 19.8, 19.0, 18.7; HRMS (ESI) calcd. for $C_{19}H_{21}O_2$ ([M-H]$^-$): 281.1547; found: 281.1547.

(*E*)-2-(3-methoxy-1-phenylbut-1-en-2-yl)-6-methylphenol (4o)

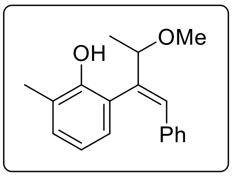

This compound was obtained in 48% yield (25.8 mg) as light yellow oil. Eluent: PE/EA = 10/1. R_f = 0.6; ^{1}H NMR (400 MHz, DMSO-d_6): δ 8.20 (s, 1H), 7.14-7.04 (m, 4H), 6.98 (dd, J = 7.7, 1.4 Hz, 2H), 6.74-6.66 (m, 3H), 4.04 (q, J = 6.3 Hz, 1H), 3.38 (s, 3H), 2.19 (s, 3H), 1.14 (d, J = 6.4 Hz, 3H); ^{13}C NMR (100 MHz, DMSO-d_6): δ

152.3, 139.8, 136.5, 130.1, 128.6, 128.0, 127.9, 126.8, 125.5, 124.7, 119.6, 81.4, 55.8, 19.7, 16.6; HRMS (ESI) calcd. for $C_{18}H_{19}O_2$ ([M-H]$^-$): 267.1390; found: 267.1388.

(*E*)-3-(3-methoxy-1-phenylbut-1-en-2-yl)naphthalen-2-ol (4p)

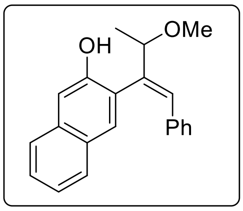

This compound was obtained in 54% yield (32.8 mg) as light yellow oil. Eluent: PE/EA = 10/1. R_f = 0.35; ^{1}H NMR (400 MHz, $CDCl_3$): δ 7.97 (brs, 1H), 7.70 (d, *J* = 8.3 Hz, 1H), 7.56 (d, *J* = 8.2 Hz, 1H), 7.41-7.35 (m, 3H), 7.2-7.21 (m, 1H), 7.08-7.02 (m, 3H), 6.99-6.95 (m, 2H), 6.86 (s, 1H), 4.18 (q, *J* = 6.3 Hz, 1H), 3.53 (s, 3H), 1.25 (d, *J* = 6.5 Hz, 3H); ^{13}C NMR (100 MHz, $CDCl_3$): δ 152.7, 137.6, 135.6, 134.9, 133.7, 130.7, 129.5, 128.9, 128.3, 127.8, 127.7, 127.3, 126.5, 126.4, 123.4, 111.7, 84.6, 56.4, 18.8; HRMS (ESI) calcd. for $C_{21}H_{19}O_2$ ([M-H]$^-$): 303.1390; found: 303.1393.

(*E*)-2-(3-ethoxy-1-phenylbut-1-en-2-yl)phenol (4q)

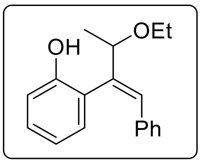

This compound was obtained in 57% yield (30.5 mg) as light yellow oil. Eluent: PE/EA = 10/1. R_f = 0.6; ^{1}H NMR (400 MHz, $CDCl_3$): δ 8.44 (brs, 1H), 7.22-7.17 (m, 1H), 7.13-7.09 (m, 3H), 6.99-6.94 (m, 3H), 6.80 (dd, *J* = 7.6, 1.7 Hz, 1H), 6.75-6.70 (m, 2H), 4.23 (q, *J* = 6.4 Hz, 1H), 3.84-3.75 (m, 1H), 3.66-3.58 (m, 1H), 1.30 (t, *J* = 7.0 Hz, 3H), 1.24 (d, *J* = 6.4 Hz, 3H); ^{13}C NMR (100 MHz, $CDCl_3$): δ 154.9, 138.6, 135.9, 133.0, 131.9, 129.6, 128.1, 127.5, 124.2, 120.4, 117.3, 83.0, 64.3, 19.1, 15.2; HRMS (ESI) calcd. for $C_{18}H_{19}O_2$ ([M-H]$^-$): 267.1390; found: 267.1385.

(*E*)-2-(3-butoxy-1-phenylbut-1-en-2-yl)phenol (4r)

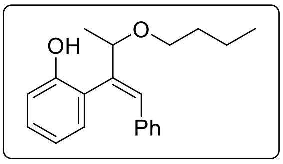

This compound was obtained in 35% yield (20.8 mg) as light yellow oil. Eluent: PE/EA = 10/1. R_f = 0.6; ^{1}H NMR (400 MHz, $CDCl_3$): δ 8.48 (brs, 1H), 7.22-7.16 (m, 1H), 7.13-7.08 (m, 3H), 6.98-6.93 (m, 3H), 6.79 (dd, *J* = 7.6, 1.7 Hz, 1H), 6.74-6.67 (m, 2H), 4.21 (q, *J* = 6.4 Hz, 1H), 3.73 (dt, *J* = 9.1, 6.6 Hz, 1H), 3.54 (dt, *J* = 9.1, 6.8 Hz, 1H), 1.69-1.61 (m, 2H), 1.48-1.37 (m, 2H), 1.22 (d, *J* = 6.4 Hz, 3H), 0.93 (t, *J* = 7.4 Hz, 3H); ^{13}C NMR (100 MHz, $CDCl_3$): δ154.9, 138.7, 136.0, 133.0, 131.9, 129.7, 129.6, 128.1, 127.5, 124.2, 120.4, 117.3, 83.3, 68.9, 31.8, 19.5, 19.0, 14.0; HRMS (ESI) calcd. for $C_{20}H_{23}O_2$ ($[M-H]^-$): 295.1703; found: 295.1702.

(*E*)-2-(3-methoxy-1-(p-tolyl)but-1-en-2-yl)phenol (4s)

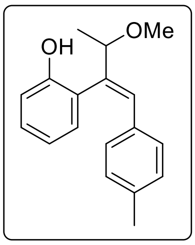

This compound was obtained in 64% yield (34.3 mg) as light yellow oil. Eluent: PE/EA = 10/1. R_f = 0.6; ^{1}H NMR (400 MHz, $CDCl_3$): δ 8.05 (brs, 1H), 7.23-7.17 (m, 1H), 6.97-6.92 (m, 3H), 6.87-6.82 (m, 3H), 6.75 (t, *J* = 7.4 Hz, 1H), 6.71 (s, 1H), 4.10 (q, *J* = 6.4 Hz, 1H), 3.49 (s, 3H), 2.24 (s, 3H), 1.23 (d, *J* = 6.4 Hz, 3H); ^{13}C NMR (100 MHz, $CDCl_3$): δ 154.6, 137.6, 136.9, 133.2, 132.9, 131.7, 129.5, 128.94, 128.93, 124.3, 120.5, 117.2, 84.8, 56.2, 21.3, 18.7; HRMS (ESI) calcd. for $C_{18}H_{19}O_2$ ($[M-H]^-$): 267.1391; found: 267.1389.

(*E*)-2-(1-(4-fluorophenyl)-3-methoxybut-1-en-2-yl)phenol (4t)

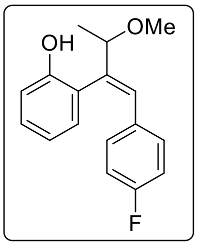

This compound was obtained in 53% yield (28.9 mg) as light yellow oil. Eluent: PE/EA = 10/1. R_f = 0.55; ^{1}H NMR (400 MHz, $CDCl_3$): δ 8.10 (brs, 1H), 7.23-7.17 (m, 1H), 6.97 (dd, *J* = 8.2, 0.9 Hz, 1H), 6.95-6.90 (m, 2H), 6.82-6.78 (m, 3H), 6.77-6.72 (m, 1H), 6.70 (s, 1H), 4.11 (q, *J* = 6.4 Hz, 1H), 3.50 (s, 3H), 1.23 (d, *J* = 6.4 Hz, 3H); ^{13}C NMR (100 MHz, $CDCl_3$): δ 162.0 (d, *J* = 248.1 Hz), 154.7, 137.9, 131.9, 131.8 (d,

J = 27.5 Hz), 131.2 (d, J = 7.8 Hz), 129.7, 123.9, 120.6, 117.3, 115.2, 115.0, 84.7, 56.2, 18.6; ^{19}F NMR (376 MHz, $CDCl_3$): δ -113.64; HRMS (ESI) calcd. for $C_{17}H_{16}FO_2$ ($[M-H]^-$): 271.1140; found: 271.1140.

(*E*)-2-(1-(4-chlorophenyl)-3-methoxybut-1-en-2-yl)phenol (4u)

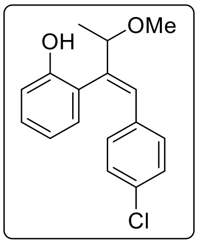

This compound was obtained in 42% yield (24.2 mg) as light yellow oil. Eluent: PE/EA = 10/1. R_f = 0.4; ^{1}H NMR (400 MHz, $CDCl_3$): δ 8.10 (brs, 1H), 7.23-7.17 (m, 1H), 7.10-7.05 (m, 2H), 6.97 (d, J = 8.0 Hz, 1H), 6.90-6.86 (m, 2H), 6.80-6.71 (m, 2H), 6.68 (s, 1H), 4.11 (q, J = 6.4 Hz, 1H), 3.49 (s, 3H), 1.23 (d, J = 6.4 Hz, 3H); ^{13}C NMR (100 MHz, $CDCl_3$): δ 154.6, 138.9, 134.3, 133.3, 131.8, 131.6, 130.8, 129.8, 128.4, 123.8, 120.6, 117.4, 84.5, 56.3, 18.6; HRMS (ESI) calcd. for $C_{17}H_{16}ClO_2$ ($[M-H]^-$): 287.0844; found: 287.0843.

(*E*)-2-(1-(4-bromophenyl)-3-methoxybut-1-en-2-yl)phenol (4v)

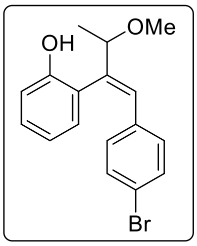

This compound was obtained in 61% yield (40.5 mg) as light yellow oil. Eluent: PE/EA = 10/1. R_f = 0.4; ^{1}H NMR (400 MHz, $CDCl_3$): δ 8.10 (brs, 1H), 7.26-7.17 (m, 3H), 6.98-6.95 (m, 1H), 6.83-6.79 (m, 2H), 6.79-6.71 (m, 2H), 6.66 (s, 1H), 4.11 (q, J = 6.4 Hz, 1H), 3.49 (s, 3H), 1.23 (d, J = 6.4 Hz, 3H); ^{13}C NMR (100 MHz, $CDCl_3$): δ 154.6, 139.0, 134.8, 131.8, 131.6, 131.3, 131.1, 129.8, 123.7, 121.6, 120.6, 117.4, 84.6, 56.3, 18.5; HRMS (ESI) calcd. for $C_{17}H_{16}BrO_2$ ($[M-H]^-$): 331.0339; found: 331.0339.

(*E*)-2-(3-methoxy-1-(4-(trifluoromethyl)phenyl)but-1-en-2-yl)phenol (4w)

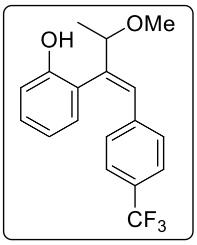

This compound was obtained in 36% yield (23.3 mg) as light yellow oil. Eluent: PE/EA = 10/1. R_f = 0.45; ^{1}H NMR (400 MHz, $CDCl_3$): δ 8.11 (brs, 1H), 7.37 (d, *J* = 8.3 Hz, 2H), 7.25-7.19 (m, 1H), 7.05 (d, *J* = 8.3 Hz, 2H), 6.98 (d, *J* = 8.2 Hz, 1H), 6.77-6.73 (m, 3H), 4.15 (q, *J* = 6.4 Hz, 1H), 3.51 (s, 3H), 1.26 (d, *J* = 6.4 Hz, 3H); ^{13}C NMR (100 MHz, $CDCl_3$): δ 154.6, 140.7, 139.4, 131.65, 131.59, 130.0, 129.7, 129.2 (q, *J* = 32.5 Hz), 125.0 (q, *J* = 3.8 Hz), 124.1 (q, *J* = 270.3 Hz), 123.5, 120.7, 117.5, 84.5, 56.4, 18.5; ^{19}F NMR (376 MHz, $CDCl_3$): δ -62.57; HRMS (ESI) calcd. for $C_{18}H_{16}F_3O_2$ ($[M-H]^-$): 321.1108; found: 321.1109.

(*E*)-2-(3-methoxy-1-(naphthalen-2-yl)but-1-en-2-yl)phenol (4x)

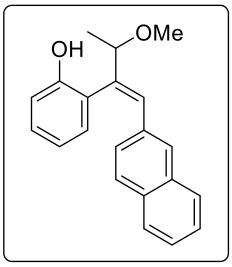

This compound was obtained in 48% yield (29.3 mg) as light yellow oil. Eluent: PE/EA = 10/1. R_f = 0.5; ^{1}H NMR (400 MHz, $CDCl_3$): δ 8.23 (brs, 1H), 7.71-7.65 (m, 1H), 7.63-7.60 (m, 1H), 7.53-7.50 (m, 2H), 7.40-7.35 (m, 2H), 7.25-7.17 (m, 1H), 7.03-6.93 (m, 2H), 6.89 (s, 1H), 6.83 (dd, *J* = 7.6, 1.3 Hz, 1H), 6.71 (t, *J* = 7.6 Hz, 1H), 4.16 (q, *J* = 6.4 Hz, 1H), 3.52 (s, 3H), 1.27 (d, *J* = 6.4 Hz, 3H); ^{13}C NMR (100 MHz, $CDCl_3$): δ 154.8, 138.3, 133.4, 133.3, 133.2, 132.7, 132.0, 130.0, 129.3, 128.2, 127.6, 127.5, 127.0, 126.2, 126.1, 124.1, 120.5, 117.3, 84.7, 56.2, 18.7; HRMS (ESI) calcd. for $C_{21}H_{19}O_2$ ($[M-H]^-$): 303.1391; found: 303.1392.

(*E*)-2-(3-methoxy-1-(thiophen-2-yl)but-1-en-2-yl)phenol (4y)

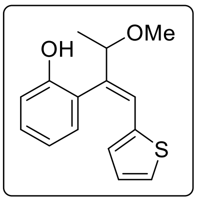

This compound was obtained in28% yield (14.6 mg) as light yellow oil. Eluent: PE/EA = 10/1. R_f = 0.5; ^{1}H NMR (400 MHz, $CDCl_3$): δ 7.71 (brs, 1H), 7.34-7.28 (m, 1H), 7.07 (d, *J* = 5.1 Hz, 1H), 7.04-6.99 (m, 2H), 6.95-6.92 (m, 2H), 6.91-6.88 (m, 1H), 6.85 (dd, *J* = 5.0, 3.7 Hz, 1H), 4.11 (q, *J* = 6.3 Hz, 1H), 3.47 (s, 3H), 1.26 (d, *J* = 6.2 Hz, 3H); ^{13}C NMR (100 MHz, $CDCl_3$): δ 154.5, 139.2, 135.4, 131.9, 131.8, 130.3, 129.6, 127.5, 126.3, 126.0, 121.0, 117.5, 84.2, 56.3, 18.5; HRMS (ESI) calcd. for $C_{15}H_{14}O_2S$([M-H]$^-$): 259.0798; found: 259.0799.

(*E*)-2-(1-(furan-2-yl)-3-methoxybut-1-en-2-yl)phenol (4z)

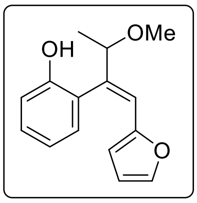

This compound was obtained in 43% yield (21.1 mg) as light yellow oil. Eluent: PE/EA = 10/1. R_f = 0.5; ^{1}H NMR (400 MHz, $CDCl_3$): δ 7.76 (brs, 1H), 7.29-7.23 (m, 2H), 7.00 (dd, *J* = 7.8, 1.3 Hz, 2H), 6.90 (dd, *J* = 7.4, 0.8 Hz, 1H), 6.65 (s, 1H), 6.20-6.17 (m, 1H), 5.52 (d, *J* = 3.5 Hz, 1H), 4.09 (q, *J* = 6.3 Hz, 1H), 3.47 (s, 3H), 1.25 (d, *J* = 6.3 Hz, 3H); ^{13}C NMR (100 MHz, $CDCl_3$): δ 153.9, 151.2, 142.0, 135.7, 131.2, 129.8, 124.1, 121.6, 120.6, 117.3, 111.8, 110.1, 84.0, 56.4, 18.6; HRMS (ESI) calcd. for $C_{15}H_{15}O_3$ ([M-H]$^-$): 243.1027; found: 243.1022.

General procedure for the synthesis of 2,3-dihydrobenzofuran5:

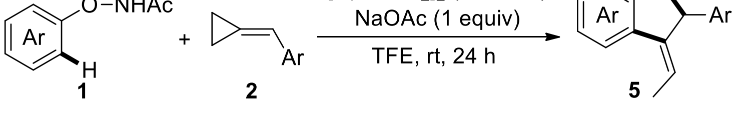

The mixture of *N*-phenoxyacetamides **1** (0.2 mmol, 1.0 equiv), methylenecyclopropanes **2** (0.4 mmol, 2.0 equiv), $[Cp^*RhCl_2]_2$ (5 mol %) and NaOAc (0.2 mmol, 1.0 equiv) in TFE (1.0 mL) was stirred at room temperature for 24 h without exclusion of air or moisture. Afterwards, the solvent was removed under

reduced pressure, and theresulted mixture was purified by preparative TLC to afford the corresponding 2,3-dihydrobenzofuran derivatives **5.**

Characterization of products 5:

(*E*)-3-ethylidene-2-(4-methoxyphenyl)-5-methyl-2,3-dihydrobenzofuran (5a)&(*E*)-3-(4-methoxybenzylidene)-2,5-dimethyl-2,3-dihydrobenzofuran (5a')

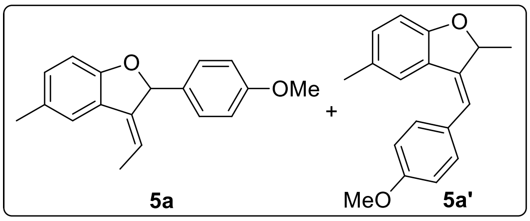

This compound was obtained in 59% yield (31.4 mg) as light yellow oil. An inseparable mixture of two isomers was obtained; the ratio was determined to be 7.5/1 by ^{1}H-NMR analysis. Eluent: PE/EA = 10/1. R_f = 0.75; ^{1}H NMR (400 MHz, $CDCl_3$): δ 7.38 (s, 1H), 7.30-7.24 (m, 2+0.26=2.26H), 7.17 (s, 0.13H), 7.00 (d, *J* = 8.0 Hz, 1H), 6.95 (d, *J* = 8.4 Hz, 0.13H), 6.89-6.84 (m, 2+0.26=2.26H), 6.77 (d, *J* = 8.2 Hz, 1H), 6.69 (d, *J* = 8.2 Hz, 0.13H), 6.11-6.08 (m, 0.13H), 6.00 (qd, *J* = 7.3, 2.9 Hz, 0.13H), 5.95-5.91 (m, 1H), 5.27 (qd, *J* = 7.3, 2.4 Hz, 1H), 3.774 (s, 3H), 3.766 (s, 0.4H), 2.33 (s, 3H), 2.30 (s, 0.4H), 1.98 (dd, *J* = 7.3, 2.5 Hz, 3H), 1.49 (dd, *J* = 7.2, 1.7 Hz, 0.4H); ^{13}C NMR (100 MHz, $CDCl_3$): δ 160.7, 159.9, 159.8, 159.4, 140.1, 139.6, 133.4, 131.7, 130.3, 130.2, 129.9, 129.1, 128.9, 126.7, 126.2, 125.0, 120.2, 117.5, 114.2, 114.1, 109.9, 109.8, 87.9, 86.2, 55.4, 21.2, 21.0, 14.8, 14.3; HRMS (ESI) calcd. for $C_{18}H_{19}O_2$ ($[M+H]^+$): 267.1380; found: 267.1374.

NOESY (400 MHz, $CDCl_3$)

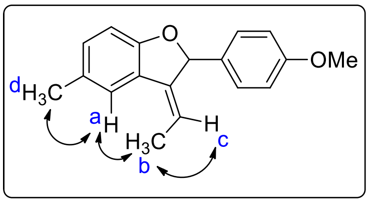

(*E*)-5-(*tert*-butyl)-3-ethylidene-2-(4-methoxyphenyl)-2,3-dihydrobenzofuran (5b) & (*E*)-5-(*tert*-butyl)-3-(4-methoxybenzylidene)-2-methyl-2,3-dihydrobenzofuran (5b')

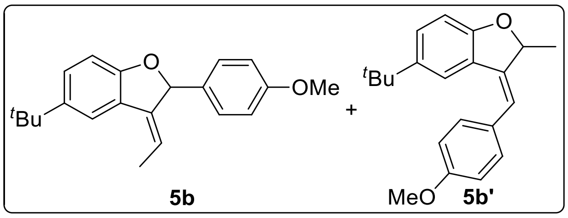

This compound was obtained in 64% yield (39.4 mg) as white solid. An inseparable mixture of two isomers was obtained; the ratio was determined to be 12/1 by ^{1}H-NMR analysis. Eluent: PE/EA = 10/1. R_f = 0.7; ^{1}H NMR (400 MHz, $CDCl_3$): δ 7.60 (s, 1H), 7.31-7.22 (m, 3H), 6.88 (d, *J* = 8.8 Hz, 2H), 6.81 (d, *J* = 8.4 Hz, 1H), 6.12-6.10 (m, 0.08H), 6.10-6.01 (m, 0.08H), 5.97-5.92 (m, 1H), 5.27 (qd, *J* = 7.3, 2.5 Hz, 1H), 3.79 (s, 3H), 3.78 (s, 0.25H), 2.01 (dd, *J* = 7.3, 2.5 Hz, 3H), 1.34 (s, 9H); ^{13}C NMR (100 MHz, $CDCl_3$): δ 160.6, 159.8, 143.6, 140.4, 133.4, 129.0, 126.9, 125.8, 121.3, 117.2, 114.1, 109.5, 88.1, 55.4, 34.5, 31.8, 14.4; HRMS (ESI) calcd. for $C_{21}H_{25}O_2$ ($[M+H]^+$): 309.1849; found: 309.1842.

(*E*)-3-ethylidene-2-(4-methoxyphenyl)-5-(trifluoromethyl)-2,3-dihydrobenzofuran (5c)

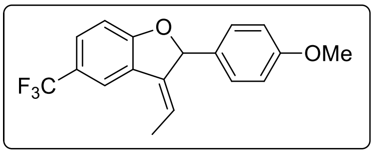

This compound was obtained in 50% yield (32.1 mg) as light yellow oil. Eluent: PE/EA = 10/1. R_f = 0.85; ^{1}H NMR (400 MHz, $CDCl_3$): δ 7.78 (s, 1H), 7.47 (dd, *J* = 8.5, 1.0 Hz, 1H), 7.28-7.22 (m, 2H), 6.93-6.87 (m, 3H), 6.06-6.03 (m, 1H), 5.42 (qd, *J* = 7.4, 2.6 Hz, 1H), 3.80 (s, 3H), 2.01 (dd, *J* = 7.4, 2.6 Hz, 3H); ^{13}C NMR (100 MHz, $CDCl_3$): δ 164.9, 160.2, 138.4, 132.5, 129.1, 127.3 (q, *J* = 3.7 Hz), 126.8, 124.7 (q, *J* = 270.4 Hz), 123.2 (q, *J* = 32.4 Hz), 121.7 (q, *J* = 3.7 Hz), 120.2, 114.2, 110.3, 88.9, 55.4, 14.4; ^{19}F NMR (376 MHz, $CDCl_3$): δ -61.10; HRMS (ESI) calcd. for $C_{18}H_{16}F_3O_2$($[M+H]^+$): 321.1097; found: 321.1089.

NOESY (400 MHz, $CDCl_3$)

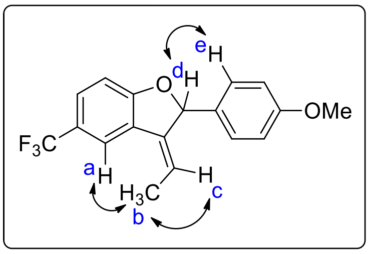

(*E*)-3-ethylidene-2-(4-methoxyphenyl)-5-nitro-2,3-dihydrobenzofuran (5d)

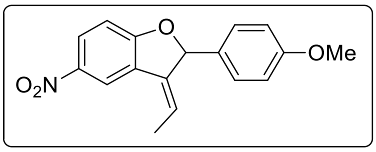

This compound was obtained in 48% yield (28.5 mg) as light yellow oil. Eluent: PE/EA = 10/1. R_f = 0.7; ^{1}H NMR (400 MHz, $CDCl_3$): δ 8.43 (d, *J* = 2.3 Hz, 1H), 8.19 (dd, *J* = 8.9, 2.4 Hz, 1H), 7.28-7.25 (m, 2H), 6.94-6.87 (m, 3H), 6.17-6.14 (m, 1H), 5.52 (qd, *J* = 7.4, 2.6 Hz, 1H), 3.82 (s, 3H), 2.08 (dd, *J* = 7.4, 2.6 Hz, 3H); ^{13}C NMR (100 MHz, $CDCl_3$): δ 167.4, 160.4, 142.1, 137.1, 131.7, 129.2, 127.2, 126.8, 122.1, 120.4, 114.3, 110.1, 90.2, 55.5, 14.6; HRMS (ESI) calcd. for $C_{17}H_{16}NO_4$ ($[M+H]^+$): 298.1074; found: 298.1067.

(*E*)-methyl 3-ethylidene-2-(4-methoxyphenyl)-2,3-dihydrobenzofuran-5-carboxylate (5e)

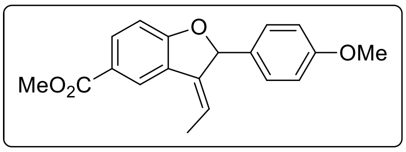

This compound was obtained in 80% yield (49.6 mg) as light yellow oil. Eluent: PE/EA = 10/1. R_f = 0.8; ^{1}H NMR (400 MHz, $CDCl_3$): δ 8.23 (s, 1H), 7.94 (d, *J* = 8.5 Hz, 1H), 7.27-7.20 (m, 2H), 6.89-6.83 (m, 3H), 6.03-6.01 (m, 1H), 5.37 (q, *J* = 7.2 Hz, 1H), 3.88 (s, 3H), 3.76 (s, 3H), 2.03 (d, *J* = 7.3 Hz, 3H); ^{13}C NMR (100 MHz, $CDCl_3$): δ 166.9, 166.2, 160.0, 138.2, 132.4, 132.2, 128.9, 126.4, 126.1, 122.8, 119.6, 114.0, 109.8, 89.0, 55.2, 51.9, 14.3; HRMS (ESI) calcd. for $C_{19}H_{19}O_4$ ($[M+H]^+$): 311.1278; found: 311.1273.

(*E*)-3-ethylidene-2-(4-methoxyphenyl)-6-methyl-2,3-dihydrobenzofuran (5f)

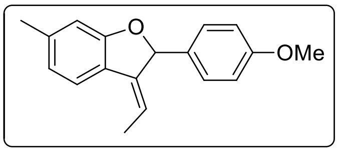

This compound was obtained in 72% yield (38.5 mg) as light yellow oil. Eluent: PE/EA = 20/1. R_f = 0.6; ^{1}H NMR (400 MHz, $CDCl_3$): δ 7.44 (d, *J* = 7.8 Hz, 1H), 7.28-7.23 (m, 2H), 6.88 (d, *J* = 8.6 Hz, 2H), 6.75 (d, *J* = 7.8 Hz, 1H), 6.70 (s, 1H), 5.97-5.93 (m, 1H), 5.23 (qd, *J* = 7.3, 2.5 Hz, 1H), 3.79 (s, 3H), 2.34 (s, 3H), 1.96 (dd, *J* = 7.3, 2.5 Hz, 3H); ^{13}C NMR (100 MHz, $CDCl_3$): δ 163.0, 159.9, 140.3, 139.9, 133.4, 128.9, 124.2, 123.6, 121.7, 116.5, 114.08, 110.9, 88.0, 55.4, 21.9, 14.3; HRMS (ESI) calcd. for $C_{18}H_{19}O_2$ ([M+H]$^+$): 267.1380; found: 267.1377.

(*E*)-3-ethylidene-2-(4-methoxyphenyl)-7-methyl-2,3-dihydrobenzofuran (5g)

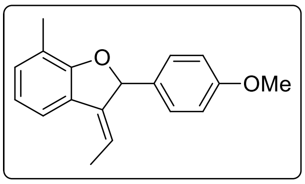

This compound was obtained in 61% yield (32.5 mg) as light yellow oil. Eluent: PE/EA = 20/1. R_f = 0.6; ^{1}H NMR (400 MHz, $CDCl_3$): δ 7.44 (d, *J* = 7.6 Hz, 1H), 7.31 (d, *J* = 8.6 Hz, 2H), 7.07 (d, *J* = 7.5 Hz, 1H), 6.94-6.85 (m, 3H), 6.01-5.96 (m, 1H), 5.31 (qd, *J* = 7.3, 2.5 Hz, 1H), 3.83 (s, 3H), 2.27 (s, 3H), 2.00 (dd, *J* = 7.3, 2.4 Hz, 3H); ^{13}C NMR (100 MHz, $CDCl_3$): δ 161.2, 159.8, 140.6, 133.6, 130.8, 128.9, 125.5, 122.0, 120.6, 120.3, 117.4, 114.1, 87.6, 55.4, 15.3, 14.2; HRMS (ESI) calcd. for $C_{18}H_{19}O_2$ ([M+H]$^+$): 267.1380; found: 267.1378.

(*E*)-7-bromo-3-ethylidene-2-(4-methoxyphenyl)-2,3-dihydrobenzofuran (5h)

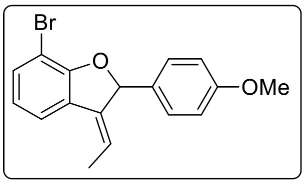

This compound was obtained in 37% yield (24.1 mg) as light yellow oil. Eluent: PE/EA = 20/1. R_f = 0.6; ^{1}H NMR (400 MHz, $CDCl_3$): δ 7.49 (d, *J* = 7.5 Hz, 1H), 7.36 (dd, *J* = 8.0, 0.9 Hz, 1H), 7.27 (dt, *J* = 4.5, 2.7 Hz, 2H), 6.91-6.87 (m, 2H), 6.83 (t, *J* = 7.8 Hz, 1H), 6.05 (t, *J* = 2.6 Hz, 1H), 5.35 (qd, *J* = 7.3, 2.6 Hz, 1H), 3.80 (s, 3H), 1.98

(dd, J = 7.4, 2.5 Hz, 3H); ^{13}C NMR (100 MHz, $CDCl_3$): δ 160.1, 159.7, 139.8, 132.6, 132.4, 129.1, 127.7, 123.4, 122.1, 119.7, 114.1, 103.4, 88.4, 55.4, 14.3; HRMS (ESI) calcd. for $C_{17}H_{16}BrO_2$ ($[M+H]^+$): 331.0328; found: 331.0325.

(*E*)-methyl 3-ethylidene-2-phenyl-2,3-dihydrobenzofuran-5-carboxylate (5i)

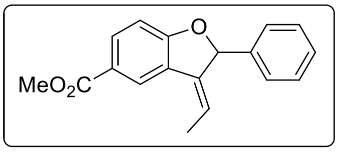

This compound was obtained in 60% yield (33.7 mg) as light yellow oil. Eluent: PE/EA = 10/1. R_f = 0.7; ^{1}H NMR (400 MHz, $CDCl_3$): δ 8.23 (s, 1H), 7.95 (dd, J = 8.4, 1.7 Hz, 1H), 7.39-7.28 (m, 5H), 6.88 (d, J = 8.5 Hz, 1H), 6.10-6.02 (m, 1H), 5.40 (qd, J = 7.3, 2.3 Hz, 1H), 3.88 (s, 3H), 2.02 (dd, J = 7.3, 2.3 Hz, 3H); ^{13}C NMR (100 MHz, $CDCl_3$): δ 167.0, 166.4, 140.3, 138.1, 132.3, 128.8, 127.9, 127.3, 126.4, 126.3, 123.0, 119.8, 109.9, 89.1, 52.1, 14.4; HRMS (ESI) calcd. for $C_{18}H_{17}O_3$ ($[M+H]^+$): 281.1172; found: 281.1169.

(*E*)-methyl 3-ethylidene-2-(4-fluorophenyl)-2,3-dihydrobenzofuran-5-carboxylate (5j)

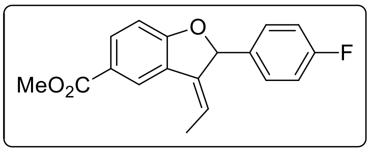

This compound was obtained in 80% yield (47.7 mg) as light yellow oil. Eluent: PE/EA = 10/1. R_f = 0.65; ^{1}H NMR (400 MHz, $CDCl_3$): δ 8.24 (d, J = 1.6 Hz, 1H), 7.97 (dd, J = 8.5, 1.8 Hz, 1H), 7.34-7.28 (m, 2H), 7.09-7.03 (m, 2H), 6.89 (d, J = 8.6 Hz, 1H), 6.09-6.05 (m, 1H), 5.40 (qd, J = 7.4, 2.6 Hz, 1H), 3.91 (s, 3H), 2.05 (dd, J = 7.4, 2.5 Hz, 3H); ^{13}C NMR (100 MHz, $CDCl_3$): δ 167.0, 166.2, 163.0 (d, J = 247.5 Hz), 138.1, 136.2 (d, J = 2.9 Hz), 132.4, 129.3 (d, J = 8.2 Hz), 126.32, 126.29, 123.2, 120.1, 115.7(d, J = 21.7 Hz), 110.0, 88.4, 52.1, 14.5; ^{19}F NMR (376 MHz, $CDCl_3$): δ -112.98; HRMS (ESI) calcd. for $C_{18}H_{16}FO_3$ ($[M+H]^+$): 299.1078; found: 299.1069.

NOESY (400 MHz, $CDCl_3$)

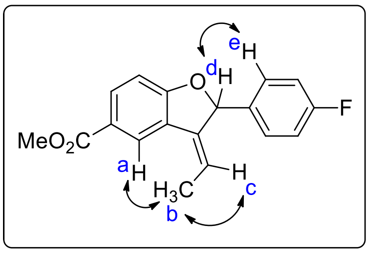

General procedure for the C-H cyclopropylation:

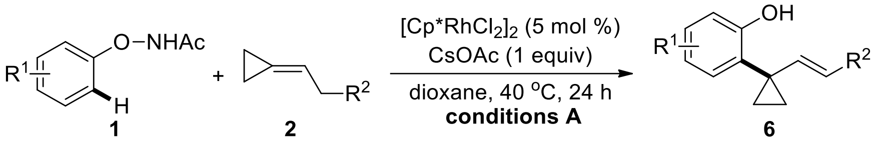

The mixture of *N*-phenoxyacetamides **1** (0.2 mmol, 1.0 equiv), methylenecyclopropanes **2** (0.4 mmol, 2.0 equiv), $[Cp^*RhCl_2]_2$ (5 mol %) and CsOAc (0.2 mmol, 1.0 equiv) in dioxane (1.0 mL) was stirred at 40 ^oC for 24 h without exclusion of air or moisture. Afterwards, the solvent was removed under reduced pressure, and theresulted mixture was purified by preparative TLC to afford the corresponding*ortho*-cyclopropyl phenols **6.**

Characterization of products 6:

(*E*)-2-(1-(3-phenylprop-1-en-1-yl)cyclopropyl)phenol (6a)

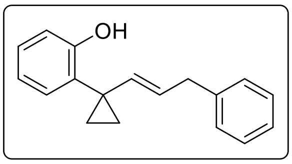

This compound was obtained in 73% yield (36.4 mg) as light yellow oil. Eluent: PE/EA = 10/1. R_f = 0.6; ^{1}H NMR (400 MHz, $CDCl_3$): δ 7.29-7.23 (m, 2H), 7.21-7.14 (m, 3H), 7.10 (d, J = 7.3 Hz, 2H), 6.90-6.85 (m, 2H), 5.47 (s, 1H), 5.28 (dt, J = 15.4, 6.5 Hz, 1H), 5.18 (d, J = 15.5 Hz, 1H), 3.30 (d, J = 6.4 Hz, 2H), 1.09-1.02 (m, 4H); ^{13}C NMR (100 MHz, $CDCl_3$): δ 155.5, 140.4, 135.9, 130.7, 128.8, 128.6, 128.5, 128.1, 127.5, 126.1, 120.5, 115.4, 38.6, 23.1, 14.6; HRMS (ESI) calcd. for $C_{18}H_{17}O$ ($[M-H]^-$): 249.1285; found: 249.1283.

(*E*)-4-methyl-2-(1-(3-phenylprop-1-en-1-yl)cyclopropyl)phenol (6b)

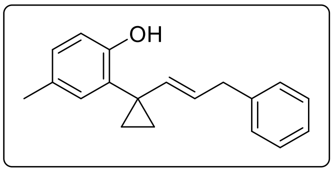

This compound was obtained in 57% yield (30.3 mg) as light yellow oil. Eluent: PE/EA = 10/1. R_f = 0.6; ^{1}H NMR (400 MHz, $CDCl_3$): δ 7.30-7.23 (m, 2H), 7.21-7.15 (m, 1H), 7.11 (d, *J* = 8.1 Hz, 2H), 7.01-6.95 (m, 2H), 6.82-6.77 (m, 1H), 5.36-5.25 (m, 2H), 5.16 (d, *J* = 15.4 Hz, 1H), 3.31 (d, *J* = 6.6 Hz, 2H), 2.26 (s, 3H), 1.09-1.00 (m, 4H); ^{13}C NMR (100 MHz, $CDCl_3$): δ 153.2, 140.4, 136.0, 131.1, 129.6, 129.2, 128.6, 128.5, 128.0, 127.2, 126.1, 115.2, 38.6, 23.1, 20.7, 14.5; HRMS (ESI) calcd. for $C_{19}H_{19}O$ ($[M-H]^-$): 265.1587; found: 265.1586.

(*E*)-4-(*tert*-butyl)-2-(1-(3-phenylprop-1-en-1-yl)cyclopropyl)phenol (6c)

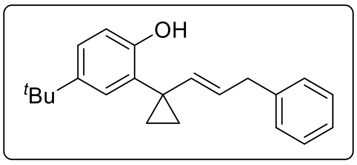

This compound was obtained in 61% yield (37.4 mg) as light yellow oil. Eluent: PE/EA = 10/1. R_f = 0.7; ^{1}H NMR (400 MHz, $CDCl_3$): δ 7.27-7.22 (m, 2H), 7.21-7.13 (m, 3H), 7.12-7.07 (m, 2H), 6.83-6.79 (m, 1H), 5.35-5.25 (m, 2H), 5.20 (d, *J* = 15.3 Hz, 1H), 3.30 (d, *J* = 6.4 Hz, 2H), 1.28 (s, 9H), 1.12-1.02 (m, 4H); ^{13}C NMR (100 MHz, $CDCl_3$): δ 153.1, 143.2, 140.5, 136.2, 128.6, 128.5, 127.9, 127.5, 126.7, 126.1, 125.5, 114.8, 38.5, 34.2, 31.7, 23.5, 14.5; HRMS (ESI) calcd. for $C_{22}H_{25}O$ ($[M-H]^-$): 305.1911; found: 305.1909.

(*E*)-4-methoxy-2-(1-(3-phenylprop-1-en-1-yl)cyclopropyl)phenol (6d)

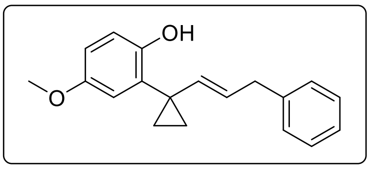

This compound was obtained in 44% yield (24.4 mg) as light yellow oil. Eluent: PE/EA = 10/1. R_f = 0.4; ^{1}H NMR (400 MHz, $CDCl_3$): δ 7.26 (t, *J* = 7.6 Hz, 2H), 7.16 (t, *J* = 7.0 Hz, 1H), 7.09 (d, *J* = 7.5 Hz, 2H), 6.83-6.78 (m, 1H), 6.75-6.70 (m, 2H), 5.35-5.25 (m, 1H), 5.19-5.13 (m, 2H), 3.73 (s, 3H), 3.30 (d, *J* = 6.6 Hz, 2H),

1.10-0.99 (m, 4H); ^{13}C NMR (100 MHz, $CDCl_3$): δ 153.4, 149.4, 140.4, 135.7, 128.6, 128.5, 128.3, 128.1, 126.1, 116.2, 115.9, 113.7, 55.8, 38.6, 23.5, 14.6; HRMS (ESI) calcd. for $C_{19}H_{19}O_2$ ($[M-H]^-$): 279.1390; found: 279.1385.

(*E*)-4-fluoro-2-(1-(3-phenylprop-1-en-1-yl)cyclopropyl)phenol (6e)

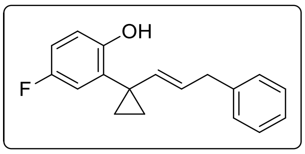

This compound was obtained in 66% yield (35.4 mg) as light yellow oil. Eluent: PE/EA = 10/1. R_f = 0.6; ^{1}H NMR (400 MHz, $CDCl_3$): δ 7.25 (t, *J* = 8.4 Hz, 2H), 7.17 (t, *J* = 7.6 Hz, 1H), 7.09 (d, *J* = 8.1 Hz, 2H), 6.90-6.77 (m, 3H), 5.33-5.25 (m, 2H), 5.15 (d, *J* = 15.4 Hz, 1H), 3.30 (d, *J* = 6.6 Hz, 2H), 1.06-1.03 (m, 4H); ^{13}C NMR (100 MHz, $CDCl_3$): δ 156.8 (d, *J* = 237.6 Hz), 151.5, 140.2, 135.2, 128.8, 128.7, 128.5, 128.4, 126.2, 117.0 (d, *J* = 22.9 Hz), 116.2 (d, *J* = 8.1 Hz), 115.1 (d, *J* = 23.0 Hz), 38.5, 23.3, 14.6; ^{19}F NMR (376 MHz, $CDCl_3$): δ -124.06; HRMS (ESI) calcd. for $C_{18}H_{16}FO$ ($[M-H]^-$): 267.1190; found: 267.1185.

(*E*)-4-chloro-2-(1-(3-phenylprop-1-en-1-yl)cyclopropyl)phenol (6f)

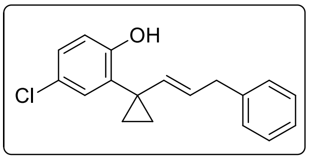

This compound was obtained in 63% yield (35.8 mg) as light yellow oil. Eluent: PE/EA = 10/1. R_f = 0.6; ^{1}H NMR (400 MHz, $CDCl_3$): δ 7.26 (t, *J* = 7.4 Hz, 2H), 7.17 (t, *J* = 7.3 Hz, 1H), 7.14-7.06 (m, 4H), 6.81 (d, *J* = 8.3 Hz, 1H), 5.44 (s, 1H), 5.29 (dt, *J* = 15.3, 6.6 Hz, 1H), 5.14 (d, *J* = 15.4 Hz, 1H), 3.30 (d, *J* = 6.6 Hz, 2H), 1.05-1.02 (m, 4H); ^{13}C NMR (100 MHz, $CDCl_3$): δ 154.1, 140.2, 135.1, 130.5, 129.2, 128.7, 128.6, 126.2, 125.1, 116.8, 38.6, 23.1, 14.6; HRMS (ESI) calcd. for $C_{18}H_{16}ClO$ ($[M-H]^-$): 283.0895; found: 283.0894.

(*E*)-4-bromo-2-(1-(3-phenylprop-1-en-1-yl)cyclopropyl)phenol (6g)

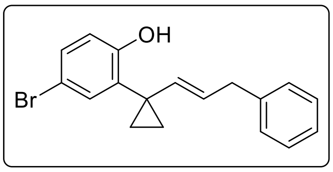

This compound was obtained in 64% yield (42.1 mg) as light yellow oil. Eluent: PE/EA = 10/1. R_f = 0.5; ^{1}H NMR (400 MHz, $CDCl_3$): δ 7.30-7.21 (m, 4H), 7.17 (t, *J* = 7.3 Hz, 1H), 7.09 (d, *J* = 7.1 Hz, 2H), 6.76 (d, *J* = 8.4 Hz, 1H), 5.46 (s, 1H), 5.29 (dt, *J* = 15.3, 6.6 Hz, 1H), 5.14 (d, *J* = 15.4 Hz, 1H), 3.30 (d, *J* = 6.6 Hz, 2H), 1.06-1.02 (m, 4H); ^{13}C NMR (100 MHz, $CDCl_3$): δ 154.7, 140.1, 135.1, 133.3, 131.6, 129.8, 128.6, 126.2, 117.3, 112.3, 38.5, 23.1, 14.6; HRMS (ESI) calcd. for $C_{18}H_{16}BrO$ ($[M-H]^-$): 327.0390; found: 327.0391.

(*E*)-4-iodo-2-(1-(3-phenylprop-1-en-1-yl)cyclopropyl)phenol (6h)

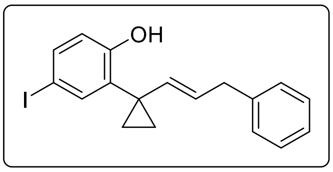

This compound was obtained in 50% yield (37.5 mg) as light yellow oil. Eluent: PE/EA = 10/1. R_f = 0.6; ^{1}H NMR (400 MHz, $CDCl_3$): δ 7.48-7.42 (m, 2H), 7.27 (m, 2H), 7.18 (t, *J* = 7.3 Hz, 1H), 7.10 (d, *J* = 7.1 Hz, 2H), 6.66 (d, *J* = 8.1 Hz, 1H), 5.47 (s, 1H), 5.29 (dt, *J* = 15.3, 6.6 Hz, 1H), 5.13 (d, *J* = 15.4 Hz, 1H), 3.30 (d, *J* = 6.6 Hz, 2H), 1.04-1.02 (m, 4H); ^{13}C NMR (100 MHz, $CDCl_3$): δ 155.5, 140.1, 139.2, 137.6, 135.1, 130.3, 128.6, 126.2, 117.9, 82.3, 38.5, 22.9, 14.6; HRMS (ESI) calcd. for $C_{18}H_{16}IO$ ($[M-H]^-$): 375.0251; found: 375.0251.

(*E*)-3-(1-(3-phenylprop-1-en-1-yl)cyclopropyl)-[1,1'-biphenyl]-4-ol (6i)

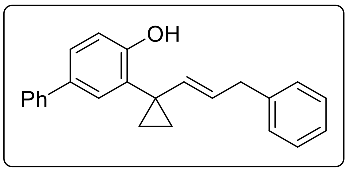

This compound was obtained in 59% yield (38.3 mg) as light yellow oil. Eluent: PE/EA = 10/1. R_f = 0.5; ^{1}H NMR (400 MHz, $CDCl_3$): δ 7.53 (d, *J* = 7.9 Hz, 2H), 7.46-7.34 (m, 4H), 7.30-7.23 (m, 3H), 7.16 (t, *J* = 6.9 Hz, 1H), 7.10 (d, *J* = 7.3 Hz, 2H), 6.96 (d, *J* = 8.7 Hz, 1H), 5.52 (s, 1H), 5.41-5.30 (m, 1H), 5.20 (d, *J* = 15.5 Hz,

1H), 3.31 (d, *J* = 6.3 Hz, 2H), 1.12-1.06 (m, 4H); ^{13}C NMR (100 MHz, $CDCl_3$): δ 155.1, 141.0, 140.4, 135.8, 133.7, 129.5, 128.8, 128.6, 128.5, 128.3, 127.8, 127.4, 126.8, 126.7, 126.2, 115.8, 38.6, 23.3, 14.6; HRMS (ESI) calcd. for $C_{24}H_{21}O$ ([M-H]$^-$): 325.1598; found: 325.1598.

(*E*)-2-(1-(3-phenylprop-1-en-1-yl)cyclopropyl)-4-(trifluoromethyl)phenol (6j)

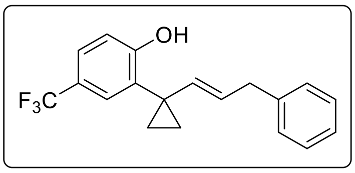

This compound was obtained in 44% yield (27.9 mg) as light yellow oil. Eluent: PE/EA = 10/1. R_f = 0.5; ^{1}H NMR (400 MHz, $CDCl_3$): δ 7.47-7.41 (m, 2H), 7.26 (t, *J* = 7.6 Hz, 2H), 7.17 (t, *J* = 7.3 Hz, 1H), 7.09 (d, *J* = 7.2 Hz, 2H), 6.95 (d, *J* = 9.1 Hz, 1H), 5.80 (brs, 1H), 5.27 (dd, *J* = 15.4, 6.4 Hz, 1H), 5.18 (d, *J* = 15.5 Hz, 1H), 3.31 (d, *J* = 6.4 Hz, 2H), 1.11-1.05 (m, 4H); ^{13}C NMR (100 MHz, $CDCl_3$): δ 158.2, 140.1, 135.0, 128.7, 128.58, 128.57, 128.02, 127.99, 126.3, 126.2 (q, *J* = 3.6 Hz), 124.5 (q, *J* = 269.7 Hz), 122.9 (q, *J* = 32.7 Hz), 115.8, 38.5, 23.0, 14.6; ^{19}F NMR (376 MHz, $CDCl_3$): δ -61.20; HRMS (ESI) calcd. for $C_{19}H_{16}F_3O$ ([M-H]$^-$): 317.1158; found: 317.1158.

(*E*)-4-hydroxy-3-(1-(3-phenylprop-1-en-1-yl)cyclopropyl)benzonitrile (6k)

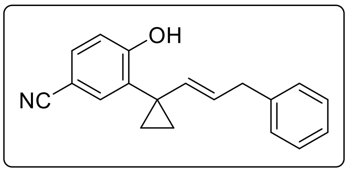

This compound was obtained in 73% yield (40.1 mg) as light yellow oil. Eluent: PE/EA = 3/1. R_f = 0.6; ^{1}H NMR (400 MHz, $CDCl_3$): δ 7.50-7.45 (m, 2H), 7.29-7.23 (m, 2H), 7.18 (t, *J* = 7.3 Hz, 1H), 7.08 (d, *J* = 7.4 Hz, 2H), 6.94 (d, *J* = 7.9 Hz, 1H), 6.01 (s, 1H), 5.23 (dt, *J* = 15.4, 6.2 Hz, 1H), 5.15 (d, *J* = 15.6 Hz, 1H), 3.31 (d, *J* = 6.2 Hz, 2H), 1.13-1.03 (m, 4H); ^{13}C NMR (100 MHz, $CDCl_3$): δ 159.3, 139.9, 135.2, 134.5, 133.2, 129.0, 128.6, 128.5, 126.3, 119.3, 116.6, 104.1, 38.5, 22.7, 14.5; HRMS (ESI) calcd. for $C_{19}H_{16}ON$ ([M-H]$^-$): 274.1237; found: 274.1237.

methyl (*E*)-4-hydroxy-3-(1-(3-phenylprop-1-en-1-yl)cyclopropyl)benzoate (6l)

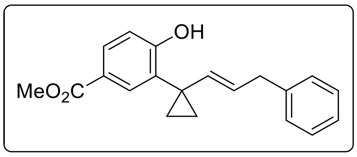

This compound was obtained in 72% yield (44.5 mg) as light yellow oil. Eluent: PE/EA = 3/1. R_f = 0.7; ^{1}H NMR (400 MHz, $CDCl_3$): δ 7.97-7.82 (m, 2H), 7.25 (t, *J* = 7.3 Hz, 2H), 7.16 (t, *J* = 7.2 Hz, 1H), 7.08 (d, *J* = 7.3 Hz, 2H), 6.91 (d, *J* = 8.3 Hz, 1H), 5.97 (s, 1H), 5.23-5.14 (m, 2H), 3.86 (s, 3H), 3.29 (d, *J* = 6.1 Hz, 2H), 1.09-1.04 (m, 4H); ^{13}C NMR (100 MHz, $CDCl_3$): δ 167.0, 159.6, 140.2, 135.2, 132.8, 130.9, 128.53, 128.47, 127.6, 126.2, 122.6, 115.4, 52.0, 38.5, 22.9, 14.6; HRMS (ESI) calcd. for $C_{20}H_{19}O_3$ ($[M-H]^-$): 307.1339; found: 307.1338.

(*E*)-5-methyl-2-(1-(3-phenylprop-1-en-1-yl)cyclopropyl)phenol (6m)

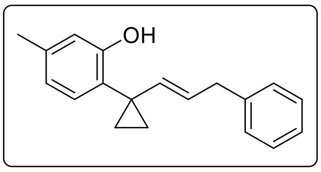

This compound was obtained in 56% yield (29.8 mg) as light yellow oil. Eluent: PE/EA = 10/1. R_f = 0.5; ^{1}H NMR (400 MHz, $CDCl_3$): δ 7.25 (t, *J* = 7.4 Hz, 2H), 7.16 (t, *J* = 7.2 Hz, 1H), 7.09 (d, *J* = 7.4 Hz, 2H), 7.03 (d, *J* = 7.7 Hz, 1H), 6.71 (s, 1H), 6.68 (d, *J* = 7.7 Hz, 1H), 5.41 (s, 1H), 5.32-5.24 (m, 1H), 5.16 (d, *J* = 15.4 Hz, 1H), 3.29 (d, *J* = 6.4 Hz, 2H), 2.28 (s, 3H), 1.06-0.97 (m, 4H); ^{13}C NMR (100 MHz, $CDCl_3$): δ 155.2, 140.5, 138.8, 136.1, 130.5, 128.6, 128.5, 128.0, 126.1, 124.5, 121.3, 116.0, 38.5, 22.7, 21.3, 14.5; HRMS (ESI) calcd. for $C_{19}H_{19}O$ ($[M-H]^-$): 263.1441; found: 263.1439.

(*E*)-5-fluoro-2-(1-(3-phenylprop-1-en-1-yl)cyclopropyl)phenol (6n)

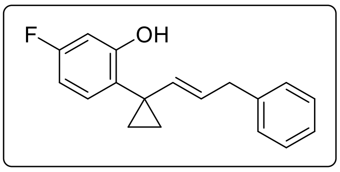

This compound was obtained in 67% yield (35.8 mg) as light yellow oil. Eluent: PE/EA = 10/1. R_f = 0.5; ^{1}H NMR (400 MHz, $CDCl_3$): δ 7.29-7.24 (m, 2H), 7.20-7.16 (m, 1H), 7.13-7.07 (m, 3H), 6.64-6.54 (m, 2H), 5.61 (d, *J* = 1.6 Hz, 1H), 5.25 (dt, *J* =

15.3, 6.5 Hz, 1H), 5.14 (t, *J* = 15.1 Hz, 1H), 3.30 (d, *J* = 6.5 Hz, 2H), 1.07-1.02 (m, 4H); ^{13}C NMR (100 MHz, $CDCl_3$): δ 163.0 (d, *J* = 243.1 Hz), 156.5 (d, *J* = 12.3 Hz), 140.3, 135.7, 131.6 (d, *J* = 9.8 Hz), 128.5, 128.3, 126.2, 123.3 (d, *J* = 3.0 Hz), 107.3 (d, *J* = 21.4 Hz), 103.0 (d, *J* = 24.9 Hz), 38.5, 22.5, 14.6; ^{19}F NMR (376 MHz, $CDCl_3$): δ -113.32; HRMS (ESI) calcd. for $C_{18}H_{16}FO$ ($[M-H]^-$): 267.1190; found: 267.1183.

(*E*)-5-chloro-2-(1-(3-phenylprop-1-en-1-yl)cyclopropyl)phenol (6o)

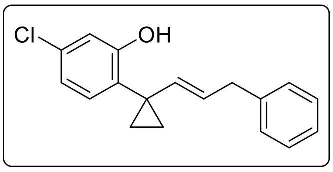

This compound was obtained in 57% yield (32.5 mg) as light yellow oil. Eluent: PE/EA = 10/1. R_f = 0.65; ^{1}H NMR (400 MHz, $CDCl_3$): δ 7.26 (t, *J* = 7.1 Hz, 2H), 7.19 (d, *J* = 7.3 Hz, 1H), 7.12-7.05 (m, *J* , 3H), 6.91 (s, 1H), 6.85 (d, *J* = 8.1 Hz, 1H), 5.56 (s, 1H), 5.30-5.22 (m, 1H), 5.15 (d, *J* = 15.5 Hz, 1H), 3.30 (d, *J* = 6.3 Hz, 2H), 1.06-1.02 (m, 4H); ^{13}C NMR (100 MHz, $CDCl_3$): δ 156.1, 140.2, 135.4, 133.9, 131.6, 128.54, 128.45, 126.2, 120.7, 115.9, 38.5, 22.6, 14.5; HRMS (ESI) calcd. for $C_{18}H_{16}ClO$ ($[M-H]^-$): 283.0895; found: 283.0895.

(*E*)-5-methoxy-2-(1-(3-phenylprop-1-en-1-yl)cyclopropyl)phenol (6p)

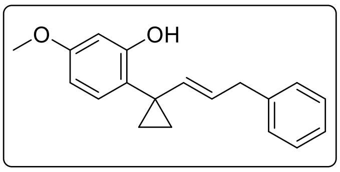

This compound was obtained in 57% yield (32.1 mg) as light yellow oil. Eluent: PE/EA = 10/1. R_f = 0.5; ^{1}H NMR (400 MHz, $CDCl_3$): δ 7.26 (t, *J* = 7.2 Hz, 2H), 7.17 (t, *J* = 7.2 Hz, 1H), 7.10 (d, *J* = 7.4 Hz, 2H), 7.05 (d, *J* = 8.3 Hz, 1H), 6.48 (s, 1H), 6.44 (d, *J* = 8.4 Hz, 1H), 5.51 (s, 1H), 5.34-5.24 (m, 1H), 5.16 (d, *J* = 15.4 Hz, 1H), 3.76 (s, 3H), 3.30 (d, *J* = 6.5 Hz, 2H), 1.05-1.01 (d, *J* = 9.6 Hz, 4H); ^{13}C NMR (100 MHz, $CDCl_3$): δ 160.3, 156.3, 140.5, 136.3, 131.3, 128.6, 128.5, 127.9, 126.1, 119.8, 106.4, 101.0, 55.4, 38.5, 22.4, 14.6; HRMS (ESI) calcd. for $C_{19}H_{19}O_2$ ($[M-H]^-$): 279.1390; found: 279.1390.

(*E*)-2-methyl-6-(1-(3-phenylprop-1-en-1-yl)cyclopropyl)phenol(6q)

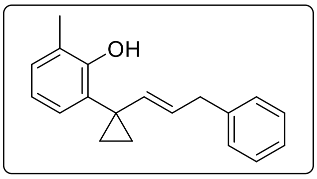

This compound was obtained in 67% yield (35.3 mg) as light yellow oil. Eluent: PE/EA = 10/1. R_f = 0.5; ^{1}H NMR (400 MHz, $CDCl_3$): δ 7.25 (t, *J* = 7.6 Hz, 2H), 7.16 (t, *J* = 7.3 Hz, 1H), 7.09 (d, *J* = 7.5 Hz, 2H), 7.04-6.98 (m, 2H), 6.76 (t, *J* = 7.5 Hz, 1H), 5.54 (s, 1H), 5.28 (dt, *J* = 15.4, 6.6 Hz, 1H), 5.17 (d, *J* = 15.3 Hz, 1H), 3.30 (d, *J* = 6.4 Hz, 2H), 2.24 (s, 3H), 1.08-1.00 (m, 4H); ^{13}C NMR (100 MHz, $CDCl_3$): δ 153.6, 140.4, 135.9, 130.0, 128.6, 128.5, 128.2, 128.0, 126.9, 126.1, 124.3, 120.0, 38.5, 23.2, 16.0, 14.7; HRMS (ESI) calcd. for $C_{19}H_{19}O$ ($[M-H]^-$): 263.1441; found: 263.1440.

(*E*)-3-(1-(3-phenylprop-1-en-1-yl)cyclopropyl)naphthalen-2-ol (6r)

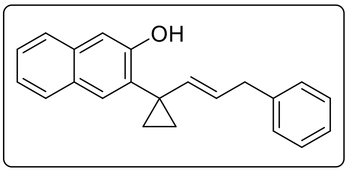

This compound was obtained in 45% yield (27.1 mg) as light yellow oil. Eluent: PE/EA = 10/1. R_f = 0.5; ^{1}H NMR (400 MHz, $CDCl_3$): δ 7.73-7.63 (m, 3H), 7.40-7.34 (m, 1H), 7.29 (dd, *J* = 6.9, 1.2 Hz, 1H), 7.26-7.21 (m, 3H), 7.14 (t, *J* = 7.3 Hz, 1H), 7.10-7.04 (m, 2H), 5.63 (s, 1H), 5.36-5.20 (m, 2H), 3.29 (d, *J* = 5.5 Hz, 2H), 1.20-1.08 (m, 4H); ^{13}C NMR (100 MHz, $CDCl_3$): δ 153.5, 140.2, 135.9, 134.3, 130.2, 129.9, 128.9, 128.8, 128.6, 128.5, 127.6, 126.4, 126.3, 126.2, 123.6, 110.1, 38.6, 23.6, 14.7; HRMS (ESI) calcd. for $C_{22}H_{19}O$ ($[M-H]^-$): 299.1441; found: 299.1440.

(*E*)-2-(1-(3-(4-bromophenyl)prop-1-en-1-yl)cyclopropyl)phenol (6s)

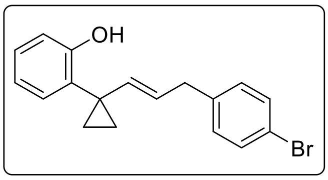

This compound was obtained in 67% yield (44.1 mg) as light yellow oil. Eluent: PE/EA = 10/1. R_f = 0.7; ^{1}H NMR (400 MHz, $CDCl_3$): δ 7.35 (d, *J* = 8.3 Hz, 2H), 7.20-7.13 (m, 2H), 6.95 (d, *J* = 8.3 Hz, 2H), 6.91-6.83 (m, 2H), 5.47 (s, 1H),

5.24-5.11 (m, 2H), 3.23 (d, J = 5.8 Hz, 2H), 1.09-1.00 (m, 4H); ^{13}C NMR (100 MHz, $CDCl_3$): δ 155.4, 139.4, 136.4, 131.5, 130.8, 130.3, 128.8, 127.4, 127.3, 120.6, 119.9, 115.4, 37.9, 23.1, 14.6; HRMS (ESI) calcd. for $C_{18}H_{16}BrO$ ([M-H]$^-$): 327.0390; found: 327.0393.

(*E*)-2-(1-(3-(5-methylfuran-2-yl)prop-1-en-1-yl)cyclopropyl)phenol (6t)

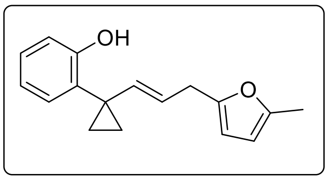

This compound was obtained in 69% yield (35.1 mg) as light yellow oil. Eluent: PE/EA = 10/1. R_f = 0.7; ^{1}H NMR (400 MHz, $CDCl_3$): δ 7.21-7.16 (m, 2H), 6.92-6.84 (m, 2H), 5.83-5.77 (m, 2H), 5.49 (s, 1H), 5.26-5.12 (m, 2H), 3.24 (d, J = 4.9 Hz, 2H), 2.22 (s, 3H), 1.09-1.02 (m, 4H); ^{13}C NMR (100 MHz, $CDCl_3$): δ 155.5, 152.3, 150.8, 136.6, 130.8, 128.8, 127.4, 125.0, 120.5, 115.5, 106.13, 106.05, 31.2, 23.1, 14.5, 13.6; HRMS (ESI) calcd. for $C_{17}H_{17}O_2$ ([M-H]$^-$): 253.1234; found: 253.1227.

3. Synthetic applications

Synthetic procedure of compound 7:

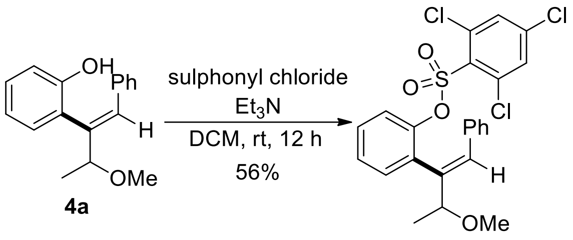

To a solution of **4a** (0.4 mmol, 1.0 equiv) and 2,4,6-trichlorobenzenesulfonyl chloride (0.8 mmol, 2.0 equiv) in DCM (8 mL) was added Et_3N (1.2 mmol, 3.0 equiv) at 0 °C, the mixture was allowed to warm to room temperature and stirred for 12 h. Afterwards, the solvent was removed under reduced pressure, and theresulted mixture was purified by column chromatography (Eluent: PE/DCM = 2/1, R_f = 0.3) to afford the desired product **7** in 56% (111.2 mg) isolated yield.

^{1}H NMR (400 MHz, $CDCl_3$): δ 7.40-7.33 (m, 2H), 7.30 (d, J = 8.2 Hz, 1H), 7.26-7.15 (m, 3H), 7.11-7.06 (m, 3H), 6.88 (s, 2H), 6.69 (s, 1H), 4.12 (q, J = 7.2 Hz, 1H), 3.45

(s, 3H), 1.28-1.15 (m, 3H); ^{13}C NMR (100 MHz, $CDCl_3$): δ 147.7, 139.6, 137.3, 136.8, 136.1, 132.6, 132.2, 131.2, 129.2, 129.0, 128.0, 127.5, 127.2, 121.5, 81.1, 56.7, 20.4; HRMS (ESI) calcd. for $C_{23}H_{19}Cl_3NaSO_4$ ($[M+Na]^+$): 518.9962; found: 518.9951.

Late-stage C-H modification of natural products:

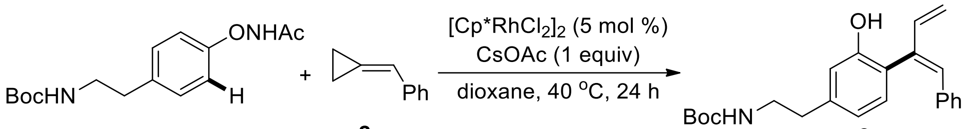

2a 8

The mixture of dopamine derivative (0.2 mmol, 1.0 equiv), methylenecyclopropane **2a** (0.4 mmol, 2.0 equiv), $[Cp^*RhCl_2]_2$ (5 mol %) and CsOAc (0.2 mmol, 1.0 equiv) in dioxane (1.0 mL) was stirred at 40 °C for 24 h without exclusion of air or moisture. Afterwards, the solvent was removed under reduced pressure, and theresulted mixture was purified by preparative TLC (Eluent: PE/EA = 2/1,R_f = 0.6) to afford the diene product **8** in 60% (43.8 mg) isolated yield as light yellow oil.

^{1}H NMR (400 MHz, DMSO-d_6): δ 8.98 (s, 1H), 7.15-7.06 (m, 3H), 7.02 (dd, J = 8.2, 2.2 Hz, 1H), 6.98 (d, J = 8.0 Hz, 2H), 6.81 (d, J = 8.2 Hz, 1H), 6.77 (t, J = 5.6 Hz, 1H), 6.74-6.65 (m, 3H), 5.06 (d, J = 11.4 Hz, 1H), 4.67 (d, J = 16.9 Hz, 1H), 3.07-2.95 (m, 2H), 2.55 (t, J = 7.5 Hz, 2H); ^{13}C NMR (100 MHz, $CDCl_3$): δ 156.0, 151.4, 140.0, 135.9, 135.8, 134.4, 131.5, 130.6, 129.9, 129.2, 128.4, 127.9, 123.5, 117.2, 116.2, 79.4, 42.1, 35.2, 28.5; HRMS (ESI) calcd. for $C_{23}H_{26}NO_3$ ($[M-H]^-$): 364.1918; found: 364.1918.

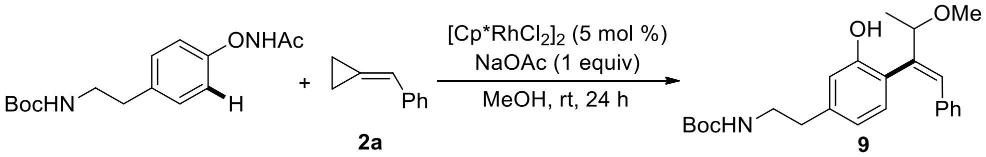

2a 9

The mixture of dopamine derivative (0.2 mmol, 1.0 equiv), methylenecyclopropane **2a** (0.4 mmol, 2.0 equiv), $[Cp^*RhCl_2]_2$ (5 mol %) and NaOAc (0.2 mmol, 1.0 equiv) in MeOH (1.0 mL) was stirred at room temperature for 24 h without exclusion of air or moisture. Afterwards, the solvent was removed under

reduced pressure, and theresulted mixture was purified by preparative TLC (Eluent: PE/EA = 2/1,R_f = 0.65) to afford the allyl methyl ether product **9** in 50% (39.7 mg) isolated yield as light yellow oil.

^{1}H NMR (400 MHz, $CDCl_3$): δ 8.18 (brs, 1H), 7.16-7.09 (m, 3H), 7.00 (dd, *J* = 8.2, 2.1 Hz, 1H), 6.96-6.92 (m, 2H), 6.90 (d, *J* = 8.2 Hz, 1H), 6.74 (s, 1H), 4.23 (brs, 1H), 4.12 (q, *J* = 6.5 Hz, 1H), 3.51 (s, 3H), 3.16-3.00 (m, 2H), 2.54 (t, *J* = 6.7 Hz, 2H), 1.42 (s, 9H), 1.25 (d, *J* = 6.3 Hz, 3H); ^{13}C NMR (100 MHz, $CDCl_3$): δ 155.9, 153.3, 138.1, 135.9, 133.4, 132.0, 130.7, 130.1, 129.6, 128.1, 127.6, 124.0, 117.3, 84.6, 79.2, 56.2, 41.9, 35.0, 28.5, 18.7; HRMS (ESI) calcd. for $C_{24}H_{30}NO_4$ ([M-H]$^-$): 396.2180; found: 396.2182.

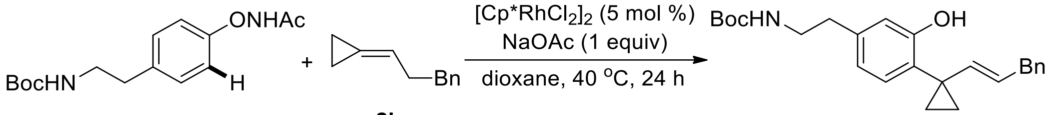

2j 10

The mixture of dopamine derivative (0.2 mmol, 1.0 equiv), methylenecyclopropane **2j** (0.4 mmol, 2.0 equiv), $[Cp^*RhCl_2]_2$ (5 mol %) and CsOAc (0.2 mmol, 1.0 equiv) in dioxane (1.0 mL) was stirred at 40 ^{o}C for 24 h without exclusion of air or moisture. Afterwards, the solvent was removed under reduced pressure, and the resulted mixture was purified by preparative TLC (Eluent: PE/EA = 2/1, R_f = 0.7) to afford the cyclopropane product **10** in 60% (47.1 mg) isolated yield as light yellow oil.

^{1}H NMR (400 MHz, $CDCl_3$): δ 7.28-7.23 (m, 2H), 7.19-7.13 (m, 1H), 7.12-7.07 (m, 2H), 7.01-6.95 (m, 2H), 6.81 (d, *J* = 8.7 Hz, 1H), 5.42 (s, 1H), 5.26 (dt, *J* = 15.4, 6.5 Hz, 1H), 5.16 (d, *J* = 15.4 Hz, 1H), 3.31-3.25 (m, 4H), 2.69 (t, *J* = 7.0 Hz, 2H), 1.43 (s, 9H), 1.10-0.98 (m, 4H); ^{13}C NMR (100 MHz, $CDCl_3$): δ 156.0, 154.0, 140.4, 135.9, 131.0, 130.8, 128.9, 128.6, 128.5, 128.1, 127.6, 126.1, 115.4, 79.3, 42.1, 38.5, 35.5, 28.6, 23.1, 14.5; HRMS (ESI) calcd. for $C_{25}H_{31}NO_3$ ([M-H]$^-$): 392.2231; found: 392.2230.

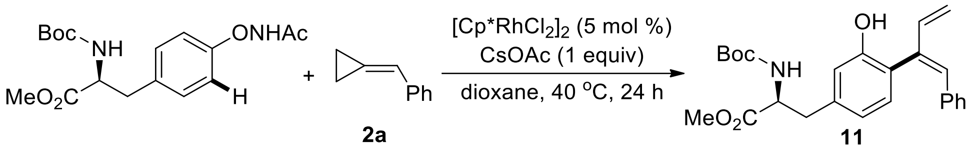

The mixture oftyrosine derivative (0.2 mmol, 1.0 equiv), methylenecyclopropane **2a** (0.4 mmol, 2.0 equiv), $[Cp^{*}RhCl_2]_2$ (5 mol %) and CsOAc (0.2 mmol, 1.0 equiv) in dioxane (1.0 mL) was stirred at 40 °C for 24 h without exclusion of air or moisture. Afterwards, the solvent was removed under reduced pressure, and the resulted mixture was purified by preparative TLC (Eluent: PE/EA = 3/1,R_f = 0.5) to afford the diene product **11** in 68% (57.5 mg) isolated yield as light yellow oil.

^{1}H NMR (400 MHz, DMSO-d_6): δ 9.01 (s, 1H), 7.14-7.07 (m, 3H), 7.04 (dd, J = 8.4, 2.0 Hz, 1H), 6.99 (d, J = 7.9 Hz, 2H), 6.80 (d, J = 8.2 Hz, 1H), 6.74 (s, 1H), 6.73-6.66 (m, 2H), 5.05 (d, J = 11.2 Hz, 1H), 4.65 (d, J = 17.0 Hz, 1H), 4.11-4.05 (m, 1H), 3.63-3.59 (m, 1H), 3.52 (s, 3H), 2.87-2.73 (m, 2H), 1.31 (s, 9H); ^{13}C NMR (100 MHz, $CDCl_3$): δ 172.4, 155.2, 151.8, 140.1, 135.7, 135.4, 134.5, 131.2, 130.5, 129.2, 128.5, 128.3, 128.1, 123.6, 117.3, 116.2, 80.1, 54.7, 52.3, 37.5, 28.4; HRMS (ESI) calcd. for $C_{25}H_{28}NO_5$ ($[M-H]^-$): 422.1973; found: 422.1971.

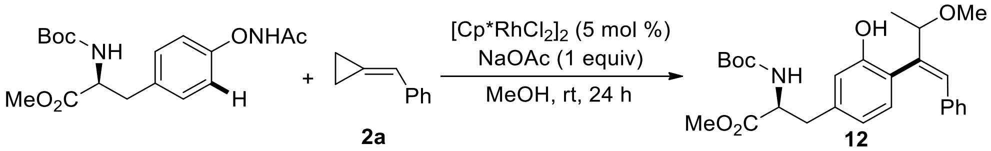

The mixture of tyrosine derivative (0.2 mmol, 1.0 equiv), methylenecyclopropane **2a** (0.4 mmol, 2.0 equiv), $[Cp^{*}RhCl_2]_2$ (5 mol %) and NaOAc (0.2 mmol, 1.0 equiv) in MeOH (1.0 mL) was stirred at room temperature for 24 h without exclusion of air or moisture. Afterwards, the solvent was removed under reduced pressure, and the resulted mixture was purified by preparative TLC (Eluent: PE/EA = 2/1,R_f = 0.5) to afford the allyl methyl ether product **12** in 55% (50.0 mg) isolated yield as light yellow oil. Two inseparable diastereomers were obtained, and the ratio was determined to be 1/1 by ^{1}H-NMR analysis.

^{1}H NMR (400 MHz, $CDCl_3$): δ 8.04 (brs, 1H), 7.21-7.09 (m, 3H), 6.99-6.93 (m, 3H), 6.90 (d, J = 8.3 Hz, 1H), 6.73 (s, 1H), 6.61-6.58 (m, 1H), 4.82-4.72 (m, 1H),

4.41-4.38 (m, 1H), 4.15-4.07 (m, 1H), 3.63 (s, 1.5H), 3.55 (s, 1.5H), 3.49 (s, 3H), 2.95-2.78 (m, 2H), 1.41 (s, 4.5H), 1.39 (s, 4.5H), 1.24-1.21 (m, 3H); ^{13}C NMR (100 MHz, $CDCl_3$): δ 172.5, 172.2, 155.1, 153.7, 153.6, 137.5, 137.4, 135.5, 133.4, 132.3, 132.2, 130.5, 130.3, 129.6, 128.4, 128.3, 127.82, 127.78, 124.3, 117.5, 84.8, 79.9, 79.8, 56.2, 54.7, 54.5, 53.8, 52.2, 37.23, 37.15, 29.4, 28.4, 18.6; HRMS (ESI) calcd. for $C_{26}H_{32}NO_6$ ([M-H]$^-$): 454.2235; found: 454.2240.

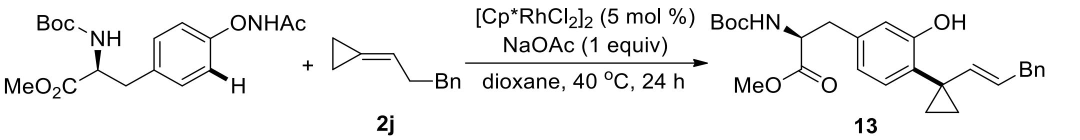

2j 13

The mixture oftyrosine derivative (0.2 mmol, 1.0 equiv), methylenecyclopropane **2j** (0.4 mmol, 2.0 equiv), $[Cp^*RhCl_2]_2$ (5 mol %) and CsOAc (0.2 mmol, 1.0 equiv) in dioxane (1.0 mL) was stirred at 40 °C for 24 h without exclusion of air or moisture. Afterwards, the solvent was removed under reduced pressure, and the resulted mixture was purified by preparative TLC (Eluent: PE/EA = 3/1,R_f = 0.6) to afford the cyclopropane product **13** in 49% (44.2 mg) isolated yield as light yellow oil.

^{1}H NMR (400 MHz, $CDCl_3$): δ 7.25 (t, J = 7.3 Hz, 2H), 7.16 (t, J = 7.4 Hz, 1H), 7.09 (d, J = 8.3 Hz, 2H), 6.93-6.89 (m, 2H), 6.80 (d, J = 8.1 Hz, 1H), 5.42 (s, 1H), 5.24 (dt, J = 15.3, 6.3 Hz, 1H), 5.15 (d, J = 15.4 Hz, 1H), 4.95 (d, J = 8.0 Hz, 1H), 4.55-4.49 (m, 1H), 3.60 (s, 3H), 3.29 (d, J = 6.3 Hz, 2H), 3.04-2.92 (m, 2H), 1.41 (s, 9H), 1.06-1.00 (m, 4H); ^{13}C NMR (100 MHz, $CDCl_3$): δ 172.5, 155.1, 154.5, 140.3, 135.8, 131.7, 129.5, 128.6, 128.5, 128.1, 127.7, 127.5, 126.1, 115.5, 80.0, 54.6, 52.2, 38.6, 37.7, 28.4, 23.1, 14.5; HRMS (ESI) calcd. for $C_{27}H_{32}NO_5$ ([M-H]$^-$): 450.2286; found: 450.2285.

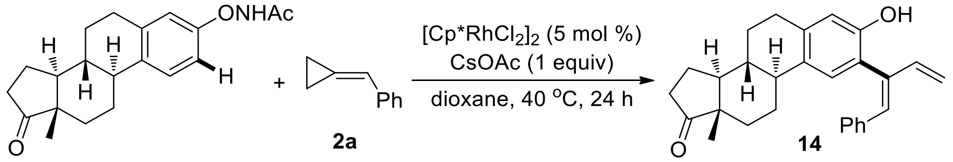

2a 14

The mixture ofestrone derivative (0.2 mmol, 1.0 equiv), methylenecyclopropane **2a** (0.4 mmol, 2.0 equiv), $[Cp^*RhCl_2]_2$ (5 mol %) and CsOAc (0.2 mmol, 1.0 equiv)

in dioxane (1.0 mL) was stirred at 40 °C for 24 h without exclusion of air or moisture. Afterwards, the solvent was removed under reduced pressure, and the resulted mixture was purified by preparative TLC (Eluent: PE/EA = 3/1,R_f = 0.6) to afford the diene product **14** in 47% (37.4 mg) isolated yield as light yellow oil.

^{1}H NMR (400 MHz, DMSO-*d*$_6$): δ 8.88 (s, 1H), 7.16-7.07 (m, 3H), 7.03 (d, *J* = 8.5 Hz, 2H), 6.73-6.64 (m, 3H), 6.60 (s, 1H), 5.05 (dd, *J* = 10.3, 1.2 Hz, 1H), 4.66 (dd, *J* = 17.0, 1.2 Hz, 1H), 2.88-2.76 (m, 2H), 2.43 (dd, *J* = 18.8, 8.4 Hz, 1H), 2.17-2.12 (m, 2H), 2.10-1.90 (m, 4H), 1.67 (d, *J* = 11.7 Hz, 1H), 1.60 -1.36 (m, 5H), 0.80 (s, 3H); ^{13}C NMR (100 MHz, $CDCl_3$): δ 221.4, 150.4, 140.6, 138.0, 136.0, 135.9, 134.2, 132.6, 129.2, 128.4, 127.9, 127.3, 120.7, 117.2, 115.7, 50.5, 48.2, 44.0, 38.4, 36.0, 31.6, 29.5, 26.6, 26.1, 21.7, 14.0; HRMS (ESI) calcd. for $C_{28}H_{29}O_2$ ([M-H]$^-$): 397.2173; found: 397.2169.

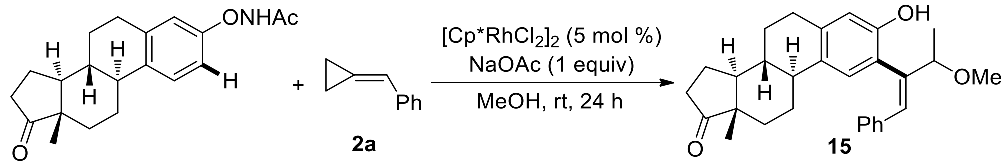

The mixture ofestrone derivative (0.2 mmol, 1.0 equiv), methylenecyclopropane **2a** (0.4 mmol, 2.0 equiv), $[Cp^*RhCl_2]_2$ (5 mol %) and NaOAc (0.2 mmol, 1.0 equiv) in MeOH (1.0 mL) was stirred at room temperature for 24 h without exclusion of air or moisture. Afterwards, the solvent was removed under reduced pressure, and the resulted mixture was purified by preparative TLC (Eluent: PE/EA = 2/1,R_f = 0.7) to afford the allyl methyl ether product **15** in 48% (41.2 mg) isolated yield as light yellow oil. Two inseparable diastereomers were obtained, and the ratio was determined to be 1/1 by ^{1}H-NMR analysis.

^{1}H NMR (400 MHz, $CDCl_3$): δ 8.10 (brs, 0.5H), 7.64 (brs, 0.5H), 7.16-7.08 (m, 3H), 7.01-6.97 (m, 2H), 6.75-6.66 (m, 3H), 4.15-4.05 (m, 1H), 3.50 (s, 1.5H), 3.49 (s, 1.5H), 2.91-2.84 (m, 2H), 2.16-2.00 (m, 5H), 1.98-1.80 (m, 1H), 1.74-1.53 (m, 3H), 1.49-1.36 (m, 3H), 1.27-1.24 (m, 3H), 0.88 (s, 1.5H), 0.80(s, 1.5H); ^{13}C NMR (100 MHz, $CDCl_3$): δ 221.4, 152.3, 152.1, 138.4, 138.10, 138.07, 137.96, 136.2, 135.9,

133.2, 132.6, 131.9, 131.7, 129.6, 129.5, 129.4, 128.3, 128.15, 128.0, 127.5, 127.3, 121.5, 120.8, 116.8, 84.7, 84.6, 56.22, 56.17, 50.44, 50.40, 48.11, 48.05, 43.74, 43.68, 38.4, 38.3, 36.0, 31.5, 31.4, 29.3, 29.2, 26.6, 26.5, 26.0, 25.5, 21.7, 19.0, 18.9, 14.0, 13.8; HRMS (ESI) calcd. for $C_{29}H_{33}O_3$ ($[M-H]^-$): 429.2435; found: 429.2434.

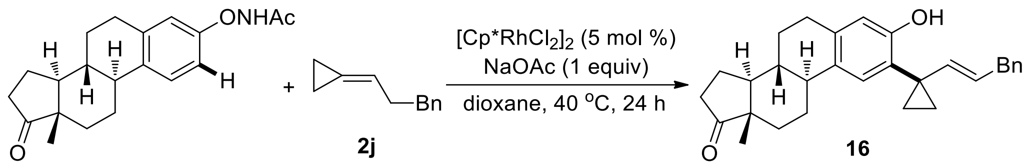

The mixture ofestrone derivative (0.2 mmol, 1.0 equiv), methylenecyclopropane **2j** (0.4 mmol, 2.0 equiv), $[Cp^*RhCl_2]_2$ (5 mol %) and CsOAc (0.2 mmol, 1.0 equiv) in dioxane (1.0 mL) was stirred at 40 °C for 24 h without exclusion of air or moisture. Afterwards, the solvent was removed under reduced pressure, and the resulted mixture was purified by preparative TLC (Eluent: PE/EA = 3/1,R_f = 0.6) to afford the cyclopropane product **16** in 56% (47.7 mg) isolated yield as light yellow oil.

^{1}H NMR (400 MHz, $CDCl_3$): δ 7.28-7.22 (m, 2H), 7.17 (t, J = 7.2 Hz, 1H), 7.12-7.05 (m, 3H), 6.62 (s, 1H), 5.33-5.25 (m, 2H), 5.17 (d, J = 15.4 Hz, 1H), 3.30 (d, J = 6.5 Hz, 2H), 2.89-2.80 (m, 2H), 2.49 (dd, J = 18.8, 8.5 Hz, 1H), 2.43-2.34 (m, 1H), 2.25-2.19 (m, 1H), 2.18-2.08 (m, 1H), 2.07-2.01 (m, 1H), 2.02-1.91 (m, 2H), 1.64-1.56 (m, 2H), 1.57-1.38 (m, 4H), 1.08-0.97 (m, 4H), 0.90 (s, 3H); ^{13}C NMR (100 MHz, $CDCl_3$): δ 221.3, 153.3, 140.5, 137.1, 136.2, 131.7, 128.6, 128.5, 127.9, 127.6, 126.1, 125.0, 115.2, 50.5, 48.2, 44.1, 38.5, 38.41, 36.0, 31.7, 29.4, 26.7, 26.1, 23.1, 21.7, 14.6, 14.0; HRMS (ESI) calcd. for $C_{30}H_{33}O_2$ ($[M-H]^-$): 425.2486; found: 425.2486.

Derivations of product 3a:

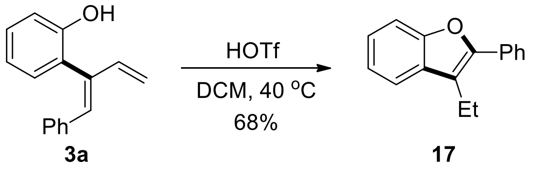

The mixture of **3a** (0.1 mmol, 1.0 equiv) and two drop of HOTf in DCM (1 mL) was stirred at 40 °C for 4 h without exclusion of air or moisture. Then, the reaction

mixture was concentrated and purified by preparative TLC (eluent: PE/acetone = 100/1, R_f = 0.9) to give thebenzofuran derivative **17** in 68% isolated yield (15.1 mg) as light yellow oil.

^{1}H NMR (400 MHz, $CDCl_3$): δ 7.80-7.76 (m, 2H), 7.61-7.57 (m, 1H), 7.51-7.45 (m, 3H), 7.40-7.34 (m, 1H), 7.32-7.22 (m, 2H), 2.95 (q, J = 7.6 Hz, 2H), 1.38 (t, J = 7.6 Hz, 3H); ^{13}C NMR (100 MHz, $CDCl_3$): δ 154.1, 150.4, 131.5, 130.4, 128.8, 128.2, 126.9, 124.4, 122.4, 119.6, 117.8, 111.2, 17.7, 14.4; HRMS (ESI) calcd. for $C_{16}H_{15}O$ ($[M+H]^+$): 223.1118; found: 223.1116.

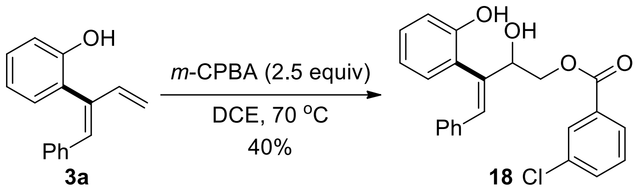

The mixture of **3a** (0.1 mmol, 1.0 equiv) and *m*-CPBA(0.25 mmol, 2.5 equiv) in DCE (0.5 mL) was stirred at 70 °C for 24 h without exclusion of air or moisture. Then, the reaction mixture was concentrated and purified by preparative TLC (eluent: PE/EA = 3/1,R_f = 0.7) to give theoxidative product **18** in 40% isolated yield (15.7 mg) as light yellow crystal.

^{1}H NMR (400 MHz, CD_3OD): δ 8.05 (s, 1H), 8.00 (d, J = 6.4 Hz, 1H), 7.62 (d, J = 7.9 Hz, 1H), 7.48 (t, J = 7.9 Hz, 1H), 7.23-7.18 (m, 1H), 7.09-6.96 (m, 6H), 6.91-6.79 (m, 3H), 5.83-5.75 (m, 1H), 3.95-3.79 (m, 2H); ^{13}C NMR (100 MHz, DMSO-d_6): δ166.1, 164.2, 154.9, 136.3, 133.5, 133.4, 133.1, 133.0, 132.7, 132.3, 130.7, 128.91, 128.87, 128.6, 127.99, 127.95, 127.1, 119.4, 115.7, 80.3, 62.2; HRMS (ESI) calcd. for $C_{23}H_{19}ClNaO_4$ ($[M+Na]^+$): 417.0864; found: 417.0854.

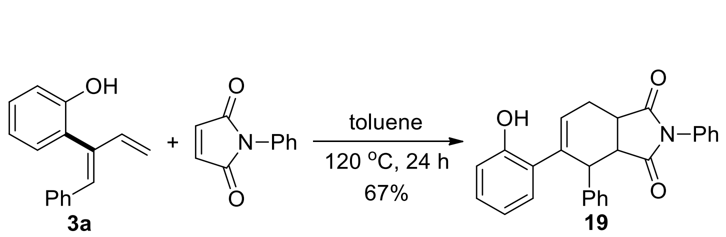

The mixture of **3a** (0.2 mmol, 1.0 equiv) and *N*-phenylmaleimide (0.24 mmol, 1.2 equiv) in toluene (2 mL) was stirred at 120 °C for 24 h under an atmosphere of

N_2.Then, the reaction mixture was concentrated and purified by preparative TLC (eluent: PE/EA = 3/1,R_f = 0.2) to give the oxidative product **19** in 67% isolated yield (52.8 mg) as light yellow solid.

^{1}H NMR (400 MHz, CD_3OD): δ 7.27-7.23 (m, 3H), 7.23-7.19 (m, 3H), 7.17-7.13 (m, 2H), 7.01 (t, J = 7.7 Hz, 1H), 6.83 (dd, J = 7.5, 1.6 Hz, 1H), 6.75 (d, J = 8.0 Hz, 1H), 6.62 (t, J = 7.5 Hz, 1H), 6.44-6.40 (m, 2H), 6.12 (dd, J = 5.8, 2.5 Hz, 1H), 4.61 (d, J = 7.1 Hz, 1H), 3.70 (dd, J = 8.9, 7.1 Hz, 1H), 3.64-3.54 (m, 1H), 3.09-3.00 (m, 1H), 2.93-2.82 (m, 1H); ^{13}C NMR (100 MHz, CD_3OD): δ 181.4, 179.2, 155.7, 140.8, 139.1, 133.2, 131.3, 130.8, 130.5, 129.61, 129.56, 129.48, 129.40, 128.5, 127.9, 126.4, 120.4, 116.3, 46.9, 45.7, 38.0, 22.8; HRMS (ESI) calcd. for $C_{26}H_{20}NO_3$ ([M-H]$^-$): 394.1448; found: 394.1461.

Derivation of product 5a:

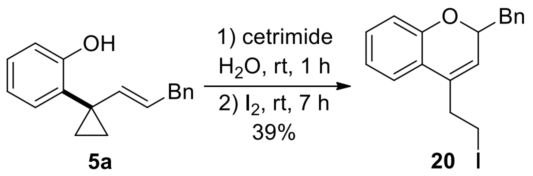

A reaction mixture containing **5a** (0.2 mmol, 1 equiv), surfactant cetrimide (0.12 mmol, 0.6 equiv) and water (2 mL) was stirred at room temperature for 1 h. Molecular iodine (0.28 mmol, 1.4 equiv) was added, and the reaction mixture was stirred at this temperature for an additional period of 7 h. After the reaction was over, the reaction mixture was extracted with EtOAc, washed with saturation $NaHCO_3$ and $Na_2S_2O_3$ solution, and then by brine, dried over anhydrous Na_2SO_4. The crude mass was purified by preparative TLC using PE/EA = 10/1 (R_f = 0.85) as eluent to give the pure product in 39% (29.3 mg) yieldas light yellow oil.

^{1}H NMR (400 MHz, $CDCl_3$): δ 7.32 (t, J = 7.1 Hz, 2H), 7.26-7.21 (m, 3H), 7.19-7.14 (m, 1H), 7.11 (dd, J = 7.7, 1.5 Hz, 1H), 6.92 (td, J = 7.5, 1.1 Hz, 1H), 6.85 (dd, J = 8.0, 1.1 Hz, 1H), 5.57 (d, J = 3.8 Hz, 1H), 4.98 (ddd, J = 7.3, 6.5, 3.8 Hz, 1H), 3.28 (t, J = 7.4 Hz, 2H), 3.12 (dd, J = 13.6, 7.5 Hz, 1H), 2.96 (t, J = 7.9 Hz, 2H), 2.91 (dd, J = 13.7, 6.4 Hz, 1H); ^{13}C NMR (100 MHz, $CDCl_3$): δ 153.5, 137.2, 132.8, 129.7, 129.6,

128.5, 126.7, 123.0, 122.8, 121.9, 121.3, 117.0, 75.6, 41.4, 36.2, 2.9; HRMS (ESI) calcd. for $C_{18}H_{18}IO$ ([M+H]$^+$): 377.0397; found: 377.0388.

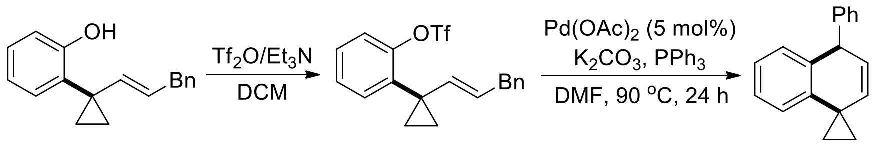

5a **21** **22**

The **5a** (0.4 mmol, 1.0 equiv) was dissolved in DCM (4 mL) with Et_3N (0.48 mmol, 1.2 equiv), the resulted mixture was stirred at 0 °C, then Tf_2O (0.48mmol, 1.2 equiv) was added slowly. The reaction was allowed warm to room temperature and stirred for 30 minutes, afterwards, the reaction mixture was concentrated and purified by preparative TLC to give the desired triflate derivative **21**.

The mixture of triflate derivative **21** (0.1 mmol, 1.0 equiv), $Pd(OAc)_2$ (5 mol %), K_2CO_3 (0.1 mmol, 1.0 equiv), triphenylphosphine (20 mol %) in DMF (0.5 mL) was stirred at 90 °C for 24 h without exclusion of air or moisture. Then, the reaction mixture was concentrated and purified by preparative TLC to give the product **22**.

2-(1-(3-phenylprop-1-en-1-yl)cyclopropyl)phenyl trifluoromethanesulfonate (21)

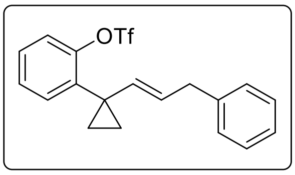

This compound was obtained in 52% yield (79.5 mg) as light yellow oil. Eluent: PE/EA = 10/1. R_f = 0.8; ^{1}H NMR (400 MHz, $CDCl_3$): δ 7.41-7.39 (m, 1H), 7.30-7.28(m, 2H), 7.27-7.20 (m, 3H), 7.16 (d, *J* = 6.5 Hz, 1H), 7.11 (d, *J* = 7.6 Hz, 2H), 5.28 (d, *J* = 15.3 Hz, 1H), 5.14-5.04 (m, 1H), 3.28 (d, *J* = 6.6 Hz, 2H), 1.14-1.11 (m, 2H), 1.09-1.01 (m, 2H); ^{13}C NMR (100 MHz, $CDCl_3$): δ 149.9, 140.6, 136.7, 136.2, 133.2, 128.8, 128.6, 128.4, 128.3, 127.5, 126.0, 121.2, 118.5 (q, *J* = 317.8 Hz), 38.6, 24.5, 14.8; ^{19}F NMR (376 MHz, $CDCl_3$): δ-75.54; HRMS (ESI) calcd. for $C_{19}H_{18}F_3O_3S$ ([M+H]$^+$): 383.0923; found: 383.0921.

4'-phenyl-4'H-spiro[cyclopropane-1,1'-naphthalene] (22)

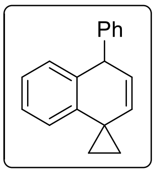

This compound was obtained in 60% yield (13.9 mg) as light yellow oil. Eluent: PE/EA = 3/1. R_f = 0.9; ^{1}H NMR (400 MHz, $CDCl_3$): δ 7.44-7.38 (m, 2H), 7.36-7.30 (m, 2H), 7.30-7.26 (m, 1H), 7.10 (dd, *J* = 7.7, 1.3 Hz, 1H), 6.89-6.81 (m, 2H), 6.69 (dd, *J* = 7.4, 1.2 Hz, 1H), 6.57 (d, *J* = 15.8 Hz, 1H), 6.23 (dd, *J* = 15.9, 8.7 Hz, 1H), 5.18 (d, *J* = 8.7 Hz, 1H), 1.19-1.12 (m, 2H), 0.93-0.85 (m, 2H); ^{13}C NMR (100 MHz, $CDCl_3$): δ 159.6, 136.1, 134.2, 132.5, 128.8, 128.3, 127.5, 126.9, 125.8, 120.8, 119.1, 109.2, 88.8, 28.5, 14.3, 14.2; HRMS (ESI) calcd. for $C_{18}H_{16}$ (M^+): 232.1252; found: 232.1249.

4. Mechanistic Studies

Deuteration experiments:

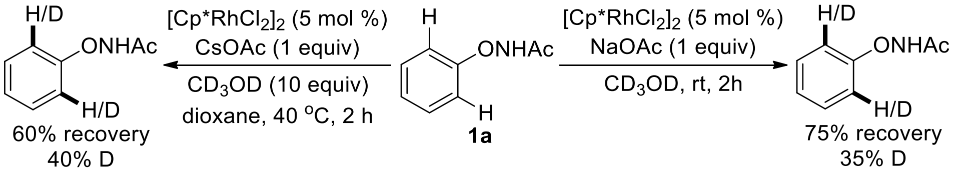

Conditions A: **1a** (0.2 mmol, 1.0 equiv) was dissolved in dioxane (1.0 mL) in thepresence of $[Cp^*RhCl_2]_2$ (5 mol %) andCsOAc (0.2 mmol, 1.0 equiv). CD_3OD (10 equiv) was added as the source of deuterium. The reaction was conducted under the standard conditions for 2 h, afterwards, **1a** was recovered by flash column chromatography on silica gel (eluent: Petroleum ether/Ethyl acetate = 3/1). 40% deuteration was detected by ^{1}H-NMR analysis.

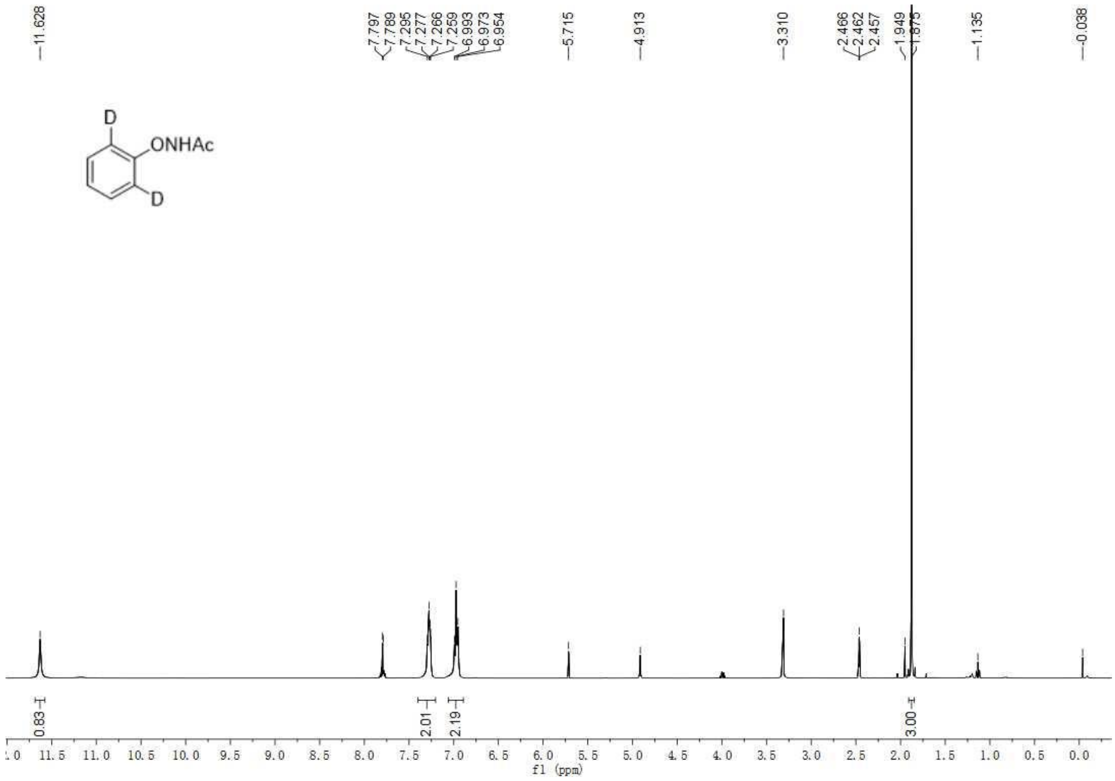

Conditions B: **1a** (0.2 mmol, 1.0 equiv) was dissolved in CD_3OD (1.0 mL) in thepresence of $[Cp^*RhCl_2]_2$ (5 mol %) and NaOAc (0.2 mmol, 1.0 equiv). The reaction was conducted under the standard conditions for 2 h, afterwards, **1a** was recovered by flash column chromatography on silica gel (eluent: Petroleum ether/Ethyl acetate = 3/1). 35% deuteration was detected by ^{1}H-NMR analysis.

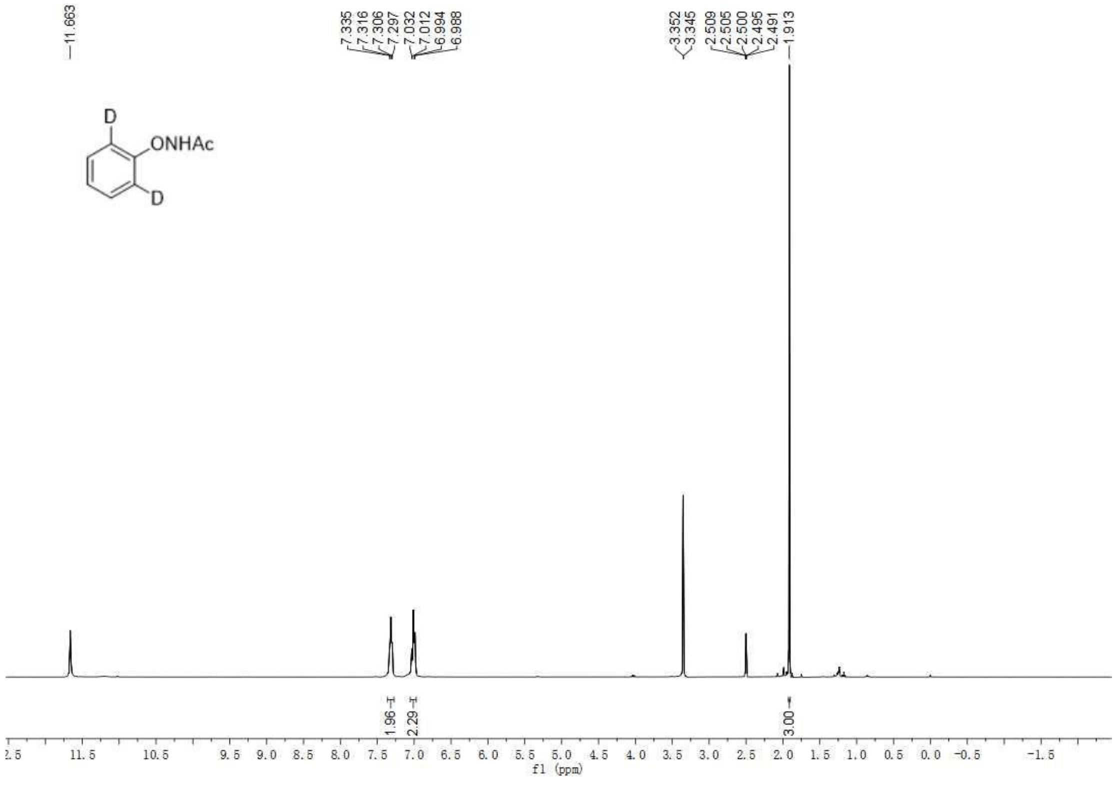

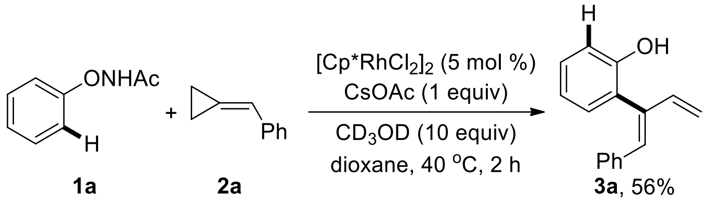

The mixture of *N*-phenoxyacetamide **1a** (0.2 mmol, 1.0 equiv), methylenecyclopropanes **2a** (0.4 mmol, 2.0 equiv), [Cp*RhCl$_2$]$_2$ (5 mol %) and CsOAc (0.2 mmol, 1.0 equiv) in dioxane (1.0 mL) was stirred under the standard conditions for 2 h. CD$_3$OD (10 equiv) was added as the source of deuterium. Afterwards, the solvent was removed under reduced pressure, and theresulted mixture was purified by preparative TLC (eluent: PE/EA = 10/1) to afford the desired product **3a** in 56% yield. The deuterium incorporation was analyzed by ^{1}H-NMR spectroscopy and no deuterium incorporation was observed at either the *ortho* position of the directing group or the methylenecyclopropanes.

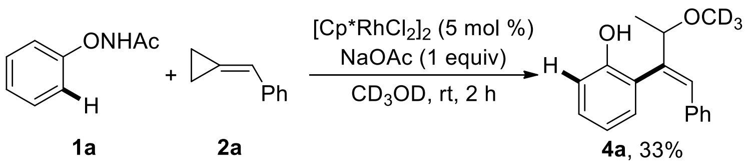

The mixture of *N*-phenoxyacetamide **1a** (0.2 mmol, 1.0 equiv),

methylenecyclopropanes **2a** (0.4 mmol, 2.0 equiv), [Cp*$RhCl_2$]$_2$ (5 mol %) and NaOAc (0.2 mmol, 1.0 equiv) in CD_3OD (1.0 mL) was stirred under the standard conditions for 2 h. Afterwards, the solvent was removed under reduced pressure, and the resulted mixture was purified by preparative TLC (eluent: PE/EA = 10/1) to afford the desired product **4a** in 33% yield. The deuterium incorporation was analyzed by ^{1}H-NMR spectroscopy and no deuterium incorporation was observed at either the *ortho* position of the directing group or the methylenecyclopropanes, while OCD_3 was detected instead of OMe.

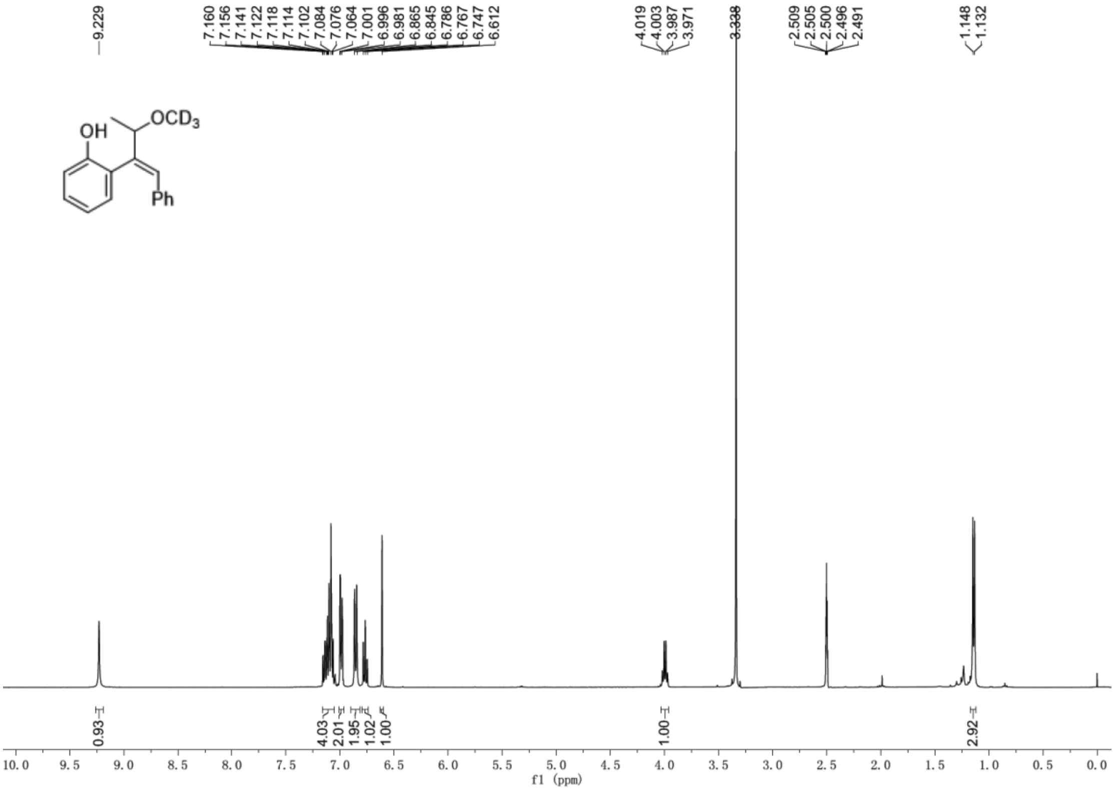

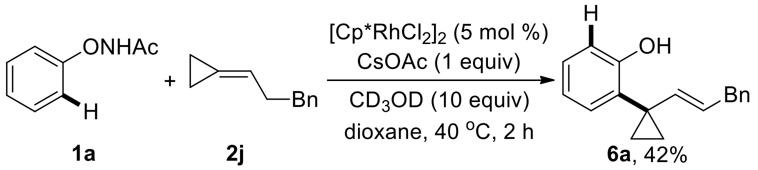

The mixture of *N*-phenoxyacetamide **1a** (0.2 mmol, 1.0 equiv), methylenecyclopropanes **2j** (0.4 mmol, 2.0 equiv), [Cp*$RhCl_2$]$_2$ (5 mol %) and CsOAc (0.2 mmol, 1.0 equiv) in dioxane (1.0 mL) was stirred under the standard conditions for 2 h. CD_3OD (10 equiv) was added as the source of deuterium. Afterwards, the solvent was removed under reduced pressure, and the resulted mixture was purified by preparative TLC (eluent: PE/EA = 10/1) to afford the desired

product **6a** in 42% yield. The deuterium incorporation was analyzed by ^{1}H-NMR spectroscopy and no deuterium incorporation was observed at either the *ortho* position of the directing group or the methylenecyclopropanes.

General procedure for estimation of the KIE:

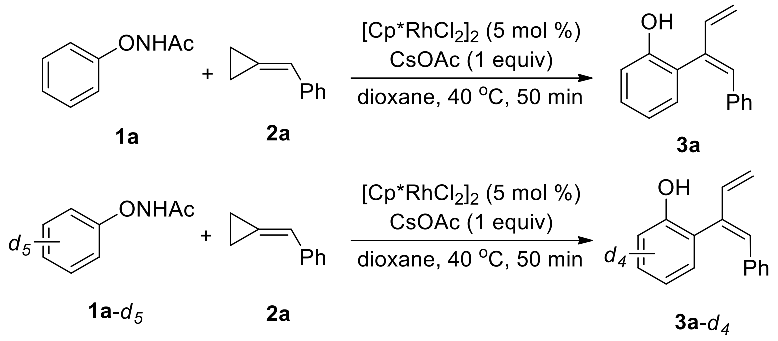

Without any particular precautions to exclude oxygen or moisture, **1a** (30.2 mg, 0.2 mmol, 1.0 equiv) or **1a-*d*5**(31.2 mg, 0.2 mmol, 1.0 equiv), $[Cp^*RhCl_2]_2$ (6.4 mg, 5 mol %), CsOAc (38.4 mg, 0.2 mmol, 1.0 equiv) and **2a** (0.4 mmol, 2.0 equiv) were added to a 5 mL vial equipped with a stir bar at 40 ^{o}C. Dioxane (1 mL) was added and timing was started. An aliquot of 0.1 mL was taken every 10 minutes for 50 minutes and immediately diluted with EtOAc (1 mL), filtered through a short silica column, washed with 25 mL of EtOAc. The solvent was then removed under reduced pressure and analyzed by ^{1}H-NMR with 1,3,5-trimethoxybenzene as the internal standard. The double doublet at δ: 5.07 and 4.64 was used for the calculation of ^{1}H-NMR yields, which were shown below:

entry	time/min	Yield of**3a** (%)	Yield of **3a-*d*4** (%)
1	10	9	3
2	20	12	8
3	30	16	10
4	40	19	14
5	50	23	16

KIE value was obtained by comparing the initial rates measured separately, the slopes of the reaction rate curves were calculated, thus resulted in the KIE = 0.35/0.32=1.09.

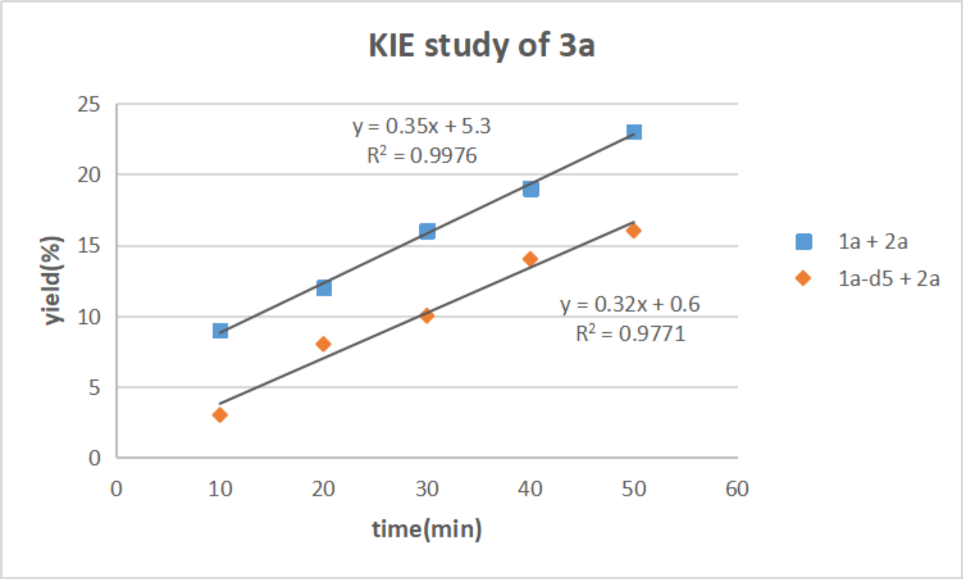

Crude ^{1}H-NMR of the synthesis for **3a** under standard conditions for 50 min:

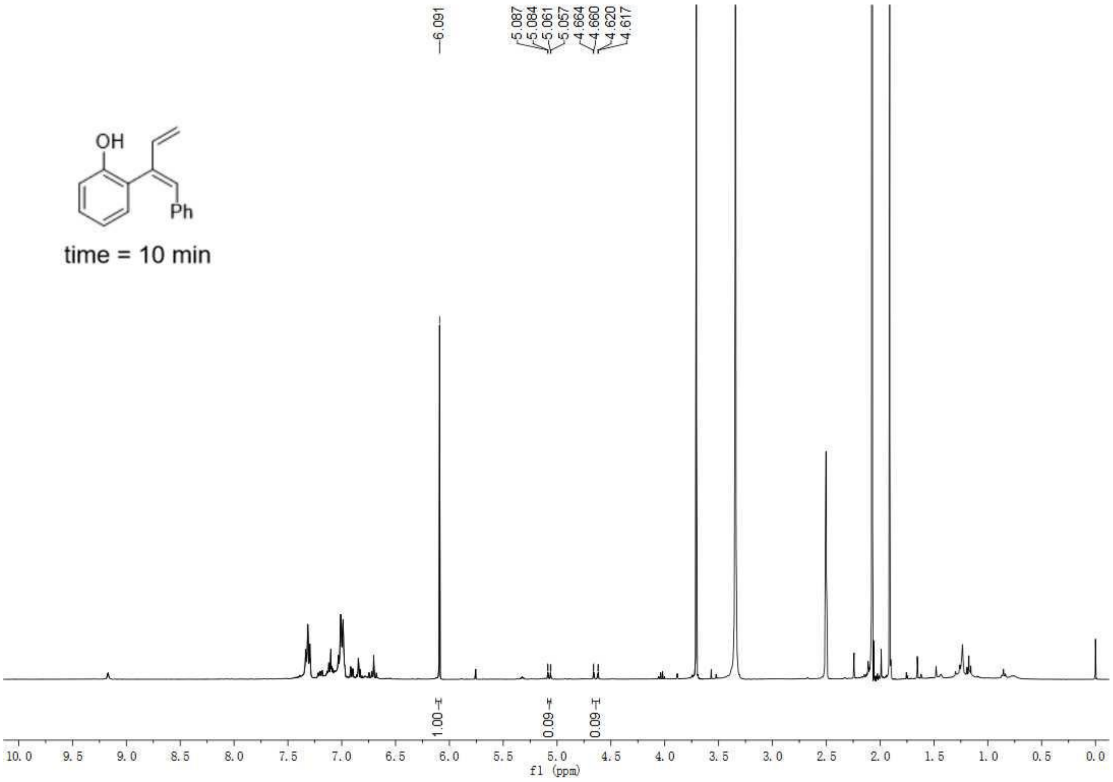

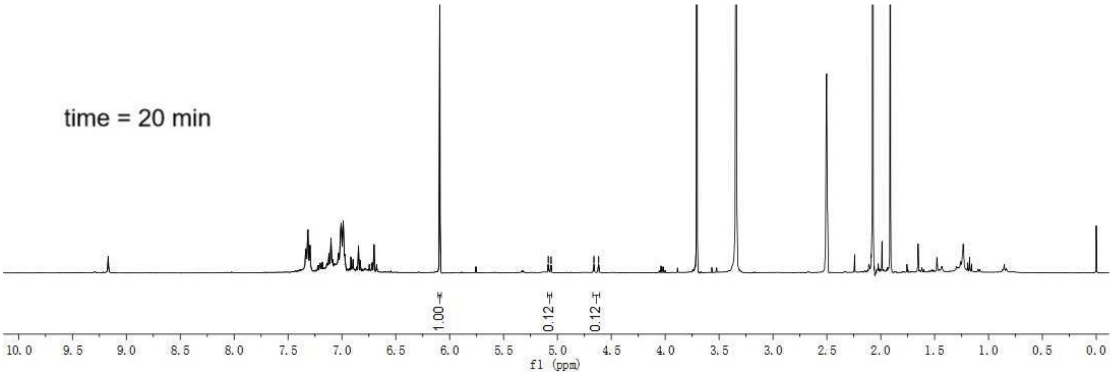

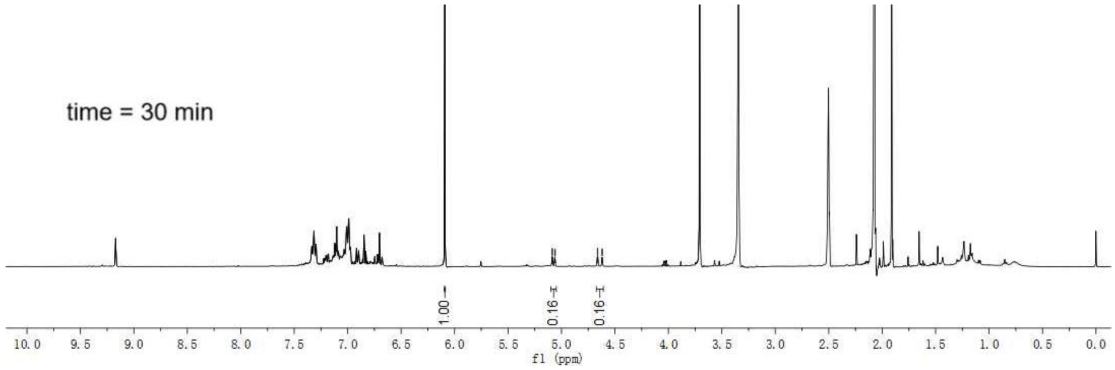
time = 30 min
1.00
0.16
0.16
10.0 9.5 9.0 8.5 8.0 7.5 7.0 6.5 6.0 5.5 5.0 4.5 4.0 3.5 3.0 2.5 2.0 1.5 1.0 0.5 0.0
f1 (ppm)

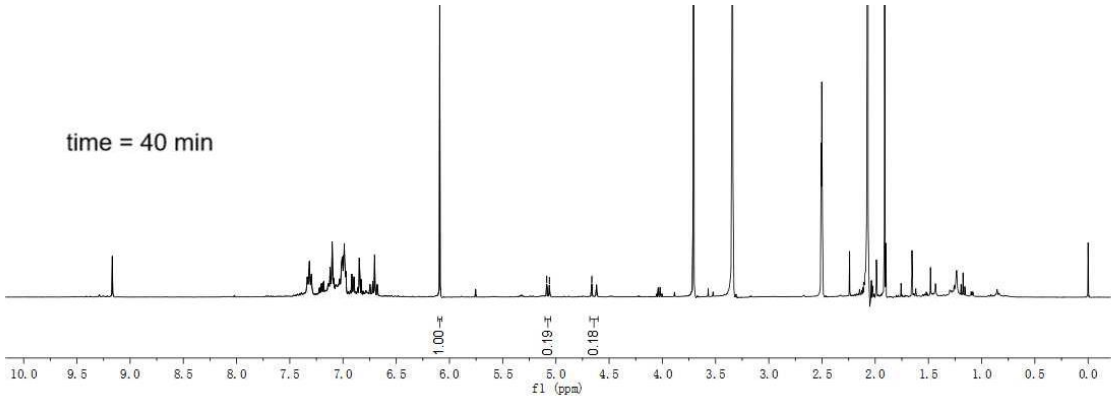
time = 40 min
1.00
0.19
0.18
10.0 9.5 9.0 8.5 8.0 7.5 7.0 6.5 6.0 5.5 5.0 4.5 4.0 3.5 3.0 2.5 2.0 1.5 1.0 0.5 0.0
f1 (ppm)

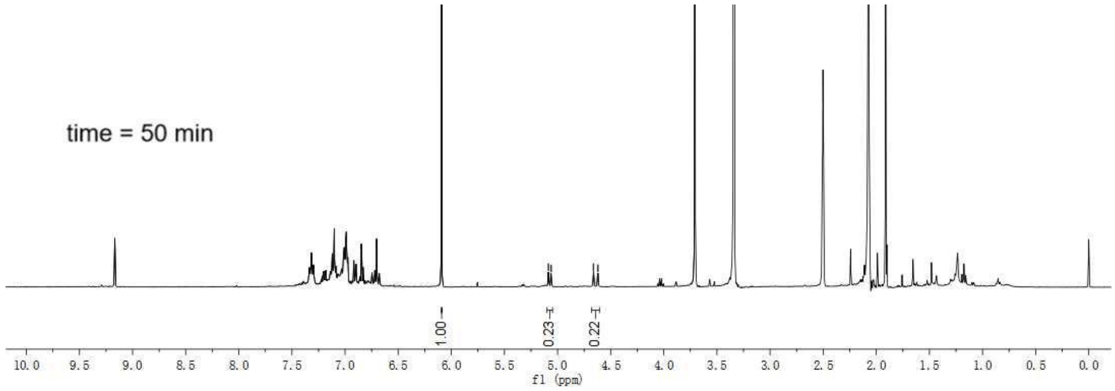
time = 50 min
1.00
0.23
0.22
10.0 9.5 9.0 8.5 8.0 7.5 7.0 6.5 6.0 5.5 5.0 4.5 4.0 3.5 3.0 2.5 2.0 1.5 1.0 0.5 0.0
f1 (ppm)

Crude ^{1}H-NMR of the synthesis for **3a-*d*$_4$** under standard conditions for 50 min:

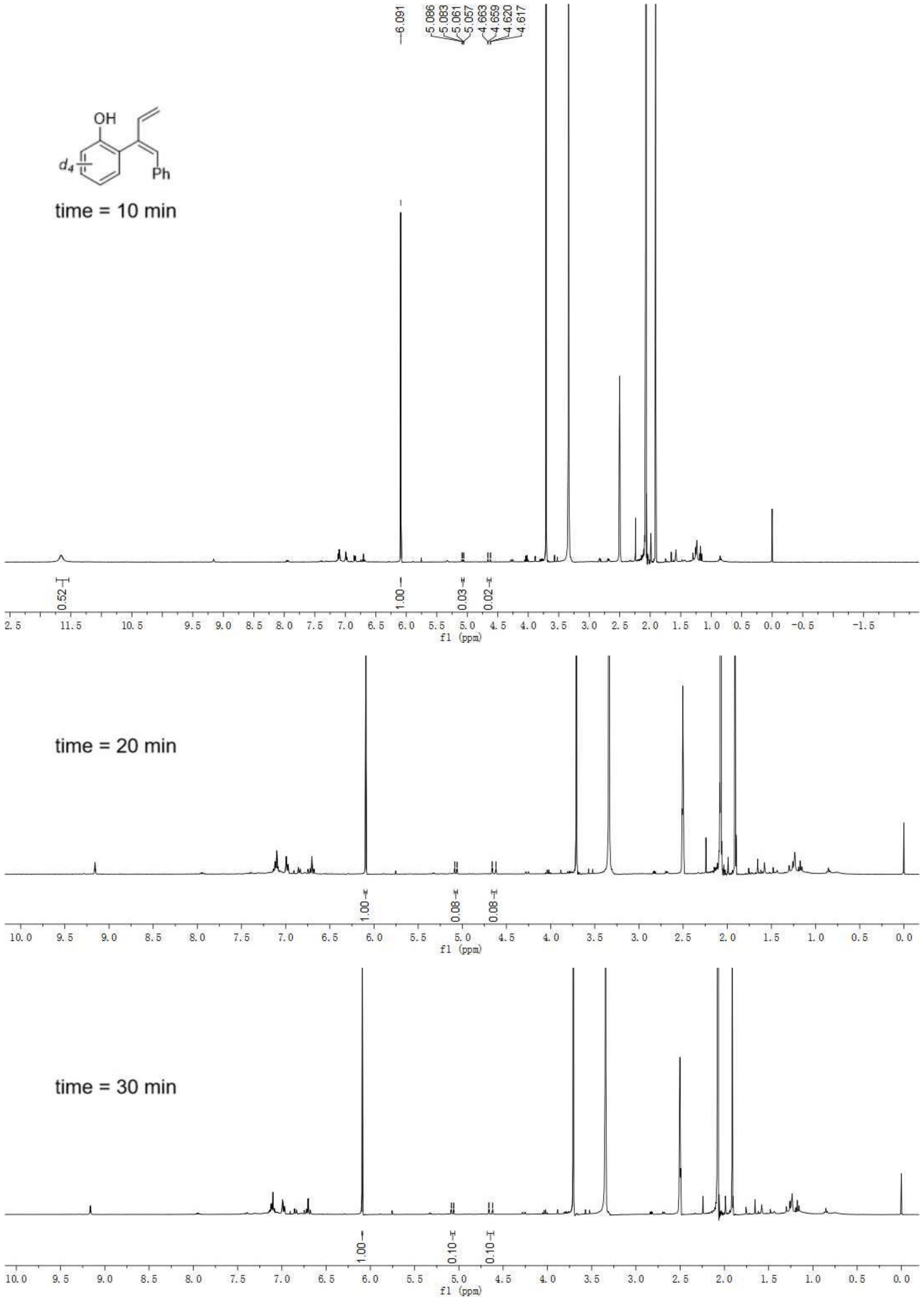

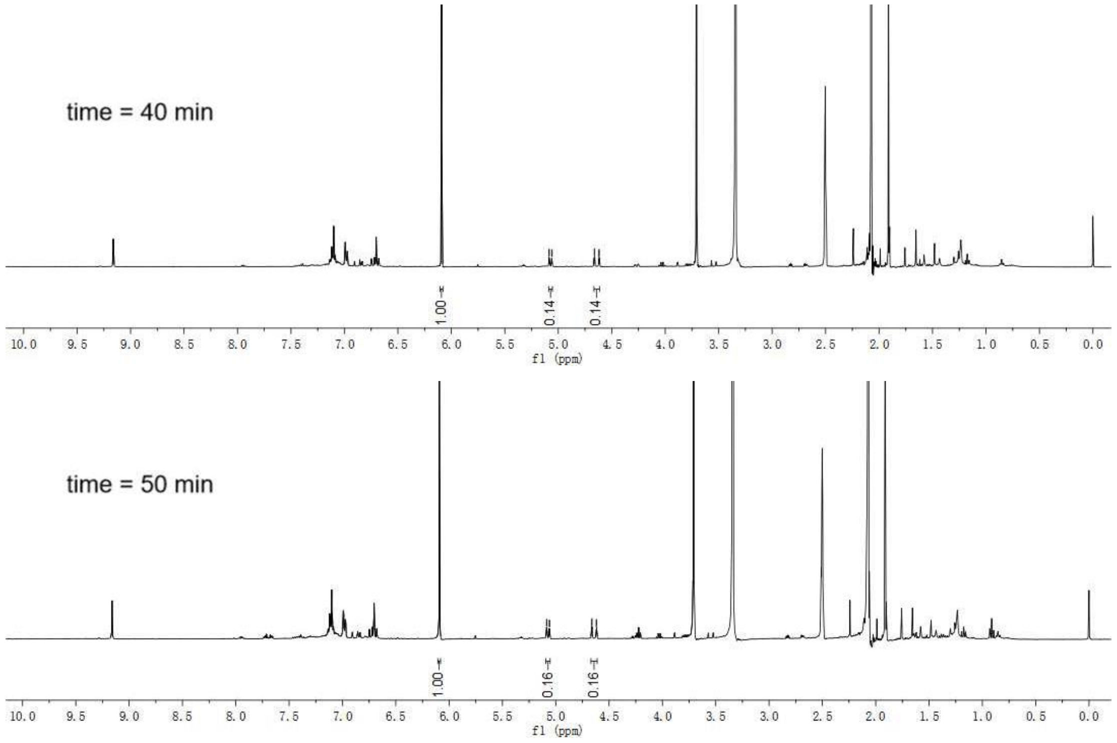

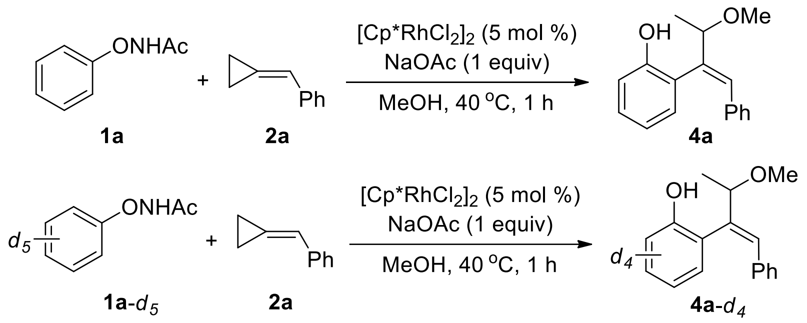

Without any particular precautions to exclude oxygen or moisture, **1a** (30.2 mg, 0.2 mmol, 1.0 equiv) or **1a-*d*₅** (31.2 mg, 0.2 mmol, 1.0 equiv), $[Cp^*RhCl_2]_2$ (6.4 mg, 5 mol %), NaOAc (16.4 mg, 0.2 mmol, 1.0 equiv) and **2a** (0.4 mmol, 2.0 equiv) were added to a 5 mL vial equipped with a stir bar at 40 °C. MeOH (1 mL) was added and timing was started. An aliquot of 0.1 mL was taken every 10 minutes for 1 hours and immediately diluted withEtOAc (1 mL), filtered through a short silica column, washed with 25 mL of EtOAc. The solvent was then removed under reduced pressure and analyzed by ^{1}H-NMR with 1,3,5-trimethoxybenzene as the internal standard. The singlet at δ: 3.51 (CH_3) was used for the calculation of ^{1}H-NMR yields, which were shown below:

entry	time/min	Yield of **4a** (%)	Yield of **4a-*d*₄** (%)

1	10	2	2
2	20	5	5
3	30	9	7
4	40	13	9
5	50	16	12
6	60	20	14

KIE value was obtained by comparing the initial rates measured separately, the slopes of the reaction rate curves were calculated, thus resulted in the KIE = 0.3629/0.2371 = 1.53.

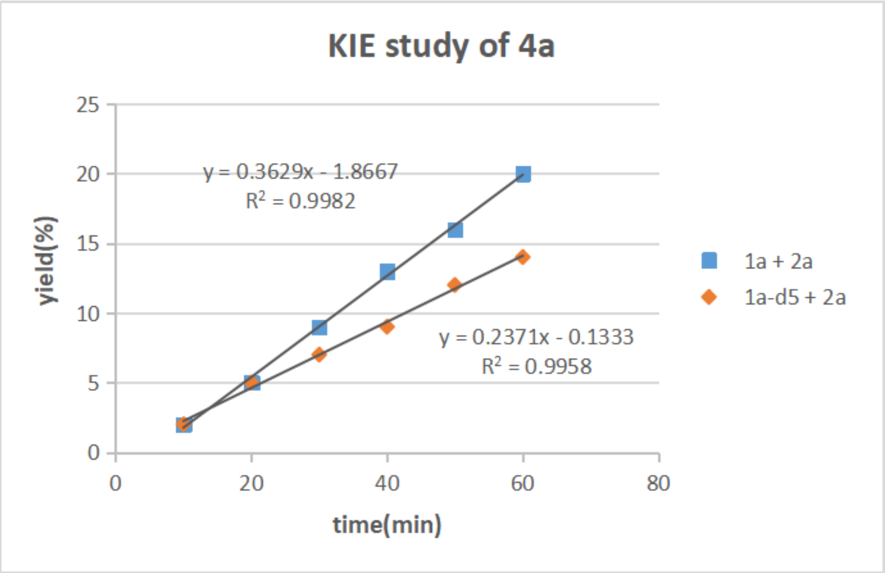

Crude ^{1}H-NMR of the synthesis for **4a** under standard conditions for 1 hour:

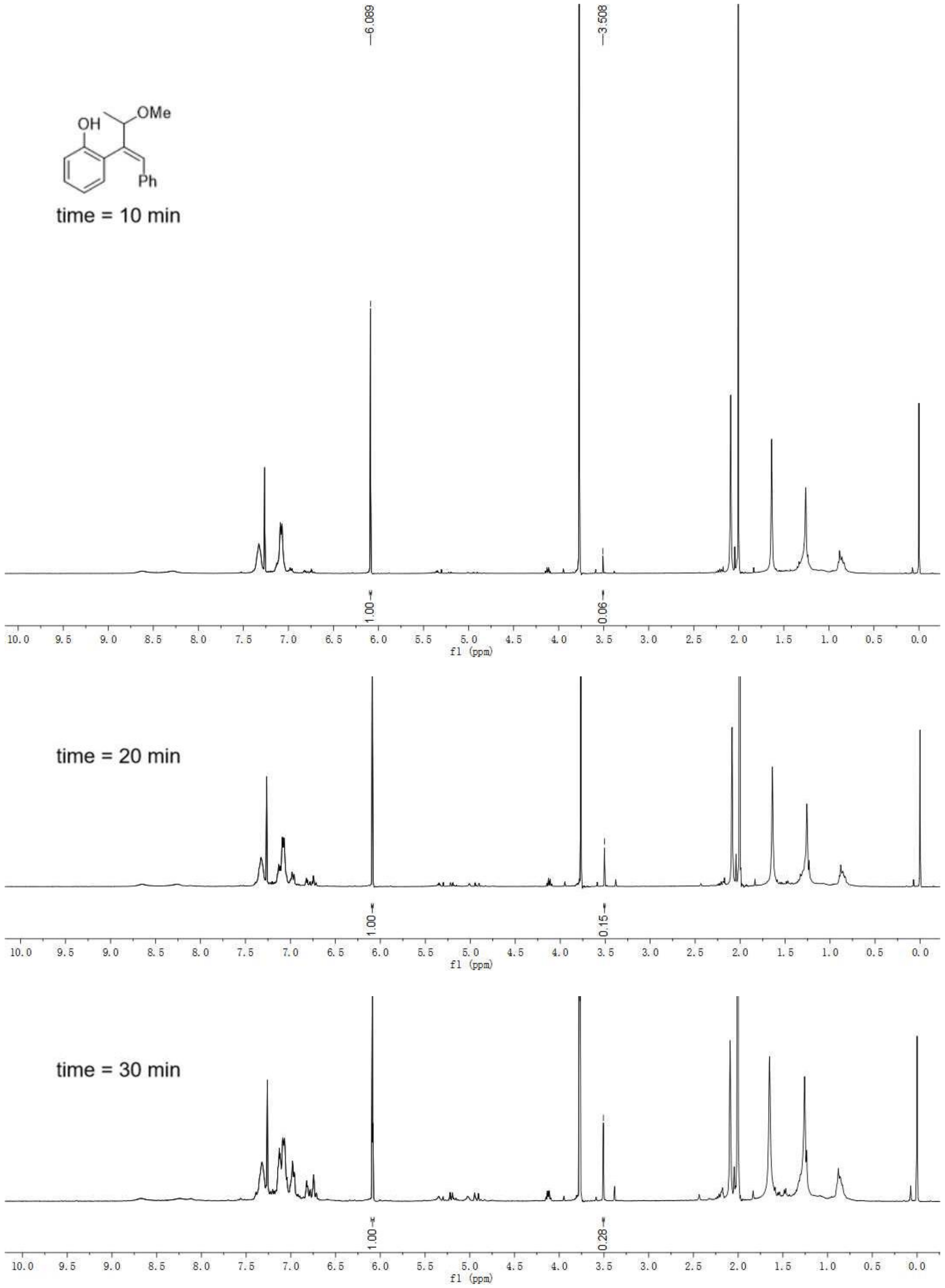

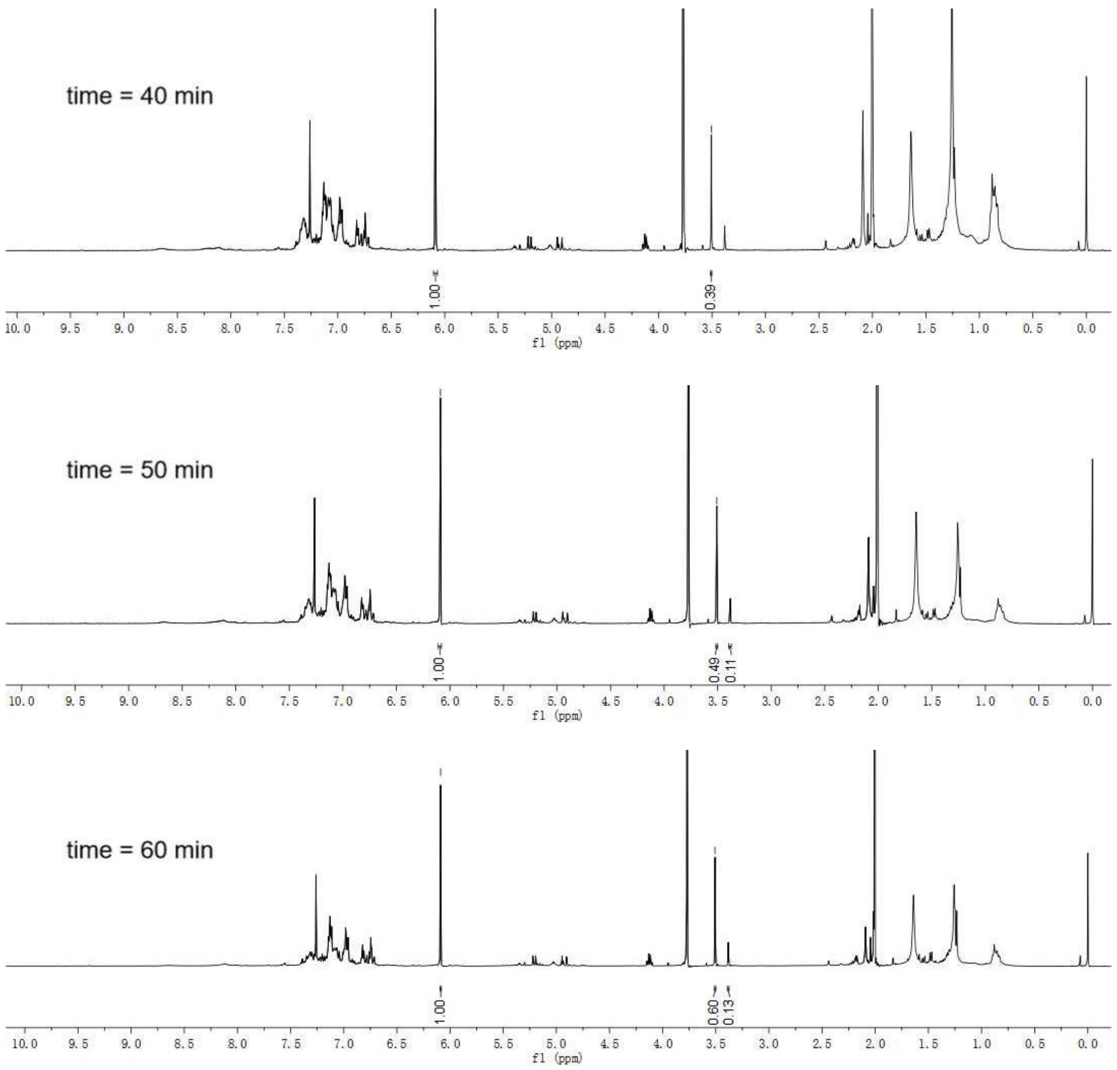
time = 40 min
1.00
0.39
time = 50 min
1.00
0.49
0.11
time = 60 min
1.00
0.60
0.13
f1 (ppm)

Crude ^{1}H-NMR of the synthesis for **4a-*d*$_4$** under standard conditions for 1 hour:

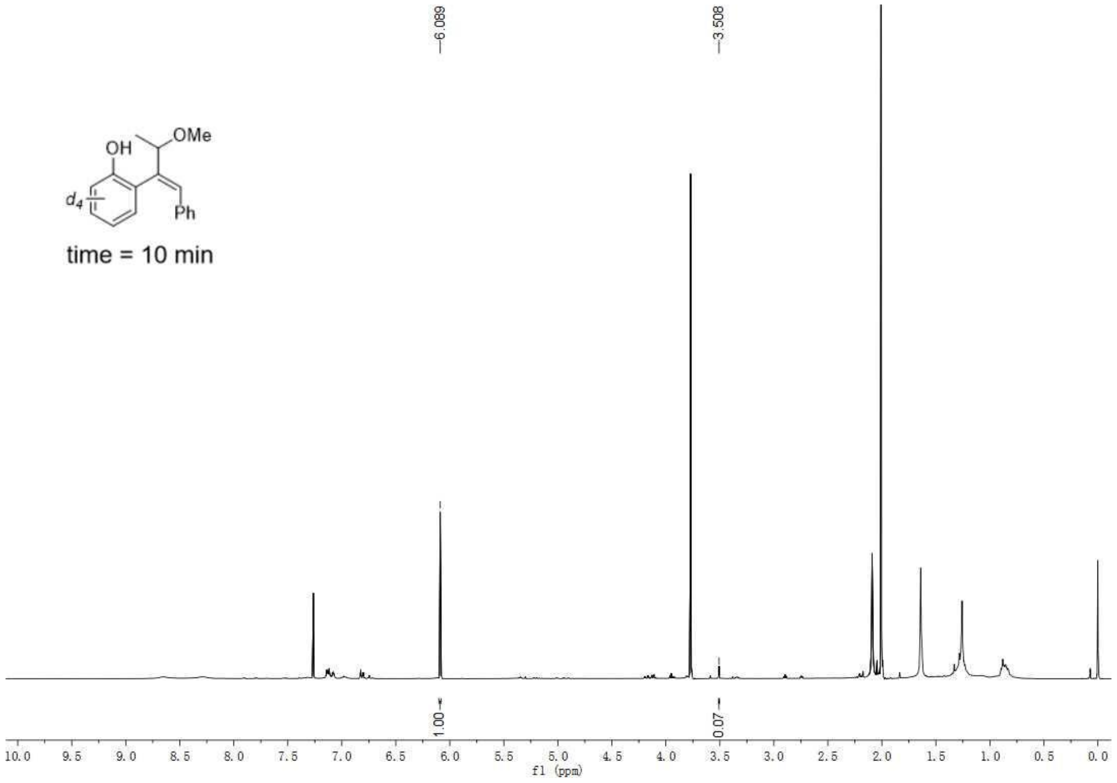

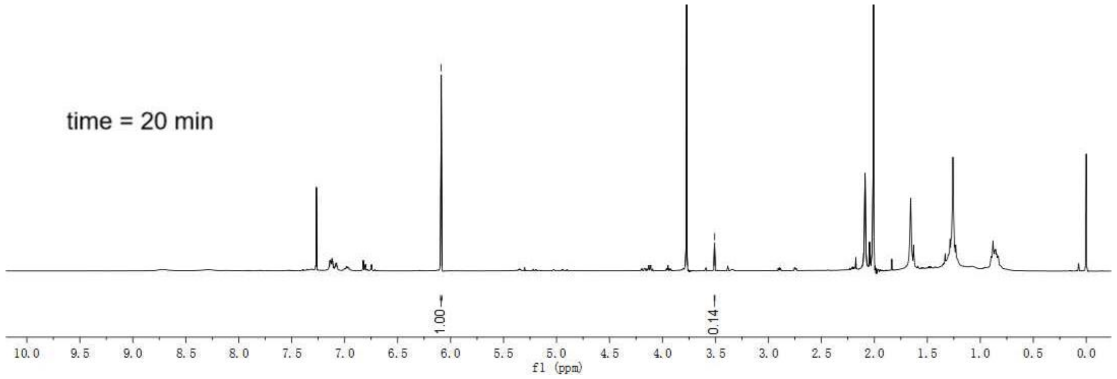

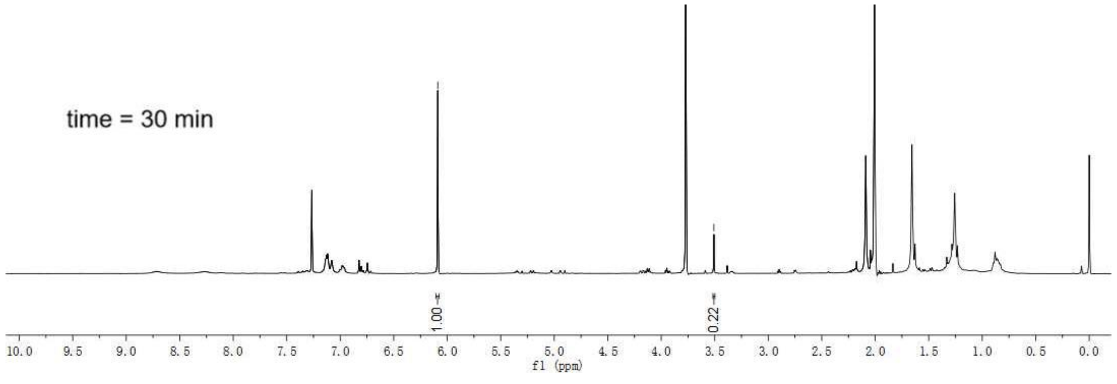

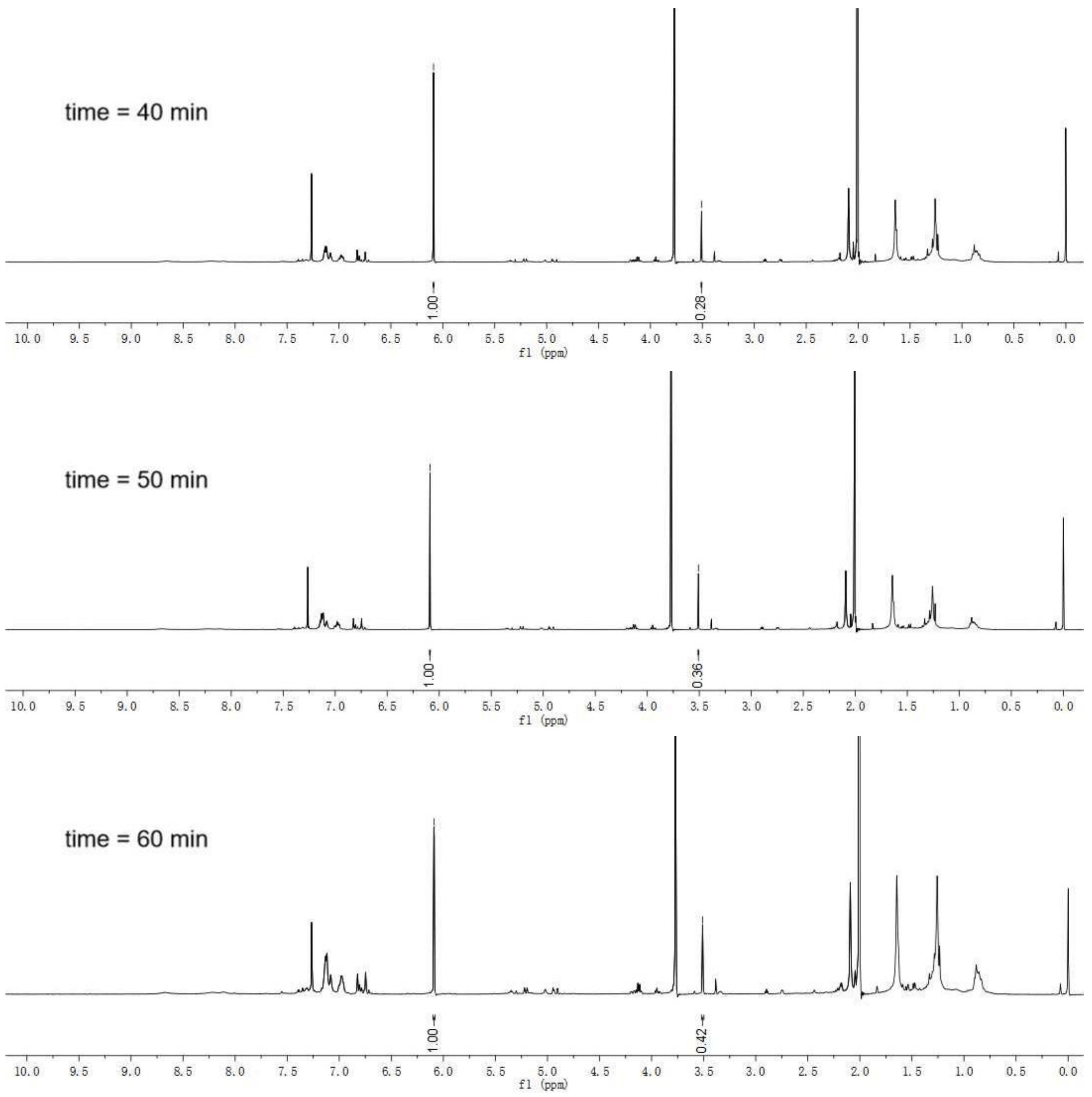

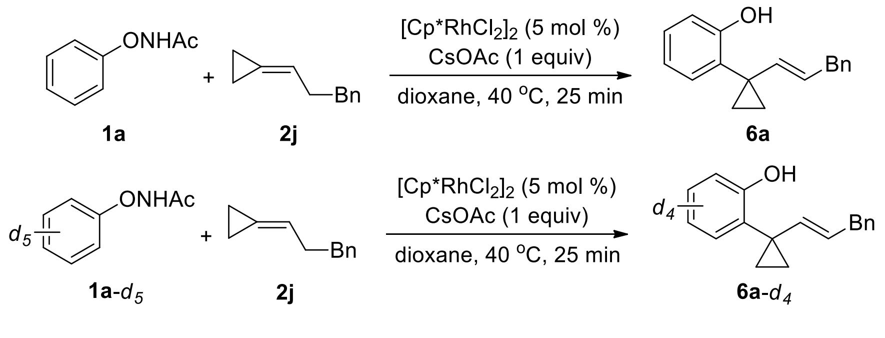

Without any particular precautions to exclude oxygen or moisture, **1a** (30.2 mg, 0.2 mmol, 1.0 equiv) or **1a-*d*5** (31.2 mg, 0.2 mmol, 1.0 equiv), $[Cp^*RhCl_2]_2$ (6.4 mg, 5 mol %), CsOAc (38.4 mg, 0.2 mmol, 1.0 equiv) and **2j** (0.4 mmol, 2.0 equiv) were added to a 5 mL vial equipped with a stir bar at 40 ^{o}C. Dioxane (1 mL) was added and timing was started. An aliquot of 0.1 mL was taken every 5 minutes for 25 minutes

and immediately diluted with EtOAc (1 mL), filtered through a short silica column, washed with 25 mL of EtOAc. The solvent was then removed under reduced pressure and analyzed by ^{1}H-NMR with 1,3,5-trimethoxybenzene as the internal standard. The doublet at δ: 3.37 (CH_2) was used for calculation of ^{1}H-NMR yields, which were shown below:

entry	time/min	Yield of 6**a** (%)	Yield of 6**a**-***d*4** (%)
1	5	13	12
2	10	19	17
3	15	24	22
4	20	28	26
5	25	33	30

KIE value was obtained by comparing the initial rates measured separately, the slopes of the reaction rate curves were calculated, thus resulted in the KIE = 0.98/0.90 = 1.09.

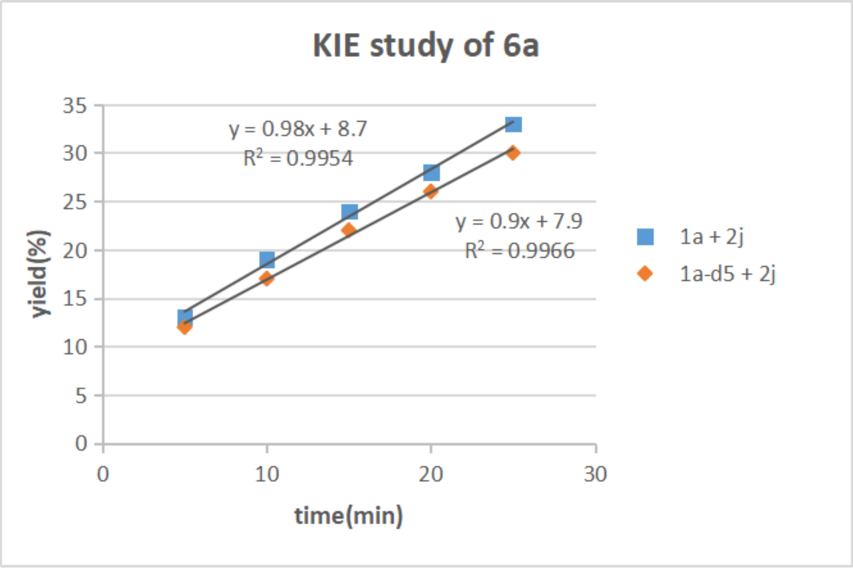

Crude ^{1}H-NMR of the synthesis for **6a** under standard conditions for 25 min:

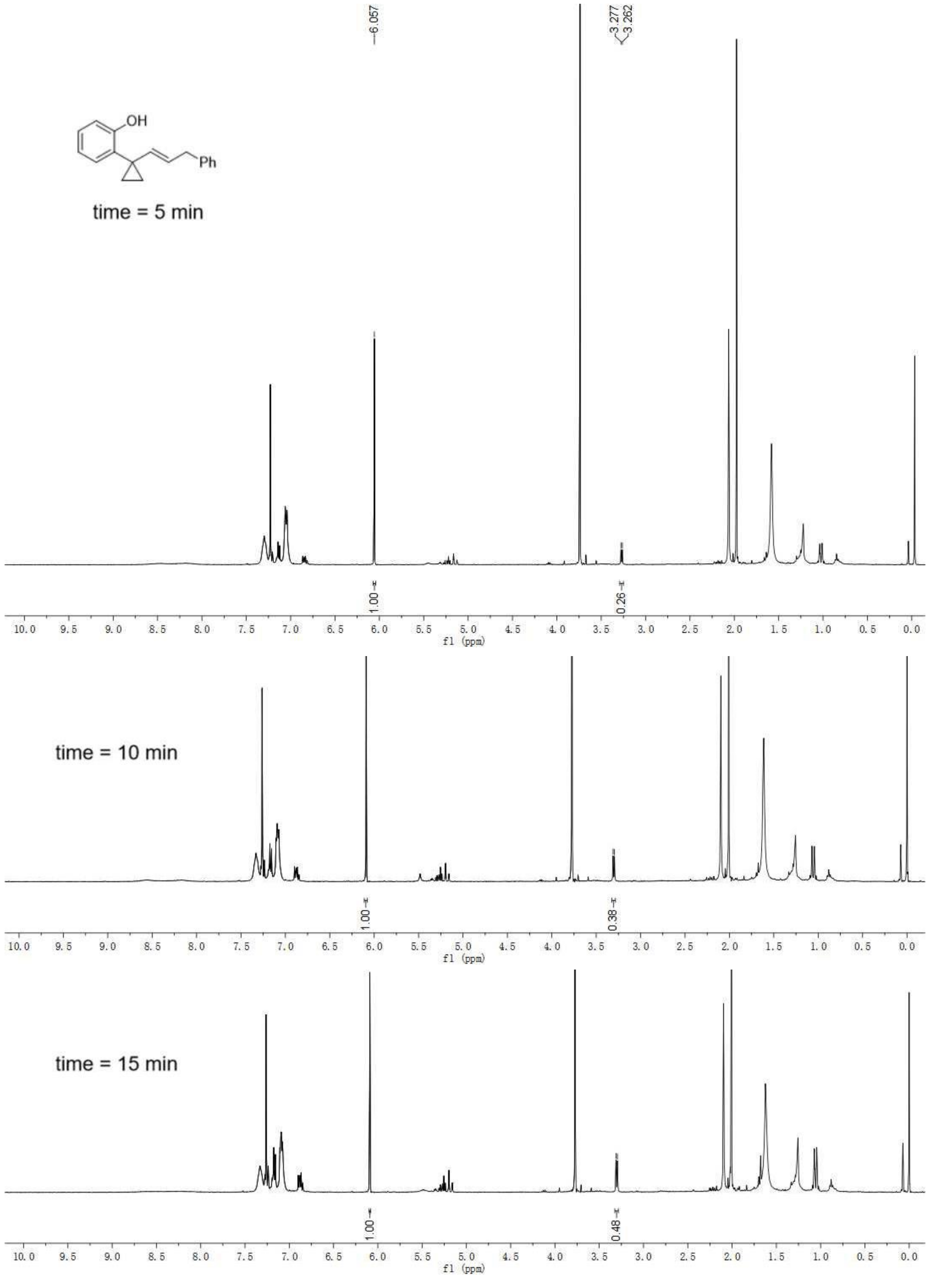

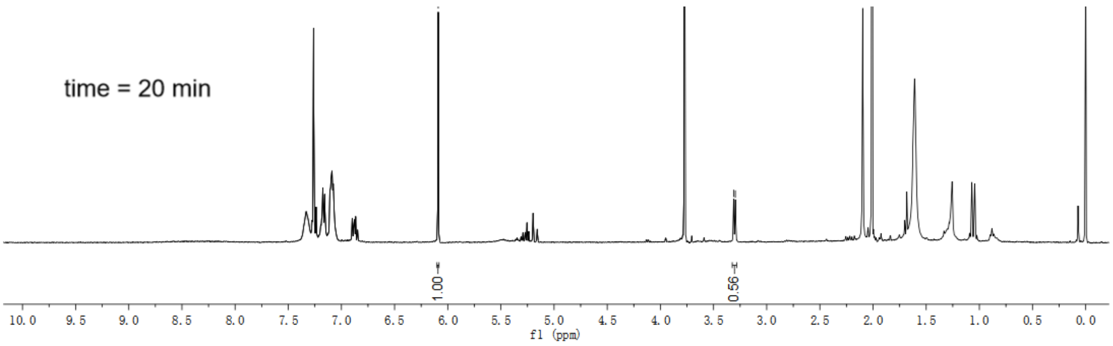

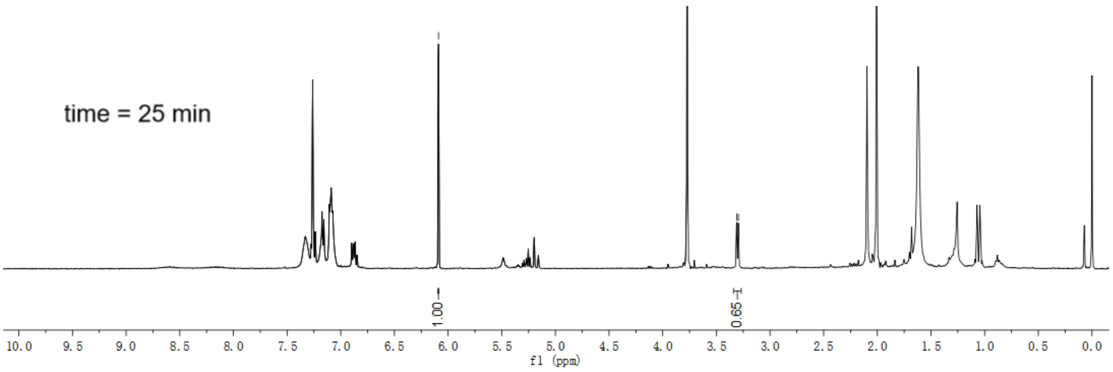

Crude ^{1}H-NMR of the synthesis for **6a-*d*$_4$** under standard conditions for 25 min:

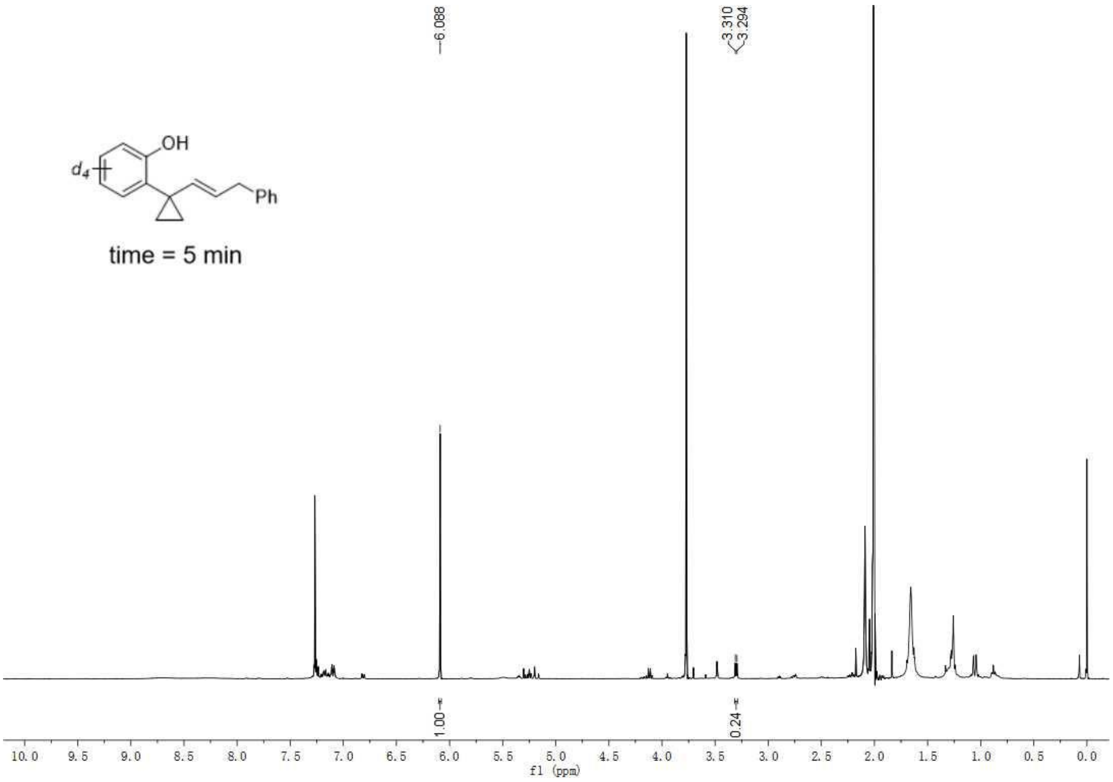

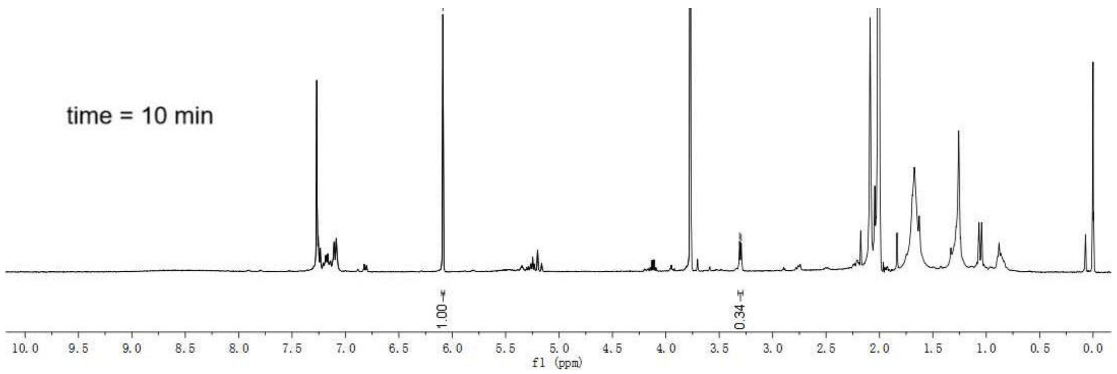

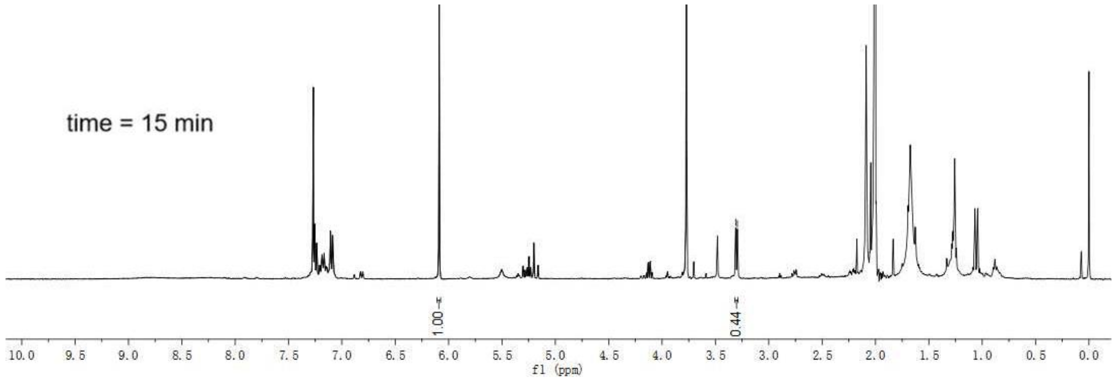

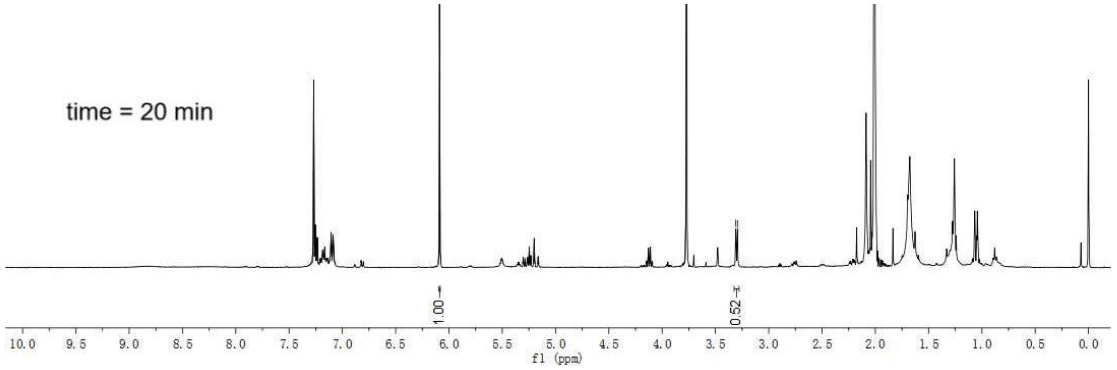

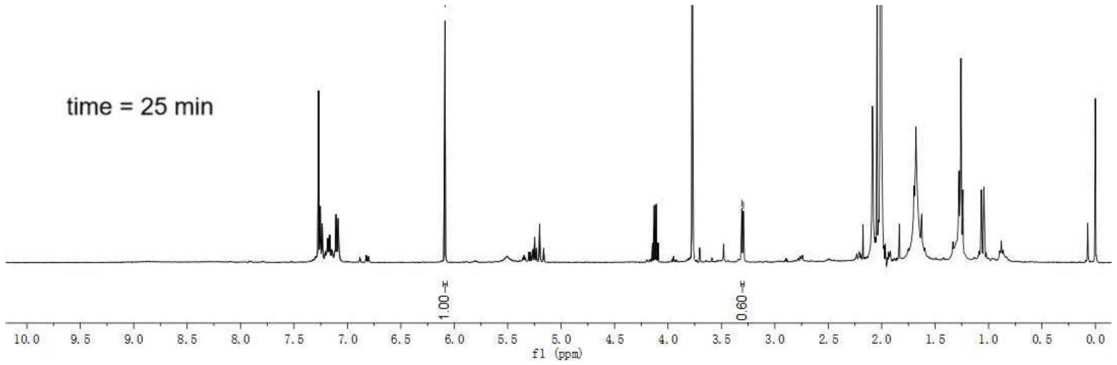

Defining the five-membered rhodacycle as the active intermediate:

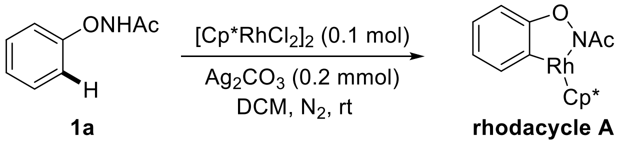

1a **rhodacycle A**

The mixture of $[Cp^*RhCl_2]_2$ (0.1 mmol, 1.0 equiv), Ag_2CO_3 (0.2 mmol, 2.0

equiv) and *N*-phenoxyacetamide **1a** (0.2 mmol, 2.0 equiv) in DCM (2.0 mL) was stirred for 12 h at room temperature. Afterwards, the filtration was conducted to remove any precipitate with celite. The solvent was removed under reduced pressure to afford the analytically pure rhodacycle complex in 85% yield. The rhodacycle complex is a known compound and all data were in agreement with those reported.[S3]

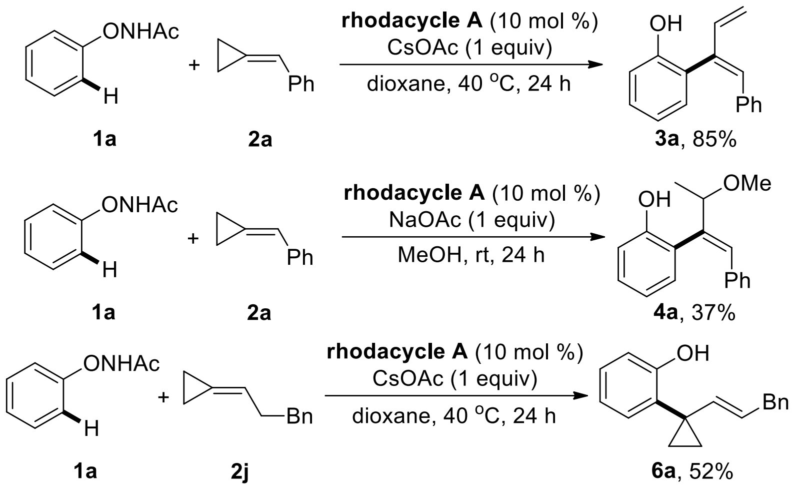

The mixture of *N*-phenoxyacetamide **1a** (0.1 mmol, 1.0 equiv), methylenecyclopropanes (0.2 mmol, 2.0 equiv), rhodacycle A (5 mol %) and base (0.1 mmol, 1.0 equiv) in corresponding solvent (0.5 mL) was stirred at room temperature or 40 °C for 24 h without exclusion of air or moisture. Afterwards, the solvent was removed under reduced pressure, and the resulted mixture was purified by preparative TLC to afford the corresponding products **3a**, **4a** or **6a**.

Kinetics of the C-H cyclopropylation with $[Cp^*RhCl_2]_2$ or rhodacycle A:

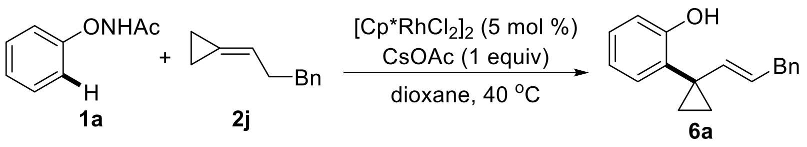

Without any particular precautions to exclude oxygen or moisture, **1a** (45.3 mg, 0.3mmol, 1.0 equiv), $[Cp^*RhCl_2]_2$ (9.27 mg, 0.015mmol, 5 mol %), CsOAc (57.6 mg, 0.3mmol, 1.0 equiv) and **2a** (0.6mmol, 2.0 equiv) were added to a 10 mL vial equipped with a stir bar. Dioxane (1.5 mL) was added and timing was started. An aliquot of 0.1 mL was taken every 2 minutes for 20 minutes and immediately diluted with EtOAc (1 mL), filtered through a short silica column, washed with 15 mL of

EtOAc. The solvent was then removed under reduced pressure and analyzed by ^{1}H-NMR with 1,3,5-trimethoxybenzene as the internal standard. The doublet at δ: 3.31 (-CH_2-) was used for the calculation of ^{1}H-NMR yields.

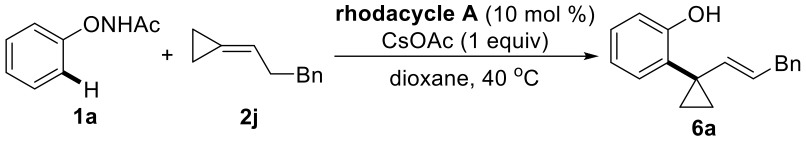

Without any particular precautions to exclude oxygen or moisture, **1a** (45.3 mg, 0.3mmol, 1.0 equiv), rhodacycle **A** (11.61 mg, 0.03 mmol, 10 mol %), CsOAc (57.6 mg, 0.3 mmol, 1.0 equiv) and **2a** (0.6 mmol, 2.0 equiv) were added to a 10 mL vial equipped with a stir bar. Dioxane (1.5 mL) was added and timing was started. An aliquot of 0.1 mL was taken every 1minutes for 14 minutes and immediately diluted with EtOAc (1 mL), filtered through a short silica column, washed with 15 mL of EtOAc. The solvent was then removed under reduced pressure and analyzed by ^{1}H-NMR with 1,3,5-trimethoxybenzene as the internal standard. The doublet at δ: 3.31 (-CH_2-) was used for the calculation of ^{1}H-NMR yields.

Supplementary Table 3. Time course and ^{1}H-NMR yields of **6a** with different catalyst

time/min	Yield/%		time/min	Yield/%	
	[Cp*RhCl2]2	rhodacycle**A**		[Cp*RhCl2]2	rhodacycle**A**
1	-	21	10	41	39
2	1	24	11	-	39
3	-	30	12	47	40
4	13	32	13	-	40
5	-	34	14	50	41
6	26	35	16	52	-
7	-	37	18	54	-
8	33	38	20	57	-
9	-	38			

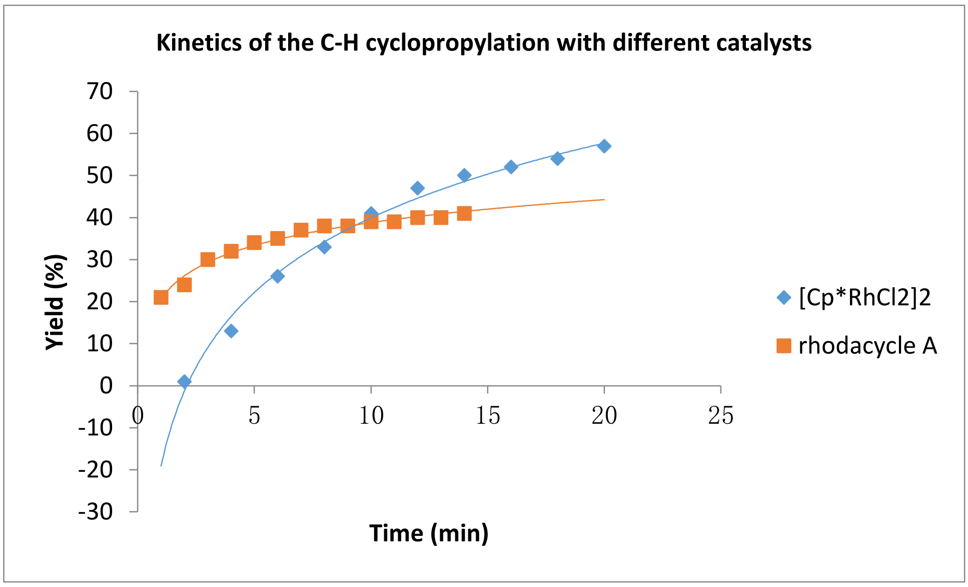

Crude ^{1}H-NMR of **6a** under the catalysis of $[Cp^*RhCl_2]_2$

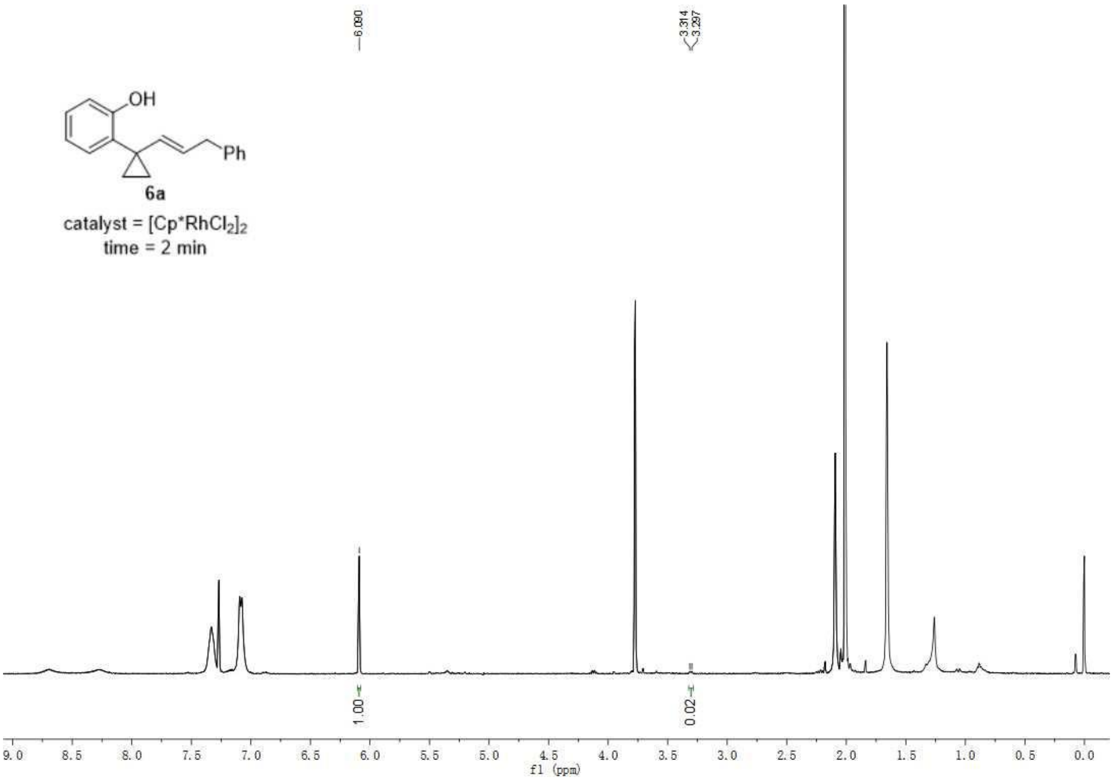

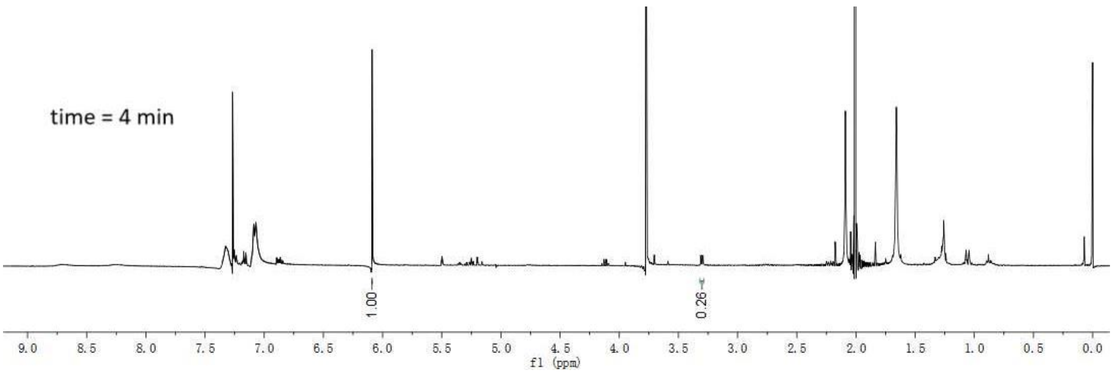
time = 4 min
1.00
0.26
9.0 8.5 8.0 7.5 7.0 6.5 6.0 5.5 5.0 4.5 4.0 3.5 3.0 2.5 2.0 1.5 1.0 0.5 0.0
f1 (ppm)

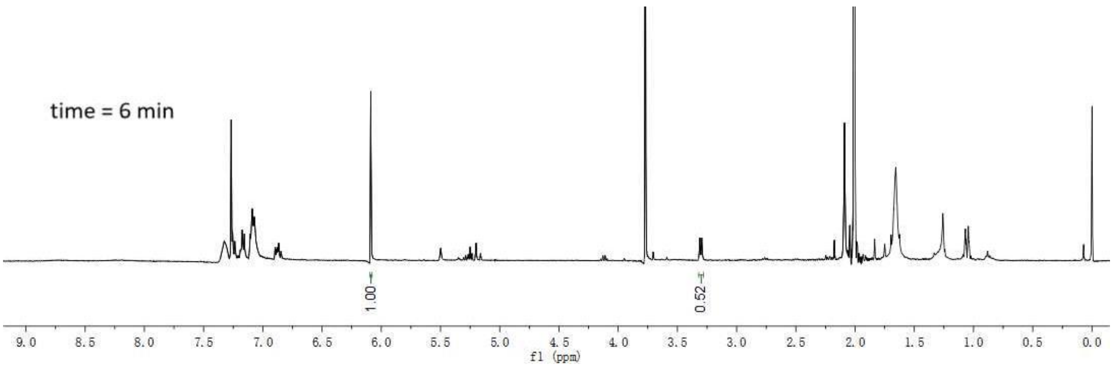
time = 6 min
1.00
0.52
9.0 8.5 8.0 7.5 7.0 6.5 6.0 5.5 5.0 4.5 4.0 3.5 3.0 2.5 2.0 1.5 1.0 0.5 0.0
f1 (ppm)

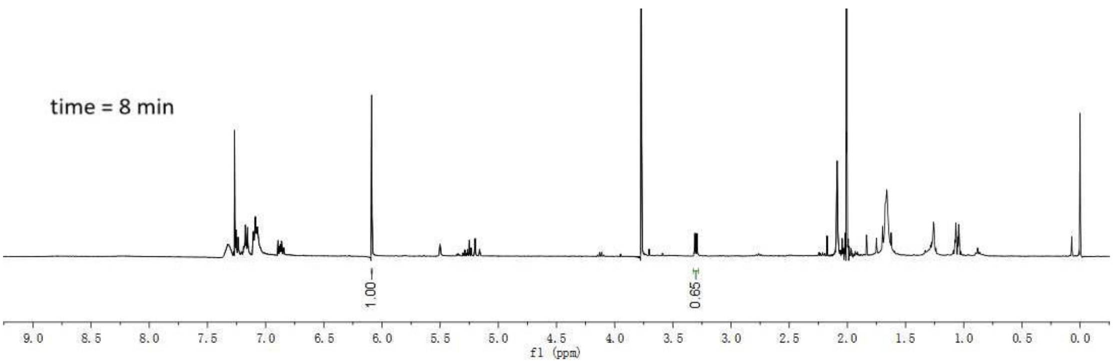
time = 8 min
1.00
0.65
9.0 8.5 8.0 7.5 7.0 6.5 6.0 5.5 5.0 4.5 4.0 3.5 3.0 2.5 2.0 1.5 1.0 0.5 0.0
f1 (ppm)

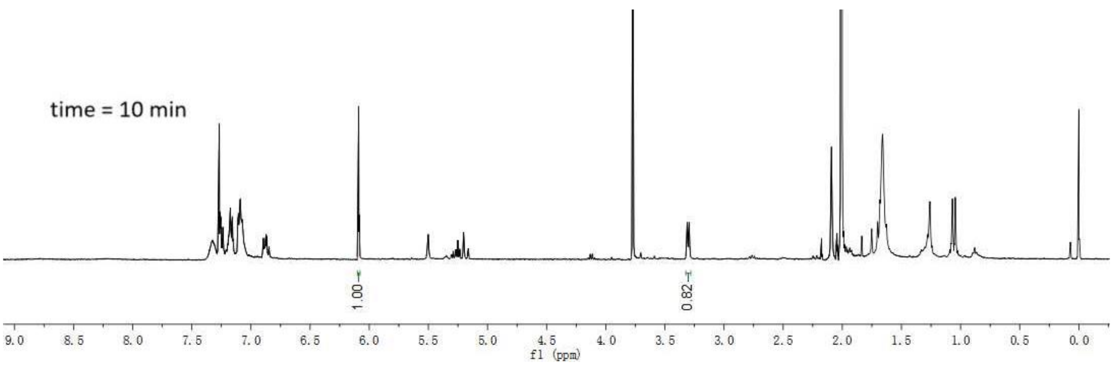
time = 10 min
1.00
0.82
9.0 8.5 8.0 7.5 7.0 6.5 6.0 5.5 5.0 4.5 4.0 3.5 3.0 2.5 2.0 1.5 1.0 0.5 0.0
f1 (ppm)

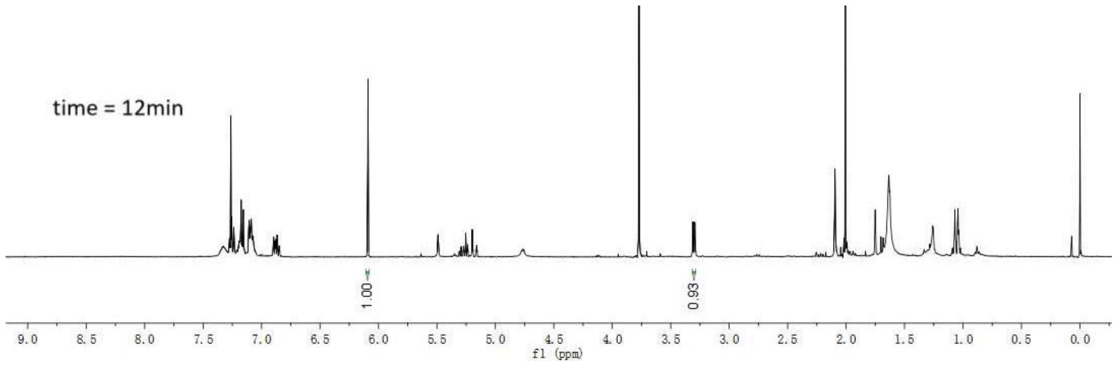
time = 12min
1.00
0.93
9.0 8.5 8.0 7.5 7.0 6.5 6.0 5.5 5.0 4.5 4.0 3.5 3.0 2.5 2.0 1.5 1.0 0.5 0.0
f1 (ppm)

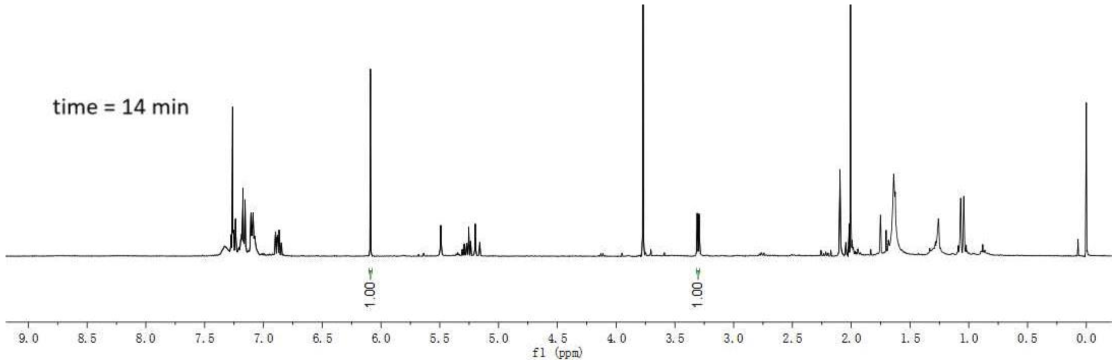
time = 14 min
1.00
1.00
9.0 8.5 8.0 7.5 7.0 6.5 6.0 5.5 5.0 4.5 4.0 3.5 3.0 2.5 2.0 1.5 1.0 0.5 0.0
f1 (ppm)

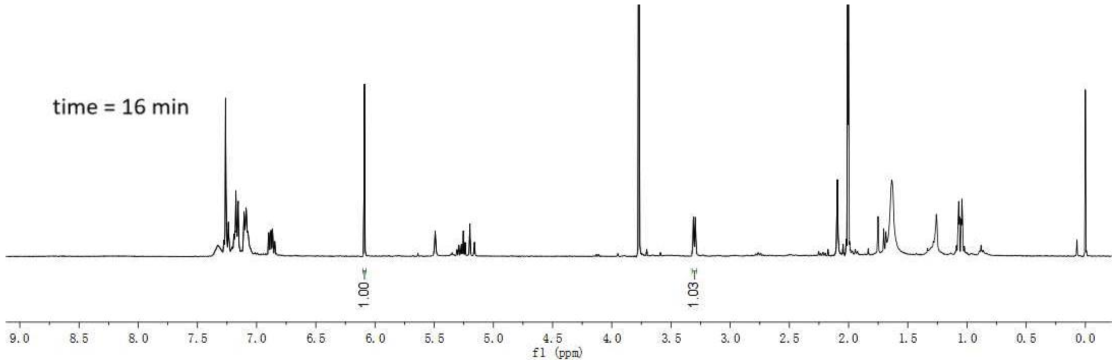
time = 16 min
1.00
1.03
9.0 8.5 8.0 7.5 7.0 6.5 6.0 5.5 5.0 4.5 4.0 3.5 3.0 2.5 2.0 1.5 1.0 0.5 0.0
f1 (ppm)

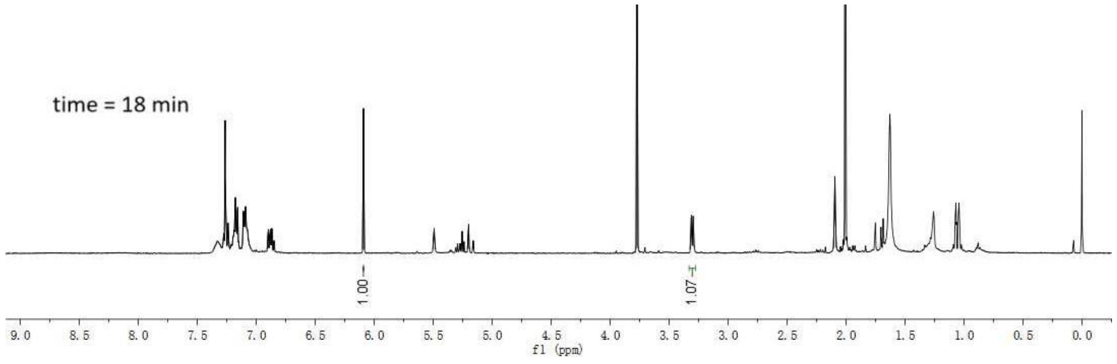
time = 18 min
1.00
1.07
9.0 8.5 8.0 7.5 7.0 6.5 6.0 5.5 5.0 4.5 4.0 3.5 3.0 2.5 2.0 1.5 1.0 0.5 0.0
f1 (ppm)

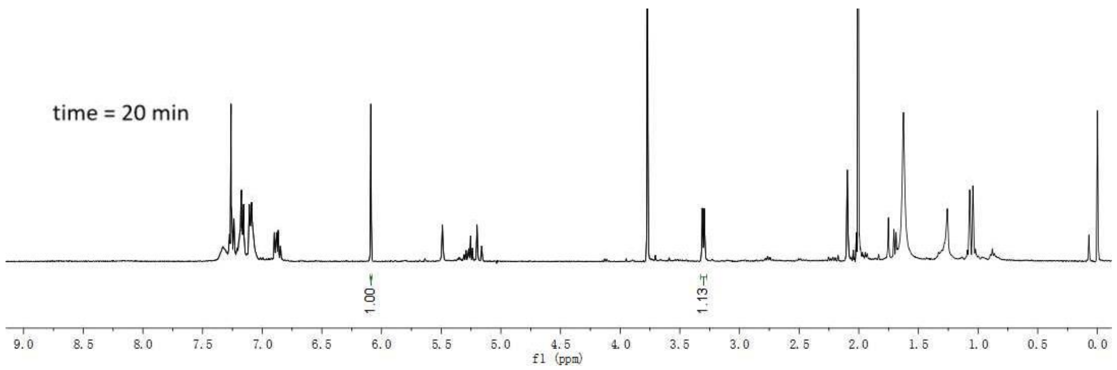

Crude ^{1}H-NMR of **6a** under the catalysis of rhodacycle**A**

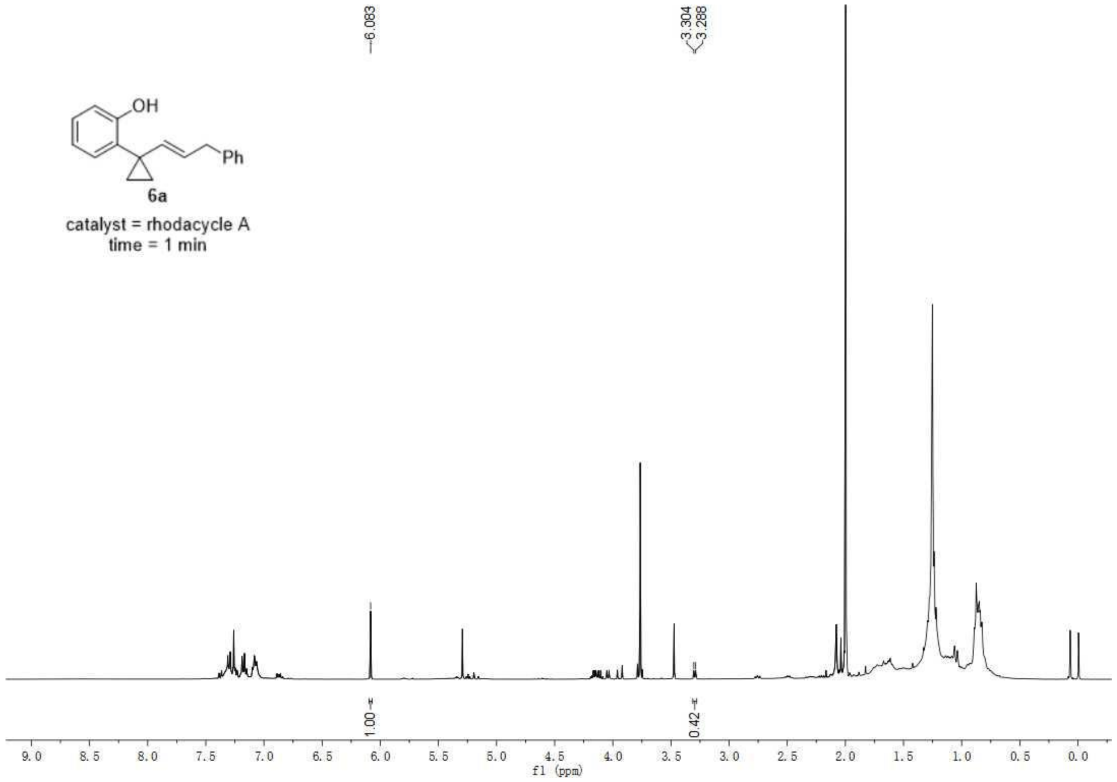

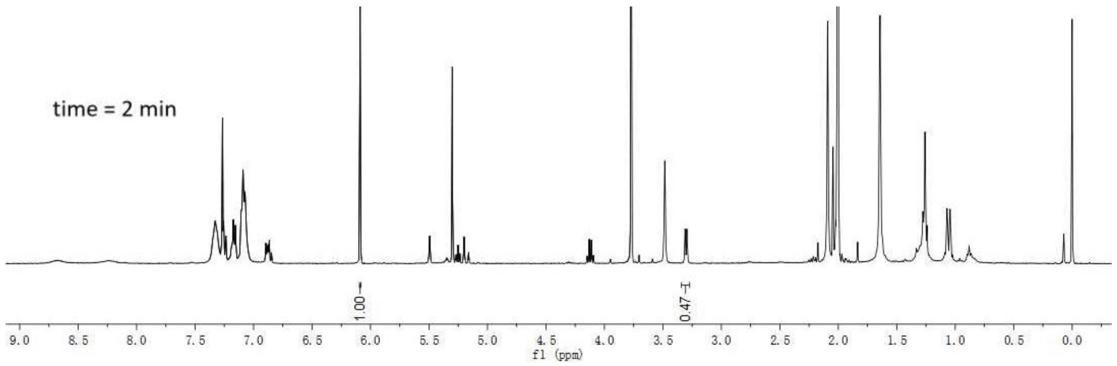

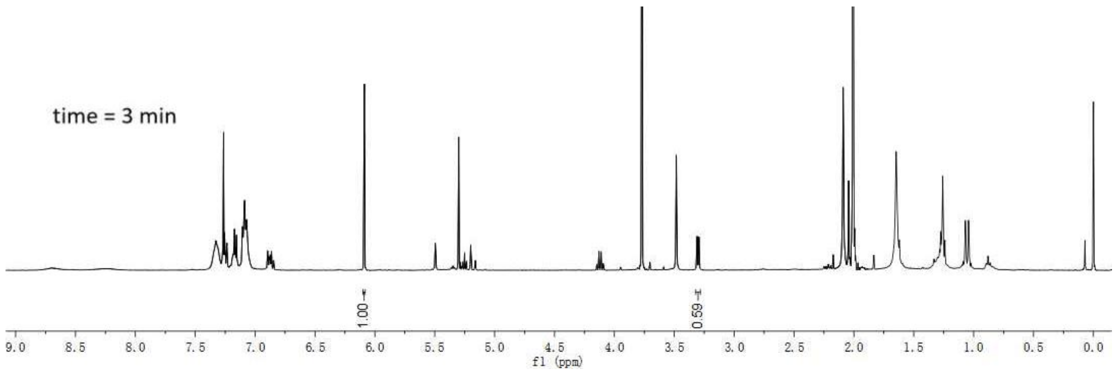
time = 3 min
1.00
0.59
9.0 8.5 8.0 7.5 7.0 6.5 6.0 5.5 5.0 4.5 4.0 3.5 3.0 2.5 2.0 1.5 1.0 0.5 0.0
f1 (ppm)

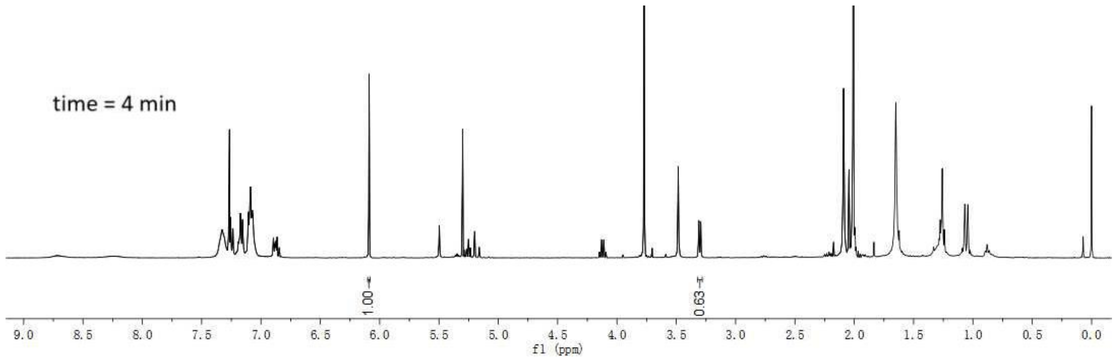
time = 4 min
1.00
0.63
9.0 8.5 8.0 7.5 7.0 6.5 6.0 5.5 5.0 4.5 4.0 3.5 3.0 2.5 2.0 1.5 1.0 0.5 0.0
f1 (ppm)

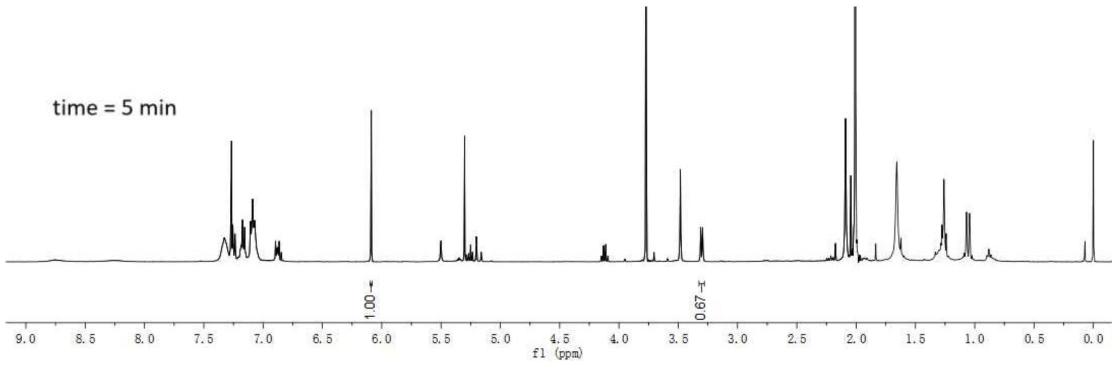
time = 5 min
1.00
0.67
9.0 8.5 8.0 7.5 7.0 6.5 6.0 5.5 5.0 4.5 4.0 3.5 3.0 2.5 2.0 1.5 1.0 0.5 0.0
f1 (ppm)

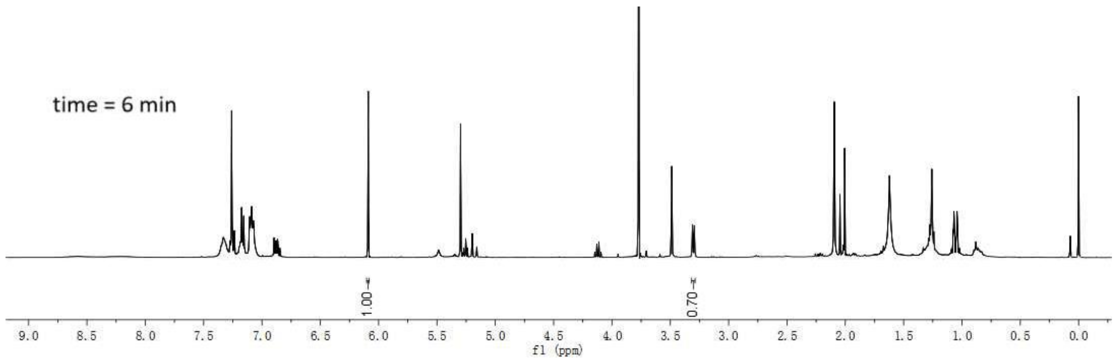
time = 6 min
1.00
0.70
9.0 8.5 8.0 7.5 7.0 6.5 6.0 5.5 5.0 4.5 4.0 3.5 3.0 2.5 2.0 1.5 1.0 0.5 0.0
f1 (ppm)

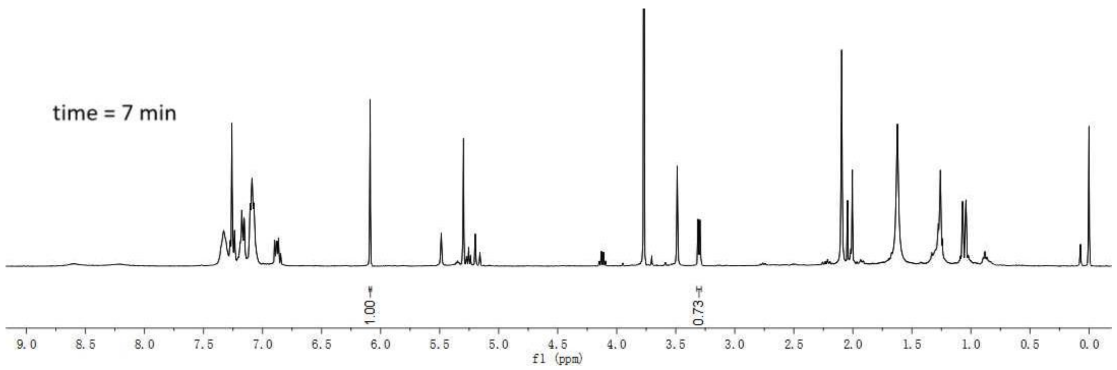
time = 7 min
1.00
0.73
9.0 8.5 8.0 7.5 7.0 6.5 6.0 5.5 5.0 4.5 4.0 3.5 3.0 2.5 2.0 1.5 1.0 0.5 0.0
f1 (ppm)

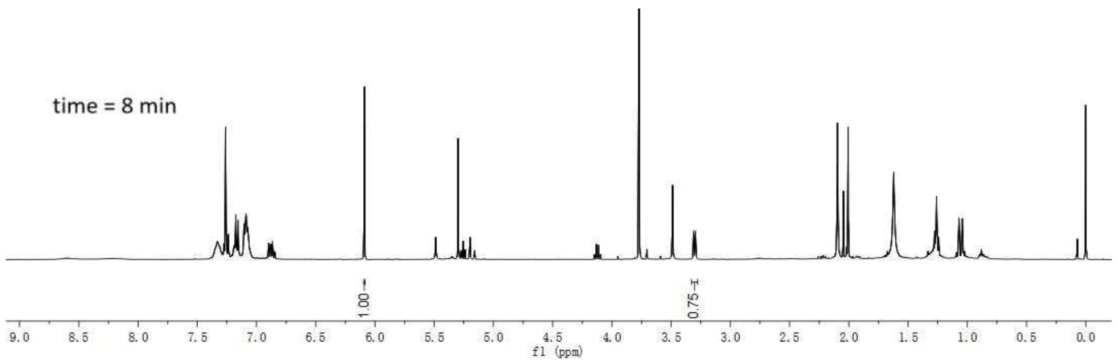
time = 8 min
1.00
0.75
9.0 8.5 8.0 7.5 7.0 6.5 6.0 5.5 5.0 4.5 4.0 3.5 3.0 2.5 2.0 1.5 1.0 0.5 0.0
f1 (ppm)

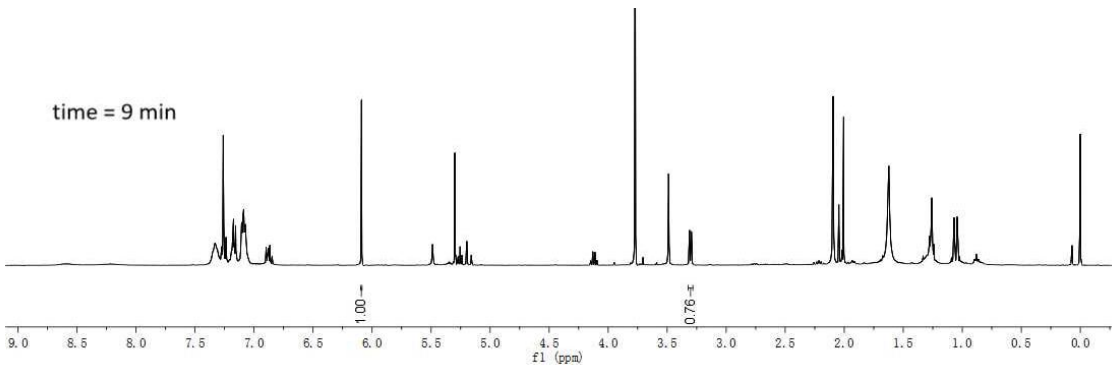
time = 9 min
1.00
0.76
9.0 8.5 8.0 7.5 7.0 6.5 6.0 5.5 5.0 4.5 4.0 3.5 3.0 2.5 2.0 1.5 1.0 0.5 0.0
f1 (ppm)

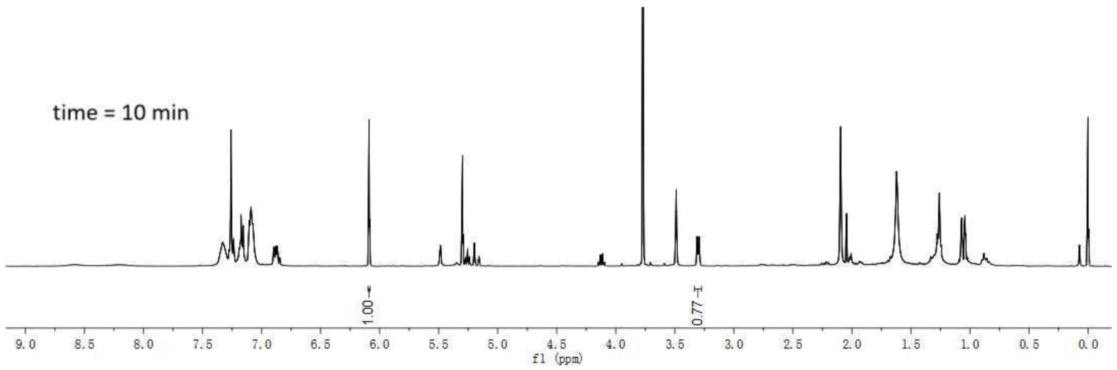
time = 10 min
1.00
0.77
9.0 8.5 8.0 7.5 7.0 6.5 6.0 5.5 5.0 4.5 4.0 3.5 3.0 2.5 2.0 1.5 1.0 0.5 0.0
f1 (ppm)

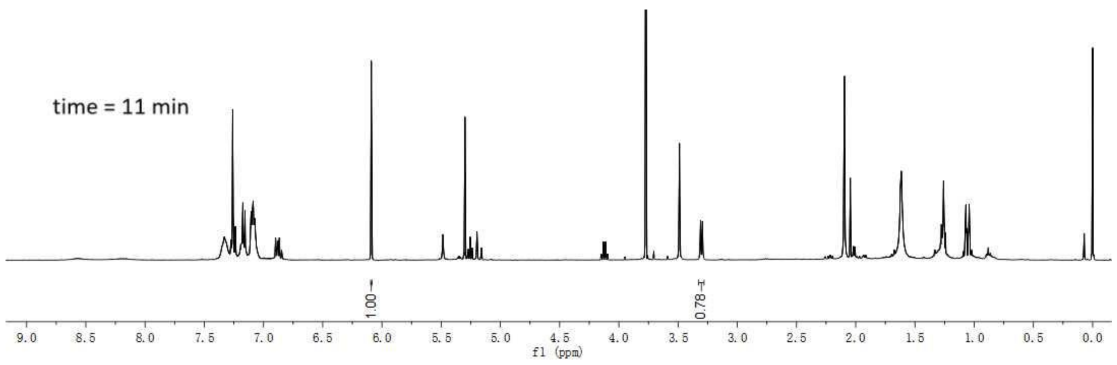
time = 11 min
1.00
0.78
9.0 8.5 8.0 7.5 7.0 6.5 6.0 5.5 5.0 4.5 4.0 3.5 3.0 2.5 2.0 1.5 1.0 0.5 0.0
f1 (ppm)

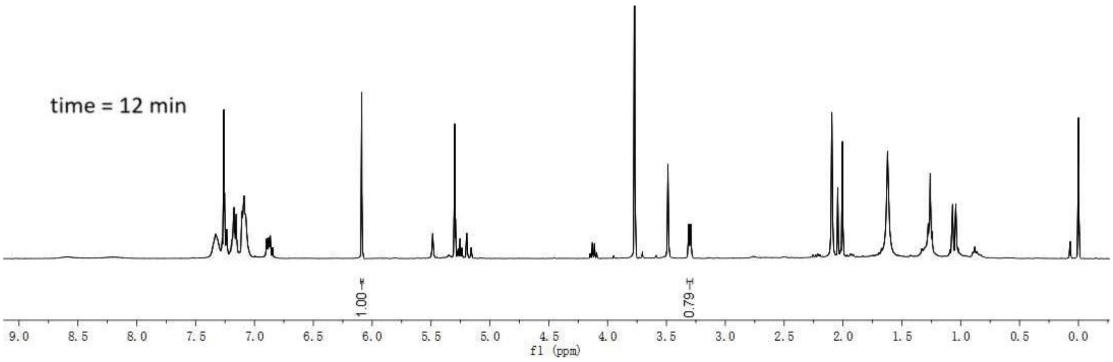

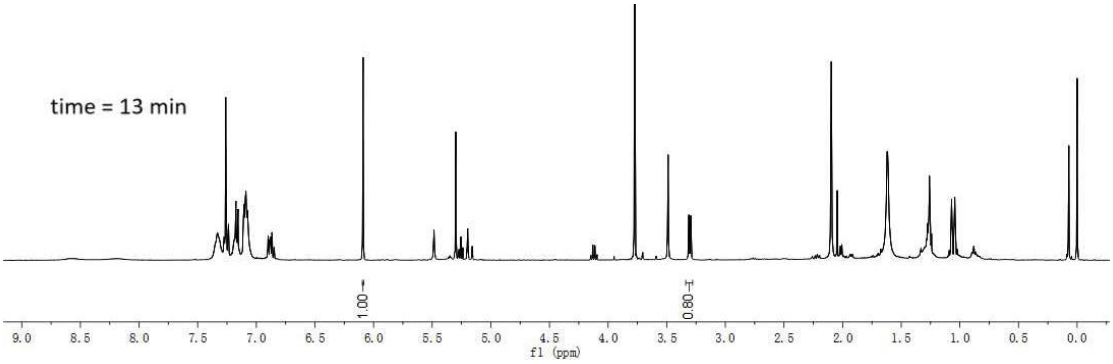

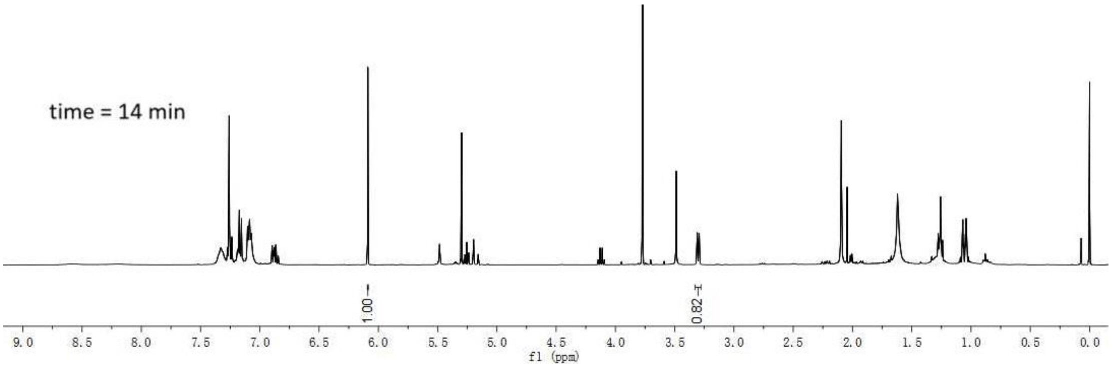

Control experiment:

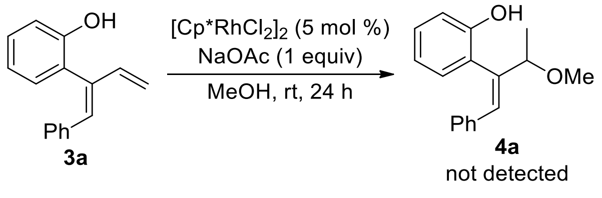

The mixture of diene**3a**(0.1 mmol, 1.0 equiv), $[Cp^*RhCl_2]_2$ (5 mol %) and NaOAc (0.1 mmol, 1.0 equiv) in MeOH (0.5 mL) was stirred at room temperature for 24 h without exclusion of air or moisture. Afterwards, the reaction was monitored by TLC and no **4a** was detected.

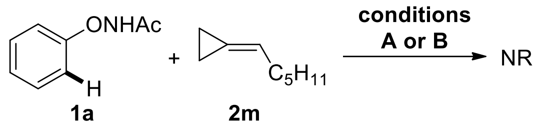

The reaction between *N*-phenoxyacetamide **1a** and hexylidenecyclopropane **2m** was tested under standard conditions A or B, and resulted in the recovery of both substrates.

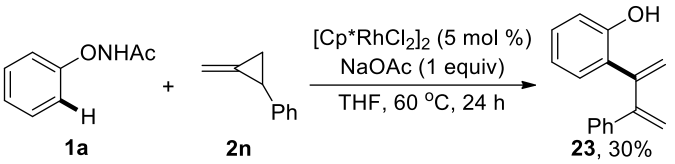

The mixture of *N*-phenoxyacetamide **1a** (0.1 mmol, 1.0 equiv), (2-methylenecyclopropyl)benzene **2n** (0.2 mmol, 2.0 equiv), $[Cp^*RhCl_2]_2$(5 mol %) and NaOAc (0.1 mmol, 1.0 equiv) in THF (1.0 mL) was stirred at 60 °C for 24 h without exclusion of air or moisture. Afterwards, the solvent was removed under reduced pressure, and the resulted mixture was purified by preparative TLC (Eluent: PE/EA = 10/1. R_f = 0.6) to afford the desired diene derivative **23** in 30% (6.9 mg) isolated yield as light yellow oil.

^{1}H NMR (400 MHz, CD_3OD): δ 7.42-7.39 (m, 2H), 7.28-7.22 (m, 3H), 7.10-7.05 (m, 2H), 6.80-6.74 (m, 2H), 5.35 (s, 1H), 5.28 (d, *J* = 2.1 Hz, 1H), 5.22 (s, 1H), 5.05 (d, *J* = 1.8 Hz, 1H); ^{13}C NMR (100 MHz, $CDCl_3$): δ 153.0, 149.8, 145.7, 140.2, 130.3, 129.4, 128.5, 128.4, 127.9, 127.2, 120.8, 120.6, 118.3, 116.0; HRMS (ESI) calcd. for $C_{16}H_{15}O$ ($[M+H]^+$): 223.1118; found: 223.1117.

5. X-Ray Crystallographic Data

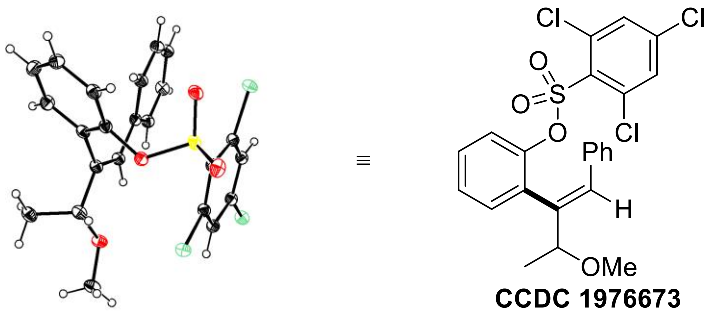

CCDC 1976673

Supplementary Table 4. Crystal data and structure refinement for fanwx_926-1_190927

Identification code	fanwx_926-1_190927
Empirical formula	$C_{23}H_{19}Cl_3O_4S$

Formula weight	497.79
Temperature	100.00(10) K
Wavelength	1.54184 Å
Crystal system	triclinic
Space group	P-1
Unit cell dimensions	a = 7.82530(10) Å α = 94.9400(10)°
	b = 10.8216(2) Å β = 94.9390(10) °
	c = 13.4928(2) Å γ = 104.9380(10)°
Volume	1092.90(3) $Å^3$
Z	2
Density (calculated)	1.513 g cm^{-3}
Absorption coefficient	4.938 mm^{-1}
F(000)	512.0
Crystal size	0.2 × 0.2 × 0.1mm
Theta range for data collection	6.618-153.706 °
Index ranges	-9<=h<=9, -13<=k<=13, -16<=l<=15
Reflections collected	41552
Independent reflections	4509 (R_{int}= 0.0640, R_{sigma} = 0.0261)
Completeness to theta = 76.853 °	98.0%
Absorption correction	multi-scan
Max. and min. transmission	T_{min} = 0.551, T_{max} = 1.000
Refinement method	Full-matrix least-squares on F^2
Data / restraints / parameters	4509/0/282
Goodness-of-fit on F^2	1.090
Final R indices [I>2sigma(I)]	R = 0.0418(4227), wR2 = 0.1191(4509)
Largest diff. peak and hole	0.47 and -0.67 e $Å^{-3}$

6. DFT Studies

Computational details:

Density functional theory (DFT) calculations were carried out using Gaussian 09[S4]. Geometry optimizations and frequency analyses were calculated using SMD[S5] solvation model (solvent = TFE, methanol and 1,4-dioxane) at the level of the B3LYP functional[S6] with Grimme's DFT-D3 method[S7] and a mixed basis set of SDD for Rh and 6-31G(d,p) for other atoms.

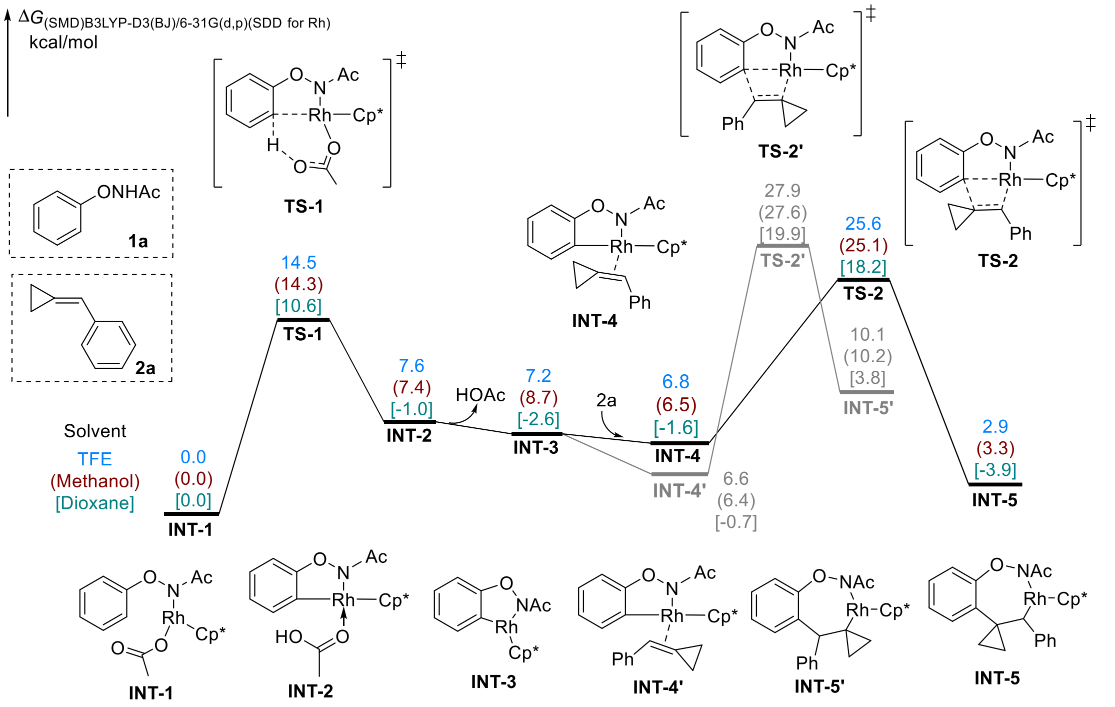

Supplementary Figure 1. Computed pathways for C-H activation and alkene insertion.

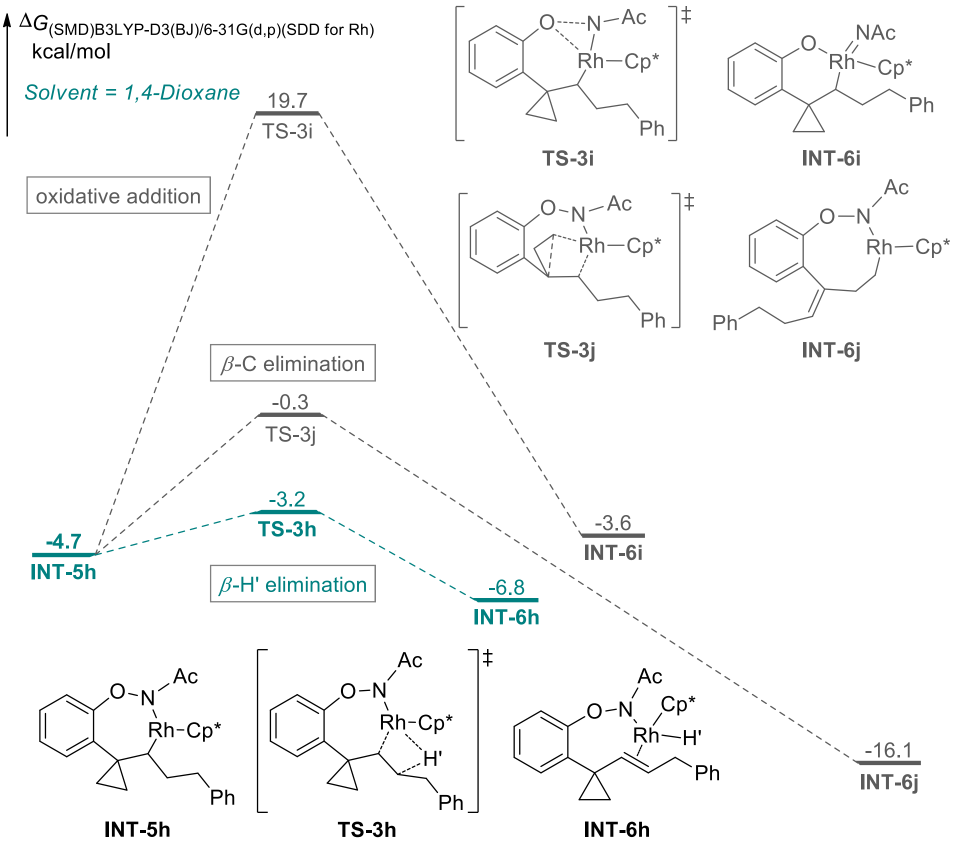

Supplementary Figure 2. Computed Gibbs free energy changes for different reaction

paths from **INT-1h** in dioxane

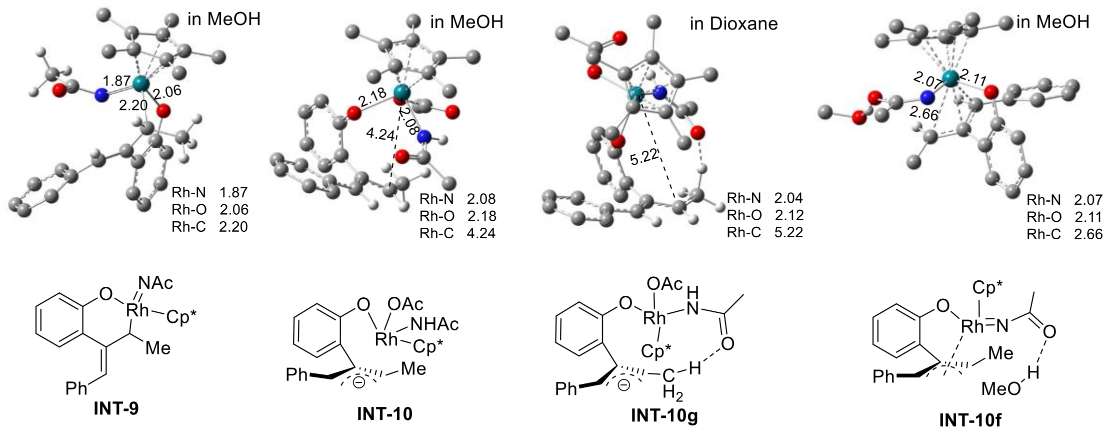

Supplementary Figure 3. Geometry analysis of **INT-9**, **INT-10**, **INT-10f** and **INT-10g**.

7. Supplementary References

[S1] (a) Petrassi, H. M., Sharpless, K. B. & Kelly, J. W. The Copper-Mediated Cross-Coupling of Phenylboronic Acids and *N*-Hydroxyphthalimide at Room Temperature: Synthesis of Aryloxyamines. *Org. Lett.* **3**, 139-142 (2001). (b) Liu, G., Shen, Y., Zhou, Z. & Lu, X. Rhodium(III)-Catalyzed Redox-Neutral Coupling of *N*-Phenoxyacetamides and Alkynes with Tunable Selectivity. *Angew. Chem., Int. Ed.* **52**, 6033-6037 (2013). (c) Li, B., Lan, J., Wu, D. & You, J. Rhodium(III)-Catalyzed *ortho*-Heteroarylation of Phenols through Internal Oxidative C-H Activation: Rapid Screening of Single-Molecular White-Light-Emitting Materials. *Angew. Chem., Int. Ed.* **54**, 14008-14012 (2015).

[S2] Ai, W., Liu, Y., Wang, Q., Lu, Z. & Liu, Q. Cu-Catalyzed Redox-Neutral Ring Cleavage of Cycloketone O-Acyl Oximes: Chemodivergent Access to Distal Oxygenated Nitriles. *Org. Lett.* **20**, 409-412 (2018).

[S3] (a) Wang, X., Lerchen, A., Gensch, T., Knecht, T., Daniliuc, C. G. & Glorius, F. Combination of $Cp^{*}Rh^{III}$Catalyzed C-H Activation and a Wagner-Meerwein-Type Rearrangement. *Angew. Chem., Int. Ed.* **56**, 1381-1384 (2017). (b) Zhou, Z., Bian, M., Zhao, L., Gao, H., Huang, J., Liu, X., Yu, X., Li, X. & Yi, W.

2*H*-Chromene-3-carboxylic Acid Synthesis via Solvent-Controlled and Rhodium(III)-Catalyzed Redox-Neutral C-H Activation/[3+3] Annulation Cascade. *Org. Lett.* **20**, 3892-3896 (2018).

[S4] Frisch, M. J., Trucks, G. W., Schlegel, H. B., Scuseria, G. E., Robb, M. A., Cheeseman, J. R., Scalmani, G., Barone, V., Mennucci, B., Petersson, G. A., Nakatsuji, H., Caricato, M., Li, X., Hratchian, H. P., Izmaylov, A. F., Bloino, J., Zheng, G., Sonnenberg, J. L., Hada, M., Ehara, M., Toyota, K., Fukuda, R., Hasegawa, J., Ishida, M., Nakajima, T., Honda, Y., Kitao, O., Nakai, H., Vreven, T., Montgomery, J. A., Peralta, Jr., J. E., Ogliaro, F., Bearpark, M., Heyd, J. J., Brothers, E., Kudin, K. N., Staroverov, V. N., Keith, T., Kobayashi, R., Normand, J., Raghavachari, K., Rendell, A., Burant, J. C., Iyengar, S. S., Tomasi, J., Cossi, M., Rega, N., Millam, J. M., Klene, M., Knox, J. E., Cross, J. B., Bakken, V.; Adamo, C.; Jaramillo, J.; Gomperts, R.; Stratmann, R. E.; Yazyev, O.; Austin, A. J., Cammi, R., Pomelli, C., Ochterski, J. W., Martin, R. L., Morokuma, K., Zakrzewski, V. G., Voth, G. A., Salvador, P., Dannenberg, J. J., Dapprich, S., Daniels, A. D., Farkas, O., Foresman, J. B., Ortiz, J. V., Cioslowski J. & Fox, D. J. Gaussian 09, revision D. 01, Gaussian, Inc., Wallingford CT, 2013.

[S5] Marenich, A. V., Cramer, C. J. & Truhlar, D. G. Universal Solvation Model Based on Solute Electron Density and on a Continuum Model of the Solvent Defined by the Bulk Dielectric Constant and Atomic Surface Tensions. *J. Phys. Chem. B* **113**, 6378-6396 (2009).

[S6] (a) Lee, C., Yang, W. & Parr, R. G. Development of the Colle-Salvetti Correlation-Energy Formula into A Functional of the Electron-Density. *Phys. Rev. B* **37**, 785-789 (1988). (b) Becke, A. D. Density-Functional Thermochemistry. III. The Role of Exact Exchange. *J. Chem. Phys.* **98**, 5648-5652 (1993).

[S7] Grimme, S., Antony, J., Ehrlich, S. & Krieg, H. A Consistent and Accurate ab initio Parametrization of Density Functional Dispersion Correction (DFT-D) for the 94 Elements H-Pu. *J. Chem. Phys.* **132**, 154104 (2010).
