# Supplementary material for: Chemodivergent assembly of ortho-functionalized phenols with tunable selectivity via rhodium(III)-catalyzed and solvent-controlled C-H activation
Source: Commun Chem. 2021 Jun 3;4:81. doi: 10.1038/s42004-021-00518-x (PMC9814747; doi:10.1038/s42004-021-00518-x)
Supplement: Supplementary file 2 — Description of Additional Supplementary Files [file 42004_2021_518_MOESM2_ESM.pdf]

Description of Additional Supplementary Files

File Name: Supplementary Data 1

Description: crystallographic cif data of compound 7

File Name: Supplementary Data 2

Description: computed energy values and coordinates

File Name: Supplementary Data 3

Description: original ^{1}H, ^{13}C and ^{19}F NMR spectra
