# Supplementary material for: Chemodivergent assembly of ortho-functionalized phenols with tunable selectivity via rhodium(III)-catalyzed and solvent-controlled C-H activation
Source: Commun Chem. 2021 Jun 3;4:81. doi: 10.1038/s42004-021-00518-x (PMC9814747; doi:10.1038/s42004-021-00518-x)
Supplement: Supplementary file 4 — Supplementary Data 2 [file 42004_2021_518_MOESM4_ESM.docx]

**Supplementary Data 2**

**Various energy values for all of the relevant species** [optimized at the level of B3LYP/6-31G(d,p) (SDD for Rh) with SMD atomic radii for experimental solvent TFE, methanol and 1,4-dioxane, respectively]**:**

**CH_3_COOH (in TFE)**

Zero-point correction= 0.061418 (Hartree/Particle)

Thermal correction to Energy= 0.065181

Thermal correction to Enthalpy= 0.066125

Thermal correction to Gibbs Free Energy= 0.035391

Sum of electronic and zero-point Energies= -229.039369

Sum of electronic and thermal Energies= -229.035606

Sum of electronic and thermal Enthalpies= -229.034662

Sum of electronic and thermal Free Energies= -229.065396

**CH_3_COOH (in Methanol)**

Zero-point correction= 0.061453 (Hartree/Particle)

Thermal correction to Energy= 0.065214

Thermal correction to Enthalpy= 0.066158

Thermal correction to Gibbs Free Energy= 0.035427

Sum of electronic and zero-point Energies= -229.042917

Sum of electronic and thermal Energies= -229.039156

Sum of electronic and thermal Enthalpies= -229.038212

Sum of electronic and thermal Free Energies= -229.068943

**CH_3_COOH (in Dioxane)**

Zero-point correction= 0.061983 (Hartree/Particle)

Thermal correction to Energy= 0.066600

Thermal correction to Enthalpy= 0.067544

Thermal correction to Gibbs Free Energy= 0.034190

Sum of electronic and zero-point Energies= -229.037997

Sum of electronic and thermal Energies= -229.033380

Sum of electronic and thermal Enthalpies= -229.032436

Sum of electronic and thermal Free Energies= -229.065789

**MeOH (in Methanol)**

Zero-point correction= 0.051377(Hartree/Particle)

Thermal correction to Energy= 0.054675

Thermal correction to Enthalpy= 0.055619

Thermal correction to Gibbs Free Energy= 0.028647

Sum of electronic and zero-point Energies= -115.681386

Sum of electronic and thermal Energies= -115.678088

Sum of electronic and thermal Enthalpies= -115.677144

Sum of electronic and thermal Free Energies= -115.704117

**AcNH_2_ (in TFE)**

Zero-point correction= 0.073670 (Hartree/Particle)

Thermal correction to Energy= 0.078751

Thermal correction to Enthalpy= 0.079696

Thermal correction to Gibbs Free Energy= 0.045791

Sum of electronic and zero-point Energies= -209.167004

Sum of electronic and thermal Energies= -209.161923

Sum of electronic and thermal Enthalpies= -209.160979

Sum of electronic and thermal Free Energies= -209.194883

**AcNH_2_ (in Methanol)**

Zero-point correction= 0.073717 (Hartree/Particle)

Thermal correction to Energy= 0.078760

Thermal correction to Enthalpy= 0.079704

Thermal correction to Gibbs Free Energy= 0.046104

Sum of electronic and zero-point Energies= -209.169441

Sum of electronic and thermal Energies= -209.164398

Sum of electronic and thermal Enthalpies= -209.163453

Sum of electronic and thermal Free Energies= -209.197054

**AcNH_2_ (in Dioxane)**

Zero-point correction= 0.073759 (Hartree/Particle)

Thermal correction to Energy= 0.078835

Thermal correction to Enthalpy= 0.079779

Thermal correction to Gibbs Free Energy= 0.046439

Sum of electronic and zero-point Energies= -209.159874

Sum of electronic and thermal Energies= -209.154798

Sum of electronic and thermal Enthalpies= -209.153854

Sum of electronic and thermal Free Energies= -209.187194

**2a (in TFE)**

Zero-point correction= 0.167183 (Hartree/Particle)

Thermal correction to Energy= 0.175735

Thermal correction to Enthalpy= 0.176680

Thermal correction to Gibbs Free Energy= 0.133165

Sum of electronic and zero-point Energies= -386.908459

Sum of electronic and thermal Energies= -386.899906

Sum of electronic and thermal Enthalpies= -386.898962

Sum of electronic and thermal Free Energies= -386.942477

**2a (in Methanol)**

Zero-point correction= 0.167166 (Hartree/Particle)

Thermal correction to Energy= 0.175722

Thermal correction to Enthalpy= 0.176666

Thermal correction to Gibbs Free Energy= 0.133135

Sum of electronic and zero-point Energies= -386.909503

Sum of electronic and thermal Energies= -386.900948

Sum of electronic and thermal Enthalpies= -386.900003

Sum of electronic and thermal Free Energies= -386.943534

**2a (in Dioxane)**

Zero-point correction= 0.167336 (Hartree/Particle)

Thermal correction to Energy= 0.175903

Thermal correction to Enthalpy= 0.176847

Thermal correction to Gibbs Free Energy= 0.133210

Sum of electronic and zero-point Energies= -386.906179

Sum of electronic and thermal Energies= -386.897611

Sum of electronic and thermal Enthalpies= -386.896667

Sum of electronic and thermal Free Energies= -386.940304

**2a’ (in Dioxane)**

Zero-point correction= 0.224320 (Hartree/Particle)

Thermal correction to Energy= 0.235633

Thermal correction to Enthalpy= 0.236578

Thermal correction to Gibbs Free Energy= 0.185135

Sum of electronic and zero-point Energies= -465.485721

Sum of electronic and thermal Energies= -465.474407

Sum of electronic and thermal Enthalpies= -465.473463

Sum of electronic and thermal Free Energies= -465.524906

**Cp*Rh(OAc)_2_ (in TFE)**

Zero-point correction= 0.325698 (Hartree/Particle)

Thermal correction to Energy= 0.349615

Thermal correction to Enthalpy= 0.350559

Thermal correction to Gibbs Free Energy= 0.272800

Sum of electronic and zero-point Energies= -957.541586

Sum of electronic and thermal Energies= -957.517669

Sum of electronic and thermal Enthalpies= -957.516724

Sum of electronic and thermal Free Energies= -957.594484

**Cp*Rh(OAc)_2_ (in Methanol)**

Zero-point correction= 0.325575 (Hartree/Particle)

Thermal correction to Energy= 0.349645

Thermal correction to Enthalpy= 0.350589

Thermal correction to Gibbs Free Energy= 0.271497

Sum of electronic and zero-point Energies= -957.548439

Sum of electronic and thermal Energies= -957.524370

Sum of electronic and thermal Enthalpies= -957.523425

Sum of electronic and thermal Free Energies= -957.602517

**Cp*Rh(OAc)_2_ (in Dioxane)**

Zero-point correction= 0.326781 (Hartree/Particle)

Thermal correction to Energy= 0.350797

Thermal correction to Enthalpy= 0.351741

Thermal correction to Gibbs Free Energy= 0.273174

Sum of electronic and zero-point Energies= -957.517705

Sum of electronic and thermal Energies= -957.493689

Sum of electronic and thermal Enthalpies= -957.492744

Sum of electronic and thermal Free Energies= -957.571311

**INT-1 (in TFE)**

Zero-point correction= 0.422781 (Hartree/Particle)

Thermal correction to Energy= 0.452136

Thermal correction to Enthalpy= 0.453080

Thermal correction to Gibbs Free Energy= 0.363047

Sum of electronic and zero-point Energies= -1243.811797

Sum of electronic and thermal Energies= -1243.782442

Sum of electronic and thermal Enthalpies= -1243.781498

Sum of electronic and thermal Free Energies= -1243.871530

**INT-1 (in Methanol)**

Zero-point correction= 0.422675(Hartree/Particle)

Thermal correction to Energy= 0.452106

Thermal correction to Enthalpy= 0.453050

Thermal correction to Gibbs Free Energy= 0.362536

Sum of electronic and zero-point Energies= -1243.817761

Sum of electronic and thermal Energies= -1243.788331

Sum of electronic and thermal Enthalpies= -1243.787386

Sum of electronic and thermal Free Energies= -1243.877900

**INT-1 (in Dioxane)**

Zero-point correction= 0.423987(Hartree/Particle)

Thermal correction to Energy= 0.452263

Thermal correction to Enthalpy= 0.453207

Thermal correction to Gibbs Free Energy= 0.366941

Sum of electronic and zero-point Energies= -1243.789226

Sum of electronic and thermal Energies= -1243.760951

Sum of electronic and thermal Enthalpies= -1243.760007

Sum of electronic and thermal Free Energies= -1243.846272

**INT-2 (in TFE)**

Zero-point correction= 0.422370(Hartree/Particle)

Thermal correction to Energy= 0.451792

Thermal correction to Enthalpy= 0.452736

Thermal correction to Gibbs Free Energy= 0.363310

Sum of electronic and zero-point Energies= -1243.800311

Sum of electronic and thermal Energies= -1243.770890

Sum of electronic and thermal Enthalpies= -1243.769946

Sum of electronic and thermal Free Energies= -1243.859371

**INT-2 (in Methanol)**

Zero-point correction= 0.422160(Hartree/Particle)

Thermal correction to Energy= 0.451689

Thermal correction to Enthalpy= 0.452633

Thermal correction to Gibbs Free Energy= 0.362489

Sum of electronic and zero-point Energies= -1243.806368

Sum of electronic and thermal Energies= -1243.776839

Sum of electronic and thermal Enthalpies= -1243.775894

Sum of electronic and thermal Free Energies= -1243.866039

**INT-2 (in Dioxane)**

Zero-point correction= 0.423265(Hartree/Particle)

Thermal correction to Energy= 0.452935

Thermal correction to Enthalpy= 0.453879

Thermal correction to Gibbs Free Energy= 0.361016

Sum of electronic and zero-point Energies= -1243.785551

Sum of electronic and thermal Energies= -1243.755881

Sum of electronic and thermal Enthalpies= -1243.754937

Sum of electronic and thermal Free Energies= -1243.847801

**INT-3 (in TFE)**

Zero-point correction= 0.359420 (Hartree/Particle)

Thermal correction to Energy= 0.383069

Thermal correction to Enthalpy= 0.384013

Thermal correction to Gibbs Free Energy= 0.305991

Sum of electronic and zero-point Energies= -1014.741311

Sum of electronic and thermal Energies= -1014.717662

Sum of electronic and thermal Enthalpies= -1014.716718

Sum of electronic and thermal Free Energies= -1014.794739

**INT-3 (in Methanol)**

Zero-point correction= 0.359384 (Hartree/Particle)

Thermal correction to Energy= 0.382136

Thermal correction to Enthalpy= 0.383080

Thermal correction to Gibbs Free Energy= 0.309236

Sum of electronic and zero-point Energies= -1014.744994

Sum of electronic and thermal Energies= -1014.722242

Sum of electronic and thermal Enthalpies= -1014.721298

Sum of electronic and thermal Free Energies= -1014.795142

**INT-3 (in Dioxane)**

Zero-point correction= 0.359912(Hartree/Particle)

Thermal correction to Energy= 0.383762

Thermal correction to Enthalpy= 0.384706

Thermal correction to Gibbs Free Energy= 0.305946

Sum of electronic and zero-point Energies= -1014.730709

Sum of electronic and thermal Energies= -1014.706859

Sum of electronic and thermal Enthalpies= -1014.705915

Sum of electronic and thermal Free Energies= -1014.784676

**INT-4 (in TFE)**

Zero-point correction= 0.530071(Hartree/Particle)

Thermal correction to Energy= 0.562717

Thermal correction to Enthalpy= 0.563662

Thermal correction to Gibbs Free Energy= 0.469119

Sum of electronic and zero-point Energies= -1401.676870

Sum of electronic and thermal Energies= -1401.644224

Sum of electronic and thermal Enthalpies= -1401.643280

Sum of electronic and thermal Free Energies= -1401.737822

**INT-4 (in Methanol)**

Zero-point correction= 0.529954(Hartree/Particle)

Thermal correction to Energy= 0.562657

Thermal correction to Enthalpy= 0.563601

Thermal correction to Gibbs Free Energy= 0.468630

Sum of electronic and zero-point Energies= -1401.680800

Sum of electronic and thermal Energies= -1401.648097

Sum of electronic and thermal Enthalpies= -1401.647153

Sum of electronic and thermal Free Energies= -1401.742124

**INT-4 (in Dioxane)**

Zero-point correction= 0.530302(Hartree/Particle)

Thermal correction to Energy= 0.563233

Thermal correction to Enthalpy= 0.564177

Thermal correction to Gibbs Free Energy= 0.468328

Sum of electronic and zero-point Energies= -1401.661420

Sum of electronic and thermal Energies= -1401.628489

Sum of electronic and thermal Enthalpies= -1401.627545

Sum of electronic and thermal Free Energies= -1401.723395

**INT-4’ (in TFE)**

Zero-point correction= 0.529471(Hartree/Particle)

Thermal correction to Energy= 0.562383

Thermal correction to Enthalpy= 0.563327

Thermal correction to Gibbs Free Energy= 0.467415

Sum of electronic and zero-point Energies= -1401.675986

Sum of electronic and thermal Energies= -1401.643074

Sum of electronic and thermal Enthalpies= -1401.642130

Sum of electronic and thermal Free Energies= -1401.738042

**INT-4’ (in Methanol)**

Zero-point correction= 0.529401(Hartree/Particle)

Thermal correction to Energy= 0.562368

Thermal correction to Enthalpy= 0.563313

Thermal correction to Gibbs Free Energy= 0.467026

Sum of electronic and zero-point Energies= -1401.679928

Sum of electronic and thermal Energies= -1401.646961

Sum of electronic and thermal Enthalpies= -1401.646017

Sum of electronic and thermal Free Energies= -1401.742303

**INT-4’ (in Dioxane)**

Zero-point correction= 0.529623(Hartree/Particle)

Thermal correction to Energy= 0.561962

Thermal correction to Enthalpy= 0.562906

Thermal correction to Gibbs Free Energy= 0.469129

Sum of electronic and zero-point Energies= -1401.661403

Sum of electronic and thermal Energies= -1401.629063

Sum of electronic and thermal Enthalpies= -1401.628119

Sum of electronic and thermal Free Energies= -1401.721897

**INT-5 (in TFE)**

Zero-point correction= 0.530994 (Hartree/Particle)

Thermal correction to Energy= 0.563395

Thermal correction to Enthalpy= 0.564339

Thermal correction to Gibbs Free Energy= 0.468799

Sum of electronic and zero-point Energies= -1401.681728

Sum of electronic and thermal Energies= -1401.649327

Sum of electronic and thermal Enthalpies= -1401.648382

Sum of electronic and thermal Free Energies= -1401.743923

**INT-5 (in Methanol)**

Zero-point correction= 0.530941 (Hartree/Particle)

Thermal correction to Energy= 0.563314

Thermal correction to Enthalpy= 0.564258

Thermal correction to Gibbs Free Energy= 0.469415

Sum of electronic and zero-point Energies= -1401.685678

Sum of electronic and thermal Energies= -1401.653305

Sum of electronic and thermal Enthalpies= -1401.652361

Sum of electronic and thermal Free Energies= -1401.747205

**INT-5 (in Dioxane)**

Zero-point correction= 0.531725 (Hartree/Particle)

Thermal correction to Energy= 0.564099

Thermal correction to Enthalpy= 0.565043

Thermal correction to Gibbs Free Energy= 0.470570

Sum of electronic and zero-point Energies= -1401.665846

Sum of electronic and thermal Energies= -1401.633472

Sum of electronic and thermal Enthalpies= -1401.632527

Sum of electronic and thermal Free Energies= -1401.727000

**INT-5’ (in TFE)**

Zero-point correction= 0.530416(Hartree/Particle)

Thermal correction to Energy= 0.563112

Thermal correction to Enthalpy= 0.564056

Thermal correction to Gibbs Free Energy= 0.467109

Sum of electronic and zero-point Energies= -1401.669205

Sum of electronic and thermal Energies= -1401.636508

Sum of electronic and thermal Enthalpies= -1401.635564

Sum of electronic and thermal Free Energies= -1401.732511

**INT-5’ (in Methanol)**

Zero-point correction= 0.530305(Hartree/Particle)

Thermal correction to Energy= 0.563033

Thermal correction to Enthalpy= 0.563977

Thermal correction to Gibbs Free Energy= 0.467271

Sum of electronic and zero-point Energies= -1401.673197

Sum of electronic and thermal Energies= -1401.640469

Sum of electronic and thermal Enthalpies= -1401.639525

Sum of electronic and thermal Free Energies= -1401.736231

**INT-5’ (in Dioxane)**

Zero-point correction= 0.531477 (Hartree/Particle)

Thermal correction to Energy= 0.564048

Thermal correction to Enthalpy= 0.564992

Thermal correction to Gibbs Free Energy= 0.469059

Sum of electronic and zero-point Energies= -1401.652350

Sum of electronic and thermal Energies= -1401.619779

Sum of electronic and thermal Enthalpies= -1401.618835

Sum of electronic and thermal Free Energies= -1401.714768

**INT-5h (in Dioxane)**

Zero-point correction= 0.588162 (Hartree/Particle)

Thermal correction to Energy= 0.622648

Thermal correction to Enthalpy= 0.623592

Thermal correction to Gibbs Free Energy= 0.523780

Sum of electronic and zero-point Energies= -1480.248472

Sum of electronic and thermal Energies= -1480.213986

Sum of electronic and thermal Enthalpies= -1480.213042

Sum of electronic and thermal Free Energies= -1480.312854

**INT-6 (in TFE)**

Zero-point correction= 0.531059 (Hartree/Particle)

Thermal correction to Energy= 0.563523

Thermal correction to Enthalpy= 0.564467

Thermal correction to Gibbs Free Energy= 0.468359

Sum of electronic and zero-point Energies= -1401.696420

Sum of electronic and thermal Energies= -1401.663956

Sum of electronic and thermal Enthalpies= -1401.663012

Sum of electronic and thermal Free Energies= -1401.759120

**INT-6 (in Methanol)**

Zero-point correction= 0.530975 (Hartree/Particle)

Thermal correction to Energy= 0.563479

Thermal correction to Enthalpy= 0.564424

Thermal correction to Gibbs Free Energy= 0.467932

Sum of electronic and zero-point Energies= -1401.700433

Sum of electronic and thermal Energies= -1401.667928

Sum of electronic and thermal Enthalpies= -1401.666984

Sum of electronic and thermal Free Energies= -1401.763476

**INT-6 (in Dioxane)**

Zero-point correction= 0.531818 (Hartree/Particle)

Thermal correction to Energy= 0.564256

Thermal correction to Enthalpy= 0.565200

Thermal correction to Gibbs Free Energy= 0.469866

Sum of electronic and zero-point Energies= -1401.682042

Sum of electronic and thermal Energies= -1401.649604

Sum of electronic and thermal Enthalpies= -1401.648660

Sum of electronic and thermal Free Energies= -1401.743994

**INT-6a (in TFE)**

Zero-point correction= 0.530344 (Hartree/Particle)

Thermal correction to Energy= 0.562923

Thermal correction to Enthalpy= 0.563867

Thermal correction to Gibbs Free Energy= 0.468047

Sum of electronic and zero-point Energies= -1401.681315

Sum of electronic and thermal Energies= -1401.648736

Sum of electronic and thermal Enthalpies= -1401.647792

Sum of electronic and thermal Free Energies= -1401.743612

**INT-6a (in Methanol)**

Zero-point correction= 0.530267 (Hartree/Particle)

Thermal correction to Energy= 0.562889

Thermal correction to Enthalpy= 0.563833

Thermal correction to Gibbs Free Energy= 0.468049

Sum of electronic and zero-point Energies= -1401.685980

Sum of electronic and thermal Energies= -1401.653358

Sum of electronic and thermal Enthalpies= -1401.652414

Sum of electronic and thermal Free Energies= -1401.748197

**INT-6a (in Dioxane)**

Zero-point correction= 0.529521 (Hartree/Particle)

Thermal correction to Energy= 0.562797

Thermal correction to Enthalpy= 0.563741

Thermal correction to Gibbs Free Energy= 0.465527

Sum of electronic and zero-point Energies= -1401.658930

Sum of electronic and thermal Energies= -1401.625654

Sum of electronic and thermal Enthalpies= -1401.624710

Sum of electronic and thermal Free Energies= -1401.722923

**INT-6h (in Dioxane)**

Zero-point correction= 0.586992 (Hartree/Particle)

Thermal correction to Energy= 0.621436

Thermal correction to Enthalpy= 0.622380

Thermal correction to Gibbs Free Energy= 0.523487

Sum of electronic and zero-point Energies= -1480.252692

Sum of electronic and thermal Energies= -1480.218248

Sum of electronic and thermal Enthalpies= -1480.217304

Sum of electronic and thermal Free Energies= -1480.316197

**INT-6i (in Dioxane)**

Zero-point correction= 0.587435 (Hartree/Particle)

Thermal correction to Energy= 0.622799

Thermal correction to Enthalpy= 0.623743

Thermal correction to Gibbs Free Energy= 0.518471

Sum of electronic and zero-point Energies= -1480.242124

Sum of electronic and thermal Energies= -1480.206760

Sum of electronic and thermal Enthalpies= -1480.205816

Sum of electronic and thermal Free Energies= -1480.311088

**INT-6j (in Dioxane)**

Zero-point correction= 0.589236 (Hartree/Particle)

Thermal correction to Energy= 0.624216

Thermal correction to Enthalpy= 0.625160

Thermal correction to Gibbs Free Energy= 0.521987

Sum of electronic and zero-point Energies= -1480.263849

Sum of electronic and thermal Energies= -1480.228869

Sum of electronic and thermal Enthalpies= -1480.227925

Sum of electronic and thermal Free Energies= -1480.331098

**INT-7 (in TFE)**

Zero-point correction= 0.528135 (Hartree/Particle)

Thermal correction to Energy= 0.560714

Thermal correction to Enthalpy= 0.561658

Thermal correction to Gibbs Free Energy= 0.463629

Sum of electronic and zero-point Energies= -1401.697716

Sum of electronic and thermal Energies= -1401.665137

Sum of electronic and thermal Enthalpies= -1401.664193

Sum of electronic and thermal Free Energies= -1401.762222

**INT-7 (in Methanol)**

Zero-point correction= 0.528020 (Hartree/Particle)

Thermal correction to Energy= 0.560674

Thermal correction to Enthalpy= 0.561618

Thermal correction to Gibbs Free Energy= 0.463354

Sum of electronic and zero-point Energies= -1401.701816

Sum of electronic and thermal Energies= -1401.669162

Sum of electronic and thermal Enthalpies= -1401.668218

Sum of electronic and thermal Free Energies= -1401.766482

**INT-7 (in Dioxane)**

Zero-point correction= 0.529666 (Hartree/Particle)

Thermal correction to Energy= 0.562079

Thermal correction to Enthalpy= 0.563023

Thermal correction to Gibbs Free Energy= 0.466363

Sum of electronic and zero-point Energies= -1401.681271

Sum of electronic and thermal Energies= -1401.648858

Sum of electronic and thermal Enthalpies= -1401.647913

Sum of electronic and thermal Free Energies= -1401.744573

**INT-8 (in TFE)**

Zero-point correction= 0.530233 (Hartree/Particle)

Thermal correction to Energy= 0.563157

Thermal correction to Enthalpy= 0.564101

Thermal correction to Gibbs Free Energy= 0.467155

Sum of electronic and zero-point Energies= -1401.699066

Sum of electronic and thermal Energies= -1401.666142

Sum of electronic and thermal Enthalpies= -1401.665198

Sum of electronic and thermal Free Energies= -1401.762144

**INT-8 (in Methanol)**

Zero-point correction= 0.530202 (Hartree/Particle)

Thermal correction to Energy= 0.563126

Thermal correction to Enthalpy= 0.564070

Thermal correction to Gibbs Free Energy= 0.466984

Sum of electronic and zero-point Energies= -1401.703439

Sum of electronic and thermal Energies= -1401.670515

Sum of electronic and thermal Enthalpies= -1401.669571

Sum of electronic and thermal Free Energies= -1401.766657

**INT-8 (in Dioxane)**

Zero-point correction= 0.530682 (Hartree/Particle)

Thermal correction to Energy= 0.563772

Thermal correction to Enthalpy= 0.564716

Thermal correction to Gibbs Free Energy= 0.466990

Sum of electronic and zero-point Energies= -1401.679637

Sum of electronic and thermal Energies= -1401.646546

Sum of electronic and thermal Enthalpies= -1401.645602

Sum of electronic and thermal Free Energies= -1401.743328

**INT-8d (in TFE)**

Zero-point correction= 0.530977 (Hartree/Particle)

Thermal correction to Energy= 0.563593

Thermal correction to Enthalpy= 0.564538

Thermal correction to Gibbs Free Energy= 0.467319

Sum of electronic and zero-point Energies= -1401.677562

Sum of electronic and thermal Energies= -1401.644945

Sum of electronic and thermal Enthalpies= -1401.644001

Sum of electronic and thermal Free Energies= -1401.741219

**INT-8d (in Methanol)**

Zero-point correction= 0.531087 (Hartree/Particle)

Thermal correction to Energy= 0.563620

Thermal correction to Enthalpy= 0.564564

Thermal correction to Gibbs Free Energy= 0.467857

Sum of electronic and zero-point Energies= -1401.682050

Sum of electronic and thermal Energies= -1401.649518

Sum of electronic and thermal Enthalpies= -1401.648574

Sum of electronic and thermal Free Energies= -1401.745281

**INT-8d (in Dioxane)**

Zero-point correction= 0.531100 (Hartree/Particle)

Thermal correction to Energy= 0.564013

Thermal correction to Enthalpy= 0.564957

Thermal correction to Gibbs Free Energy= 0.466810

Sum of electronic and zero-point Energies= -1401.668352

Sum of electronic and thermal Energies= -1401.635439

Sum of electronic and thermal Enthalpies= -1401.634495

Sum of electronic and thermal Free Energies= -1401.732642

**INT-9 (in TFE)**

Zero-point correction= 0.528789 (Hartree/Particle)

Thermal correction to Energy= 0.562068

Thermal correction to Enthalpy= 0.563013

Thermal correction to Gibbs Free Energy= 0.464871

Sum of electronic and zero-point Energies= -1401.699468

Sum of electronic and thermal Energies= -1401.666189

Sum of electronic and thermal Enthalpies= -1401.665245

Sum of electronic and thermal Free Energies= -1401.763386

**INT-9 (in Methanol)**

Zero-point correction= 0.528737 (Hartree/Particle)

Thermal correction to Energy= 0.562058

Thermal correction to Enthalpy= 0.563002

Thermal correction to Gibbs Free Energy= 0.464958

Sum of electronic and zero-point Energies= -1401.704187

Sum of electronic and thermal Energies= -1401.670865

Sum of electronic and thermal Enthalpies= -1401.669921

Sum of electronic and thermal Free Energies= -1401.767966

**INT-9 (in Dioxane)**

Zero-point correction= 0.529158 (Hartree/Particle)

Thermal correction to Energy= 0.562681

Thermal correction to Enthalpy= 0.563625

Thermal correction to Gibbs Free Energy= 0.463965

Sum of electronic and zero-point Energies= -1401.677833

Sum of electronic and thermal Energies= -1401.644310

Sum of electronic and thermal Enthalpies= -1401.643366

Sum of electronic and thermal Free Energies= -1401.743026

**INT-10 (in TFE)**

Zero-point correction= 0.592354 (Hartree/Particle)

Thermal correction to Energy= 0.632006

Thermal correction to Enthalpy= 0.632950

Thermal correction to Gibbs Free Energy= 0.518336

Sum of electronic and zero-point Energies= -1630.815711

Sum of electronic and thermal Energies= -1630.776059

Sum of electronic and thermal Enthalpies= -1630.775114

Sum of electronic and thermal Free Energies= -1630.889729

**INT-10 (in Methanol)**

Zero-point correction= 0.591839 (Hartree/Particle)

Thermal correction to Energy= 0.631753

Thermal correction to Enthalpy= 0.632697

Thermal correction to Gibbs Free Energy= 0.518962

Sum of electronic and zero-point Energies= -1630.822056

Sum of electronic and thermal Energies= -1630.782142

Sum of electronic and thermal Enthalpies= -1630.781198

Sum of electronic and thermal Free Energies= -1630.894933

**INT-10g (in Dioxane)**

Zero-point correction= 0.592806 (Hartree/Particle)

Thermal correction to Energy= 0.632264

Thermal correction to Enthalpy= 0.633208

Thermal correction to Gibbs Free Energy= 0.521273

Sum of electronic and zero-point Energies= -1630.775571

Sum of electronic and thermal Energies= -1630.736113

Sum of electronic and thermal Enthalpies= -1630.735169

Sum of electronic and thermal Free Energies= -1630.847104

**INT-10f (in Methanol)**

Zero-point correction= 0.582368 (Hartree/Particle)

Thermal correction to Energy= 0.620157

Thermal correction to Enthalpy= 0.621101

Thermal correction to Gibbs Free Energy= 0.513668

Sum of electronic and zero-point Energies= -1517.409759

Sum of electronic and thermal Energies= -1517.371971

Sum of electronic and thermal Enthalpies= -1517.371026

Sum of electronic and thermal Free Energies= -1517.478459

**TS-1(in TFE)**

Zero-point correction= 0.416873(Hartree/Particle)

Thermal correction to Energy= 0.446003

Thermal correction to Enthalpy= 0.446947

Thermal correction to Gibbs Free Energy= 0.357583

Sum of electronic and zero-point Energies= -1243.789107

Sum of electronic and thermal Energies= -1243.759977

Sum of electronic and thermal Enthalpies= -1243.759033

Sum of electronic and thermal Free Energies= -1243.848397

**TS-1(in Methanol)**

Zero-point correction= 0.416660(Hartree/Particle)

Thermal correction to Energy= 0.445902

Thermal correction to Enthalpy= 0.446847

Thermal correction to Gibbs Free Energy= 0.356918

Sum of electronic and zero-point Energies= -1243.795293

Sum of electronic and thermal Energies= -1243.766051

Sum of electronic and thermal Enthalpies= -1243.765107

Sum of electronic and thermal Free Energies= -1243.855035

**TS-1(in Dioxane)**

Zero-point correction= 0.418852(Hartree/Particle)

Thermal correction to Energy= 0.447464

Thermal correction to Enthalpy= 0.448408

Thermal correction to Gibbs Free Energy= 0.361714

Sum of electronic and zero-point Energies= -1243.772166

Sum of electronic and thermal Energies= -1243.743554

Sum of electronic and thermal Enthalpies= -1243.742610

Sum of electronic and thermal Free Energies= -1243.829303

**TS-2(in TFE)**

Zero-point correction= 0.529272 (Hartree/Particle)

Thermal correction to Energy= 0.561153

Thermal correction to Enthalpy= 0.562098

Thermal correction to Gibbs Free Energy= 0.469152

Sum of electronic and zero-point Energies= -1401.647623

Sum of electronic and thermal Energies= -1401.615742

Sum of electronic and thermal Enthalpies= -1401.614798

Sum of electronic and thermal Free Energies= -1401.707744

**TS-2(in Methanol)**

Zero-point correction= 0.528992(Hartree/Particle)

Thermal correction to Energy= 0.561029

Thermal correction to Enthalpy= 0.561974

Thermal correction to Gibbs Free Energy= 0.468308

Sum of electronic and zero-point Energies= -1401.651863

Sum of electronic and thermal Energies= -1401.619825

Sum of electronic and thermal Enthalpies= -1401.618881

Sum of electronic and thermal Free Energies= -1401.712546

**TS-2(in Dioxane)**

Zero-point correction= 0.529991(Hartree/Particle)

Thermal correction to Energy= 0.562000

Thermal correction to Enthalpy= 0.562944

Thermal correction to Gibbs Free Energy= 0.469295

Sum of electronic and zero-point Energies= -1401.631035

Sum of electronic and thermal Energies= -1401.599026

Sum of electronic and thermal Enthalpies= -1401.598082

Sum of electronic and thermal Free Energies= -1401.691731

**TS-2’(in TFE)**

Zero-point correction= 0.529059(Hartree/Particle)

Thermal correction to Energy= 0.561209

Thermal correction to Enthalpy= 0.562153

Thermal correction to Gibbs Free Energy= 0.468192

Sum of electronic and zero-point Energies= -1401.643322

Sum of electronic and thermal Energies= -1401.611172

Sum of electronic and thermal Enthalpies= -1401.610228

Sum of electronic and thermal Free Energies= -1401.704189

**TS-2’(in Methanol)**

Zero-point correction= 0.528935(Hartree/Particle)

Thermal correction to Energy= 0.561155

Thermal correction to Enthalpy= 0.562100

Thermal correction to Gibbs Free Energy= 0.467827

Sum of electronic and zero-point Energies= -1401.647468

Sum of electronic and thermal Energies= -1401.615248

Sum of electronic and thermal Enthalpies= -1401.614303

Sum of electronic and thermal Free Energies= -1401.708576

**TS-2’ (in Dioxane)**

Zero-point correction= 0.529691(Hartree/Particle)

Thermal correction to Energy= 0.561866

Thermal correction to Enthalpy= 0.562810

Thermal correction to Gibbs Free Energy= 0.469030

Sum of electronic and zero-point Energies= -1401.628358

Sum of electronic and thermal Energies= -1401.596183

Sum of electronic and thermal Enthalpies= -1401.595239

Sum of electronic and thermal Free Energies= -1401.689019

**TS-3 (in TFE)**

Zero-point correction= 0.530979 (Hartree/Particle)

Thermal correction to Energy= 0.562431

Thermal correction to Enthalpy= 0.563375

Thermal correction to Gibbs Free Energy= 0.471219

Sum of electronic and zero-point Energies= -1401.671136

Sum of electronic and thermal Energies= -1401.639684

Sum of electronic and thermal Enthalpies= -1401.638740

Sum of electronic and thermal Free Energies= -1401.730897

**TS-3 (in Methanol)**

Zero-point correction= 0.530966 (Hartree/Particle)

Thermal correction to Energy= 0.562439

Thermal correction to Enthalpy= 0.563383

Thermal correction to Gibbs Free Energy= 0.470905

Sum of electronic and zero-point Energies= -1401.675282

Sum of electronic and thermal Energies= -1401.643809

Sum of electronic and thermal Enthalpies= -1401.642865

Sum of electronic and thermal Free Energies= -1401.735343

**TS-3 (in Dioxane)**

Zero-point correction= 0.531393 (Hartree/Particle)

Thermal correction to Energy= 0.562980

Thermal correction to Enthalpy= 0.563924

Thermal correction to Gibbs Free Energy= 0.470978

Sum of electronic and zero-point Energies= -1401.653502

Sum of electronic and thermal Energies= -1401.621915

Sum of electronic and thermal Enthalpies= -1401.620971

Sum of electronic and thermal Free Energies= -1401.713917

**TS-3a (in TFE)**

Zero-point correction= 0.528152 (Hartree/Particle)

Thermal correction to Energy= 0.560422

Thermal correction to Enthalpy= 0.561366

Thermal correction to Gibbs Free Energy= 0.466653

Sum of electronic and zero-point Energies= -1401.645881

Sum of electronic and thermal Energies= -1401.613611

Sum of electronic and thermal Enthalpies= -1401.612667

Sum of electronic and thermal Free Energies= -1401.707379

**TS-3a (in Methanol)**

Zero-point correction= 0.528035 (Hartree/Particle)

Thermal correction to Energy= 0.560360

Thermal correction to Enthalpy= 0.561304

Thermal correction to Gibbs Free Energy= 0.466309

Sum of electronic and zero-point Energies= -1401.650205

Sum of electronic and thermal Energies= -1401.617880

Sum of electronic and thermal Enthalpies= -1401.616936

Sum of electronic and thermal Free Energies= -1401.711932

**TS-3a (in Dioxane)**

Zero-point correction= 0.528675 (Hartree/Particle)

Thermal correction to Energy= 0.561233

Thermal correction to Enthalpy= 0.562177

Thermal correction to Gibbs Free Energy= 0.464956

Sum of electronic and zero-point Energies= -1401.633318

Sum of electronic and thermal Energies= -1401.600761

Sum of electronic and thermal Enthalpies= -1401.599816

Sum of electronic and thermal Free Energies= -1401.697038

**TS-3h (in Dioxane)**

Zero-point correction= 0.585392 (Hartree/Particle)

Thermal correction to Energy= 0.619635

Thermal correction to Enthalpy= 0.620579

Thermal correction to Gibbs Free Energy= 0.521648

Sum of electronic and zero-point Energies= -1480.246809

Sum of electronic and thermal Energies= -1480.212567

Sum of electronic and thermal Enthalpies= -1480.211623

Sum of electronic and thermal Free Energies= -1480.310553

**TS-3i (in Dioxane)**

Zero-point correction= 0.585941 (Hartree/Particle)

Thermal correction to Energy= 0.620984

Thermal correction to Enthalpy= 0.621928

Thermal correction to Gibbs Free Energy= 0.517452

Sum of electronic and zero-point Energies= -1480.205472

Sum of electronic and thermal Energies= -1480.170428

Sum of electronic and thermal Enthalpies= -1480.169484

Sum of electronic and thermal Free Energies= -1480.273960

**TS-3j (in Dioxane)**

Zero-point correction= 0.588384 (Hartree/Particle)

Thermal correction to Energy= 0.622661

Thermal correction to Enthalpy= 0.623605

Thermal correction to Gibbs Free Energy= 0.524028

Sum of electronic and zero-point Energies= -1480.241532

Sum of electronic and thermal Energies= -1480.207255

Sum of electronic and thermal Enthalpies= -1480.206311

Sum of electronic and thermal Free Energies= -1480.305888

**TS-4 (in TFE)**

Zero-point correction= 0.527106 (Hartree/Particle)

Thermal correction to Energy= 0.559252

Thermal correction to Enthalpy= 0.560196

Thermal correction to Gibbs Free Energy= 0.462956

Sum of electronic and zero-point Energies= -1401.681159

Sum of electronic and thermal Energies= -1401.649013

Sum of electronic and thermal Enthalpies= -1401.648069

Sum of electronic and thermal Free Energies= -1401.745309

**TS-4 (in Methanol)**

Zero-point correction= 0.527072 (Hartree/Particle)

Thermal correction to Energy= 0.559229

Thermal correction to Enthalpy= 0.560173

Thermal correction to Gibbs Free Energy= 0.462133

Sum of electronic and zero-point Energies= -1401.685732

Sum of electronic and thermal Energies= -1401.653575

Sum of electronic and thermal Enthalpies= -1401.652631

Sum of electronic and thermal Free Energies= -1401.750671

**TS-4 (in Dioxane)**

Zero-point correction= 0.527355(Hartree/Particle)

Thermal correction to Energy= 0.558918

Thermal correction to Enthalpy= 0.559862

Thermal correction to Gibbs Free Energy= 0.465516

Sum of electronic and zero-point Energies= -1401.664205

Sum of electronic and thermal Energies= -1401.632643

Sum of electronic and thermal Enthalpies= -1401.631699

Sum of electronic and thermal Free Energies= -1401.726044

**TS-4a (in TFE)**

Zero-point correction= 0.528465 (Hartree/Particle)

Thermal correction to Energy= 0.560703

Thermal correction to Enthalpy= 0.561648

Thermal correction to Gibbs Free Energy= 0.467043

Sum of electronic and zero-point Energies= -1401.639207

Sum of electronic and thermal Energies= -1401.606969

Sum of electronic and thermal Enthalpies= -1401.606025

Sum of electronic and thermal Free Energies= -1401.700629

**TS-4a (in Methanol)**

Zero-point correction= 0.528585 (Hartree/Particle)

Thermal correction to Energy= 0.560755

Thermal correction to Enthalpy= 0.561700

Thermal correction to Gibbs Free Energy= 0.467905

Sum of electronic and zero-point Energies= -1401.644405

Sum of electronic and thermal Energies= -1401.612234

Sum of electronic and thermal Enthalpies= -1401.611289

Sum of electronic and thermal Free Energies= -1401.705084

**TS-4a (in Dioxane)**

Zero-point correction= 0.528989 (Hartree/Particle)

Thermal correction to Energy= 0.561186

Thermal correction to Enthalpy= 0.562131

Thermal correction to Gibbs Free Energy= 0.468236

Sum of electronic and zero-point Energies= -1401.603895

Sum of electronic and thermal Energies= -1401.571698

Sum of electronic and thermal Enthalpies= -1401.570753

Sum of electronic and thermal Free Energies= -1401.664648

**TS-4b (in TFE)**

Zero-point correction= 0.527119 (Hartree/Particle)

Thermal correction to Energy= 0.559910

Thermal correction to Enthalpy= 0.560854

Thermal correction to Gibbs Free Energy= 0.462573

Sum of electronic and zero-point Energies= -1401.649449

Sum of electronic and thermal Energies= -1401.616658

Sum of electronic and thermal Enthalpies= -1401.615714

Sum of electronic and thermal Free Energies= -1401.713995

**TS-4b (in Methanol)**

Zero-point correction= 0.526014(Hartree/Particle)

Thermal correction to Energy= 0.558485

Thermal correction to Enthalpy= 0.559429

Thermal correction to Gibbs Free Energy= 0.462223

Sum of electronic and zero-point Energies= -1401.654948

Sum of electronic and thermal Energies= -1401.622477

Sum of electronic and thermal Enthalpies= -1401.621533

Sum of electronic and thermal Free Energies= -1401.718739

**TS-4b (in Dioxane)**

Zero-point correction= 0.527821 (Hartree/Particle)

Thermal correction to Energy= 0.560796

Thermal correction to Enthalpy= 0.561740

Thermal correction to Gibbs Free Energy= 0.462884

Sum of electronic and zero-point Energies= -1401.634733

Sum of electronic and thermal Energies= -1401.601759

Sum of electronic and thermal Enthalpies= -1401.600815

Sum of electronic and thermal Free Energies= -1401.699670

**TS-5 (in TFE)**

Zero-point correction= 0.527105 (Hartree/Particle)

Thermal correction to Energy= 0.559203

Thermal correction to Enthalpy= 0.560147

Thermal correction to Gibbs Free Energy= 0.464557

Sum of electronic and zero-point Energies= -1401.687881

Sum of electronic and thermal Energies= -1401.655783

Sum of electronic and thermal Enthalpies= -1401.654839

Sum of electronic and thermal Free Energies= -1401.750429

**TS-5 (in Methanol)**

Zero-point correction= 0.526929 (Hartree/Particle)

Thermal correction to Energy= 0.559108

Thermal correction to Enthalpy= 0.560052

Thermal correction to Gibbs Free Energy= 0.463949

Sum of electronic and zero-point Energies= -1401.692154

Sum of electronic and thermal Energies= -1401.659975

Sum of electronic and thermal Enthalpies= -1401.659031

Sum of electronic and thermal Free Energies= -1401.755135

**TS-5 (in Dioxane)**

Zero-point correction= 0.527735 (Hartree/Particle)

Thermal correction to Energy= 0.559900

Thermal correction to Enthalpy= 0.560844

Thermal correction to Gibbs Free Energy= 0.464351

Sum of electronic and zero-point Energies= -1401.671617

Sum of electronic and thermal Energies= -1401.639453

Sum of electronic and thermal Enthalpies= -1401.638509

Sum of electronic and thermal Free Energies= -1401.735002

**TS-5c (in TFE)**

Zero-point correction= 0.524356 (Hartree/Particle)

Thermal correction to Energy= 0.557517

Thermal correction to Enthalpy= 0.558462

Thermal correction to Gibbs Free Energy= 0.460138

Sum of electronic and zero-point Energies= -1401.657951

Sum of electronic and thermal Energies= -1401.624790

Sum of electronic and thermal Enthalpies= -1401.623845

Sum of electronic and thermal Free Energies= -1401.722169

**TS-5c (in Methanol)**

Zero-point correction= 0.524594 (Hartree/Particle)

Thermal correction to Energy= 0.557652

Thermal correction to Enthalpy= 0.558596

Thermal correction to Gibbs Free Energy= 0.461002

Sum of electronic and zero-point Energies= -1401.663015

Sum of electronic and thermal Energies= -1401.629957

Sum of electronic and thermal Enthalpies= -1401.629013

Sum of electronic and thermal Free Energies= -1401.726607

**TS-5d (in TFE)**

Zero-point correction= 0.525648 (Hartree/Particle)

Thermal correction to Energy= 0.558028

Thermal correction to Enthalpy= 0.558972

Thermal correction to Gibbs Free Energy= 0.462747

Sum of electronic and zero-point Energies= -1401.660031

Sum of electronic and thermal Energies= -1401.627651

Sum of electronic and thermal Enthalpies= -1401.626706

Sum of electronic and thermal Free Energies= -1401.722931

**TS-5d (in Methanol)**

Zero-point correction= 0.525276 (Hartree/Particle)

Thermal correction to Energy= 0.557861

Thermal correction to Enthalpy= 0.558805

Thermal correction to Gibbs Free Energy= 0.461668

Sum of electronic and zero-point Energies= -1401.664475

Sum of electronic and thermal Energies= -1401.631890

Sum of electronic and thermal Enthalpies= -1401.630946

Sum of electronic and thermal Free Energies= -1401.728083

**TS-5d (in Dioxane)**

Zero-point correction= 0.526586 (Hartree/Particle)

Thermal correction to Energy= 0.559029

Thermal correction to Enthalpy= 0.559973

Thermal correction to Gibbs Free Energy= 0.462524

Sum of electronic and zero-point Energies= -1401.648774

Sum of electronic and thermal Energies= -1401.616331

Sum of electronic and thermal Enthalpies= -1401.615387

Sum of electronic and thermal Free Energies= -1401.712836

**TS-6 (in TFE)**

Zero-point correction= 0.527352 (Hartree/Particle)

Thermal correction to Energy= 0.560300

Thermal correction to Enthalpy= 0.561244

Thermal correction to Gibbs Free Energy= 0.463710

Sum of electronic and zero-point Energies= -1401.665015

Sum of electronic and thermal Energies= -1401.632067

Sum of electronic and thermal Enthalpies= -1401.631123

Sum of electronic and thermal Free Energies= -1401.728656

**TS-6 (in Methanol)**

Zero-point correction= 0.527358 (Hartree/Particle)

Thermal correction to Energy= 0.560311

Thermal correction to Enthalpy= 0.561255

Thermal correction to Gibbs Free Energy= 0.463496

Sum of electronic and zero-point Energies= -1401.669710

Sum of electronic and thermal Energies= -1401.636756

Sum of electronic and thermal Enthalpies= -1401.635812

Sum of electronic and thermal Free Energies= -1401.733572

**TS-6 (in Dioxane)**

Zero-point correction= 0.527672 (Hartree/Particle)

Thermal correction to Energy= 0.560963

Thermal correction to Enthalpy= 0.561907

Thermal correction to Gibbs Free Energy= 0.461792

Sum of electronic and zero-point Energies= -1401.649094

Sum of electronic and thermal Energies= -1401.615803

Sum of electronic and thermal Enthalpies= -1401.614858

Sum of electronic and thermal Free Energies= -1401.714974

**TS-6d (in TFE)**

Zero-point correction= 0.528888 (Hartree/Particle)

Thermal correction to Energy= 0.561453

Thermal correction to Enthalpy= 0.562397

Thermal correction to Gibbs Free Energy= 0.464471

Sum of electronic and zero-point Energies= -1401.668327

Sum of electronic and thermal Energies= -1401.635762

Sum of electronic and thermal Enthalpies= -1401.634818

Sum of electronic and thermal Free Energies= -1401.732745

**TS-6d (in Methanol)**

Zero-point correction= 0.528821 (Hartree/Particle)

Thermal correction to Energy= 0.561413

Thermal correction to Enthalpy= 0.562357

Thermal correction to Gibbs Free Energy= 0.464775

Sum of electronic and zero-point Energies= -1401.672997

Sum of electronic and thermal Energies= -1401.640405

Sum of electronic and thermal Enthalpies= -1401.639461

Sum of electronic and thermal Free Energies= -1401.737043

**TS-6d (in Dioxane)**

Zero-point correction= 0.529694 (Hartree/Particle)

Thermal correction to Energy= 0.562371

Thermal correction to Enthalpy= 0.563315

Thermal correction to Gibbs Free Energy= 0.464814

Sum of electronic and zero-point Energies= -1401.660049

Sum of electronic and thermal Energies= -1401.627373

Sum of electronic and thermal Enthalpies= -1401.626429

Sum of electronic and thermal Free Energies= -1401.724930

**TS-7 (in TFE)**

Zero-point correction= 0.592140 (Hartree/Particle)

Thermal correction to Energy= 0.631191

Thermal correction to Enthalpy= 0.632136

Thermal correction to Gibbs Free Energy= 0.520748

Sum of electronic and zero-point Energies= -1630.810583

Sum of electronic and thermal Energies= -1630.771532

Sum of electronic and thermal Enthalpies= -1630.770588

Sum of electronic and thermal Free Energies= -1630.881975

**TS-7 (in Methanol)**

Zero-point correction= 0.591892 (Hartree/Particle)

Thermal correction to Energy= 0.631047

Thermal correction to Enthalpy= 0.631991

Thermal correction to Gibbs Free Energy= 0.520516

Sum of electronic and zero-point Energies= -1630.816536

Sum of electronic and thermal Energies= -1630.777382

Sum of electronic and thermal Enthalpies= -1630.776438

Sum of electronic and thermal Free Energies= -1630.887913

**TS-7' (in TFE)**

Zero-point correction= 0.592227 (Hartree/Particle)

Thermal correction to Energy= 0.631161

Thermal correction to Enthalpy= 0.632105

Thermal correction to Gibbs Free Energy= 0.521363

Sum of electronic and zero-point Energies= -1630.807342

Sum of electronic and thermal Energies= -1630.768408

Sum of electronic and thermal Enthalpies= -1630.767464

Sum of electronic and thermal Free Energies= -1630.878206

**TS-7' (in Methanol)**

Zero-point correction= 0.592194 (Hartree/Particle)

Thermal correction to Energy= 0.631159

Thermal correction to Enthalpy= 0.632103

Thermal correction to Gibbs Free Energy= 0.520805

Sum of electronic and zero-point Energies= -1630.813095

Sum of electronic and thermal Energies= -1630.774129

Sum of electronic and thermal Enthalpies= -1630.773185

Sum of electronic and thermal Free Energies= -1630.884483

**TS-7e (in TFE)**

Zero-point correction= 0.588587 (Hartree/Particle)

Thermal correction to Energy= 0.627251

Thermal correction to Enthalpy= 0.628195

Thermal correction to Gibbs Free Energy= 0.517724

Sum of electronic and zero-point Energies= -1630.809048

Sum of electronic and thermal Energies= -1630.770385

Sum of electronic and thermal Enthalpies= -1630.769440

Sum of electronic and thermal Free Energies= -1630.879912

**TS-7e (in Methanol)**

Zero-point correction= 0.588599 (Hartree/Particle)

Thermal correction to Energy= 0.627195

Thermal correction to Enthalpy= 0.628139

Thermal correction to Gibbs Free Energy= 0.518679

Sum of electronic and zero-point Energies= -1630.814675

Sum of electronic and thermal Energies= -1630.776078

Sum of electronic and thermal Enthalpies= -1630.775134

Sum of electronic and thermal Free Energies= -1630.884594

**TS-7e (in Dioxane)**

Zero-point correction= 0.591919 (Hartree/Particle)

Thermal correction to Energy= 0.630541

Thermal correction to Enthalpy= 0.631485

Thermal correction to Gibbs Free Energy= 0.521446

Sum of electronic and zero-point Energies= -1630.776427

Sum of electronic and thermal Energies= -1630.737806

Sum of electronic and thermal Enthalpies= -1630.736861

Sum of electronic and thermal Free Energies= -1630.846901

**TS-7f (in Methanol)**

Zero-point correction= 0.580716 (Hartree/Particle)

Thermal correction to Energy= 0.618338

Thermal correction to Enthalpy= 0.619283

Thermal correction to Gibbs Free Energy= 0.511056

Sum of electronic and zero-point Energies= -1517.401851

Sum of electronic and thermal Energies= -1517.364228

Sum of electronic and thermal Enthalpies= -1517.363284

Sum of electronic and thermal Free Energies= -1517.471511

**TS-7f’ (in Methanol)**

Zero-point correction= 0.580755 (Hartree/Particle)

Thermal correction to Energy= 0.618108

Thermal correction to Enthalpy= 0.619052

Thermal correction to Gibbs Free Energy= 0.511374

Sum of electronic and zero-point Energies= -1517.397029

Sum of electronic and thermal Energies= -1517.359676

Sum of electronic and thermal Enthalpies= -1517.358732

Sum of electronic and thermal Free Energies= -1517.466410

**PC1 (in TFE)**

Zero-point correction= 0.252456 (Hartree/Particle)

Thermal correction to Energy= 0.267103

Thermal correction to Enthalpy= 0.268047

Thermal correction to Gibbs Free Energy= 0.209266

Sum of electronic and zero-point Energies= -693.163247

Sum of electronic and thermal Energies= -693.148600

Sum of electronic and thermal Enthalpies= -693.147655

Sum of electronic and thermal Free Energies= -693.206437

**PC1 (in Methanol)**

Zero-point correction= 0.253004 (Hartree/Particle)

Thermal correction to Energy= 0.267430

Thermal correction to Enthalpy= 0.268374

Thermal correction to Gibbs Free Energy= 0.210475

Sum of electronic and zero-point Energies= -693.166296

Sum of electronic and thermal Energies= -693.151870

Sum of electronic and thermal Enthalpies= -693.150926

Sum of electronic and thermal Free Energies= -693.208825

**PC1 (in Dioxane)**

Zero-point correction= 0.253394 (Hartree/Particle)

Thermal correction to Energy= 0.267751

Thermal correction to Enthalpy= 0.268695

Thermal correction to Gibbs Free Energy= 0.211393

Sum of electronic and zero-point Energies= -693.159061

Sum of electronic and thermal Energies= -693.144704

Sum of electronic and thermal Enthalpies= -693.143760

Sum of electronic and thermal Free Energies= -693.201062

**PC2 (in Methanol)**

Zero-point correction= 0.309163 (Hartree/Particle)

Thermal correction to Energy= 0.326364

Thermal correction to Enthalpy= 0.327308

Thermal correction to Gibbs Free Energy= 0.263333

Sum of electronic and zero-point Energies= -808.874180

Sum of electronic and thermal Energies= -808.856979

Sum of electronic and thermal Enthalpies= -808.856034

Sum of electronic and thermal Free Energies= -808.920010

**PC2’ (in Methanol)**

Zero-point correction= 0.308659 (Hartree/Particle)

Thermal correction to Energy= 0.326330

Thermal correction to Enthalpy= 0.327274

Thermal correction to Gibbs Free Energy= 0.261952

Sum of electronic and zero-point Energies= -808.868370

Sum of electronic and thermal Energies= -808.850700

Sum of electronic and thermal Enthalpies= -808.849755

Sum of electronic and thermal Free Energies= -808.915077

**PC3 (in TFE)**

Zero-point correction= 0.254169 (Hartree/Particle)

Thermal correction to Energy= 0.267976

Thermal correction to Enthalpy= 0.268920

Thermal correction to Gibbs Free Energy= 0.212031

Sum of electronic and zero-point Energies= -693.174578

Sum of electronic and thermal Energies= -693.160772

Sum of electronic and thermal Enthalpies= -693.159828

Sum of electronic and thermal Free Energies= -693.216717

**PC3 (in Methanol)**

Zero-point correction= 0.254159 (Hartree/Particle)

Thermal correction to Energy= 0.267974

Thermal correction to Enthalpy= 0.268918

Thermal correction to Gibbs Free Energy= 0.211989

Sum of electronic and zero-point Energies= -693.176806

Sum of electronic and thermal Energies= -693.162992

Sum of electronic and thermal Enthalpies= -693.162047

Sum of electronic and thermal Free Energies= -693.218976

**PC3’ (in TFE)**

Zero-point correction= 0.254523 (Hartree/Particle)

Thermal correction to Energy= 0.268104

Thermal correction to Enthalpy= 0.269048

Thermal correction to Gibbs Free Energy= 0.213138

Sum of electronic and zero-point Energies= -693.179078

Sum of electronic and thermal Energies= -693.165496

Sum of electronic and thermal Enthalpies= -693.164552

Sum of electronic and thermal Free Energies= -693.220462

**PC3’ (in Methanol)**

Zero-point correction= 0.254583 (Hartree/Particle)

Thermal correction to Energy= 0.268150

Thermal correction to Enthalpy= 0.269094

Thermal correction to Gibbs Free Energy= 0.213257

Sum of electronic and zero-point Energies= -693.181314

Sum of electronic and thermal Energies= -693.167747

Sum of electronic and thermal Enthalpies= -693.166803

Sum of electronic and thermal Free Energies= -693.222639

**Cartesian coordinates for all of the species:**

**CH_3_COOH (in TFE)**

C -0.08897400 0.11848200 -0.00056400

O -0.63552700 1.20859200 0.00009200

O -0.78377300 -1.03696200 0.00014500

H -1.73374900 -0.81903800 0.00047200

C 1.39252400 -0.11701700 0.00006700

H 1.67335600 -0.69988700 0.88282100

H 1.67403000 -0.69974900 -0.88255800

H 1.91946800 0.83684700 0.00035500

**CH_3_COOH (in Methanol)**

C -0.08919300 0.11767900 -0.00046400

O -0.63477900 1.20854800 0.00006800

O -0.78456100 -1.03619200 0.00011800

H -1.73421000 -0.81676200 0.00037800

C 1.39259200 -0.11745400 0.00006300

H 1.67418700 -0.69995900 0.88284600

H 1.67475100 -0.69985800 -0.88260000

H 1.91959600 0.83638900 0.00029600

**CH_3_COOH (in Dioxane)**

C -0.09118200 0.12261500 -0.00038100

O -0.64181200 1.20259500 0.00003800

O -0.78049300 -1.04281500 0.00010600

H -1.72302500 -0.80097800 0.00029900

C 1.39452000 -0.11197600 0.00006000

H 1.68218900 -0.69270400 0.88179100

H 1.68265900 -0.69262100 -0.88156800

H 1.91659000 0.84423200 0.00025300

**MeOH(in Methanol)**

C 0.66540900 -0.01871100 0.00000000

H 1.09458700 0.98774900 -0.00001500

H 1.03115500 -0.54825600 -0.89113500

H 1.03115200 -0.54823100 0.89115100

O -0.75226800 0.12342700 0.00000000

H -1.13120400 -0.76641300 -0.00000200

**AcNH_2_ (in TFE)**

C -1.88471600 -0.97741700 0.12751400

O -2.52050200 -1.98446700 0.47255400

N -0.56455200 -1.02015300 -0.14718500

H -0.07180200 -1.89965700 -0.06521200

H -0.04965100 -0.19818100 -0.42608000

C -2.55132500 0.37349600 -0.00361700

H -2.92832100 0.67690000 0.97835000

H -1.88328500 1.14889900 -0.38471500

H -3.41248500 0.27743800 -0.67125700

**AcNH_2_ (in Methanol)**

C -1.88574700 -0.97882400 0.12436500

O -2.52293100 -1.98708200 0.46368000

N -0.56448400 -1.01966600 -0.14432700

H -0.07002900 -1.89829600 -0.06285800

H -0.04946800 -0.19616700 -0.41876600

C -2.55143600 0.37308500 -0.00456000

H -2.91803300 0.68065300 0.98019100

H -1.88520300 1.14591400 -0.39398400

H -3.41930900 0.27724000 -0.66338900

**AcNH_2_ (in Dioxane)**

C -1.90141600 -0.99029100 0.12217000

O -2.52883300 -1.99599100 0.43093400

N -0.55799300 -1.01172000 -0.11927000

H -0.05338700 -1.87037300 0.04494600

H -0.03072400 -0.16951400 -0.28743100

C -2.54805500 0.37825900 -0.00547100

H -2.43508100 0.92526600 0.93765900

H -2.09771100 0.97874200 -0.80163000

H -3.61343800 0.25247800 -0.20155500

**Cp*Rh(OAc)_2_ (in TFE)**

Rh 0.05818700 0.17269200 -0.10864500

C 1.39122200 -0.83404700 1.25610800

C 1.20393900 -1.66190000 0.10140100

C 1.64039100 -0.91647000 -1.05646700

C 2.16909000 0.35197100 -0.59236000

C 1.99905300 0.41683000 0.82459300

C -1.42479500 2.30620000 -0.01500800

O -0.76220600 2.00613000 -1.06590900

O -1.26130800 1.59633400 1.02988600

C -2.41031600 3.43695200 -0.03533100

H -3.37680100 3.04863400 -0.37734900

H -2.54113000 3.85369900 0.96534100

H -2.08775700 4.21376700 -0.73198400

O -1.75389000 -0.67443500 -0.82243300

C -2.50041700 -1.32497600 0.01628300

O -2.21888800 -1.52453900 1.21229200

C -3.81078900 -1.82232600 -0.56641300

H -4.46808200 -0.96503500 -0.75018900

H -3.64359700 -2.31969700 -1.52604600

H -4.30404000 -2.50676700 0.12616700

C 2.73103900 1.41629600 -1.47077600

H 3.78352200 1.19560300 -1.68747600

H 2.19758300 1.46150100 -2.42338800

H 2.67978700 2.39760400 -0.99580000

C 2.38918300 1.53751300 1.72892300

H 3.36851800 1.33444500 2.17957800

H 2.45681900 2.48287400 1.18673300

H 1.66694100 1.65600700 2.54042300

C 1.63815300 -1.40616100 -2.46571800

H 1.55492200 -0.57741700 -3.17267100

H 2.57205400 -1.94043900 -2.68112600

H 0.80911800 -2.09555700 -2.64141000

C 0.63486200 -3.04038500 0.06783200

H 0.01409900 -3.18576800 -0.81946300

H 1.45190400 -3.77120800 0.02846600

H 0.02960200 -3.24891700 0.95033600

C 1.05680200 -1.17107700 2.66884000

H 0.35547100 -2.00429800 2.72837900

H 1.97087800 -1.44853800 3.20847300

H 0.61760800 -0.31007900 3.17991200

**Cp*Rh(OAc)_2_ (in Methanol)**

Rh 0.04210400 0.18194400 -0.10613900

C 1.11195100 -1.21162800 1.15559100

C 0.94924500 -1.79444500 -0.14727600

C 1.58198200 -0.92852400 -1.10522200

C 2.19695800 0.17343100 -0.37618700

C 1.90375800 0.00057500 1.00660300

C -1.01639600 2.56381800 -0.12318700

O -0.50545600 2.06951000 -1.18261400

O -0.93753800 1.89917200 0.96206000

C -1.73668300 3.88014400 -0.16097600

H -2.79666000 3.69205500 -0.36775200

H -1.66065300 4.38692500 0.80339700

H -1.33858000 4.51540000 -0.95483800

O -1.88810700 -0.42566100 -0.72554800

C -2.68073000 -1.00258600 0.12265500

O -2.43491900 -1.16738800 1.33152100

C -3.97817900 -1.50520700 -0.48574800

H -4.43323400 -0.73212200 -1.11138200

H -3.76130800 -2.36421300 -1.13075600

H -4.67922400 -1.81280100 0.29210400

C 2.98004700 1.27906900 -0.99917400

H 4.01632000 0.95519000 -1.15674200

H 2.56681000 1.55394600 -1.97277100

H 2.99577400 2.16736200 -0.36445500

C 2.30685300 0.89291800 2.13130800

H 3.20640900 0.49473500 2.61669500

H 2.52835900 1.90378100 1.78386700

H 1.51999900 0.94898900 2.88754700

C 1.67678700 -1.14738200 -2.57715600

H 1.66327200 -0.19787300 -3.11779900

H 2.61778900 -1.65698300 -2.81975900

H 0.85396800 -1.76541500 -2.94201200

C 0.20701400 -3.05117000 -0.45535400

H -0.19170100 -3.03796800 -1.47156500

H 0.88604100 -3.90821300 -0.36968100

H -0.61973300 -3.20403600 0.24104400

C 0.66239200 -1.78986500 2.45407400

H -0.16000300 -2.49281300 2.31762300

H 1.49713200 -2.32608500 2.92333100

H 0.34050600 -1.00787600 3.14590200

**Cp*Rh(OAc)_2_ (in Dioxane)**

Rh 0.03842900 0.20979700 -0.09707000

C 1.20723700 -1.14351900 1.12873000

C 1.04923900 -1.72858700 -0.17075700

C 1.60609900 -0.82404400 -1.14094600

C 2.18845200 0.29926200 -0.41771900

C 1.93564500 0.10963100 0.97067500

C -1.18258500 2.43431500 0.05255700

O -0.56669800 2.09962800 -1.01149400

O -1.07974800 1.68382500 1.07569900

C -2.04093400 3.66542300 0.08712900

H -3.05953700 3.38983600 -0.20823000

H -2.07913800 4.07963600 1.09663000

H -1.67086400 4.41283600 -0.61760500

O -1.83479200 -0.39580200 -0.73651100

C -2.51860700 -1.22531000 0.00013700

O -2.13508400 -1.75867600 1.04544700

C -3.91554600 -1.48442700 -0.54815100

H -4.53588400 -0.59596400 -0.38743900

H -3.88286700 -1.66801000 -1.62580500

H -4.37398000 -2.33275000 -0.03707000

C 2.89576700 1.44876100 -1.05615700

H 3.93233300 1.17375100 -1.28702000

H 2.41150000 1.73982800 -1.99114300

H 2.91575800 2.32259800 -0.40221400

C 2.32893300 1.01718000 2.08981100

H 3.25354800 0.66316300 2.56194500

H 2.49763800 2.03719900 1.73885900

H 1.55354500 1.05069100 2.85823900

C 1.67582900 -1.03659400 -2.61783100

H 1.67067200 -0.08432100 -3.15323500

H 2.59527700 -1.57062300 -2.88940900

H 0.82632900 -1.62305800 -2.97415000

C 0.39862800 -3.04116700 -0.45732400

H 0.06022100 -3.10091100 -1.49351300

H 1.11744400 -3.85327800 -0.29114200

H -0.46168400 -3.19320800 0.19507700

C 0.77674800 -1.75526800 2.41917800

H -0.19046300 -2.24378200 2.29732900

H 1.51714600 -2.49182200 2.75685400

H 0.67485200 -0.99841600 3.20007200

**2a(in TFE)**

C 0.99529400 -0.98153400 -0.00026300

H 1.06071600 -2.06948000 -0.00028300

C 2.12679300 -0.27992000 -0.00034400

C 2.79721500 1.02457400 0.00019600

C 3.59243100 -0.29231000 0.00026400

H 2.78993300 1.61668900 0.91436800

H 2.79079400 1.61670100 -0.91398100

H 4.11599300 -0.56966100 -0.91366700

H 4.11496600 -0.57018400 0.91462400

C -0.36676700 -0.43010500 -0.00016700

C -1.46036200 -1.31424300 0.00003600

C -0.63003500 0.95361200 -0.00018900

C -2.77110700 -0.83808900 0.00017400

H -1.27312000 -2.38508800 0.00007600

C -1.93919900 1.42792000 -0.00005200

H 0.19675900 1.65585100 -0.00032700

C -3.01722300 0.53619900 0.00012900

H -3.59965900 -1.54088400 0.00032000

H -2.12178500 2.49905300 -0.00008400

H -4.03683200 0.91037900 0.00024800

**2a(in Methanol)**

C 0.99534600 -0.98148400 -0.00028000

H 1.06070400 -2.06945600 -0.00030700

C 2.12691600 -0.27989700 -0.00035700

C 2.79741900 1.02455900 0.00021300

C 3.59258200 -0.29234100 0.00027500

H 2.79012300 1.61667600 0.91441500

H 2.79102100 1.61670500 -0.91398400

H 4.11617100 -0.56968000 -0.91367700

H 4.11510600 -0.57023800 0.91466900

C -0.36676000 -0.43004300 -0.00017900

C -1.46037200 -1.31423800 0.00003800

C -0.63015000 0.95369000 -0.00020500

C -2.77116800 -0.83816100 0.00018800

H -1.27312700 -2.38509800 0.00008100

C -1.93938100 1.42790300 -0.00005700

H 0.19652200 1.65610900 -0.00035600

C -3.01739600 0.53613600 0.00013900

H -3.59964500 -1.54108600 0.00034500

H -2.12204300 2.49904800 -0.00009400

H -4.03704400 0.91027900 0.00026600

**2a(in Dioxane)**

C 0.99415200 -0.97854400 -0.00031600

H 1.06228300 -2.06612500 -0.00036800

C 2.12631500 -0.27974300 -0.00037500

C 2.79933500 1.02362600 0.00025600

C 3.59128600 -0.29403500 0.00028400

H 2.79563100 1.61705900 0.91349200

H 2.79655500 1.61719900 -0.91289700

H 4.11658000 -0.57123000 -0.91245600

H 4.11547400 -0.57182600 0.91348000

C -0.36751200 -0.42936600 -0.00020300

C -1.46039500 -1.31280500 0.00004400

C -0.63149000 0.95319300 -0.00024700

C -2.77050600 -0.83786300 0.00021900

H -1.27324500 -2.38357400 0.00009000

C -1.93970900 1.42671200 -0.00007400

H 0.19629200 1.65373200 -0.00044000

C -3.01676100 0.53531000 0.00016300

H -3.59861700 -1.54088500 0.00040100

H -2.12292700 2.49757800 -0.00012200

H -4.03630500 0.90916300 0.00031400

**2a’(in Dioxane)**

C 2.33613100 0.94396900 0.05416900

H 2.47750100 1.97179200 -0.28366700

C 3.38279200 0.13341200 0.09803300

C 3.90214000 -1.20578300 0.40603900

C 4.82053000 -0.07797100 -0.09851100

H 3.68540700 -2.02660400 -0.27551700

H 3.99599300 -1.50890200 1.44744900

H 5.52049000 0.36099400 0.61056800

H 5.20933600 -0.15603100 -1.11254100

C 0.93479900 0.53638400 0.41426600

H 0.54181000 1.19561700 1.20052300

H 0.93597200 -0.47948200 0.82384900

C -0.02663800 0.59907500 -0.79540700

H 0.34871000 -0.07182200 -1.57615100

H -0.00994300 1.61257500 -1.21376000

C -1.43924600 0.22066800 -0.42067800

C -1.85381000 -1.11709400 -0.45344600

C -2.34932600 1.18949900 0.02164000

C -3.14206800 -1.47806800 -0.05867300

H -1.15928400 -1.88068200 -0.79539700

C -3.63887000 0.83377000 0.41757500

H -2.04373300 2.23261400 0.05149900

C -4.03975200 -0.50265300 0.37869300

H -3.44628100 -2.52036600 -0.09552900

H -4.33179200 1.60033400 0.75306800

H -5.04427100 -0.78128300 0.68339900

**INT-1(in TFE)**

C -1.72827500 -1.66015000 1.02649300

C -0.42284000 -2.03244500 0.49050000

C -0.49607800 -1.96888900 -0.93972600

C -1.79978500 -1.47653600 -1.29585900

C -2.57242000 -1.33457100 -0.06944100

C -2.32114600 -1.29060100 -2.68083900

H -3.05666500 -0.48377500 -2.72220400

H -1.51327200 -1.06018000 -3.37865800

H -2.81292700 -2.21059100 -3.02174500

C -3.99266400 -0.88784900 -0.00153300

H -4.20867600 -0.13948300 -0.76743400

H -4.65356200 -1.74545400 -0.17764800

H -4.23577500 -0.46693200 0.97581500

C -2.08325700 -1.63186000 2.47476300

H -2.89327500 -0.92729700 2.67405500

H -2.41168700 -2.62721900 2.79871200

H -1.22190700 -1.35167700 3.08558300

C 0.72900900 -2.51002300 1.30846100

H 0.82929400 -1.92681100 2.22670000

H 0.56079000 -3.55559500 1.59656600

H 1.66813400 -2.45270800 0.75757600

C 0.55451400 -2.39662700 -1.90598200

H 0.28400800 -3.38601100 -2.29616900

H 0.62843700 -1.71267500 -2.75279700

H 1.53180900 -2.47500700 -1.43175300

Rh -0.86205900 0.02138600 -0.07183800

C -1.50503700 2.15481500 1.31255500

O -2.16982900 1.81890500 0.27947900

O -0.53685300 1.41726800 1.68660300

C -1.82901900 3.42719300 2.03987700

H -1.47551600 3.38967000 3.07192600

H -1.32316400 4.25226000 1.52479300

H -2.90401500 3.61984100 2.01372200

C 3.46557200 0.19132200 1.96086100

C 2.56313000 0.56516600 0.96102600

C 2.68563300 -0.00841900 -0.30573100

C 3.70655900 -0.92531500 -0.58335400

C 4.59996800 -1.28139500 0.42453500

C 4.48100400 -0.73106400 1.70523600

H 3.36846200 0.63340100 2.94865600

H 1.77321400 1.27582700 1.16158600

H 3.78548600 -1.34364900 -1.58175800

H 5.39119100 -1.99313400 0.20715100

H 5.17577500 -1.01441000 2.48972700

O 1.84725800 0.26947100 -1.36215500

N 0.69982700 1.05342700 -1.03553400

C 0.82566700 2.32010700 -1.47733400

O -0.12293000 3.13366200 -1.39756800

C 2.14714500 2.75304500 -2.08671600

H 2.97603500 2.60276900 -1.38879000

H 2.37141800 2.18034400 -2.99149800

H 2.07299600 3.81228100 -2.33827500

**INT-1(in Methanol)**

C -1.73175600 -1.66277700 1.01946000

C -0.42734900 -2.03481700 0.48097600

C -0.50069600 -1.96260400 -0.94869900

C -1.80309000 -1.46453800 -1.30170100

C -2.57543300 -1.32940500 -0.07436200

C -2.32458100 -1.26863100 -2.68516900

H -3.06119000 -0.46249400 -2.72037900

H -1.51709600 -1.03224100 -3.38146600

H -2.81534500 -2.18667200 -3.03281000

C -3.99686100 -0.88718600 -0.00396600

H -4.21614500 -0.13495500 -0.76507100

H -4.65406900 -1.74634500 -0.18660000

H -4.24277100 -0.47504300 0.97641300

C -2.08765500 -1.64512800 2.46760800

H -2.89627200 -0.94039900 2.67207000

H -2.41872200 -2.64238700 2.78302000

H -1.22625000 -1.37232800 3.08167900

C 0.72338600 -2.52052500 1.29561300

H 0.82282800 -1.94618800 2.21955900

H 0.55432200 -3.56873800 1.57357800

H 1.66314800 -2.45892700 0.74626100

C 0.54772800 -2.39047700 -1.91692700

H 0.27881600 -3.38284900 -2.30081900

H 0.61487900 -1.71109200 -2.76796200

H 1.52733300 -2.46329900 -1.44669100

Rh -0.86126600 0.02419700 -0.06876200

C -1.50626000 2.15221900 1.32879900

O -2.16397500 1.82655900 0.28779600

O -0.53935300 1.41295700 1.70159900

C -1.83863700 3.41587400 2.06830500

H -1.48494100 3.37076800 3.09997400

H -1.33919100 4.25030600 1.56212000

H -2.91496000 3.60159100 2.04476900

C 3.47337400 0.19879800 1.96584100

C 2.57040700 0.56959900 0.96536300

C 2.68982100 -0.00983400 -0.29905000

C 3.70861000 -0.93018400 -0.57358000

C 4.60251300 -1.28343700 0.43490400

C 4.48653500 -0.72691200 1.71319900

H 3.37864900 0.64601500 2.95157800

H 1.78245000 1.28311000 1.16306300

H 3.78610500 -1.35356300 -1.56995100

H 5.39193300 -1.99788100 0.21975000

H 5.18179900 -1.00797600 2.49810300

O 1.85118200 0.26583900 -1.35522100

N 0.70634300 1.05520500 -1.02994900

C 0.83045900 2.31430200 -1.49270300

O -0.11822300 3.12937600 -1.42359400

C 2.15092400 2.73814400 -2.11084000

H 2.98266500 2.58940200 -1.41611100

H 2.36839300 2.15845000 -3.01286800

H 2.08054800 3.79572200 -2.37032200

**INT-1(in Dioxane)**

C 1.59247300 -1.75123700 -1.03629000

C 0.27668100 -2.04122600 -0.47456400

C 0.38523300 -1.97439500 0.95142300

C 1.72218400 -1.56191000 1.27887600

C 2.48109900 -1.47806400 0.03791600

C 2.28159800 -1.38775100 2.65244600

H 3.03846500 -0.59990000 2.67240000

H 1.49842700 -1.12216800 3.36559400

H 2.75462600 -2.31665000 2.99623100

C 3.92810600 -1.12324200 -0.04965200

H 4.17754800 -0.32056200 0.64758500

H 4.54548200 -1.99436500 0.20174600

H 4.20229200 -0.79206800 -1.05290200

C 1.90655300 -1.75207200 -2.49575600

H 2.81442800 -1.18598400 -2.71234800

H 2.05207400 -2.77866700 -2.85397300

H 1.08940000 -1.31074700 -3.07111100

C -0.90430800 -2.48849700 -1.27135800

H -0.99935100 -1.91318100 -2.19441700

H -0.78687100 -3.54477900 -1.54683200

H -1.83604200 -2.38196100 -0.71629900

C -0.67156400 -2.31801900 1.94672900

H -0.43531800 -3.29012700 2.39771700

H -0.72730100 -1.57633900 2.74440700

H -1.65375900 -2.38935000 1.48165300

Rh 0.87315600 -0.00130000 0.06440900

C 1.69135600 2.13389000 -1.07096200

O 2.26501200 1.66283500 -0.03599600

O 0.75612700 1.45284600 -1.60795000

C 2.09575000 3.46329100 -1.62927300

H 1.92177800 3.50555500 -2.70653100

H 1.47582900 4.21808500 -1.13672600

H 3.14320900 3.67367600 -1.40321000

C -3.43599900 0.09693900 -1.92726300

C -2.50325000 0.51497400 -0.97563700

C -2.63966400 0.07235100 0.34359800

C -3.70398100 -0.76212400 0.70851900

C -4.62271100 -1.16922700 -0.25602900

C -4.49161600 -0.74749200 -1.58238000

H -3.32684800 0.43840200 -2.95295400

H -1.67227300 1.15126000 -1.25064400

H -3.79596400 -1.07590900 1.74336000

H -5.44516300 -1.81783800 0.03196600

H -5.20741700 -1.06815100 -2.33269600

O -1.77105300 0.39483200 1.35727700

N -0.62510300 1.13992800 0.93783100

C -0.79254300 2.48008600 1.13138000

O 0.07673800 3.30106100 0.82259100

C -2.10630500 2.94166900 1.75137700

H -2.95200200 2.72780300 1.09008100

H -2.30324500 2.44339500 2.70424200

H -2.03742000 4.01933300 1.90447200

**INT-2(in TFE)**

C -1.57759300 -2.01829500 0.19703500

C -1.49955800 -1.48475100 -1.15124200

C -2.30375500 -0.26006500 -1.19119600

C -2.72581500 0.02331800 0.11827200

C -2.24374200 -1.05208900 0.99462700

C -3.50512200 1.20679100 0.59099400

H -3.05343800 1.64110900 1.48823400

H -3.55269400 1.98391100 -0.17433100

H -4.53194000 0.91886100 0.84872600

C -2.52736300 -1.13482600 2.45977100

H -2.41495400 -0.15785900 2.93831500

H -3.55818700 -1.46915900 2.63231300

H -1.85674900 -1.84017500 2.95581200

C -1.08992800 -3.36194800 0.62835100

H -0.87402500 -3.39138400 1.69837300

H -1.86525900 -4.11132900 0.42386800

H -0.18979300 -3.65818300 0.08685500

C -0.94255800 -2.19028900 -2.34608900

H -0.12547100 -2.85818600 -2.06370600

H -1.71821700 -2.79236400 -2.83649500

H -0.55977700 -1.47627800 -3.07991300

C -2.56308300 0.53314300 -2.43041400

H -3.36243900 0.06032200 -3.01455500

H -2.86437700 1.55628100 -2.20120400

H -1.67343100 0.57200100 -3.06409500

Rh -0.42948700 -0.12560600 0.07806100

C 0.84821100 2.00074300 2.10523500

O -0.20182800 1.53431800 1.65107500

O 2.02589200 1.42032500 1.92384900

C 0.90285700 3.27166000 2.88898400

H 1.50822600 3.13284400 3.78844000

H 1.38464800 4.04114300 2.27564000

H -0.10558300 3.59274700 3.14869200

C 1.88826900 -1.98307000 1.08057200

C 1.42877600 -0.97372100 0.22115300

C 2.32839800 -0.52179100 -0.76578700

C 3.60525300 -1.06384500 -0.93084600

C 4.01736000 -2.07997800 -0.06755900

C 3.16784900 -2.53422300 0.94569500

H 1.23716900 -2.34579400 1.87103700

H 1.89611100 0.58447200 1.40966100

H 4.25772100 -0.68621600 -1.71259700

H 5.01038300 -2.50556000 -0.18071600

H 3.49850500 -3.31279900 1.62742100

O 1.95373700 0.48899200 -1.61258100

N 0.69037600 1.03965900 -1.18499900

C 0.53248600 2.32205800 -1.51803300

O -0.52141500 2.93193500 -1.19431700

C 1.63184900 3.01918500 -2.29285300

H 2.55762300 3.04503500 -1.70892600

H 1.85158300 2.49908300 -3.23003100

H 1.31079000 4.03951700 -2.50881000

**INT-2(in Methanol)**

C -1.56950200 -2.01859800 0.17833300

C -1.49564300 -1.46756400 -1.16286000

C -2.30793600 -0.24795400 -1.18684100

C -2.73016200 0.01677900 0.12635100

C -2.24077900 -1.06677400 0.98859500

C -3.52276600 1.18536000 0.61374600

H -3.08314600 1.60681100 1.52296000

H -3.57161400 1.97589700 -0.13766500

H -4.54916100 0.88408400 0.85782100

C -2.52784800 -1.17445500 2.45134900

H -2.43116100 -0.20355700 2.94515400

H -3.55463800 -1.52564300 2.61461600

H -1.84958900 -1.87807800 2.93940500

C -1.07676000 -3.36610600 0.59110200

H -0.85553900 -3.40845300 1.65961700

H -1.85202300 -4.11416700 0.38128400

H -0.17902100 -3.65430800 0.04139400

C -0.93917300 -2.15687400 -2.36740900

H -0.11320100 -2.81864500 -2.09647500

H -1.71197900 -2.76325900 -2.85710100

H -0.56975200 -1.43305500 -3.09849200

C -2.57652000 0.55469200 -2.41781700

H -3.35661200 0.06776400 -3.01627200

H -2.90941500 1.56562400 -2.17845700

H -1.68216700 0.62642400 -3.04202100

Rh -0.42916400 -0.11928800 0.08229700

C 0.86093500 1.97275800 2.13961400

O -0.19138000 1.51651000 1.67936300

O 2.03517600 1.38877300 1.95290600

C 0.92112800 3.23522400 2.93686300

H 1.52589900 3.08509400 3.83488800

H 1.40639200 4.00873600 2.33125500

H -0.08525600 3.55918400 3.20085700

C 1.89118600 -1.98652200 1.06267100

C 1.42904300 -0.97127100 0.21183400

C 2.32461500 -0.51191600 -0.77519400

C 3.60098500 -1.05280900 -0.94827500

C 4.01594800 -2.07539600 -0.09393700

C 3.17018900 -2.53690000 0.91913500

H 1.24304100 -2.35415100 1.85328900

H 1.90129000 0.56320500 1.42307600

H 4.25141000 -0.66937100 -1.72893100

H 5.00865600 -2.49998500 -0.21363100

H 3.50329100 -3.32027200 1.59417400

O 1.94683600 0.50444200 -1.61278100

N 0.69069000 1.06073200 -1.16846000

C 0.52560200 2.33703500 -1.52004700

O -0.52541300 2.95136300 -1.19375100

C 1.61535900 3.02472300 -2.31728600

H 2.55211300 3.04781800 -1.75130400

H 1.81387100 2.49986200 -3.25661500

H 1.29670100 4.04596700 -2.53245100

**INT-2(in Dioxane)**

C -1.42657300 -2.14111900 0.18365200

C -1.23117900 -1.64965000 -1.16816200

C -2.06778400 -0.46565600 -1.32822600

C -2.67795000 -0.17568400 -0.08610400

C -2.25598800 -1.20164100 0.85901900

C -3.57375800 0.98852800 0.19882200

H -3.66136700 1.16735500 1.27310700

H -3.16856600 1.89427700 -0.25932200

H -4.58482800 0.81810800 -0.19237900

C -2.68354300 -1.26151400 2.28989200

H -2.66095200 -0.26934000 2.74749300

H -3.71005700 -1.64076800 2.36809800

H -2.03649800 -1.91819300 2.87554200

C -0.90279500 -3.43299100 0.72293700

H -0.78062700 -3.40041600 1.80826300

H -1.59867900 -4.25007300 0.49303500

H 0.06328000 -3.68679000 0.28273100

C -0.49517600 -2.36192300 -2.25834700

H 0.36314900 -2.90751900 -1.86005500

H -1.14955100 -3.08126200 -2.76725500

H -0.12138200 -1.66021200 -3.00734100

C -2.22350900 0.32423100 -2.58613700

H -3.14284600 0.02425900 -3.10477700

H -2.27404700 1.39251300 -2.36854100

H -1.38708000 0.15385800 -3.26734500

Rh -0.40196500 -0.17475000 0.13044700

C 0.67197700 1.83589600 2.28493200

O -0.29660600 1.17183300 1.90427100

O 1.91974100 1.56346300 1.95057600

C 0.51557100 3.04398100 3.15576000

H 0.49369600 3.92753900 2.50729300

H -0.42740600 2.99178300 3.70005700

H 1.35704200 3.14637300 3.84345000

C 2.11499400 -1.85157800 0.95448900

C 1.54031700 -0.81516000 0.20465400

C 2.36256500 -0.18302200 -0.75284600

C 3.68342200 -0.58075500 -0.98415000

C 4.21031900 -1.62948400 -0.23249300

C 3.43581100 -2.26063700 0.74566900

H 1.52077300 -2.34745400 1.71715800

H 1.91665100 0.77377500 1.35273800

H 4.27821700 -0.06803200 -1.73368600

H 5.23665900 -1.94235700 -0.40140500

H 3.85834400 -3.06368300 1.34276600

O 1.86486600 0.85055700 -1.48437000

N 0.59433400 1.26297000 -0.92333300

C 0.15855200 2.44209100 -1.40746400

O -0.95468400 2.89320800 -1.07800400

C 1.07476900 3.20352800 -2.35088700

H 2.06289600 3.36119400 -1.90999800

H 1.22350200 2.64589900 -3.28141900

H 0.61096700 4.16499000 -2.57481400

**INT-3(in TFE)**

C 1.36549700 -1.94379700 -0.19466900

C 1.60069200 -1.35032600 1.08314200

C 2.30998400 -0.08134200 0.87619200

C 2.46150900 0.11444100 -0.50992400

C 1.81089800 -0.99842700 -1.18445200

C 3.16152600 1.23118400 -1.21472600

H 2.53946200 1.65500600 -2.00850000

H 3.43021400 2.03492200 -0.52857000

H 4.08165800 0.85921300 -1.68220300

C 1.76483500 -1.19030700 -2.66265200

H 1.65873300 -0.23454000 -3.18220500

H 2.69875400 -1.65460100 -3.00665600

H 0.93830800 -1.84189100 -2.95469200

C 0.86285800 -3.32276200 -0.47611300

H 0.20203300 -3.34544000 -1.34606000

H 1.71115200 -3.98531800 -0.68910300

H 0.32725900 -3.74079100 0.37824000

C 1.32440700 -1.96343400 2.41562600

H 0.52792600 -2.70809000 2.35511900

H 2.22631500 -2.46188000 2.79434200

H 1.03391000 -1.20546700 3.14744400

C 2.78686200 0.80930400 1.97518500

H 3.75216300 0.45574400 2.35945900

H 2.91208800 1.83743300 1.63087900

H 2.08289200 0.81181800 2.81179700

Rh 0.22652000 -0.07205500 -0.01266300

C -2.26122400 -1.96352300 0.04553000

C -1.70082000 -0.67049200 -0.01318000

C -2.62140800 0.39840200 -0.05449200

C -4.01192000 0.22309800 -0.04805600

C -4.51441200 -1.07110400 0.00828800

C -3.63927700 -2.16803700 0.05707000

H -1.60751000 -2.82766800 0.08437000

H -4.66714100 1.08842800 -0.08140400

H -5.58897200 -1.22934300 0.01729800

H -4.03822700 -3.17754500 0.10461500

O -2.16400400 1.68013300 -0.09674100

N -0.73446600 1.69868400 -0.06190100

C -0.27233400 2.98019100 0.01656000

O 0.95184800 3.18534600 0.08419500

C -1.26666900 4.11722500 0.02011100

H -1.87250300 4.10740700 -0.89112400

H -1.95314900 4.03205100 0.86762200

H -0.71627700 5.05666300 0.08620300

**INT-3(in Methanol)**

C 1.36832800 -1.94055800 -0.18837300

C 1.60621600 -1.34003600 1.08572900

C 2.31590800 -0.07280400 0.87054600

C 2.46288700 0.11636500 -0.51663300

C 1.80977300 -0.99984300 -1.18402900

C 3.16312000 1.22785900 -1.22908800

H 2.54543700 1.63953200 -2.03254000

H 3.42565400 2.04002600 -0.55048200

H 4.08764900 0.85384900 -1.68634300

C 1.76009900 -1.19934000 -2.66110000

H 1.65004900 -0.24651900 -3.18530400

H 2.69437700 -1.66279000 -3.00534900

H 0.93456600 -1.85465400 -2.94756400

C 0.86917900 -3.32251300 -0.46100900

H 0.20781800 -3.35279500 -1.33030300

H 1.71965000 -3.98331800 -0.67093300

H 0.33614100 -3.73743700 0.39644200

C 1.33448200 -1.94562100 2.42252700

H 0.53932300 -2.69221700 2.36894000

H 2.23844300 -2.44003100 2.80174000

H 1.04493200 -1.18348200 3.15046600

C 2.79981900 0.81916600 1.96519500

H 3.75213900 0.44749800 2.36453900

H 2.95333800 1.84034500 1.61226400

H 2.08683500 0.84635500 2.79384200

Rh 0.22739800 -0.06899600 -0.01286000

C -2.25233800 -1.97028100 0.04726700

C -1.69786100 -0.67470600 -0.01267800

C -2.62331800 0.39000800 -0.05486100

C -4.01311200 0.20756100 -0.04896300

C -4.50958600 -1.08891600 0.00836700

C -3.62935500 -2.18157500 0.05865800

H -1.59488900 -2.83136400 0.08746800

H -4.67325700 1.06907900 -0.08323500

H -5.58344200 -1.25205400 0.01712400

H -4.02335700 -3.19299600 0.10722000

O -2.17253700 1.67342600 -0.09755200

N -0.74234800 1.69981400 -0.06048400

C -0.28778500 2.98392300 0.01709500

O 0.93550100 3.19677300 0.08287100

C -1.28819000 4.11589900 0.02195000

H -1.88893600 4.10833400 -0.89266600

H -1.97855700 4.02344300 0.86542200

H -0.74367300 5.05821200 0.09517400

**INT-3(in Dioxane)**

C -1.34885100 -1.98382100 -0.00703100

C -1.70043300 -1.19701100 -1.15328800

C -2.37054500 0.01532300 -0.69341400

C -2.36225800 0.00648600 0.71625000

C -1.68520200 -1.21185700 1.15309300

C -2.93935900 1.05564900 1.60985700

H -2.53479000 0.98239300 2.62230000

H -2.71238900 2.05140200 1.22395200

H -4.02899400 0.94533400 1.67939500

C -1.50576600 -1.63603900 2.57380600

H -1.28649900 -0.78166300 3.21862100

H -2.42035900 -2.11271600 2.95034600

H -0.68661600 -2.35104100 2.67483100

C -0.83277800 -3.38673200 -0.02186800

H -0.23196700 -3.60785600 0.86311100

H -1.67031200 -4.09586000 -0.03484900

H -0.22282900 -3.58511900 -0.90604000

C -1.54438000 -1.60677000 -2.58081100

H -0.71287800 -2.30348000 -2.70678000

H -2.45738400 -2.10115000 -2.93816200

H -1.36090000 -0.74354400 -3.22490300

C -2.95882600 1.07072900 -1.57165400

H -4.02497800 0.87710300 -1.74735700

H -2.84870700 2.05539200 -1.11563000

H -2.46339700 1.09613900 -2.54561600

Rh -0.21348600 -0.09247800 -0.00247400

C 2.31443600 -1.92579300 0.01022500

C 1.71883400 -0.64826400 0.00006200

C 2.60795900 0.44845400 -0.00963300

C 4.00314300 0.31003400 -0.01100800

C 4.53979100 -0.96946700 -0.00094400

C 3.69578500 -2.09174900 0.01002100

H 1.68362500 -2.80781700 0.01854500

H 4.63548000 1.19215900 -0.01903300

H 5.61814400 -1.09876400 -0.00115000

H 4.12407000 -3.09017300 0.01841500

O 2.11032900 1.70846100 -0.01720300

N 0.68612400 1.67880600 -0.01187800

C 0.17477700 2.95773300 0.00258500

O -1.04537100 3.13181600 0.00961800

C 1.14737800 4.11812500 0.00759300

H 1.81551400 4.06884100 0.87181800

H 1.77664100 4.10425900 -0.88708300

H 0.57160500 5.04363700 0.03739100

**INT-4(in TFE)**

Rh 0.57200600 -0.35472500 -0.03603600

C 2.37254000 2.09487900 -0.69673300

C 1.26013500 1.57165600 -0.03531600

C 0.50628600 2.45250200 0.75203600

C 0.83816300 3.80277500 0.89306400

C 1.95870400 4.29652600 0.22269300

C 2.72747300 3.44535200 -0.57335900

H 2.96933200 1.45588400 -1.33675800

H 0.22417600 4.44534600 1.51737700

H 2.22493200 5.34461900 0.32491200

H 3.59799900 3.82478100 -1.10136900

O -0.61222600 2.01055000 1.40117500

N -0.76200300 0.58662100 1.23489900

C -1.79123600 0.10518000 1.93555800

O -2.08324000 -1.11743800 1.89218900

C -2.59832000 1.06123900 2.79158300

H -1.96773300 1.53971900 3.54746800

H -3.04259900 1.85328500 2.18245800

H -3.39005800 0.49389300 3.28362900

C 1.31225500 -1.84886400 1.56943600

C 0.74666800 -2.61457800 0.50311000

C 1.50395200 -2.31831900 -0.68557600

C 2.54487700 -1.39087600 -0.35223500

C 2.41019900 -1.07791300 1.04823400

C 3.68533000 -1.02873700 -1.24729200

H 4.36811100 -1.88404700 -1.33121800

H 3.35045500 -0.78209100 -2.25787300

H 4.25933800 -0.18904200 -0.85383000

C 3.33746800 -0.23324900 1.86037800

H 4.12567100 -0.85465700 2.30385700

H 3.81580200 0.53412600 1.24891800

H 2.80812800 0.26466700 2.67655000

C 1.34613200 -2.98369700 -2.01300500

H 1.56321700 -2.29815800 -2.83471600

H 2.05078000 -3.82178900 -2.08589500

H 0.34094900 -3.38764700 -2.15015800

C -0.34878800 -3.62823500 0.59885200

H -1.01138100 -3.58104700 -0.26968300

H 0.07023200 -4.64172600 0.63845300

H -0.95435800 -3.47645500 1.49250400

C 0.90310600 -1.86452700 3.00611300

H -0.10890400 -2.24682900 3.13467000

H 1.59021200 -2.50044800 3.57841000

H 0.94892400 -0.86191200 3.43894100

C -1.12670400 -0.64984800 -1.52701200

H -1.03010000 -1.71077600 -1.73865500

C -0.14090900 0.21638000 -2.00707900

C -0.17314600 1.48751000 -2.76210700

C 0.69821200 0.31750600 -3.22361700

H 0.33830900 2.36143600 -2.37334400

H -1.08491500 1.70060100 -3.31623900

H 0.39869800 -0.24608500 -4.10449100

H 1.77098000 0.44414300 -3.11005700

C -2.48754500 -0.23082300 -1.16489200

C -3.49500100 -1.20786300 -1.09305000

C -2.83389900 1.10960400 -0.91568800

C -4.80907900 -0.86124400 -0.78422000

H -3.23670000 -2.24696400 -1.27929200

C -4.14600700 1.45444700 -0.60210100

H -2.06866000 1.87537600 -0.94549000

C -5.13975700 0.47247000 -0.53443300

H -5.57338400 -1.63165300 -0.73536300

H -4.39493900 2.49345200 -0.40506200

H -6.16170800 0.74586300 -0.28829400

**INT-4(in Methanol)**

Rh 0.57211300 -0.35402500 -0.03595600

C 2.35558800 2.10463200 -0.70858900

C 1.25050900 1.57572600 -0.03937600

C 0.49915900 2.45198600 0.75533300

C 0.82655300 3.80360600 0.89557200

C 1.93977200 4.30308400 0.21740400

C 2.70602500 3.45629800 -0.58580500

H 2.95014000 1.46897300 -1.35397400

H 0.21470900 4.44294800 1.52532400

H 2.20226800 5.35219500 0.31912700

H 3.57096900 3.84009500 -1.11983800

O -0.61061200 2.00457900 1.41466500

N -0.76284000 0.58132800 1.23994500

C -1.79049400 0.09801400 1.94149200

O -2.08334000 -1.12472900 1.89578600

C -2.59673800 1.05207700 2.80085500

H -1.96597500 1.52837100 3.55793300

H -3.04127600 1.84596400 2.19432700

H -3.38866700 0.48472800 3.29252600

C 1.32676300 -1.84141900 1.56941900

C 0.76304700 -2.61294200 0.50632800

C 1.51405700 -2.31323900 -0.68536200

C 2.55048000 -1.37876800 -0.35657000

C 2.41814800 -1.06439600 1.04367100

C 3.68686100 -1.01173900 -1.25472300

H 4.37751700 -1.86133700 -1.33285200

H 3.34927900 -0.77547200 -2.26681600

H 4.25334800 -0.16415700 -0.86735300

C 3.34288500 -0.21364300 1.85231000

H 4.13485600 -0.83059600 2.29533800

H 3.81662200 0.55472100 1.23851000

H 2.81293000 0.28311300 2.66884100

C 1.35565900 -2.98200400 -2.01095800

H 1.57379100 -2.29907400 -2.83451400

H 2.05937200 -3.82115900 -2.08122200

H 0.35000500 -3.38518000 -2.14728200

C -0.32287700 -3.63593600 0.60883300

H -0.98802100 -3.59890700 -0.25823000

H 0.10575800 -4.64528000 0.65177600

H -0.92714200 -3.48665200 1.50383600

C 0.92300100 -1.85753800 3.00755600

H -0.08300000 -2.25363000 3.14177100

H 1.62056100 -2.48254600 3.57924700

H 0.95718600 -0.85306000 3.43716300

C -1.12841600 -0.65695300 -1.52377900

H -1.02981600 -1.71826100 -1.73279900

C -0.14578200 0.21033900 -2.00814400

C -0.18435500 1.47891800 -2.76717600

C 0.68977200 0.31040200 -3.22714600

H 0.32548600 2.35576600 -2.38292400

H -1.09849700 1.68726800 -3.31925300

H 0.38989200 -0.25744000 -4.10518300

H 1.76235300 0.44154300 -3.11726300

C -2.48999400 -0.23874700 -1.16271100

C -3.49803800 -1.21561500 -1.09618300

C -2.83660500 1.10108800 -0.91033000

C -4.81286100 -0.86944100 -0.78951600

H -3.23998100 -2.25421000 -1.28588100

C -4.14934200 1.44542000 -0.59861700

H -2.07120500 1.86687200 -0.93663700

C -5.14361300 0.46356200 -0.53600900

H -5.57759500 -1.63975000 -0.74521500

H -4.39842100 2.48393800 -0.39907000

H -6.16602800 0.73660900 -0.29131100

**INT-4(in Dioxane)**

Rh 0.65990900 -0.25726400 -0.06688100

C 1.57192200 2.66356300 -0.55542800

C 0.70621100 1.77313600 0.07931000

C -0.22656200 2.30169000 0.98553100

C -0.29906400 3.67280500 1.25242000

C 0.58115600 4.53922300 0.60462700

C 1.51985200 4.04029300 -0.29954000

H 2.29167500 2.29032200 -1.27680800

H -1.03611600 4.04124500 1.95923600

H 0.52940000 5.60454900 0.80965500

H 2.20476300 4.71264000 -0.80835300

O -1.09979700 1.48462200 1.62650100

N -0.82889400 0.10611300 1.29865000

C -1.74180000 -0.74209200 1.80917900

O -1.66521300 -1.96753700 1.60099300

C -2.85555400 -0.15485600 2.65583000

H -2.45986400 0.41931200 3.49861500

H -3.47049800 0.52184900 2.05656200

H -3.46902500 -0.97774000 3.02404900

C 1.71725500 -1.61795900 1.47855800

C 1.47179700 -2.41073400 0.31695700

C 2.17717700 -1.80118800 -0.77636900

C 2.86895700 -0.63862300 -0.28977500

C 2.56037800 -0.51053300 1.11116300

C 3.89105800 0.15586600 -1.03811800

H 4.84707200 -0.38316200 -1.05531600

H 3.60204000 0.33203000 -2.07715100

H 4.06911300 1.12348000 -0.56669500

C 3.11860000 0.50968900 2.05131800

H 4.05217600 0.15271500 2.50443100

H 3.32732500 1.45161000 1.54035000

H 2.41658600 0.72378800 2.86064700

C 2.28342600 -2.37589800 -2.15236600

H 2.72982100 -1.66985800 -2.85285100

H 2.91522300 -3.27222500 -2.13252800

H 1.30870400 -2.67438100 -2.54661000

C 0.67883700 -3.67765100 0.26704600

H 0.46427300 -3.97015600 -0.76437900

H 1.23102800 -4.50376200 0.73263400

H -0.26839100 -3.54257800 0.79329600

C 1.22563600 -1.90024400 2.85960200

H 0.31727400 -2.50059100 2.83586700

H 1.99852300 -2.43528100 3.42655300

H 1.00443300 -0.97406200 3.39501700

C -0.96278000 -0.82552300 -1.51572900

H -0.76004600 -1.88487800 -1.65758800

C -0.08234100 0.11364200 -2.06519900

C -0.27590100 1.31727900 -2.90160500

C 0.72497600 0.23832000 -3.30433800

H 0.12490700 2.27064000 -2.57482600

H -1.21361700 1.38408900 -3.44891000

H 0.49334800 -0.41401500 -4.14348400

H 1.77447100 0.50524100 -3.22463700

C -2.35925600 -0.50910900 -1.17432600

C -3.27952600 -1.55828500 -1.03328800

C -2.81555100 0.81039000 -1.00715700

C -4.62052900 -1.30189000 -0.75678600

H -2.93164500 -2.58197900 -1.12971800

C -4.15424900 1.06627200 -0.72678000

H -2.11244300 1.63136300 -1.07121500

C -5.06441400 0.01231800 -0.60422800

H -5.31663800 -2.12879400 -0.65132500

H -4.48728600 2.09175100 -0.59478400

H -6.10840700 0.21482700 -0.38364200

**INT-4’(in TFE)**

Rh -0.66083500 -0.21612200 0.03379600

C 1.95084700 -0.39282000 1.68725200

C 0.99889000 0.39850400 1.04242000

C 1.17604000 1.78581300 1.08587400

C 2.26115200 2.38237600 1.73472700

C 3.20077300 1.56595700 2.36460400

C 3.04738800 0.17733700 2.34354700

H 1.86593900 -1.47240700 1.65133400

H 2.36234400 3.46367700 1.73258800

H 4.05159500 2.01795900 2.86631300

H 3.78079900 -0.46306600 2.82551000

O 0.29098800 2.61417700 0.45235100

N -0.76446500 1.84973000 -0.16504100

C -1.66729800 2.63087500 -0.75594400

O -2.64331000 2.12967100 -1.37695300

C -1.49459700 4.13508800 -0.67685100

H -1.44724500 4.47626100 0.36154200

H -0.56621600 4.44662900 -1.16652300

H -2.34223700 4.60519500 -1.17800900

C -2.88466300 -1.00444800 0.07285700

C -1.98326400 -2.09453500 -0.04946200

C -1.12102300 -2.12282100 1.11577600

C -1.51796800 -1.03963200 1.97081700

C -2.56424000 -0.31977500 1.30027600

C -1.01598600 -0.75877000 3.35010400

H -1.66705100 -1.24310500 4.08875600

H -0.00254200 -1.13616500 3.49454100

H -1.01133200 0.31289100 3.56327500

C -3.27840400 0.87555500 1.84121100

H -4.12700200 0.55549900 2.45908400

H -2.62155200 1.48237700 2.46861900

H -3.66411000 1.50298100 1.03603700

C -0.17288500 -3.23133100 1.44649500

H 0.47350100 -2.97083900 2.28588000

H -0.73145700 -4.13253900 1.72904300

H 0.45873400 -3.49148600 0.59268100

C -2.00431500 -3.11878200 -1.13600100

H -1.07777200 -3.69504200 -1.16922500

H -2.82521700 -3.82416000 -0.95603200

H -2.17114900 -2.66421600 -2.11532100

C -4.04258000 -0.72790100 -0.83028400

H -3.81609700 -1.00306900 -1.86329400

H -4.91118400 -1.32163600 -0.51627000

H -4.32688100 0.32428300 -0.81314700

C 0.78580400 -1.11624100 -1.49680100

C -0.13904200 -0.24795500 -2.07620800

C -1.10789600 -0.30918300 -3.19662200

C -0.07713500 0.81993400 -3.09330600

H -2.14009400 -0.02892500 -3.01577400

H -0.96644300 -1.08129200 -3.95023800

H 0.77652900 0.80990600 -3.76804300

H -0.45954900 1.80746900 -2.86150500

C 2.22347700 -0.84901700 -1.35238100

C 3.07743600 -1.92390100 -1.06029000

C 2.77919100 0.43857200 -1.46840200

C 4.44629900 -1.72441900 -0.87993300

H 2.65702800 -2.92170200 -0.96463700

C 4.14227500 0.63878200 -1.27720400

H 2.13266400 1.28406800 -1.67110600

C 4.98315400 -0.44025600 -0.98063000

H 5.08976600 -2.56951100 -0.65228200

H 4.55260000 1.64179700 -1.35151300

H 6.04619300 -0.27760000 -0.82885700

H 0.52624900 -2.16966500 -1.43602900

**INT-4’(in Methanol)**

Rh -0.66034400 -0.21605300 0.03370300

C 1.94810100 -0.37282400 1.69408700

C 0.99650300 0.41072300 1.03929000

C 1.16923500 1.79883100 1.07179700

C 2.25025900 2.40364800 1.72024600

C 3.18938000 1.59508200 2.36100800

C 3.04016100 0.20583300 2.35053100

H 1.86557900 -1.45294700 1.66718400

H 2.34836800 3.48523100 1.71005700

H 4.03666000 2.05370600 2.86276000

H 3.77316600 -0.42845500 2.84120300

O 0.28299500 2.61956300 0.43161700

N -0.76037600 1.84668800 -0.19842300

C -1.66143300 2.62278600 -0.79876600

O -2.62955400 2.11650200 -1.42839900

C -1.49683200 4.12822500 -0.72079000

H -1.45899600 4.47117200 0.31736800

H -0.56700500 4.44466700 -1.20445900

H -2.34312800 4.59378900 -1.22840900

C -2.88864400 -0.99018200 0.09074500

C -1.99546100 -2.08701700 -0.02680400

C -1.12645800 -2.11045700 1.13399700

C -1.51268700 -1.01818100 1.98224300

C -2.55575900 -0.29578900 1.31014600

C -1.00480300 -0.73027600 3.35786100

H -1.65866100 -1.20353200 4.10127400

H 0.00557400 -1.11514600 3.50363600

H -0.99116000 0.34291500 3.56286700

C -3.25758400 0.91020500 1.84347700

H -4.10594900 0.60337700 2.46836900

H -2.59261100 1.51747200 2.46185500

H -3.64239700 1.53334700 1.03448800

C -0.18405300 -3.22234800 1.46954900

H 0.47150400 -2.95762500 2.30049900

H -0.74732200 -4.11599600 1.76660800

H 0.43802600 -3.49709800 0.61339600

C -2.02911600 -3.12124200 -1.10329400

H -1.09828200 -3.68970500 -1.15001700

H -2.83986500 -3.83209900 -0.89972200

H -2.21960500 -2.67717900 -2.08297800

C -4.05271000 -0.71751800 -0.80567400

H -3.82731500 -0.97708100 -1.84306000

H -4.91189300 -1.32697500 -0.49576600

H -4.35219400 0.33005900 -0.77355700

C 0.79224500 -1.14281200 -1.47738800

C -0.13427400 -0.28827000 -2.07407200

C -1.10225600 -0.37414100 -3.19339100

C -0.07471300 0.75981900 -3.11169800

H -2.13553300 -0.09414300 -3.01810300

H -0.95827600 -1.16058000 -3.93150500

H 0.77908500 0.73898500 -3.78606300

H -0.45980700 1.75061900 -2.89886300

C 2.22912400 -0.86786200 -1.33874600

C 3.08808500 -1.93436200 -1.03073800

C 2.77968200 0.41972000 -1.47793500

C 4.45670200 -1.72664600 -0.85770200

H 2.67197600 -2.93212300 -0.91761600

C 4.14252700 0.62836000 -1.29393600

H 2.12964500 1.25914700 -1.69417500

C 4.98841300 -0.44233900 -0.98148900

H 5.10401000 -2.56543900 -0.61786200

H 4.54897000 1.63147700 -1.38669000

H 6.05129500 -0.27313900 -0.83571000

H 0.53492200 -2.19543900 -1.39574000

**INT-4’(in Dioxane)**

Rh -0.63725600 -0.23763700 0.04816800

C 2.00007200 -0.25446300 1.66576700

C 1.00631600 0.47574200 1.01542100

C 1.11284000 1.87243000 1.01957300

C 2.17712100 2.53089300 1.64316900

C 3.15946800 1.77542500 2.28126500

C 3.07467600 0.38222700 2.29549300

H 1.97162400 -1.33755000 1.64592300

H 2.22604100 3.61513200 1.61514600

H 3.99321500 2.27925400 2.76162100

H 3.84566600 -0.21029000 2.77917600

O 0.18466800 2.63395200 0.38292700

N -0.83463400 1.79799300 -0.20262100

C -1.77160300 2.50754800 -0.84793800

O -2.71033100 1.94462800 -1.45016000

C -1.65744200 4.02273900 -0.82107900

H -1.65271000 4.40249800 0.20477800

H -0.72668400 4.35587900 -1.29051600

H -2.50888000 4.43524800 -1.36348500

C -2.81043600 -1.13405400 0.05790300

C -1.84819000 -2.18294100 0.09279000

C -1.05781200 -2.05356700 1.29849600

C -1.54994600 -0.90986300 2.01446500

C -2.60502100 -0.32942900 1.22981300

C -1.11389200 -0.45151600 3.36936900

H -1.70651800 -0.94368700 4.15091300

H -0.06061800 -0.67409900 3.54904000

H -1.24289300 0.62723100 3.48263600

C -3.41555300 0.86763100 1.60403500

H -4.29021100 0.55887400 2.19104600

H -2.83764700 1.56699900 2.21188900

H -3.76323900 1.39038600 0.71235800

C -0.07086000 -3.06236100 1.79706200

H 0.50879400 -2.67162100 2.63455200

H -0.58881100 -3.96418900 2.14713300

H 0.63092400 -3.37043100 1.01686000

C -1.74916500 -3.30170000 -0.89514200

H -0.83265000 -3.88004200 -0.76153700

H -2.59231700 -3.99068800 -0.76563900

H -1.77983500 -2.93655600 -1.92505000

C -3.89269900 -0.94409900 -0.95498800

H -3.72684000 -1.57212200 -1.83324800

H -4.86805500 -1.21874300 -0.53336900

H -3.92718100 0.09903500 -1.27548000

C 0.79202100 -1.14772500 -1.46642300

C -0.14721600 -0.29452000 -2.05109400

C -1.13104500 -0.37341800 -3.15857000

C -0.09559400 0.75111200 -3.09075100

H -2.15105900 -0.06566600 -2.96014200

H -1.00819100 -1.15942600 -3.90155000

H 0.75297600 0.72451500 -3.77114600

H -0.48046000 1.74088500 -2.87764000

C 2.22987400 -0.87462300 -1.33910800

C 3.09097900 -1.93941200 -1.03504000

C 2.77681200 0.41379100 -1.47312800

C 4.45802900 -1.72964400 -0.85940800

H 2.67863700 -2.93996600 -0.92874800

C 4.13731400 0.62457000 -1.28631100

H 2.12204700 1.25102200 -1.68061300

C 4.98486900 -0.44409400 -0.97710000

H 5.10772600 -2.56736700 -0.62287200

H 4.53939400 1.62985500 -1.36817500

H 6.04651800 -0.27210000 -0.82714700

H 0.54202600 -2.20327300 -1.39929500

**INT-5 (in TFE)**

Rh 2.47910700 8.80304700 -2.05562500

C 2.90451300 11.83876300 -3.95961000

C 1.93255000 11.38436800 -3.05086000

C 2.15950800 11.63024000 -1.68005600

C 3.28950400 12.32971700 -1.24270500

C 4.22689300 12.78080800 -2.16819400

C 4.03749900 12.52621400 -3.53219400

H 2.74709200 11.65420000 -5.01849100

H 3.41575400 12.50026300 -0.17810900

H 5.10559300 13.32037300 -1.82824600

H 4.76805100 12.87146000 -4.25741400

O 1.25295000 11.23334500 -0.71669600

N 1.14724400 9.77910400 -0.71243300

C 0.32578300 9.35646900 0.24269800

O 0.12009100 8.11833300 0.40527300

C -0.36994300 10.37464100 1.12411600

H 0.35488600 11.00632100 1.64681800

H -1.00743300 11.03645100 0.52881900

H -0.98259300 9.84261800 1.85377200

C 3.64941300 7.45592700 -0.60298400

C 2.85399600 6.75048800 -1.62098200

C 3.42793700 7.03783400 -2.92068800

C 4.40961200 8.04523100 -2.71865200

C 4.58132900 8.25519700 -1.27139100

C 5.24172500 8.71233400 -3.76360200

H 6.23351900 8.24478800 -3.81403100

H 4.78198500 8.63537500 -4.75126300

H 5.38944900 9.77137200 -3.53323400

C 5.56374700 9.21544800 -0.68661300

H 6.58476000 8.82309700 -0.77322400

H 5.53906900 10.17363300 -1.21564700

H 5.36192400 9.40433400 0.36990500

C 3.07104100 6.37172800 -4.20759200

H 3.23901000 7.03179500 -5.06198600

H 3.69387200 5.47935700 -4.34813800

H 2.02646500 6.05691500 -4.22031500

C 1.83264600 5.70057300 -1.33762500

H 1.13486000 5.59189700 -2.17066000

H 2.32800300 4.73261400 -1.18219900

H 1.26062900 5.93995100 -0.44030200

C 3.42960300 7.34126800 0.87050300

H 2.36428000 7.36955300 1.10988900

H 3.83013300 6.38930100 1.24118300

H 3.92679800 8.14875600 1.41283800

C 1.13395600 9.12253900 -3.64918100

H 1.79782200 9.04213700 -4.51911300

C 0.74440300 10.59465000 -3.53142900

C -0.63259300 11.05586000 -3.14346500

C -0.16542000 11.22295700 -4.56911300

H -0.70547700 11.94634900 -2.52703400

H -1.38369300 10.30121300 -2.93597200

H -0.58347300 10.55882900 -5.32105100

H 0.06797800 12.22416600 -4.92088200

C 0.03326200 8.12560800 -3.80910300

C -0.15167900 7.48743100 -5.04913000

C -0.84061300 7.77887900 -2.76185800

C -1.16171800 6.54165400 -5.23945200

H 0.50973400 7.73853700 -5.87426900

C -1.85393600 6.84007500 -2.95066500

H -0.69776700 8.22069600 -1.78489000

C -2.02123400 6.21402500 -4.18967100

H -1.27632500 6.06333800 -6.20862700

H -2.51002500 6.58771200 -2.12154700

H -2.80803200 5.47864400 -4.33184500

**INT-5 (in Methanol)**

Rh 2.48088900 8.80187100 -2.06193200

C 2.92657200 11.84715900 -3.94575100

C 1.94216200 11.38851200 -3.05288600

C 2.14919600 11.62732200 -1.67765100

C 3.27315500 12.32440900 -1.22142500

C 4.22379900 12.77981000 -2.13142100

C 4.05380600 12.53195800 -3.49907700

H 2.78379200 11.66775300 -5.00752300

H 3.38555500 12.48981800 -0.15447400

H 5.09755700 13.31768600 -1.77634300

H 4.79464800 12.88022100 -4.21236000

O 1.22804200 11.22453300 -0.73042800

N 1.14432100 9.76766700 -0.71799000

C 0.31921100 9.33641500 0.23106800

O 0.12735600 8.09649100 0.39412200

C -0.39973700 10.34798600 1.10155300

H 0.31022500 10.98572100 1.63720600

H -1.03346400 11.00418200 0.49610500

H -1.01996100 9.81050800 1.82075700

C 3.64673000 7.45092200 -0.61086900

C 2.86143300 6.74880300 -1.63916400

C 3.44556300 7.04565700 -2.93236700

C 4.42051100 8.05626600 -2.71656100

C 4.57933400 8.25926000 -1.26775400

C 5.25924300 8.73222100 -3.75004600

H 6.25632300 8.27469400 -3.78710100

H 4.81377300 8.65119500 -4.74393800

H 5.39343500 9.79230900 -3.51621800

C 5.55286000 9.22050800 -0.67017400

H 6.57779600 8.84051500 -0.76458100

H 5.51621400 10.18625400 -1.18460700

H 5.35132700 9.39135300 0.38951000

C 3.11409700 6.38035800 -4.22592000

H 3.26261600 7.05358400 -5.07368600

H 3.76866600 5.51190400 -4.37271700

H 2.08214100 6.02761300 -4.24478800

C 1.83961400 5.69522700 -1.37069700

H 1.14972000 5.58804900 -2.21086700

H 2.33454000 4.72729900 -1.21388400

H 1.25873600 5.93083800 -0.47838600

C 3.43210700 7.31720600 0.86185000

H 2.36814900 7.30101300 1.10633600

H 3.87243100 6.37930300 1.22354900

H 3.89948700 8.13878800 1.40976600

C 1.12885400 9.12804100 -3.65396800

H 1.78815200 9.02739300 -4.52499200

C 0.75740400 10.60624000 -3.55365900

C -0.62169300 11.09947000 -3.20960500

C -0.11208400 11.23834400 -4.62307400

H -0.69215800 11.99894900 -2.60569100

H -1.39742300 10.36648000 -3.01670800

H -0.52303200 10.57161800 -5.37673800

H 0.15070900 12.22936600 -4.98274800

C 0.02073100 8.13719500 -3.78813800

C -0.12222200 7.40709400 -4.98247400

C -0.90380100 7.88223600 -2.75729100

C -1.13256900 6.45545900 -5.14010900

H 0.57124400 7.59125700 -5.79857500

C -1.91737200 6.93860100 -2.91394000

H -0.80296600 8.40451900 -1.81646000

C -2.03759200 6.21570700 -4.10501300

H -1.21291500 5.90527900 -6.07397100

H -2.61205500 6.75968500 -2.09723100

H -2.82515900 5.47670400 -4.22209300

**INT-5 (in Dioxane)**

Rh 2.48970300 8.84645900 -2.08757700

C 2.94155500 11.76140200 -3.96425200

C 1.96231700 11.32533000 -3.05062500

C 2.17701100 11.60518300 -1.68000500

C 3.30045000 12.33287900 -1.26209400

C 4.23800500 12.76631100 -2.19275700

C 4.06541100 12.46811100 -3.55120800

H 2.78545100 11.55281600 -5.01889600

H 3.41841800 12.53278600 -0.20225300

H 5.10720200 13.32702200 -1.86222500

H 4.79740900 12.80107700 -4.28054100

O 1.28492500 11.22200100 -0.71335700

N 1.13693900 9.76709500 -0.75820900

C 0.34594800 9.31718200 0.22089500

O 0.14765900 8.08775700 0.36498400

C -0.31969800 10.32775000 1.13810700

H 0.42349900 10.92363200 1.67663900

H -0.94079800 11.02696100 0.57016100

H -0.93963200 9.78286600 1.85113700

C 3.61245100 7.51494300 -0.59439800

C 2.83537000 6.80235500 -1.62239600

C 3.45055200 7.06291800 -2.90918400

C 4.43881600 8.06212500 -2.69429700

C 4.57586500 8.29012100 -1.24683700

C 5.31063800 8.70367900 -3.72503800

H 6.29902700 8.22686800 -3.74572500

H 4.87809500 8.62112600 -4.72454900

H 5.46061700 9.76541800 -3.50900300

C 5.55454800 9.24616600 -0.64648000

H 6.57724400 8.85375800 -0.71110100

H 5.53986600 10.20748100 -1.17059800

H 5.33310500 9.43677800 0.40580400

C 3.10579200 6.39414100 -4.19922200

H 3.35490400 7.02219100 -5.05818400

H 3.66742300 5.45694000 -4.30005200

H 2.04225500 6.15562800 -4.25577700

C 1.76981800 5.79900000 -1.33354800

H 1.20054800 5.55495600 -2.23206200

H 2.21668700 4.87194600 -0.94920800

H 1.07809100 6.19789800 -0.58886400

C 3.32913300 7.43074800 0.86954500

H 2.25214000 7.47381500 1.04968900

H 3.70622800 6.48396800 1.27697500

H 3.80738600 8.24511400 1.41886400

C 1.13396400 9.09518200 -3.67593400

H 1.78961500 9.01605800 -4.55337900

C 0.75486700 10.56404700 -3.53754200

C -0.61300400 11.01765600 -3.11974100

C -0.16708700 11.22388000 -4.54596700

H -0.67433800 11.88590200 -2.47240300

H -1.35607500 10.25310600 -2.92113600

H -0.59864100 10.58263100 -5.30954400

H 0.06443200 12.23379300 -4.87336600

C 0.03374600 8.09593100 -3.82636700

C -0.21022600 7.52789200 -5.08960300

C -0.77904400 7.68650900 -2.75470100

C -1.22841800 6.59296200 -5.28331800

H 0.40979300 7.82825700 -5.93081900

C -1.79891200 6.75622500 -2.94853400

H -0.57653500 8.05454100 -1.75682100

C -2.03185800 6.20510000 -4.21104100

H -1.39219500 6.17007300 -6.27091100

H -2.40580000 6.44985500 -2.10083700

H -2.82464200 5.47649900 -4.35455600

**INT-5h (in Dioxane)**

Rh 1.21811900 8.05404100 -2.45317900

C 3.94013600 11.16560900 -0.82628500

C 2.61595500 10.71002800 -0.86631900

C 2.16282000 9.91734900 0.20770800

C 3.00295100 9.67274900 1.30265800

C 4.30338800 10.16645500 1.32991700

C 4.78881600 10.90214600 0.24766100

H 4.29574800 11.76560200 -1.65902800

H 2.61331200 9.07684300 2.12131900

H 4.93653700 9.96349500 2.18870300

H 5.80650100 11.27991900 0.24640100

O 0.91470200 9.36802100 0.33985600

N 0.23665000 8.97529200 -0.87224000

C -1.08599800 9.20118300 -0.80769700

O -1.83699500 8.85990200 -1.74744000

C -1.64549800 9.88427100 0.42890000

H -1.47643200 9.27996600 1.32542900

H -1.16376500 10.85083200 0.59993000

H -2.71719000 10.02752100 0.28483900

C 1.13797100 5.84869400 -1.86987500

C 1.20493800 5.90416200 -3.27642500

C 2.41875700 6.62071500 -3.65296500

C 3.13721300 6.94270300 -2.45365000

C 2.31798700 6.53145600 -1.34815700

C 4.48595400 7.58426200 -2.36382000

H 5.27976700 6.82586000 -2.35656800

H 4.67104600 8.24498400 -3.21487600

H 4.58270200 8.18367100 -1.45594100

C 2.68714700 6.56806300 0.09918700

H 2.99572000 5.56672100 0.42730000

H 3.51112100 7.25543900 0.28809700

H 1.84100200 6.87687600 0.71617900

C 2.92337800 6.79368000 -5.05229400

H 3.53701400 7.69354000 -5.14948600

H 3.54713700 5.94069800 -5.35060100

H 2.10180600 6.86194400 -5.76871200

C 0.20951900 5.33583100 -4.23811100

H 0.18089800 5.89834200 -5.17236200

H 0.46935300 4.29765700 -4.48284200

H -0.79845700 5.33811800 -3.81832700

C 0.07078000 5.22863900 -1.02531000

H -0.86062500 5.11231100 -1.58444400

H 0.37236800 4.23729300 -0.66223100

H -0.14515300 5.84995900 -0.15169800

C 1.67212900 9.98730400 -3.07464100

H 2.58173000 9.96532300 -3.67967600

C 1.73144600 11.07063100 -2.02550300

C 0.56278900 11.97504300 -1.72995300

C 1.74093200 12.51680300 -2.49500200

H 0.44727400 12.29214100 -0.69836500

H -0.37803100 11.82459700 -2.24790400

H 1.61571800 12.68535500 -3.56134700

H 2.39761900 13.22656300 -2.00125700

C -0.46957400 8.55485800 -5.93945400

C -0.16988700 7.93124300 -7.15919000

C -1.67595900 8.23253100 -5.30310000

C -1.04395700 7.00546900 -7.72799400

H 0.76287700 8.17132500 -7.66422100

C -2.55103400 7.30394500 -5.87084600

H -1.93474600 8.67834800 -4.34745100

C -2.23944200 6.68518300 -7.08119400

H -0.78837200 6.52936500 -8.67034300

H -3.47614300 7.06128300 -5.35610700

H -2.91936600 5.95884700 -7.51644500

C 0.42091100 9.71094200 -3.82641900

C 0.52577500 9.52738700 -5.33909100

H -0.40852800 10.35885600 -3.53889300

H -0.11111700 8.72118900 -3.39823900

H 0.40895400 10.51552100 -5.80547400

H 1.53696000 9.19459600 -5.59186100

**INT-5’(in TFE)**

Rh 1.03801600 -0.10017300 -0.16212800

C -1.18725100 2.77424700 0.11318800

C -1.23267500 1.39266700 0.38245300

C -0.85130600 0.98551200 1.67741000

C -0.44519800 1.90709400 2.64769500

C -0.40831200 3.26496300 2.34821500

C -0.78217400 3.69854800 1.07038000

H -1.48053700 3.11614000 -0.87524800

H -0.15965400 1.53353200 3.62609000

H -0.09134700 3.97965000 3.10136800

H -0.75942400 4.75596300 0.82495100

O -0.88344300 -0.35704500 2.03876100

N 0.39032800 -0.95387200 1.63904300

C 0.50940200 -2.20403500 2.10147900

O 1.46723900 -2.92973800 1.72376400

C -0.51034600 -2.72721400 3.08927700

H -0.65724600 -2.03580700 3.92337800

H -1.48050900 -2.85486500 2.59634300

H -0.16904000 -3.69387600 3.46388600

C 2.71496900 -0.96588600 -1.13927100

C 2.15304500 0.01373500 -2.05000600

C 2.24076000 1.27152500 -1.39881200

C 3.02334700 1.11902900 -0.16502400

C 3.32953600 -0.23675100 -0.01520500

C 3.34677000 2.25194100 0.75414000

H 4.04114100 2.95353900 0.27582900

H 2.44440500 2.81670900 1.01434200

H 3.80394100 1.89996000 1.68123200

C 4.11849200 -0.88264200 1.07627900

H 5.11420700 -1.15947700 0.70710500

H 4.24945600 -0.21163200 1.92817500

H 3.62671300 -1.79165100 1.42699400

C 1.75893200 2.57964300 -1.93282100

H 1.41680900 3.23640500 -1.12956800

H 2.57747600 3.09346600 -2.45409900

H 0.94207200 2.44466000 -2.64526400

C 1.66367500 -0.22290400 -3.44159500

H 0.76759100 0.36168300 -3.66494600

H 2.44059400 0.07530500 -4.15696800

H 1.44048100 -1.27517100 -3.61999100

C 2.89611900 -2.42061100 -1.41492800

H 2.12675400 -2.79159100 -2.09655600

H 3.87496400 -2.60193700 -1.87895000

H 2.84428600 -3.00040400 -0.49135200

C -1.70555200 0.44917500 -0.72955800

C -0.78570700 -0.78546400 -0.86549200

C -1.01061200 -1.71209400 -2.02707100

C -1.29172900 -2.17774000 -0.61372600

H -0.15579700 -2.14200300 -2.53932100

H -1.86494900 -1.52414000 -2.67662500

H -2.32899600 -2.32160100 -0.32076200

H -0.60483300 -2.89924500 -0.18060600

C -3.19320500 0.14299700 -0.64756000

C -3.97573900 0.22939400 -1.80679300

C -3.80735000 -0.26689200 0.54505900

C -5.33611100 -0.08570500 -1.78155700

H -3.51383700 0.55322400 -2.73598100

C -5.16683300 -0.57937000 0.57406100

H -3.21739200 -0.35130200 1.45151800

C -5.93694000 -0.49155900 -0.58899100

H -5.92452900 -0.00884100 -2.69174800

H -5.62582100 -0.89465000 1.50711000

H -6.99522000 -0.73516200 -0.56424800

H -1.56319000 1.02262700 -1.65119200

**INT-5’(in Methanol)**

Rh 1.03956800 -0.10194200 -0.16430400

C -1.21040200 2.76530100 0.12898700

C -1.24183200 1.38227500 0.39246900

C -0.85468300 0.97323200 1.68490300

C -0.45519000 1.89460100 2.65823600

C -0.43188900 3.25402200 2.36451600

C -0.81261800 3.68947300 1.08936500

H -1.50857900 3.10847500 -0.85754700

H -0.16414500 1.52029800 3.63474900

H -0.12014600 3.96842700 3.12015200

H -0.80035000 4.74809900 0.84832500

O -0.87490100 -0.37081800 2.04121600

N 0.40396300 -0.95631100 1.64111000

C 0.54104100 -2.19975500 2.11607000

O 1.50976200 -2.91563200 1.74628100

C -0.47276100 -2.72731900 3.10818500

H -0.62388000 -2.03341000 3.93944700

H -1.44291000 -2.86535700 2.61813000

H -0.12360200 -3.68912600 3.48788300

C 2.72352800 -0.94966100 -1.14856100

C 2.14461900 0.02086100 -2.05809000

C 2.22072200 1.28105000 -1.41047600

C 3.01141000 1.14024300 -0.18010600

C 3.33436200 -0.21138500 -0.02870300

C 3.32800900 2.27895300 0.73398700

H 4.01843000 2.98209800 0.25211900

H 2.42267000 2.84015500 0.99122500

H 3.78731600 1.93388900 1.66264300

C 4.13590000 -0.84512600 1.06074500

H 5.12879000 -1.12274200 0.68462800

H 4.27304500 -0.16581000 1.90506800

H 3.65032100 -1.75233900 1.42474700

C 1.72032500 2.58245700 -1.94364500

H 1.37155100 3.23476000 -1.13961400

H 2.53093300 3.10698700 -2.46672600

H 0.90373100 2.43675300 -2.65421700

C 1.65291400 -0.22782900 -3.44663100

H 0.75447200 0.35205400 -3.67275800

H 2.42740900 0.06767100 -4.16584800

H 1.43334100 -1.28235100 -3.61637100

C 2.92794000 -2.40002200 -1.43070100

H 2.13677800 -2.79175200 -2.07494200

H 3.88744900 -2.55529000 -1.94234200

H 2.93833000 -2.98139000 -0.50684000

C -1.70650900 0.43953500 -0.72367600

C -0.78584700 -0.79496000 -0.85780600

C -1.01192700 -1.72583200 -2.01566900

C -1.29275600 -2.18576300 -0.60026100

H -0.15682200 -2.15731400 -2.52635800

H -1.86628800 -1.54086400 -2.66602300

H -2.33024200 -2.32697100 -0.30670000

H -0.60817400 -2.90720800 -0.16373000

C -3.19452100 0.13258100 -0.64837800

C -3.97163300 0.21416300 -1.81163300

C -3.81427200 -0.27225400 0.54300100

C -5.33205200 -0.10123700 -1.79133300

H -3.50557800 0.53471900 -2.73988300

C -5.17375400 -0.58523300 0.56712400

H -3.22897600 -0.35155400 1.45298700

C -5.93834300 -0.50259800 -0.59995400

H -5.91635700 -0.02792000 -2.70449300

H -5.63704500 -0.89650600 1.49942000

H -6.99670400 -0.74632600 -0.57915100

H -1.56063700 1.01437500 -1.64383600

**INT-5’(in Dioxane)**

Rh 1.01148600 -0.07588800 -0.17688900

C -1.07829200 2.79099000 0.21388200

C -1.18263400 1.40003000 0.41620900

C -0.80056000 0.90745300 1.68359900

C -0.33117400 1.76611900 2.68547900

C -0.23579100 3.13250600 2.45131300

C -0.61370000 3.64733100 1.20464900

H -1.37897000 3.19376200 -0.74921900

H -0.04146600 1.32984500 3.63548800

H 0.12832500 3.79432900 3.23108000

H -0.55037900 4.71404000 1.01221100

O -0.89119500 -0.44140900 1.97787600

N 0.37177600 -1.05013000 1.53931000

C 0.43517900 -2.35576200 1.87832400

O 1.36920600 -3.07222700 1.46880000

C -0.64257700 -2.91444100 2.78699100

H -0.79639400 -2.28574000 3.66762300

H -1.59672700 -2.95809100 2.25111900

H -0.35165900 -3.92095800 3.09035500

C 2.69045500 -0.96420800 -1.11994900

C 2.21607000 0.07647100 -2.01571600

C 2.32303300 1.30161000 -1.31025000

C 3.02849700 1.06535500 -0.04426500

C 3.27365200 -0.30816000 0.06258600

C 3.33454100 2.14153400 0.94719400

H 4.08452900 2.83933900 0.55450300

H 2.43807100 2.72471600 1.18537900

H 3.71742900 1.72730200 1.88200100

C 3.93467300 -1.04357800 1.18236600

H 4.93945800 -1.36979200 0.88467300

H 4.03458100 -0.41546800 2.07048600

H 3.34778000 -1.92534500 1.44885300

C 1.92962400 2.65203000 -1.81567500

H 1.56195800 3.28820400 -1.00683300

H 2.79372100 3.15689700 -2.26755800

H 1.14971900 2.58171000 -2.57767500

C 1.76962900 -0.08337300 -3.43353400

H 0.86778200 0.49577700 -3.64739200

H 2.55747400 0.26195800 -4.11479700

H 1.56285700 -1.12585300 -3.67717800

C 2.79965300 -2.42245800 -1.41606100

H 2.13164100 -2.71231800 -2.23069400

H 3.82470700 -2.68483100 -1.71026700

H 2.52729100 -2.99873800 -0.52824700

C -1.71647200 0.53111500 -0.72907700

C -0.80363000 -0.69333600 -0.95570600

C -1.02954500 -1.52370400 -2.18869600

C -1.31789300 -2.09607200 -0.81823100

H -0.18039400 -1.92208300 -2.73359000

H -1.88148600 -1.27828500 -2.82196600

H -2.35617300 -2.25562800 -0.54091100

H -0.63403800 -2.85005200 -0.44180000

C -3.20250200 0.23167300 -0.61423000

C -4.03761300 0.45877700 -1.71484300

C -3.76289700 -0.31263700 0.55036300

C -5.39892100 0.15431200 -1.65995000

H -3.61653500 0.88180700 -2.62362200

C -5.12269100 -0.61350400 0.60890500

H -3.12308500 -0.51528600 1.40157100

C -5.94649000 -0.38249900 -0.49534000

H -6.02912400 0.33990200 -2.52520200

H -5.54034000 -1.03476500 1.51912800

H -7.00528300 -0.61973100 -0.44727200

H -1.59842300 1.15685700 -1.62084700

**INT-6 (in TFE)**

Rh 3.18055000 8.55623500 -2.22171700

C 1.21628200 12.50267100 -4.49171000

C 1.83291200 11.65392400 -3.55606900

C 2.08111400 12.17530900 -2.27366100

C 1.75475200 13.49824700 -1.96166500

C 1.16630700 14.32444100 -2.91569300

C 0.88057600 13.81975100 -4.18544600

H 1.01633900 12.11034000 -5.48460000

H 1.95977900 13.85486300 -0.95740200

H 0.91551200 15.34935100 -2.65934300

H 0.40642200 14.44607100 -4.93462500

O 2.66540900 11.45963900 -1.24080600

N 2.22821300 10.09011300 -1.17700600

C 1.08232000 9.94854700 -0.48114200

O 0.51606000 8.83172100 -0.41094300

C 0.47513300 11.14514900 0.22463300

H 1.22289200 11.70359500 0.79294200

H 0.02346600 11.83541600 -0.49463800

H -0.30525800 10.78116400 0.89519400

C 3.13959100 6.85259100 -0.63269300

C 2.45231900 6.34271600 -1.74098400

C 3.35128100 6.43481200 -2.89262700

C 4.61797600 6.91382700 -2.46076100

C 4.46586500 7.30102600 -1.07539000

C 5.87948500 6.93309600 -3.26242500

H 6.35406300 5.94406600 -3.23216300

H 5.69108600 7.17638000 -4.31157700

H 6.59942700 7.65265800 -2.86690500

C 5.55362700 7.77495500 -0.16605500

H 6.07828600 6.92311500 0.28700400

H 6.29151600 8.37198900 -0.70795200

H 5.14953200 8.38580000 0.64536300

C 3.02172600 5.92278900 -4.25689500

H 3.63196900 6.40114300 -5.02654100

H 3.21102900 4.84242200 -4.30585700

H 1.96810300 6.08172100 -4.49675100

C 1.05994400 5.80200800 -1.80317500

H 0.46056800 6.32654000 -2.55489100

H 1.06757300 4.73976200 -2.07804500

H 0.55115100 5.89782300 -0.84253800

C 2.68130400 6.93827100 0.78583300

H 1.61770000 6.72307300 0.88601000

H 3.24087200 6.21776200 1.39607900

H 2.86895700 7.93135900 1.20387500

C 1.50828900 9.26233200 -4.34259800

H 1.98259200 8.44879900 -4.88645500

C 2.30313100 10.33135000 -4.06644800

C 3.79494300 10.27822900 -4.38375800

C 4.50076900 9.93751400 -3.07381800

H 4.12240800 11.23110300 -4.82428300

H 3.97244700 9.49007700 -5.12394200

H 5.48872300 9.50296700 -3.23878400

H 4.59304800 10.80719300 -2.41983200

C 0.08228600 9.04207100 -4.07361900

C -0.51043000 7.89325400 -4.63418900

C -0.72222700 9.87610400 -3.27301100

C -1.85058800 7.58552400 -4.40640600

H 0.09410400 7.23977600 -5.25708500

C -2.05911000 9.56437800 -3.04208600

H -0.30155000 10.76268700 -2.82010700

C -2.63194100 8.42025400 -3.60526900

H -2.28220100 6.69383500 -4.85198300

H -2.65792100 10.21869200 -2.41482200

H -3.67562600 8.18320500 -3.42081300

**INT-6 (in Methanol)**

Rh 3.17715600 8.56826400 -2.21673900

C 1.17528800 12.47243100 -4.51265900

C 1.82117300 11.64747300 -3.57574600

C 2.08494500 12.19013700 -2.30550000

C 1.74711100 13.51345800 -2.00770200

C 1.13046000 14.31719300 -2.96356100

C 0.82773700 13.78956800 -4.21987900

H 0.96309100 12.06244000 -5.49579300

H 1.96602800 13.88975900 -1.01370200

H 0.87148900 15.34282300 -2.71839600

H 0.33179300 14.39822300 -4.96957300

O 2.69812800 11.49384500 -1.27679600

N 2.25130600 10.12715100 -1.17046700

C 1.14067100 10.01390700 -0.41526600

O 0.56916000 8.90448900 -0.28700300

C 0.57634000 11.23425100 0.28636200

H 1.35263600 11.79741500 0.81003800

H 0.10359300 11.91304200 -0.43018100

H -0.17969600 10.89638500 0.99722700

C 3.09742900 6.82636200 -0.64457300

C 2.43437500 6.34623800 -1.77874500

C 3.35983600 6.45940200 -2.90986800

C 4.61588500 6.92745600 -2.43796400

C 4.42970800 7.29269300 -1.05081300

C 5.89829800 6.95158600 -3.20474900

H 6.37212700 5.96254100 -3.16201000

H 5.73883800 7.19525600 -4.25848500

H 6.60698800 7.67060400 -2.78838300

C 5.49447900 7.75113800 -0.10667600

H 5.99512900 6.89230700 0.36024000

H 6.25596900 8.34077700 -0.62322500

H 5.07232900 8.36455800 0.69367900

C 3.06683000 5.95726200 -4.28622500

H 3.68951700 6.44854600 -5.03764300

H 3.26888100 4.87937900 -4.34020100

H 2.01734300 6.10663800 -4.54973800

C 1.04534900 5.80484800 -1.88117900

H 0.47210100 6.32279100 -2.65701800

H 1.06037600 4.74003600 -2.14607900

H 0.50551900 5.91014700 -0.93827100

C 2.61903800 6.86005600 0.76913900

H 1.55798000 6.62423300 0.84890700

H 3.18112900 6.12554000 1.36049600

H 2.78676100 7.83899300 1.22625100

C 1.51155900 9.24992900 -4.34017800

H 1.98698900 8.43705200 -4.88329200

C 2.30198800 10.32429500 -4.07181500

C 3.79369300 10.27325000 -4.39080300

C 4.50432100 9.93383700 -3.08313700

H 4.11968800 11.22591500 -4.83258800

H 3.97008000 9.48486000 -5.13094400

H 5.48833300 9.49246800 -3.25191800

H 4.60542100 10.80522100 -2.43332800

C 0.08651000 9.02684400 -4.06890800

C -0.51066400 7.89113700 -4.65092300

C -0.71123300 9.84461900 -3.24483900

C -1.85117000 7.58290200 -4.42566800

H 0.09115300 7.24848000 -5.28769100

C -2.04865400 9.53198800 -3.01679100

H -0.28489600 10.71929900 -2.77413700

C -2.62717300 8.40287000 -3.60436400

H -2.28695800 6.70192200 -4.88821000

H -2.64358100 10.17371200 -2.37300700

H -3.67115000 8.16535200 -3.42205800

**INT-6 (in Dioxane)**

Rh 3.04364900 8.75425900 -2.13639500

C 0.99013700 12.16391700 -4.40383500

C 1.79714400 11.58159600 -3.41901400

C 1.88864400 12.21680300 -2.16946700

C 1.25025700 13.43435400 -1.93733100

C 0.48127900 14.01766700 -2.94538100

C 0.33205900 13.37206800 -4.17260400

H 0.90324100 11.67256600 -5.36774600

H 1.35736300 13.90555000 -0.96657000

H -0.01522100 14.96581900 -2.76161100

H -0.28185300 13.81092200 -4.95282000

O 2.61528700 11.64653700 -1.14820900

N 2.17430900 10.26921000 -0.95976600

C 1.18485500 10.18005500 -0.04408400

O 0.59528100 9.10165400 0.15543200

C 0.79005300 11.41380200 0.75299600

H 1.65489800 11.99938500 1.07045200

H 0.14613100 12.06460300 0.15309200

H 0.22310900 11.07662700 1.62223700

C 3.21816100 7.17782600 -0.57211100

C 2.12852100 6.64098600 -1.38034200

C 2.63048700 6.44566100 -2.67550700

C 4.03136200 6.85353400 -2.71129300

C 4.41013200 7.19707900 -1.36993300

C 4.95870000 6.66639800 -3.87292200

H 5.36070600 5.64521000 -3.90531300

H 4.45072700 6.84236500 -4.82552500

H 5.80802600 7.35286700 -3.82104400

C 5.79187200 7.45507100 -0.85797500

H 6.17815600 6.56196200 -0.35058500

H 6.48688400 7.69724700 -1.66445400

H 5.80969400 8.27724000 -0.13781000

C 1.88417900 5.85143100 -3.82380300

H 2.23930700 6.22754500 -4.78550500

H 2.02666400 4.76261900 -3.83120600

H 0.81250700 6.04671900 -3.75928700

C 0.76771500 6.26311400 -0.89247000

H 0.02174000 6.35880300 -1.68662700

H 0.75565700 5.21890700 -0.55020500

H 0.46039800 6.91150700 -0.07456900

C 3.15972100 7.42528000 0.90106400

H 2.18830100 7.83307300 1.18149900

H 3.32052100 6.48659100 1.44813500

H 3.93386500 8.13024600 1.21416600

C 2.18355300 9.24376800 -4.28702200

H 2.87708600 8.64163600 -4.86997200

C 2.66435400 10.43151000 -3.77647800

C 4.17562300 10.69173900 -3.78021100

C 4.66188100 10.02870700 -2.49918200

H 4.38359800 11.76524300 -3.84172300

H 4.61278200 10.21463200 -4.66504200

H 5.58274300 9.45997200 -2.63071800

H 4.74847300 10.71438200 -1.65820300

C 0.75879600 8.87192900 -4.42272900

C 0.32183900 8.31220100 -5.63246900

C -0.16460200 9.02800100 -3.37571300

C -1.00971600 7.93417700 -5.80427100

H 1.03160200 8.17677000 -6.44457100

C -1.49083400 8.63993700 -3.54595900

H 0.16905000 9.40827800 -2.41799900

C -1.92044100 8.09575100 -4.75981200

H -1.33184500 7.50670800 -6.74931700

H -2.18824100 8.75351200 -2.72145000

H -2.95544600 7.79206600 -4.88597800

**INT-6a (in TFE)**

Rh 1.03378900 8.71598400 -1.72722800

C 1.04811100 13.00801700 -3.87382700

C 1.10969000 11.63955600 -3.60210600

C 2.31791100 11.08477200 -3.11446800

C 3.43900800 11.92228100 -2.95856200

C 3.36086800 13.28279500 -3.25274500

C 2.15994700 13.83730400 -3.69981800

H 0.11345400 13.43533800 -4.22479600

H 4.36850700 11.48254200 -2.61136700

H 4.23894200 13.91007000 -3.12266100

H 2.08762000 14.89890200 -3.91644800

O 2.43299500 9.77701900 -2.85566100

N 1.57165400 9.61379500 -0.18607400

C 2.42733100 10.62459400 0.02869400

O 2.01737100 11.74964200 0.39372000

C 3.90696900 10.29314400 0.01003800

H 4.19669800 9.94798000 1.00920300

H 4.14367800 9.50671900 -0.70852600

H 4.47610000 11.19659200 -0.21979300

C -0.01497500 6.71701200 -1.08054000

C -0.12635300 6.78759100 -2.52443200

C 1.17942000 6.86314800 -3.06767000

C 2.12394200 6.81924800 -1.96242700

C 1.37191300 6.68704400 -0.74192500

C 3.60685400 6.77705200 -2.11462500

H 3.93920200 7.46682300 -2.89457700

H 4.11479500 7.03214800 -1.18276500

H 3.92288400 5.76786300 -2.40784300

C 1.92585700 6.51377100 0.63187300

H 1.97003900 5.44544600 0.87744200

H 2.93432200 6.92365400 0.71448900

H 1.29245400 7.00040700 1.37700800

C 1.58574200 6.89847300 -4.50353200

H 2.26443300 7.73518900 -4.69712600

H 2.11878100 5.97637100 -4.76439200

H 0.72459900 6.99146900 -5.16575300

C -1.40513900 6.68460700 -3.28390000

H -2.22196800 7.20521800 -2.77833800

H -1.31146600 7.08375100 -4.29547400

H -1.69395900 5.62905900 -3.36407500

C -1.13885200 6.53080200 -0.11553300

H -0.98910900 7.12595100 0.78896200

H -2.09976900 6.80074700 -0.55440600

H -1.19360500 5.47781300 0.18880300

C -0.54658500 9.87441300 -2.69555500

H -1.18663900 9.09041800 -3.08867600

C -0.02410300 10.69476400 -3.84404700

C -1.07810300 11.05412600 -4.88282000

C -0.05368800 10.00013400 -5.19539800

H -0.99901900 12.01329200 -5.38332500

H -2.09650400 10.73534500 -4.68198300

H -0.37469200 8.96404000 -5.21922600

H 0.73620700 10.25229100 -5.89649300

C -1.24522200 10.55053000 -1.56865400

C -2.27203400 9.85191700 -0.90502300

C -0.93891300 11.85188300 -1.12860700

C -2.94110600 10.40930900 0.18196000

H -2.54041300 8.85925400 -1.25107700

C -1.61290100 12.41179200 -0.04685400

H -0.15631200 12.41527300 -1.61865100

C -2.60816700 11.69262000 0.62307800

H -3.72657700 9.84649000 0.67870200

H -1.35757200 13.41620100 0.27930900

H -3.12613400 12.13315700 1.46998500

**INT-6a (in Methanol)**

Rh 1.03105900 8.71617500 -1.73136400

C 1.05192700 13.00916100 -3.87815900

C 1.11169900 11.64069600 -3.60573100

C 2.31879100 11.08504100 -3.11574800

C 3.44019300 11.92198800 -2.95785600

C 3.36355200 13.28263700 -3.25225000

C 2.16400500 13.83778400 -3.70220700

H 0.11845700 13.43729200 -4.23130100

H 4.36900200 11.48193200 -2.60912700

H 4.24192200 13.90924800 -3.12068400

H 2.09285300 14.89933400 -3.91958000

O 2.43322400 9.77710100 -2.85880600

N 1.56526800 9.61241000 -0.18732800

C 2.41417900 10.61876600 0.05870300

O 1.99646800 11.72726400 0.46788800

C 3.89685400 10.30156700 0.02246200

H 4.20167200 9.96444900 1.01995400

H 4.13393400 9.51476600 -0.69545300

H 4.45482600 11.20947900 -0.21740100

C -0.01378200 6.71613700 -1.08106900

C -0.12869200 6.78712800 -2.52428000

C 1.17616800 6.86351400 -3.07028700

C 2.12334500 6.81929300 -1.96688500

C 1.37421700 6.68764100 -0.74527300

C 3.60588200 6.77482800 -2.12093200

H 3.93763300 7.45156500 -2.91228000

H 4.11560900 7.04394200 -1.19395100

H 3.92060400 5.76084000 -2.39875600

C 1.93024000 6.51530400 0.62772900

H 1.97253300 5.44719500 0.87467300

H 2.93974700 6.92310600 0.70810200

H 1.29896400 7.00418300 1.37328000

C 1.57796900 6.89190600 -4.50750900

H 2.26548500 7.71993900 -4.70604000

H 2.09978800 5.96314300 -4.76776900

H 0.71563500 6.99272000 -5.16696100

C -1.40865700 6.68337100 -3.28158500

H -2.22498500 7.20331700 -2.77468200

H -1.31683100 7.08283700 -4.29320600

H -1.69661500 5.62754400 -3.36157400

C -1.13447600 6.52838000 -0.11276600

H -0.98792900 7.13097600 0.78735400

H -2.09848000 6.78651300 -0.55197700

H -1.17926200 5.47720300 0.19944900

C -0.54671000 9.87630400 -2.70181500

H -1.18724200 9.09258700 -3.09482800

C -0.02289200 10.69696600 -3.84928700

C -1.07643100 11.05824100 -4.88808600

C -0.05310400 10.00360400 -5.20145100

H -0.99640800 12.01780000 -5.38770000

H -2.09511500 10.74006600 -4.68764300

H -0.37536900 8.96790500 -5.22621600

H 0.73709700 10.25562100 -5.90231900

C -1.24291700 10.55188300 -1.57312000

C -2.26121900 9.84896600 -0.90067900

C -0.94267600 11.85683700 -1.13941100

C -2.92673800 10.40551800 0.18877700

H -2.52529900 8.85350800 -1.24156500

C -1.61395100 12.41622100 -0.05550100

H -0.16871000 12.42505000 -1.63761700

C -2.59963000 11.69259500 0.62358200

H -3.70533700 9.83918300 0.69233700

H -1.36397100 13.42386000 0.26493000

H -3.11516700 12.13263100 1.47225600

**INT-6a (in Dioxane)**

Rh 1.06477800 8.70369000 -1.74254800

C 1.03212400 12.99112800 -3.77151500

C 1.09593400 11.61465500 -3.55520600

C 2.32273700 11.02748000 -3.16175400

C 3.46703400 11.84156600 -3.07601300

C 3.38756800 13.21060200 -3.32161000

C 2.16583800 13.79819100 -3.65121600

H 0.07946500 13.43933900 -4.03806400

H 4.41189300 11.37820800 -2.81396300

H 4.28311300 13.82099400 -3.24068900

H 2.09487300 14.86815500 -3.82065000

O 2.42338600 9.71613000 -2.92401900

N 1.63125300 9.59243900 -0.22569200

C 2.46525500 10.64945900 -0.03379000

O 2.03765200 11.73014400 0.38130800

C 3.94670500 10.33662900 -0.16119100

H 4.27899900 9.80987500 0.74034900

H 4.13945000 9.70503700 -1.03009400

H 4.49935500 11.27444400 -0.24464600

C 0.00873200 6.72081000 -1.08994700

C -0.07449600 6.75052100 -2.53695800

C 1.23900000 6.81933500 -3.05574000

C 2.16384400 6.79430800 -1.93136700

C 1.38928200 6.68227400 -0.72452600

C 3.65072500 6.78696900 -2.07113500

H 3.97021000 7.57338500 -2.76059000

H 4.14592700 6.95211000 -1.11242200

H 3.99734400 5.82545200 -2.46971400

C 1.90168000 6.58055900 0.67412900

H 1.72830300 5.57418900 1.07318000

H 2.97222200 6.78698700 0.72493200

H 1.39553500 7.29811400 1.32533600

C 1.67581800 6.90271500 -4.48159500

H 2.25663300 7.81783300 -4.64069800

H 2.31078100 6.04873000 -4.74398600

H 0.82695100 6.91312100 -5.16739200

C -1.34175400 6.62642200 -3.31631500

H -2.16078000 7.19184500 -2.86347000

H -1.22339700 6.96492000 -4.34729700

H -1.65768500 5.57613300 -3.34523500

C -1.13820900 6.58581000 -0.14168900

H -0.94082200 7.11619100 0.79283800

H -2.06152800 6.98645000 -0.56297500

H -1.31459200 5.52989800 0.10158500

C -0.54183900 9.84588800 -2.62187200

H -1.18354400 9.06574500 -3.02431000

C -0.05318400 10.68635800 -3.77587100

C -1.12900800 11.06806100 -4.77960900

C -0.11869700 10.01256800 -5.13444400

H -1.05551300 12.03252600 -5.27034100

H -2.14582300 10.75813200 -4.55821100

H -0.44494100 8.97775900 -5.16980000

H 0.65602100 10.26828900 -5.85011700

C -1.27022700 10.51975300 -1.50886000

C -2.43792500 9.91995800 -1.00610800

C -0.85408300 11.73847500 -0.94598700

C -3.16230500 10.50286600 0.03211700

H -2.78350500 8.98727800 -1.44427200

C -1.57980100 12.32345500 0.08552100

H 0.05938600 12.20475900 -1.28750700

C -2.73323400 11.71007900 0.58419100

H -4.06227500 10.01992100 0.40270100

H -1.23314700 13.25896900 0.51423000

H -3.29128400 12.17087000 1.39406700

**INT-6h(in Dioxane)**

Rh 1.02384600 8.05681800 -2.53092600

C 3.92415700 11.12931600 -0.78960300

C 2.59979200 10.67511300 -0.87982700

C 2.12202400 9.85964800 0.16532800

C 2.93193800 9.59546200 1.27929700

C 4.22949900 10.08790400 1.35327500

C 4.74380200 10.84578300 0.29963000

H 4.30386800 11.74395100 -1.60026500

H 2.52000700 8.98435700 2.07533000

H 4.83757100 9.86892800 2.22603000

H 5.76066200 11.22398800 0.33264100

O 0.88324500 9.27597900 0.25551800

N 0.15795900 9.08087900 -0.96578800

C -1.13296500 9.44873000 -0.85642100

O -1.94024700 9.25872700 -1.78808200

C -1.58166700 10.11244300 0.43461400

H -1.51621200 9.41321000 1.27424300

H -0.95516000 10.97244800 0.68450500

H -2.61703300 10.43224400 0.31141300

C 1.10258700 5.93748000 -1.91789700

C 1.22099400 5.98724800 -3.35084400

C 2.42675600 6.69918900 -3.65445100

C 3.11671900 6.99149400 -2.40708000

C 2.30895300 6.51212800 -1.35208000

C 4.45571100 7.65032100 -2.29276900

H 5.26757300 6.92696000 -2.44533500

H 4.57990500 8.43537600 -3.04439500

H 4.59477800 8.11192900 -1.31306200

C 2.62126900 6.51009100 0.10880600

H 2.82776900 5.48553800 0.44362100

H 3.49239200 7.12309900 0.33863000

H 1.77847000 6.88690100 0.69242600

C 3.01093500 6.90284600 -5.01690900

H 3.54803800 7.85283100 -5.09051000

H 3.73284300 6.10776600 -5.24491400

H 2.24185100 6.88047600 -5.79076100

C 0.30017200 5.33137200 -4.33181800

H 0.29703700 5.84465800 -5.29324000

H 0.61316000 4.29300400 -4.50418100

H -0.72794700 5.31432300 -3.96628600

C 0.03315500 5.24637300 -1.12987100

H -0.89737900 5.18313900 -1.69831800

H 0.33378500 4.22559200 -0.85905200

H -0.18238100 5.78732100 -0.20473900

C 1.70978000 10.04740100 -3.16177700

H 2.67628400 9.87293700 -3.63703500

C 1.76375400 11.07115400 -2.06494700

C 0.60963300 12.01143500 -1.83373700

C 1.84255300 12.52209700 -2.52571100

H 0.44304200 12.32887100 -0.80965600

H -0.29977900 11.87708700 -2.40752300

H 1.78779000 12.70225600 -3.59544400

H 2.49369600 13.20162400 -1.98506200

C -0.43871000 8.46035100 -5.90324900

C -0.18381700 7.78388200 -7.10397600

C -1.65053200 8.21828400 -5.24320300

C -1.10361700 6.87519300 -7.62844700

H 0.74912800 7.96963900 -7.63153800

C -2.57044400 7.30720600 -5.76536800

H -1.87518100 8.71956400 -4.30769600

C -2.30093200 6.62869300 -6.95457600

H -0.88216400 6.35639500 -8.55695000

H -3.50005800 7.12594300 -5.23321900

H -3.01723000 5.91667000 -7.35398900

C 0.55808300 9.72845500 -3.89306300

C 0.59308800 9.43407000 -5.37259800

H -0.38173500 10.13087500 -3.53030300

H -0.46958200 7.99634100 -2.89567100

H 0.44848100 10.39948000 -5.88561500

H 1.59229800 9.09820500 -5.66330100

**INT-6i(in Dioxane)**

Rh 0.89921400 7.76698200 -1.58374700

C 3.12832800 11.92065700 -1.02099600

C 2.20828700 10.87253800 -1.02229500

C 2.27796900 9.89689800 0.00408300

C 3.23660100 10.04007200 1.02578400

C 4.12365000 11.11354200 1.02213000

C 4.08356300 12.05353400 -0.00898300

H 3.09165800 12.65345600 -1.82203300

H 3.25793800 9.29925600 1.81946800

H 4.85336300 11.20964400 1.82160700

H 4.78363900 12.88325000 -0.02584400

O 1.43466900 8.86322800 0.04912000

N -0.90108900 8.14833200 -1.87549900

C -1.96983000 7.34534200 -1.73348200

O -2.36932700 6.53570800 -2.58871800

C -2.71919600 7.56908300 -0.42595200

H -2.40274300 8.48176500 0.08455800

H -3.79345900 7.60522300 -0.62679000

H -2.51833300 6.71440500 0.23014700

C 1.92362500 5.87745800 -0.43345200

C 0.87824500 5.36825100 -1.22827600

C 1.13220800 5.77708300 -2.60029600

C 2.41073700 6.44943400 -2.63953500

C 2.86416100 6.57297200 -1.30143800

C 3.15739000 6.83438300 -3.87373100

H 3.73417400 5.97662800 -4.24164700

H 2.48368600 7.14271400 -4.67502800

H 3.86276600 7.64655300 -3.68476800

C 4.14904600 7.18782500 -0.85292600

H 4.94982600 6.43712300 -0.84479100

H 4.46104700 8.00002300 -1.51361000

H 4.06447100 7.59858600 0.15380900

C 0.34204400 5.32948600 -3.78767000

H 0.61109800 5.89440400 -4.68281800

H 0.54552100 4.26944200 -3.98937100

H -0.72837900 5.45783200 -3.61297300

C -0.29786500 4.55430000 -0.79657900

H -1.20080500 4.84888600 -1.33854200

H -0.12559500 3.49156400 -1.00963700

H -0.48597200 4.65156800 0.27506000

C 2.05787000 5.83119400 1.05027400

H 1.26263800 5.24379300 1.51373100

H 3.01966200 5.39823500 1.34709000

H 2.01237800 6.85157400 1.45043400

C 1.33570000 9.41244800 -2.85543100

H 2.40811500 9.25528200 -3.00695500

C 1.15188000 10.69170400 -2.06669400

C -0.24129800 11.17919100 -1.71571900

C 0.56947700 11.94356000 -2.71678100

H -0.36900300 11.60519000 -0.72526900

H -1.07139200 10.57731800 -2.06603600

H 0.26846500 11.89209400 -3.75625700

H 0.98581700 12.90177400 -2.42353300

C 0.64987400 9.73699600 -6.66975800

C 0.90058200 8.62551600 -7.48451200

C -0.34615300 10.64017000 -7.06371900

C 0.17803200 8.41963700 -8.66013800

H 1.67596900 7.91888600 -7.19753600

C -1.07118900 10.43932500 -8.23796500

H -0.55297200 11.50933900 -6.44405100

C -0.81183300 9.32674700 -9.04049000

H 0.39010000 7.55308500 -9.28011500

H -1.83795900 11.15220100 -8.52773800

H -1.37509300 9.16952400 -9.95559900

C 0.60898200 9.28314400 -4.18782000

C 1.38507900 9.91918900 -5.36479600

H -0.39812600 9.70666700 -4.12630100

H 0.45799100 8.22754800 -4.42859600

H 1.55717500 10.98239700 -5.17227600

H 2.37427900 9.45034300 -5.42769600

**INT-6j(in Dioxane)**

Rh 3.51507700 8.70094300 -3.47758800

C 0.84584200 11.61291000 -6.19287400

C 1.67217700 11.12028800 -5.17009600

C 1.94293500 11.97039000 -4.08338200

C 1.43287100 13.27238200 -4.05487800

C 0.63628100 13.74402800 -5.09405600

C 0.32690600 12.90536600 -6.16490600

H 0.62745600 10.96239900 -7.03527100

H 1.66766200 13.89504800 -3.19823700

H 0.24475900 14.75606900 -5.05554700

H -0.30847700 13.25268400 -6.97337300

O 2.73320100 11.62115800 -3.01352600

N 2.50728600 10.26942500 -2.57019300

C 1.62499400 10.21227000 -1.55196100

O 1.31723400 9.12095900 -1.03047400

C 0.99365900 11.49973500 -1.04784100

H 1.74874400 12.25051200 -0.80162000

H 0.33194000 11.93491200 -1.80325200

H 0.40694400 11.25876800 -0.16049700

C 4.37850100 7.76130800 -1.68279200

C 3.41717800 6.75221300 -2.05951200

C 3.74929800 6.33903000 -3.37317900

C 4.94179300 7.02991500 -3.81094100

C 5.37035600 7.87398100 -2.72639400

C 5.70398300 6.73212800 -5.06531900

H 6.40530700 5.90084500 -4.91470000

H 5.03538300 6.44777500 -5.88264500

H 6.28758700 7.59393800 -5.39862600

C 6.66586800 8.61225700 -2.61454900

H 7.39156800 8.01802300 -2.04421100

H 7.10682400 8.81232700 -3.59337000

H 6.54102400 9.56765900 -2.09877300

C 3.04106400 5.30266600 -4.18513400

H 2.96237300 5.59537600 -5.23619400

H 3.59904200 4.35790600 -4.15825200

H 2.03629000 5.10699300 -3.80877200

C 2.35318200 6.19922600 -1.16636100

H 1.65765400 5.55929200 -1.71422700

H 2.80027700 5.58884500 -0.37041900

H 1.78510600 7.00849700 -0.70619800

C 4.43080900 8.44254800 -0.35386300

H 3.42188300 8.62447500 0.01890300

H 4.96486200 7.81549000 0.37235900

H 4.95423100 9.39919100 -0.41842600

C 1.60798500 8.61714000 -5.22751500

H 2.02665800 7.69721800 -5.63314300

C 2.29479900 9.77960100 -5.41407800

C 3.70325900 9.84216200 -5.99579000

C 4.57023100 9.99632600 -4.75573800

H 3.78853000 10.66039900 -6.72479100

H 3.93217200 8.90297400 -6.51265000

H 5.59679200 9.65895700 -4.89895900

H 4.56090600 11.00687700 -4.34815900

C -1.42300300 7.40091200 -3.13409300

C -2.45845500 6.75145400 -3.81792900

C -1.72610600 8.10200700 -1.95856700

C -3.76928400 6.79804100 -3.34206200

H -2.23406200 6.20164600 -4.72955700

C -3.03670300 8.14944300 -1.48195600

H -0.92094200 8.59790400 -1.42195700

C -4.06244700 7.49958200 -2.17126100

H -4.56029100 6.28509200 -3.88240300

H -3.25684700 8.69276600 -0.56690500

H -5.08185800 7.53561100 -1.79762100

C 0.21208300 8.54778300 -4.67324100

H -0.49056000 8.42811300 -5.51118900

H -0.04075800 9.49629700 -4.19298500

C -0.01231600 7.40064200 -3.67135600

H 0.69283000 7.52931900 -2.84826300

H 0.20384000 6.44440800 -4.16267900

**INT-7 (in TFE)**

Rh 1.75876800 7.63778300 -2.31970800

C 4.46042200 9.41410600 -5.87146600

C 3.60006700 9.55343700 -4.76690900

C 4.17997900 9.93535600 -3.54282700

C 5.55487500 10.18762900 -3.44906200

C 6.37399300 10.05995100 -4.56465000

C 5.82620500 9.66445500 -5.78854400

H 4.02111900 9.11431600 -6.81834200

H 5.95679600 10.49020500 -2.48692600

H 7.43688900 10.26366400 -4.47570600

H 6.45531300 9.55188800 -6.66577100

O 3.50648200 10.13481400 -2.35381600

N 2.15584100 9.68546000 -2.35018700

C 1.27229500 10.68681400 -2.49582900

O 0.04681400 10.44387600 -2.59593400

C 1.77990000 12.11548700 -2.51127500

H 0.92508100 12.78096000 -2.64103500

H 2.29054000 12.35724200 -1.57380900

H 2.48849200 12.28297400 -3.32492900

C 0.19688100 7.69661800 -0.60695000

C -0.47925600 7.14876000 -1.73998000

C 0.23771400 5.96933800 -2.13418700

C 1.34177000 5.76763200 -1.22847600

C 1.32179400 6.85413000 -0.28124200

C 2.24454400 4.57340400 -1.19629600

H 1.78221600 3.75845700 -0.62518400

H 2.44703600 4.19822500 -2.20238200

H 3.20140000 4.81088000 -0.72612400

C 2.18368400 7.00097600 0.93424300

H 1.68360500 6.56928200 1.81099300

H 3.14147800 6.49096800 0.81057300

H 2.38384400 8.05271400 1.15391200

C -0.17348200 5.05096300 -3.23938200

H 0.61696600 4.34255400 -3.49413000

H -1.05274900 4.47216500 -2.93094600

H -0.45025800 5.60525700 -4.14106900

C -1.74329700 7.64394100 -2.36767300

H -1.87070600 8.71666800 -2.21482200

H -1.75077500 7.45220000 -3.44453300

H -2.61569700 7.13355000 -1.93895900

C -0.21729300 8.88416100 0.20030700

H 0.64940300 9.47734100 0.50407900

H -0.89991400 9.53094800 -0.35005300

H -0.71971800 8.54625600 1.11558000

C 1.29178500 10.06485700 -5.64401400

H 0.28899600 9.66544500 -5.78656300

C 2.15311100 9.24876900 -4.98855400

C 1.67324600 7.92510000 -4.56754600

H 0.63738500 7.70258300 -4.81089500

C 1.45906500 11.41273900 -6.19061000

C 2.60835700 12.22036500 -6.04945000

C 0.36639500 11.95265600 -6.90112900

C 2.65227600 13.50019800 -6.59712200

H 3.46812800 11.85477800 -5.50592000

C 0.41405500 13.23120100 -7.45110100

H -0.53178600 11.35154500 -7.01649400

C 1.56068400 14.01423600 -7.30257300

H 3.54801400 14.10167600 -6.46947300

H -0.44490700 13.61670300 -7.99300800

H 1.60287100 15.01295300 -7.72711700

C 2.53365600 6.87315900 -4.22674700

H 3.60572200 7.00343600 -4.31429200

H 3.28851200 7.52143000 -2.05877800

H 2.18381200 5.84919900 -4.29352300

**INT-7 (in Methanol)**

Rh 1.75967600 7.63575100 -2.32503300

C 4.44739200 9.40413600 -5.88001400

C 3.59188100 9.54981400 -4.77253600

C 4.17891000 9.92475600 -3.54947400

C 5.55662100 10.16347100 -3.45996100

C 6.37105000 10.02971700 -4.57833800

C 5.81580800 9.64128800 -5.80108900

H 4.00237300 9.10983700 -6.82596000

H 5.96506100 10.46083400 -2.49895100

H 7.43611200 10.22320500 -4.49227400

H 6.44101100 9.52391200 -6.68051100

O 3.51092700 10.13020400 -2.35913500

N 2.15855000 9.68374300 -2.34912300

C 1.27726800 10.68802500 -2.48647600

O 0.04987800 10.44941000 -2.57755900

C 1.78864500 12.11534100 -2.50414700

H 0.93532300 12.78328100 -2.63144900

H 2.30226200 12.35660900 -1.56816700

H 2.49534700 12.28059800 -3.31984800

C 0.22305700 7.68458000 -0.58747000

C -0.47054600 7.14228100 -1.71237100

C 0.24134000 5.96594400 -2.12441000

C 1.36002200 5.76015500 -1.23761200

C 1.35443000 6.84168300 -0.28451300

C 2.26445900 4.56676200 -1.22751500

H 1.81162900 3.74706500 -0.65557500

H 2.45248200 4.19927000 -2.23926500

H 3.22792400 4.80192000 -0.76980100

C 2.23493500 6.98329700 0.91825100

H 1.74914400 6.54639600 1.80047400

H 3.19146800 6.47529100 0.77742400

H 2.43710800 8.03419900 1.14039000

C -0.18934400 5.05333300 -3.22689200

H 0.59353500 4.34150800 -3.49496600

H -1.06737000 4.47834500 -2.90779600

H -0.47604900 5.61189900 -4.12289300

C -1.74760200 7.63388900 -2.31595800

H -1.89843700 8.69709000 -2.12386100

H -1.75818700 7.47983400 -3.39888400

H -2.60671000 7.09074100 -1.90086700

C -0.17879500 8.86643900 0.23414600

H 0.69101900 9.46436600 0.51919100

H -0.88013300 9.51062100 -0.29547900

H -0.65667400 8.52201200 1.16005800

C 1.28578100 10.08365000 -5.64190300

H 0.27930700 9.69321700 -5.78366200

C 2.14152400 9.25851000 -4.99042100

C 1.65176300 7.93742500 -4.57229600

H 0.61127300 7.72673400 -4.80631100

C 1.46388200 11.43088600 -6.18688700

C 2.61962000 12.22909400 -6.04455100

C 0.37571100 11.98038300 -6.89705000

C 2.67417000 13.50897800 -6.59121800

H 3.47608000 11.85619900 -5.50075500

C 0.43404300 13.25892600 -7.44608900

H -0.52736600 11.38676600 -7.01330900

C 1.58706700 14.03240300 -7.29675900

H 3.57475000 14.10305800 -6.46274700

H -0.42159900 13.65189100 -7.98793100

H 1.63758000 15.03101100 -7.72071900

C 2.50452400 6.87497100 -4.24616500

H 3.57696900 6.99431500 -4.34431400

H 3.29288300 7.51452500 -2.09039200

H 2.14367000 5.85503600 -4.31461800

**INT-7 (in Dioxane)**

Rh 1.73565300 7.62844500 -2.38025000

C 4.47761800 9.34819300 -5.79714600

C 3.56924200 9.53659400 -4.74099600

C 4.08989200 9.95160700 -3.49914800

C 5.46407300 10.18636500 -3.35095000

C 6.33295100 10.01205100 -4.42064900

C 5.84140500 9.58262000 -5.65634700

H 4.07747500 9.02825800 -6.75469400

H 5.82380200 10.50990000 -2.37957600

H 7.39345400 10.20310600 -4.28621800

H 6.51097300 9.43310000 -6.49715200

O 3.36259100 10.19541300 -2.36362300

N 2.03504900 9.67874600 -2.38345500

C 1.09050700 10.63423900 -2.54470100

O -0.11121300 10.33582100 -2.63799800

C 1.54096100 12.08493200 -2.57735600

H 0.65472600 12.70747900 -2.70443400

H 2.05474600 12.35933900 -1.65098200

H 2.23040300 12.27502600 -3.40274400

C 0.26286200 7.69213100 -0.61322900

C -0.46684200 7.11009700 -1.69750400

C 0.24003600 5.93205300 -2.09883300

C 1.39243300 5.76165500 -1.24495600

C 1.40370200 6.86263000 -0.31655300

C 2.31613700 4.58238500 -1.23398600

H 1.91425900 3.77556600 -0.60781900

H 2.46489500 4.17747400 -2.23801900

H 3.29853100 4.85300400 -0.84098000

C 2.33079200 7.04716700 0.84466600

H 1.89113900 6.63476500 1.76228500

H 3.28703900 6.54674700 0.67753900

H 2.53632600 8.10545400 1.02206900

C -0.20202900 4.98594000 -3.17092200

H 0.58923300 4.28597500 -3.44703900

H -1.05669100 4.39356900 -2.82285300

H -0.52189500 5.51424800 -4.07402900

C -1.74776200 7.62518700 -2.27234600

H -1.67279700 8.70072100 -2.44385400

H -1.97779400 7.14012600 -3.22499800

H -2.59036200 7.43234900 -1.59574100

C -0.12795800 8.91238200 0.15473400

H 0.75305800 9.45930000 0.49901300

H -0.72414800 9.58544000 -0.45990700

H -0.70814800 8.62567200 1.04167300

C 1.29932000 10.09901100 -5.68236600

H 0.28760700 9.72910400 -5.83721500

C 2.12627300 9.25977700 -5.01416100

C 1.61087800 7.94500100 -4.60551200

H 0.56639900 7.76036900 -4.84354400

C 1.50523000 11.44793500 -6.21198100

C 2.69174400 12.20379100 -6.09976500

C 0.41266800 12.04770700 -6.87113200

C 2.76914500 13.49080900 -6.62452300

H 3.55550100 11.78986700 -5.59975000

C 0.49338000 13.33347300 -7.39713600

H -0.51472400 11.48849200 -6.96177100

C 1.67586400 14.06460900 -7.27714800

H 3.69439000 14.05057700 -6.52079700

H -0.36834000 13.76587500 -7.89744600

H 1.74452500 15.06911800 -7.68398900

C 2.44539400 6.86052600 -4.29344000

H 3.51865800 6.95839800 -4.40269200

H 3.27227800 7.58412200 -2.18955500

H 2.06391600 5.84900000 -4.38040000

**INT-8 (in TFE)**

Rh 1.63078300 7.68107200 -2.15476600

C 3.93882400 10.90063100 -5.34949700

C 3.29751100 10.30329200 -4.24941200

C 3.87753100 10.51032100 -2.98635500

C 5.03704500 11.27519700 -2.83291500

C 5.65134800 11.84661400 -3.94366900

C 5.09499900 11.66390400 -5.21208400

H 3.50933300 10.74172300 -6.33362600

H 5.43456200 11.41762400 -1.83271600

H 6.55229400 12.43889900 -3.81490100

H 5.56053300 12.10639000 -6.08730500

O 3.38300100 9.98331100 -1.79975400

N 1.97548800 9.69396800 -1.81332000

C 1.19854300 10.76140300 -1.57606900

O -0.05025200 10.66009100 -1.65766500

C 1.84002200 12.07830300 -1.17978300

H 1.04405900 12.78538300 -0.94169600

H 2.48903800 11.94633300 -0.30860400

H 2.45470300 12.49437900 -1.98212700

C -0.06697200 7.17131300 -0.64939100

C -0.40925800 7.11482100 -2.08013900

C 0.31832500 6.00314500 -2.66991200

C 1.23696100 5.55151600 -1.69155300

C 0.95336800 6.24515500 -0.42415400

C 2.24227200 4.45659900 -1.84139700

H 1.86867400 3.52992400 -1.38708200

H 2.45853100 4.25168300 -2.89207000

H 3.18067100 4.71004400 -1.33882700

C 1.68898200 5.97491500 0.84798600

H 1.38966900 5.00650800 1.26823800

H 2.76925300 5.92993000 0.67517200

H 1.49511400 6.74369300 1.59898200

C 0.09051700 5.43118200 -4.03043700

H 1.00273400 5.00121400 -4.45125100

H -0.65876700 4.63149100 -3.97395400

H -0.28813200 6.18425000 -4.72536800

C -1.54342400 7.83749300 -2.72647100

H -1.69234600 8.81840100 -2.27508700

H -1.36548600 7.97523100 -3.79588700

H -2.47026100 7.25908000 -2.61164800

C -0.71136900 8.08446400 0.34278600

H -0.81920300 9.09209100 -0.06285400

H -1.71302200 7.71580600 0.59883200

H -0.13159600 8.14468600 1.26691800

C 1.00788000 9.82620000 -5.15188200

H 0.30274300 9.04196900 -5.42649700

C 2.13413100 9.40910900 -4.51810100

C 2.27581600 7.97796600 -4.13494700

H 1.60768000 7.37810200 -4.75450500

C 0.54782300 11.17493900 -5.48661400

C 1.02614000 12.35494800 -4.87885100

C -0.48111400 11.30718300 -6.44265300

C 0.51489800 13.60136600 -5.23204800

H 1.79572100 12.29481300 -4.12167500

C -0.98944500 12.55469800 -6.79781300

H -0.87961900 10.41039000 -6.91085200

C -0.49106900 13.71302800 -6.19660900

H 0.89991600 14.49251600 -4.74351800

H -1.77788300 12.62226400 -7.54256500

H -0.88621700 14.68748100 -6.46853000

C 3.69366200 7.39958800 -4.17113400

H 4.34666300 7.83459200 -3.40535700

H 3.67034100 6.31717100 -4.01049700

H 4.18094600 7.57924200 -5.14126500

**INT-8 (in Methanol)**

Rh 1.62326800 7.68378500 -2.15356400

C 3.93737800 10.89671100 -5.34997400

C 3.29309700 10.30208800 -4.25007700

C 3.87116600 10.50986600 -2.98612500

C 5.03152300 11.27369400 -2.83216500

C 5.64844000 11.84279500 -3.94264100

C 5.09429000 11.65868800 -5.21181700

H 3.50950000 10.73692000 -6.33466700

H 5.42789700 11.41750000 -1.83170400

H 6.54978100 12.43433100 -3.81299400

H 5.56193500 12.09921900 -6.08693000

O 3.37477100 9.98490300 -1.80037100

N 1.96598600 9.69899500 -1.81507500

C 1.19229600 10.76598200 -1.56781900

O -0.05769000 10.66562000 -1.63926800

C 1.83814400 12.08174800 -1.17441800

H 1.04549000 12.79283300 -0.93721500

H 2.48634000 11.94967700 -0.30263100

H 2.45448600 12.49373300 -1.97753200

C -0.06406800 7.16468000 -0.63878000

C -0.41494300 7.10890700 -2.06739000

C 0.31447800 6.00201200 -2.66370000

C 1.24139800 5.55314600 -1.69170400

C 0.96209000 6.24313500 -0.42130300

C 2.25070400 4.46305000 -1.84933500

H 1.88335700 3.53367300 -1.39536400

H 2.46248800 4.26138100 -2.90153100

H 3.19053400 4.71939600 -1.35093500

C 1.70667000 5.97431900 0.84581500

H 1.41641900 5.00245900 1.26447300

H 2.78628700 5.93749300 0.66692500

H 1.51133700 6.73920000 1.60043600

C 0.08122600 5.43131500 -4.02379400

H 0.99282600 5.00596400 -4.45059800

H -0.66421700 4.62821900 -3.96411900

H -0.30482100 6.18365100 -4.71544800

C -1.55803300 7.82631500 -2.70399800

H -1.71200600 8.80400500 -2.24696000

H -1.38763100 7.97041300 -3.77390700

H -2.48009900 7.24097100 -2.58624200

C -0.70690200 8.07309000 0.35869000

H -0.82579500 9.07983100 -0.04596200

H -1.70368400 7.69722200 0.62329700

H -0.11992900 8.13809100 1.27796600

C 1.00466800 9.83065800 -5.15764900

H 0.29771700 9.04850800 -5.43362100

C 2.12773100 9.41067200 -4.51993600

C 2.26447100 7.97931400 -4.13555300

H 1.59314400 7.38154700 -4.75370100

C 0.55140500 11.18062800 -5.49743900

C 1.03127200 12.36001700 -4.88946100

C -0.47172600 11.31506100 -6.45944700

C 0.52747500 13.60785400 -5.24844500

H 1.79575800 12.29811000 -4.12714700

C -0.97259400 12.56401200 -6.82029000

H -0.87152800 10.41895500 -6.92789500

C -0.47240900 13.72162400 -6.21913300

H 0.91353200 14.49849600 -4.75975800

H -1.75657100 12.63322600 -7.56961600

H -0.86160600 14.69717900 -6.49573400

C 3.68030900 7.39639200 -4.17466900

H 4.33707300 7.83019200 -3.41150800

H 3.65438800 6.31427800 -4.01276200

H 4.16531200 7.57328700 -5.14652600

**INT-8 (in Dioxane)**

Rh 1.63228900 7.67876300 -2.13224000

C 3.84301400 11.02853000 -5.35445100

C 3.29793800 10.34196900 -4.25468700

C 3.90829600 10.54963000 -3.00618200

C 5.01173800 11.39767800 -2.87069200

C 5.53154600 12.05666900 -3.97992400

C 4.93822400 11.87707300 -5.23128900

H 3.38805400 10.86938600 -6.32625600

H 5.43961300 11.53114200 -1.88245000

H 6.38820300 12.71375200 -3.86377600

H 5.33008100 12.38698500 -6.10569700

O 3.51443000 9.92990300 -1.83596200

N 2.11028900 9.63453400 -1.73983500

C 1.34939700 10.68570600 -1.35049400

O 0.11433500 10.57720500 -1.27580400

C 2.03562700 11.99525500 -0.99610900

H 1.28444500 12.65949000 -0.56744100

H 2.84837900 11.83926900 -0.28151900

H 2.46978000 12.47548000 -1.87797000

C -0.09725000 7.18858000 -0.62844900

C -0.43855200 7.23044200 -2.05568100

C 0.22941000 6.11517800 -2.71487900

C 1.13128900 5.56770100 -1.76972400

C 0.88881700 6.21359200 -0.46803900

C 2.07168200 4.42403000 -1.97835300

H 1.63540400 3.48792000 -1.60611300

H 2.30519000 4.28370600 -3.03618000

H 3.01254000 4.58013800 -1.44229800

C 1.63006000 5.85672100 0.77873600

H 1.32570100 4.86779100 1.14386500

H 2.70912100 5.81458400 0.59719800

H 1.45185800 6.58059900 1.57616400

C -0.03398600 5.63053900 -4.10286200

H 0.84394900 5.15079800 -4.54273400

H -0.84485900 4.89153600 -4.09415300

H -0.34041500 6.44498200 -4.76313500

C -1.48348700 8.11415300 -2.64644100

H -1.34661300 9.13469400 -2.28174400

H -1.42681400 8.12768900 -3.73695400

H -2.48497700 7.76364400 -2.36199800

C -0.70996100 8.06080400 0.41807700

H -0.78463500 9.08847600 0.06097500

H -1.71654100 7.70055700 0.66816200

H -0.12002700 8.05890400 1.33797000

C 1.07123300 9.69031600 -5.17914700

H 0.42112300 8.85944800 -5.45271000

C 2.20220200 9.36423300 -4.50453800

C 2.41021000 7.95610400 -4.06092800

H 1.81941800 7.30011400 -4.70271500

C 0.55488000 11.00624300 -5.56223900

C 0.84739300 12.18939200 -4.85560600

C -0.32626200 11.09788600 -6.65677400

C 0.30720400 13.40931100 -5.24989100

H 1.48854700 12.14408600 -3.98519800

C -0.86454500 12.31987700 -7.05288300

H -0.58109700 10.19343100 -7.20391000

C -0.54590400 13.48542300 -6.35403600

H 0.54398400 14.30575400 -4.68359000

H -1.53624700 12.36199900 -7.90588800

H -0.96648300 14.43957700 -6.65759500

C 3.85382800 7.45378400 -3.99905000

H 4.44336200 7.94724100 -3.21986500

H 3.87737700 6.37635300 -3.80321300

H 4.38080300 7.62428800 -4.94960000

**INT-8d (in TFE)**

Rh 1.78546300 7.88574200 -2.18664900

C 4.93561000 10.03615400 -5.47617000

C 3.82077700 9.96229300 -4.62954900

C 3.78921600 10.82894300 -3.53088600

C 4.80879800 11.74431400 -3.27475600

C 5.90168200 11.80875400 -4.13877800

C 5.96497800 10.94696600 -5.23603900

H 4.97909900 9.38020300 -6.33999000

H 4.72526900 12.39774900 -2.41215300

H 6.69775800 12.52292600 -3.95323200

H 6.81431400 10.98766100 -5.91118000

O 2.68532600 10.82465400 -2.68128200

N 2.81942100 9.71549700 -1.70273600

C 2.42964500 10.19208000 -0.40057500

O 2.96449600 9.68882900 0.57104000

C 1.39894700 11.27279700 -0.36027000

H 0.96301700 11.31163200 0.63801000

H 1.89431200 12.22674900 -0.57397200

H 0.62996800 11.12707300 -1.11964000

C 0.28417800 7.46634700 -0.43880500

C -0.49649100 7.92515300 -1.58480500

C -0.27742800 7.01698800 -2.64178700

C 0.67568300 6.01457200 -2.18140500

C 0.94605000 6.25802500 -0.78288100

C 1.07339400 4.78785600 -2.94465800

H 0.30233700 4.00988100 -2.86732700

H 1.21188600 5.00041000 -4.00858400

H 2.00601600 4.36403500 -2.56225800

C 1.71237600 5.36364800 0.14345700

H 1.06164200 4.59513300 0.58473400

H 2.52122100 4.84177100 -0.37640900

H 2.15511900 5.92773100 0.96949300

C -0.91970000 7.04737900 -3.99395100

H -0.27810000 6.59008900 -4.75177600

H -1.86708300 6.49138800 -3.99363400

H -1.13851900 8.07114500 -4.31158000

C -1.46390200 9.06937900 -1.58563400

H -1.16073100 9.85956600 -0.89474500

H -1.56020600 9.51282300 -2.58140200

H -2.46652000 8.74233700 -1.27607600

C 0.22494500 8.06300500 0.93358300

H 1.17743800 7.96916500 1.46172200

H -0.04361100 9.12098600 0.90558900

H -0.53814900 7.55296800 1.53679100

C 1.87684500 9.20591700 -5.98787600

H 1.18970000 8.38917200 -6.20631500

C 2.71818600 9.00465600 -4.93590100

C 2.61187300 7.76566000 -4.15561500

H 2.00132300 7.01929300 -4.66011900

C 1.75538400 10.34014700 -6.90432400

C 2.20511400 11.65113600 -6.63491700

C 1.11288300 10.11526800 -8.14140500

C 2.04421500 12.66975500 -7.57167100

H 2.66599600 11.88256600 -5.68360300

C 0.95667400 11.13410000 -9.07752500

H 0.74168400 9.11807400 -8.36469500

C 1.42734500 12.42031400 -8.80065200

H 2.39700300 13.66995600 -7.33480100

H 0.46432700 10.92514100 -10.02340500

H 1.30637100 13.21870200 -9.52697000

C 3.54750900 7.28180000 -3.17639300

H 4.44297900 7.86922800 -2.96725200

H 3.81915200 9.49161700 -1.66691000

H 3.71893300 6.20803500 -3.11783300

**INT-8d (in Methanol)**

Rh 1.78557300 7.88627900 -2.18492200

C 4.93739500 10.04525800 -5.46864300

C 3.82135400 9.96763100 -4.62385900

C 3.78429000 10.83486800 -3.52552800

C 4.80009600 11.75445900 -3.26872300

C 5.89391500 11.82288100 -4.13123200

C 5.96260100 10.96049000 -5.22772400

H 4.98505800 9.38879000 -6.33187600

H 4.71329600 12.40776300 -2.40633200

H 6.68676900 12.54046100 -3.94493000

H 6.81290400 11.00410900 -5.90149600

O 2.68066400 10.82481700 -2.67668100

N 2.82178600 9.71336600 -1.69892700

C 2.44599600 10.18921100 -0.39205800

O 2.99261000 9.68662200 0.57344500

C 1.41478800 11.26882700 -0.34052100

H 0.98933600 11.30827300 0.66222000

H 1.90703000 12.22316700 -0.55967500

H 0.63767600 11.12203200 -1.09134800

C 0.27799800 7.46951500 -0.44354300

C -0.49889800 7.92919600 -1.59146800

C -0.27816800 7.02051600 -2.64759500

C 0.67199200 6.01654100 -2.18440100

C 0.93881600 6.25961900 -0.78520500

C 1.06918700 4.78917600 -2.94689300

H 0.29485000 4.01396900 -2.87404000

H 1.21340300 5.00217100 -4.00998200

H 1.99835300 4.36138500 -2.56051300

C 1.70028700 5.36346700 0.14351600

H 1.04620200 4.59710500 0.58363700

H 2.50883000 4.83878600 -0.37402900

H 2.14287800 5.92633600 0.97049600

C -0.91763100 7.05035800 -4.00102300

H -0.27386400 6.59450800 -4.75785500

H -1.86394600 6.49249000 -4.00254300

H -1.13827300 8.07374300 -4.31866000

C -1.46443500 9.07501600 -1.59457100

H -1.16022400 9.86575100 -0.90465900

H -1.55997300 9.51708100 -2.59104000

H -2.46767400 8.75002900 -1.28472300

C 0.21390400 8.06626100 0.92843500

H 1.15912800 7.95658900 1.46628600

H -0.03848400 9.12815500 0.89884100

H -0.56287400 7.56737600 1.52354700

C 1.88325900 9.20492700 -5.98717900

H 1.19898400 8.38605000 -6.20655300

C 2.72263700 9.00634800 -4.93296100

C 2.61613600 7.76704600 -4.15292400

H 2.00636300 7.02084700 -4.65863500

C 1.75963000 10.33778800 -6.90506600

C 2.20866700 11.64981100 -6.63910800

C 1.11485700 10.11007200 -8.14053300

C 2.04472200 12.66652300 -7.57744300

H 2.67158600 11.88375900 -5.68941200

C 0.95556000 11.12704100 -9.07819800

H 0.74421600 9.11211000 -8.36135200

C 1.42539100 12.41427800 -8.80464700

H 2.39702400 13.66752900 -7.34315900

H 0.46131200 10.91577100 -10.02260900

H 1.30208300 13.21127600 -9.53212800

C 3.54949400 7.28201900 -3.17229100

H 4.44492600 7.86852400 -2.96038800

H 3.82173500 9.48909500 -1.67326000

H 3.72033800 6.20807900 -3.11469000

**INT-8d (in Dioxane)**

Rh 1.75055300 7.93179500 -2.17053700

C 4.94625900 10.02393900 -5.44649800

C 3.82111900 9.96865700 -4.61299900

C 3.78144600 10.84845900 -3.52246400

C 4.81136500 11.75621000 -3.27046600

C 5.91337600 11.80287000 -4.12269500

C 5.98045400 10.92866900 -5.20869400

H 4.98990000 9.35755100 -6.30170200

H 4.72385300 12.42397000 -2.41920500

H 6.71269800 12.51341800 -3.93728900

H 6.83583500 10.95498000 -5.87663200

O 2.67746800 10.87307200 -2.68688800

N 2.77016900 9.74054200 -1.70249800

C 2.38974300 10.26185300 -0.40647900

O 2.98013300 9.86478800 0.57465100

C 1.28734000 11.27804600 -0.41408600

H 0.84615000 11.33510000 0.58091500

H 1.71731700 12.25210400 -0.66961600

H 0.53579800 11.05033600 -1.16967100

C 0.24288900 7.53051400 -0.43796700

C -0.54439100 7.94841000 -1.59384200

C -0.30308000 7.02398100 -2.63021400

C 0.65911200 6.04395000 -2.14335500

C 0.92171900 6.32271000 -0.75282500

C 1.08378500 4.80956000 -2.87919700

H 0.32554700 4.01974400 -2.79847200

H 1.23553500 5.00198600 -3.94535100

H 2.01849000 4.40768500 -2.47898600

C 1.71160000 5.47402000 0.19507400

H 1.08113300 4.70915300 0.67067500

H 2.52672800 4.95255800 -0.31458800

H 2.15522100 6.07593700 0.99314800

C -0.93081800 7.02798300 -3.98922400

H -0.28845700 6.54290400 -4.72892800

H -1.88648500 6.48657800 -3.98835300

H -1.12625200 8.04627900 -4.33741200

C -1.53212200 9.07408600 -1.63486200

H -1.28202700 9.86648600 -0.92540300

H -1.58504200 9.52499300 -2.63038200

H -2.54347600 8.72900200 -1.37864400

C 0.18012100 8.14791100 0.92583300

H 1.16200800 8.18216100 1.40440900

H -0.21034000 9.16736500 0.89356600

H -0.48655900 7.56957600 1.57881300

C 1.89652100 9.19695300 -5.99045200

H 1.20595500 8.38016300 -6.19752700

C 2.72018700 9.01106700 -4.92384400

C 2.60162200 7.78524100 -4.12633500

H 1.99968700 7.03079500 -4.62887600

C 1.79045000 10.31042800 -6.93245700

C 2.25190600 11.62156300 -6.69080000

C 1.15448100 10.06431400 -8.16770200

C 2.10775800 12.61896500 -7.65127000

H 2.70951000 11.86808300 -5.74215100

C 1.01415000 11.06181700 -9.12753500

H 0.77580000 9.06575000 -8.37114500

C 1.49591000 12.34814100 -8.87716400

H 2.46982300 13.62047200 -7.43550500

H 0.52581300 10.83587400 -10.07143400

H 1.38769300 13.13075000 -9.62223600

C 3.52064200 7.31702200 -3.12644400

H 4.41638800 7.90420700 -2.91723000

H 3.76468200 9.50177300 -1.65497700

H 3.68994000 6.24586700 -3.03861500

**INT-9 (in TFE)**

Rh 1.56655600 8.15127700 -2.19716400

C 3.86291000 10.77686300 -5.47217500

C 3.24337500 9.92074500 -4.54539900

C 3.98018600 9.51869300 -3.39429800

C 5.27802200 10.04487900 -3.20284300

C 5.85075900 10.91143300 -4.12510200

C 5.14469300 11.27942400 -5.27726400

H 3.31509200 11.04485600 -6.36954400

H 5.82222300 9.73723200 -2.31385900

H 6.85344700 11.29312700 -3.95064600

H 5.59212600 11.93882700 -6.01462800

O 3.54331500 8.64259900 -2.48839700

N 1.14525100 9.97001100 -2.17643400

C -0.00480100 10.61144400 -1.96416700

O -1.03927300 10.41202100 -2.65172600

C 0.03259100 11.71812300 -0.92765700

H -0.81384100 11.59443900 -0.24516800

H 0.96486800 11.72077900 -0.35789900

H -0.07699200 12.68401400 -1.43307000

C 0.24019900 7.44223800 -0.57251300

C -0.28137500 6.91789200 -1.79088800

C 0.67641900 5.95694800 -2.29960500

C 1.79930900 5.91921800 -1.42229000

C 1.55691100 6.84885200 -0.36312200

C 3.03566100 5.09237100 -1.56302200

H 3.06782700 4.31598800 -0.78956900

H 3.08577200 4.60319900 -2.53705000

H 3.93110200 5.71106900 -1.44481400

C 2.45706400 7.08889300 0.80174300

H 2.28561400 6.32654500 1.57281500

H 3.50712900 7.02804500 0.50429200

H 2.27852900 8.06738200 1.25247700

C 0.38216100 5.04633900 -3.44178400

H 1.22921700 4.40986200 -3.69748200

H -0.45340100 4.39552600 -3.15414000

H 0.06256200 5.59144400 -4.33407100

C -1.63745300 7.14404400 -2.37242400

H -2.08142200 8.06988300 -2.00741200

H -1.59345500 7.19146200 -3.46382000

H -2.30184000 6.31280400 -2.10424600

C -0.46832900 8.34614200 0.38165600

H -1.22971000 8.94305000 -0.12317400

H -0.97056300 7.73870100 1.14490200

H 0.22415200 9.01766700 0.89322900

C 0.87613400 10.08153600 -5.39179200

H -0.01178800 9.51280100 -5.66348100

C 1.88758700 9.38683100 -4.80408800

C 1.63126400 7.98501500 -4.39102500

H 0.61254900 7.68009700 -4.62617700

C 0.76035600 11.52759600 -5.61002100

C 1.30206300 12.46362100 -4.70456400

C -0.00149000 12.01468200 -6.68803800

C 1.10724500 13.82926300 -4.88941600

H 1.85647900 12.10713400 -3.84477800

C -0.18756700 13.38403100 -6.87756400

H -0.44661000 11.30725300 -7.38304700

C 0.36894500 14.29897900 -5.98116600

H 1.52623900 14.53197100 -4.17415500

H -0.77253700 13.73641400 -7.72263200

H 0.22004700 15.36544800 -6.12361000

C 2.67004500 6.97145300 -4.82928400

H 3.68544500 7.29158200 -4.59617100

H 2.51664400 5.98552500 -4.39597900

H 2.60097200 6.86445200 -5.92200300

**INT-9 (in Methanol)**

Rh 1.56604800 8.15689600 -2.19207600

C 3.85971700 10.76633000 -5.48453500

C 3.24103800 9.91740300 -4.55044000

C 3.97857800 9.52430300 -3.39650700

C 5.27595400 10.05434400 -3.21000100

C 5.84748300 10.91412100 -4.13928800

C 5.14104700 11.27169200 -5.29449200

H 3.31143600 11.02616800 -6.38400800

H 5.82169500 9.75560900 -2.31892300

H 6.84973900 11.29842000 -3.96788900

H 5.58772900 11.92514900 -6.03762000

O 3.54331100 8.65510100 -2.48380800

N 1.14119400 9.97458200 -2.17595300

C -0.00134000 10.62575700 -1.95564200

O -1.03919200 10.44628400 -2.64378100

C 0.05139200 11.71991000 -0.90614600

H -0.78964200 11.59283100 -0.21757600

H 0.98843200 11.71035400 -0.34437900

H -0.05730800 12.69252400 -1.39876400

C 0.24330900 7.45149000 -0.56170700

C -0.28197600 6.92847500 -1.77946800

C 0.67145900 5.96382600 -2.28866800

C 1.79603200 5.92389000 -1.41318700

C 1.55799600 6.85461600 -0.35393300

C 3.02526300 5.08632700 -1.55200000

H 3.04135600 4.30133700 -0.78659300

H 3.07916600 4.60681500 -2.53061800

H 3.92658400 5.69281200 -1.41833300

C 2.45650600 7.08954100 0.81316200

H 2.27005200 6.33453600 1.58802100

H 3.50717500 7.01201300 0.52209400

H 2.28921100 8.07307800 1.25734900

C 0.37254300 5.05213800 -3.42876000

H 1.21722400 4.41261900 -3.68479300

H -0.46441900 4.40427400 -3.13841700

H 0.05284000 5.59660600 -4.32143400

C -1.63857600 7.15950200 -2.35786600

H -2.07333000 8.09296200 -2.00093500

H -1.59865900 7.19442200 -3.44993200

H -2.30895200 6.33703900 -2.07779900

C -0.46141600 8.35589400 0.39456800

H -1.22403000 8.95353900 -0.10730100

H -0.96193700 7.74813500 1.15876800

H 0.23311000 9.02616800 0.90496200

C 0.87408400 10.07257000 -5.39805400

H -0.01367300 9.50204400 -5.66673400

C 1.88517300 9.38180800 -4.80518200

C 1.62932000 7.98200900 -4.38452900

H 0.61068000 7.67552900 -4.61790100

C 0.76007900 11.51703000 -5.62853800

C 1.30030900 12.46007700 -4.72948600

C 0.00240800 11.99583600 -6.71318700

C 1.10882900 13.82441700 -4.92715800

H 1.85145400 12.11035500 -3.86472300

C -0.18049900 13.36384000 -6.91544800

H -0.44156200 11.28311500 -7.40353100

C 0.37497100 14.28571400 -6.02550100

H 1.52676400 14.53273800 -4.21679300

H -0.76207900 13.70976400 -7.76554100

H 0.22875700 15.35117500 -6.17808300

C 2.66812100 6.96708100 -4.81942100

H 3.68339800 7.28604400 -4.58384800

H 2.51210400 5.98157100 -4.38612700

H 2.60212600 6.85948900 -5.91231100

**INT-9 (in Dioxane)**

Rh 1.03319100 8.15950700 -2.43998400

C 4.02705500 10.27653600 -5.58579800

C 3.14069800 9.81635100 -4.60758100

C 3.62903400 9.51987600 -3.31146100

C 4.99996900 9.70530400 -3.04100200

C 5.85942200 10.18483100 -4.02494400

C 5.37907300 10.47178900 -5.30591800

H 3.63783500 10.49158700 -6.57652100

H 5.36191700 9.48323400 -2.04156700

H 6.91092000 10.32940300 -3.79164100

H 6.04988600 10.83527500 -6.07798900

O 2.83464700 9.12301400 -2.31787200

N -0.25657900 9.46367700 -2.79284100

C -1.16382200 10.03323000 -1.98143900

O -2.28401500 9.56370200 -1.71702000

C -0.70978100 11.39220300 -1.46215100

H -0.36808200 11.26637000 -0.42824200

H 0.10935700 11.80946100 -2.05229400

H -1.56077100 12.07862800 -1.46809500

C 0.32384800 7.35388000 -0.31200800

C -0.26187800 6.62671700 -1.41635900

C 0.79417100 5.91974400 -2.10732500

C 2.02549300 6.30475200 -1.51179600

C 1.72544600 7.17678100 -0.38575000

C 3.40152900 5.84588500 -1.87010800

H 3.79068400 5.15616000 -1.11091300

H 3.41678000 5.32854600 -2.83063000

H 4.08832600 6.69514800 -1.93039700

C 2.77085000 7.80116800 0.47057100

H 3.43192400 7.04024700 0.90004400

H 3.38476300 8.47012800 -0.14536100

H 2.33641500 8.38019900 1.28764500

C 0.55625300 4.84836600 -3.12203900

H 1.48901700 4.47106400 -3.54233200

H 0.04649100 4.00470200 -2.64001000

H -0.08378000 5.18680700 -3.94084000

C -1.72462100 6.42805300 -1.64525300

H -2.26893200 7.36653400 -1.52350500

H -1.91870800 6.04953100 -2.65132600

H -2.11323500 5.69316900 -0.92759200

C -0.45061100 8.12432400 0.70677900

H -1.28636400 8.65829300 0.24664300

H -0.87402900 7.44459600 1.45717600

H 0.17964200 8.84651300 1.23048200

C 0.84170600 10.46319500 -5.39037300

H -0.14658500 10.09392600 -5.65515700

C 1.71466900 9.54695800 -4.90144500

C 1.26826600 8.16958800 -4.60498300

H 0.25401500 7.98782100 -4.96274400

C 1.01749600 11.90309500 -5.59681200

C 1.94167300 12.69037600 -4.87974700

C 0.17049800 12.55624500 -6.51271900

C 2.02414300 14.06295100 -5.09335700

H 2.58737300 12.22371900 -4.14643400

C 0.26038000 13.92858800 -6.73175000

H -0.56409100 11.96966700 -7.05844500

C 1.19216300 14.69009700 -6.02494400

H 2.74043600 14.64911100 -4.52440000

H -0.40097000 14.40442800 -7.45037500

H 1.26325300 15.76118800 -6.18990700

C 2.23399700 7.07991900 -5.04462300

H 3.19575200 7.13910000 -4.53045600

H 1.81739700 6.08471400 -4.90152900

H 2.44361100 7.19206600 -6.11798000

**INT-10 (in TFE)**

Rh 1.23441500 7.37895700 -2.17777000

C 4.82989400 10.86366800 -3.32085400

C 3.74406200 10.36841500 -2.59046900

C 3.79114300 9.06432400 -2.02443600

C 4.96588000 8.30508600 -2.23264600

C 6.03991400 8.81371100 -2.95736900

C 5.98243500 10.09927600 -3.50774000

H 4.76771200 11.86412000 -3.74199300

H 5.00609500 7.30516600 -1.80926700

H 6.92969500 8.20434900 -3.09651900

H 6.81924800 10.49859700 -4.07240300

O 2.79124700 8.60504100 -1.29027800

N 0.11528200 9.02701500 -2.75706300

C -0.48102900 9.90549600 -1.96266900

O -0.15601800 10.07689500 -0.75333800

C -1.59386600 10.76421500 -2.54272100

H -1.41038100 11.81713700 -2.30676700

H -1.70484200 10.64979200 -3.62395000

H -2.53868600 10.48418500 -2.06278300

C 0.73167400 6.42885700 -0.28183600

C -0.43040300 6.42813100 -1.11558200

C -0.12276000 5.69073500 -2.31904800

C 1.22455400 5.16698700 -2.17456300

C 1.75333500 5.61759200 -0.93552200

C 1.92562300 4.32936800 -3.19243900

H 1.63301200 3.27834200 -3.07900000

H 1.66221600 4.63853800 -4.20696600

H 3.01041800 4.39219800 -3.08416100

C 3.09832900 5.32128900 -0.35876800

H 3.02324900 4.52699000 0.39454300

H 3.80008700 4.98962000 -1.12708900

H 3.51574300 6.20365500 0.13271300

C -1.07873900 5.36627000 -3.42083600

H -0.55439900 5.18814000 -4.36190400

H -1.65157100 4.46186900 -3.17744200

H -1.79180000 6.17918800 -3.57827100

C -1.74774400 7.05780700 -0.80459200

H -1.64040600 7.88622400 -0.10327300

H -2.23292500 7.43177900 -1.70882100

H -2.41184200 6.30992200 -0.35377700

C 0.88496600 7.07948700 1.05306100

H 0.12319400 7.84450400 1.21194400

H 0.79930600 6.33466700 1.85426900

H 1.86634600 7.55355900 1.14361800

C 2.00570100 11.68676300 -1.22915700

H 1.06778200 12.22542000 -1.30644200

C 2.50463200 11.17811100 -2.44318300

C 1.68535700 11.40157100 -3.53849700

H 0.79249900 12.00171100 -3.37935400

C 2.55022300 11.64969600 0.09521700

C 3.85248300 11.19869000 0.42099800

C 1.70629100 12.10192100 1.14055800

C 4.28044200 11.20018600 1.74110000

H 4.52480100 10.87480000 -0.36086500

C 2.13582500 12.08487800 2.45884900

H 0.70352400 12.43737000 0.89509500

C 3.42526800 11.63266200 2.76145000

H 5.28349100 10.86243600 1.98142600

H 1.47548300 12.42143400 3.25149900

H 3.76565900 11.62059500 3.79253400

C 1.93724200 10.98277400 -4.93151500

H 2.76900900 10.28679400 -5.02357400

H 1.02874300 10.55275500 -5.36629100

H 2.15963700 11.88158600 -5.52682300

C 2.65824500 7.62400100 -6.34839000

H 3.65821400 7.95774800 -6.06355100

H 2.26502900 8.25446300 -7.14966500

H 2.73477400 6.59988900 -6.73308500

C 1.70850100 7.62493700 -5.16113200

O 2.28995500 7.54535800 -4.01270300

O 0.47557700 7.67699700 -5.35327500

H -0.24304400 8.96470900 -3.70438500

**INT-10 (in Methanol)**

Rh 1.23665300 7.35867500 -2.17177700

C 4.83510200 10.85162500 -3.24454200

C 3.73576800 10.35502600 -2.53559600

C 3.78359300 9.06106500 -1.94610900

C 4.97523100 8.31629000 -2.10567400

C 6.06301200 8.82710800 -2.80829500

C 6.00309500 10.10086100 -3.38479100

H 4.77120200 11.84356600 -3.68503500

H 5.01805300 7.32523100 -1.66207400

H 6.96523300 8.22860000 -2.90933300

H 6.84984900 10.50193700 -3.93319600

O 2.76928200 8.59497700 -1.23416800

N 0.11246800 9.00791500 -2.75312400

C -0.49653200 9.88642900 -1.96706200

O -0.18056500 10.06747000 -0.75705100

C -1.61035800 10.73453900 -2.56091500

H -1.43345300 11.79037400 -2.33288300

H -1.71349700 10.61050400 -3.64183900

H -2.55716600 10.45436700 -2.08498000

C 0.81344100 6.29094700 -0.30412300

C -0.39500600 6.40580200 -1.05632600

C -0.20026600 5.73815500 -2.32701000

C 1.12539500 5.15604900 -2.31029100

C 1.75938900 5.50220600 -1.08216900

C 1.72932400 4.36167100 -3.42069400

H 1.47146900 3.30205200 -3.30182900

H 1.35434400 4.68850200 -4.39319600

H 2.81837400 4.44352600 -3.42462600

C 3.12278500 5.09746300 -0.62474200

H 3.06552800 4.20225200 0.00751800

H 3.77566800 4.86820500 -1.47006400

H 3.59040400 5.88919200 -0.03426900

C -1.24665700 5.53398500 -3.37424900

H -0.80091900 5.36677600 -4.35624900

H -1.86656800 4.66144900 -3.13024900

H -1.90868500 6.40070600 -3.44566200

C -1.66511100 7.05323200 -0.61312900

H -1.48428900 7.83642900 0.12386600

H -2.20086300 7.49288300 -1.45720400

H -2.32022500 6.29919500 -0.15907900

C 1.07608300 6.83602200 1.05967500

H 0.37461500 7.63375300 1.31140400

H 0.97901200 6.04198400 1.81071100

H 2.09046000 7.23753000 1.13116000

C 1.97589300 11.71475500 -1.24582900

H 1.03593000 12.24498000 -1.35459400

C 2.48659600 11.15732000 -2.43233400

C 1.68296200 11.34340000 -3.54677900

H 0.78280700 11.93934300 -3.41762400

C 2.51446300 11.74851000 0.08125800

C 3.80290000 11.28463500 0.44350900

C 1.68542600 12.30418600 1.08820100

C 4.23068000 11.37364000 1.76056100

H 4.46538200 10.88136000 -0.30899900

C 2.11595200 12.37836500 2.40426800

H 0.69514900 12.65617500 0.81594300

C 3.39111700 11.91190600 2.74293900

H 5.22265500 11.02383400 2.02813200

H 1.46742500 12.79783500 3.16657500

H 3.73271200 11.97059600 3.77205500

C 1.96797900 10.90557400 -4.92713800

H 2.81030500 10.21966000 -4.99239400

H 1.07365500 10.46017700 -5.37498900

H 2.19205900 11.79966900 -5.52914600

C 2.72703100 7.57172200 -6.31383200

H 3.73085500 7.88424700 -6.01908700

H 2.35592800 8.21230800 -7.11784900

H 2.78454600 6.54745700 -6.70138500

C 1.76214400 7.58811100 -5.13912100

O 2.32768600 7.55325800 -3.98029600

O 0.53167200 7.60950000 -5.34979100

H -0.24357500 8.94237600 -3.70092600

**INT-10g (in Dioxane)**

Rh 1.10156800 8.47353700 -0.81071200

C -3.03135800 11.83095600 -1.90796900

C -2.51168500 10.54374200 -1.69626400

C -1.32364300 10.36255800 -0.92079800

C -0.68757000 11.53346800 -0.43122300

C -1.21507900 12.79331400 -0.66241600

C -2.40074300 12.95802500 -1.39559400

H -3.95062900 11.93800700 -2.47895400

H 0.21542100 11.39592400 0.14835400

H -0.70501300 13.66494900 -0.25950300

H -2.82026900 13.94560600 -1.55910300

O -0.90526400 9.15275900 -0.64723600

N 1.42087000 9.91974300 -2.21107900

C 0.82172500 10.10729500 -3.36978800

O 0.04428300 9.27333100 -3.92333700

C 1.04921900 11.44625300 -4.05959900

H 0.18181100 12.08685700 -3.86302300

H 1.94024600 11.96339700 -3.69414300

H 1.12806600 11.30277500 -5.14058100

C 0.57528600 6.38954700 -0.19272700

C 0.61463400 6.53035500 -1.64559300

C 1.95608600 6.87749200 -2.01610000

C 2.72441800 7.04681300 -0.81100500

C 1.86101900 6.69984100 0.31227300

C 4.18146700 7.36518100 -0.70913900

H 4.51908400 7.94879300 -1.56761000

H 4.39264300 7.94951300 0.18952700

H 4.78252800 6.44715000 -0.66320200

C 2.29097500 6.76614800 1.74143800

H 3.11243700 6.06651700 1.93349800

H 2.63669200 7.77620100 1.98250100

H 1.47070000 6.52519600 2.42065600

C 2.45215400 7.05559800 -3.41359800

H 2.78877200 6.09215700 -3.81765500

H 1.66146600 7.45155300 -4.05249200

H 3.29448000 7.74945300 -3.44723000

C -0.51928500 6.27491700 -2.58583000

H -0.40877700 5.30469500 -3.08771900

H -1.46880300 6.26916100 -2.04697100

H -0.55358200 7.06775700 -3.33702900

C -0.65762800 6.06160800 0.58558300

H -0.46326200 6.04648000 1.65983200

H -1.42688800 6.81301500 0.39648100

H -1.05589200 5.08224100 0.29802900

C -3.57431800 8.20512000 -1.72502700

H -3.85632900 7.39889200 -2.40015000

C -3.11846800 9.36912400 -2.36114900

C -3.13158900 9.29451100 -3.75503200

H -3.52570800 8.37497400 -4.18738000

C -3.74201200 7.87692200 -0.34545600

C -3.48128800 8.75966600 0.73232000

C -4.18600600 6.56212700 -0.04764600

C -3.66206000 8.33442700 2.04086700

H -3.15180200 9.76819300 0.53477500

C -4.34693000 6.14061400 1.26226300

H -4.38923100 5.87798400 -0.86675700

C -4.07953900 7.02780800 2.31164100

H -3.45850300 9.01906400 2.85772300

H -4.67552000 5.12816200 1.47319500

H -4.19656000 6.69962100 3.34009300

C -2.53166600 10.23324400 -4.68449000

H -2.52715000 11.26861600 -4.34409100

H -1.43868000 9.91340700 -4.66173100

H -2.91017800 10.13385200 -5.70336600

C 3.07778200 11.25283800 1.99054900

H 2.18637400 11.65745600 2.47934900

H 3.48812900 10.48493400 2.65588600

H 3.81806800 12.04380400 1.85786800

C 2.70923400 10.62728500 0.64736900

O 1.72309900 9.78968800 0.73406500

O 3.33566800 10.93621300 -0.37629000

H 2.07116000 10.62934400 -1.88436500

**INT-10f (in Methanol)**

C -1.80616100 -2.07026100 0.15043700

C -0.43912300 -2.52909900 0.18218400

C 0.15764000 -2.28167000 -1.09139200

C -0.85498700 -1.65340800 -1.91899100

C -2.07770400 -1.58387900 -1.16318600

C -0.70305900 -1.31560000 -3.36183500

H -1.33486900 -0.46974300 -3.64125800

H 0.33321500 -1.07592400 -3.60618800

H -1.00373100 -2.17831700 -3.96965200

C -3.39691300 -1.19924700 -1.73337400

H -3.29611300 -0.35458900 -2.41801200

H -3.79179000 -2.04772000 -2.30704100

H -4.11345900 -0.93698100 -0.95705400

C -2.79474900 -2.31008000 1.24084900

H -3.72715800 -1.77761300 1.05695200

H -3.01461700 -3.38413100 1.29529400

H -2.40877900 -2.00008900 2.21324700

C 0.15573600 -3.27949400 1.32105800

H -0.13851100 -2.85104100 2.28275600

H -0.22196300 -4.30980400 1.29644300

H 1.24262200 -3.32101800 1.26309900

C 1.50446100 -2.70791000 -1.56686000

H 1.39947100 -3.59282900 -2.20707500

H 1.97978000 -1.92485300 -2.16242200

H 2.16666600 -2.96160600 -0.73943800

Rh -0.37078300 -0.23906000 -0.33618100

C 2.01591200 3.32281800 0.74676700

C 1.44422200 2.16258900 0.22449200

C 1.41320900 1.93829000 -1.17136300

C 1.97824400 2.90979300 -2.02126800

C 2.55661000 4.05929900 -1.48463400

C 2.58148300 4.27684100 -0.10272800

H 2.02332800 3.47525800 1.82259500

H 1.96076100 2.74110300 -3.09425100

H 2.98769500 4.79774200 -2.15578200

H 3.02863500 5.17716600 0.30652200

O 0.90521500 0.81068800 -1.65618100

N -1.60759400 1.31347700 -0.94025600

C -2.74392800 1.49307800 -0.32285000

O -3.27039200 0.86852600 0.67225000

C -3.58735600 2.63337600 -0.90311400

H -3.78463000 3.36837000 -0.11405600

H -3.10647900 3.13454000 -1.74578300

H -4.55318200 2.23029300 -1.23025500

C 2.71122000 -0.69345900 0.80055200

C 3.25567300 -1.66079500 1.67375300

C 4.54057800 -2.16286700 1.49016100

C 5.31635600 -1.71777700 0.41735200

C 4.79318500 -0.76374000 -0.45754900

C 3.50957700 -0.25396300 -0.27343700

H 2.66327400 -2.00496200 2.51560000

H 4.93463400 -2.89979800 2.18365600

H 6.31752200 -2.10993100 0.26500000

H 5.38605500 -0.41387300 -1.29768100

H 3.12635200 0.45663800 -0.98653300

C 1.35358800 -0.23430600 1.12377600

C 0.83351200 1.10871800 1.07848400

H 0.90663500 -0.82630200 1.91702000

H -0.48523700 3.25302000 2.68997400

C -0.32865700 1.34606800 1.80608300

C -0.99215000 2.67226600 1.90570100

H -0.66679000 0.56716700 2.48281000

H -2.03497900 2.56651400 2.19925700

H -0.91443900 3.23800700 0.97626600

C -4.25345100 0.14790700 3.62668700

H -4.27061300 -0.01001600 4.71033800

H -4.84980400 1.04422600 3.40262300

H -4.74469100 -0.71447600 3.15203200

O -2.90135500 0.29281800 3.21878700

H -2.91964000 0.48141700 2.24562300

**TS-1(in TFE)**

C -0.59328800 -2.40781800 0.10392500

C -0.24805500 -1.88130200 -1.20665500

C -1.36331800 -1.07461100 -1.65852000

C -2.33127400 -1.02105400 -0.61095400

C -1.84357400 -1.84716400 0.48307400

C -3.63862700 -0.29950500 -0.62663400

H -3.85730600 0.13599200 0.35218800

H -3.64033900 0.50361900 -1.36521900

H -4.45501200 -0.99129300 -0.87029600

C -2.57523700 -2.08269300 1.76280900

H -3.03857600 -1.16176600 2.12683200

H -3.37647900 -2.81586300 1.60808400

H -1.91011100 -2.46755800 2.53870800

C 0.19069900 -3.41616500 0.87758300

H 0.12424900 -3.24200900 1.95400900

H -0.21201000 -4.41714100 0.67731300

H 1.24271500 -3.41479600 0.59143600

C 0.95570800 -2.25313100 -2.01144900

H 1.81730700 -2.44238400 -1.36756800

H 0.76210200 -3.16180900 -2.59479200

H 1.22288900 -1.45635300 -2.70913400

C -1.46527100 -0.39829400 -2.98590200

H -2.05578600 -1.02234500 -3.66794700

H -1.95589800 0.57284800 -2.90247400

H -0.48053800 -0.25229400 -3.43394600

Rh -0.40296100 -0.19066200 0.11265400

C -0.46129900 1.75840100 2.43757100

O -1.22273400 1.11481400 1.66103200

O 0.81476900 1.65561500 2.41235500

C -1.06643800 2.72104500 3.42302100

H -0.52052000 2.68982800 4.36860400

H -0.97478000 3.73392500 3.01429400

H -2.12289400 2.50070600 3.58205200

C 2.45653900 -1.03897200 1.34734200

C 1.70539700 -0.06511800 0.65423400

C 2.36132000 0.60845700 -0.40282000

C 3.69174200 0.35387300 -0.74757000

C 4.39026400 -0.61521200 -0.03209200

C 3.77828100 -1.31927100 1.01670900

H 1.98858100 -1.56139300 2.17709700

H 1.12958100 0.77764100 1.52863700

H 4.15216800 0.89277400 -1.56973300

H 5.42263200 -0.82628400 -0.29621200

H 4.33838700 -2.06748400 1.56937900

O 1.69368700 1.52349300 -1.15705200

N 0.28105000 1.49934500 -0.86331100

C -0.38513600 2.52272500 -1.39960800

O -1.63548800 2.59665600 -1.26513700

C 0.38285100 3.57823100 -2.16762400

H 1.18623200 4.00501700 -1.56015900

H 0.84226200 3.14775400 -3.06367500

H -0.31253000 4.36589500 -2.46197800

**TS-1(in Methanol)**

C -0.62901000 -2.39513500 0.12314400

C -0.25227200 -1.89220300 -1.18795600

C -1.34654300 -1.07602200 -1.67117300

C -2.33664900 -0.99905400 -0.64530300

C -1.88023300 -1.81501100 0.46873600

C -3.64039900 -0.27321000 -0.70164200

H -3.86019400 0.21613200 0.25126500

H -3.63900300 0.48707500 -1.48423100

H -4.45896900 -0.97410200 -0.90908000

C -2.63950700 -2.02883300 1.73594400

H -3.15855100 -1.11761000 2.04416200

H -3.39821600 -2.80804100 1.59110500

H -1.98182700 -2.34658000 2.54803600

C 0.12437500 -3.40381100 0.92629900

H 0.05196600 -3.20415800 1.99804500

H -0.29997700 -4.39954100 0.74502600

H 1.17866500 -3.43291200 0.65017700

C 0.95836200 -2.29576600 -1.96637600

H 1.80735300 -2.48542300 -1.30613900

H 0.75936500 -3.21295000 -2.53453000

H 1.24695600 -1.51730400 -2.67598800

C -1.41020000 -0.41305200 -3.00744900

H -1.96870900 -1.04956900 -3.70474700

H -1.91500000 0.55254000 -2.95081700

H -0.41182700 -0.25889300 -3.42142900

Rh -0.40919200 -0.18106700 0.10628600

C -0.45749800 1.76533800 2.43654300

O -1.22060200 1.13412100 1.65179200

O 0.81830900 1.65761400 2.41146000

C -1.05711600 2.70707300 3.44554700

H -0.62778800 2.51628200 4.43284400

H -0.79898600 3.73389300 3.16441400

H -2.14223300 2.60426100 3.47845800

C 2.42939900 -1.05782000 1.36295900

C 1.69661600 -0.07728900 0.65949400

C 2.36710100 0.57727100 -0.40027400

C 3.69448400 0.29796700 -0.73806300

C 4.37461800 -0.67667500 -0.01245100

C 3.74763700 -1.36212700 1.03976900

H 1.94981700 -1.56627400 2.19469400

H 1.12852900 0.77912900 1.52846300

H 4.16701100 0.82195800 -1.56299600

H 5.40415600 -0.90701700 -0.27159600

H 4.29324800 -2.11525900 1.60025700

O 1.71719100 1.49656300 -1.16370800

N 0.30264300 1.49900400 -0.87367400

C -0.33752200 2.54445500 -1.39871800

O -1.58578800 2.64996500 -1.26375200

C 0.45721700 3.58880700 -2.15535200

H 1.27115100 3.98844000 -1.54362500

H 0.90530100 3.15755000 -3.05671900

H -0.21713800 4.39783100 -2.44061700

**TS-1(in Dioxane)**

C -0.63839500 -2.40010500 0.02273200

C -0.20043100 -1.85752400 -1.25433100

C -1.24401100 -0.98792600 -1.73513000

C -2.28472500 -0.93463600 -0.74931100

C -1.90112500 -1.81815900 0.33387800

C -3.55222400 -0.15030900 -0.85655700

H -4.05543200 -0.08394200 0.11058900

H -3.33302200 0.86565800 -1.19212400

H -4.24504800 -0.62314700 -1.56441600

C -2.71177200 -2.05590500 1.56549200

H -3.08768100 -1.11257300 1.96922200

H -3.57398600 -2.69434100 1.33862900

H -2.12374400 -2.54716300 2.34347100

C 0.06935000 -3.45811700 0.80708000

H -0.07074000 -3.32813200 1.88290700

H -0.32245400 -4.44755900 0.53881500

H 1.14047900 -3.45856700 0.60135300

C 1.03846500 -2.25765100 -1.99090700

H 1.87461200 -2.41410600 -1.30578000

H 0.87544900 -3.19074200 -2.54480600

H 1.33713600 -1.49138900 -2.70917900

C -1.25245000 -0.24769700 -3.03186200

H -1.79955800 -0.82817600 -3.78541200

H -1.74043400 0.72167800 -2.91853700

H -0.23984300 -0.08411500 -3.40618200

Rh -0.38716400 -0.20074700 0.12801900

C -0.44260200 1.52512700 2.58752800

O -1.18249300 0.87108600 1.79232900

O 0.82586000 1.51347800 2.55799300

C -1.12458500 2.39536900 3.61591000

H -1.28602000 3.38440800 3.17229400

H -2.09796100 1.98329700 3.88744800

H -0.49617900 2.51433400 4.50014700

C 2.48820600 -1.10043500 1.24299800

C 1.71595900 -0.06648800 0.66480800

C 2.33944100 0.71209600 -0.34255600

C 3.66790900 0.49745600 -0.73359000

C 4.38234600 -0.53246900 -0.13405300

C 3.79836800 -1.34316600 0.85475300

H 2.04482600 -1.69265600 2.03881400

H 1.18182500 0.68111000 1.57897300

H 4.11032000 1.11710100 -1.50669900

H 5.40989600 -0.71004600 -0.43873400

H 4.37748300 -2.13352200 1.32226400

O 1.65285900 1.67673300 -0.98077800

N 0.23178400 1.59317700 -0.68816500

C -0.47306000 2.55809600 -1.31200200

O -1.71681300 2.54947200 -1.29167600

C 0.29701700 3.65486400 -2.02881600

H 1.04130900 4.11793600 -1.37547600

H 0.83216700 3.25018400 -2.89421300

H -0.41887600 4.40519200 -2.36640900

**TS-2(in TFE)**

Rh 0.46383300 -0.50194800 -0.02004100

C 2.65624800 1.64407200 -0.78802700

C 1.36816800 1.41418400 -0.27579200

C 0.92224700 2.28869300 0.74186700

C 1.67744200 3.37886800 1.16863600

C 2.92979500 3.60870100 0.59467400

C 3.42983400 2.72625400 -0.36713700

H 3.04794600 0.98219300 -1.55027300

H 1.28924500 4.01722800 1.95659400

H 3.51959800 4.46107500 0.91830600

H 4.41568500 2.88230600 -0.79497500

O -0.27473000 2.07922600 1.35164300

N -0.64492000 0.67563200 1.30020500

C -1.74985000 0.44257300 2.01242500

O -2.27574500 -0.70094800 2.01387100

C -2.34731100 1.56299900 2.84120500

H -1.61943600 1.95671300 3.55763200

H -2.66702300 2.39566300 2.20907200

H -3.21044800 1.16640900 3.37843500

C 1.13259500 -2.06467000 1.45934100

C 0.20907500 -2.66950600 0.50710400

C 0.81627400 -2.59010400 -0.78195400

C 2.07677500 -1.91614600 -0.64580500

C 2.29295400 -1.63675000 0.75801200

C 3.06832700 -1.73122300 -1.75103400

H 3.51454800 -2.69761800 -2.01806700

H 2.59813200 -1.33027900 -2.65361500

H 3.87921200 -1.06308500 -1.45701900

C 3.52041600 -1.01692800 1.34417900

H 4.31525100 -1.76420100 1.46148200

H 3.90869400 -0.21916900 0.70546400

H 3.31722900 -0.58924600 2.32898000

C 0.27689300 -3.17396800 -2.04691900

H 0.57441900 -2.58731900 -2.92025800

H 0.66792600 -4.18999700 -2.18626600

H -0.81353300 -3.23847200 -2.02663100

C -1.03343100 -3.43754800 0.83048500

H -1.84034100 -3.21020400 0.12833300

H -0.83288500 -4.51525900 0.77154100

H -1.39136300 -3.21602400 1.83600000

C 0.90184200 -1.97093700 2.93335100

H -0.15525300 -1.81783900 3.15807400

H 1.22128400 -2.89796300 3.42601500

H 1.46873300 -1.14691400 3.37286800

C -0.90261900 0.04596200 -1.57424100

H -0.78095200 -0.78064400 -2.27099800

C 0.09810600 1.08125100 -1.70955900

C -0.13497900 2.44578400 -2.27896200

C 0.71699200 1.42518600 -3.02308800

H 0.38476900 3.29743600 -1.85310600

H -1.13976900 2.65202900 -2.63605000

H 0.29345800 0.92866400 -3.89207000

H 1.77520200 1.63979000 -3.09847100

C -2.31175800 0.26814800 -1.21349100

C -3.22004800 -0.78890700 -1.41522000

C -2.80276400 1.46554100 -0.66253800

C -4.56147100 -0.66631700 -1.06148400

H -2.85598500 -1.71835100 -1.84595700

C -4.14648800 1.58930500 -0.31321000

H -2.12494400 2.29082700 -0.48175000

C -5.03209600 0.52515400 -0.50262300

H -5.23996200 -1.49989900 -1.22095600

H -4.50366900 2.52127700 0.11641900

H -6.07704100 0.62487500 -0.22363500

**TS-2(in Methanol)**

Rh 0.46524200 -0.50138300 -0.01965500

C 2.64546100 1.65039600 -0.80217200

C 1.36198800 1.41725700 -0.27995900

C 0.92204400 2.28957200 0.74209900

C 1.67860500 3.38107800 1.16369000

C 2.92592400 3.61401100 0.58014800

C 3.42027800 2.73359200 -0.38644700

H 3.03228200 0.99002300 -1.56814800

H 1.29601500 4.01861100 1.95504700

H 3.51638700 4.46740800 0.89996000

H 4.40239900 2.89196300 -0.82200500

O -0.26915700 2.07709000 1.36170500

N -0.64038600 0.67327900 1.30753300

C -1.73899400 0.43762600 2.02818100

O -2.26148400 -0.70799900 2.03542000

C -2.33550200 1.55800600 2.85793500

H -1.60837800 1.94774300 3.57732100

H -2.65132500 2.39323400 2.22722700

H -3.20092500 1.16375600 3.39314500

C 1.15367400 -2.06043700 1.45457600

C 0.22504900 -2.67036200 0.51051000

C 0.82062800 -2.58843200 -0.78373800

C 2.07931000 -1.90872700 -0.65863700

C 2.30599400 -1.62765800 0.74326000

C 3.06107600 -1.72167800 -1.77207300

H 3.50820900 -2.68714900 -2.04109600

H 2.58217700 -1.32393600 -2.67142600

H 3.87223500 -1.05055400 -1.48583600

C 3.53547900 -1.00302300 1.31975100

H 4.32867000 -1.75069800 1.44569200

H 3.92491900 -0.21459000 0.67047000

H 3.33495900 -0.56225400 2.29939000

C 0.27285900 -3.17506300 -2.04379600

H 0.56283000 -2.58917200 -2.92017100

H 0.66467900 -4.19062800 -2.18452600

H -0.81727900 -3.24146600 -2.01542400

C -1.00922100 -3.44678400 0.84561900

H -1.82327500 -3.22939400 0.14846400

H -0.80034600 -4.52306700 0.78896600

H -1.36088300 -3.22571100 1.85341600

C 0.93520600 -1.96732400 2.93046800

H -0.12097400 -1.82154300 3.16416900

H 1.26505700 -2.89184800 3.42100400

H 1.50001200 -1.13912700 3.36483900

C -0.91340200 0.04298300 -1.56617300

H -0.79089200 -0.78276200 -2.26378100

C 0.08193900 1.08276000 -1.70622500

C -0.16023500 2.44756000 -2.27063200

C 0.69118500 1.43227500 -3.02274600

H 0.35876700 3.30035800 -1.84616800

H -1.16808500 2.65071900 -2.62084700

H 0.26400600 0.93605400 -3.89014700

H 1.74805200 1.65126100 -3.10446900

C -2.32293000 0.26105100 -1.20425600

C -3.23075000 -0.79427100 -1.41744800

C -2.81618100 1.45454000 -0.64662900

C -4.57399100 -0.67339200 -1.06965600

H -2.86521200 -1.72056400 -1.85379400

C -4.16145800 1.57643000 -0.30256600

H -2.13920900 2.27883300 -0.45887700

C -5.04689500 0.51433800 -0.50468500

H -5.25218100 -1.50526500 -1.23918000

H -4.52030000 2.50555400 0.13187800

H -6.09331200 0.61283100 -0.23074900

**TS-2(in Dioxane)**

Rh 0.48891600 -0.49586400 -0.04473300

C 2.60895400 1.71917300 -0.79003600

C 1.33265400 1.44371800 -0.27226200

C 0.86122600 2.28909500 0.76139300

C 1.58832700 3.39939700 1.18998400

C 2.82934200 3.67235200 0.61346000

C 3.35313500 2.81777000 -0.36047600

H 3.01239500 1.07892200 -1.56504000

H 1.18750900 4.01740600 1.98708200

H 3.39503900 4.53797300 0.94459100

H 4.33124500 3.01008500 -0.79100700

O -0.31593700 2.02970300 1.36987300

N -0.62668000 0.60733800 1.30960200

C -1.70077500 0.30364500 2.06224200

O -2.14137100 -0.85951800 2.10636900

C -2.33536600 1.40865400 2.89084000

H -1.62503300 1.81025200 3.62048300

H -2.66262500 2.24142400 2.26414900

H -3.19386000 0.98405400 3.41238000

C 1.09037600 -2.09314800 1.41544300

C 0.24843800 -2.67699100 0.38194700

C 0.94978000 -2.54228600 -0.85779400

C 2.18503000 -1.85540800 -0.60389000

C 2.29027000 -1.61468200 0.81607700

C 3.25694300 -1.61640700 -1.62127600

H 3.76968900 -2.55717400 -1.85938800

H 2.85090600 -1.22275000 -2.55796400

H 4.01065200 -0.91806700 -1.25360400

C 3.44827300 -0.96802200 1.50703800

H 4.28102300 -1.67153900 1.63244900

H 3.81912900 -0.10774800 0.94206400

H 3.16362300 -0.60983500 2.49899500

C 0.52768400 -3.07705600 -2.18902200

H 0.84470800 -2.42161700 -3.00513400

H 0.98076600 -4.06062600 -2.36670300

H -0.55558500 -3.20023300 -2.25195800

C -1.03986300 -3.39910300 0.61246100

H -1.57527300 -3.55943900 -0.32668100

H -0.85961200 -4.38343100 1.06475700

H -1.68191000 -2.81132800 1.27050700

C 0.75112800 -2.06254600 2.87054500

H -0.32555600 -1.95775300 3.00826500

H 1.08185600 -2.99169900 3.35254600

H 1.24385000 -1.22974200 3.37773400

C -0.92557000 0.03708900 -1.54886400

H -0.80351400 -0.77707800 -2.26124600

C 0.05719100 1.09180100 -1.68977300

C -0.21709400 2.45677400 -2.23992900

C 0.64446100 1.46804800 -3.01093500

H 0.28880800 3.31511300 -1.81153200

H -1.23247800 2.64194900 -2.57731400

H 0.21912300 0.97706300 -3.88219000

H 1.69585200 1.71023000 -3.09982600

C -2.33605500 0.24390300 -1.18082800

C -3.24673400 -0.79855600 -1.42697800

C -2.82744500 1.42669000 -0.60178400

C -4.59406900 -0.67399000 -1.10211200

H -2.88184800 -1.72151900 -1.87075000

C -4.17685700 1.55350600 -0.28111900

H -2.14393800 2.23586400 -0.37737600

C -5.06733100 0.50614000 -0.52561300

H -5.27477200 -1.49795400 -1.29642100

H -4.53505000 2.47484100 0.16987000

H -6.11740900 0.60800300 -0.26835300

**TS-2’(in TFE)**

Rh 0.93916500 -0.02531100 -0.17703200

C -0.95036400 2.54525300 -0.35759700

C -0.80218900 1.22050500 0.08918000

C -1.36975300 0.89127000 1.34058600

C -2.07986900 1.82648800 2.09423900

C -2.23002200 3.12436200 1.60599200

C -1.65046800 3.49260200 0.38587300

H -0.54112000 2.82085700 -1.32431200

H -2.49210400 1.53293800 3.05477000

H -2.78949500 3.85124200 2.18722200

H -1.75688300 4.50638500 0.01184900

O -1.23391700 -0.35243100 1.87128000

N -0.17940100 -1.10248100 1.21465500

C -0.09921000 -2.34846800 1.67795300

O 0.78534900 -3.13234300 1.23739500

C -1.07486700 -2.80672600 2.74361400

H -0.96135300 -2.21607800 3.65844500

H -2.11030000 -2.69214300 2.40891400

H -0.87493500 -3.85663000 2.96386300

C 3.07590100 -0.65008800 -0.06056300

C 2.94591700 0.21194400 -1.19278700

C 2.49443100 1.49105300 -0.72724700

C 2.44098900 1.46103200 0.72087700

C 2.77097000 0.14472200 1.12698200

C 2.08235200 2.61646300 1.60033500

H 2.94920800 3.27017900 1.75943300

H 1.28960100 3.22249200 1.15394700

H 1.73591000 2.27993200 2.58052000

C 2.82658200 -0.36879800 2.52982700

H 3.85561100 -0.32261900 2.90793500

H 2.19668900 0.22316300 3.19785500

H 2.49935400 -1.40928900 2.58163700

C 2.32038900 2.70215900 -1.58718800

H 1.71619400 3.46465700 -1.09321000

H 3.29952200 3.14582600 -1.81069600

H 1.85010300 2.45163300 -2.54207400

C 3.32804200 -0.10243000 -2.60190300

H 2.62945900 0.33306900 -3.32102800

H 4.32077400 0.31467900 -2.81490400

H 3.38099000 -1.17866400 -2.77691100

C 3.64696100 -2.03344600 -0.06835400

H 3.34787200 -2.58218900 -0.96527600

H 4.74451400 -1.99400800 -0.05286500

H 3.31884500 -2.60603500 0.79987800

C -1.16951200 0.04978400 -1.45274400

C -0.18584900 -1.00577900 -1.61400200

C 0.26863500 -1.56548000 -2.92426300

C -0.40683800 -2.45140200 -1.88498200

H 1.32644900 -1.72000000 -3.10434300

H -0.30235700 -1.30608100 -3.81492600

H -1.41770900 -2.79336100 -2.09565700

H 0.21165000 -3.17612500 -1.36369300

C -2.59912300 -0.22954100 -1.12864900

C -3.57077300 0.68353200 -1.56779900

C -3.00799500 -1.35521400 -0.39789700

C -4.92011200 0.47517200 -1.29135900

H -3.25939800 1.56545600 -2.12079300

C -4.35860900 -1.55909000 -0.11660900

H -2.26878600 -2.05891700 -0.04077100

C -5.31910000 -0.64750600 -0.56062900

H -5.65959400 1.18873100 -1.64331000

H -4.66065900 -2.43364100 0.45268100

H -6.36998500 -0.80991000 -0.33937800

H -1.06519800 0.84475600 -2.18568600

**TS-2’(in Methanol)**

Rh 0.93903400 -0.02763400 -0.17761700

C -0.95062500 2.54434700 -0.35719700

C -0.80144400 1.21999800 0.09053200

C -1.36635600 0.89195900 1.34342000

C -2.07541300 1.82816600 2.09718800

C -2.22693200 3.12543900 1.60784800

C -1.64966000 3.49244700 0.38630600

H -0.54330900 2.81911000 -1.32492800

H -2.48580000 1.53605500 3.05896600

H -2.78563700 3.85271800 2.18936400

H -1.75686100 4.50576900 0.01122200

O -1.22828700 -0.35000600 1.87688500

N -0.18009200 -1.10491000 1.21471100

C -0.10054700 -2.34976400 1.68119900

O 0.77912600 -3.13792900 1.23764000

C -1.07235000 -2.80215800 2.75310400

H -0.95345000 -2.20865400 3.66536600

H -2.10901600 -2.68695700 2.42251800

H -0.87462800 -3.85157500 2.97740000

C 3.07711500 -0.64947700 -0.06003900

C 2.94557000 0.21052000 -1.19362600

C 2.49232200 1.48970000 -0.73011200

C 2.43921800 1.46177600 0.71816100

C 2.77103600 0.14668700 1.12636400

C 2.08100700 2.61829700 1.59627700

H 2.94923200 3.26987400 1.75692700

H 1.29093500 3.22622600 1.14784400

H 1.73186100 2.28318900 2.57601300

C 2.82802900 -0.36373100 2.53031300

H 3.85587500 -0.30814100 2.91046200

H 2.19201600 0.22425000 3.19611900

H 2.50970300 -1.40688100 2.58399600

C 2.31694500 2.69920100 -1.59198200

H 1.71442700 3.46311000 -1.09816700

H 3.29590900 3.14170600 -1.81862800

H 1.84427200 2.44713300 -2.54528600

C 3.32845700 -0.10585800 -2.60203000

H 2.63130800 0.33009900 -3.32225100

H 4.32203800 0.30963300 -2.81441800

H 3.38014900 -1.18236200 -2.77596400

C 3.65507500 -2.03000300 -0.06609600

H 3.35449700 -2.58386000 -0.95941700

H 4.75247500 -1.98431900 -0.05668700

H 3.33609700 -2.60120200 0.80649200

C -1.17012700 0.05011000 -1.45245400

C -0.18729600 -1.00590200 -1.61617400

C 0.26452700 -1.56147700 -2.92913000

C -0.41288200 -2.44986500 -1.89324800

H 1.32176200 -1.71860700 -3.11054400

H -0.30612700 -1.29647300 -3.81837000

H -1.42519900 -2.78737200 -2.10423000

H 0.20353900 -3.17994600 -1.37709900

C -2.59965900 -0.22907500 -1.12793500

C -3.57160900 0.68292200 -1.56869500

C -3.00832900 -1.35410700 -0.39602300

C -4.92101000 0.47422300 -1.29241700

H -3.26058200 1.56405800 -2.12315000

C -4.35891600 -1.55821200 -0.11474500

H -2.26888700 -2.05713700 -0.03796800

C -5.31972300 -0.64759800 -0.56022700

H -5.66072700 1.18692200 -1.64568800

H -4.66070400 -2.43220900 0.45558600

H -6.37061800 -0.81017900 -0.33902900

H -1.06567600 0.84598900 -2.18433100

**TS-2’(in Dioxane)**

Rh 0.93635500 0.00865500 -0.18200400

C -0.97149700 2.55538000 -0.33072400

C -0.81277100 1.22586600 0.09906000

C -1.37299800 0.87367000 1.35127100

C -2.09467200 1.79851700 2.11007800

C -2.25282200 3.09895300 1.63789200

C -1.67426700 3.48983700 0.42376800

H -0.56316000 2.84470500 -1.29467300

H -2.50723700 1.49128300 3.06561300

H -2.81913000 3.81411900 2.22693400

H -1.78763200 4.50795900 0.06416700

O -1.21074300 -0.36056500 1.87049500

N -0.15171600 -1.09248800 1.19190900

C -0.03845100 -2.35157000 1.64391700

O 0.85115000 -3.11067900 1.20708900

C -1.01331700 -2.81966200 2.71280500

H -0.87964700 -2.25057400 3.63840900

H -2.05239500 -2.68060300 2.40074600

H -0.82201500 -3.87557400 2.90704900

C 3.05879000 -0.65382200 -0.22352000

C 2.90537400 0.33897700 -1.24325900

C 2.50525300 1.56498200 -0.61162700

C 2.48956700 1.36737200 0.81821700

C 2.80256600 0.00032300 1.05284200

C 2.15543500 2.41102300 1.83732600

H 3.00721900 3.07715400 2.02504600

H 1.31519500 3.03049100 1.50994500

H 1.87456700 1.95646700 2.79033200

C 2.88928800 -0.68402200 2.37855700

H 3.92779600 -0.68522400 2.73385800

H 2.27880100 -0.18107600 3.13176900

H 2.55268700 -1.71891000 2.29952600

C 2.32670100 2.87498100 -1.31255100

H 1.70521300 3.55979400 -0.73294900

H 3.30119400 3.35791100 -1.46350700

H 1.87128300 2.74783300 -2.29874100

C 3.21189400 0.19526000 -2.69997000

H 2.43938700 0.64790400 -3.32753500

H 4.16100400 0.69321100 -2.93591500

H 3.31457400 -0.85085200 -2.99231000

C 3.51893800 -2.06795000 -0.38692600

H 3.42465900 -2.39774000 -1.42498000

H 4.57266700 -2.17787300 -0.09857800

H 2.90928200 -2.73218900 0.22943100

C -1.18239100 0.06595800 -1.44224700

C -0.19264300 -0.98543600 -1.59020000

C 0.29065700 -1.59290400 -2.86841700

C -0.37161300 -2.44473100 -1.79351700

H 1.35482300 -1.71988400 -3.03072900

H -0.27644600 -1.39841400 -3.77799700

H -1.36827700 -2.82520600 -2.00582600

H 0.25384400 -3.10784500 -1.20382700

C -2.61263100 -0.22031200 -1.12732300

C -3.59050300 0.67712200 -1.58134400

C -3.01352100 -1.34252900 -0.38807900

C -4.93889200 0.45502200 -1.31574800

H -3.28529700 1.56037400 -2.13563700

C -4.36314800 -1.56007700 -0.11815900

H -2.26513400 -2.02740800 -0.01508200

C -5.33014300 -0.66585000 -0.58014400

H -5.68359400 1.15720000 -1.67893300

H -4.65956100 -2.43182900 0.45802700

H -6.38083800 -0.83943400 -0.36727500

H -1.08014700 0.85586000 -2.18208000

**TS-3 (in TFE)**

Rh 2.89773800 8.51283500 -2.29903400

C 1.57783700 12.53688300 -4.30033900

C 2.04221900 11.61891700 -3.34685100

C 1.99385200 11.99962100 -1.99630100

C 1.54566800 13.26850600 -1.62223700

C 1.10081600 14.16722100 -2.58954900

C 1.09795100 13.79262200 -3.93451500

H 1.60766200 12.25546300 -5.34902200

H 1.54239300 13.52814400 -0.56905300

H 0.74644300 15.14888400 -2.29002700

H 0.73978500 14.47828100 -4.69601900

O 2.41672000 11.16956600 -0.97407100

N 1.84549400 9.84546900 -1.10172000

C 0.75440000 9.68250600 -0.33952500

O 0.13724000 8.58836900 -0.33949200

C 0.25470300 10.82985800 0.51746300

H 1.05269300 11.26760400 1.12193000

H -0.16072900 11.62569000 -0.10906800

H -0.53377700 10.44872600 1.16869700

C 3.41400500 7.05858300 -0.65619400

C 2.46396000 6.44896200 -1.58545100

C 3.12217600 6.32921200 -2.84956600

C 4.42440800 6.90384600 -2.72800400

C 4.62451000 7.30874400 -1.34762700

C 5.47151600 6.90011600 -3.79777400

H 6.01252500 5.94522900 -3.79120700

H 5.03128000 7.02205400 -4.79083800

H 6.21419300 7.68909000 -3.65228400

C 5.89211500 7.87766900 -0.79518200

H 6.63915300 7.08937000 -0.63637800

H 6.33818700 8.60773800 -1.47944900

H 5.72282900 8.37449200 0.16316600

C 2.57488500 5.65059100 -4.06265100

H 2.92465600 6.12239000 -4.98458600

H 2.90174300 4.60286900 -4.08582800

H 1.48322200 5.65643100 -4.06396100

C 1.16959400 5.78812100 -1.23017600

H 0.43385200 5.89935500 -2.03195000

H 1.32889500 4.71293800 -1.07143000

H 0.74026100 6.20752900 -0.32149600

C 3.14751100 7.30079900 0.79530200

H 2.10677700 7.58373500 0.96203200

H 3.34321100 6.38892700 1.37395400

H 3.78701900 8.09220000 1.19378800

C 1.80066400 9.11292500 -4.00793700

H 2.18495300 8.46797000 -4.79795200

C 2.60323600 10.32912100 -3.87222000

C 3.85028300 10.52896400 -4.66451700

C 4.29884400 10.17691000 -3.27595800

H 4.04337800 11.54037700 -5.01162800

H 4.05248200 9.76631200 -5.41418600

H 4.98494400 9.34630800 -3.21469700

H 4.50730000 10.99730000 -2.60007800

C 0.32282600 9.03065200 -3.95120200

C -0.27437200 7.95841600 -4.64849600

C -0.53886800 9.90366700 -3.25753500

C -1.65080000 7.74440900 -4.62780700

H 0.36019000 7.28571000 -5.21769600

C -1.91627000 9.69219300 -3.24179600

H -0.14002400 10.74427400 -2.71205800

C -2.48518200 8.61055100 -3.91783600

H -2.07131700 6.90425800 -5.17394100

H -2.54970700 10.38240700 -2.69083600

H -3.55927400 8.45070400 -3.89867100

**TS-3 (in Methanol)**

Rh 2.89494300 8.51272200 -2.29654700

C 1.57645500 12.53363900 -4.29964200

C 2.04104100 11.61618800 -3.34567900

C 1.99407400 11.99809700 -1.99528100

C 1.54710200 13.26780900 -1.62224100

C 1.10205300 14.16593200 -2.59001600

C 1.09766000 13.79003300 -3.93460900

H 1.60539400 12.25138300 -5.34814600

H 1.54494400 13.52893800 -0.56940300

H 0.74879100 15.14818900 -2.29104000

H 0.73934500 14.47523900 -4.69648900

O 2.41676300 11.16874200 -0.97325400

N 1.84093800 9.84583700 -1.10032800

C 0.75186000 9.68636900 -0.33478100

O 0.13031600 8.59432600 -0.33316000

C 0.25875000 10.83497500 0.52450300

H 1.05976500 11.26944000 1.12725100

H -0.15568500 11.63276600 -0.10014600

H -0.52929700 10.45674100 1.17800100

C 3.41540900 7.06171700 -0.65166400

C 2.46402700 6.44904500 -1.57754300

C 3.11941500 6.32729300 -2.84283300

C 4.42176500 6.90277500 -2.72516200

C 4.62433000 7.31083300 -1.34604300

C 5.46739000 6.89605200 -3.79630700

H 6.00845700 5.94117200 -3.78723600

H 5.02607500 7.01475300 -4.78923600

H 6.21025500 7.68547400 -3.65422200

C 5.89313800 7.87996600 -0.79660600

H 6.63953600 7.09108100 -0.63752900

H 6.33925200 8.60826600 -1.48271900

H 5.72588400 8.37858700 0.16119600

C 2.56945400 5.64654000 -4.05352200

H 2.92094300 6.11395600 -4.97701600

H 2.89240800 4.59749200 -4.07306800

H 1.47774800 5.65623700 -4.05504300

C 1.17242500 5.78569700 -1.21676400

H 0.43414000 5.89058000 -2.01711200

H 1.33571900 4.71167700 -1.05414600

H 0.74429500 6.20637000 -0.30806500

C 3.15251000 7.30625300 0.80012900

H 2.11038400 7.58173000 0.97072700

H 3.35755000 6.39771800 1.38085900

H 3.78734400 8.10373100 1.19401400

C 1.80055800 9.11025900 -4.00911200

H 2.18792200 8.46477800 -4.79711000

C 2.60201900 10.32638800 -3.87127100

C 3.85087200 10.52653300 -4.66073700

C 4.29973600 10.17298300 -3.27282200

H 4.04437700 11.53833800 -5.00662700

H 4.05354500 9.76494200 -5.41135600

H 4.98677300 9.34312400 -3.21315800

H 4.50766800 10.99309600 -2.59642000

C 0.32246000 9.02892300 -3.95900100

C -0.27304600 7.96002300 -4.66294400

C -0.54109700 9.90174400 -3.26732700

C -1.65025500 7.74993700 -4.65171900

H 0.36336300 7.28719500 -5.23000000

C -1.91937100 9.69481300 -3.26211800

H -0.14304100 10.73898700 -2.71597400

C -2.48690500 8.61690400 -3.94537400

H -2.06960500 6.91245500 -5.20286500

H -2.55453200 10.38534800 -2.71349000

H -3.56163100 8.46045300 -3.93423000

**TS-3 (in Dioxane)**

Rh 2.89990900 8.52191700 -2.27818100

C 1.44637000 12.45562100 -4.32289900

C 1.98945300 11.59241000 -3.36217700

C 1.97196700 12.00271200 -2.01897100

C 1.47861500 13.26231200 -1.66735500

C 0.95539300 14.10919100 -2.64136600

C 0.91946700 13.69675500 -3.97399100

H 1.45030500 12.14375600 -5.36337100

H 1.50419400 13.55307200 -0.62303200

H 0.56513400 15.08146400 -2.35612600

H 0.49818700 14.34126000 -4.73888100

O 2.46952800 11.21741100 -1.00833900

N 1.89114500 9.88487800 -1.06971300

C 0.91419600 9.71638300 -0.15139200

O 0.32658100 8.62863500 -0.02895200

C 0.52486400 10.89420400 0.73141100

H 1.38866000 11.32228900 1.24623300

H 0.06422800 11.69199900 0.14049600

H -0.20050700 10.53267500 1.46163700

C 3.40333700 7.05332400 -0.62799200

C 2.42570800 6.47747500 -1.55041100

C 3.06937200 6.34132600 -2.82086000

C 4.38670600 6.88746500 -2.71191700

C 4.61081400 7.27672900 -1.33026400

C 5.43106400 6.87546100 -3.78589900

H 5.97994500 5.92518800 -3.77823000

H 4.99251700 6.99076400 -4.78064500

H 6.17152300 7.66917700 -3.64936300

C 5.89326100 7.82336900 -0.78899000

H 6.64035000 7.03034000 -0.65585300

H 6.33445100 8.56794200 -1.46166500

H 5.74216500 8.30479100 0.17984400

C 2.47874500 5.69177600 -4.03062100

H 2.92332800 6.07022100 -4.95508600

H 2.65389900 4.60858800 -4.00548800

H 1.39941200 5.84997000 -4.08025900

C 1.09761500 5.90027600 -1.18352100

H 0.42115000 5.90012100 -2.04217700

H 1.21111400 4.86125700 -0.84350300

H 0.62614600 6.49155800 -0.40117100

C 3.15526600 7.29050400 0.82681900

H 2.11563900 7.57451800 0.99480000

H 3.36058600 6.37701100 1.40007100

H 3.79702300 8.08286300 1.22013100

C 1.83968600 9.08345900 -4.01399700

H 2.26150600 8.45323200 -4.79829800

C 2.60869200 10.32208400 -3.86607000

C 3.86074900 10.56063300 -4.64082200

C 4.28119200 10.20485300 -3.24350000

H 4.03533600 11.58018400 -4.97317800

H 4.09662700 9.81331600 -5.39662800

H 4.97012000 9.37609200 -3.17005300

H 4.46338800 11.01672300 -2.55148000

C 0.36172400 8.98549000 -4.02810300

C -0.20782600 8.04028100 -4.90449700

C -0.51704200 9.73698000 -3.22575900

C -1.58506500 7.83859200 -4.96836400

H 0.44631300 7.45993300 -5.54931800

C -1.89277100 9.54014500 -3.29549800

H -0.12126000 10.45861500 -2.52893800

C -2.43880100 8.58959600 -4.16096700

H -1.98900700 7.09925900 -5.65457100

H -2.54419400 10.13064900 -2.65744500

H -3.51331400 8.43851100 -4.20486500

**TS-3a (in TFE)**

Rh 1.88775200 8.22133400 -1.78537500

C 2.11418300 12.34587300 -4.10166000

C 1.73210500 11.31976700 -3.22843300

C 2.39402000 11.25055700 -1.97551600

C 3.30636700 12.25858300 -1.60212400

C 3.65066600 13.27238100 -2.49030300

C 3.06441200 13.30949800 -3.75672000

H 1.64989600 12.39665500 -5.08065800

H 3.76142400 12.19520200 -0.61792000

H 4.37698400 14.02408100 -2.19396800

H 3.32989000 14.08740300 -4.46614700

O 2.25466700 10.24932600 -1.09214500

N 0.50849000 9.25712500 -0.79803300

C 0.31185600 9.06437300 0.52155400

O -0.71326000 8.39848200 0.79768800

C 1.20643200 9.67015400 1.56446000

H 2.23942500 9.34923600 1.41771500

H 1.18633000 10.76118200 1.48785800

H 0.85188000 9.36208500 2.55047200

C 2.43060600 6.35707800 -0.52963000

C 2.02555800 6.03352900 -1.90019400

C 3.01480100 6.57246700 -2.79499700

C 3.90826500 7.35378400 -2.00816700

C 3.58408100 7.15557800 -0.59380100

C 5.07358900 8.14932000 -2.50167900

H 6.00594500 7.58419200 -2.37635900

H 4.96787700 8.39446100 -3.56082300

H 5.17686500 9.08309200 -1.94103100

C 4.37446200 7.73523900 0.53395200

H 5.34108800 7.22364700 0.62417800

H 4.58602100 8.79613000 0.36783100

H 3.85340000 7.63310900 1.48793100

C 3.11507600 6.32619500 -4.26345600

H 3.51395700 7.19490400 -4.79353400

H 3.79368700 5.48486900 -4.45185600

H 2.14605100 6.07427000 -4.69529000

C 0.93483400 5.07711400 -2.25577100

H 0.60995700 5.20141800 -3.28975100

H 1.28935100 4.04485900 -2.13325500

H 0.06618100 5.20781300 -1.60645400

C 1.69907200 5.90059900 0.69043700

H 0.61910500 6.03192300 0.57663400

H 1.88291600 4.83400900 0.86951900

H 2.01532400 6.45088200 1.57917900

C 0.99629400 8.89578000 -3.59129000

H 1.81164300 8.80168600 -4.31662400

C 0.61169300 10.37030100 -3.55459800

C -0.76559000 10.80758800 -3.10216000

C -0.43273800 10.84758900 -4.56397900

H -0.82436900 11.75417300 -2.57258000

H -1.45068200 10.04925500 -2.74360300

H -0.86533200 10.08657400 -5.20559800

H -0.31116600 11.81487500 -5.03870900

C -0.09389500 7.93793300 -3.97198400

C -0.07309200 7.34874400 -5.24976500

C -1.15507900 7.58663000 -3.11547400

C -1.05653500 6.44317000 -5.65392800

H 0.72510200 7.61276000 -5.93770300

C -2.14711600 6.69571000 -3.52112800

H -1.17763000 7.98711600 -2.10870200

C -2.10223100 6.11245300 -4.79070500

H -1.00632500 6.00241000 -6.64600600

H -2.95074300 6.44234000 -2.83473300

H -2.86911900 5.40810000 -5.09989300

**TS-3a (in Methanol)**

Rh 1.88868400 8.22342600 -1.78416100

C 2.10959300 12.34829800 -4.10181800

C 1.72822700 11.32261200 -3.22771700

C 2.38894600 11.25572700 -1.97407800

C 3.29894700 12.26605000 -1.60095700

C 3.64257700 13.27938900 -2.48998400

C 3.05783000 13.31394500 -3.75713000

H 1.64632000 12.39705800 -5.08140400

H 3.75355800 12.20540600 -0.61638100

H 4.36720300 14.03270100 -2.19351700

H 3.32286300 14.09138200 -4.46727700

O 2.25126400 10.25451800 -1.08982300

N 0.50990200 9.26184600 -0.79622200

C 0.31041100 9.06862500 0.52314000

O -0.72208500 8.41331400 0.79735500

C 1.21040000 9.66247600 1.56849000

H 2.24532100 9.35670500 1.40550400

H 1.17642300 10.75470100 1.51450500

H 0.86925000 9.33294300 2.55229800

C 2.43051600 6.35802400 -0.52897400

C 2.02411000 6.03592800 -1.89954100

C 3.01402500 6.57347100 -2.79438100

C 3.90923900 7.35255700 -2.00749500

C 3.58540400 7.15410500 -0.59311100

C 5.07756100 8.14284600 -2.50205800

H 6.00618800 7.56962900 -2.38560200

H 4.96800300 8.39563500 -3.55905700

H 5.19155500 9.07198800 -1.93597200

C 4.37956600 7.72665900 0.53547900

H 5.33802200 7.20065800 0.63116300

H 4.60836900 8.78334600 0.36636100

H 3.85336700 7.63607600 1.48780700

C 3.11490400 6.32686800 -4.26269900

H 3.51192000 7.19614300 -4.79326400

H 3.79562600 5.48705300 -4.45049100

H 2.14664500 6.07224900 -4.69458500

C 0.93289700 5.08012300 -2.25513400

H 0.61156300 5.20149500 -3.29055200

H 1.28558200 4.04775100 -2.12815100

H 0.06232600 5.21389600 -1.60900600

C 1.70083700 5.90012100 0.69153200

H 0.61997700 6.01889200 0.57403800

H 1.89528100 4.83621500 0.87588100

H 2.00931900 6.45741800 1.57866200

C 0.99684000 8.89729600 -3.59076200

H 1.81300300 8.80486700 -4.31537600

C 0.60952900 10.37130600 -3.55445900

C -0.76859400 10.80708100 -3.10320900

C -0.43463400 10.84710200 -4.56482300

H -0.82916400 11.75387300 -2.57410500

H -1.45339300 10.04826000 -2.74510200

H -0.86593300 10.08549800 -5.20662200

H -0.31402800 11.81442900 -5.03972800

C -0.09181300 7.93863600 -3.97363900

C -0.06881400 7.34949300 -5.25141900

C -1.15458700 7.58752800 -3.11914400

C -1.05173400 6.44409200 -5.65728800

H 0.73054700 7.61340100 -5.93804400

C -2.14622700 6.69687700 -3.52645800

H -1.17886700 7.98892900 -2.11285600

C -2.09911700 6.11357400 -4.79595900

H -0.99984000 6.00325900 -6.64927600

H -2.95128500 6.44376700 -2.84158600

H -2.86563800 5.40942700 -5.10662600

**TS-3a (in Dioxane)**

Rh 1.86159800 8.22469800 -1.78799700

C 2.17081500 12.33139500 -4.06984900

C 1.75528100 11.31233800 -3.20653800

C 2.40359600 11.21136000 -1.94940300

C 3.34442500 12.18697200 -1.56384000

C 3.72333900 13.19616900 -2.44239600

C 3.14640600 13.26301300 -3.71103600

H 1.71227300 12.39984000 -5.05066500

H 3.78940800 12.10342400 -0.57710000

H 4.47025900 13.92335200 -2.13639300

H 3.43989900 14.03898600 -4.41127800

O 2.21338400 10.21174000 -1.07598600

N 0.42946500 9.20881400 -0.83581300

C 0.20335700 8.96650700 0.47660400

O -0.80455800 8.26964300 0.68809700

C 1.05752500 9.56809600 1.55798900

H 2.11823300 9.45471500 1.33131000

H 0.85316000 10.64129500 1.62572000

H 0.80621800 9.09299400 2.50881100

C 2.43061000 6.37422200 -0.51505100

C 2.02767600 6.03168300 -1.87961600

C 3.00621900 6.57976800 -2.78075000

C 3.88880400 7.38802600 -2.00676700

C 3.56482200 7.20179400 -0.59083600

C 5.04616200 8.19200500 -2.50948400

H 5.98964300 7.64799400 -2.37376700

H 4.94116300 8.41999300 -3.57240800

H 5.12954800 9.14089300 -1.97217800

C 4.32870600 7.83282700 0.52937300

H 5.35272300 7.44190400 0.56997400

H 4.39964700 8.91729100 0.39866700

H 3.86147400 7.63879200 1.49716300

C 3.09860600 6.31977500 -4.24775700

H 3.52517800 7.17077100 -4.78488200

H 3.74707000 5.45480200 -4.43493800

H 2.12063700 6.10134900 -4.67812900

C 0.94068300 5.06694500 -2.22758800

H 0.59892400 5.19679900 -3.25542600

H 1.29776600 4.03404400 -2.11707200

H 0.07617500 5.19189800 -1.57166300

C 1.70871400 5.91437100 0.71076800

H 0.65193700 6.19758700 0.68633100

H 1.75602000 4.82253600 0.79826600

H 2.14476000 6.34148700 1.61634000

C 1.00105100 8.91305000 -3.60097900

H 1.83056000 8.82971300 -4.31186000

C 0.62242000 10.38592300 -3.54453100

C -0.74828700 10.82234800 -3.07661400

C -0.42426900 10.88953300 -4.53826300

H -0.79504500 11.75493300 -2.52301300

H -1.43384300 10.06061000 -2.72790800

H -0.86812100 10.14605500 -5.19221600

H -0.29418000 11.86512200 -4.99362000

C -0.08040800 7.95840900 -4.01486000

C -0.04128900 7.39686700 -5.30424000

C -1.14640000 7.58078500 -3.17645900

C -1.01143900 6.49112100 -5.73728300

H 0.75950300 7.68591600 -5.97931800

C -2.12370600 6.68844900 -3.61117200

H -1.18284900 7.96018700 -2.16227700

C -2.06031900 6.13181100 -4.89096600

H -0.94859300 6.07297100 -6.73826000

H -2.93099400 6.41384000 -2.93782200

H -2.81685700 5.42641200 -5.22195300

**TS-3h (in Dioxane)**

Rh 1.17004100 8.06869200 -2.50904600

C 3.92755200 11.14059200 -0.77503400

C 2.60143200 10.69350600 -0.86136300

C 2.11479500 9.89125800 0.19075400

C 2.92024300 9.63575600 1.30962100

C 4.22071300 10.12233500 1.38150900

C 4.74272900 10.86377800 0.32022800

H 4.31172200 11.74587400 -1.59085200

H 2.50332400 9.03456400 2.11068200

H 4.82563000 9.90959600 2.25804100

H 5.76188800 11.23607800 0.35196300

O 0.86796800 9.32841300 0.28289500

N 0.19092500 9.02166800 -0.94962200

C -1.11654000 9.33115200 -0.89776600

O -1.87631800 9.07736700 -1.85707900

C -1.65036700 10.00458600 0.35512200

H -1.53931700 9.35578100 1.22929600

H -1.10760100 10.92837100 0.57249500

H -2.70686700 10.22596700 0.19924300

C 1.12254800 5.91158200 -1.88621500

C 1.21246400 5.94038400 -3.30314600

C 2.43397900 6.63060500 -3.65686800

C 3.14948200 6.93614100 -2.43798900

C 2.33260500 6.52411500 -1.35137100

C 4.50204900 7.57140300 -2.34635300

H 5.29624300 6.81392400 -2.37538000

H 4.67758300 8.25855300 -3.17876800

H 4.61402600 8.14241100 -1.42190700

C 2.68017700 6.55095800 0.10172900

H 2.94472300 5.54041500 0.43967400

H 3.52679800 7.20694100 0.30294300

H 1.83665400 6.89360700 0.70450500

C 2.97479400 6.79467400 -5.04345000

H 3.56375600 7.71111300 -5.14113600

H 3.63464700 5.95695900 -5.30478200

H 2.17317900 6.82283000 -5.78433100

C 0.23434700 5.33966400 -4.26393700

H 0.23285400 5.86082800 -5.22166000

H 0.48786100 4.28865400 -4.45488600

H -0.78347200 5.37188200 -3.87052000

C 0.03905800 5.29738400 -1.05659200

H -0.89371900 5.21851900 -1.61984100

H 0.31612900 4.29006100 -0.71872500

H -0.16324700 5.90223200 -0.16852000

C 1.71579700 10.03475800 -3.12770000

H 2.65618300 9.93873800 -3.67270300

C 1.76387800 11.08190600 -2.04774200

C 0.60073900 12.00827400 -1.80165300

C 1.82430300 12.53310200 -2.50244900

H 0.43982100 12.31964700 -0.77463300

H -0.31421400 11.87549400 -2.36791200

H 1.75699900 12.71541700 -3.57139300

H 2.47108100 13.22116600 -1.96708000

C -0.45345400 8.49459200 -5.90323400

C -0.18229200 7.83419000 -7.10968200

C -1.66668000 8.23162500 -5.25333400

C -1.08997400 6.92425000 -7.65138000

H 0.75385400 8.03293300 -7.62635300

C -2.57387400 7.31745100 -5.79305400

H -1.90612500 8.71300700 -4.30999700

C -2.29008000 6.65846200 -6.98923100

H -0.85685900 6.41820700 -8.58394700

H -3.50392900 7.11891000 -5.26823300

H -2.99634200 5.94444300 -7.40249200

C 0.52153800 9.70926100 -3.85079800

C 0.57211100 9.45974000 -5.34509600

H -0.38228900 10.21439100 -3.51933100

H -0.17598000 8.46099500 -3.25299500

H 0.44170300 10.43998000 -5.82906600

H 1.57367400 9.12037600 -5.62434300

**TS-3i (in Dioxane)**

Rh 1.01308600 7.72720000 -1.75545900

C 2.83229300 11.94036000 -0.73368000

C 1.92950900 10.89026800 -0.92309000

C 1.73411300 9.98517000 0.14882400

C 2.35713400 10.21939000 1.38945700

C 3.24253800 11.28116800 1.54810000

C 3.49597400 12.14045600 0.47843100

H 3.01512000 12.62237800 -1.55808000

H 2.15344200 9.53019000 2.20310000

H 3.73482300 11.43204000 2.50479100

H 4.19196500 12.96653500 0.58731700

O 1.00475900 8.85986500 0.06077900

N -0.64147200 8.62211100 -1.10928600

C -1.56399600 7.91236800 -0.41729500

O -2.55819400 7.60295800 -1.09581000

C -1.41508700 7.60974600 1.04873000

H -0.43902400 7.17064800 1.26041700

H -1.48442100 8.53707700 1.62508800

H -2.21932100 6.93438400 1.34821500

C 1.79191100 5.76217900 -0.78626200

C 0.69018400 5.41982600 -1.58483600

C 0.96891700 5.86806100 -2.95070300

C 2.30813200 6.38838700 -2.98414300

C 2.78060700 6.43043500 -1.63862000

C 3.08145600 6.77814900 -4.20135800

H 3.73453600 5.95337800 -4.51269200

H 2.42191000 7.00911200 -5.03890400

H 3.71755900 7.64758000 -4.01787000

C 4.12031600 6.90939400 -1.17433800

H 4.82750100 6.07501700 -1.08058300

H 4.54829200 7.63084000 -1.87436400

H 4.05015200 7.39649400 -0.19777600

C 0.08517800 5.57633900 -4.12167100

H 0.35126400 6.17432900 -4.99584800

H 0.16528000 4.51963900 -4.40941900

H -0.96321200 5.77656500 -3.88459900

C -0.57687000 4.74106700 -1.17312400

H -1.45672100 5.33142200 -1.44779600

H -0.66993600 3.76537000 -1.66514800

H -0.61142900 4.57345900 -0.09450800

C 1.96475700 5.56343800 0.68565300

H 1.08318000 5.10422400 1.13809200

H 2.82313900 4.91298200 0.89217100

H 2.14714500 6.51650800 1.19212100

C 1.40535600 9.44218200 -2.94484600

H 2.49148100 9.35752000 -3.07953400

C 1.12983600 10.72643100 -2.18258200

C -0.27220300 11.29754700 -2.12062800

C 0.76444600 12.01345300 -2.92881400

H -0.58122500 11.73336200 -1.17595300

H -1.05644200 10.75126500 -2.63205400

H 0.68646600 11.97061700 -4.00796500

H 1.15272200 12.95448500 -2.55393400

C 0.83934900 9.57367700 -6.81059400

C 1.14992500 8.43961300 -7.57249700

C -0.18458800 10.41738100 -7.26216300

C 0.45890200 8.15382500 -8.75097100

H 1.94980500 7.78039500 -7.24362400

C -0.87749100 10.13717000 -8.43926400

H -0.43827600 11.30274200 -6.68438600

C -0.55884700 9.00230100 -9.18798600

H 0.71832400 7.27147200 -9.32944800

H -1.66562900 10.80576500 -8.77394800

H -1.09746300 8.78378200 -10.10539000

C 0.71303100 9.30302600 -4.30429000

C 1.53692500 9.84128600 -5.49991900

H -0.26951600 9.78596500 -4.29463600

H 0.50172700 8.25316200 -4.51285000

H 1.72258400 10.91316800 -5.38272100

H 2.51869300 9.35640500 -5.49794100

**TS-3j (in Dioxane)**

Rh 2.91198500 8.53962500 -2.27996800

C 1.78779200 12.55811400 -4.40429900

C 2.16538000 11.64091400 -3.41341500

C 2.00321700 12.02102200 -2.07033400

C 1.53614400 13.29740600 -1.74378200

C 1.18198600 14.19625200 -2.74642100

C 1.28908600 13.81889400 -4.08537400

H 1.90529900 12.27391500 -5.44630500

H 1.44952300 13.56077100 -0.69534400

H 0.81153300 15.18154600 -2.48023800

H 1.00115000 14.50406700 -4.87633100

O 2.34860900 11.20420700 -1.02027900

N 1.78697700 9.87826700 -1.17291400

C 0.65747100 9.71434900 -0.45768500

O 0.05173500 8.62355100 -0.45453600

C 0.11317200 10.88616600 0.34352800

H 0.87611300 11.33287700 0.98549000

H -0.26009300 11.67078700 -0.32224600

H -0.71467200 10.51740200 0.95049600

C 3.32285000 7.09411000 -0.58912600

C 2.42807800 6.47648700 -1.56617400

C 3.15498500 6.34824700 -2.79026600

C 4.45038800 6.92105700 -2.59483900

C 4.57172700 7.33085400 -1.20418600

C 5.57021300 6.90021500 -3.59045800

H 6.12366400 5.95501600 -3.52057600

H 5.20415500 6.98861500 -4.61677200

H 6.29348200 7.70253900 -3.41844200

C 5.80420500 7.90337300 -0.57908500

H 6.57022700 7.13172500 -0.43000900

H 6.25547200 8.68518600 -1.20097100

H 5.58394400 8.34571100 0.39524300

C 2.67568900 5.67794000 -4.03855300

H 3.12201900 6.12088600 -4.93326300

H 2.94519700 4.61400400 -4.03289300

H 1.59064100 5.74453600 -4.13698300

C 1.12084000 5.83192000 -1.23211800

H 0.58666700 5.52195300 -2.13307700

H 1.29126000 4.93216200 -0.62503900

H 0.48500000 6.52100800 -0.67659900

C 2.94228700 7.36035200 0.83226000

H 1.88822600 7.63572300 0.89614500

H 3.09779900 6.46369100 1.44591200

H 3.53592400 8.16962200 1.26436700

C 2.75695600 10.34106700 -3.88245200

C 4.05449600 10.51191100 -4.59478700

C 4.38854000 10.18818200 -3.16735700

H 4.28478300 11.51186400 -4.95254200

H 4.30278200 9.72825400 -5.30884200

H 5.05941500 9.35481900 -3.02846500

H 4.54468800 11.01771400 -2.48919600

C -1.87110400 8.28462400 -3.77480700

C -2.63147400 8.22034300 -4.94893400

C -2.51237100 8.62827500 -2.57566100

C -4.00095600 8.48933800 -4.93182500

H -2.14501900 7.95347400 -5.88473300

C -3.88101400 8.89755500 -2.55713700

H -1.92352400 8.67822500 -1.66289100

C -4.63064200 8.82977100 -3.73370000

H -4.57570900 8.43023300 -5.85226900

H -4.36541700 9.15788300 -1.61965400

H -5.69688000 9.03760100 -3.71665300

C 1.90470000 9.15668300 -4.00763100

H 2.22132800 8.48213100 -4.80883900

C 0.40116800 9.35451400 -3.98703200

H 0.08173100 9.82442300 -4.93074100

H 0.12321200 10.04749800 -3.19134600

C -0.38061800 8.04742600 -3.79332900

H -0.12349300 7.34394800 -4.59575600

H -0.06574000 7.61154700 -2.84436700

**TS-4 (in TFE)**

Rh 1.02128300 7.61213700 -2.46807900

C 4.01296200 9.93344700 -6.00983000

C 3.24025300 9.69252500 -4.85583200

C 3.90022900 9.80221200 -3.61600500

C 5.25514300 10.15218700 -3.54927200

C 5.98413600 10.38651900 -4.70843400

C 5.35865600 10.27644900 -5.95361600

H 3.52160700 9.83964100 -6.97303900

H 5.71246400 10.24122100 -2.56870300

H 7.03332400 10.65759500 -4.63845600

H 5.91365400 10.45256700 -6.86964000

O 3.33855400 9.58594000 -2.37092200

N 1.91279900 9.49190000 -2.35100100

C 1.31331700 10.67155400 -2.13061400

O 0.05965600 10.75068800 -2.11081600

C 2.16365900 11.90365500 -1.88595900

H 1.50044400 12.76153300 -1.76613600

H 2.76117800 11.77604200 -0.97724300

H 2.85815800 12.09780100 -2.70636300

C -0.13167600 7.48210800 -0.51942200

C -0.82883600 6.69924200 -1.47539700

C 0.07594300 5.68126200 -1.96205800

C 1.30709500 5.77338600 -1.20202300

C 1.20609800 6.91209000 -0.35882000

C 2.46002600 4.82383700 -1.28814500

H 2.30004300 3.97131100 -0.61547300

H 2.57990400 4.42556100 -2.29858800

H 3.39675600 5.30543200 -0.99739500

C 2.22907400 7.41231100 0.61159500

H 2.00641600 7.05577000 1.62557500

H 3.23271300 7.06974100 0.34838500

H 2.23923000 8.50510300 0.64335200

C -0.26286800 4.59885000 -2.93865400

H 0.62695900 4.25335200 -3.47180500

H -0.69931700 3.73107000 -2.42761100

H -0.98774700 4.94706200 -3.67920000

C -2.25003600 6.88121100 -1.90785400

H -2.57040100 7.92027100 -1.80025000

H -2.39255100 6.58609800 -2.95047000

H -2.91702500 6.26050700 -1.29579700

C -0.68185200 8.60275000 0.30436900

H -1.52443700 9.09057200 -0.18983500

H -1.03327800 8.22391600 1.27340600

H 0.07787000 9.36150600 0.50734600

C 0.92725600 10.04277100 -5.75888100

H -0.02955200 9.56793000 -5.97537900

C 1.81953200 9.29189000 -5.07624600

C 2.31423700 7.01199800 -4.03988700

C 1.38889900 7.90759200 -4.67392600

H 2.17737800 5.94638900 -4.19846700

H 3.34408900 7.32317300 -3.90092100

H 0.65318100 7.46675900 -5.34573100

H 0.28230600 8.29032600 -3.73124700

C 1.03133800 11.41690200 -6.26144300

C 1.90265700 12.38852400 -5.73138400

C 0.17659100 11.79802400 -7.31384000

C 1.93062000 13.67891000 -6.25414300

H 2.55118800 12.13646800 -4.90292100

C 0.21256300 13.08678300 -7.84250700

H -0.51636000 11.06613800 -7.72077600

C 1.09369500 14.03376500 -7.31639500

H 2.60659500 14.41397400 -5.82641800

H -0.44995400 13.35228800 -8.66141700

H 1.12188600 15.04071600 -7.72235400

**TS-4 (in Methanol)**

Rh 1.03195800 7.61488700 -2.46001800

C 4.03532600 9.94254800 -5.98880500

C 3.25440400 9.70186500 -4.84027700

C 3.90141600 9.82628600 -3.59492200

C 5.25245300 10.18965200 -3.51856400

C 5.98973000 10.42356800 -4.67254800

C 5.37698500 10.29929400 -5.92268100

H 3.55371000 9.83745400 -6.95577100

H 5.70068500 10.28943400 -2.53484800

H 7.03553900 10.70539700 -4.59458600

H 5.93866200 10.47511500 -6.83470600

O 3.33056000 9.61331000 -2.35412400

N 1.90472000 9.50574600 -2.34551800

C 1.29289000 10.68185200 -2.14089100

O 0.03824800 10.74994900 -2.13027400

C 2.12920600 11.92497100 -1.90289100

H 1.45750100 12.77972000 -1.81096800

H 2.70903500 11.81911800 -0.97995700

H 2.83828900 12.11079700 -2.71250100

C -0.12959600 7.47741700 -0.51571700

C -0.82042800 6.69581600 -1.47720900

C 0.08977600 5.68161600 -1.96238600

C 1.31645700 5.77422900 -1.19533800

C 1.20870600 6.91009100 -0.34947000

C 2.47151800 4.82692400 -1.27749800

H 2.31043500 3.97428400 -0.60518900

H 2.59557600 4.42847800 -2.28735900

H 3.40647300 5.31000800 -0.98334700

C 2.22330900 7.40628100 0.63168500

H 1.99650200 7.03836200 1.64074000

H 3.23038200 7.07144600 0.37150200

H 2.22757300 8.49868900 0.67499900

C -0.24089300 4.60097100 -2.94368800

H 0.65291000 4.25866500 -3.47222200

H -0.67852800 3.73096300 -2.43740700

H -0.96200800 4.94935200 -3.68780600

C -2.24017800 6.87464300 -1.91590600

H -2.56418000 7.91259100 -1.80800000

H -2.37704800 6.58121000 -2.95976400

H -2.90834100 6.25096300 -1.30812800

C -0.68598600 8.59317000 0.31072600

H -1.53624600 9.07286600 -0.17824300

H -1.02889700 8.21035200 1.28126700

H 0.06772000 9.35858900 0.51129200

C 0.94646200 10.02319100 -5.76751900

H -0.00310900 9.53667300 -5.98996700

C 1.83988300 9.28583200 -5.07157400

C 2.34682300 7.01810000 -4.01435200

C 1.41950500 7.89994400 -4.66425000

H 2.22194900 5.95027200 -4.16756700

H 3.37219200 7.34001000 -3.86709900

H 0.69438800 7.44728700 -5.33971100

H 0.29954100 8.27859100 -3.73415300

C 1.03892400 11.39605000 -6.27584100

C 1.89812200 12.37916700 -5.74685400

C 0.18361700 11.76403400 -7.33256200

C 1.91367700 13.66784400 -6.27437300

H 2.54674700 12.13746600 -4.91538800

C 0.20723200 13.05116800 -7.86591100

H -0.50024300 11.02326900 -7.73885000

C 1.07627200 14.00970800 -7.34052500

H 2.58026400 14.41180000 -5.84723300

H -0.45554500 13.30645700 -8.68789500

H 1.09469500 15.01545400 -7.75007400

**TS-4 (in Dioxane)**

Rh 1.07918600 7.57461800 -2.48790900

C 4.13792300 9.91613800 -5.88104000

C 3.29486600 9.72083500 -4.76981900

C 3.86465700 9.89784100 -3.49184000

C 5.21052600 10.27109500 -3.35941000

C 6.00928800 10.46399300 -4.47803000

C 5.47132300 10.28486400 -5.75532500

H 3.70767600 9.77449500 -6.86729800

H 5.60199800 10.40890100 -2.35672700

H 7.04802000 10.75480500 -4.35237900

H 6.08264700 10.42926000 -6.64021900

O 3.22603000 9.74098300 -2.28757400

N 1.82094300 9.50146100 -2.36605600

C 1.06979900 10.62505000 -2.26376700

O -0.16671900 10.57091600 -2.33918400

C 1.78575400 11.94979500 -2.04392000

H 1.03350600 12.73828400 -2.00879200

H 2.34854100 11.93520500 -1.10510800

H 2.49813000 12.17044700 -2.84215600

C -0.01233000 7.48497900 -0.51680700

C -0.78137800 6.73762600 -1.44461900

C 0.05168400 5.67377300 -1.95761300

C 1.31119100 5.71010900 -1.24538700

C 1.29824800 6.84785600 -0.39304900

C 2.41760300 4.71144000 -1.38203200

H 2.25063800 3.85820200 -0.71214500

H 2.48504900 4.31802000 -2.39935300

H 3.38557800 5.14933400 -1.12806600

C 2.37719300 7.28721500 0.54573000

H 2.17389100 6.94327300 1.56831100

H 3.35143400 6.89366900 0.24615300

H 2.45434000 8.37680400 0.57107800

C -0.38524000 4.60034400 -2.90606900

H 0.46541000 4.15651000 -3.43010500

H -0.90066500 3.78877300 -2.37621200

H -1.07504800 4.98996900 -3.65981800

C -2.20346700 7.00366300 -1.82674200

H -2.43626300 8.06882800 -1.76490100

H -2.41420500 6.67212000 -2.84670500

H -2.89113400 6.46949300 -1.15824700

C -0.47507400 8.66302400 0.28056900

H -1.14958900 9.29437100 -0.29867700

H -0.99293600 8.33237400 1.19066100

H 0.36921100 9.28352500 0.58959900

C 1.01266800 10.01450000 -5.77761000

H 0.07171000 9.51613700 -6.01015200

C 1.89681800 9.28759800 -5.05963700

C 2.43565400 7.02658200 -4.01535300

C 1.49564400 7.88963800 -4.67579600

H 2.35165700 5.95724800 -4.18858500

H 3.44800800 7.37800600 -3.84975100

H 0.79837500 7.42518100 -5.37269500

H 0.34497000 8.23667800 -3.75576400

C 1.07709400 11.38947800 -6.28180200

C 2.00057700 12.36192200 -5.84973000

C 0.12074500 11.77470900 -7.24123200

C 1.97050400 13.65255400 -6.36875800

H 2.74016600 12.10964200 -5.10311400

C 0.09629500 13.06422900 -7.76540200

H -0.61195700 11.04473100 -7.57530200

C 1.02480000 14.01137500 -7.33222900

H 2.68936800 14.38560500 -6.01413300

H -0.65097000 13.33096700 -8.50715000

H 1.00735800 15.02019700 -7.73381700

**TS-4a(in TFE)**

Rh 1.04923600 8.82274300 -2.36204800

C 0.68162200 13.05935800 -3.61414600

C 1.00222100 11.72411200 -3.34946500

C 2.30426700 11.36938100 -2.92359900

C 3.24621400 12.40020400 -2.71950100

C 2.89973800 13.72804100 -2.95039200

C 1.61724100 14.07005100 -3.39844300

H -0.31721300 13.30322100 -3.96703200

H 4.24594200 12.13242700 -2.39125300

H 3.63928600 14.50702200 -2.78417800

H 1.35340300 15.10742300 -3.57804300

O 2.64934800 10.09540700 -2.78348800

N 1.06725800 9.83667700 -0.53368500

C 2.14581100 10.13953400 0.15782700

O 1.98464500 10.81729900 1.24265600

C 3.59370500 9.78094000 -0.15632600

H 3.71864200 9.03371600 -0.93455200

H 4.12256000 10.68794000 -0.46655600

H 4.06070600 9.43598000 0.77196500

C 0.11870800 7.10679800 -1.27321100

C -0.07805400 6.87984000 -2.67382000

C 1.21222000 6.59568700 -3.27199300

C 2.19867300 6.73134200 -2.26867900

C 1.53236700 7.02792700 -1.02220900

C 3.66439300 6.54401300 -2.47184700

H 3.90844500 5.47467800 -2.50412900

H 3.98678100 6.98390300 -3.42027400

H 4.24408900 6.99445800 -1.66455200

C 2.13249600 6.97433600 0.33746600

H 1.94254600 5.97779100 0.75799500

H 3.21055600 7.12833500 0.32174500

H 1.67413100 7.70777400 1.00245100

C 1.46993500 6.17428700 -4.67940000

H 2.21139200 6.81772300 -5.16647600

H 1.87245900 5.15457900 -4.69012500

H 0.55767400 6.17371400 -5.27934100

C -1.39524900 6.73476400 -3.36204400

H -2.19601600 7.22720500 -2.80831500

H -1.37821900 7.13375400 -4.37963500

H -1.64618200 5.66927100 -3.43508200

C -0.91230700 7.29293300 -0.21447400

H -0.71486600 8.22008700 0.33383400

H -1.91893400 7.33970800 -0.63088000

H -0.87523000 6.46217800 0.49977400

C -0.85090100 10.06928100 -2.59315400

H -1.54104100 9.34236000 -3.01210800

C -0.00053300 10.65017700 -3.59408400

C -0.30343700 10.38947200 -5.04030600

C 0.70446500 9.34149900 -4.74137300

H 0.02977700 11.18937700 -5.69655400

H -1.30696400 10.03254100 -5.25853100

H 0.34936000 8.33196400 -4.84409400

H 1.74173400 9.53492100 -4.97802900

C -1.40555500 10.73456000 -1.41933600

C -2.61684700 10.22149400 -0.91605600

C -0.81998100 11.83136700 -0.75531200

C -3.23528500 10.78665000 0.19619600

H -3.07556600 9.37107800 -1.41214500

C -1.44741900 12.40393600 0.34856700

H 0.11516600 12.23670100 -1.10771700

C -2.65076700 11.88469700 0.83303500

H -4.17166400 10.37498200 0.56150000

H -0.98424300 13.25266400 0.84347900

H -3.12953200 12.33217800 1.69946400

**TS-4a (in Methanol)**

Rh 1.05322000 8.82658600 -2.36189500

C 0.67384200 13.06254200 -3.61189900

C 0.99882200 11.72812700 -3.34836600

C 2.30221600 11.37673600 -2.92333500

C 3.23991400 12.41099200 -2.71585600

C 2.88889700 13.73802400 -2.94497300

C 1.60587900 14.07622200 -3.39434600

H -0.32574500 13.30345500 -3.96471400

H 4.24046700 12.14719400 -2.38684000

H 3.62552700 14.51934200 -2.77654800

H 1.33864900 15.11292200 -3.57297600

O 2.65177100 10.10372500 -2.78760900

N 1.07999700 9.84082200 -0.53287200

C 2.16305600 10.13079700 0.15688100

O 2.01255100 10.80209700 1.24745900

C 3.60689900 9.76467300 -0.16738300

H 3.72227900 9.02047400 -0.94972200

H 4.13978700 10.66975700 -0.47652400

H 4.07805900 9.41204300 0.75589400

C 0.12108600 7.11178700 -1.27310300

C -0.07587600 6.88480600 -2.67386100

C 1.21396800 6.59741600 -3.27127200

C 2.20017900 6.72945700 -2.26723300

C 1.53421200 7.02824400 -1.02148500

C 3.66470100 6.53124700 -2.46826200

H 3.90090400 5.45986600 -2.49293200

H 3.99109100 6.96163300 -3.41964600

H 4.24735700 6.98254300 -1.66357700

C 2.13325100 6.97095200 0.33844600

H 1.93797200 5.97494900 0.75788500

H 3.21210100 7.11927900 0.32357600

H 1.67797800 7.70566800 1.00416100

C 1.47128100 6.17340800 -4.67793400

H 2.21758300 6.81177100 -5.16425200

H 1.86769900 5.15122900 -4.68683000

H 0.56015700 6.17757800 -5.27956700

C -1.39311400 6.73955900 -3.36182900

H -2.19437200 7.23036700 -2.80737400

H -1.37703100 7.13926600 -4.37913800

H -1.64250400 5.67370000 -3.43555900

C -0.91004700 7.29693600 -0.21429800

H -0.71109100 8.22127600 0.33815200

H -1.91626000 7.34751400 -0.63111100

H -0.87537500 6.46334000 0.49681500

C -0.84687600 10.06789100 -2.58836000

H -1.53605900 9.33950100 -3.00600600

C -0.00134600 10.65172500 -3.59127600

C -0.30419400 10.38673800 -5.03654800

C 0.70698000 9.34094000 -4.74073300

H 0.02568400 11.18666300 -5.69458100

H -1.30715400 10.02697500 -5.25290200

H 0.35498100 8.33055300 -4.84521500

H 1.74295400 9.53844900 -4.97917600

C -1.40495800 10.73460200 -1.41599200

C -2.61553800 10.21932400 -0.91348700

C -0.82680700 11.83829600 -0.75741400

C -3.23999300 10.78856100 0.19348600

H -3.06924700 9.36430200 -1.40615800

C -1.46079900 12.41560300 0.34030900

H 0.10737100 12.24583200 -1.11002600

C -2.66313000 11.89368300 0.82479200

H -4.17559800 10.37475500 0.55843700

H -1.00400200 13.27075900 0.83025500

H -3.14699500 12.34498600 1.68641000

**TS-4a (in Dioxane)**

Rh 1.03812100 8.81017600 -2.39419600

C 0.74575100 13.05917500 -3.54805300

C 1.03908700 11.70955300 -3.32997600

C 2.33529900 11.31236200 -2.92164900

C 3.30527000 12.31558600 -2.71263900

C 2.98772800 13.65495800 -2.90344700

C 1.70674900 14.04049900 -3.31760000

H -0.25286200 13.33738000 -3.87587800

H 4.30012400 12.01429100 -2.40258100

H 3.74774300 14.41121900 -2.72637700

H 1.46345400 15.08872900 -3.45720000

O 2.64890200 10.03037900 -2.80613300

N 1.02015300 9.83647900 -0.56844000

C 2.08010700 10.18095900 0.16838800

O 1.84278500 10.82007200 1.22744100

C 3.54562100 9.87278400 -0.12851600

H 3.71183900 9.17326800 -0.94381000

H 4.04673600 10.81006400 -0.38758400

H 4.00254200 9.50892900 0.79694500

C 0.11301400 7.09137700 -1.27692000

C -0.09298500 6.86331500 -2.67723200

C 1.19178200 6.58748200 -3.28731200

C 2.18633500 6.74681200 -2.29433500

C 1.52749400 7.03627400 -1.04096500

C 3.65516400 6.57811400 -2.50363700

H 4.23147200 7.09150800 -1.73205200

H 3.92958100 5.51584700 -2.47397100

H 3.96442600 6.97391400 -3.47494800

C 2.14174500 7.02263200 0.31437700

H 1.97438000 6.03734900 0.77001900

H 3.21683300 7.19584900 0.28430400

H 1.68204900 7.77362000 0.95866600

C 1.44342700 6.19288400 -4.70685400

H 2.07065700 6.92191300 -5.23425000

H 1.96999900 5.23287300 -4.74816500

H 0.51283300 6.07812600 -5.26731700

C -1.41874100 6.71387200 -3.35346600

H -2.21204500 7.21405000 -2.79539500

H -1.41661400 7.11170100 -4.37257900

H -1.68518200 5.65208400 -3.42331900

C -0.89728100 7.33990300 -0.21069400

H -0.68452300 8.30986500 0.25687600

H -1.91467500 7.35660300 -0.60379200

H -0.84516600 6.56393700 0.56166400

C -0.89494700 10.09671200 -2.65110300

H -1.58172100 9.38539800 -3.10458300

C 0.01397800 10.66348000 -3.61587900

C -0.29770200 10.48090800 -5.07482500

C 0.66648400 9.39564200 -4.75586200

H 0.08077200 11.28474800 -5.69976000

H -1.31219900 10.17511200 -5.31981800

H 0.27217100 8.39718500 -4.84484900

H 1.71012600 9.54071500 -4.99940100

C -1.43816800 10.71080700 -1.46165500

C -2.67567900 10.21752500 -0.99246700

C -0.78808300 11.71229100 -0.70968600

C -3.26096400 10.71859200 0.16152100

H -3.17991300 9.43620800 -1.55615100

C -1.38500300 12.21758800 0.44478300

H 0.15656800 12.11048200 -1.03934000

C -2.61042800 11.72495400 0.88654500

H -4.21847400 10.33200700 0.49805100

H -0.86757800 12.98276900 1.01381700

H -3.06116000 12.11857200 1.79322100

**TS-4b (in TFE)**

Rh 3.13757000 7.92025800 -2.02665800

C 1.92075300 12.30401600 -4.11024200

C 1.74218100 11.03649700 -3.53645600

C 2.09396900 10.85485400 -2.18048400

C 2.64213000 11.93198300 -1.45573700

C 2.80685200 13.18008200 -2.05061400

C 2.44200300 13.37472200 -3.38536900

H 1.63682300 12.43918400 -5.15017300

H 2.90800500 11.76852000 -0.41544700

H 3.21828600 13.99967100 -1.46769500

H 2.56611800 14.34479000 -3.85689100

O 1.88256500 9.71677900 -1.48587200

N 1.21732500 7.97715400 -2.34302100

C 0.31804700 7.39884700 -1.52280000

O -0.12976700 6.30798300 -1.94388300

C -0.15623200 8.06288100 -0.26361000

H 0.68601600 8.29897100 0.39010500

H -0.66282300 9.00179300 -0.50502700

H -0.85123800 7.38916400 0.24234600

C 3.86016900 7.13857600 0.08255600

C 3.59816900 6.04895400 -0.76496000

C 4.43150900 6.19039400 -1.96762200

C 5.27514700 7.34455300 -1.78671000

C 4.84579600 7.98351300 -0.58706600

C 6.39827200 7.77400400 -2.67301300

H 7.33384700 7.30164900 -2.34874500

H 6.22660100 7.48331400 -3.71171100

H 6.54623500 8.85637100 -2.63962200

C 5.36124500 9.27445600 -0.04040900

H 6.20031300 9.09054900 0.64264700

H 5.71509000 9.93174900 -0.83803800

H 4.58644500 9.80043500 0.52289000

C 4.55772400 5.16496600 -3.04822200

H 4.91192400 5.61069700 -3.98084400

H 5.27595200 4.38804300 -2.75502100

H 3.59978800 4.67585900 -3.24236400

C 2.64219600 4.92410700 -0.53663800

H 3.18770200 4.01411000 -0.25764100

H 1.93438600 5.15187900 0.26284000

H 2.07266700 4.70049400 -1.44275300

C 3.24401500 7.44058900 1.41073600

H 2.37957900 6.80199900 1.60385400

H 3.96691800 7.27923400 2.22024900

H 2.91853500 8.48360600 1.47074400

C -0.03085900 9.85930400 -4.83800400

H -0.25730000 9.06321800 -5.54781600

C 1.23562100 9.92396100 -4.38375100

C 2.27369300 8.92493900 -4.86938200

C 3.46734800 8.66333800 -3.95466500

H 2.68454400 9.32796100 -5.81232000

H 1.77581600 7.98555300 -5.13030400

H 4.14596200 7.96286200 -4.44882900

H 4.01668300 9.59557300 -3.76480100

C -1.18074800 10.71550700 -4.51126900

C -2.23469300 10.80374100 -5.44147800

C -1.31617000 11.41982900 -3.29804100

C -3.36054200 11.58617600 -5.19025000

H -2.15932100 10.25232100 -6.37544600

C -2.44442200 12.19788400 -3.04531000

H -0.54215100 11.34574600 -2.54399500

C -3.47014000 12.29242100 -3.99023700

H -4.15423800 11.64083900 -5.93041000

H -2.52707600 12.72717300 -2.09981400

H -4.34772400 12.89985200 -3.78865700

**TS-4b (in Methanol)**

Rh 3.14971100 7.90112500 -2.05636100

C 1.87536300 12.34583300 -3.92987000

C 1.70407500 11.04366600 -3.43641400

C 2.01094400 10.79021100 -2.08166900

C 2.49373400 11.83793100 -1.27237700

C 2.65134300 13.12167900 -1.78786000

C 2.34063800 13.38405700 -3.12472100

H 1.63052100 12.53461400 -4.97141100

H 2.72152600 11.62304100 -0.23253800

H 3.01637600 13.91556700 -1.14184900

H 2.46180300 14.38185100 -3.53519800

O 1.82572800 9.60132900 -1.46657000

N 1.22992400 7.89887500 -2.42254100

C 0.32436400 7.25695500 -1.66134000

O -0.09160700 6.19036900 -2.17091200

C -0.18379700 7.81627800 -0.36573900

H 0.64251700 7.99688600 0.32562200

H -0.68623900 8.77206600 -0.54031500

H -0.88875800 7.10295900 0.06693500

C 3.94091800 7.28005700 0.06325900

C 3.61241100 6.11675900 -0.64587800

C 4.38403600 6.11642200 -1.89656200

C 5.27428000 7.24833500 -1.87211400

C 4.92287800 8.02591900 -0.73223000

C 6.36317400 7.54550200 -2.85005400

H 7.32208100 7.18195300 -2.46059000

H 6.18848700 7.05238800 -3.80869900

H 6.46513800 8.61891100 -3.02935200

C 5.53002700 9.32688000 -0.31886400

H 6.31478300 9.16184900 0.43043500

H 5.98050900 9.84499400 -1.16843600

H 4.78212000 9.98678000 0.12951500

C 4.40466900 4.99010100 -2.87877300

H 4.74610000 5.32189700 -3.86202400

H 5.08477300 4.19879800 -2.53656200

H 3.41143900 4.54747300 -2.99061300

C 2.64135500 5.04947300 -0.25885200

H 3.17282700 4.13426600 0.02922400

H 2.02049900 5.35807600 0.58494100

H 1.97933400 4.79366600 -1.09091200

C 3.39809200 7.74841100 1.37442200

H 2.53640500 7.15590100 1.68891000

H 4.16367500 7.67240300 2.15689500

H 3.09311600 8.79813600 1.32422800

C -0.00337500 9.92405000 -4.86593800

H -0.19521100 9.16430000 -5.62420500

C 1.24472600 9.97343900 -4.36030100

C 2.31259600 9.01444400 -4.86352900

C 3.49575800 8.74149200 -3.93861800

H 2.73056200 9.46387700 -5.78207100

H 1.83942200 8.07673000 -5.17244300

H 4.20014300 8.08194200 -4.45254300

H 4.01709500 9.67758200 -3.69701600

C -1.16672000 10.76911700 -4.55606800

C -2.17112800 10.90502800 -5.53431600

C -1.35774800 11.42753600 -3.32464900

C -3.30054000 11.69085800 -5.31062500

H -2.05288300 10.38974400 -6.48436600

C -2.48947000 12.20914000 -3.09994300

H -0.62459200 11.31644600 -2.53521200

C -3.46398000 12.35272200 -4.09161100

H -4.05464400 11.78353000 -6.08750700

H -2.61447000 12.70392500 -2.14046700

H -4.34374600 12.96390400 -3.91192500

**TS-4b (in Dioxane)**

Rh 3.17971800 7.92967900 -2.09023900

C 1.90236100 12.45642600 -3.80707900

C 1.76678700 11.12739700 -3.38295300

C 2.06167000 10.81255800 -2.03743700

C 2.49280200 11.83360800 -1.16821900

C 2.61502800 13.14599500 -1.61478400

C 2.31909400 13.46550600 -2.94147800

H 1.65951400 12.69046900 -4.83938300

H 2.70288500 11.57224200 -0.13556000

H 2.93859200 13.91884000 -0.92288500

H 2.41013200 14.48700600 -3.29723800

O 1.89544100 9.59110000 -1.49308300

N 1.29354100 7.89859400 -2.57338500

C 0.34212200 7.25062700 -1.87206800

O 0.02322900 6.16190700 -2.38282200

C -0.30138900 7.85671100 -0.65875100

H 0.45508400 8.18819300 0.05596100

H -0.87818400 8.73798900 -0.95440800

H -0.96710700 7.11656900 -0.20920000

C 3.95381000 7.37388600 0.03621800

C 3.54106900 6.18451700 -0.58331300

C 4.28753300 6.05380800 -1.83719800

C 5.24818000 7.12311600 -1.90161700

C 4.97790800 7.99791000 -0.80818300

C 6.33683900 7.27265000 -2.91395600

H 7.25196000 6.78460100 -2.55645200

H 6.06960100 6.81027200 -3.86683600

H 6.57379100 8.32220100 -3.10356800

C 5.70224200 9.26186100 -0.46607200

H 6.43414000 9.09134200 0.33357100

H 6.23815300 9.66119600 -1.32988200

H 5.00883400 10.03399500 -0.12022400

C 4.19810700 4.87768400 -2.75695200

H 4.53174100 5.13180300 -3.76546100

H 4.82441700 4.05239400 -2.39289800

H 3.17081800 4.51140800 -2.82615100

C 2.50524300 5.21276800 -0.11375900

H 2.96355300 4.24923000 0.13927200

H 1.98931600 5.57832900 0.77694300

H 1.74968600 5.02507300 -0.88300800

C 3.44163400 7.97926800 1.30363000

H 2.58366500 7.42884500 1.69508900

H 4.21955500 7.98210200 2.07691900

H 3.13333800 9.01693700 1.14510100

C 0.10201900 9.98856400 -4.85362600

H -0.05326700 9.22941800 -5.62023200

C 1.34807400 10.08312100 -4.35209200

C 2.44984500 9.17218500 -4.86825400

C 3.59764700 8.86606300 -3.90989900

H 2.90099100 9.67576300 -5.74207500

H 2.00459500 8.24395500 -5.23925100

H 4.32904300 8.23853200 -4.42611700

H 4.09651400 9.79334300 -3.59846100

C -1.11358900 10.74088500 -4.51631700

C -2.17824500 10.72087500 -5.43787900

C -1.30737300 11.44482800 -3.31079400

C -3.37085300 11.39492400 -5.18760300

H -2.05763100 10.16785200 -6.36600100

C -2.50271900 12.11333700 -3.05885900

H -0.52364100 11.46124200 -2.56477300

C -3.53926900 12.09990400 -3.99502600

H -4.17037800 11.36606800 -5.92275600

H -2.62675000 12.64594200 -2.11985300

H -4.46838300 12.62503400 -3.79350100

**TS-5 (in TFE)**

Rh 1.71233000 7.66588200 -2.01649600

C 4.52673900 10.32323700 -5.32033000

C 3.74032700 10.00082600 -4.19798800

C 4.32050600 10.20195500 -2.93130300

C 5.61811700 10.71442700 -2.80707200

C 6.36505700 11.02623400 -3.93689400

C 5.81602900 10.83200000 -5.20764400

H 4.09267000 10.16273700 -6.30247900

H 6.01745900 10.86485000 -1.80885000

H 7.36933200 11.42349900 -3.82379000

H 6.38727800 11.07021100 -6.09931500

O 3.71931200 9.92983400 -1.71467200

N 2.31572900 9.66272600 -1.77967500

C 1.57382600 10.77988100 -1.71944200

O 0.33026700 10.72289700 -1.87183400

C 2.25109000 12.10663600 -1.42469100

H 1.48529800 12.88337100 -1.40745800

H 2.75612200 12.06875400 -0.45397200

H 3.00381900 12.36558500 -2.17203400

C 0.16986600 7.55457200 -0.37402800

C -0.54077300 7.42879500 -1.63643200

C -0.04615600 6.25994700 -2.28366000

C 0.96568400 5.66682700 -1.44118600

C 1.05664800 6.44463800 -0.22895800

C 1.68481700 4.38302000 -1.71469500

H 1.09270700 3.52936800 -1.36171100

H 1.86031700 4.24116400 -2.78425600

H 2.65030400 4.35204500 -1.20372700

C 1.87148300 6.11204500 0.98210800

H 1.28730400 5.50232800 1.68397400

H 2.76770200 5.54468700 0.71903900

H 2.18553100 7.01448300 1.51272100

C -0.52138500 5.70807600 -3.58953100

H 0.27790200 5.18458400 -4.12059700

H -1.33138700 4.98643000 -3.42383100

H -0.90930200 6.49534700 -4.24049800

C -1.69708100 8.26166100 -2.09195500

H -2.64232200 7.82594600 -1.74164800

H -1.63002400 9.28006000 -1.71013000

H -1.74340300 8.31520000 -3.18325000

C -0.07817400 8.59707200 0.67019000

H 0.83619600 8.83070600 1.22169100

H -0.45796400 9.52025900 0.23230500

H -0.81768700 8.23150900 1.39410700

C 1.39000900 10.02843900 -5.10317300

H 0.52118500 9.41228700 -5.33238400

C 2.40300300 9.38997600 -4.46451000

C 2.21784300 7.97246000 -4.10785000

H 1.42573700 7.45940200 -4.64551700

C 1.23279100 11.42687000 -5.50245200

C 2.06160300 12.48891300 -5.08222200

C 0.13912500 11.74435800 -6.33565000

C 1.80942000 13.79644200 -5.48918100

H 2.90079300 12.29404500 -4.42995100

C -0.10859900 13.05207700 -6.74574500

H -0.52154000 10.94463500 -6.66067500

C 0.72827300 14.08842300 -6.32591100

H 2.46098800 14.59509000 -5.14525000

H -0.95770000 13.26278200 -7.38992800

H 0.53731900 15.11056100 -6.63943700

C 3.25985500 7.18210100 -3.52589300

H 4.26344600 7.59749200 -3.48466200

H 3.27259800 7.30036200 -1.95635000

H 3.24057900 6.10709300 -3.67907600

**TS-5 (in Methanol)**

Rh 1.70961400 7.66777000 -2.01560300

C 4.52716300 10.32537600 -5.31841900

C 3.74000300 10.00214900 -4.19678800

C 4.31868300 10.20390100 -2.92936100

C 5.61543800 10.71861400 -2.80429000

C 6.36308900 11.03121400 -3.93345400

C 5.81570900 10.83574100 -5.20470100

H 4.09435700 10.16400900 -6.30099100

H 6.01390300 10.87038400 -1.80591700

H 7.36664000 11.43008600 -3.81936100

H 6.38754300 11.07430400 -6.09593200

O 3.71739600 9.92994800 -1.71391100

N 2.31240800 9.66633800 -1.78023700

C 1.57382100 10.78532900 -1.71671800

O 0.32949800 10.73272700 -1.86768700

C 2.25530800 12.11007300 -1.42187600

H 1.49213300 12.88934500 -1.40213300

H 2.76222200 12.07067300 -0.45223600

H 3.00700800 12.36760900 -2.17075300

C 0.17047300 7.55102900 -0.37026400

C -0.54350000 7.42694500 -1.63116900

C -0.04892600 6.26059800 -2.28242200

C 0.96599700 5.66692700 -1.44390300

C 1.05891600 6.44197400 -0.22993100

C 1.68460000 4.38378300 -1.72156000

H 1.09384000 3.52976200 -1.36711400

H 1.85588600 4.24303300 -2.79191800

H 2.65205500 4.35227900 -1.21434500

C 1.87704200 6.10725100 0.97836300

H 1.29490400 5.49547000 1.68014200

H 2.77304200 5.54112600 0.71186900

H 2.19165400 7.00867100 1.51044100

C -0.52711100 5.71062700 -3.58794300

H 0.27229700 5.19257000 -4.12413700

H -1.33283600 4.98447000 -3.42082400

H -0.92182500 6.49798800 -4.23469000

C -1.70381100 8.25756600 -2.08059900

H -2.64694500 7.81303900 -1.73574400

H -1.64411900 9.27235600 -1.68795900

H -1.74932200 8.32178300 -3.17139500

C -0.07632600 8.59113300 0.67659900

H 0.83847700 8.82307100 1.22813100

H -0.45627900 9.51560800 0.24137700

H -0.81569100 8.22449000 1.40016300

C 1.39246300 10.02747000 -5.10876400

H 0.52478500 9.41053000 -5.34041200

C 2.40352300 9.39029700 -4.46549600

C 2.21767600 7.97315100 -4.10757900

H 1.42638100 7.45947400 -4.64584700

C 1.23553300 11.42557700 -5.50960500

C 2.06133600 12.48872100 -5.08602600

C 0.14462100 11.74173200 -6.34700400

C 1.80905900 13.79598200 -5.49389200

H 2.89797400 12.29496900 -4.43009000

C -0.10319600 13.04922300 -6.75789600

H -0.51382600 10.94124600 -6.67470800

C 0.73069500 14.08665100 -6.33471800

H 2.45823300 14.59547300 -5.14731700

H -0.95017200 13.25890800 -7.40524100

H 0.53954300 15.10859900 -6.64882000

C 3.25872100 7.18342100 -3.52369900

H 4.26221100 7.59885400 -3.48062600

H 3.26918000 7.30239800 -1.95279200

H 3.23966800 6.10830100 -3.67597900

**TS-5 (in Dioxane)**

Rh 1.72677600 7.66767800 -1.96481700

C 4.42317600 10.51788400 -5.32158500

C 3.70948600 10.06291600 -4.19651500

C 4.31486500 10.24247500 -2.93711700

C 5.57068300 10.85589900 -2.82879400

C 6.24685800 11.29321100 -3.96056300

C 5.66765100 11.12772800 -5.22115000

H 3.96930100 10.37506700 -6.29667100

H 5.98923700 10.98514800 -1.83600600

H 7.21753100 11.76892300 -3.85682700

H 6.18132300 11.46554200 -6.11545300

O 3.79013300 9.84125300 -1.73183400

N 2.36892700 9.64371600 -1.72251600

C 1.69188300 10.77046700 -1.41010900

O 0.45052600 10.78712300 -1.35362100

C 2.48915400 12.03382300 -1.10903000

H 1.77796000 12.82072100 -0.85604900

H 3.17543100 11.87324400 -0.27201000

H 3.09325500 12.35958200 -1.96011000

C 0.20215900 7.51420500 -0.31162000

C -0.52395100 7.54808700 -1.57686600

C -0.13582300 6.40037800 -2.31949600

C 0.84994700 5.67639300 -1.55298300

C 1.00285200 6.33754400 -0.27733200

C 1.47202900 4.37210700 -1.94649500

H 0.84455300 3.52803700 -1.63353200

H 1.59802100 4.29795700 -3.03036100

H 2.45417800 4.23996200 -1.48503900

C 1.82094500 5.84642200 0.87696100

H 1.22268200 5.19708500 1.52948800

H 2.68550800 5.26923700 0.53994400

H 2.19080600 6.67558300 1.48433700

C -0.66014900 5.98772300 -3.65818600

H 0.11056400 5.50328100 -4.26476900

H -1.48085800 5.26802100 -3.54586300

H -1.04630800 6.84211700 -4.21843400

C -1.59271800 8.52948400 -1.93478300

H -2.53014100 8.28515100 -1.41706700

H -1.28055900 9.53634000 -1.65890400

H -1.79527700 8.52132000 -3.00910000

C 0.02130000 8.49346900 0.80383900

H 0.88601300 8.49879500 1.47234100

H -0.11542200 9.50099400 0.40867200

H -0.86030000 8.22720900 1.40202600

C 1.40427600 9.88397000 -5.16981400

H 0.59110500 9.20330500 -5.42007200

C 2.42419500 9.34964600 -4.45204100

C 2.30465900 7.94292200 -4.03146400

H 1.56619500 7.36894100 -4.58451800

C 1.20205300 11.25289100 -5.64467600

C 1.78555500 12.38469800 -5.04250900

C 0.33792400 11.46083700 -6.73703200

C 1.53299600 13.66182600 -5.53238400

H 2.42485800 12.25961000 -4.17888300

C 0.09117300 12.73909400 -7.23091000

H -0.13784800 10.60147100 -7.20281300

C 0.69197200 13.84781300 -6.63275800

H 1.98886400 14.51953000 -5.04607200

H -0.57402000 12.87033200 -8.07964600

H 0.49816100 14.84706100 -7.01130400

C 3.35583800 7.21707300 -3.38167600

H 4.33091700 7.68964900 -3.29870100

H 3.27936000 7.30767000 -1.81251300

H 3.40639700 6.14247500 -3.53192500

**TS-5c (in TFE)**

Rh 0.49066000 8.16494900 -2.23104200

C 2.80600800 8.05754700 -5.77903100

C 2.50286200 9.07901200 -4.87325800

C 3.46081800 9.46323800 -3.85981600

C 4.66709800 8.69105400 -3.80847300

C 4.94700700 7.69427500 -4.73063900

C 4.01936400 7.36745100 -5.73140600

H 2.08031200 7.81157600 -6.55055500

H 5.38962800 8.95601600 -3.04084400

H 5.89292300 7.16052500 -4.67438100

H 4.23627700 6.58816300 -6.45526500

O 3.26979300 10.45469600 -3.06260700

N 0.89133200 9.96561200 -1.74006800

C 0.16319600 11.07965200 -1.87297000

O -1.08717700 11.12214600 -1.72362000

C 0.94063100 12.36554800 -2.08571200

H 1.08490800 12.84862800 -1.11184400

H 1.91146300 12.17981500 -2.54070800

H 0.34524400 13.03518800 -2.71126200

C -0.19283300 6.68805200 -0.70637200

C 0.26886500 5.92762700 -1.83229700

C 1.69259200 6.14454300 -1.96853900

C 2.10938900 7.02422300 -0.92728800

C 0.94019200 7.40696500 -0.17423700

C 3.48745000 7.51672900 -0.64228400

H 3.78107000 7.23166100 0.37495300

H 4.21470000 7.10768400 -1.34363700

H 3.53151300 8.60892700 -0.69931100

C 0.94899100 8.26903300 1.04287500

H 1.22602500 7.67047800 1.91959000

H 1.67666300 9.07768500 0.94446600

H -0.03403600 8.70661100 1.22752700

C 2.57162700 5.46436100 -2.96366500

H 3.51176300 5.99846300 -3.10031000

H 2.79925700 4.44582900 -2.62437500

H 2.08403700 5.37793700 -3.93779000

C -0.53629700 4.94043000 -2.60967600

H -1.58147300 5.24656900 -2.69076000

H -0.13436300 4.79548100 -3.61439800

H -0.51176100 3.96983700 -2.09834100

C -1.55753900 6.63562600 -0.10356300

H -2.31816600 6.40127400 -0.85061200

H -1.58257000 5.84901600 0.66133900

H -1.81896500 7.57980900 0.37834200

C 0.97610500 11.08357100 -5.21138200

H -0.04322100 11.44110400 -5.07493200

C 1.18727700 9.76960200 -4.91893100

C -0.06305700 7.63253800 -4.33970100

C 0.02020000 9.01083900 -4.50075900

H -1.04597800 7.17997300 -4.27401100

H 0.75020000 6.99355100 -4.66211900

H -0.89991000 9.58083700 -4.39261200

H -0.96228200 8.67061400 -2.14233200

C 1.89751500 12.11599600 -5.66714900

C 3.23528900 11.88950500 -6.05376900

C 1.40609600 13.43767800 -5.72763900

C 4.04040500 12.94527100 -6.47135100

H 3.63874000 10.88582500 -6.04144100

C 2.21639100 14.49245900 -6.13709400

H 0.37456900 13.62820400 -5.44381600

C 3.54100500 14.25062800 -6.50949100

H 5.06703800 12.74866800 -6.76680800

H 1.81566700 15.50146400 -6.16764700

H 4.17764100 15.07044300 -6.82905600

**TS-5c (in Methanol)**

Rh 0.49748500 8.16007500 -2.24558100

C 2.82387300 8.06069500 -5.77068500

C 2.50692000 9.08272700 -4.87019200

C 3.45278200 9.47486100 -3.84898200

C 4.66203400 8.70887700 -3.78370600

C 4.95607100 7.71113500 -4.70062800

C 4.04017500 7.37722400 -5.70967200

H 2.10671000 7.80899500 -6.54828500

H 5.37646400 8.97790100 -3.01005700

H 5.90409600 7.18239700 -4.63368100

H 4.26799500 6.59709800 -6.42931000

O 3.24880300 10.46807300 -3.05619800

N 0.89939700 9.96485600 -1.76620200

C 0.16480200 11.07663200 -1.85980700

O -1.08062000 11.10920700 -1.66436600

C 0.92279300 12.37178000 -2.08486100

H 1.06470900 12.86384700 -1.11515600

H 1.89298400 12.19965400 -2.54579000

H 0.31304600 13.02877000 -2.71033300

C -0.18813800 6.70067200 -0.70632000

C 0.26339300 5.92991600 -1.82945300

C 1.68972900 6.13184600 -1.96992100

C 2.11691000 7.01382300 -0.93648100

C 0.95214900 7.41462600 -0.18455300

C 3.50064000 7.49022300 -0.65215900

H 3.79714400 7.18494900 0.35864700

H 4.22074500 7.08672500 -1.36390700

H 3.55378600 8.58273500 -0.68957400

C 0.97254900 8.28622300 1.02564300

H 1.25687100 7.69442300 1.90464200

H 1.69998700 9.09356600 0.91476400

H -0.00838400 8.72619000 1.21567800

C 2.55880500 5.43854100 -2.96483100

H 3.50287900 5.96405400 -3.10846200

H 2.77991000 4.42001100 -2.62127500

H 2.06541300 5.35116100 -3.93596000

C -0.55226200 4.94422400 -2.59778100

H -1.59410300 5.26089100 -2.68189200

H -0.15191600 4.78607200 -3.60115900

H -0.53849500 3.97812900 -2.07749100

C -1.55058200 6.66221500 -0.09729700

H -2.31649200 6.42890800 -0.83925900

H -1.57779000 5.88064600 0.67270900

H -1.80290500 7.61132500 0.37981600

C 0.96897500 11.07470200 -5.22769200

H -0.05351000 11.42633100 -5.09927200

C 1.18722100 9.76369100 -4.92908100

C -0.05066500 7.61783100 -4.35467000

C 0.02356100 8.99644300 -4.51544200

H -1.03072500 7.15906400 -4.28966300

H 0.76774300 6.98331200 -4.67300900

H -0.90151700 9.55950400 -4.41366700

H -0.95498800 8.66419400 -2.15841100

C 1.88830700 12.11172100 -5.67832900

C 3.22716600 11.88985100 -6.06383500

C 1.39425900 13.43258900 -5.73356300

C 4.03107600 12.94923000 -6.47474300

H 3.63242400 10.88674200 -6.05579000

C 2.20342300 14.49107100 -6.13601800

H 0.36181800 13.61972800 -5.45078700

C 3.52922500 14.25384000 -6.50714100

H 5.05850200 12.75600200 -6.76978200

H 1.80072100 15.49944300 -6.16215400

H 4.16478600 15.07652600 -6.82148900

**TS-5d (in TFE)**

Rh 1.79308800 7.84468700 -2.09377700

C 4.60936700 9.94016400 -5.91633500

C 3.95213300 9.68230600 -4.69671700

C 4.52670700 10.23821000 -3.54458900

C 5.68590300 11.01762300 -3.60747300

C 6.31240000 11.24761000 -4.82741100

C 5.76780700 10.70492800 -5.99369200

H 4.18576600 9.51132500 -6.81844300

H 6.07722700 11.43381100 -2.68489000

H 7.21406700 11.85114100 -4.86507500

H 6.24278300 10.87297600 -6.95516800

O 4.06983600 10.05508800 -2.24387800

N 2.66713200 9.87678200 -2.09119400

C 2.00344000 11.02734000 -1.75691700

O 0.79849600 10.96798900 -1.47127900

C 2.77286200 12.32594000 -1.76149200

H 2.08743100 13.13207000 -1.49756500

H 3.59316700 12.28569400 -1.03806900

H 3.21049400 12.52894900 -2.74308000

C 0.17072900 7.64200600 -0.49454400

C -0.59795700 7.78007900 -1.73094900

C -0.24470000 6.72760400 -2.58665900

C 0.77944700 5.93306900 -1.91152500

C 0.94606700 6.44575600 -0.57292800

C 1.32457500 4.63787300 -2.42815800

H 0.58384900 3.83775000 -2.30194400

H 1.56131000 4.69531900 -3.49452000

H 2.22865500 4.33990200 -1.89238800

C 1.70895000 5.79139100 0.53829800

H 1.07937600 5.05869300 1.06149400

H 2.58808000 5.26045600 0.16379900

H 2.04789000 6.52321400 1.27604200

C -0.80602400 6.44184500 -3.94263000

H -0.06457100 5.97123900 -4.59405000

H -1.65751100 5.75130600 -3.87452500

H -1.16031600 7.35385900 -4.43120900

C -1.63349800 8.82547000 -2.00376700

H -1.44887200 9.73369900 -1.42945500

H -1.66232900 9.09288000 -3.06405100

H -2.63112700 8.45701100 -1.73020500

C -0.01701300 8.48241100 0.73153600

H 0.87178400 8.46299000 1.36784600

H -0.22808500 9.52273400 0.47861200

H -0.85954900 8.10757600 1.32810100

C 1.69336400 9.09815900 -5.56980200

H 0.93958900 8.31900000 -5.67591600

C 2.74261700 8.81218300 -4.75551200

C 2.71247900 7.54068800 -4.00741800

H 2.09032700 6.78105600 -4.47562700

C 1.39447000 10.33303800 -6.29848300

C 1.81592900 11.60807800 -5.86770400

C 0.59124400 10.25903900 -7.45348500

C 1.47305400 12.75180300 -6.58401000

H 2.39727200 11.70095500 -4.95781500

C 0.25454400 11.40410600 -8.17283900

H 0.23773900 9.28687800 -7.78763600

C 0.69871800 12.65737700 -7.74469800

H 1.80436700 13.72402700 -6.22969000

H -0.35797400 11.31843200 -9.06606500

H 0.43437000 13.55196700 -8.30094400

C 3.61437100 7.12272500 -2.99553300

H 4.48701400 7.71914600 -2.75237600

H 2.69582400 8.86915600 -1.20397600

H 3.73265100 6.05905700 -2.81118600

**TS-5d (in Methanol)**

Rh 1.78635300 7.84363600 -2.09510700

C 4.63808800 9.92719400 -5.89647200

C 3.96440700 9.67976400 -4.68382500

C 4.52844900 10.23733400 -3.52705400

C 5.69431200 11.00740700 -3.57897000

C 6.33722500 11.22694400 -4.79231600

C 5.80287100 10.68346300 -5.96299500

H 4.22192000 9.49763100 -6.80178700

H 6.07727100 11.42619300 -2.65401000

H 7.24360400 11.82389300 -4.82158600

H 6.29051900 10.84460400 -6.91931400

O 4.04788700 10.07036100 -2.23298200

N 2.64408900 9.87923600 -2.11202100

C 1.94994500 11.03058000 -1.85369500

O 0.73105100 10.96281500 -1.63507900

C 2.70073600 12.33989000 -1.86211200

H 1.99627300 13.14151500 -1.63751800

H 3.49976200 12.32846700 -1.11460200

H 3.16347900 12.52781000 -2.83525800

C 0.18348500 7.62774000 -0.47562500

C -0.60607300 7.76806700 -1.69839200

C -0.25832500 6.72526000 -2.56797500

C 0.77835100 5.93067700 -1.91263200

C 0.96216500 6.43502100 -0.57221200

C 1.31885800 4.63969100 -2.44464000

H 0.58130100 3.83747800 -2.31298900

H 1.54081200 4.70396400 -3.51378900

H 2.23057500 4.33997300 -1.92304400

C 1.74478700 5.77417600 0.52146500

H 1.12712400 5.03196500 1.04544700

H 2.62155700 5.25219700 0.12903700

H 2.09042200 6.49959600 1.26237600

C -0.83518400 6.44604500 -3.91878900

H -0.09259100 6.00504900 -4.58962100

H -1.66745700 5.73252300 -3.84889100

H -1.22025700 7.35637800 -4.38666900

C -1.66426400 8.79911100 -1.93651800

H -1.48879500 9.70095400 -1.34923600

H -1.71793000 9.08393800 -2.99131300

H -2.64899300 8.40495700 -1.65212600

C 0.02011700 8.47537200 0.74927000

H 0.89059200 8.39691300 1.40564200

H -0.11492300 9.52829700 0.49326300

H -0.86000300 8.15886900 1.32488100

C 1.70502100 9.11214100 -5.57023700

H 0.94673000 8.33764200 -5.67929500

C 2.74924100 8.81765600 -4.75285900

C 2.70950700 7.54441100 -4.00759300

H 2.08754300 6.78700200 -4.47995400

C 1.41331000 10.34948000 -6.29760900

C 1.85248300 11.62126200 -5.87460900

C 0.59584200 10.28186300 -7.44322300

C 1.51207900 12.76706900 -6.58884500

H 2.44586000 11.71116300 -4.97254200

C 0.26172900 11.42890200 -8.16060100

H 0.22856200 9.31278700 -7.77145300

C 0.72287500 12.67858600 -7.73992300

H 1.85694000 13.73654400 -6.23985200

H -0.36239500 11.34758900 -9.04620200

H 0.46040700 13.57487900 -8.29436800

C 3.60834700 7.12488700 -2.99304800

H 4.48094000 7.72091000 -2.74798400

H 2.66570700 8.88879400 -1.20609900

H 3.72691600 6.06111900 -2.80945400

**TS-5d (in Dioxane)**

Rh 1.79873400 7.82309500 -2.09730700

C 4.60177400 9.98811000 -5.89301700

C 3.94184200 9.70361600 -4.68132900

C 4.50425400 10.24526500 -3.51492100

C 5.65645400 11.03789900 -3.56509100

C 6.28508100 11.29455600 -4.77744500

C 5.75130800 10.76590000 -5.95418600

H 4.18392200 9.57101100 -6.80276600

H 6.04028600 11.44015400 -2.63356200

H 7.18069800 11.90771100 -4.80154000

H 6.22721900 10.95513500 -6.91099600

O 4.05102200 10.03602100 -2.22398300

N 2.64500400 9.84599600 -2.07287900

C 1.97934800 10.98949800 -1.68844600

O 0.79307400 10.93087000 -1.37028900

C 2.75657100 12.28982900 -1.68772500

H 2.06564600 13.09289800 -1.43005400

H 3.56741300 12.25255300 -0.95371100

H 3.20989100 12.49679200 -2.66094900

C 0.20009100 7.64662700 -0.50062500

C -0.57529600 7.80061700 -1.73101900

C -0.25553900 6.73683800 -2.58551300

C 0.75757000 5.92209500 -1.91715000

C 0.94219500 6.42881400 -0.58016400

C 1.28140800 4.62019700 -2.44085700

H 0.53203600 3.82705000 -2.32243100

H 1.52381700 4.67720100 -3.50634900

H 2.18129400 4.30571000 -1.90743900

C 1.70994600 5.76396900 0.52075000

H 1.08582800 5.02633700 1.04351000

H 2.58999500 5.24020200 0.13874100

H 2.05521300 6.48987000 1.26089400

C -0.82356200 6.45833600 -3.94086800

H -0.08456700 5.99506000 -4.60129200

H -1.67194500 5.76367900 -3.87862900

H -1.18068400 7.37155800 -4.42401500

C -1.57216600 8.88724300 -1.97817800

H -1.19378300 9.84440700 -1.61982900

H -1.79522400 8.99007000 -3.04337500

H -2.51615800 8.67026500 -1.46033700

C 0.04126800 8.51352900 0.71055400

H 0.89914800 8.42201600 1.38207600

H -0.05412800 9.56160500 0.42483700

H -0.85484500 8.22743200 1.27768100

C 1.70322400 9.09470700 -5.58944000

H 0.95847400 8.30717100 -5.69820400

C 2.74328300 8.82115300 -4.76019500

C 2.71539000 7.54693100 -4.01692600

H 2.09616700 6.78979400 -4.49399600

C 1.39551900 10.31727200 -6.33399300

C 1.80638200 11.60118600 -5.92366300

C 0.59684100 10.22055700 -7.48955200

C 1.45726200 12.73007200 -6.65795100

H 2.38708600 11.71037100 -5.01614000

C 0.25304500 11.35061500 -8.22723800

H 0.25219500 9.24082100 -7.81069400

C 0.68649300 12.61257100 -7.81767100

H 1.78108900 13.70975300 -6.31820500

H -0.35664700 11.24640300 -9.12024900

H 0.41685500 13.49653000 -8.38788800

C 3.61672000 7.11646300 -3.01125100

H 4.48446900 7.71250100 -2.75342000

H 2.70866000 8.80411000 -1.19003200

H 3.73727700 6.05101600 -2.83960200

**TS-6 (in TFE)**

Rh 1.22760000 8.03953200 -2.39465600

C 3.86286300 10.80434800 -5.45079800

C 3.09925600 10.10525000 -4.50415200

C 3.57788600 10.04710300 -3.17051700

C 4.75943800 10.73139700 -2.82298600

C 5.49485100 11.41568900 -3.78506300

C 5.04800400 11.45251800 -5.10999200

H 3.50933600 10.83215100 -6.47672500

H 5.08791100 10.69112200 -1.78849900

H 6.41340700 11.92135500 -3.50065100

H 5.61695500 11.98037600 -5.86924800

O 2.95648200 9.38674300 -2.18144700

N 1.01978500 10.01351400 -2.28543100

C 0.67226000 10.72706800 -1.20926800

O -0.47253200 11.24454200 -1.26068800

C 1.62408600 10.95658600 -0.06608600

H 1.09112400 11.47607100 0.73341500

H 2.03101100 10.01272300 0.30227900

H 2.46457700 11.57171300 -0.40220600

C 0.03111200 7.33620200 -0.53191100

C -0.52039900 6.87996100 -1.80646500

C 0.41774000 5.96939300 -2.40994200

C 1.60174000 5.99858900 -1.61361700

C 1.33322800 6.82157100 -0.42886200

C 2.83404200 5.16992600 -1.78406300

H 2.83596300 4.34981300 -1.05499200

H 2.89311800 4.73080800 -2.78053200

H 3.73884900 5.76005800 -1.61262300

C 2.32604300 7.04657900 0.66408100

H 2.46424800 6.12804700 1.24798500

H 3.30438800 7.31905500 0.25631500

H 2.00530600 7.83489100 1.34764400

C 0.14633200 5.11991500 -3.60753600

H 1.06766100 4.75546800 -4.06401600

H -0.44746100 4.24604800 -3.31170900

H -0.42510400 5.66124200 -4.36586900

C -1.91328900 7.14455600 -2.27639600

H -2.24742300 8.14258700 -1.98184800

H -1.99091600 7.05975600 -3.36279100

H -2.60531800 6.41662100 -1.83250300

C -0.69581800 8.20997300 0.43724600

H -1.17763900 9.05360900 -0.06494300

H -1.48418400 7.63916900 0.94308800

H -0.02646100 8.60888100 1.20227300

C 0.88667400 9.99684000 -5.66355700

H 0.09567900 9.34668000 -6.03760900

C 1.85676000 9.40477300 -4.91691700

C 1.71021200 7.99927000 -4.48526200

H 0.83663800 7.54347300 -4.95749400

C 0.70375400 11.41676100 -5.97474300

C 1.12339000 12.44951400 -5.10923200

C 0.01053100 11.78062600 -7.14633900

C 0.88677600 13.78484700 -5.42479900

H 1.61954100 12.19776000 -4.17964100

C -0.22013700 13.11806200 -7.46321000

H -0.34221300 10.99853700 -7.81399800

C 0.22233500 14.12893400 -6.60617000

H 1.21463900 14.56204900 -4.73976000

H -0.74974300 13.37192200 -8.37733800

H 0.03962300 15.17190600 -6.84815200

C 2.95702600 7.14056600 -4.66932600

H 3.77040600 7.43450600 -3.99887200

H 2.74260500 6.08606900 -4.49429200

H 3.33955600 7.23092800 -5.69733100

**TS-6 (in Methanol)**

Rh 1.23253800 8.04143800 -2.39102400

C 3.86350500 10.80647100 -5.45289300

C 3.10280400 10.10769300 -4.50357400

C 3.58531600 10.04996400 -3.17104200

C 4.76682900 10.73682400 -2.82766600

C 5.49892000 11.42121300 -3.79221100

C 5.04892700 11.45609100 -5.11607800

H 3.50735400 10.83278400 -6.47796000

H 5.09918400 10.69879700 -1.79437100

H 6.41746300 11.92839300 -3.51040200

H 5.61545000 11.98362500 -5.87740700

O 2.96857700 9.38752400 -2.18037800

N 1.03575000 10.01676400 -2.27785400

C 0.68830500 10.73178500 -1.20250000

O -0.45039400 11.26195500 -1.26045500

C 1.63488800 10.94801300 -0.05224000

H 1.09941100 11.46082700 0.74989100

H 2.03871000 10.00016200 0.30894100

H 2.47753900 11.56603400 -0.37772300

C 0.02696200 7.33690600 -0.53433200

C -0.51997400 6.88455900 -1.81254700

C 0.41826900 5.97169100 -2.41255300

C 1.59843400 5.99676200 -1.61087700

C 1.32632000 6.81734600 -0.42514100

C 2.83030100 5.16689900 -1.77826500

H 2.82894100 4.34617800 -1.04985500

H 2.89210000 4.72867300 -2.77498100

H 3.73521400 5.75586900 -1.60312100

C 2.31249100 7.03426700 0.67535000

H 2.43403300 6.11622100 1.26385800

H 3.29798100 7.29244200 0.27576100

H 1.99581900 7.82890200 1.35351800

C 0.14929600 5.12357600 -3.61157400

H 1.07116100 4.75267400 -4.06175500

H -0.45206600 4.25382600 -3.31876800

H -0.41416300 5.66765700 -4.37392200

C -1.91044100 7.15181400 -2.28805600

H -2.24561600 8.14896400 -1.99159500

H -1.98319800 7.07062600 -3.37510400

H -2.60495400 6.42277200 -1.84979200

C -0.70201100 8.21077800 0.43302500

H -1.18357200 9.05318800 -0.07118500

H -1.49104500 7.63996100 0.93789900

H -0.03435200 8.61092300 1.19885300

C 0.88802100 9.99785600 -5.65980700

H 0.09705500 9.34619100 -6.03133700

C 1.85963600 9.40704500 -4.91396100

C 1.71360600 8.00176100 -4.48167800

H 0.83991600 7.54583300 -4.95337700

C 0.70191100 11.41651500 -5.97471000

C 1.12811300 12.45427600 -5.11831800

C -0.00252000 11.77401200 -7.14173700

C 0.88640600 13.78773900 -5.43813300

H 1.63434600 12.20854200 -4.19256700

C -0.23822900 13.10950900 -7.46294900

H -0.36028000 10.98822700 -7.80238100

C 0.21038900 14.12521800 -6.61485700

H 1.21964500 14.56874200 -4.76000000

H -0.77661500 13.35800100 -8.37344600

H 0.02379600 15.16675600 -6.86010600

C 2.96015800 7.14279000 -4.66603900

H 3.77445300 7.43738900 -3.99701000

H 2.74579400 6.08852300 -4.48956400

H 3.34149700 7.23178800 -5.69466000

**TS-6 (in Dioxane)**

Rh 1.21811500 8.05174700 -2.36059200

C 3.79828900 10.81379200 -5.45912700

C 3.04510400 10.10946200 -4.50859000

C 3.55890800 10.00352000 -3.18956700

C 4.76197300 10.66228300 -2.86182000

C 5.48221100 11.35628100 -3.82605100

C 5.00349700 11.43087000 -5.13745900

H 3.41561900 10.87438900 -6.47244500

H 5.11648000 10.59204500 -1.83812100

H 6.41637500 11.83981400 -3.55402800

H 5.56143500 11.96578100 -5.89957700

O 2.96364100 9.33059200 -2.19947000

N 0.96917200 9.99546500 -2.25186200

C 0.60161900 10.66993300 -1.14581800

O -0.56552700 11.09999600 -1.15138000

C 1.59750300 10.94806500 -0.04799300

H 1.06970300 11.39343900 0.79837400

H 2.11683400 10.03702800 0.25518300

H 2.35693700 11.64740900 -0.41096200

C 0.04295600 7.29358400 -0.50061700

C -0.48624200 6.82380700 -1.77918800

C 0.48114700 5.93748900 -2.36931100

C 1.66176000 6.01083200 -1.57225400

C 1.36004200 6.81956100 -0.38697000

C 2.93536400 5.24597400 -1.75170700

H 2.98670200 4.41072900 -1.04189700

H 3.02590500 4.83355600 -2.75750400

H 3.80668400 5.88230700 -1.57272000

C 2.33156100 7.06729300 0.72180000

H 2.50667000 6.14885200 1.29590900

H 3.30065900 7.39708600 0.33601300

H 1.96700400 7.82642300 1.41692400

C 0.22649900 5.07005000 -3.56048800

H 1.13147700 4.56226500 -3.89666900

H -0.50872700 4.29769000 -3.30451900

H -0.17823900 5.63622000 -4.40417700

C -1.88617900 7.04028800 -2.25781400

H -2.23496500 8.04673700 -2.01455700

H -1.95942400 6.90934000 -3.33999800

H -2.57182400 6.32348100 -1.78643500

C -0.72543900 8.13534000 0.46538500

H -1.18986800 8.99389700 -0.02767700

H -1.52706600 7.54634100 0.92739800

H -0.08931000 8.51547800 1.26765500

C 0.81133900 10.07417400 -5.63363900

H -0.01369700 9.45049700 -5.97774600

C 1.77043900 9.45753100 -4.89709000

C 1.58152400 8.05579700 -4.46555300

H 0.66248900 7.64770900 -4.89449700

C 0.69543000 11.49145500 -5.98779500

C 1.12196100 12.52022400 -5.12459400

C 0.08063800 11.85532500 -7.20001200

C 0.96708000 13.85587200 -5.48163600

H 1.55516400 12.25964500 -4.16649500

C -0.06716200 13.19322100 -7.55886900

H -0.27333600 11.07379600 -7.86771300

C 0.38112300 14.20072000 -6.70282900

H 1.29710000 14.63376400 -4.79884800

H -0.53542000 13.44989700 -8.50502300

H 0.26346600 15.24483000 -6.97786600

C 2.77959200 7.15290500 -4.74565000

H 3.64932300 7.41833400 -4.13813300

H 2.54567900 6.10411200 -4.56180400

H 3.08633800 7.24019600 -5.79823000

**TS-6d (in TFE)**

Rh 1.44216600 7.86192400 -2.38651300

C 3.97080900 9.95878500 -6.45498700

C 3.40367000 9.72769300 -5.18747200

C 4.13613300 10.16677800 -4.05762400

C 5.34755300 10.86406900 -4.23450300

C 5.88136100 11.06392900 -5.50120800

C 5.19193400 10.60083300 -6.62534400

H 3.41804000 9.61775900 -7.32451600

H 5.86203800 11.21931100 -3.34705600

H 6.82782600 11.58589600 -5.60883300

H 5.59492100 10.74835800 -7.62237900

O 3.81494500 9.94273400 -2.75777100

N 2.12997800 9.75588400 -2.48079200

C 1.90540300 10.56926400 -1.32334600

O 0.96952400 11.35320400 -1.33959000

C 2.84713900 10.38858700 -0.17557100

H 2.32777400 10.60955900 0.75809000

H 3.28029800 9.38908300 -0.15954400

H 3.66294100 11.11058100 -0.29626300

C -0.11846000 7.80176500 -0.70464200

C -0.65508600 7.16239000 -1.86631300

C 0.20898600 6.05272900 -2.16631300

C 1.19822100 5.94162200 -1.10433300

C 0.99714400 7.01121700 -0.20517700

C 2.19750300 4.83522700 -0.97147500

H 1.75641800 3.97123600 -0.45650200

H 2.54158600 4.48547500 -1.94877500

H 3.07292400 5.15070700 -0.39697300

C 1.71710100 7.23900300 1.08737800

H 1.23365700 6.68637700 1.90472700

H 2.75672600 6.90238000 1.03682300

H 1.71629800 8.29352900 1.37059300

C -0.00153300 5.04574400 -3.25546900

H 0.94790200 4.65461400 -3.63179900

H -0.58402000 4.19041700 -2.88929200

H -0.54647700 5.47802800 -4.09883800

C -1.90937800 7.54128600 -2.59234300

H -2.09810900 8.61690400 -2.53170200

H -1.85897900 7.26998000 -3.65064600

H -2.78411500 7.02987000 -2.16651100

C -0.73814400 8.96324500 0.00839900

H -1.19558100 9.66776900 -0.69116100

H -1.52564100 8.61540000 0.69010400

H -0.00758700 9.50971500 0.60845600

C 0.99892000 9.63256500 -5.82681000

H 0.09777800 9.02144700 -5.88105700

C 2.05284200 9.07793900 -5.17151000

C 2.87001300 7.09991000 -3.76683600

C 1.85689300 7.77147100 -4.50788700

H 2.86245400 6.01392800 -3.72276800

H 3.85626200 7.54633700 -3.67209000

H 1.04930600 7.18350500 -4.93873100

H 1.77510600 10.26303900 -3.29662700

C 0.86790100 10.94555300 -6.46724700

C 1.59465200 12.08904900 -6.07520700

C -0.08124700 11.09083000 -7.49830100

C 1.39720700 13.31187000 -6.71117200

H 2.30561600 12.02269700 -5.26037300

C -0.27179600 12.31355500 -8.13924300

H -0.66714200 10.22633100 -7.80002200

C 0.47062800 13.43106800 -7.75162200

H 1.96524000 14.17974600 -6.38779700

H -1.00376600 12.39470800 -8.93793000

H 0.32193500 14.38670600 -8.24583900

**TS-6d (in Methanol)**

Rh 1.44238200 7.86318800 -2.38662700

C 3.97304500 9.95511000 -6.45346900

C 3.40329500 9.72718300 -5.18647600

C 4.13519600 10.16614000 -4.05626200

C 5.34867300 10.85984700 -4.23221300

C 5.88479900 11.05685800 -5.49842800

C 5.19591300 10.59403100 -6.62295500

H 3.42092300 9.61385400 -7.32334100

H 5.86307500 11.21540400 -3.34485600

H 6.83264500 11.57650700 -5.60521400

H 5.60072500 10.73920400 -7.61962300

O 3.81155300 9.94545500 -2.75590800

N 2.13043700 9.75934400 -2.48452200

C 1.89725400 10.57908100 -1.33276900

O 0.96536600 11.36741900 -1.36245300

C 2.82632400 10.39913400 -0.17447300

H 2.29293800 10.60698500 0.75436300

H 3.26965800 9.40424200 -0.15933800

H 3.63492200 11.13201700 -0.27746200

C -0.11504600 7.79874200 -0.69961300

C -0.65432200 7.16186300 -1.86134600

C 0.20996600 6.05368200 -2.16645900

C 1.20225600 5.94086200 -1.10736900

C 1.00286500 7.00821100 -0.20527200

C 2.20182300 4.83419400 -0.97897600

H 1.76165500 3.96928200 -0.46468100

H 2.54373700 4.48605900 -1.95761100

H 3.07854100 5.14827100 -0.40562500

C 1.72552800 7.23326000 1.08629600

H 1.24458500 6.67785200 1.90330100

H 2.76549400 6.89806700 1.03271000

H 1.72407900 8.28698800 1.37240800

C -0.00339300 5.04838800 -3.25663800

H 0.94499600 4.65781300 -3.63617800

H -0.58479600 4.19241200 -2.89011700

H -0.55076200 5.48179400 -4.09786900

C -1.91169900 7.54038700 -2.58236200

H -2.10244500 8.61549900 -2.51844900

H -1.86436400 7.27188600 -3.64155400

H -2.78418500 7.02633000 -2.15500200

C -0.73447900 8.95608000 0.02024700

H -1.20350200 9.65832100 -0.67383700

H -1.51321500 8.60290400 0.70932000

H -0.00136700 9.50567000 0.61440400

C 0.99874000 9.63510500 -5.82872000

H 0.09706800 9.02482500 -5.88378700

C 2.05141700 9.07922900 -5.17243500

C 2.86783100 7.10080600 -3.76899800

C 1.85406700 7.77297700 -4.50882500

H 2.85958400 6.01481300 -3.72518000

H 3.85514900 7.54568100 -3.67788400

H 1.04588700 7.18566100 -4.93943400

H 1.77663700 10.26109700 -3.30412500

C 0.86947300 10.94798900 -6.46969700

C 1.59877700 12.09053900 -6.07950500

C -0.08058200 11.09415200 -7.49988300

C 1.40297200 13.31312200 -6.71653900

H 2.31019900 12.02386300 -5.26510300

C -0.26958600 12.31665000 -8.14174600

H -0.66850700 10.23052300 -7.80020700

C 0.47553000 13.43310900 -7.75615800

H 1.97307100 14.18023200 -6.39465300

H -1.00237500 12.39839000 -8.93965100

H 0.32818900 14.38853000 -8.25125100

**TS-6d (in Dioxane)**

Rh 1.45481700 7.86250900 -2.37834800

C 4.00009100 9.98502400 -6.43899600

C 3.43978600 9.73781800 -5.17245500

C 4.16567800 10.17872100 -4.03639100

C 5.36024900 10.90719000 -4.21679600

C 5.88739000 11.12296300 -5.48237800

C 5.20796700 10.65002800 -6.60788900

H 3.44908800 9.64135900 -7.30823600

H 5.86877400 11.26590300 -3.32791600

H 6.82251000 11.66518200 -5.58915700

H 5.60628000 10.81065400 -7.60450300

O 3.85946100 9.92601300 -2.74723300

N 2.14968700 9.74225500 -2.45540000

C 1.96218700 10.58501800 -1.30540600

O 1.09586700 11.43571200 -1.33674100

C 2.87970900 10.32288300 -0.14812900

H 2.40696300 10.65223900 0.77812200

H 3.16666300 9.27316100 -0.09811400

H 3.79290100 10.90618100 -0.30272200

C -0.12433300 7.81809900 -0.71930500

C -0.65776700 7.19170700 -1.89020300

C 0.18844600 6.06744800 -2.18194000

C 1.16229600 5.93705500 -1.11055000

C 0.96648300 7.00410900 -0.20737800

C 2.15104200 4.82219500 -0.97159400

H 1.70976500 3.96640800 -0.44341400

H 2.48829000 4.45866700 -1.94602800

H 3.03510400 5.13737900 -0.41075300

C 1.66989400 7.20096800 1.09917400

H 1.18679600 6.61836100 1.89544900

H 2.71475400 6.87999200 1.05104700

H 1.65394600 8.24541400 1.41720600

C -0.02426500 5.06886000 -3.27862000

H 0.92285400 4.67449800 -3.65795600

H -0.61277500 4.21313400 -2.92364400

H -0.56343500 5.50853200 -4.12179600

C -1.88995600 7.60325600 -2.63505200

H -2.05484300 8.68211300 -2.56966800

H -1.82542900 7.34517100 -3.69575100

H -2.78475500 7.10775600 -2.23291700

C -0.73027400 8.99292200 -0.01522600

H -1.15587400 9.71107800 -0.71999700

H -1.53590200 8.66633700 0.65556000

H 0.00136300 9.52667400 0.59478100

C 1.03327700 9.62222300 -5.80774500

H 0.13691200 9.00266700 -5.84066000

C 2.09524300 9.07850100 -5.15593900

C 2.90980100 7.11041300 -3.73145000

C 1.90504700 7.77239100 -4.48905100

H 2.91451800 6.02431200 -3.69158000

H 3.88414900 7.57085400 -3.60212900

H 1.10999300 7.17512200 -4.93002700

H 1.80217200 10.24740500 -3.27459700

C 0.87040900 10.92228900 -6.46601600

C 1.62077600 12.07432700 -6.15313000

C -0.14330600 11.04461600 -7.43647600

C 1.38002500 13.27969300 -6.80505600

H 2.38881300 12.02985800 -5.39126000

C -0.37716800 12.24940200 -8.09402400

H -0.74710100 10.17337900 -7.67750300

C 0.38763300 13.37466900 -7.78398700

H 1.96899500 14.15372900 -6.54225300

H -1.15964300 12.31052800 -8.84500800

H 0.20657200 14.31778600 -8.29092100

**TS-7 (in TFE)**

Rh -1.24387200 -0.32228100 -0.19338400

C 2.54312900 3.09080700 -0.44694300

C 2.03528700 1.84671200 -0.07425000

C 1.34334800 1.03014300 -1.01064600

C 1.17647100 1.51080500 -2.32046700

C 1.69006900 2.75710700 -2.68129900

C 2.37230700 3.55311800 -1.75510300

H 3.08556300 3.68901000 0.27949300

H 0.63927100 0.90283300 -3.04071600

H 1.55954100 3.10948300 -3.70133500

H 2.77344800 4.51779500 -2.04978000

O 0.89757700 -0.16143900 -0.60428100

N -0.92939600 -0.29358500 1.84368400

C -0.41618600 -1.22335700 2.62591400

O 0.13059800 -2.28776000 2.20601600

C -0.46894400 -0.99090500 4.13035600

H 0.53899200 -1.08141900 4.54866600

H -0.88422400 -0.01704000 4.40244700

H -1.08152500 -1.77456800 4.59041200

C -1.66980000 -2.33126300 -0.92542700

C -2.73179400 -1.91571100 -0.05252300

C -3.32204100 -0.72741700 -0.60519700

C -2.68209800 -0.47043300 -1.89333100

C -1.68200700 -1.45139100 -2.08783600

C -3.03440800 0.66770500 -2.79374400

H -3.11948100 1.59921800 -2.22596400

H -2.28304700 0.81222600 -3.57260100

H -4.00074100 0.48638100 -3.27943500

C -0.75799300 -1.60420900 -3.24974400

H -1.03341400 -2.49368300 -3.82967800

H -0.79380300 -0.74136700 -3.91685000

H 0.27286400 -1.73981400 -2.91140200

C -4.49026200 0.02792300 -0.05979700

H -4.42451700 1.08981800 -0.30830400

H -5.42554300 -0.35754700 -0.48594600

H -4.55092500 -0.06477200 1.02634800

C -3.16199600 -2.59624000 1.20407700

H -2.32920000 -3.10870300 1.68688200

H -3.59134700 -1.88692500 1.91440500

H -3.93178600 -3.34102100 0.96639700

C -0.80129200 -3.53902100 -0.78578700

H -0.76644700 -3.88640500 0.24722000

H -1.17928900 -4.35708400 -1.41249900

H 0.22199100 -3.32122600 -1.10433000

C 2.38658300 -0.17110400 1.29474500

H 1.91682400 -0.75268500 2.08090600

C 2.07557200 1.22533700 1.26444800

C 1.58357000 1.77971800 2.41012800

H 1.55511100 1.14682800 3.29508100

C 3.35471200 -0.89572900 0.53008300

C 4.30339400 -0.28234200 -0.32211100

C 3.35977800 -2.30435800 0.67012700

C 5.21695400 -1.06345800 -1.01444400

H 4.32396300 0.79563700 -0.42094800

C 4.26487000 -3.07877100 -0.04197200

H 2.62590800 -2.76521900 1.32385600

C 5.19401800 -2.45865900 -0.88436100

H 5.95213000 -0.59228700 -1.65895100

H 4.25544800 -4.15921400 0.05904400

H 5.90823500 -3.06213500 -1.43662200

C 1.08034500 3.16238800 2.58510000

H 1.03746700 3.72238400 1.65120100

H 0.07667500 3.11597300 3.02825800

H 1.70470000 3.71267500 3.30185200

C -1.76103900 4.05912100 0.35525500

H -0.82200300 4.31483600 -0.14125700

H -1.89082000 4.66582300 1.25380800

H -2.57884200 4.29076900 -0.33786300

C -1.81800200 2.57641800 0.69020400

O -1.23502100 1.81966500 -0.17350800

O -2.42118700 2.18995400 1.71453400

H -1.35216300 0.50819300 2.29988000

**TS-7 (in Methanol)**

Rh -1.24798800 -0.31696200 -0.18858100

C 2.57058200 3.07852100 -0.48620800

C 2.05381300 1.84260500 -0.09783200

C 1.34372700 1.02523100 -1.02019600

C 1.16628100 1.49891000 -2.33142600

C 1.68837700 2.73711400 -2.70762100

C 2.38992400 3.53274700 -1.79563700

H 3.12735800 3.67634600 0.22958600

H 0.61510300 0.89151500 -3.04133600

H 1.54938500 3.08311200 -3.72873800

H 2.79765300 4.49099700 -2.10208300

O 0.89295700 -0.15968000 -0.60127500

N -0.91912800 -0.31123800 1.84661500

C -0.41108400 -1.24737700 2.62533200

O 0.12182600 -2.31783700 2.20359200

C -0.45661200 -1.01444500 4.13039600

H 0.55161600 -1.11366600 4.54598500

H -0.86207500 -0.03684300 4.40395300

H -1.07485600 -1.79247500 4.59249200

C -1.69329700 -2.31799600 -0.93200600

C -2.75207800 -1.89684000 -0.05710000

C -3.33174000 -0.70160600 -0.60455100

C -2.68834600 -0.44337300 -1.89067600

C -1.69834000 -1.43335500 -2.09026200

C -3.03176300 0.70032300 -2.78733100

H -3.98951900 0.51869700 -3.28981700

H -3.13070900 1.62671700 -2.21366100

H -2.26899900 0.85464900 -3.55318800

C -0.78031900 -1.59300500 -3.25588500

H -1.06655100 -2.48023500 -3.83411300

H -0.81254300 -0.73045300 -3.92348300

H 0.25107500 -1.73855300 -2.92339500

C -4.49599000 0.05939000 -0.05863900

H -4.41752000 1.12366100 -0.29305200

H -5.43096000 -0.31093300 -0.49870900

H -4.56813600 -0.04749900 1.02541300

C -3.18953800 -2.58052100 1.19521400

H -2.36309700 -3.10614400 1.67469000

H -3.61125600 -1.87142900 1.91034500

H -3.96774900 -3.31495600 0.95256900

C -0.84182600 -3.53899000 -0.80232400

H -0.81240400 -3.89571500 0.22761900

H -1.23230900 -4.34633700 -1.43525800

H 0.18436300 -3.33459400 -1.12037200

C 2.39909300 -0.16345900 1.29261400

H 1.93085200 -0.72813500 2.09188200

C 2.10326400 1.23619700 1.24699600

C 1.64367200 1.81470200 2.39415900

H 1.62354200 1.19459600 3.28853700

C 3.35321000 -0.90685700 0.52917800

C 4.29518900 -0.31248700 -0.34429100

C 3.35372600 -2.31320700 0.69268000

C 5.19655200 -1.10930000 -1.03430200

H 4.31997900 0.76352300 -0.46143600

C 4.24662600 -3.10362500 -0.01719900

H 2.62681500 -2.76011400 1.36344700

C 5.16844800 -2.50214300 -0.88085300

H 5.92625800 -0.65246600 -1.69515900

H 4.23371400 -4.18215300 0.10229000

H 5.87312000 -3.11818800 -1.43151700

C 1.16843300 3.20841900 2.55994700

H 1.09234100 3.74821400 1.61627500

H 0.18636100 3.18884000 3.05021000

H 1.83346400 3.76568600 3.23380200

C -1.75844400 4.05981100 0.42089400

H -0.82541100 4.32288800 -0.08278800

H -1.87742300 4.65408700 1.32922900

H -2.58463400 4.30212300 -0.25861300

C -1.81443100 2.57242400 0.73500500

O -1.22877100 1.82739500 -0.13744400

O -2.42172900 2.17229800 1.75149500

H -1.33041900 0.49293700 2.30883200

**TS-7' (in TFE)**

Rh 1.38055600 -0.17778500 0.14110900

C -2.74699000 2.93041300 -0.28942600

C -1.78221300 2.08517400 -0.83578500

C -0.41110100 2.22636100 -0.49869900

C -0.04413400 3.21406800 0.43119600

C -1.01993700 4.04343900 0.98288400

C -2.36889300 3.91323800 0.62745700

H -3.78897700 2.81327700 -0.57332000

H 0.99837100 3.31245100 0.71605400

H -0.72404300 4.80755800 1.69712200

H -3.11472900 4.57527700 1.05616000

O 0.46363700 1.41702500 -1.10459100

N 0.02449800 -1.57918500 -0.53542900

C -0.07593700 -2.12409700 -1.73435400

O 0.57799200 -1.73847600 -2.74858700

C -1.03659500 -3.29419700 -1.90285100

H -0.45372500 -4.20299200 -2.09305600

H -1.67236200 -3.46175600 -1.03001200

H -1.66766900 -3.12295600 -2.78020500

C 2.94854900 -1.17249900 1.24076200

C 3.11667700 -1.47488600 -0.15799300

C 3.32370500 -0.24161100 -0.85625100

C 3.38859000 0.82696600 0.13571500

C 3.16012700 0.26104500 1.41133400

C 3.64240100 2.26054400 -0.19415100

H 3.03560000 2.57920200 -1.04610200

H 3.42251000 2.91414700 0.65197500

H 4.69535700 2.40142200 -0.46757200

C 3.09742400 0.97403100 2.72171000

H 4.02580200 0.81665900 3.28420300

H 2.95896400 2.04878500 2.58699600

H 2.27247800 0.59663300 3.33196900

C 3.57362900 -0.06316300 -2.31782400

H 3.12349800 0.86532300 -2.67960800

H 4.65174600 -0.01027100 -2.51748800

H 3.15813800 -0.89031000 -2.89444800

C 3.09198700 -2.83979700 -0.76169200

H 2.48972200 -3.52728300 -0.16457700

H 2.69053300 -2.81959100 -1.77537400

H 4.11392100 -3.23691900 -0.80349000

C 2.78950700 -2.16002100 2.35025600

H 2.26690700 -3.05670600 2.01131900

H 3.77401200 -2.46563000 2.72801600

H 2.22844000 -1.73352100 3.18428500

C -2.72554300 -0.16538700 -1.64491100

H -2.67373000 -0.86292900 -2.47762900

C -1.96914400 0.97724800 -1.79336600

C -1.02700500 0.97516900 -2.85309200

H -0.81257200 0.01531300 -3.31902600

C -3.58557000 -0.61036200 -0.57375500

C -3.57298200 -0.06816000 0.73142200

C -4.40766600 -1.73131500 -0.83240400

C -4.36646300 -0.62456000 1.72680500

H -2.92261400 0.76256800 0.96423500

C -5.21134400 -2.27250200 0.16378300

H -4.40421200 -2.16812300 -1.82674100

C -5.19336800 -1.71838100 1.44695500

H -4.34077600 -0.20701900 2.72787400

H -5.84260500 -3.12847000 -0.05335200

H -5.81442000 -2.14248800 2.23030000

C -0.49297600 2.15370000 -3.56030900

H -0.62925600 3.09215900 -3.02344400

H 0.55778700 2.00678000 -3.82534100

H -1.04400600 2.20882800 -4.51395900

C -1.39041400 0.13238700 3.57174000

H -1.82173700 1.08671600 3.26095400

H -0.69680800 0.33170600 4.39787500

H -2.17109600 -0.53794600 3.93565200

C -0.60855500 -0.51453900 2.43909800

O -0.04007200 0.32876700 1.64460300

O -0.52673400 -1.75750300 2.36519300

H -0.53814000 -1.99361600 0.19940500

**TS-7' (in Methanol)**

Rh 1.38092500 -0.17893700 0.14015600

C -2.73693300 2.94037300 -0.28476200

C -1.77552300 2.09263200 -0.83310700

C -0.40151200 2.23867000 -0.50806000

C -0.02867000 3.23481900 0.41062000

C -1.00164900 4.06583600 0.96524100

C -2.35321400 3.93001200 0.62261700

H -3.78086100 2.82080400 -0.56044600

H 1.01645800 3.33914400 0.68406900

H -0.70118100 4.83608300 1.67097100

H -3.09692000 4.59344500 1.05290300

O 0.47049000 1.42681300 -1.11492500

N 0.03162900 -1.57740500 -0.55492800

C -0.06165500 -2.12261700 -1.75433400

O 0.60043200 -1.74047800 -2.76478300

C -1.02526300 -3.28973300 -1.92777300

H -0.44477500 -4.19995400 -2.11862400

H -1.66346100 -3.45750800 -1.05679900

H -1.65412800 -3.11501400 -2.80605200

C 2.93032800 -1.17518500 1.26241300

C 3.11455600 -1.48323800 -0.13323100

C 3.33929000 -0.25373200 -0.83284300

C 3.39655400 0.81763800 0.15638300

C 3.14925700 0.25767200 1.43083800

C 3.66437300 2.24864900 -0.17306100

H 3.08482600 2.56591300 -1.04413400

H 3.42268100 2.90731300 0.66315600

H 4.72551200 2.38419000 -0.41631600

C 3.07967800 0.97567400 2.73797100

H 4.00951700 0.82848300 3.30095300

H 2.93260300 2.04874200 2.59860100

H 2.25837000 0.59397800 3.35026300

C 3.61935600 -0.08548600 -2.28994900

H 3.19212000 0.84807300 -2.66589900

H 4.70201400 -0.05031700 -2.46801700

H 3.20388200 -0.90947700 -2.87124200

C 3.09143500 -2.84963100 -0.73339000

H 2.47849200 -3.53316100 -0.14263800

H 2.70385600 -2.83049200 -1.75247500

H 4.11221800 -3.25098100 -0.76084300

C 2.75361600 -2.15784900 2.37354800

H 2.23316600 -3.05462600 2.03164500

H 3.73270700 -2.46456700 2.76425900

H 2.18461000 -1.72580500 3.19932700

C -2.72145700 -0.16188500 -1.63745000

H -2.66522100 -0.86046600 -2.46891300

C -1.96698300 0.98195200 -1.78685900

C -1.02990700 0.98126400 -2.85152900

H -0.81466100 0.02175900 -3.31768700

C -3.58463700 -0.60970800 -0.57005200

C -3.59737700 -0.05483800 0.72970900

C -4.38481500 -1.74701200 -0.82711800

C -4.39198800 -0.61597900 1.72158500

H -2.96677600 0.79120800 0.96151400

C -5.18885200 -2.29401000 0.16555100

H -4.36320100 -2.19252600 -1.81734100

C -5.19509200 -1.72786200 1.44357300

H -4.38632200 -0.18765200 2.71843300

H -5.80202600 -3.16336200 -0.05041600

H -5.81691000 -2.15594800 2.22417300

C -0.50889400 2.16059800 -3.56715300

H -0.64255600 3.09974200 -3.03081000

H 0.53877700 2.01782200 -3.84644600

H -1.07283600 2.21139500 -4.51359800

C -1.41373500 0.12961800 3.55480100

H -1.84595500 1.08481200 3.24795900

H -0.71424500 0.32829700 4.37614500

H -2.19346500 -0.53882100 3.92393300

C -0.64007000 -0.51772100 2.41651100

O -0.05440000 0.32474800 1.63367700

O -0.58049700 -1.76114500 2.32836400

H -0.53883500 -1.98954500 0.17503700

**TS-7e (in TFE)**

Rh 1.17724900 8.46703400 -0.87731700

C -3.08191100 11.72890300 -1.84836100

C -2.51686400 10.46647200 -1.61635200

C -1.32847400 10.34430700 -0.83418900

C -0.75248800 11.54197500 -0.34338800

C -1.32430600 12.78367300 -0.59469900

C -2.50198100 12.89119600 -1.34468300

H -3.99465700 11.79088200 -2.43592000

H 0.15334400 11.45972400 0.24537400

H -0.85230400 13.67824500 -0.19510900

H -2.95762300 13.85869200 -1.53152000

O -0.83672100 9.15022400 -0.54846900

N 1.43123000 10.04167500 -2.19159400

C 0.77381600 10.25342600 -3.30154600

O 0.02815400 9.34231000 -3.82588700

C 0.85257600 11.59618300 -3.98866200

H -0.10588700 12.11135300 -3.85818500

H 1.64330200 12.22788200 -3.57792200

H 1.01305700 11.45724600 -5.06162900

C 0.67343700 6.35851900 -0.37834500

C 0.72548800 6.55275200 -1.82234700

C 2.06634500 6.93591900 -2.16283000

C 2.82353900 7.05209400 -0.94588500

C 1.95255900 6.65926300 0.15354500

C 4.28452400 7.34247000 -0.82855800

H 4.63099800 7.97615900 -1.64759300

H 4.51526300 7.84503000 0.11322100

H 4.85954800 6.40762800 -0.85923100

C 2.36874600 6.61709600 1.58661500

H 2.96808600 5.71846000 1.77690300

H 2.98346200 7.48416200 1.84264000

H 1.50588800 6.59910400 2.25548000

C 2.60134500 7.16427100 -3.53815100

H 3.03147100 6.23130400 -3.92320200

H 1.81755200 7.48881000 -4.22356200

H 3.39120500 7.91856100 -3.53311200

C -0.39198100 6.23012800 -2.75881500

H -0.44036900 5.14586800 -2.92381200

H -1.35122600 6.54712300 -2.34395800

H -0.26006100 6.71749100 -3.72508500

C -0.54985600 5.94058800 0.36808300

H -0.43796200 6.09297500 1.44340600

H -1.42043600 6.50748700 0.03253600

H -0.75872800 4.87831300 0.19237200

C -3.63611700 8.16492400 -1.60294700

H -3.92591100 7.33360200 -2.24305100

C -3.10916700 9.26267900 -2.25846900

C -3.05511300 9.14395700 -3.67200300

H -3.42248100 8.20656200 -4.08792400

C -3.85088100 7.91491000 -0.19871000

C -3.72785800 8.88794000 0.81938800

C -4.18891100 6.59186400 0.16966700

C -3.92259000 8.53812000 2.14900900

H -3.49398000 9.91197200 0.56166500

C -4.36245500 6.24342200 1.50361100

H -4.29423800 5.83988100 -0.60706300

C -4.22799000 7.21701300 2.49738200

H -3.83256700 9.29531900 2.92176100

H -4.60667000 5.21998800 1.77032100

H -4.36615300 6.95030500 3.54095300

C -2.43238200 10.04752300 -4.56077000

H -2.40220700 11.09561200 -4.26421200

H -1.24984700 9.72942000 -4.33777100

H -2.62567100 9.88086700 -5.61946200

C 2.94098400 11.40983900 1.93490400

H 2.50211700 10.95386500 2.82521000

H 3.99926700 11.62223500 2.10444600

H 2.42887600 12.36292800 1.75527800

C 2.76554200 10.52677000 0.71034400

O 1.71853200 9.77158600 0.73691200

O 3.58501500 10.59843100 -0.22841400

H 2.02756000 10.79965600 -1.87793500

**TS-7e (in Methanol)**

Rh 1.17763500 8.45863300 -0.87857400

C -3.06398700 11.74300100 -1.86083700

C -2.50554800 10.47845200 -1.62428900

C -1.31773000 10.35303500 -0.84200700

C -0.73294800 11.54981400 -0.35991600

C -1.29808800 12.79373200 -0.61550500

C -2.47718500 12.90427600 -1.36270000

H -3.97675600 11.80757600 -2.44807600

H 0.17506900 11.46569100 0.22502700

H -0.81940600 13.68742900 -0.22195100

H -2.92792300 13.87340400 -1.55305200

O -0.83309500 9.15735900 -0.54845000

N 1.43986200 10.03231200 -2.19332100

C 0.78995900 10.24968900 -3.30650600

O 0.04811200 9.34091800 -3.84178300

C 0.87330400 11.59608400 -3.98595600

H -0.08908800 12.10679500 -3.86784300

H 1.65517100 12.22902400 -3.56043700

H 1.05030100 11.46380300 -5.05721100

C 0.65704500 6.35406900 -0.38101200

C 0.71940100 6.54559600 -1.82498800

C 2.06506400 6.91997900 -2.15731700

C 2.81493400 7.03255100 -0.93595300

C 1.93497700 6.64579200 0.15837000

C 4.27695100 7.31323500 -0.80916900

H 4.63610000 7.93333000 -1.63311200

H 4.50361600 7.82501500 0.12874000

H 4.84457200 6.37347000 -0.82301100

C 2.34339400 6.59748200 1.59329200

H 2.93249200 5.69224000 1.78452100

H 2.96628700 7.45730200 1.85391300

H 1.47718200 6.58664000 2.25803300

C 2.61150800 7.14134300 -3.52915100

H 3.04877100 6.20733200 -3.90351500

H 1.83303500 7.45743200 -4.22432600

H 3.39865100 7.89853500 -3.52236500

C -0.39168000 6.22309300 -2.76899400

H -0.44318300 5.13800800 -2.92755000

H -1.35375600 6.54606400 -2.36522100

H -0.24882800 6.70278700 -3.73749400

C -0.57274800 5.94487900 0.35926800

H -0.47799600 6.12702900 1.43171200

H -1.44355900 6.49379400 -0.00396600

H -0.76948700 4.87624200 0.20966200

C -3.63891100 8.18385500 -1.60816900

H -3.92966900 7.35117000 -2.24603300

C -3.10239100 9.27588500 -2.26470500

C -3.04234600 9.15350700 -3.67812600

H -3.41173400 8.21664900 -4.09347600

C -3.86268100 7.94228000 -0.20356600

C -3.74466700 8.92152400 0.80911900

C -4.20478100 6.62205700 0.17084900

C -3.94717300 8.58011800 2.13984900

H -3.50854700 9.94385200 0.54632700

C -4.38607400 6.28200900 1.50602500

H -4.30688000 5.86556800 -0.60192500

C -4.25591600 7.26144500 2.49461700

H -3.86120200 9.34206300 2.90845100

H -4.63319000 5.26053200 1.77762100

H -4.40033000 7.00129600 3.53902500

C -2.41340300 10.05295700 -4.56569700

H -2.37899800 11.10142600 -4.27103600

H -1.22995600 9.73100100 -4.33944500

H -2.60208900 9.88475400 -5.62500000

C 2.94404000 11.37448400 1.96332000

H 3.93918500 11.82250200 1.99366200

H 2.19592600 12.17566300 1.95346000

H 2.77588100 10.78054000 2.86609700

C 2.77145200 10.51895100 0.71843400

O 1.72685400 9.75974500 0.73964800

O 3.58808400 10.60359400 -0.22127500

H 2.03217300 10.79047500 -1.87265500

**TS-7e (in Dioxane)**

Rh 1.10744100 8.47327400 -0.82531500

C -3.02909000 11.81419700 -1.90275400

C -2.50821600 10.52915000 -1.67989100

C -1.32287300 10.35526400 -0.89960200

C -0.69660100 11.53043000 -0.40843200

C -1.22548000 12.78830100 -0.64939800

C -2.40539400 12.94639000 -1.39256300

H -3.94402700 11.91538900 -2.48197000

H 0.20389300 11.39871500 0.17669400

H -0.72058700 13.66307900 -0.24657400

H -2.82634700 13.93188200 -1.56489100

O -0.89521300 9.14837900 -0.62346100

N 1.41205800 9.93263900 -2.22011200

C 0.82189300 10.12654500 -3.37721700

O 0.06100700 9.27587500 -3.94300100

C 1.02723800 11.46974200 -4.06002700

H 0.13818100 12.08634300 -3.88462300

H 1.89381800 12.01000100 -3.67081600

H 1.13697800 11.33136800 -5.13900300

C 0.57095600 6.39178400 -0.22092800

C 0.62265900 6.53677900 -1.67250700

C 1.96947200 6.88192300 -2.02980300

C 2.72809700 7.04236800 -0.81820400

C 1.85359400 6.69608300 0.29661800

C 4.18467100 7.35782100 -0.70216800

H 4.52993200 7.94985800 -1.55186900

H 4.38971000 7.93174500 0.20466700

H 4.78421400 6.43857900 -0.66112600

C 2.27077100 6.75642200 1.72972100

H 3.08867200 6.05403400 1.92682400

H 2.61661900 7.76494800 1.97718500

H 1.44364900 6.51526600 2.40048900

C 2.48017600 7.06575600 -3.42133300

H 2.83206400 6.10630800 -3.82173800

H 1.69392000 7.45292500 -4.07085500

H 3.31552200 7.76855900 -3.44431600

C -0.50369400 6.27824600 -2.62079400

H -0.40694400 5.29204500 -3.09365900

H -1.45989400 6.30807500 -2.09454900

H -0.51513300 7.04945600 -3.39401300

C -0.67164800 6.07351700 0.54602500

H -0.48215700 6.03384200 1.62052400

H -1.42357300 6.84537700 0.36774600

H -1.08801500 5.10824300 0.23779400

C -3.59718900 8.20488300 -1.70617900

H -3.89211000 7.40019300 -2.37785000

C -3.11566700 9.35148800 -2.34111900

C -3.10277700 9.26728500 -3.74162700

H -3.50391400 8.35115200 -4.17507000

C -3.77187900 7.88335700 -0.32199200

C -3.51267600 8.77073000 0.75056700

C -4.21427300 6.57089100 -0.01916500

C -3.68837300 8.35024600 2.06140900

H -3.18534100 9.77925800 0.54781500

C -4.37067400 6.15308100 1.29340300

H -4.41791400 5.88316800 -0.83523400

C -4.10225800 7.04369900 2.33865600

H -3.48489200 9.03895600 2.87492300

H -4.69730000 5.14074900 1.50824400

H -4.21659200 6.71938700 3.36865800

C -2.46140600 10.18099500 -4.64814100

H -2.43546200 11.21947500 -4.31806100

H -1.35090000 9.83729400 -4.55431800

H -2.77282600 10.07136200 -5.68768300

C 3.09481600 11.25736700 1.96792700

H 2.20584000 11.65352800 2.46799100

H 3.51736600 10.48924600 2.62522900

H 3.82782800 12.05432800 1.83104900

C 2.71523000 10.63594800 0.62609800

O 1.74206000 9.78515900 0.72033100

O 3.32238600 10.96154400 -0.40478800

H 2.05963400 10.64247700 -1.88546700

**TS-7f (in Methanol)**

C -3.07290900 -1.53253300 -0.54503300

C -2.64744600 -1.33230400 0.80612700

C -1.37210800 -2.00534200 0.95854600

C -1.07408900 -2.70509900 -0.27390400

C -2.10090700 -2.40812900 -1.20307700

C 0.15945700 -3.51554200 -0.51804400

H 0.36911300 -3.61337000 -1.58586500

H 1.03220900 -3.05770500 -0.04391800

H 0.05184400 -4.52547600 -0.10342000

C -2.20959700 -2.89000600 -2.61307200

H -1.23772300 -3.19052500 -3.01095600

H -2.87865600 -3.75861200 -2.66881300

H -2.62329300 -2.11673900 -3.26623900

C -4.35030200 -1.04814400 -1.15213600

H -4.70566300 -0.14423500 -0.65175200

H -4.22405100 -0.82377100 -2.21466600

H -5.13176900 -1.81387500 -1.06450900

C -3.41369400 -0.64828300 1.89142300

H -4.04354900 0.15052500 1.49522300

H -4.06459000 -1.37238600 2.39903700

H -2.74237900 -0.21600100 2.63387400

C -0.58181200 -2.10422700 2.22089300

H -0.97578600 -2.91716600 2.84600700

H 0.46758800 -2.32188400 2.01285100

H -0.63553700 -1.17665700 2.79280400

Rh -1.20132500 -0.47065800 -0.56317100

C 2.65890100 2.96656900 -1.05385000

C 2.01739300 1.81422700 -0.57720100

C 1.39136500 0.91996400 -1.49104800

C 1.43092800 1.25390200 -2.86424400

C 2.07418400 2.40090800 -3.31775100

C 2.69877300 3.26803200 -2.41386900

H 3.14333200 3.62892600 -0.34113200

H 0.94767400 0.57593300 -3.56335700

H 2.09179600 2.62066200 -4.38247800

H 3.20756300 4.16135600 -2.76259400

O 0.84862300 -0.21503800 -1.08621500

N -1.46985500 1.41664300 -0.77242300

C -2.14636400 2.36186200 -0.18690300

O -2.37386700 2.46625600 1.08728700

C -2.67415100 3.50966200 -1.03981800

H -2.23877600 4.45653500 -0.69904100

H -2.44983700 3.37452400 -2.10034100

H -3.76043300 3.58877500 -0.91231800

C 3.15104600 -0.74230600 1.01847600

C 3.27657900 -1.84756400 1.89776100

C 3.91789100 -3.01008000 1.49267300

C 4.47442500 -3.08151400 0.21158900

C 4.39304100 -1.98607300 -0.65866200

C 3.73396000 -0.83012600 -0.27004100

H 2.85115400 -1.77733100 2.89420600

H 3.99288300 -3.85489700 2.16943000

H 4.98057900 -3.98742800 -0.10829700

H 4.84339900 -2.04340300 -1.64441400

H 3.68067800 0.01274600 -0.94487900

C 2.43632900 0.39320200 1.51537100

C 1.96466600 1.54867100 0.88275600

H 2.19972700 0.32802500 2.57508800

H 0.52064500 3.81287900 0.30960300

C 1.36507100 2.46552900 1.75524100

C 0.73288600 3.74162800 1.37539100

H 1.47591600 2.28460200 2.81801700

H 1.41006700 4.56199800 1.65799800

H -0.18340200 3.88982200 1.95429600

C -0.99750300 1.82834200 3.90044100

H -2.04281600 1.65421000 4.19334300

H -0.81538400 2.91322600 3.93581200

H -0.35287100 1.35056500 4.64570600

O -0.70761500 1.27616100 2.62812000

H -1.36427100 1.67887800 1.96357900

**TS-7f’ (in Methanol)**

C -3.69955400 0.71362100 0.17061900

C -3.23276500 -0.09021000 1.27304200

C -3.00183900 -1.43552600 0.78625600

C -3.25375200 -1.43497600 -0.62419200

C -3.70123200 -0.09772800 -0.99993500

C -3.17741400 -2.62224800 -1.53079100

H -2.82439400 -2.34140500 -2.52654700

H -2.49963100 -3.37933800 -1.12922100

H -4.16660900 -3.08353300 -1.64525900

C -4.12386300 0.31291200 -2.37291400

H -3.56527400 -0.23058300 -3.13935500

H -5.18961100 0.09606200 -2.52361600

H -3.97479800 1.38336100 -2.53468100

C -4.06469700 2.16152800 0.25234700

H -3.42090300 2.69240000 0.95923700

H -3.97432700 2.65072500 -0.72073700

H -5.10025000 2.28352700 0.59392100

C -3.13178400 0.37002700 2.69115300

H -2.78036800 1.40363300 2.74690400

H -4.11624100 0.32896400 3.17591300

H -2.44967100 -0.25406800 3.27128100

C -2.63768100 -2.63404700 1.60329000

H -3.51172700 -3.28817300 1.71585700

H -1.84194800 -3.21583900 1.13176800

H -2.30531500 -2.34919200 2.60270000

Rh -1.63912800 -0.07271000 -0.23487700

C 2.88260600 2.48578700 -0.99802400

C 1.81480300 2.04791400 -0.20126100

C 0.48941200 2.06676200 -0.71440300

C 0.31552700 2.47623100 -2.05889400

C 1.38902000 2.88770700 -2.84030500

C 2.68606600 2.90627200 -2.31089400

H 3.88480600 2.47905200 -0.57754600

H -0.69359000 2.46673300 -2.46406100

H 1.21601200 3.20395200 -3.86623900

H 3.52623100 3.23997600 -2.91208900

O -0.54968200 1.75009400 0.04391100

N -0.03284700 -0.91057200 -0.81010100

C 0.49709800 -2.09918800 -0.79543000

O 0.79803200 -2.74847100 0.28515800

C 0.85100100 -2.77369100 -2.11192400

H 0.15592000 -3.60578400 -2.28037300

H 0.77791600 -2.08492900 -2.95666600

H 1.86154300 -3.19136000 -2.06493200

C 3.66048300 -0.39553400 0.61431900

C 4.75886600 -1.07861900 1.18240300

C 5.56849400 -1.89487200 0.40212300

C 5.27723600 -2.06462200 -0.95455800

C 4.17245400 -1.42144400 -1.52401600

C 3.36791700 -0.59156200 -0.75356300

H 4.97312600 -0.94967100 2.23913700

H 6.41802500 -2.40286400 0.84731300

H 5.90464700 -2.70556900 -1.56697900

H 3.93807500 -1.57127700 -2.57289600

H 2.49660400 -0.12710200 -1.18964300

C 2.88663500 0.44313000 1.50060800

C 2.07565900 1.57997400 1.18805900

H 3.18298500 0.37710500 2.54051300

H 0.74044900 3.95764600 1.26354500

C 1.60286800 2.26639800 2.27812800

C 0.80893800 3.51479700 2.25763500

H 1.83967700 1.87141500 3.26411500

H 1.24154300 4.23752500 2.96074800

H -0.20693800 3.30846700 2.62170900

C 0.40243700 -0.70251200 2.92944900

H -0.32082300 -1.50912600 3.09504600

H -0.10957800 0.12159400 2.41629000

H 0.75769700 -0.35155400 3.90362300

O 1.50929500 -1.18324300 2.18748600

H 1.16860500 -1.73956900 1.39898500

**PC1 (in TFE)**

C 3.43922400 10.11434800 -6.98009300

C 3.32668500 9.86778900 -5.60480700

C 4.47243300 10.02007100 -4.80254000

C 5.68582800 10.42785800 -5.36647800

C 5.76898700 10.67826500 -6.73401300

C 4.64504400 10.51724500 -7.54962600

H 2.55505500 9.99471700 -7.59889800

H 6.54828500 10.54542000 -4.71764400

H 6.71517900 10.99614200 -7.16217400

H 4.70767000 10.70534900 -8.61687200

O 4.45955200 9.80683700 -3.44931400

C 0.93397400 10.28892100 -4.99042000

H 0.00832900 9.84883100 -4.62088500

C 2.01644400 9.46899100 -5.01567000

C 2.82838500 7.16216800 -4.48327800

C 1.87803400 8.11095800 -4.47793300

H 2.63818000 6.18330100 -4.05371800

H 3.80996200 7.32532200 -4.91882000

H 0.90913400 7.88465400 -4.03455400

H 3.59332700 9.46643000 -3.17681800

C 0.80128200 11.68698200 -5.40924000

C 1.87026400 12.60460500 -5.46454000

C -0.48753800 12.15231300 -5.73800400

C 1.65445300 13.92272800 -5.85860400

H 2.86675100 12.29220100 -5.17951700

C -0.69902400 13.46845200 -6.14267400

H -1.32607100 11.46324200 -5.68124800

C 0.37355700 14.36041600 -6.20842900

H 2.49103100 14.61521200 -5.88701100

H -1.70101500 13.79867300 -6.40129900

H 0.21251200 15.38887800 -6.51816500

**PC1 (in Methanol)**

C 3.94119100 9.44171200 -6.90253800

C 3.35821300 9.82642700 -5.68756800

C 4.09529900 10.66120700 -4.82586300

C 5.37040700 11.10569900 -5.18744300

C 5.92213700 10.72277800 -6.40791100

C 5.20992600 9.88665700 -7.27215700

H 3.37642800 8.79350900 -7.56597600

H 5.91068100 11.75139400 -4.50199200

H 6.91170700 11.07704200 -6.68182100

H 5.63752400 9.58417100 -8.22294600

O 3.60826300 11.08326500 -3.62050700

C 0.92214800 10.18007800 -5.17847400

H 0.00654200 9.71815800 -4.81103500

C 1.99697200 9.35524400 -5.30287200

C 2.78664300 6.99918200 -4.99263500

C 1.82229800 7.93211400 -4.99511600

H 2.55704900 5.96616900 -4.74855700

H 3.82031700 7.23240200 -5.22913900

H 0.80377200 7.63698800 -4.74499700

H 2.72844000 10.69759600 -3.47729600

C 0.79841500 11.61788400 -5.43834900

C 1.65536800 12.35140700 -6.28409800

C -0.26738300 12.30309300 -4.82096900

C 1.46268300 13.71717200 -6.47711800

H 2.46016800 11.85019800 -6.80618200

C -0.45304400 13.67047300 -5.00976100

H -0.94843800 11.74909400 -4.18028300

C 0.41540600 14.38610200 -5.83710400

H 2.13166500 14.26212400 -7.13719500

H -1.27719700 14.17623600 -4.51492600

H 0.27231300 15.45174300 -5.99045500

**PC1 (in Dioxane)**

C 3.43313600 10.12265700 -6.98519900

C 3.33191300 9.85066100 -5.61426200

C 4.48603300 9.97605700 -4.81613900

C 5.69397200 10.39484000 -5.38454300

C 5.76411200 10.67610800 -6.74574400

C 4.63443600 10.53308700 -7.55591600

H 2.54171700 10.02299500 -7.59647300

H 6.56235900 10.49257500 -4.74128500

H 6.70701400 11.00125400 -7.17554600

H 4.68899000 10.74307900 -8.61923800

O 4.48058800 9.73370200 -3.47530500

C 0.94657700 10.29784800 -4.98634300

H 0.02446600 9.86695800 -4.59668700

C 2.02259900 9.46926200 -5.01356000

C 2.81360900 7.15978800 -4.45789100

C 1.87995000 8.12535300 -4.44241000

H 2.62383000 6.19429800 -3.99998100

H 3.77648700 7.29363100 -4.94154700

H 0.92445500 7.92390900 -3.95952000

H 3.65753600 9.27839200 -3.24141000

C 0.80994100 11.69213800 -5.41347400

C 1.88208100 12.60121100 -5.51527100

C -0.48614300 12.16500800 -5.69677900

C 1.65970800 13.91748600 -5.90790200

H 2.88647400 12.28203700 -5.27040900

C -0.70455000 13.47937800 -6.10001700

H -1.32701400 11.48245200 -5.60553200

C 0.37033600 14.36247400 -6.21056900

H 2.49959100 14.60304800 -5.97292600

H -1.71319700 13.81523500 -6.32215800

H 0.20490700 15.39039700 -6.51924800

**PC2 (in Methanol)**

C 3.37188700 1.87071400 0.59189600

C 2.10543800 1.51660800 0.09621400

C 0.97196500 1.85573400 0.86912500

C 1.13491400 2.48313000 2.11297700

C 2.40305900 2.80205200 2.58770400

C 3.53327600 2.50498300 1.81994700

H 4.24180400 1.62755500 -0.01024700

H 0.24405500 2.71614500 2.68865000

H 2.50745300 3.29220600 3.55156900

H 4.52621100 2.76516100 2.17333000

O -0.30662100 1.58888700 0.48689600

C 3.44500500 -1.21607600 -0.55493400

C 4.48140400 -1.99518400 -1.10033500

C 5.24754400 -2.83864900 -0.29676900

C 4.97766800 -2.93708800 1.06957500

C 3.93125000 -2.19102300 1.62067400

C 3.17280500 -1.33967900 0.82085500

H 4.68810400 -1.92806500 -2.16534000

H 6.05001800 -3.42246800 -0.73869000

H 5.56884700 -3.59663700 1.69826300

H 3.70144200 -2.27675500 2.67903500

H 2.35654900 -0.77858700 1.26009500

C 2.66791900 -0.35673100 -1.46270300

C 2.01922400 0.79826200 -1.20644100

H 2.62843100 -0.72081800 -2.48882700

H 0.89536000 3.41471300 -1.52527500

C 1.19565900 1.41394700 -2.32556600

C 1.32108700 2.93109100 -2.40906600

H 1.50312700 0.96859000 -3.28009400

H 0.82225000 3.32417800 -3.29847600

H 2.37942800 3.20258500 -2.47144200

C -1.14902600 1.46070600 -2.99423600

H -2.07004400 0.91360300 -2.78014000

H -0.84100300 1.25669800 -4.02719800

H -1.33536700 2.53360700 -2.87604900

O -0.16906100 0.97912900 -2.07372100

H -0.34255400 1.29304900 -0.45983600

**PC2’ (in Methanol)**

C 2.92823900 0.57710300 1.81032300

C 1.86591200 -0.22913400 1.37558700

C 0.55226100 0.19718500 1.65517000

C 0.31597500 1.39786600 2.33095000

C 1.38938000 2.18398100 2.74404700

C 2.70048600 1.77274100 2.49152400

H 3.94434300 0.25134900 1.61879200

H -0.70984500 1.69536600 2.52559200

H 1.19934600 3.11500400 3.27048700

H 3.53999400 2.37653300 2.82215800

O -0.54153300 -0.53882100 1.28664700

C 3.96681700 -0.74066400 -0.89665200

C 5.02827700 -1.52130700 -0.42120200

C 6.32766100 -1.01686000 -0.40662700

C 6.58133100 0.27911300 -0.86686700

C 5.52746500 1.05879100 -1.34470400

C 4.22515400 0.55219000 -1.35972300

H 4.83013600 -2.52390700 -0.05154300

H 7.14222600 -1.63324100 -0.03686500

H 7.59292800 0.67445000 -0.85392700

H 5.71547300 2.06687100 -1.70368900

H 3.40752000 1.16153200 -1.72619500

C 2.55942700 -1.30929700 -0.84652300

C 2.07971000 -1.48047600 0.59336800

H 2.56786000 -2.30183500 -1.31867100

H 1.37523100 -2.16260000 3.12512300

C 1.83105000 -2.70790000 1.08093900

C 1.40698300 -3.04304200 2.47852200

H 1.97044100 -3.55546600 0.40982500

H 2.10043000 -3.76990800 2.91962100

H 0.41635200 -3.51480400 2.48726800

C 0.52287900 -1.08023200 -2.06693800

H -0.04236600 -0.32329000 -2.61634100

H -0.10916500 -1.47190500 -1.26101200

H 0.77279000 -1.90728000 -2.74637200

O 1.69436800 -0.44357300 -1.57855000

H -0.23443700 -1.36029900 0.86746600

**PC3 (in TFE)**

C -1.80178200 -2.06898800 0.15780200

C -0.43411000 -2.52662900 0.18925100

C 0.16154800 -2.28059300 -1.08527400

C -0.85307700 -1.65663700 -1.91357100

C -2.07567800 -1.58782300 -1.15746300

C -0.70134300 -1.32053400 -3.35671700

H -1.33521300 -0.47642000 -3.63673600

H 0.33468300 -1.07863500 -3.60021200

H -0.99954300 -2.18451700 -3.96392900

C -3.39566000 -1.20776700 -1.72928800

H -3.29815700 -0.35790100 -2.40800700

H -3.78282300 -2.05476400 -2.31024000

H -4.11708900 -0.95573800 -0.95412300

C -2.78706800 -2.30473500 1.25245900

H -3.73154300 -1.79999600 1.05230000

H -2.98110500 -3.38184300 1.33536600

H -2.41261500 -1.95894700 2.21767700

C 0.15947600 -3.27606300 1.32913400

H -0.13217400 -2.84383200 2.28991900

H -0.22274500 -4.30475600 1.30731000

H 1.24596800 -3.32279900 1.26995200

C 1.51140800 -2.69819000 -1.56023800

H 1.41062300 -3.55650300 -2.23609200

H 1.99946100 -1.89494500 -2.11777600

H 2.16101300 -2.98684200 -0.73407500

Rh -0.37052000 -0.23880900 -0.33404700

C 2.01524100 3.32781400 0.73482700

C 1.44166900 2.16672700 0.21654100

C 1.40682000 1.93925500 -1.17879400

C 1.96909000 2.90919800 -2.03225800

C 2.54899700 4.05986600 -1.49964700

C 2.57816700 4.28014200 -0.11829300

H 2.02669400 3.48204300 1.81040000

H 1.94889700 2.73830500 -3.10485400

H 2.97835100 4.79679200 -2.17354800

H 3.02695400 5.18106300 0.28783500

O 0.89791900 0.81036500 -1.65959600

N -1.61573100 1.30868000 -0.93435400

C -2.75030400 1.48440800 -0.31268500

O -3.27180200 0.85777300 0.68410300

C -3.59851600 2.62322300 -0.88919400

H -3.79654000 3.35586300 -0.09820500

H -3.12026400 3.12744400 -1.73149000

H -4.56358100 2.21791600 -1.21583200

C 2.71419700 -0.68782300 0.79851400

C 3.25665600 -1.66328600 1.66395300

C 4.53918300 -2.16891500 1.47458500

C 5.31512100 -1.71842200 0.40403600

C 4.79479200 -0.75503900 -0.46225300

C 3.51321800 -0.24214400 -0.27269000

H 2.66437500 -2.01079700 2.50442200

H 4.93151000 -2.91258600 2.16181300

H 6.31443100 -2.11348900 0.24706900

H 5.38831100 -0.40030500 -1.29985900

H 3.13283300 0.47751600 -0.97829400

C 1.35813900 -0.22761600 1.12457700

C 0.83492900 1.11395200 1.07482700

H 0.91181300 -0.81841600 1.91898900

H -0.49071600 3.25346900 2.69401500

C -0.32507700 1.34998900 1.80612200

C -0.99356500 2.67355600 1.90648800

H -0.65707800 0.57239200 2.48735900

H -2.03641300 2.56275400 2.19821200

H -0.91712900 3.24309700 0.97925400

C -4.25342700 0.15943600 3.64245300

H -4.27074400 0.00704300 4.72688400

H -4.85138900 1.05346000 3.41353800

H -4.74290600 -0.70627400 3.17210300

O -2.90141700 0.30447200 3.23423100

H -2.92041400 0.48809000 2.26011700

**PC3 (in Methanol)**

C 4.68985900 9.43790400 -5.52995900

C 3.40818300 9.49481500 -4.97162500

C 3.26117800 9.32270800 -3.58462500

C 4.33601700 9.10149000 -2.73450300

C 5.61047100 9.04957400 -3.31239500

C 5.78699900 9.21340200 -4.69100900

H 4.83845000 9.55764700 -6.59624200

H 4.18480900 8.97380400 -1.66785400

H 6.47339100 8.87528200 -2.67634000

H 6.78498400 9.16410700 -5.11540200

O 1.96233000 9.39987300 -3.16605800

C 1.10924000 9.59369900 -4.33873300

H 0.49698400 8.69013100 -4.43325600

C 2.07374000 9.72320900 -5.52452400

C 1.67811200 10.00751900 -6.77521600

H 0.61235400 10.15695900 -6.93870100

C 0.21921500 10.79303800 -4.13193400

C 0.75542100 12.00158600 -3.66865100

C -1.13927600 10.72076500 -4.45099200

C -0.06126200 13.12140900 -3.52544700

H 1.81091300 12.05949300 -3.41983300

C -1.95652800 11.84671900 -4.31733300

H -1.55740100 9.78130300 -4.80235800

C -1.41944300 13.04768800 -3.85288900

H 0.35984300 14.05404300 -3.16092400

H -3.01123500 11.78144300 -4.56871700

H -2.05383300 13.92240600 -3.74251300

C 2.57790200 10.14447300 -7.97038800

H 3.05239000 9.19087400 -8.23781200

H 2.01710900 10.48751400 -8.84369100

H 3.38880600 10.86118000 -7.79116600

**PC3’ (in TFE)**

C 3.86464800 11.69861800 -4.41441300

C 3.02963500 10.57372700 -4.40377200

C 3.25522800 9.57768600 -3.43606200

C 4.24560400 9.67619800 -2.46523600

C 5.05193500 10.81739300 -2.48365000

C 4.87031000 11.81327000 -3.45236800

H 3.74060800 12.47363100 -5.16025500

H 4.38300000 8.88770500 -1.73296200

H 5.83425900 10.92689200 -1.73830400

H 5.51718500 12.68507500 -3.45588600

O 2.42119900 8.50576600 -3.55523700

C 1.28965300 10.63397200 -6.31569800

H 0.57926000 9.98294000 -6.82491400

C 1.93626400 10.10157900 -5.25793600

C 1.60904900 8.69414400 -4.75752800

H 0.56491300 8.62118100 -4.43756500

C 1.40866200 11.97968800 -6.89566900

C 1.49548900 13.13751300 -6.10194100

C 1.35959000 12.13400900 -8.29340800

C 1.57409400 14.40048700 -6.68703800

H 1.47955400 13.04443100 -5.02123100

C 1.44254300 13.39647500 -8.87852100

H 1.26528300 11.25120000 -8.92021300

C 1.55646300 14.53585100 -8.07777200

H 1.63783800 15.28184000 -6.05506100

H 1.41518900 13.49181300 -9.96035800

H 1.61806500 15.52023300 -8.53238200

C 1.94303800 7.58470600 -5.74317500

H 2.99504400 7.64252200 -6.03926000

H 1.75256000 6.60463300 -5.29547300

H 1.32327100 7.68255300 -6.63948300

**PC3’ (in Methanol)**

C 3.86648000 11.69716600 -4.41380100

C 3.03032000 10.57311400 -4.40314100

C 3.25374400 9.57717500 -3.43461900

C 4.24316800 9.67612000 -2.46263200

C 5.05071200 10.81645900 -2.48112300

C 4.87133800 11.81170600 -3.45090900

H 3.74403000 12.47166200 -5.16047600

H 4.37942400 8.88840900 -1.72926800

H 5.83233800 10.92562700 -1.73496400

H 5.51930300 12.68272700 -3.45467300

O 2.41996300 8.50612200 -3.55427200

C 1.29031800 10.63481700 -6.31535300

H 0.57964600 9.98390900 -6.82434800

C 1.93717500 10.10184200 -5.25797600

C 1.60970600 8.69413800 -4.75862500

H 0.56504700 8.62038200 -4.44065700

C 1.40883500 11.98044100 -6.89562900

C 1.49855900 13.13862900 -6.10268100

C 1.35576800 12.13422800 -8.29335600

C 1.57612600 14.40130800 -6.68864900

H 1.48555900 13.04629900 -5.02186200

C 1.43769900 13.39637200 -8.87932400

H 1.25888200 11.25117900 -8.91945800

C 1.55454200 14.53607400 -8.07940400

H 1.64227000 15.28294000 -6.05726600

H 1.40717000 13.49120200 -9.96114700

H 1.61533200 15.52023200 -8.53466600

C 1.94579200 7.58534900 -5.74428000

H 2.99863100 7.64256800 -6.03764600

H 1.75315700 6.60479600 -5.29849300

H 1.32840000 7.68474200 -6.64207400
