# Supplementary material for: Chemodivergent assembly of ortho-functionalized phenols with tunable selectivity via rhodium(III)-catalyzed and solvent-controlled C-H activation
Source: Commun Chem. 2021 Jun 3;4:81. doi: 10.1038/s42004-021-00518-x (PMC9814747; doi:10.1038/s42004-021-00518-x)
Supplement: Supplementary file 5 — Supplementary Data 3 [file 42004_2021_518_MOESM5_ESM.docx]

**Supplementary Data 3: ^1^H, ^13^C and ^19^F NMR spectra**

**2i**

**
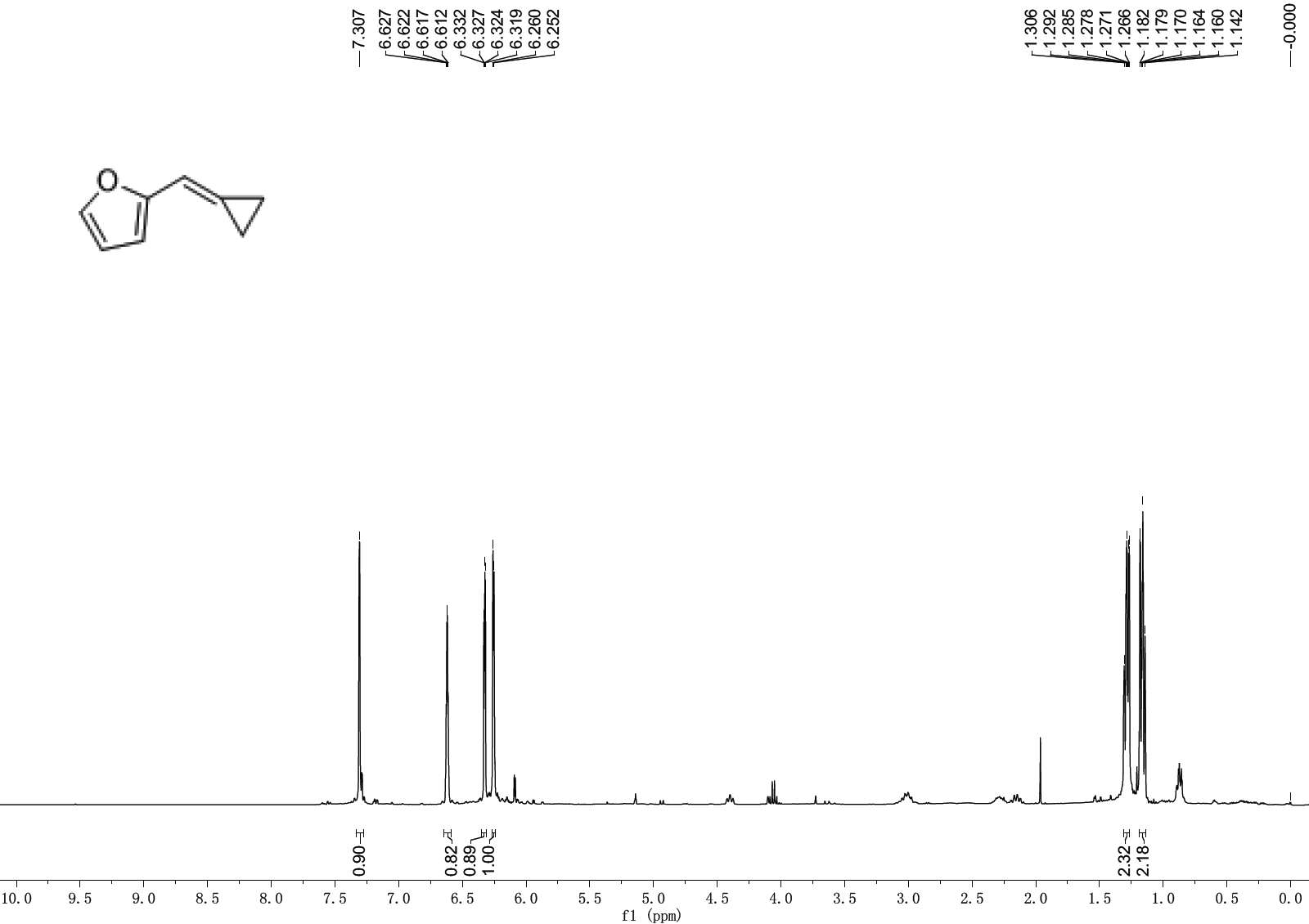
**

**Supplementary Figure 4.** ^1^H-NMR spectrum of **2i**

**
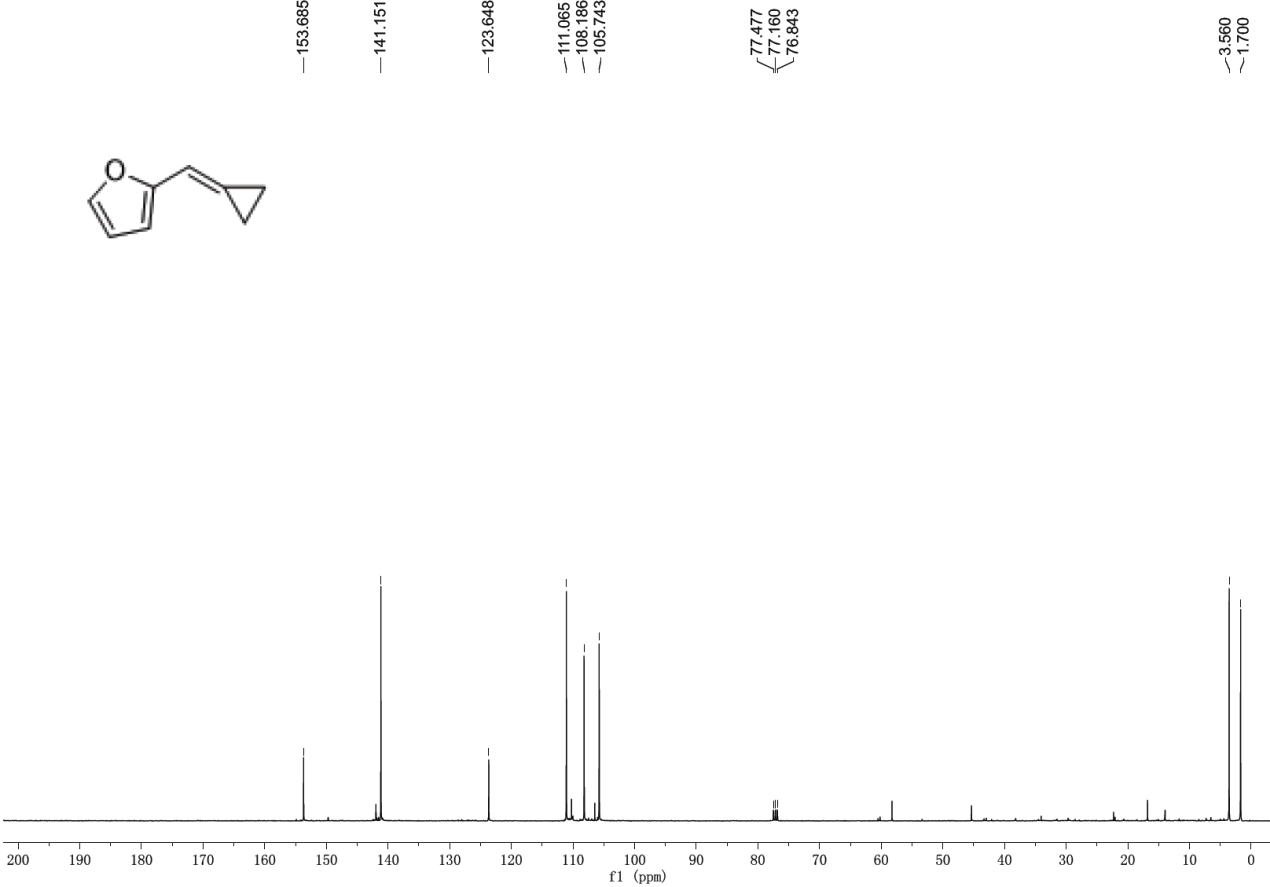
**

**Supplementary Figure 5.** ^13^C-NMR spectrum of **2i**

**2k**

**
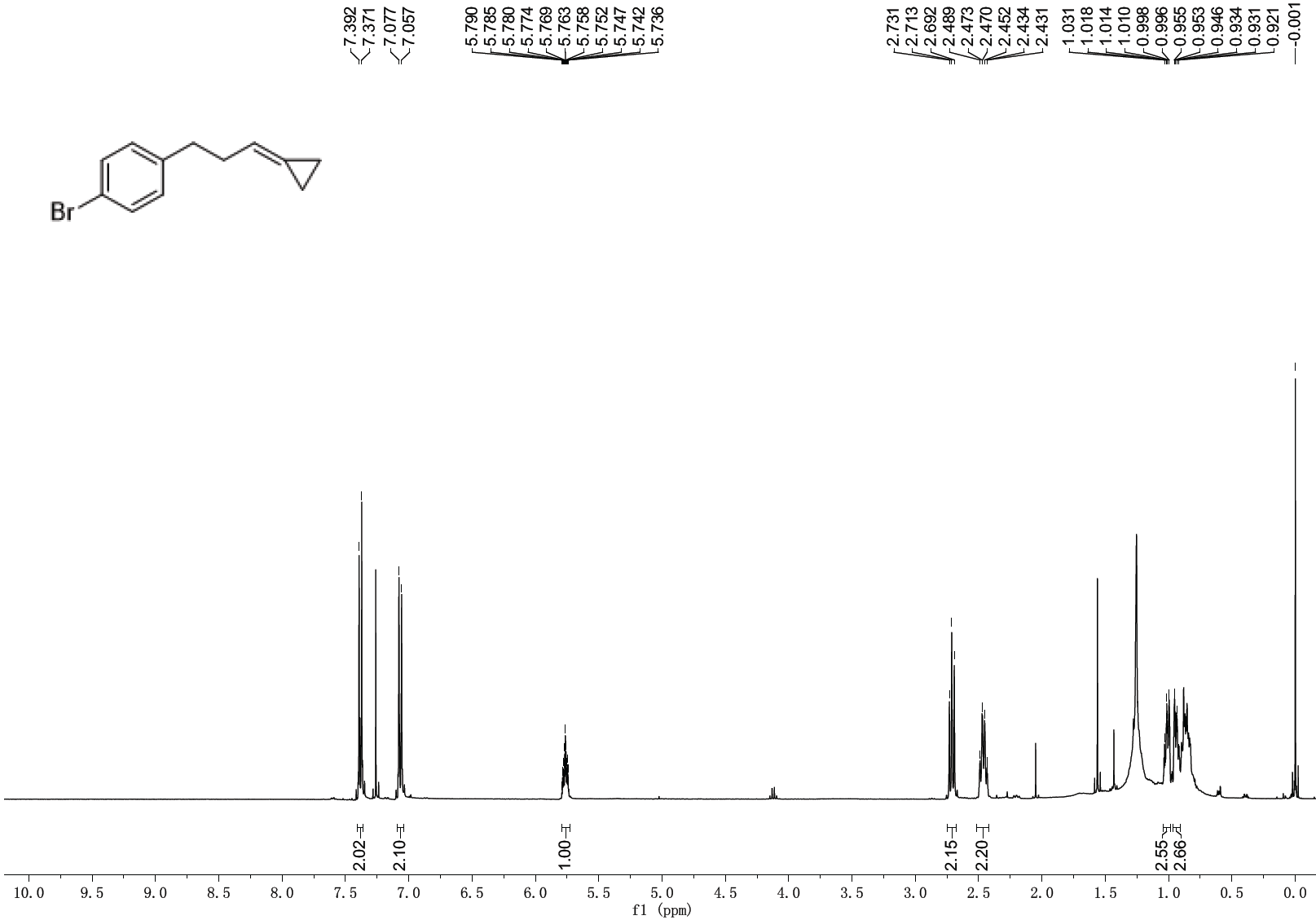
**

**Supplementary Figure 6.** ^1^H-NMR spectrum of **2k**

**
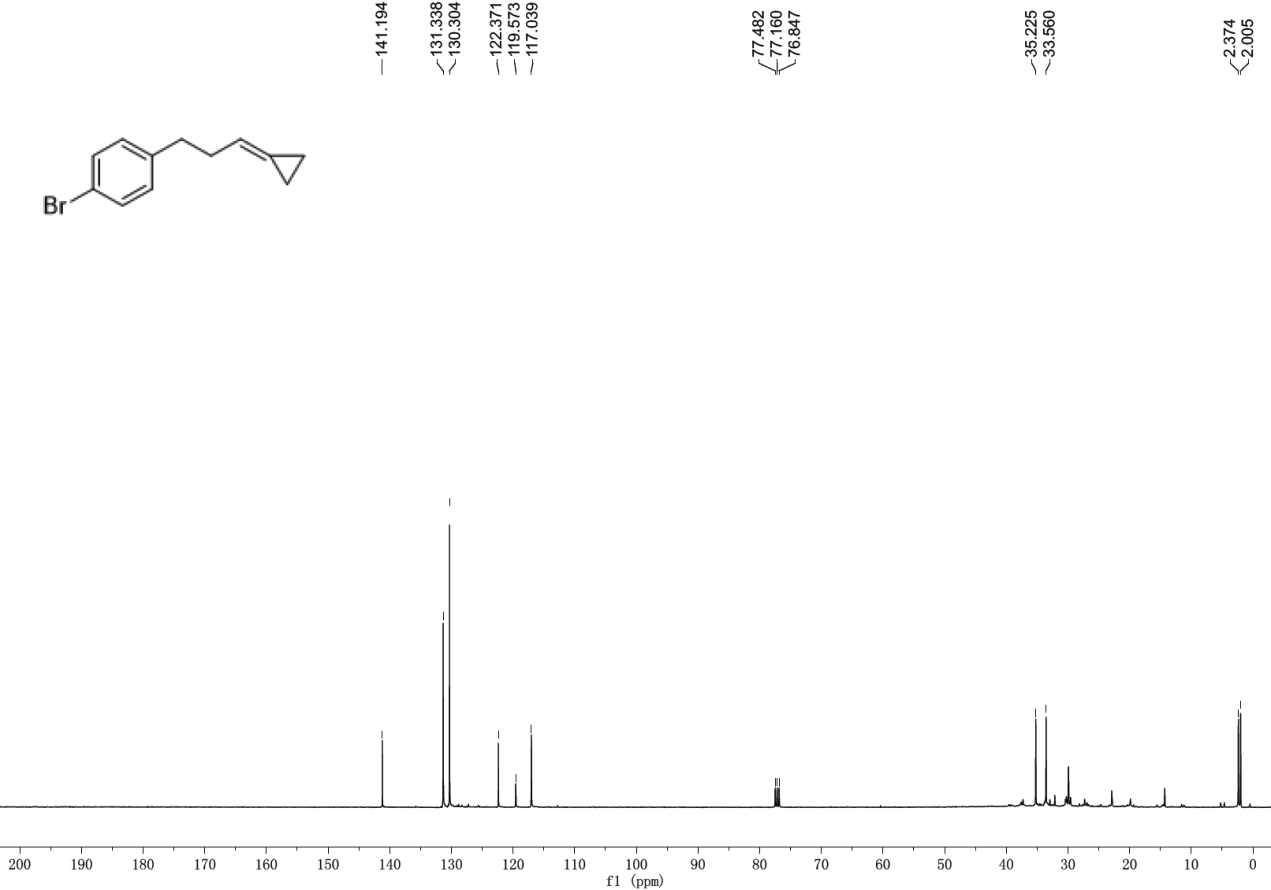
**

**Supplementary Figure 7.** ^13^C-NMR spectrum of **2k**

**2l**

**
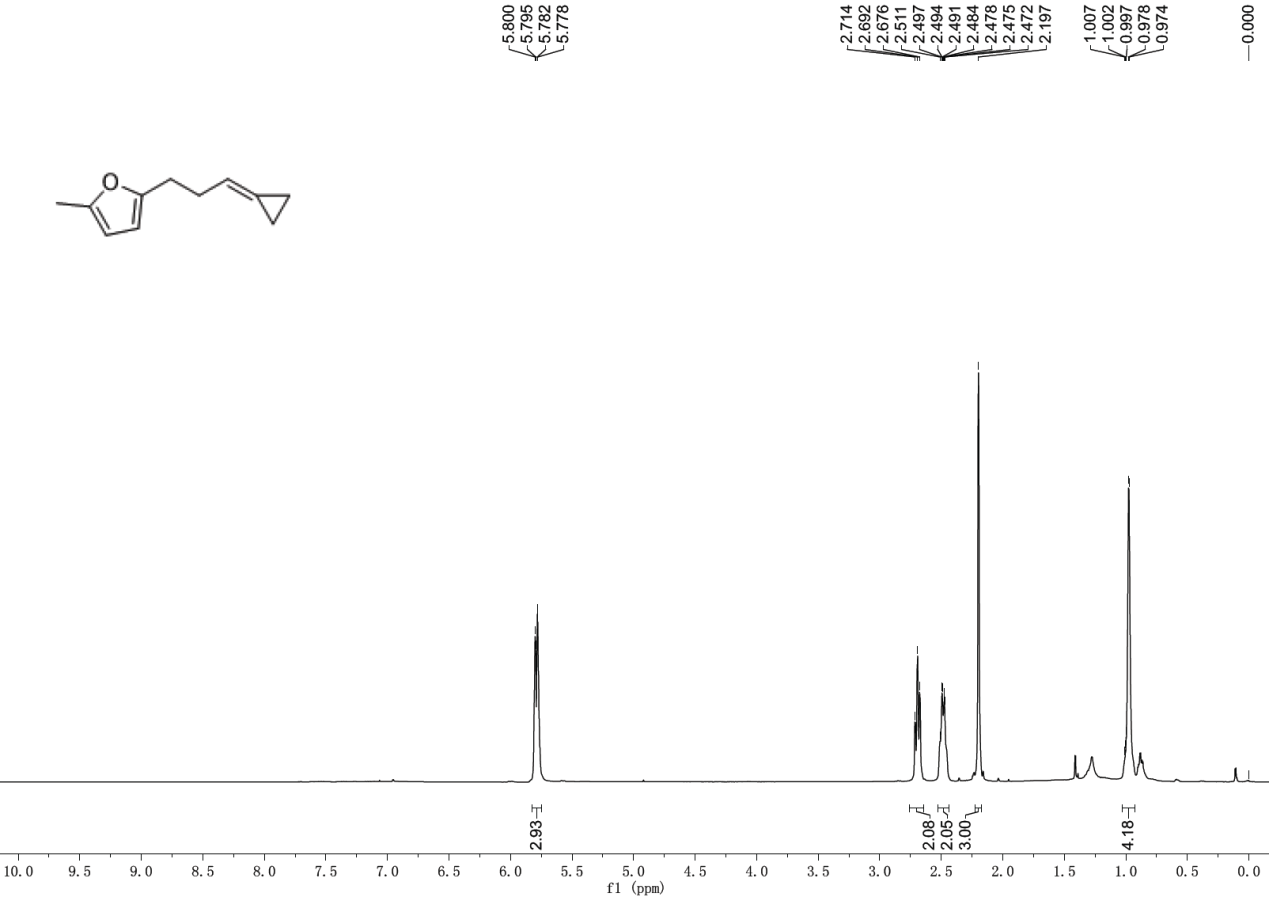
**

**Supplementary Figure 8.** ^1^H-NMR spectrum of **2l**

**
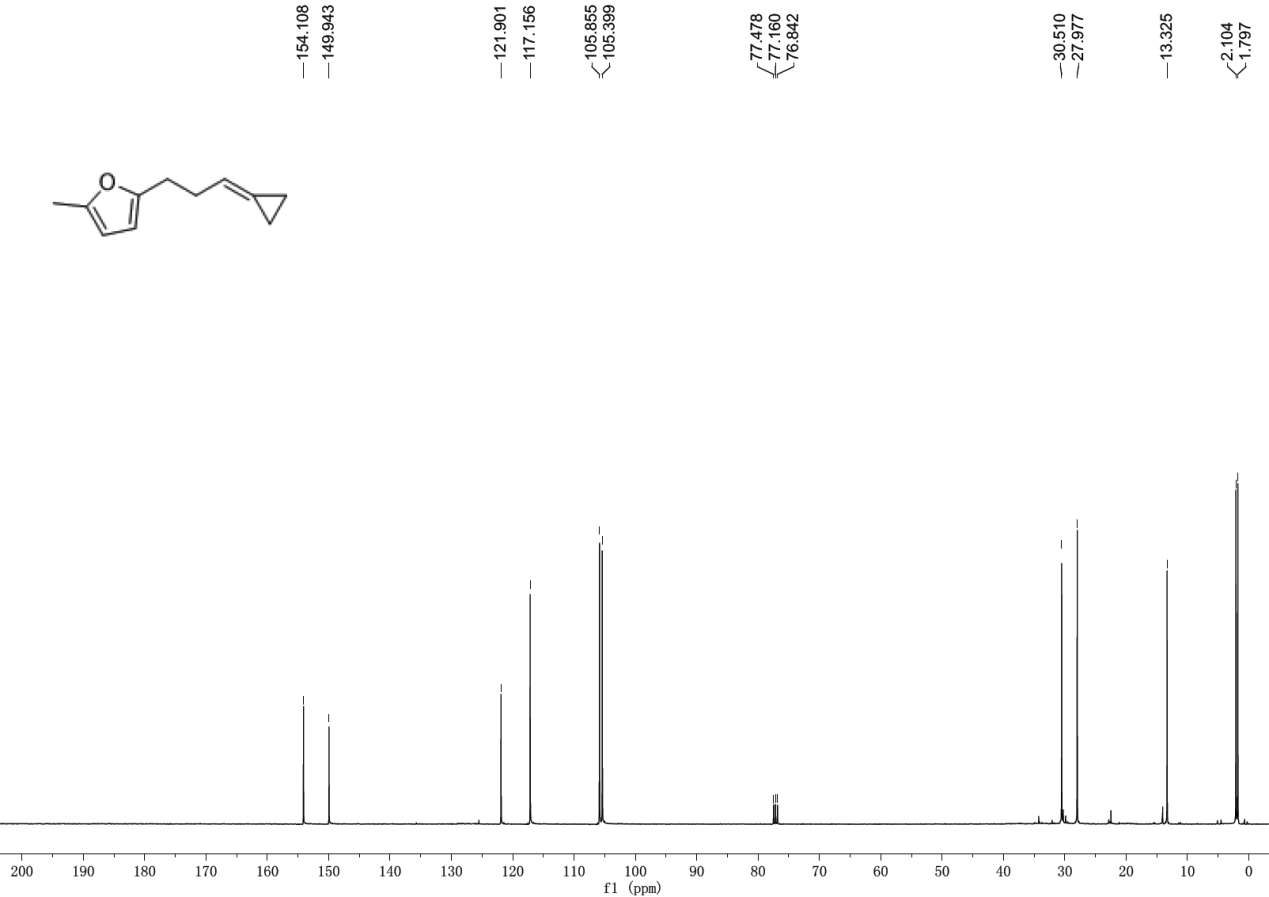
**

**Supplementary Figure 9.** ^13^C-NMR spectrum of **2l**

**3a**


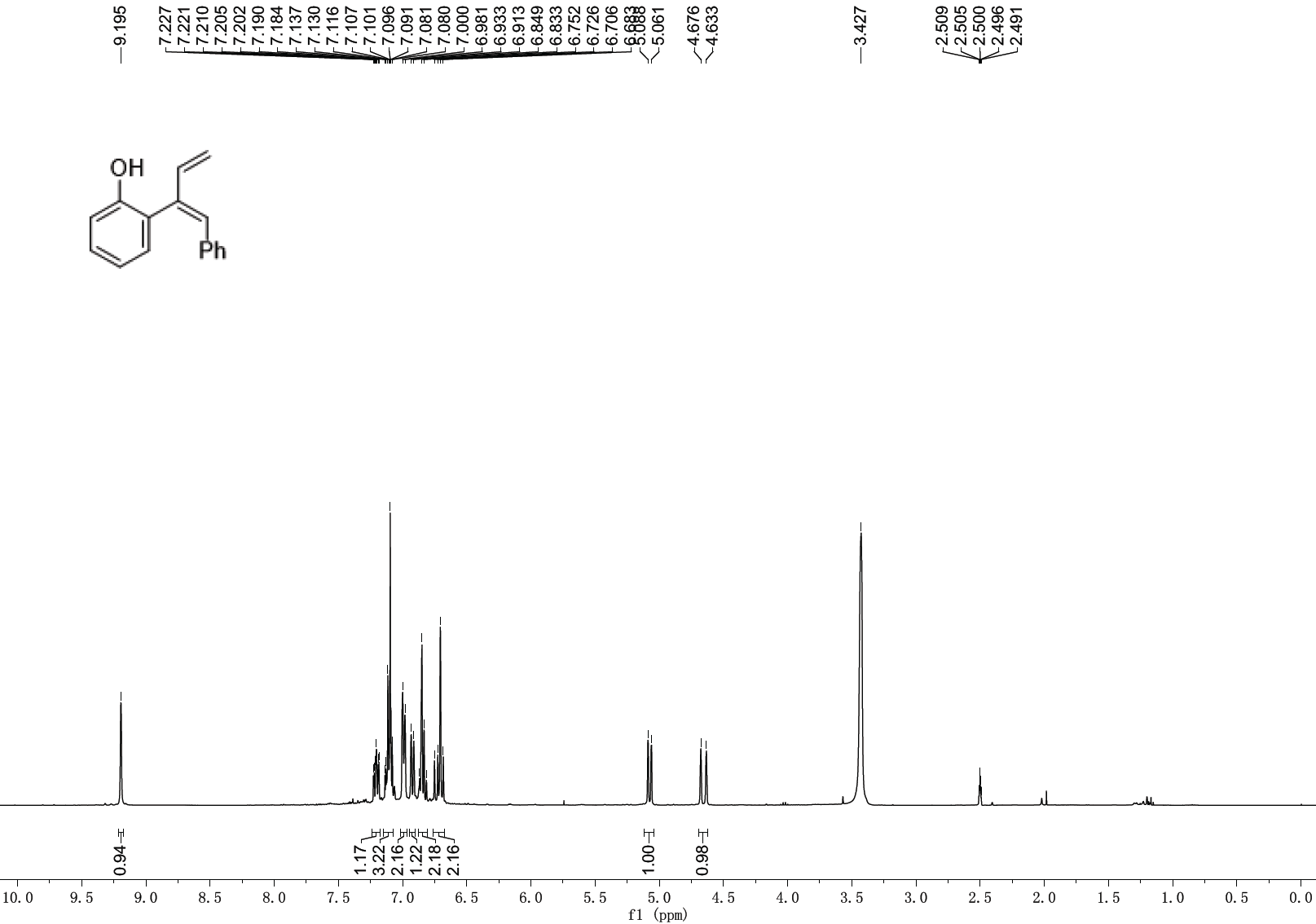


**Supplementary Figure 10.** ^1^H-NMR spectrum of **3a**


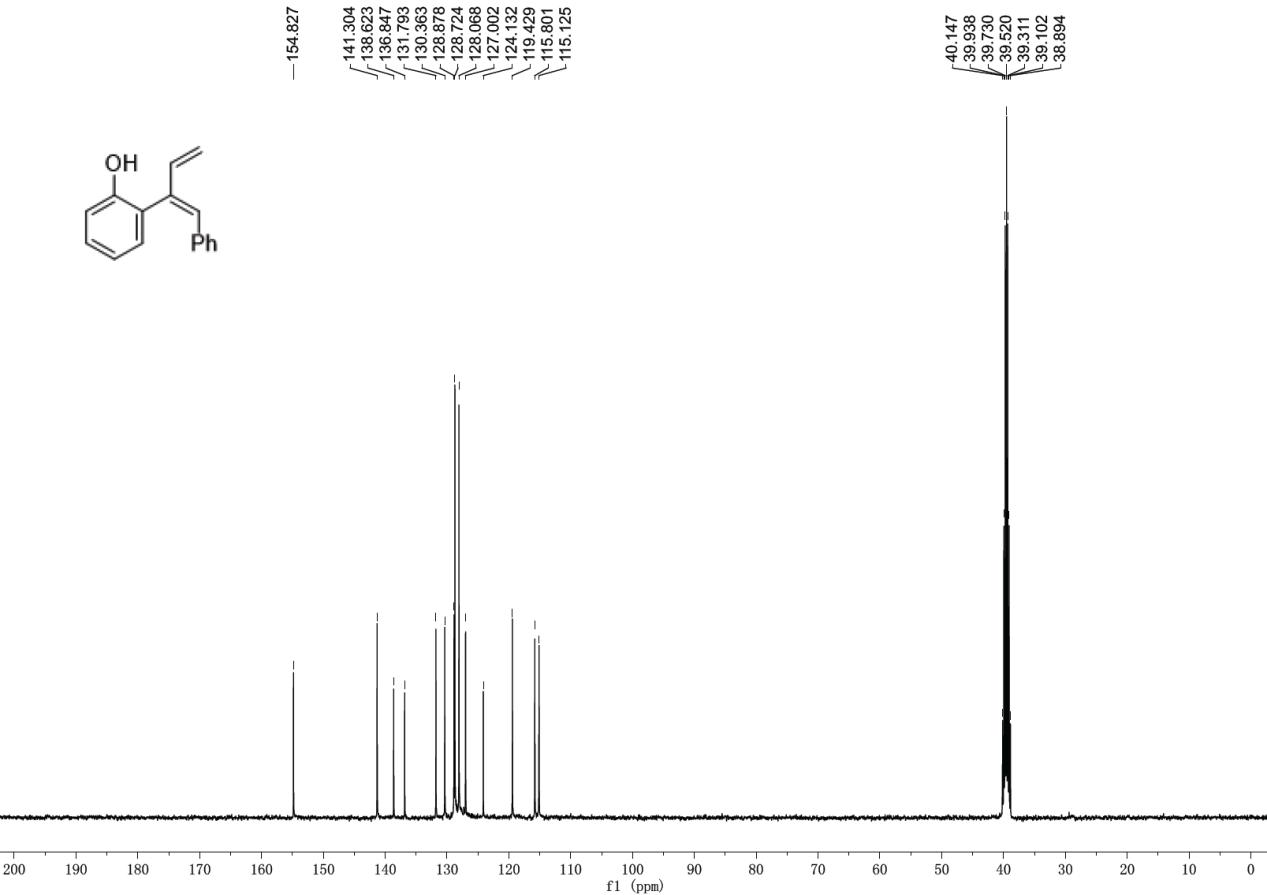


**Supplementary Figure 11.** ^13^C-NMR spectrum of **3a**

**3b**


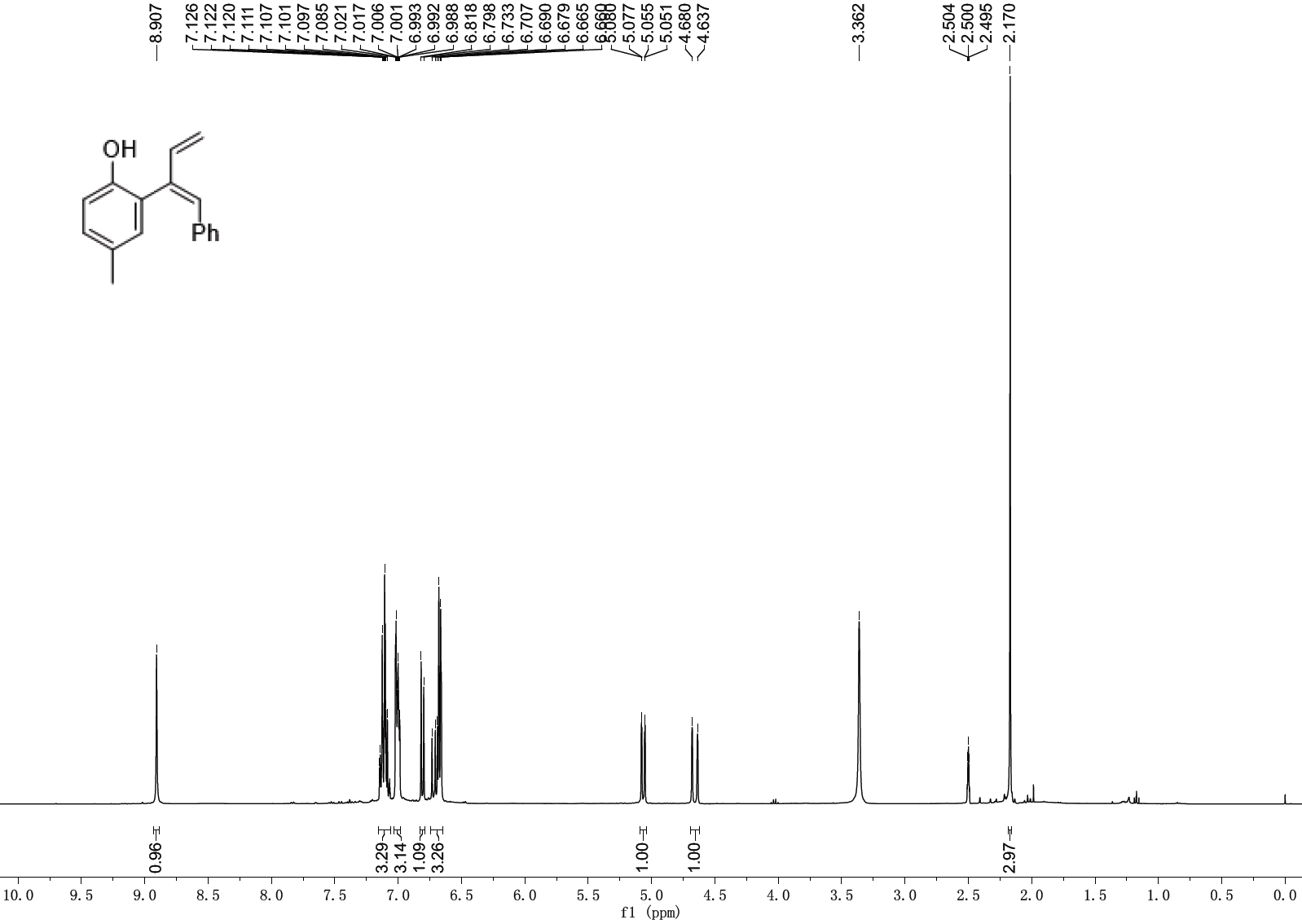


**Supplementary Figure 12.** ^1^H-NMR spectrum of **3b**


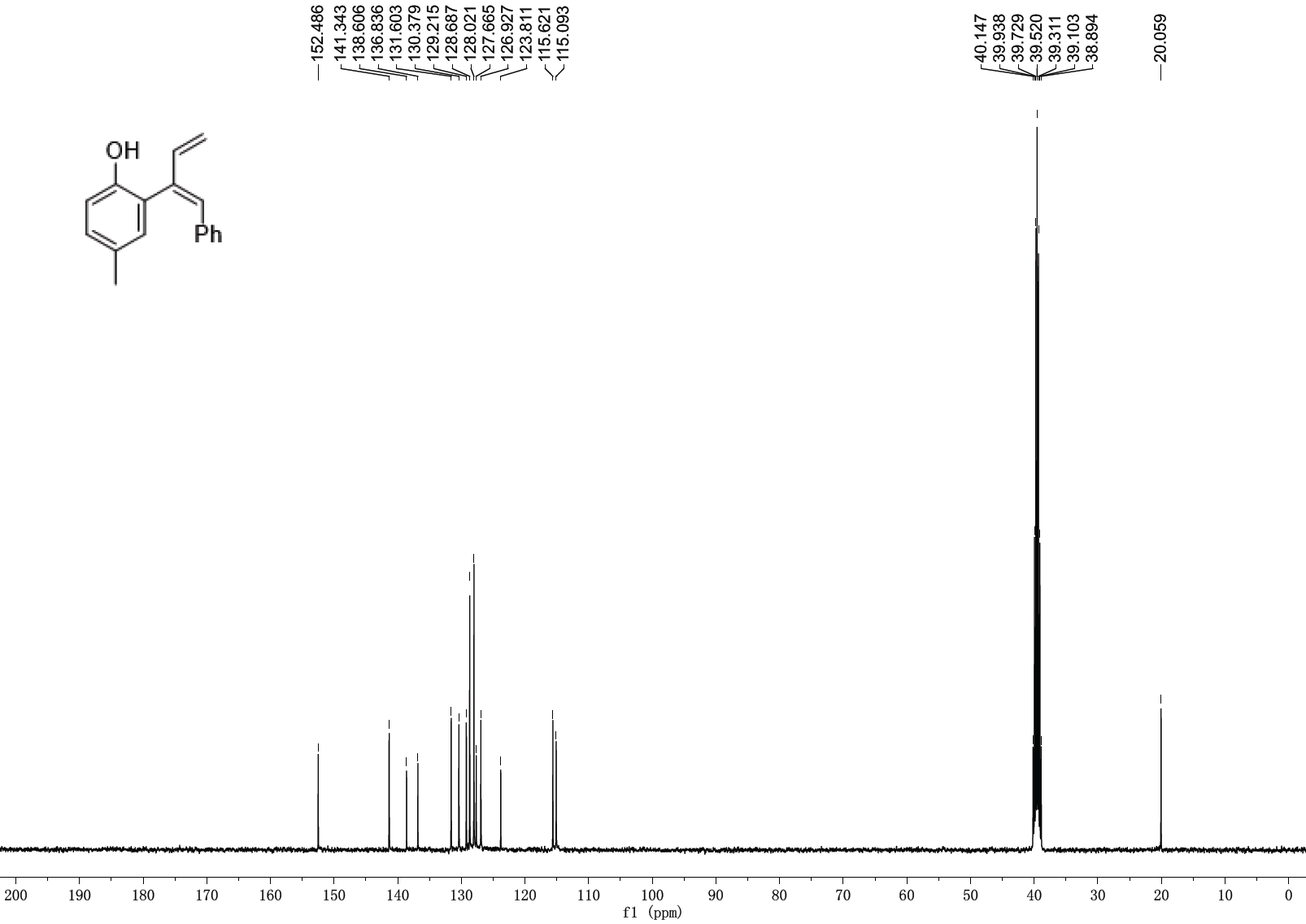


**Supplementary Figure 13.** ^13^C-NMR spectrum of **3b**

**3c**


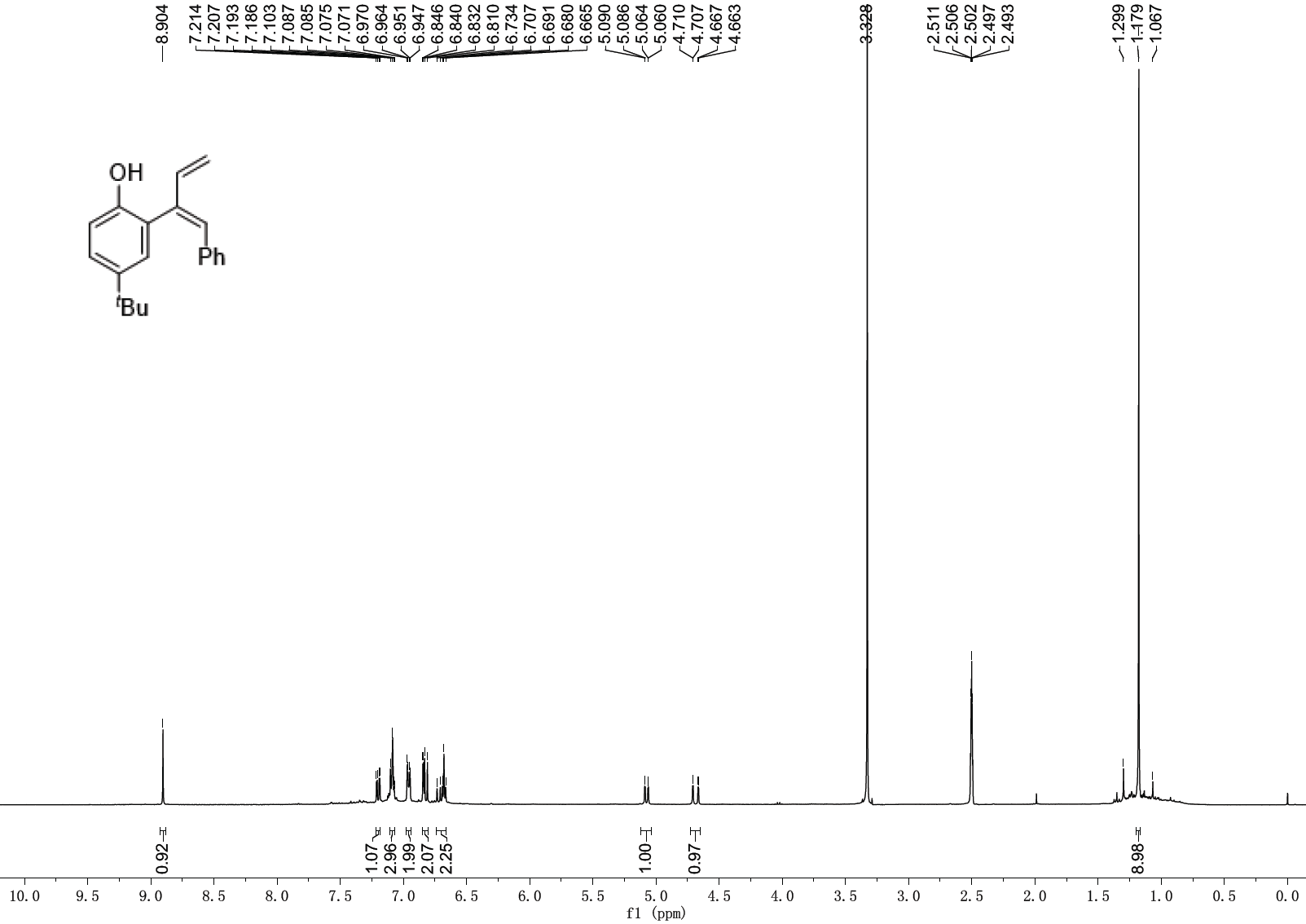


**Supplementary Figure 14.** ^1^H-NMR spectrum of **3c**


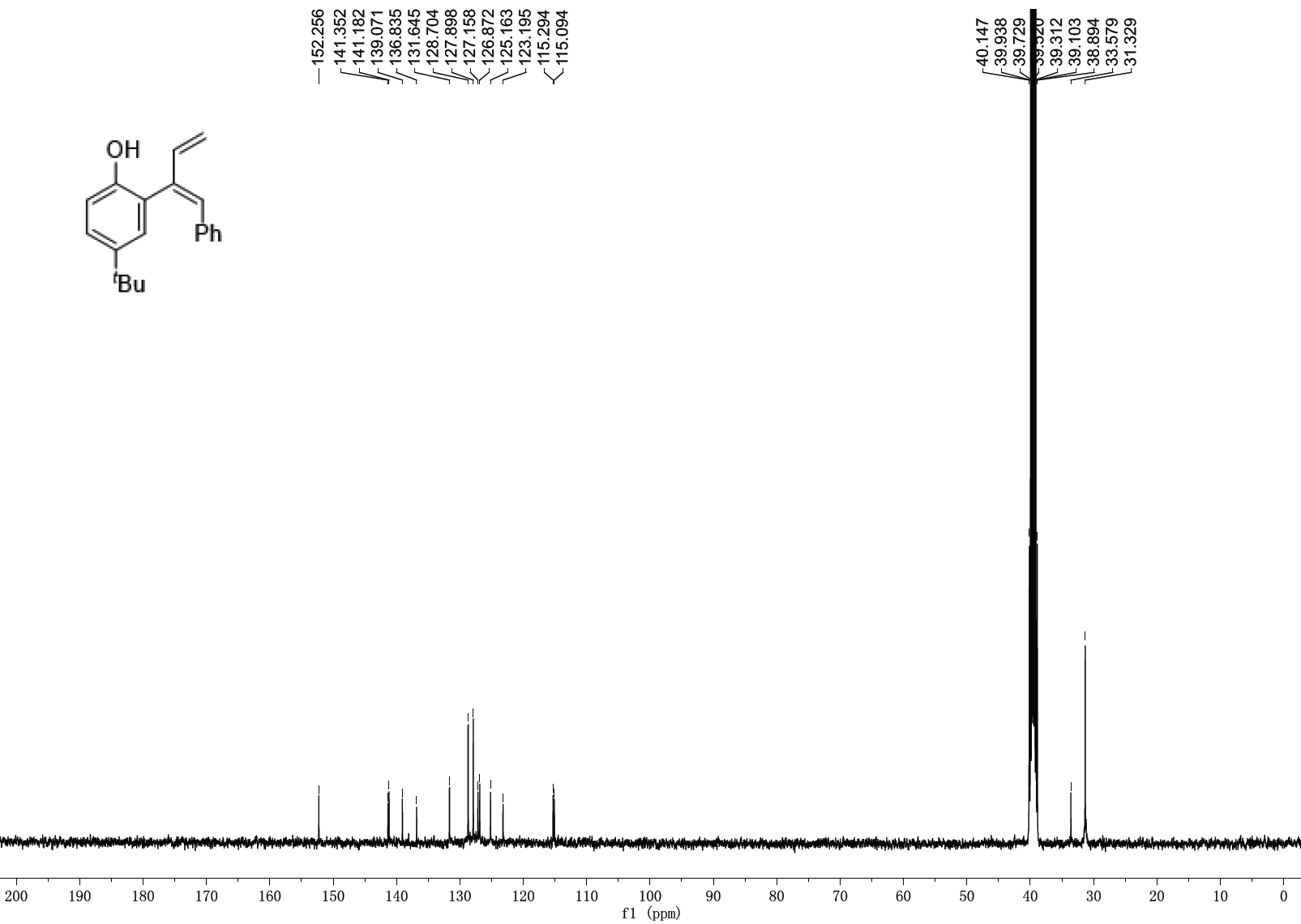


**Supplementary Figure 15.** ^13^C-NMR spectrum of **3c**

**3d**

**^
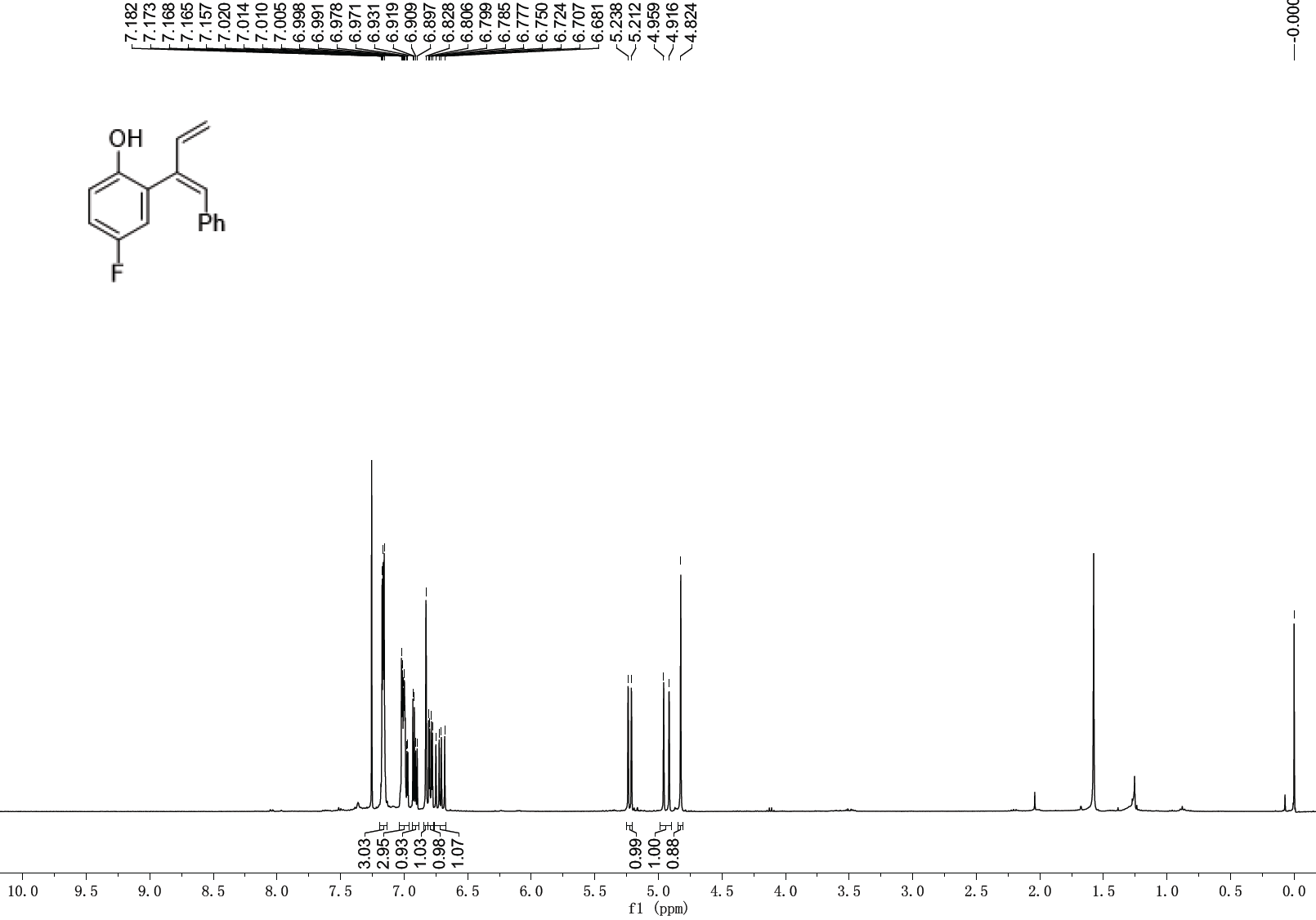
^**

**Supplementary Figure 16.** ^1^H-NMR spectrum of **3d**

**^
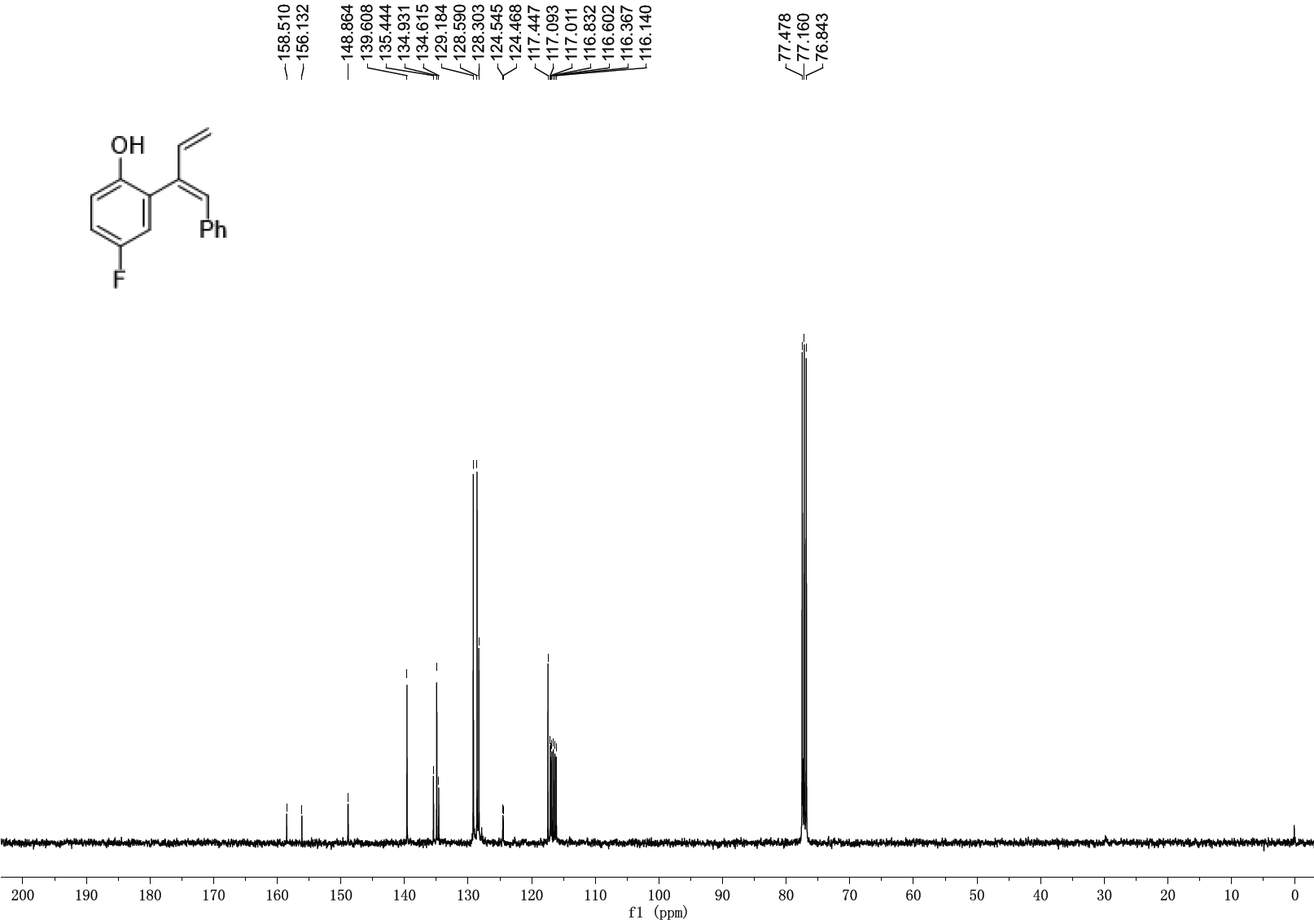
^**

**Supplementary Figure 17.** ^13^C-NMR spectrum of **3d**


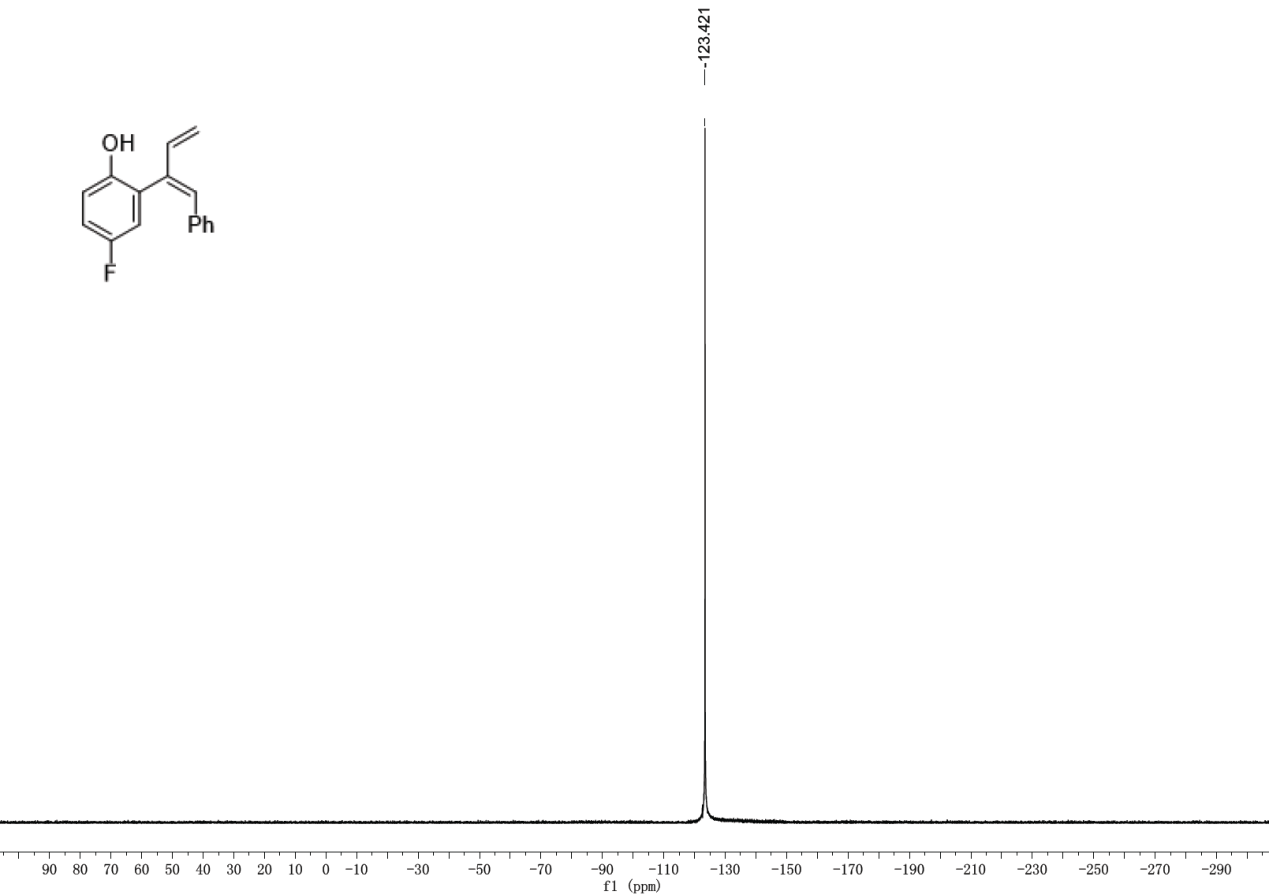


**Supplementary Figure 18.** ^19^F-NMR spectrum of **3d**

**3e**


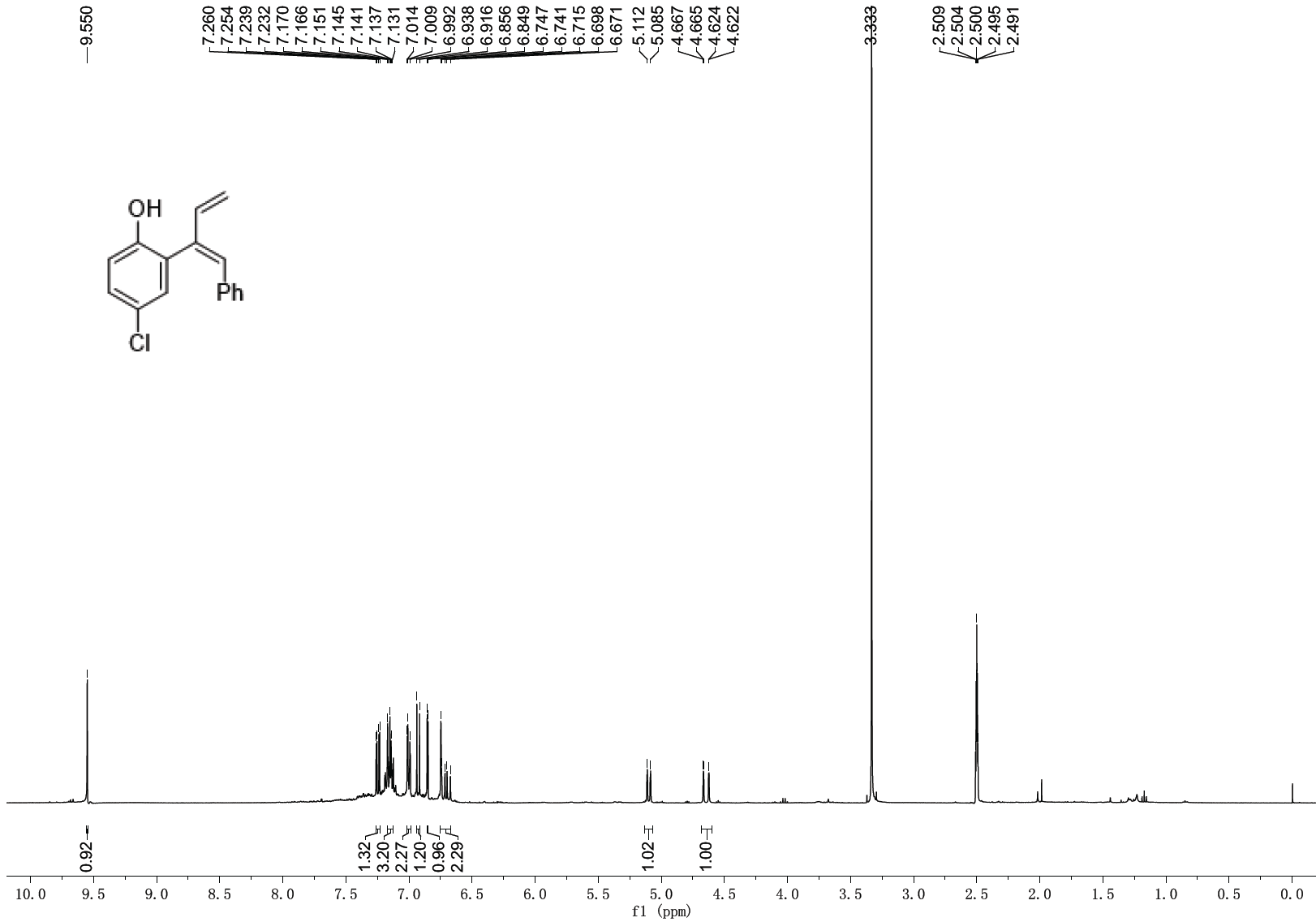


**Supplementary Figure 19.** ^1^H-NMR spectrum of **3e**


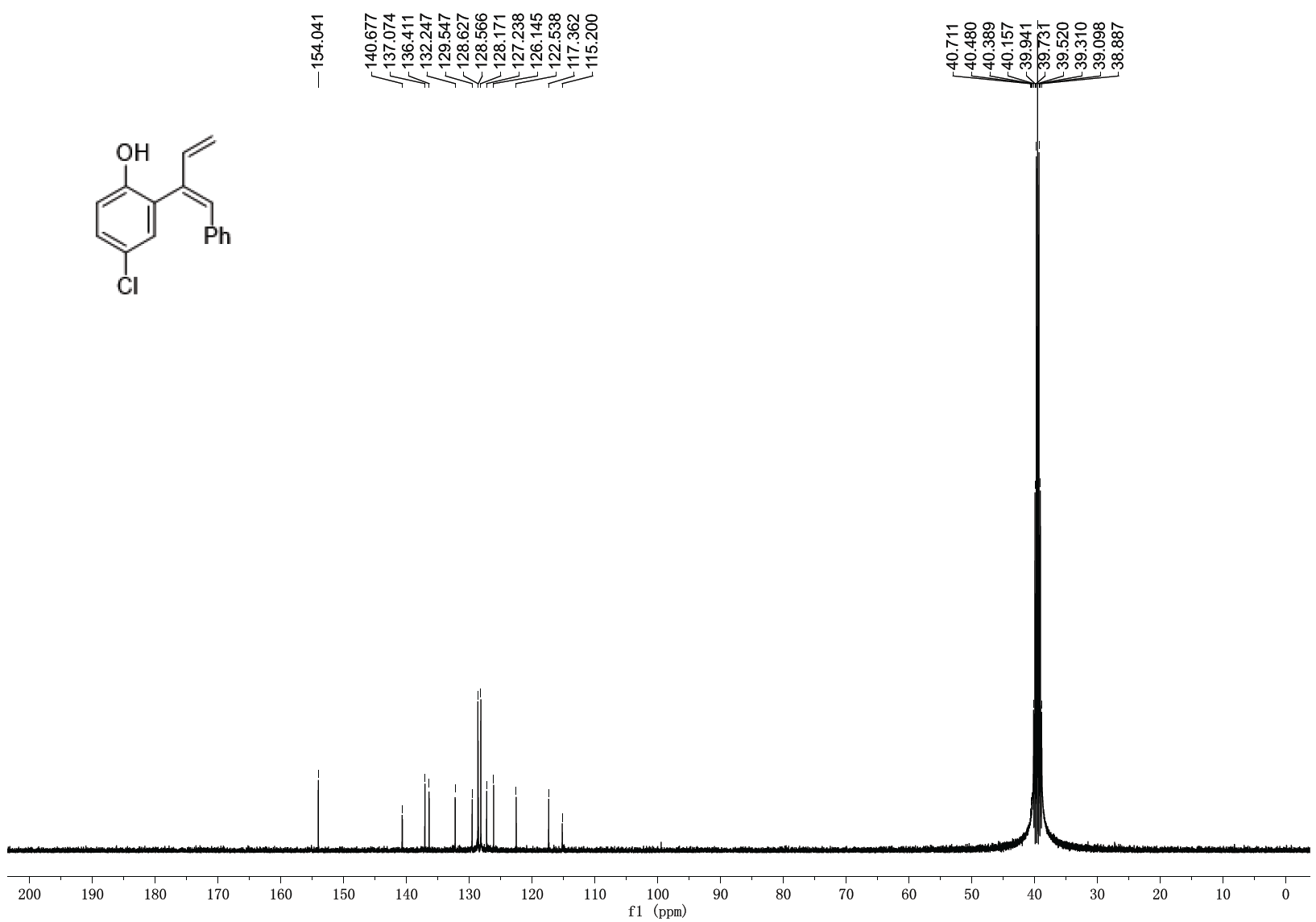


**Supplementary Figure 20.** ^13^C-NMR spectrum of **3e**

**3f**


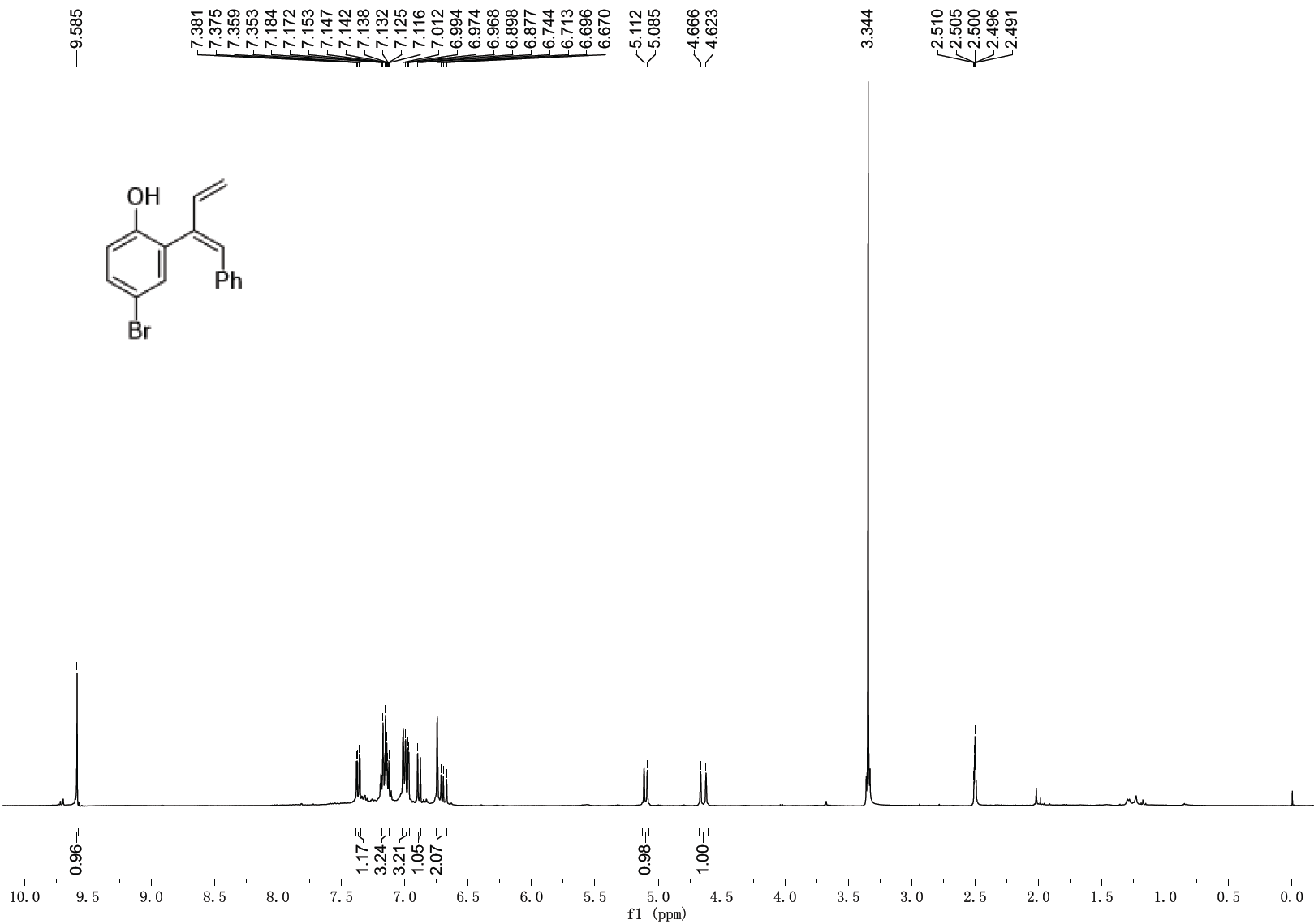


**Supplementary Figure 21.** ^1^H-NMR spectrum of **3f**


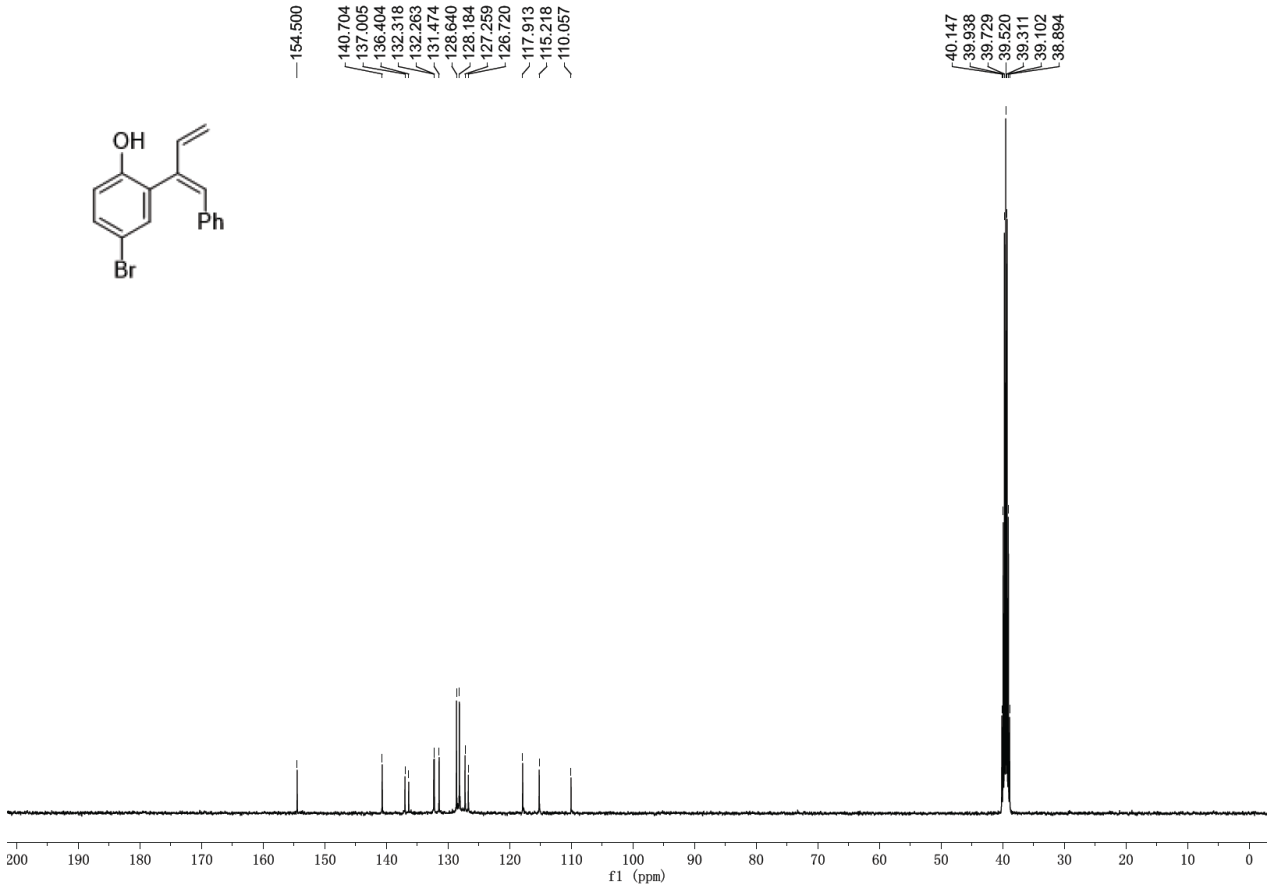


**Supplementary Figure 22.** ^13^C-NMR spectrum of **3f**

**3g**

**
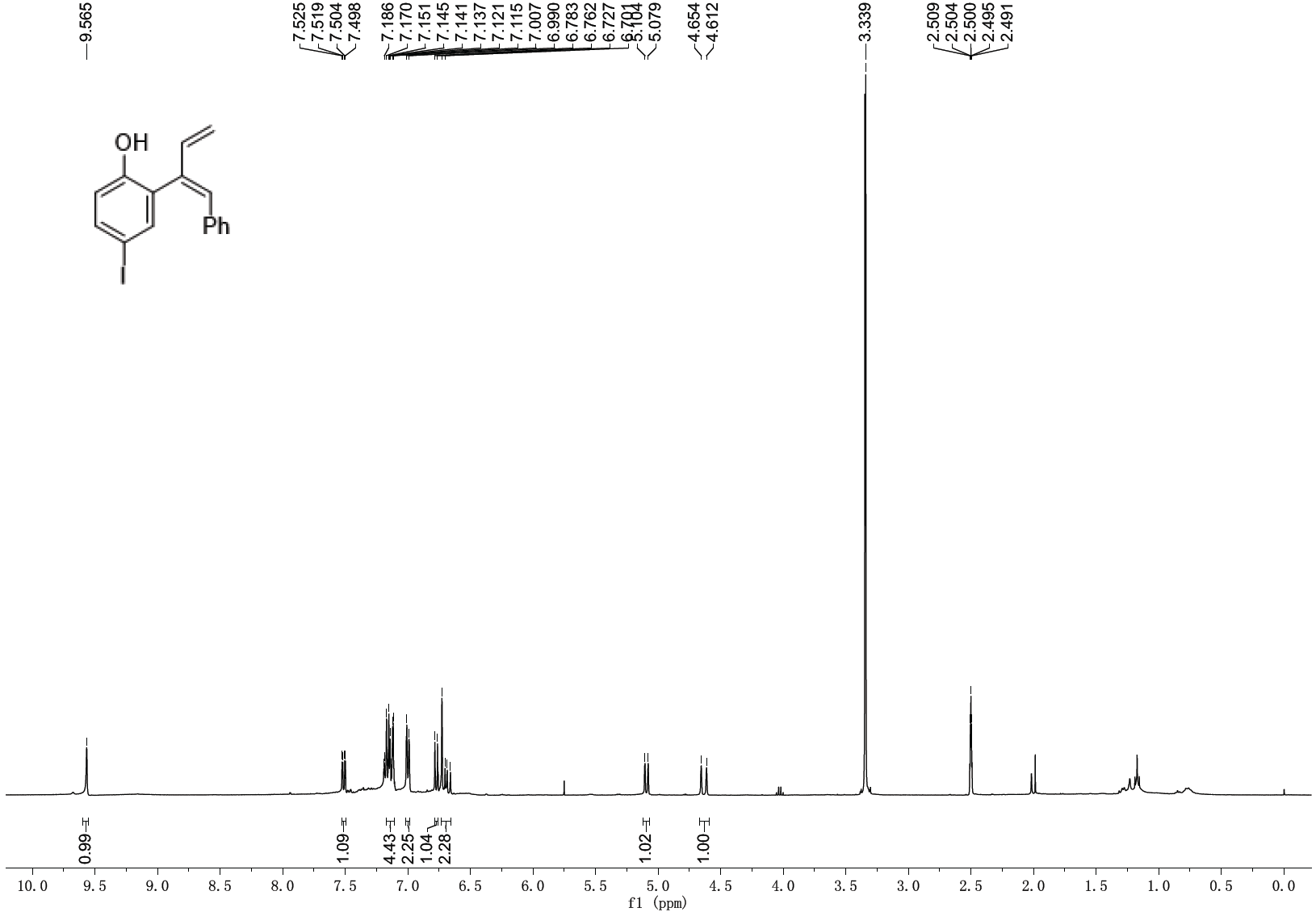
**

**Supplementary Figure 23.** ^1^H-NMR spectrum of **3g**

**
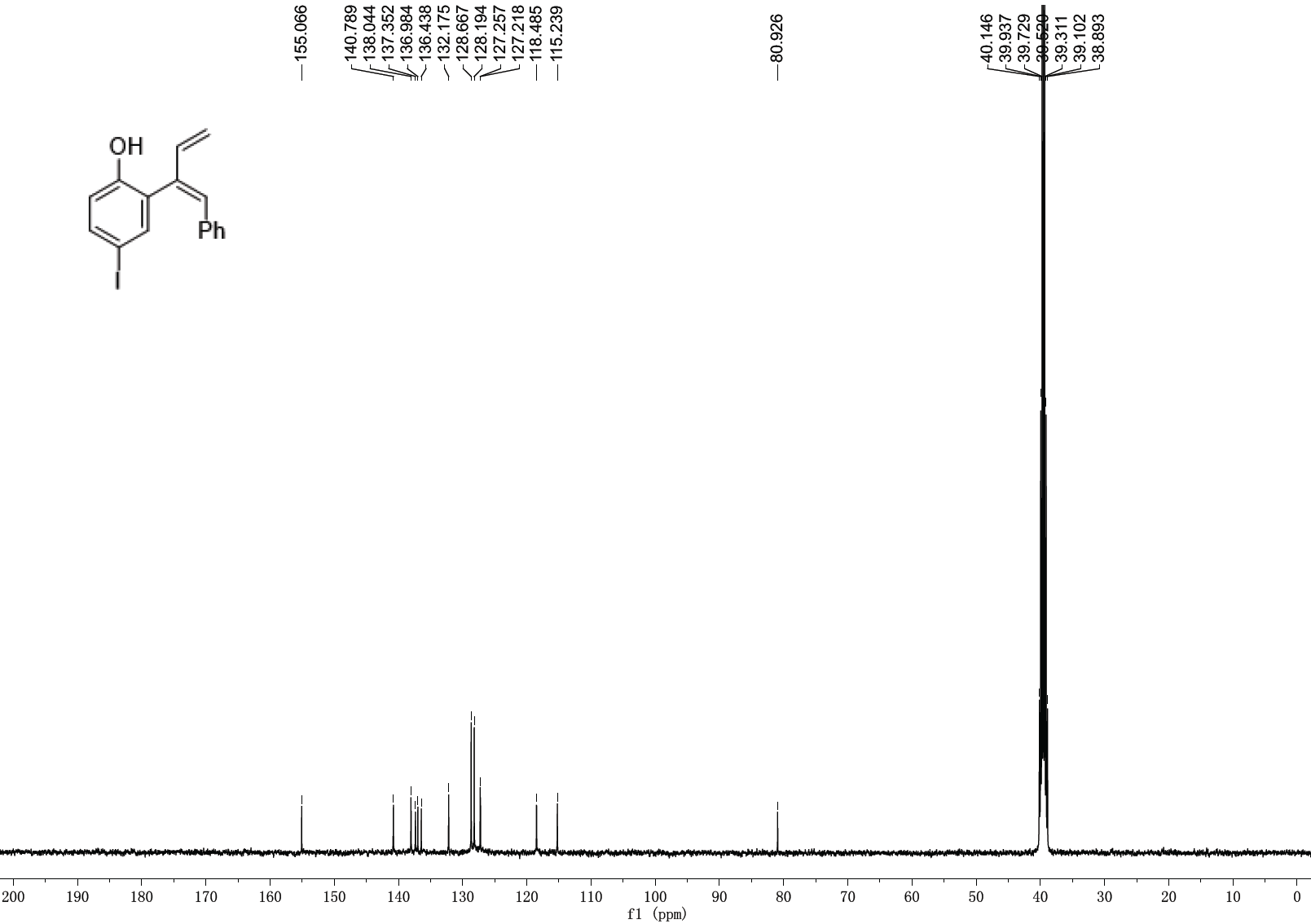
**

**Supplementary Figure 24.** ^13^C-NMR spectrum of **3g**

**3h**

**
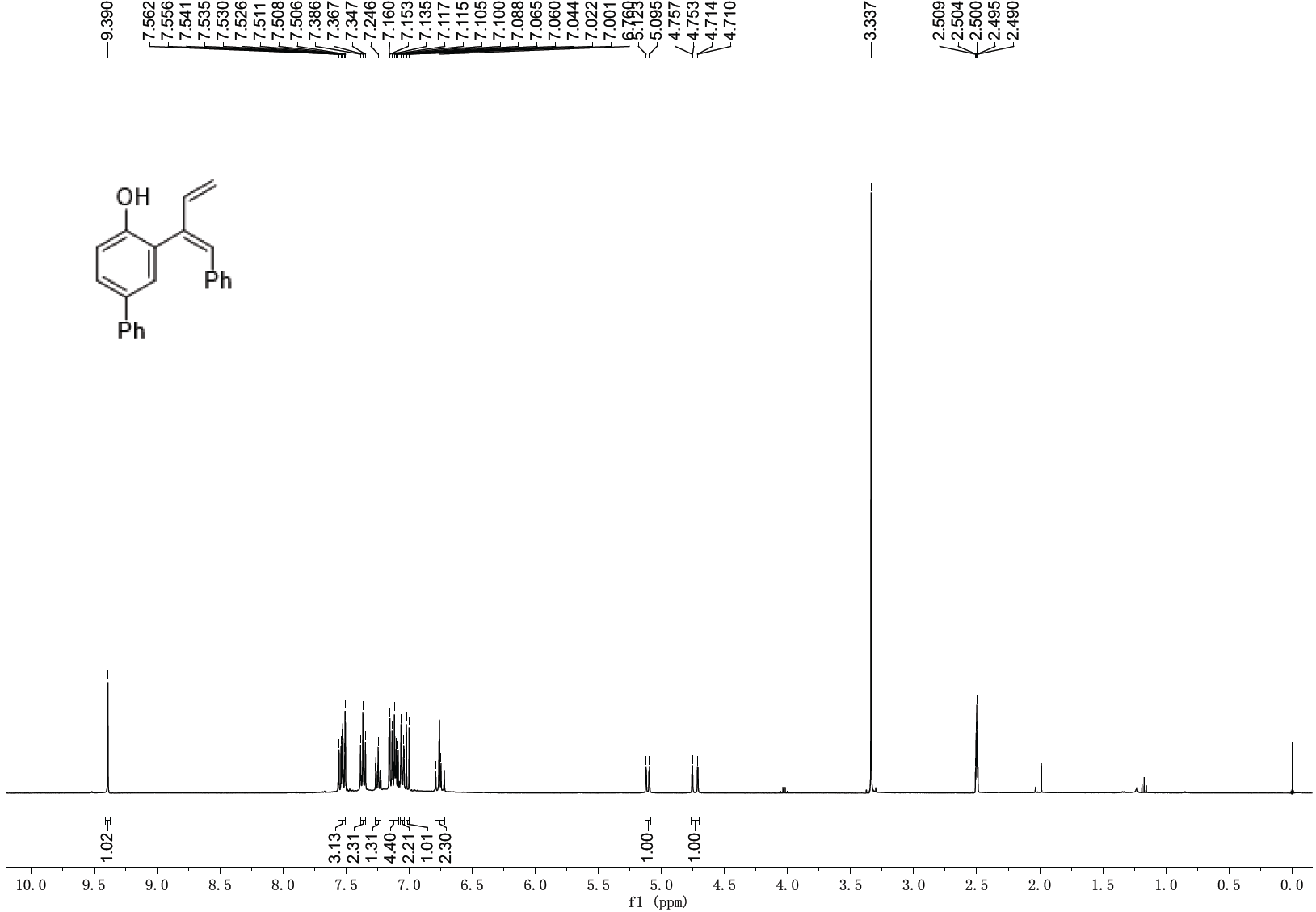
**

**Supplementary Figure 25.** ^1^H-NMR spectrum of **3h**


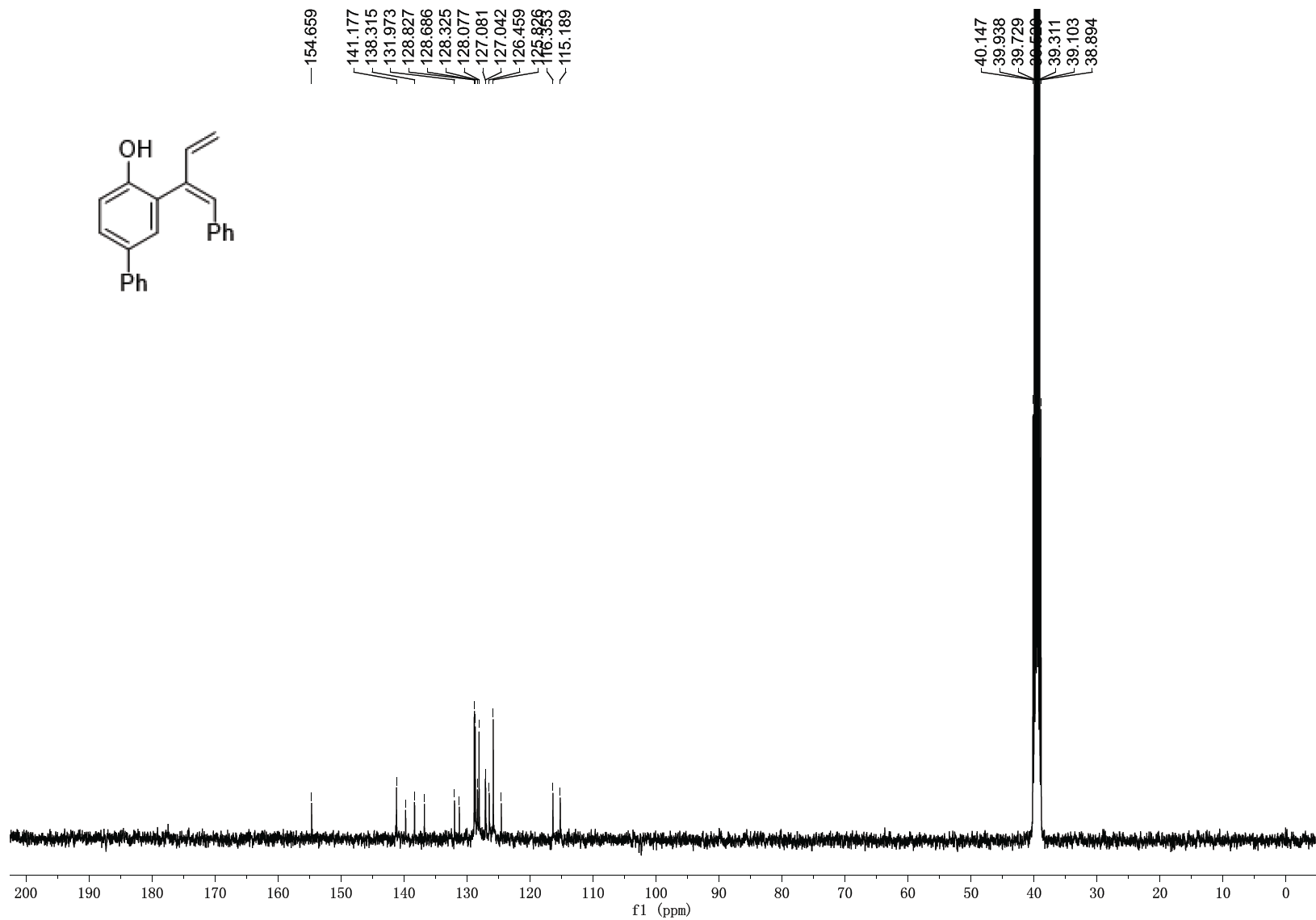


**Supplementary Figure 26.** ^13^C-NMR spectrum of **3h**

**3i**

**
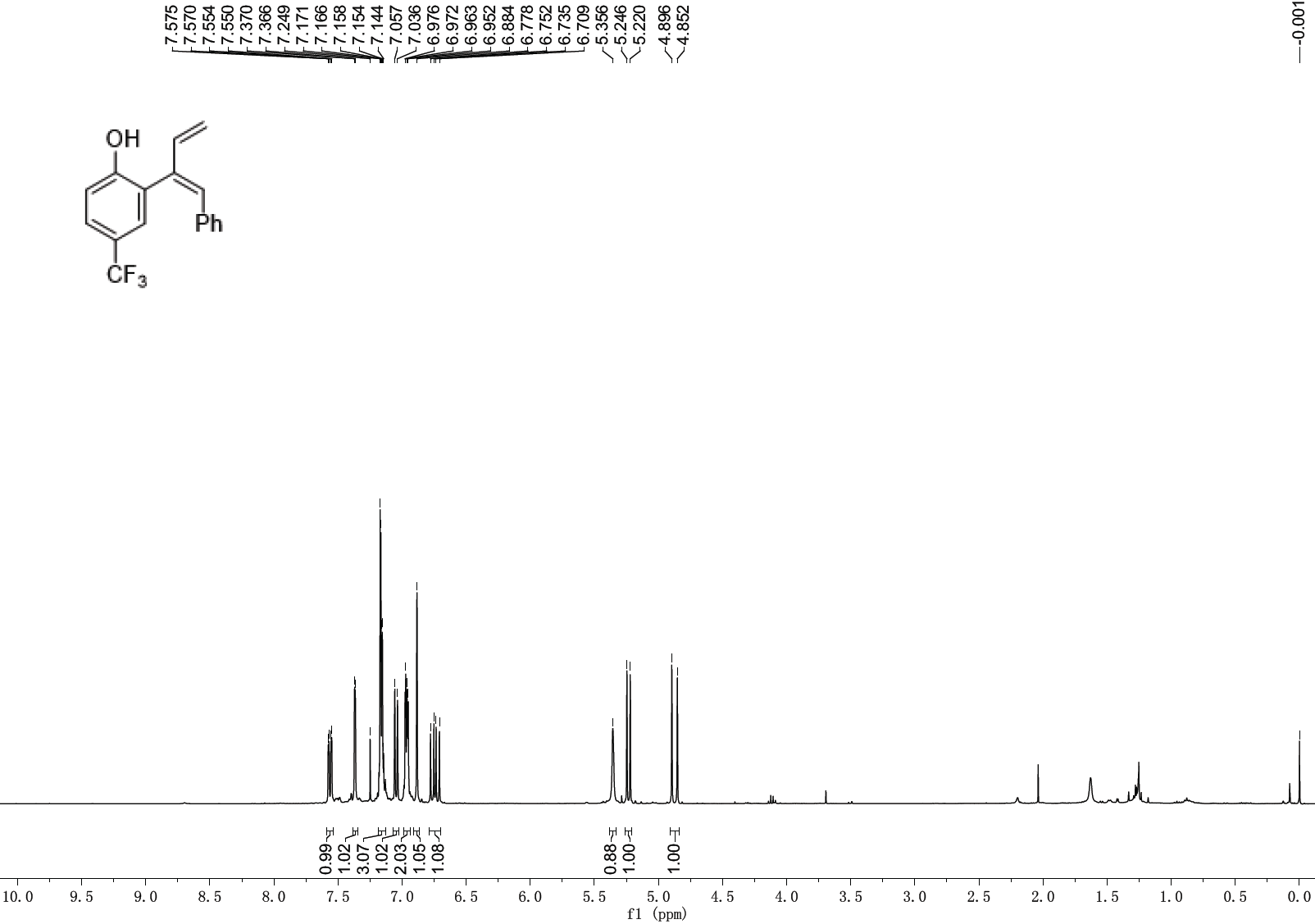
**

**Supplementary Figure 27.** ^1^H-NMR spectrum of **3i**

**
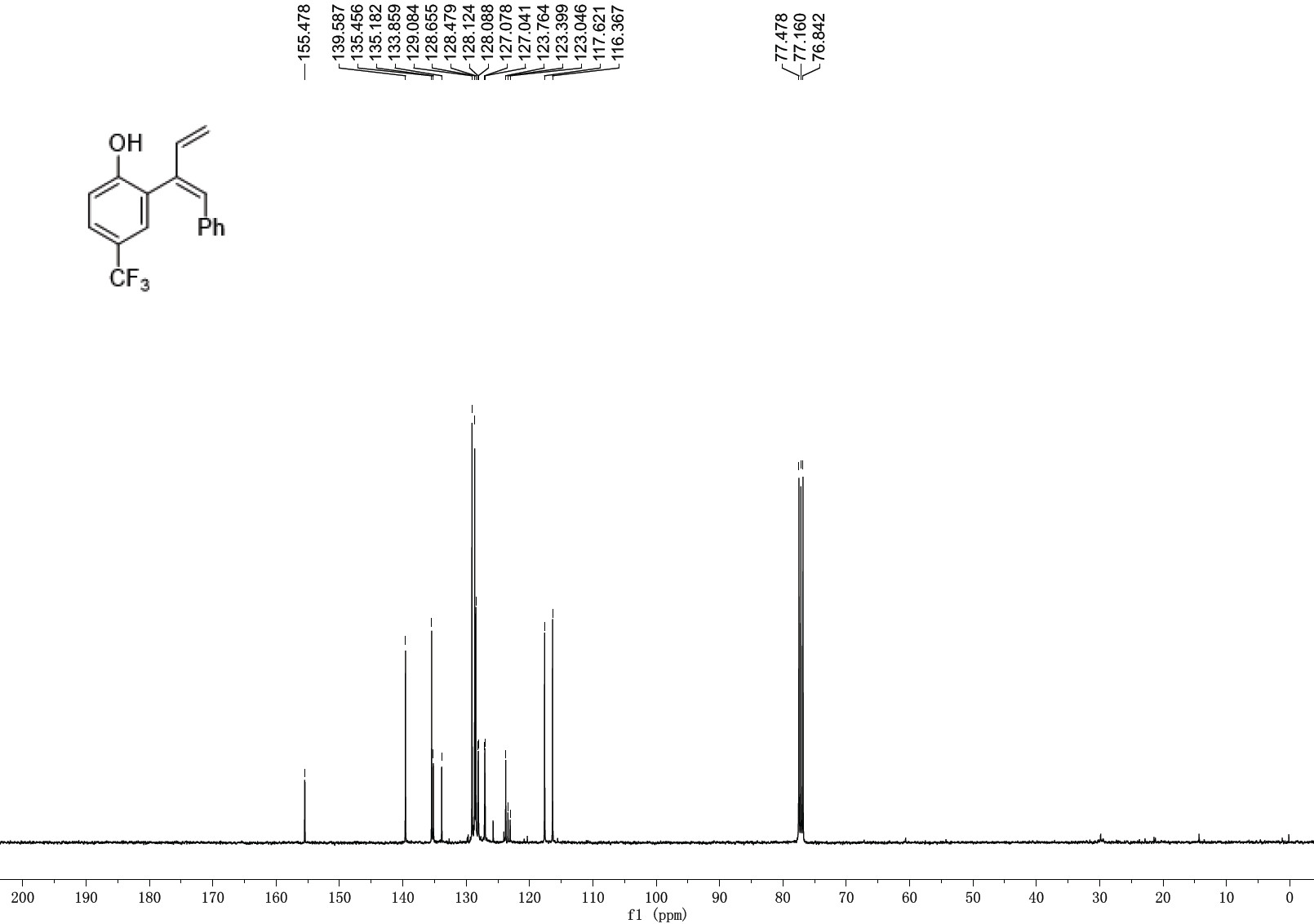
**

**Supplementary Figure 28.** ^13^C-NMR spectrum of **3i**

**
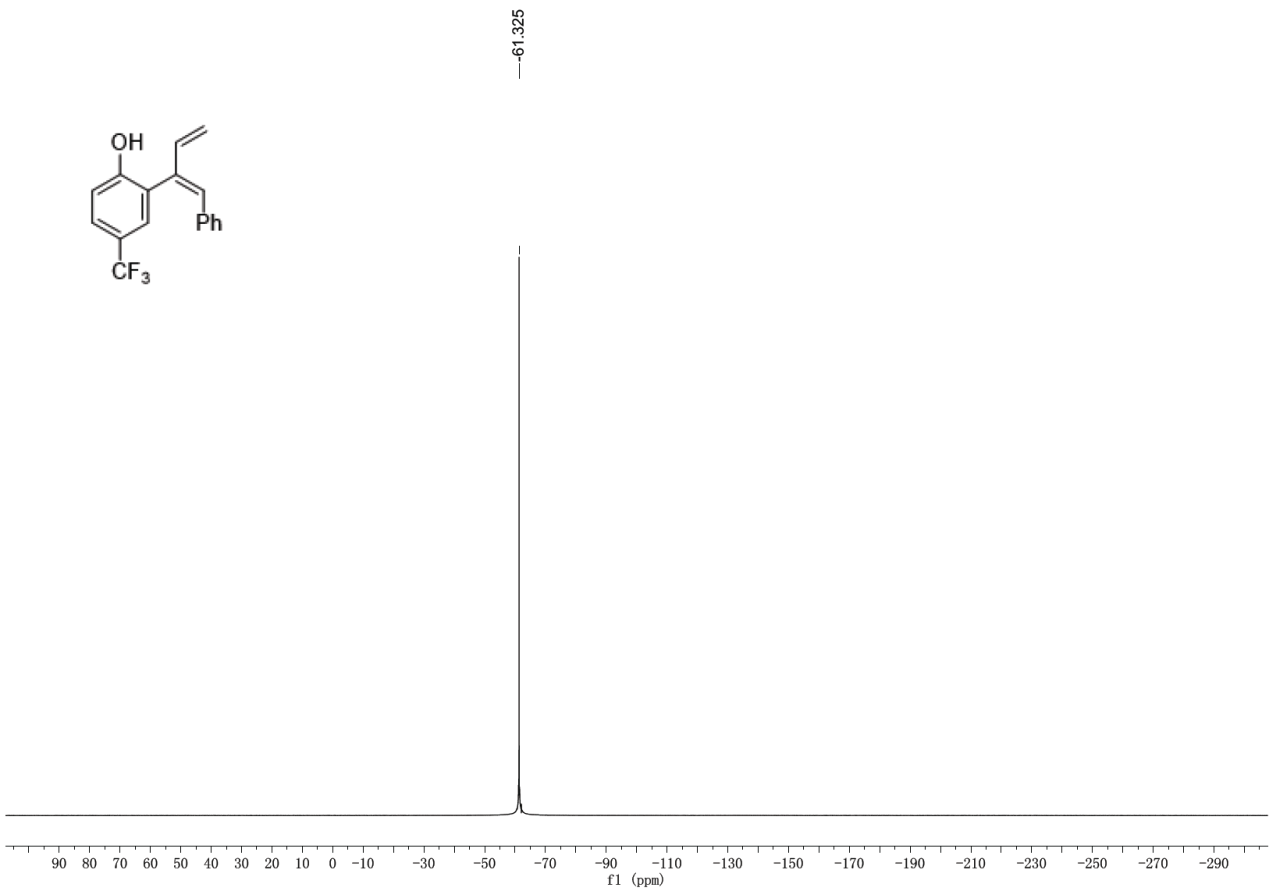
**

**Supplementary Figure 29.** ^19^F-NMR spectrum of **3i**

**3j**

**
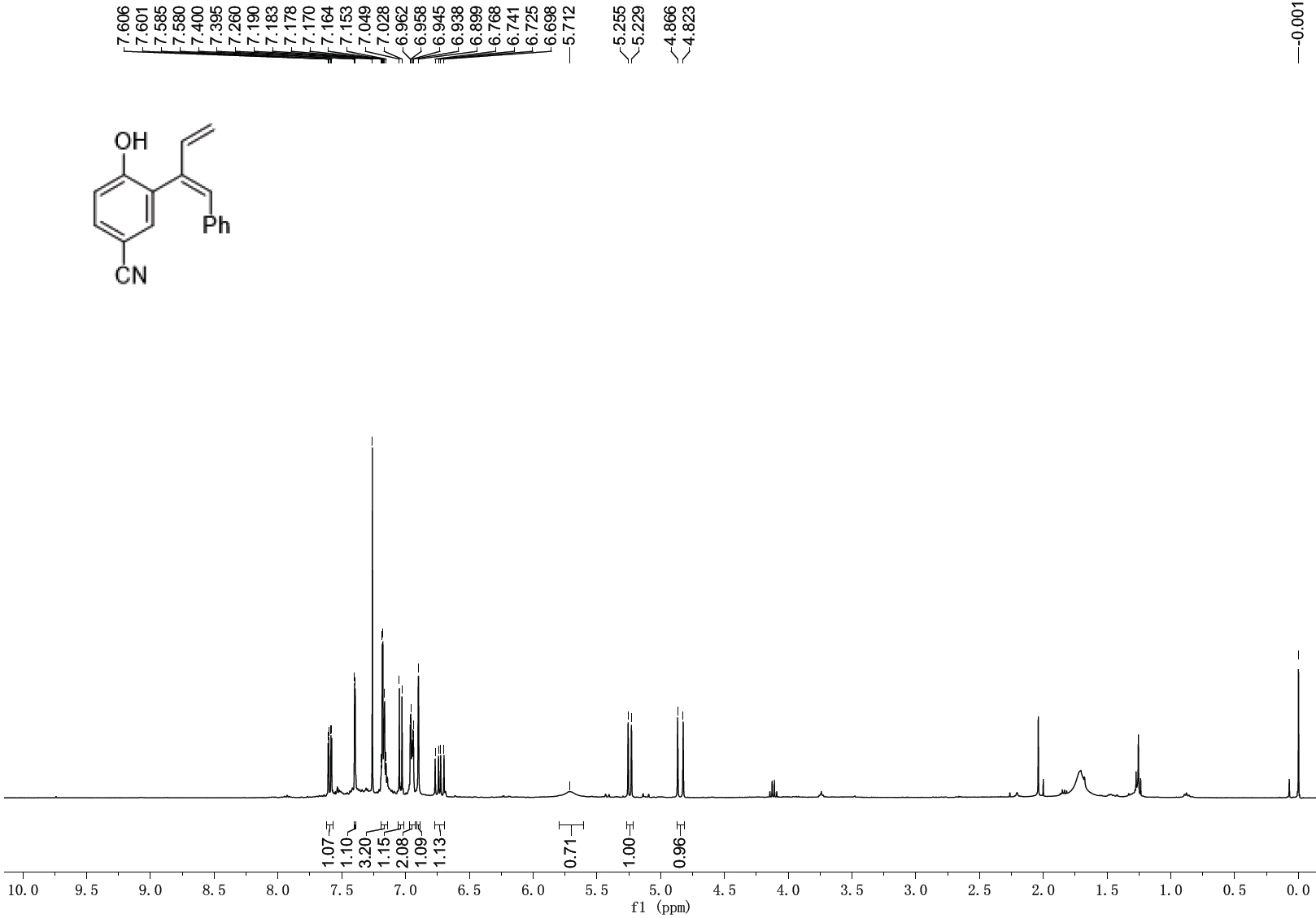
**

**Supplementary Figure 30.** ^1^H-NMR spectrum of **3j**

**
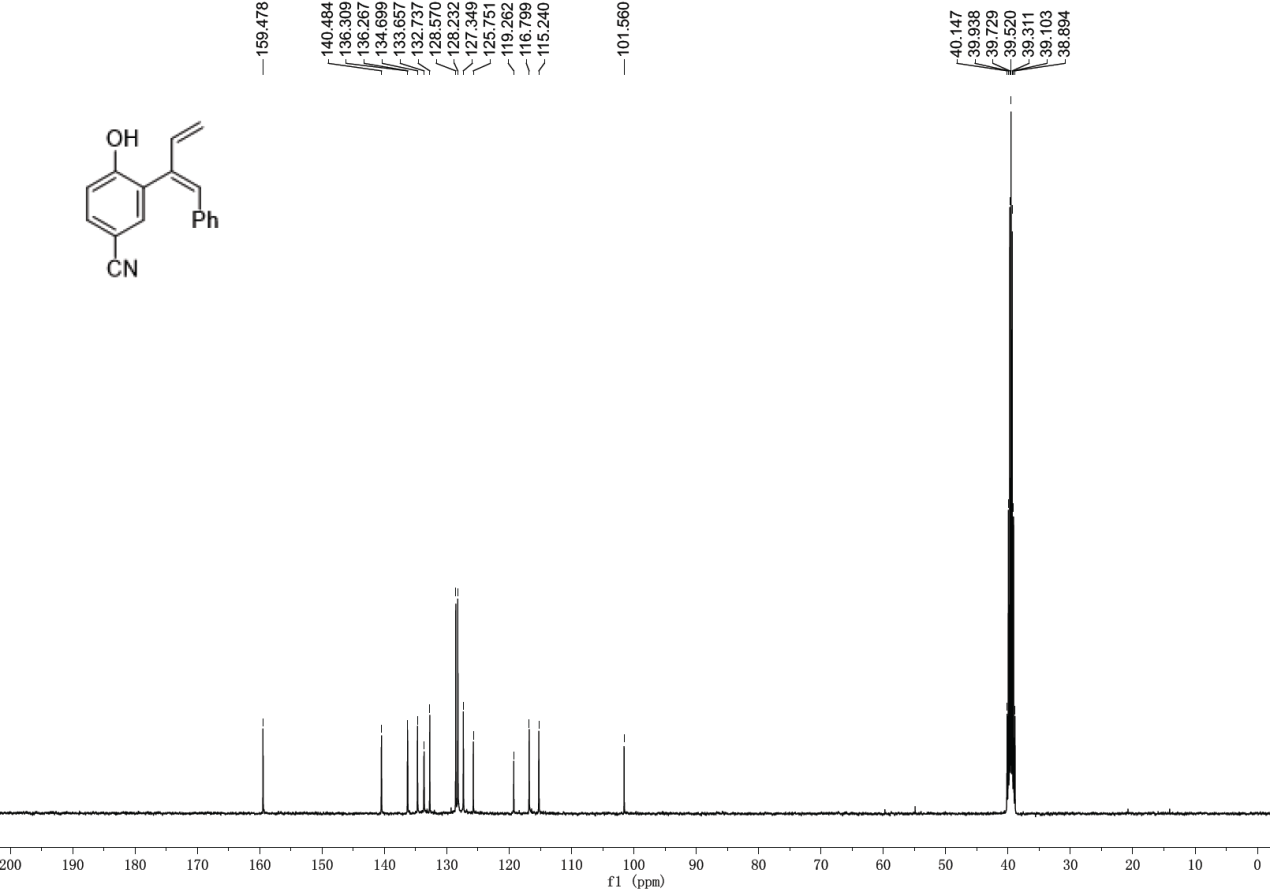
**

**Supplementary Figure 31.** ^13^C-NMR spectrum of **3j**

**3k**

**
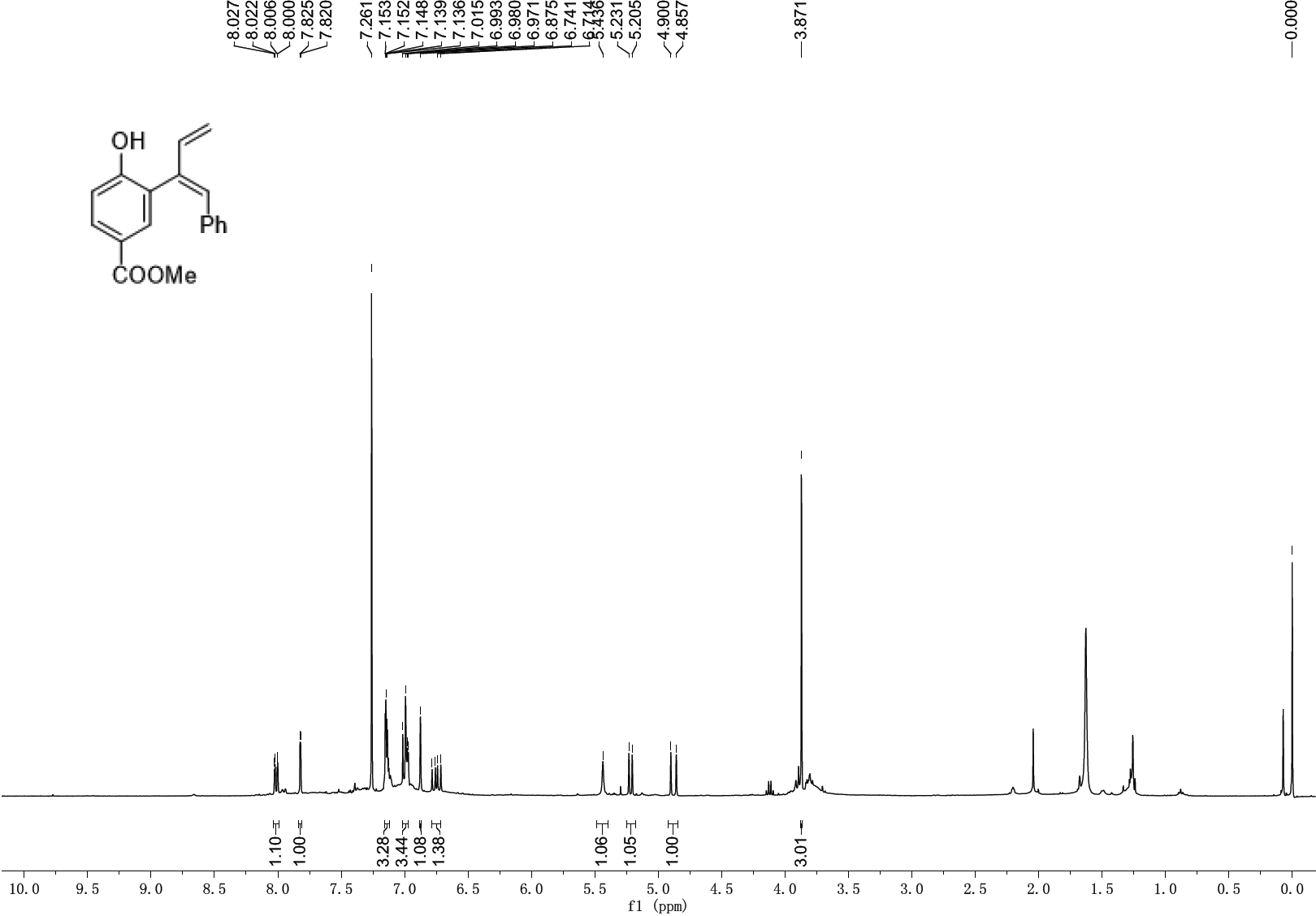
**

**Supplementary Figure 32.** ^1^H-NMR spectrum of **3k**

**
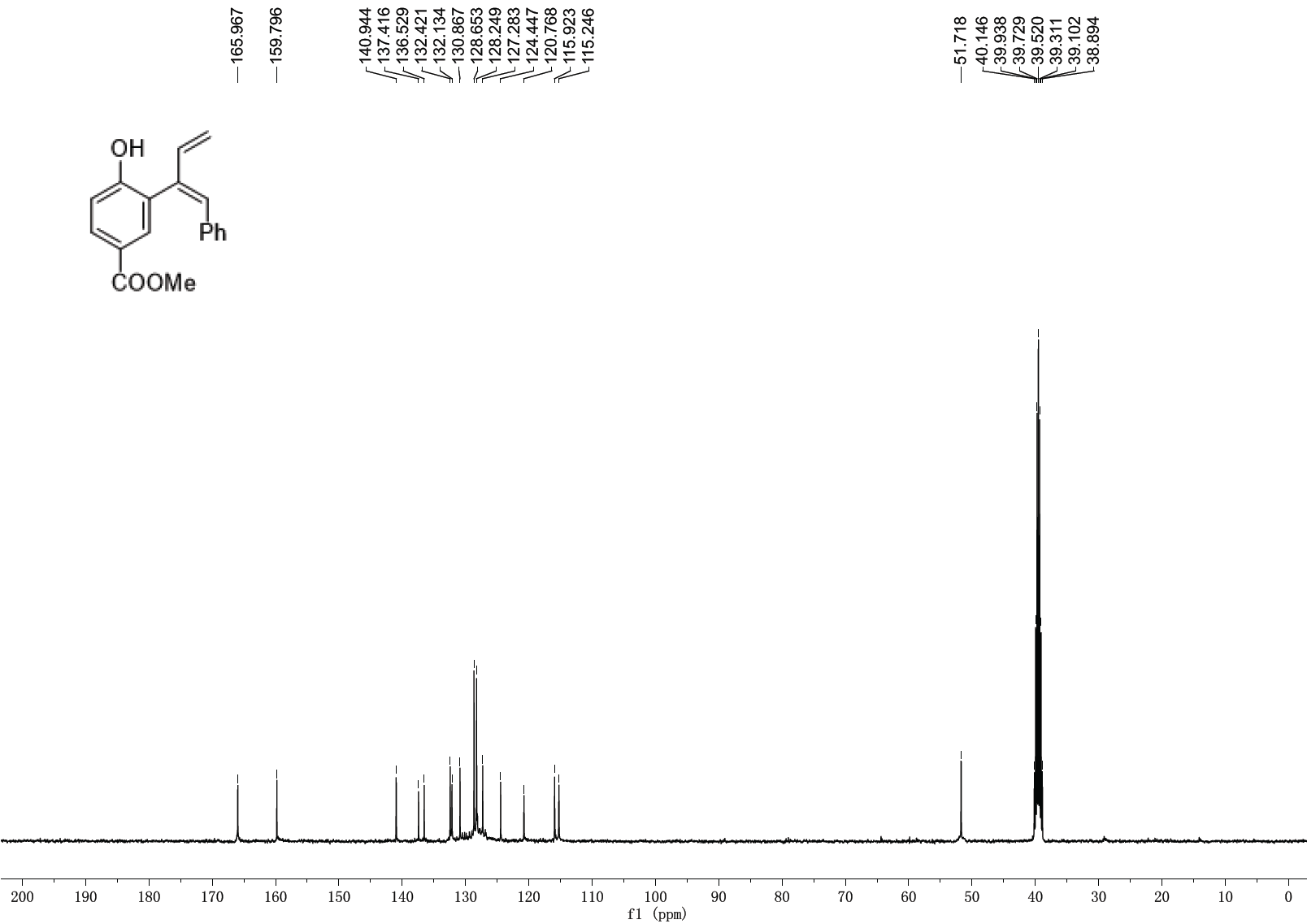
**

**Supplementary Figure 33.** ^13^C-NMR spectrum of **3k**

**3l**

**
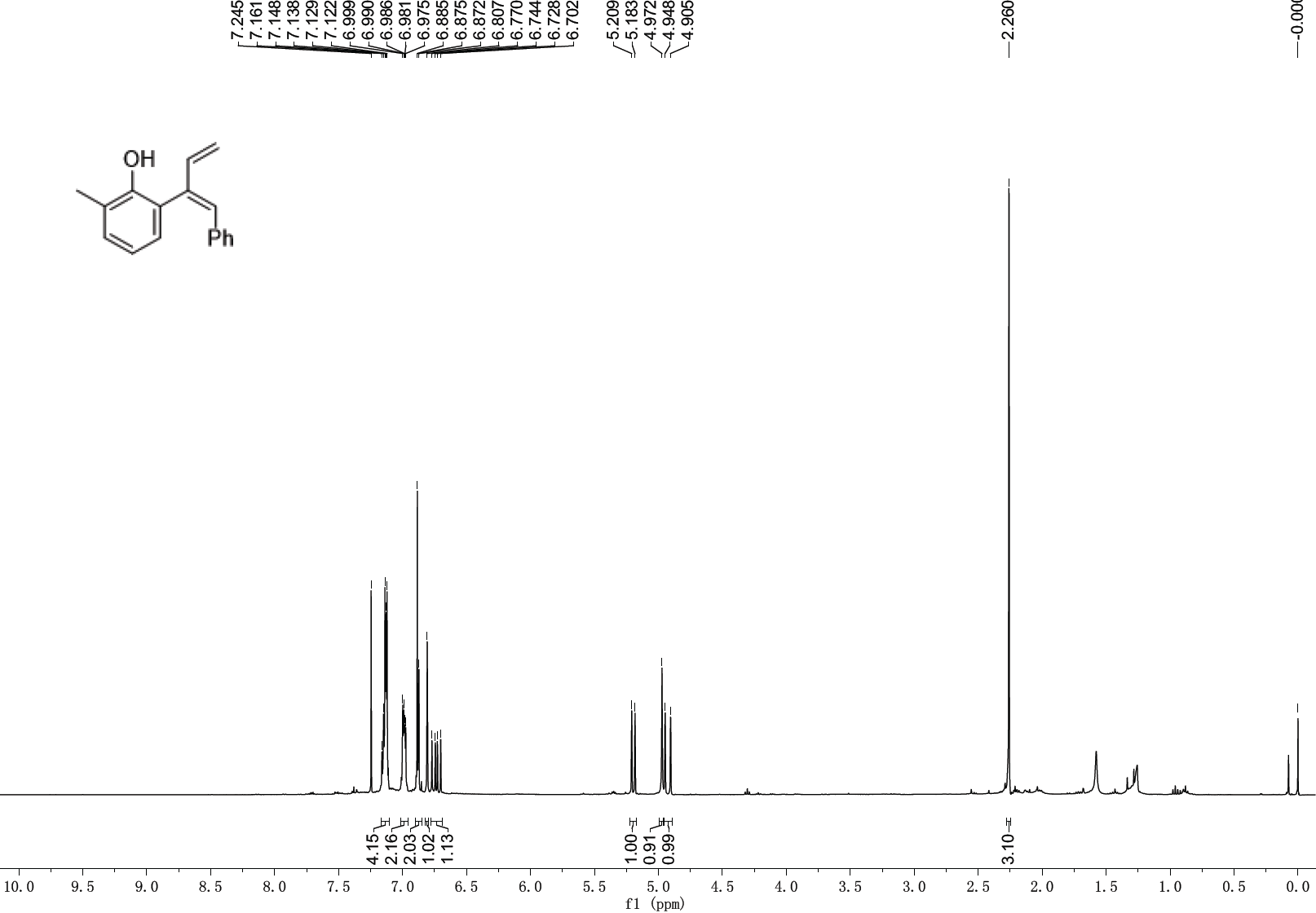
**

**Supplementary Figure 34.** ^1^H-NMR spectrum of **3l**

**
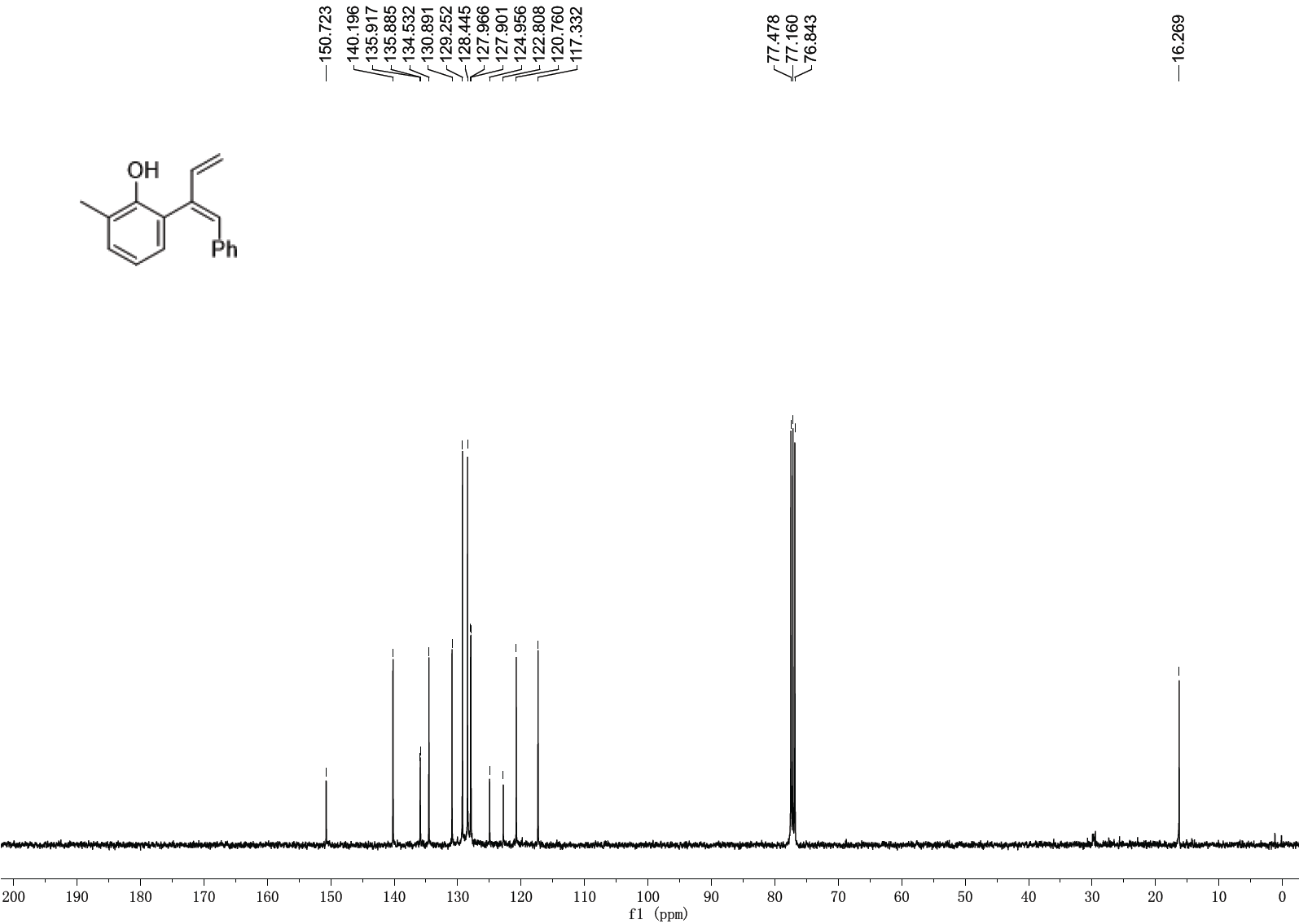
**

**Supplementary Figure 35.** ^13^C-NMR spectrum of **3l**

**3m**

**
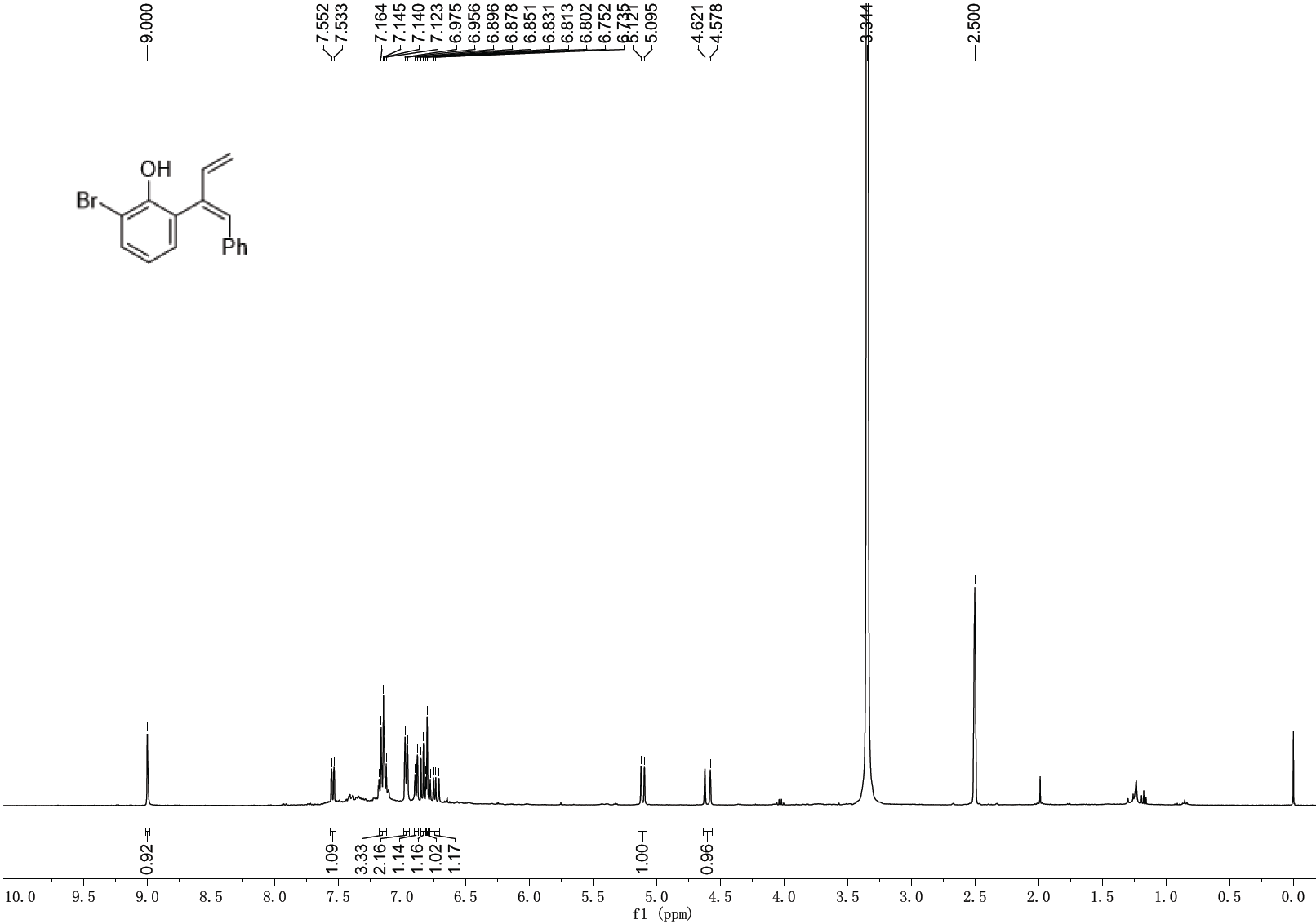
**

**Supplementary Figure 36.** ^1^H-NMR spectrum of **3m**

**
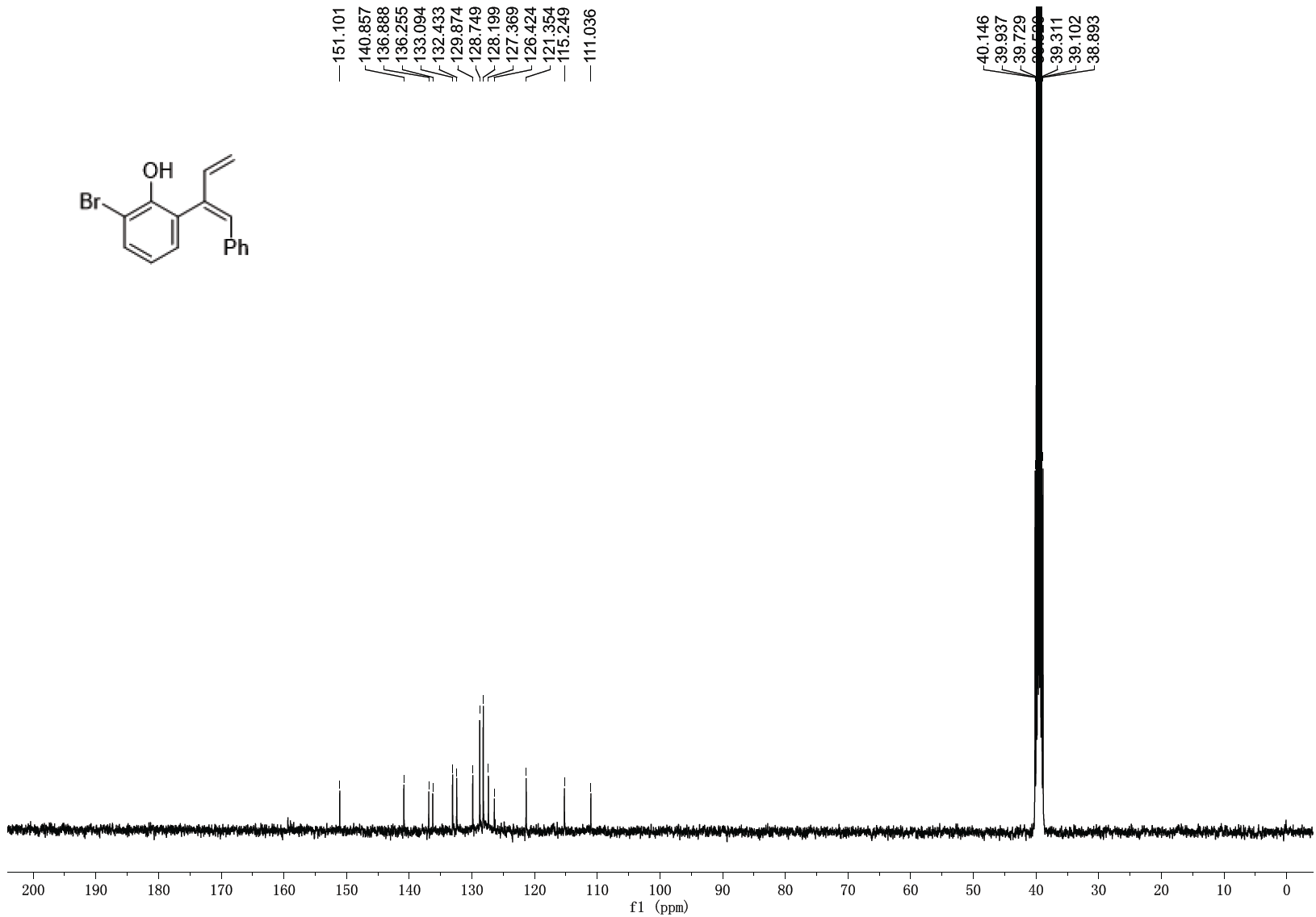
**

**Supplementary Figure 37.** ^13^C-NMR spectrum of **3m**

**3n**

**
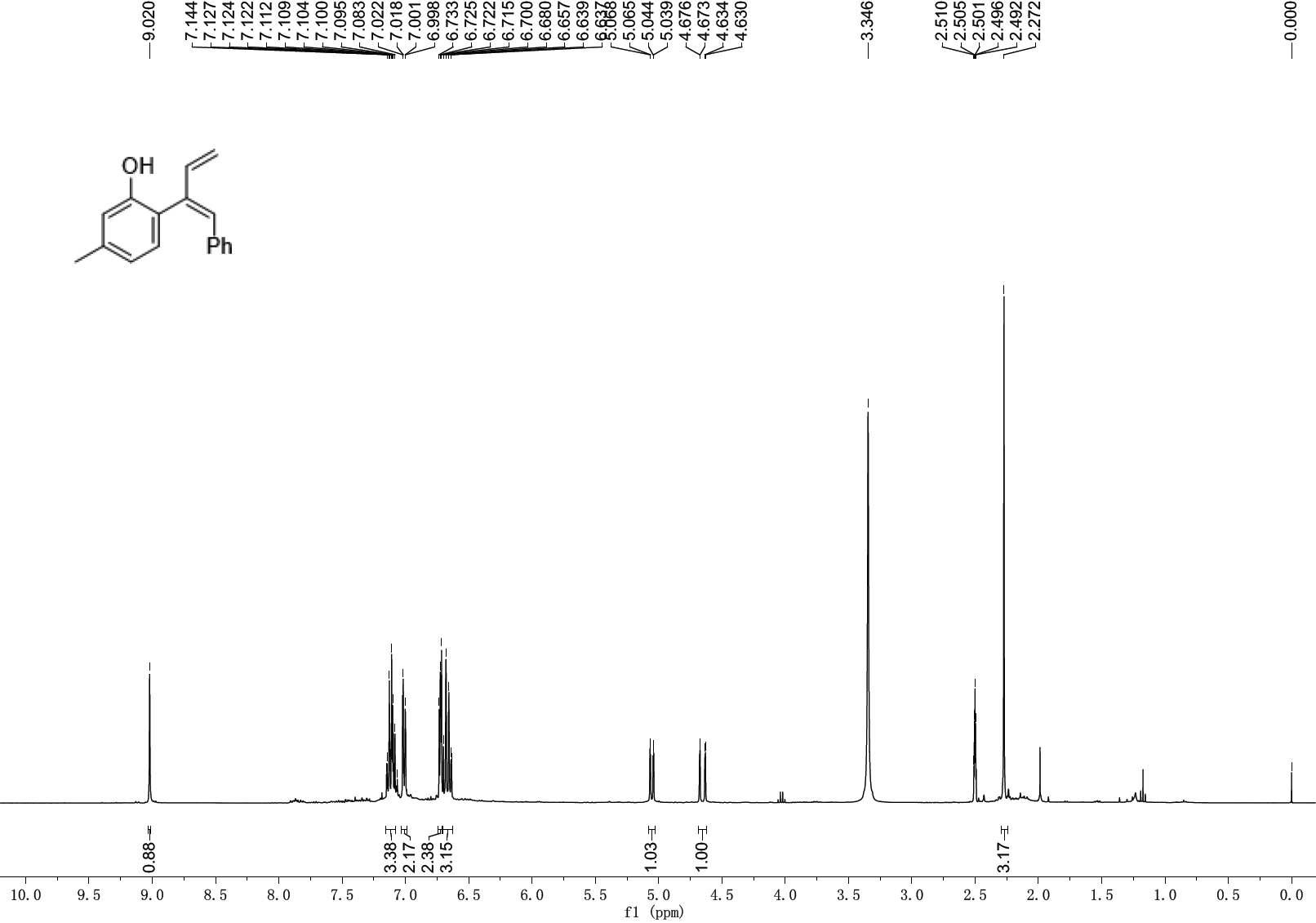
**

**Supplementary Figure 38.** ^1^H-NMR spectrum of **3n**

**
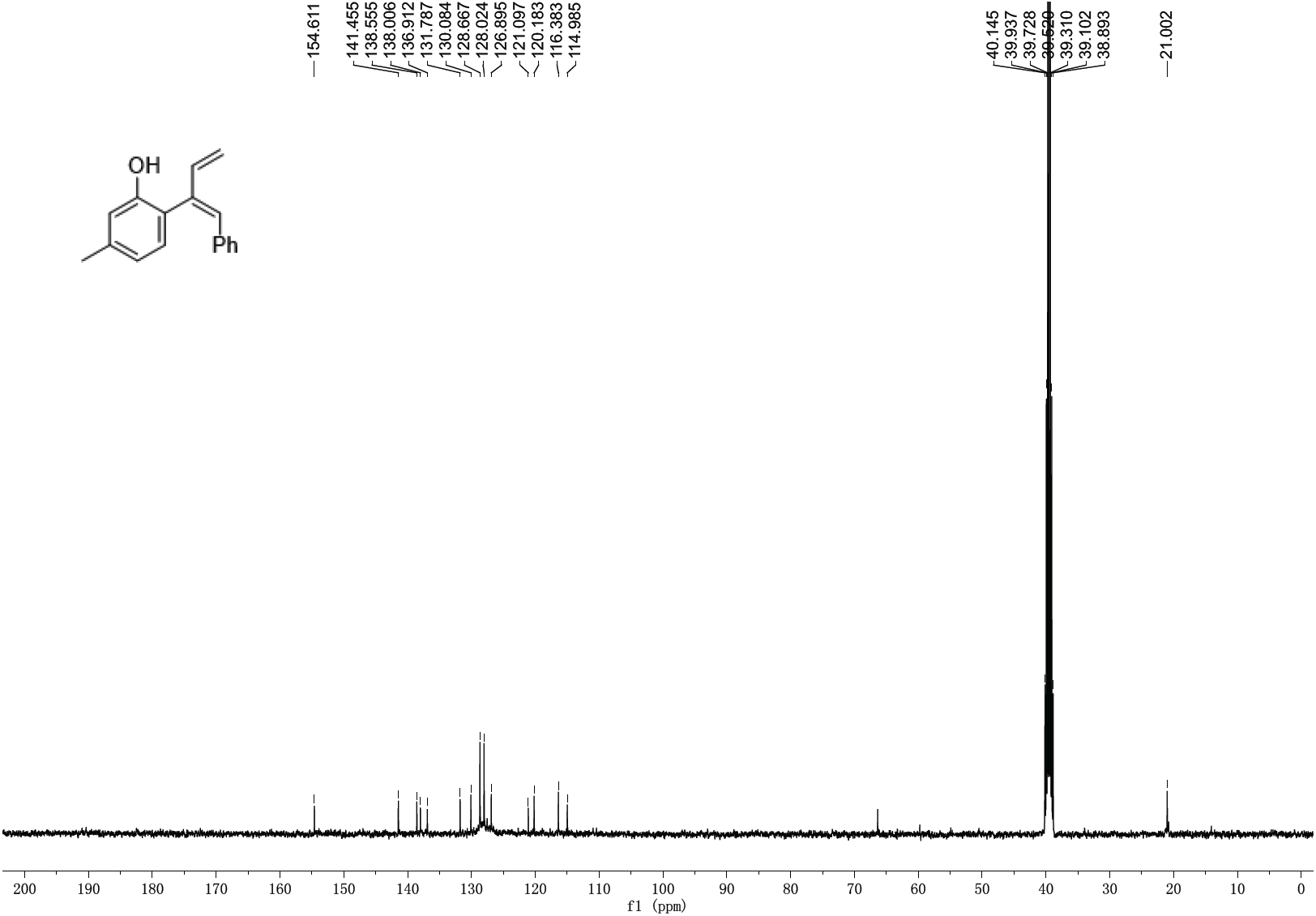
**

**Supplementary Figure 39.** ^13^C-NMR spectrum of **3n**

**3o**

**
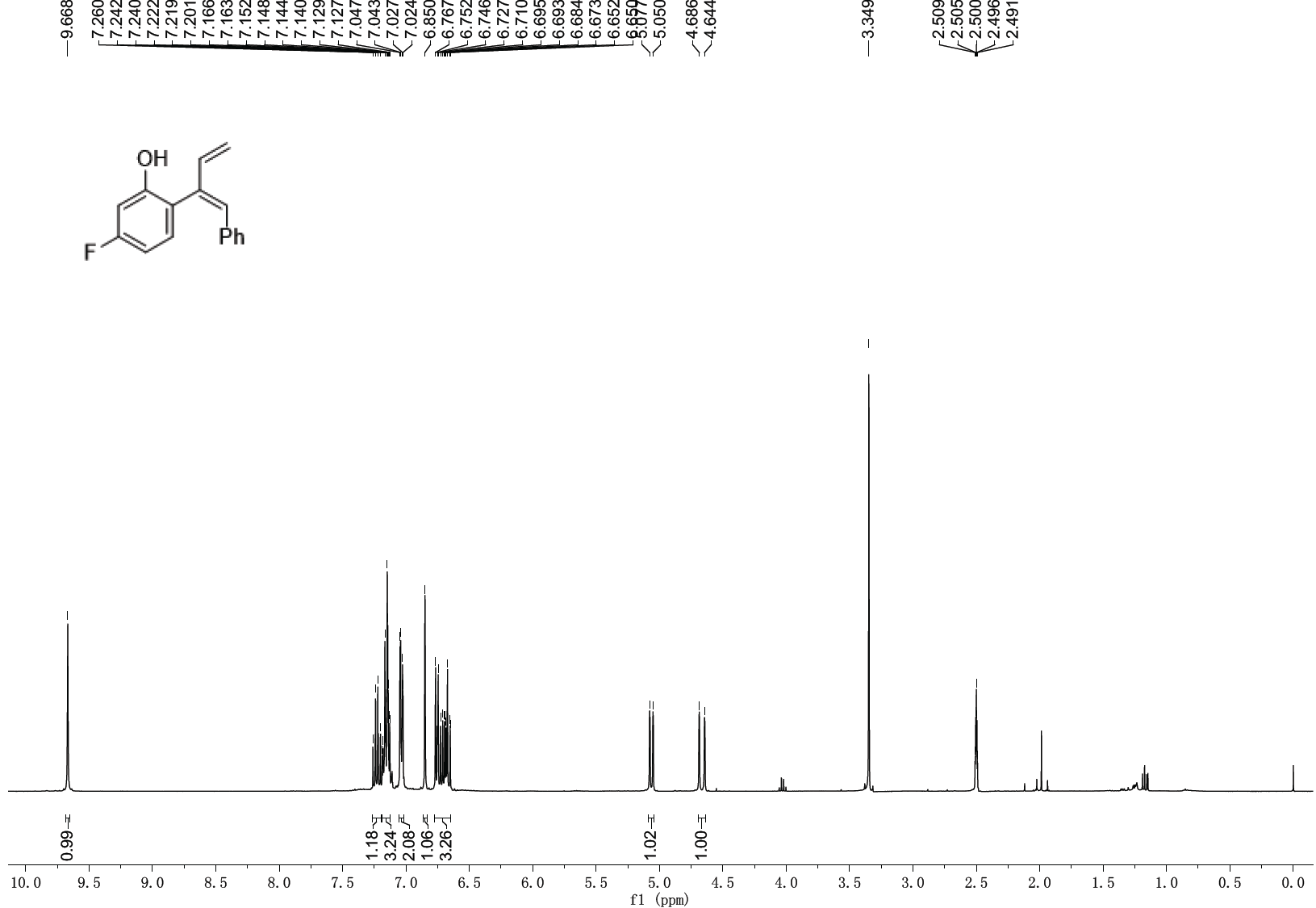
**

**Supplementary Figure 40.** ^1^H-NMR spectrum of **3o**

**
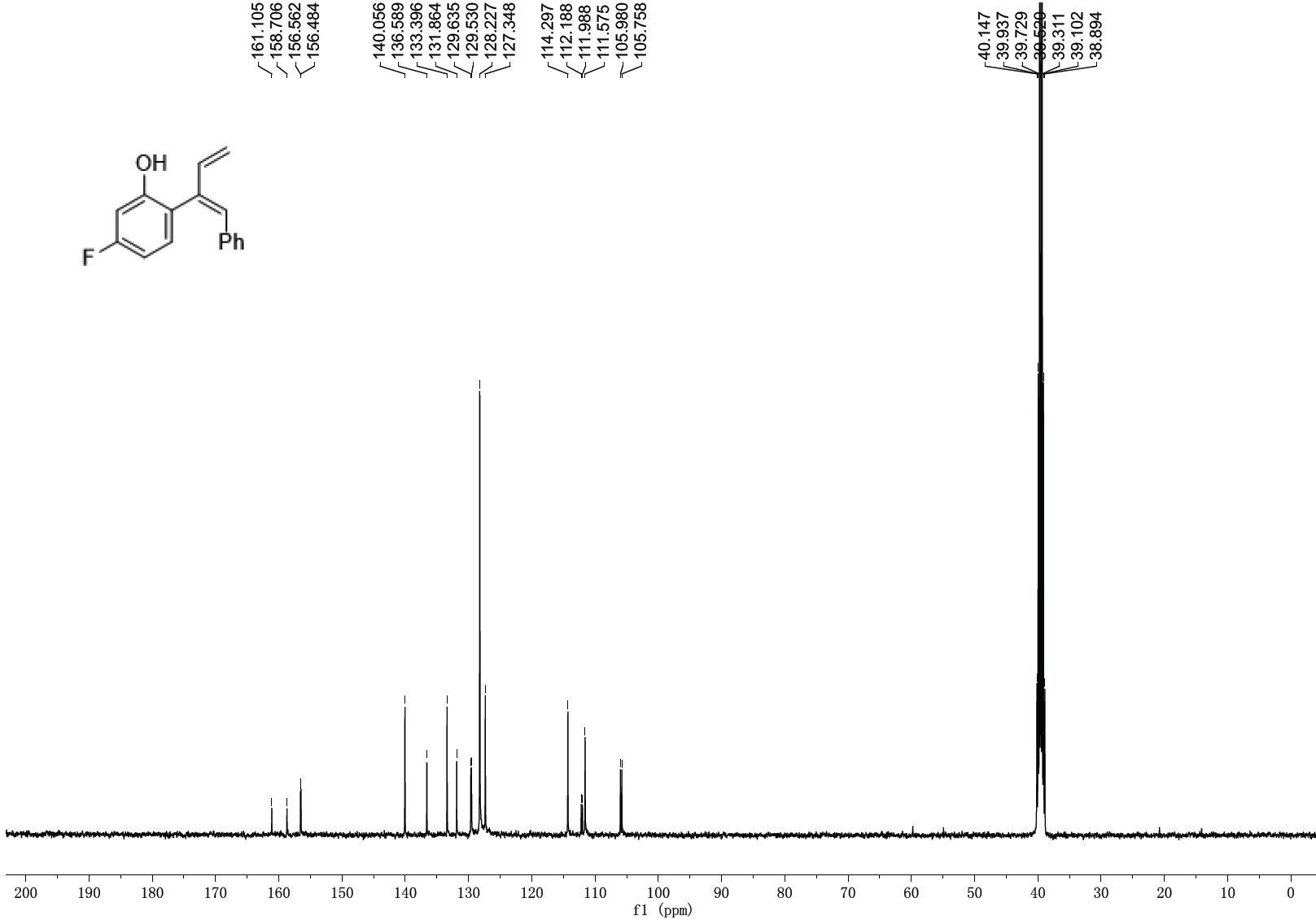
**

**Supplementary Figure 41.** ^13^C-NMR spectrum of **3o**

**
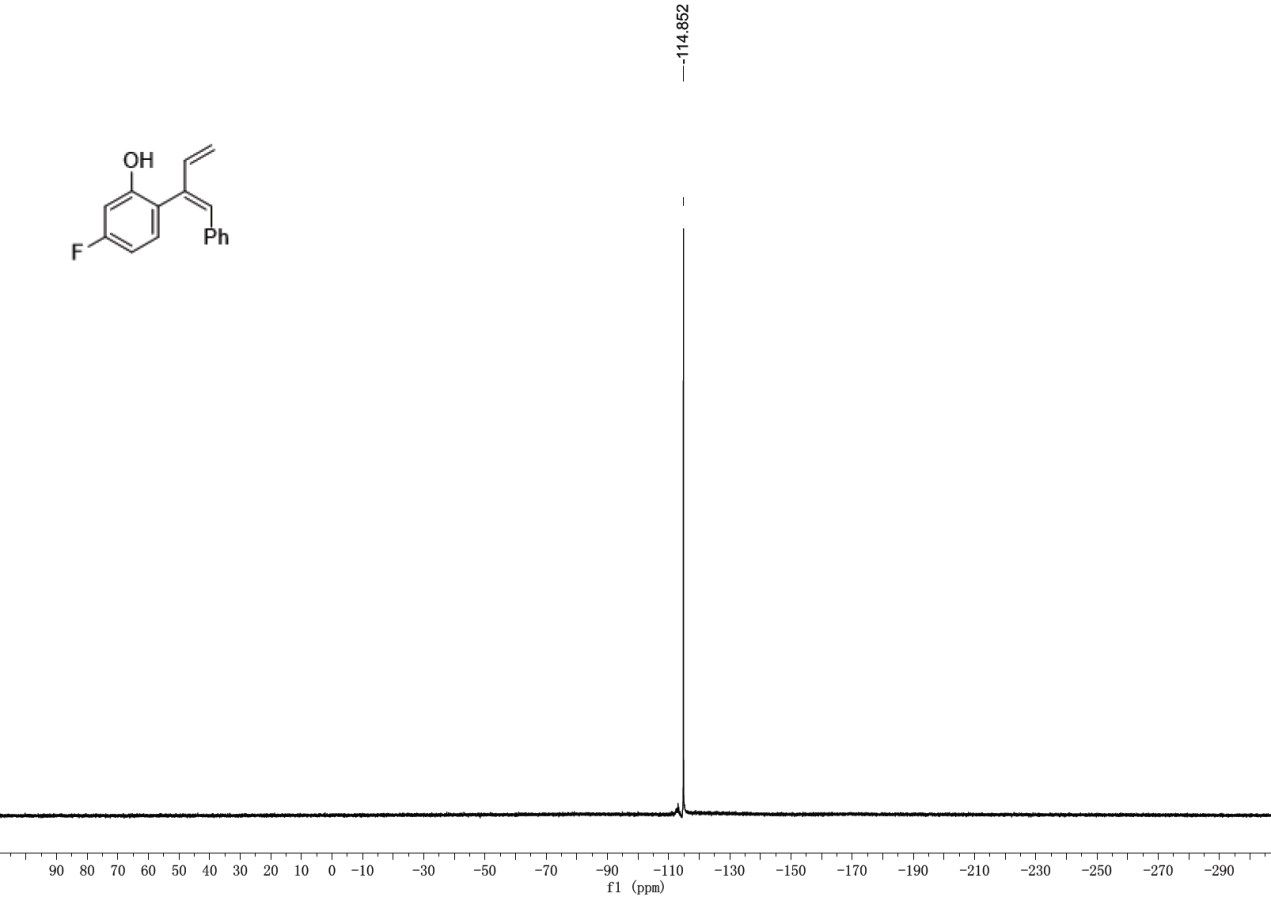
**

**Supplementary Figure 42.** ^19^F-NMR spectrum of **3o**

**3p**

**
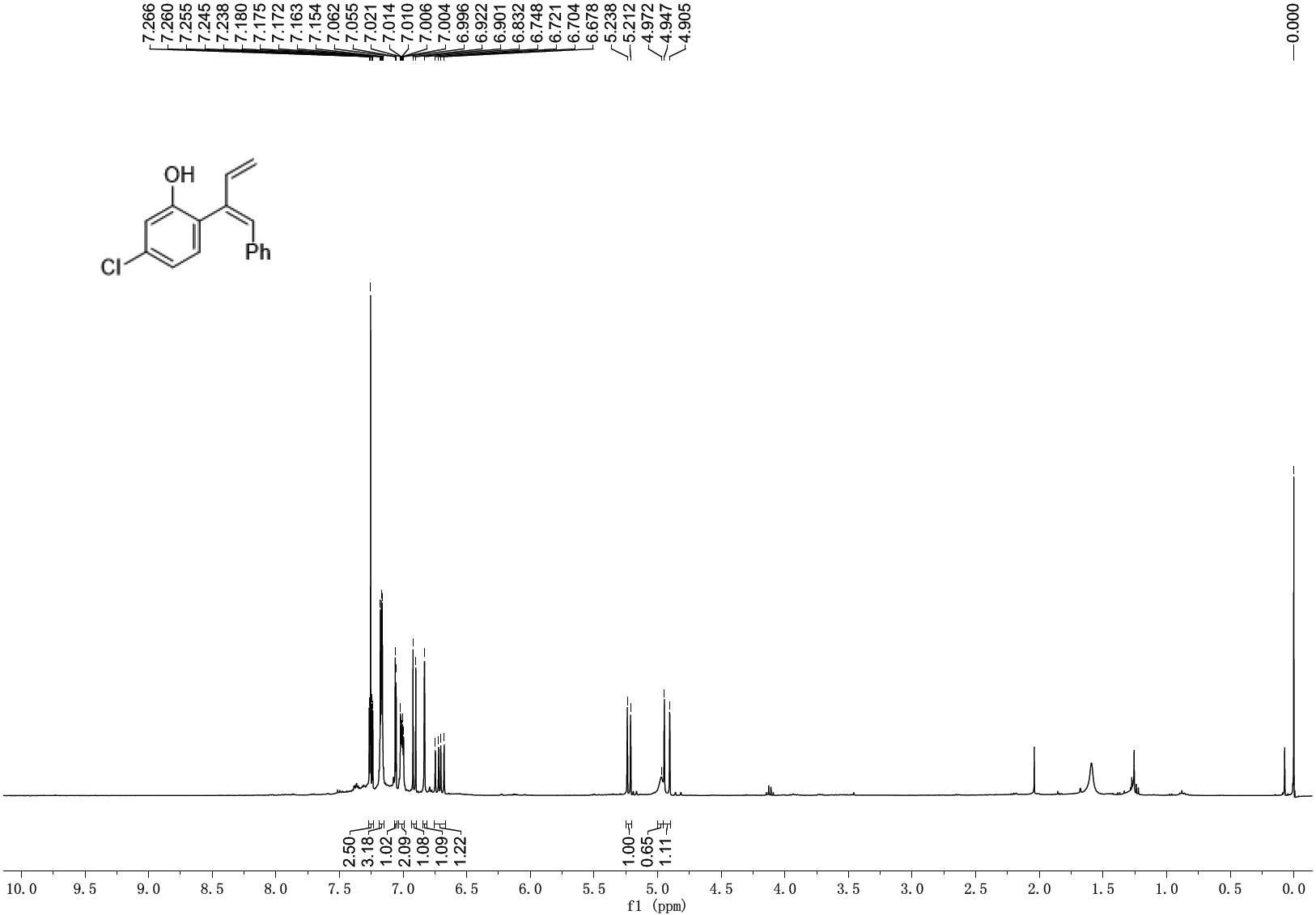
**

**Supplementary Figure 43.** ^1^H-NMR spectrum of **3p**

**
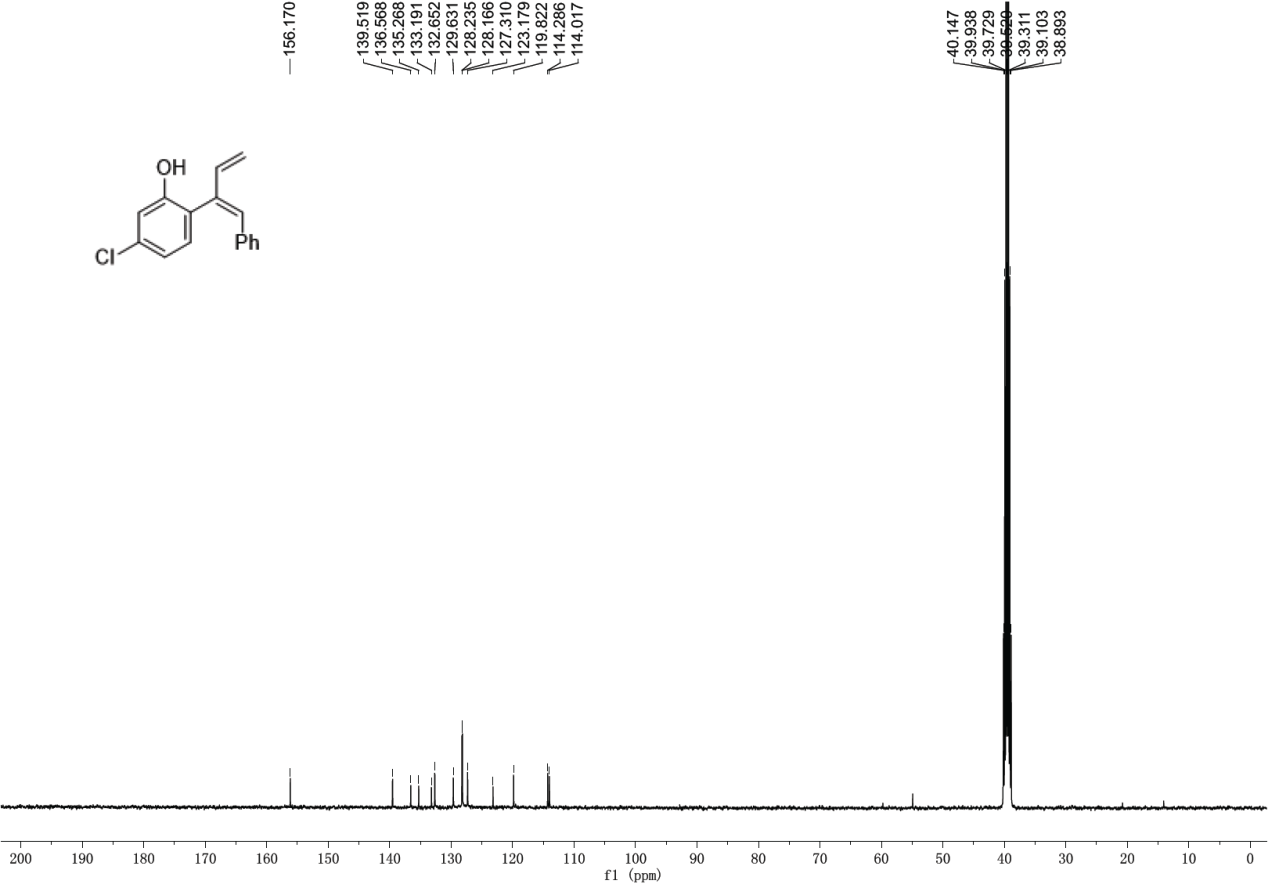
**

**Supplementary Figure 44.** ^13^C-NMR spectrum of **3p**

**3p’**

**
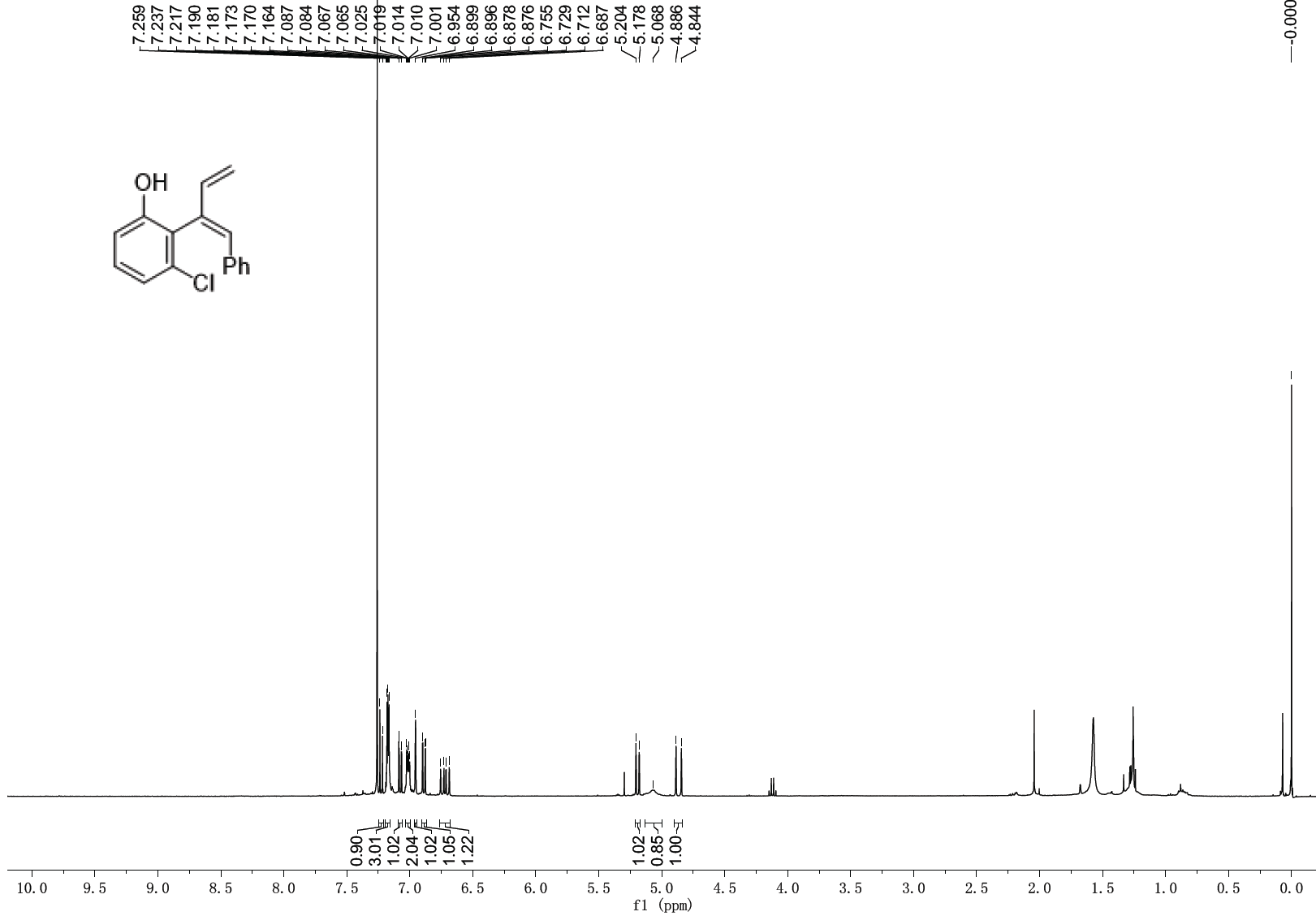
**

**Supplementary Figure 45.** ^1^H-NMR spectrum of **3p’**

**
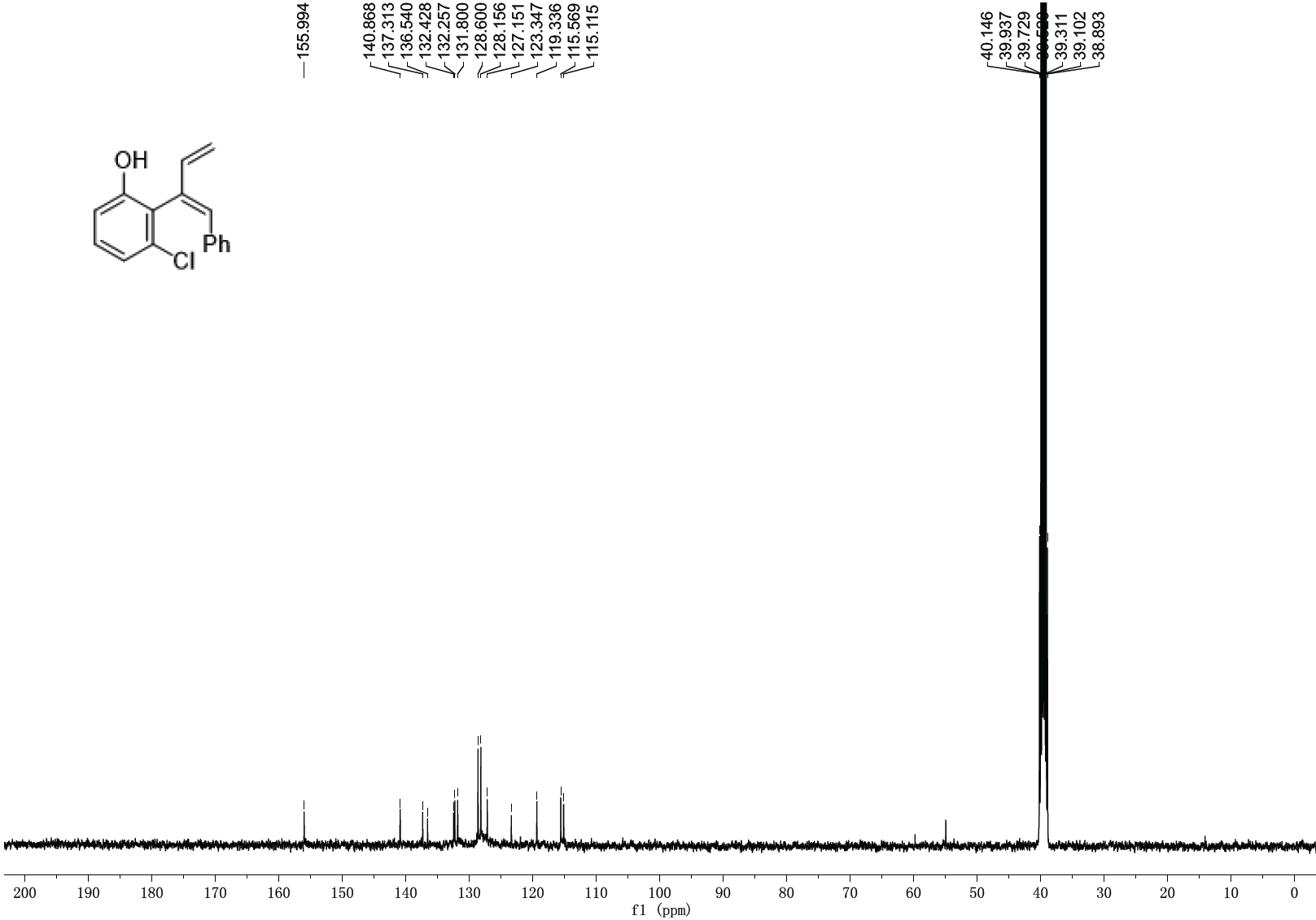
**

**Supplementary Figure 46.** ^13^C-NMR spectrum of **3p’**

**3q**

**
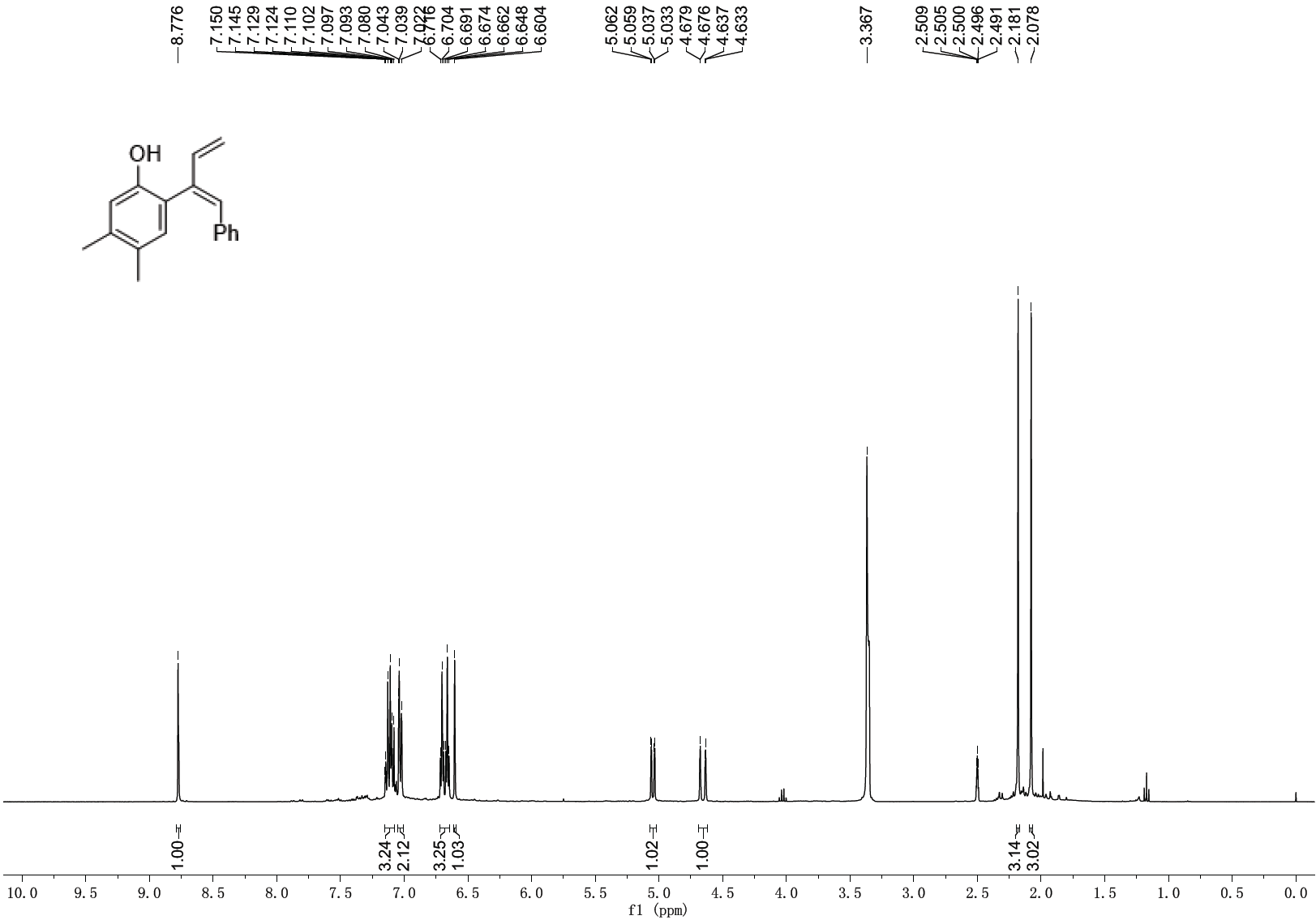
**

**Supplementary Figure 47.** ^1^H-NMR spectrum of **3q**

**
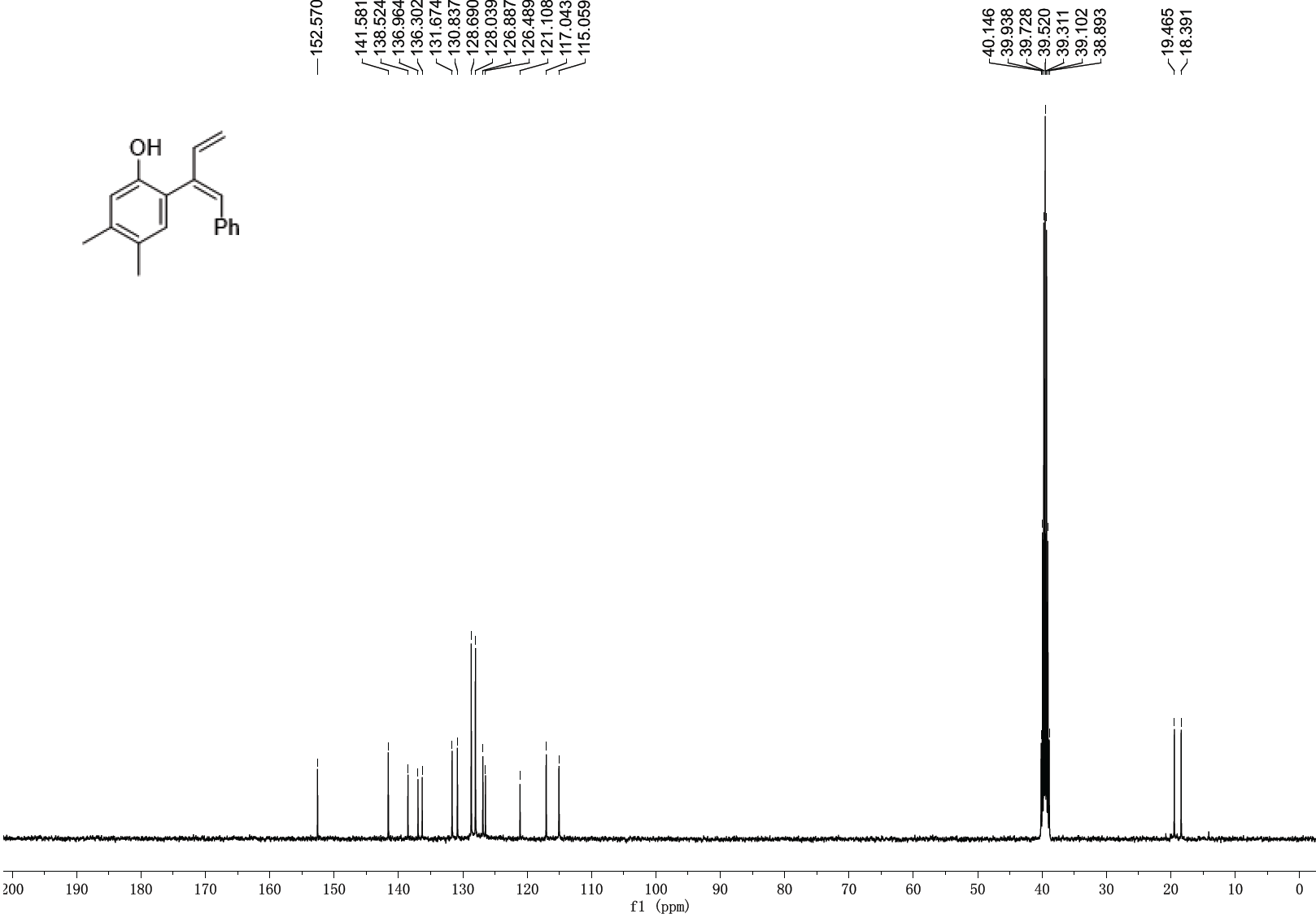
**

**Supplementary Figure 48.** ^13^C-NMR spectrum of **3q**

**3r**

**
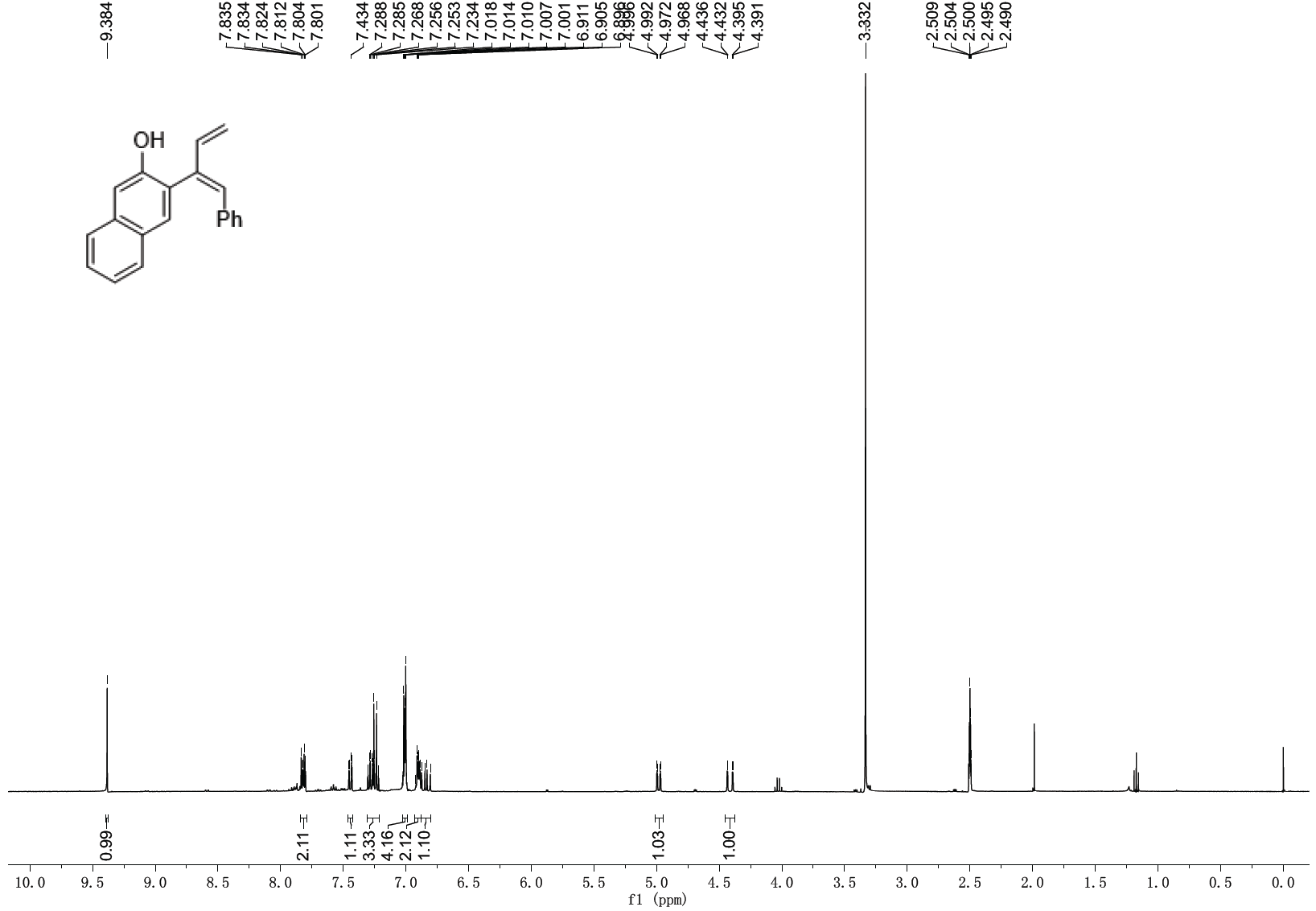
**

**Supplementary Figure 49.** ^1^H-NMR spectrum of **3r**

**
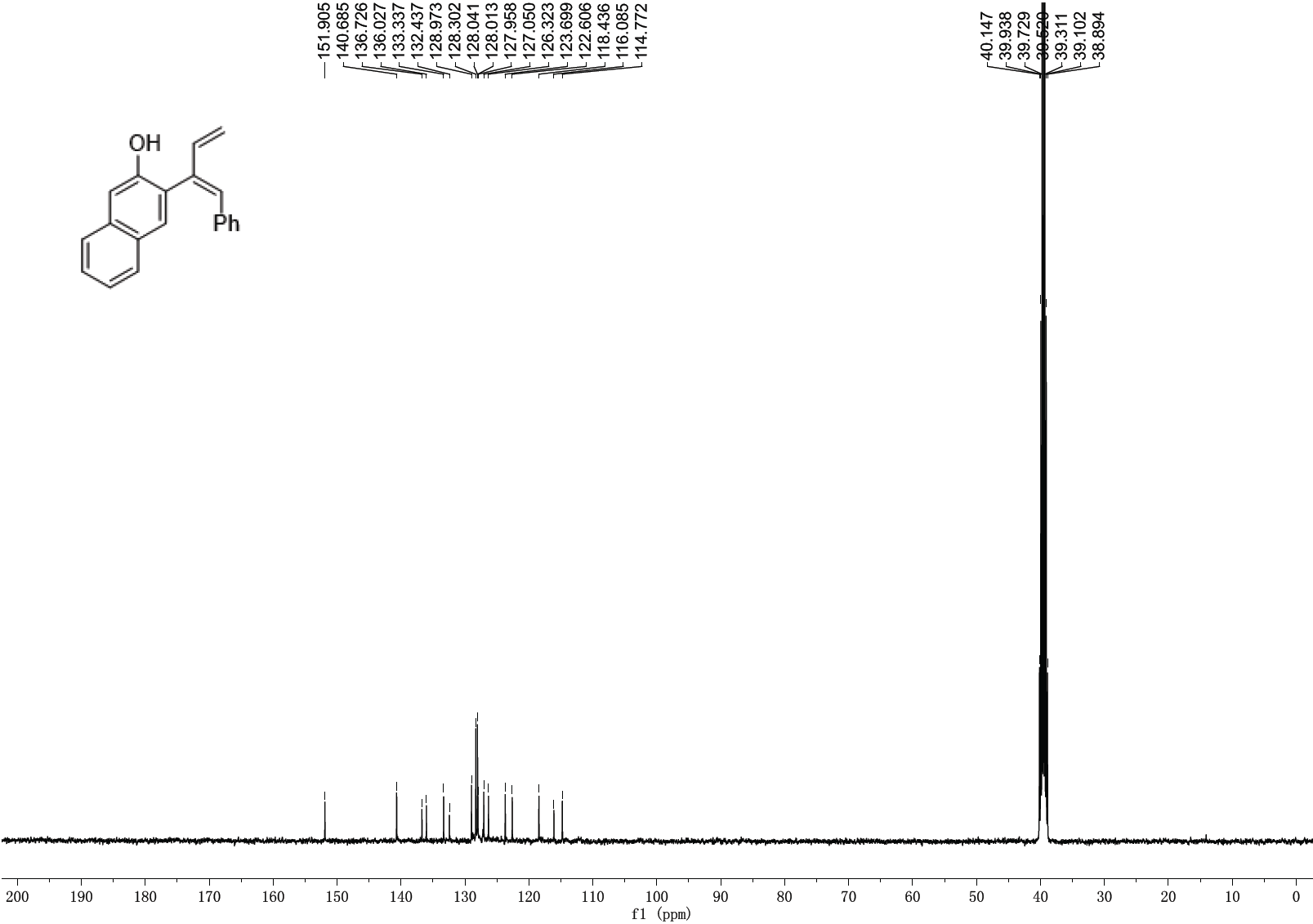
**

**Supplementary Figure 50.** ^13^C-NMR spectrum of **3r**

**3s**

**
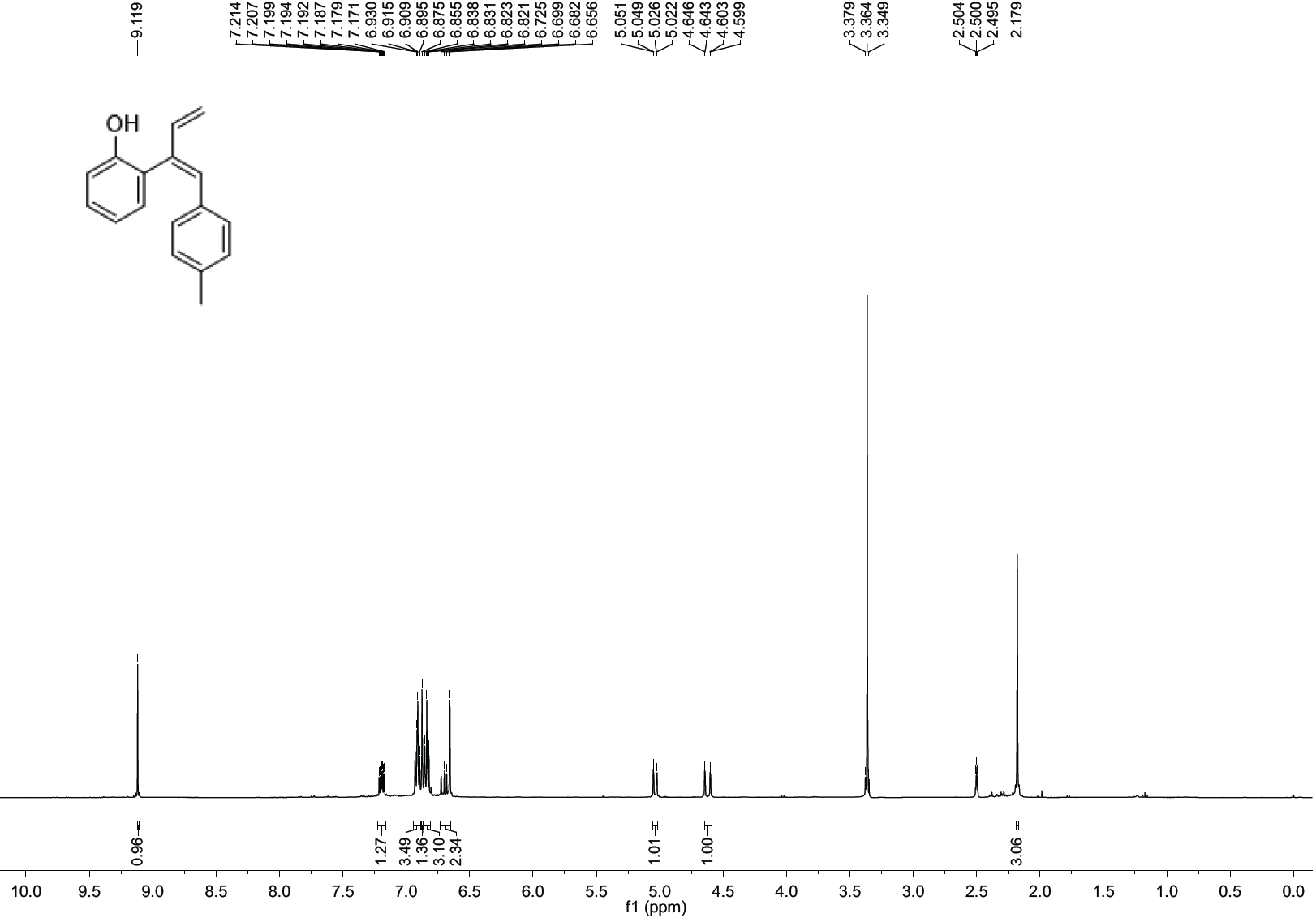
**

**Supplementary Figure 51.** ^1^H-NMR spectrum of **3s**

**
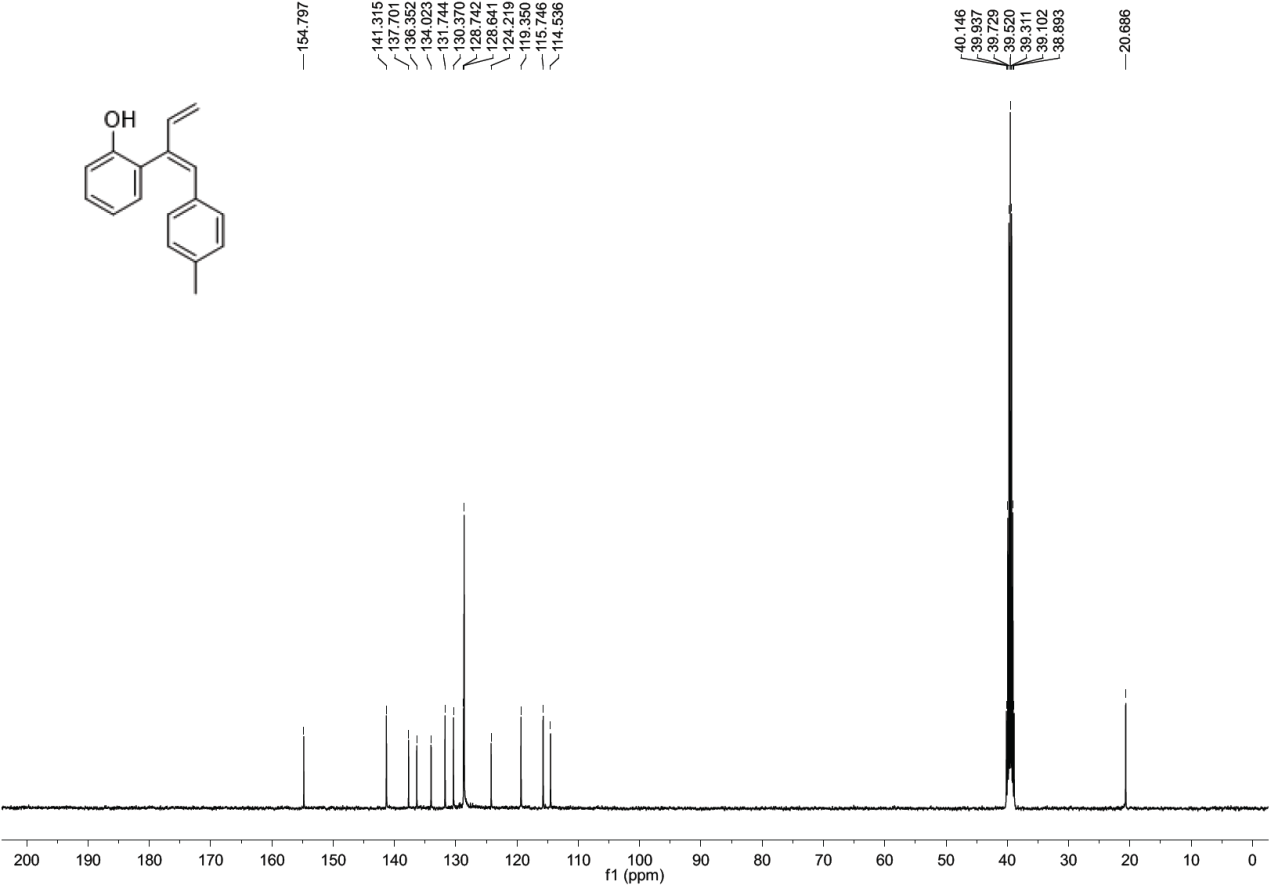
**

**Supplementary Figure 52.** ^13^C-NMR spectrum of **3s**

**3t**

**
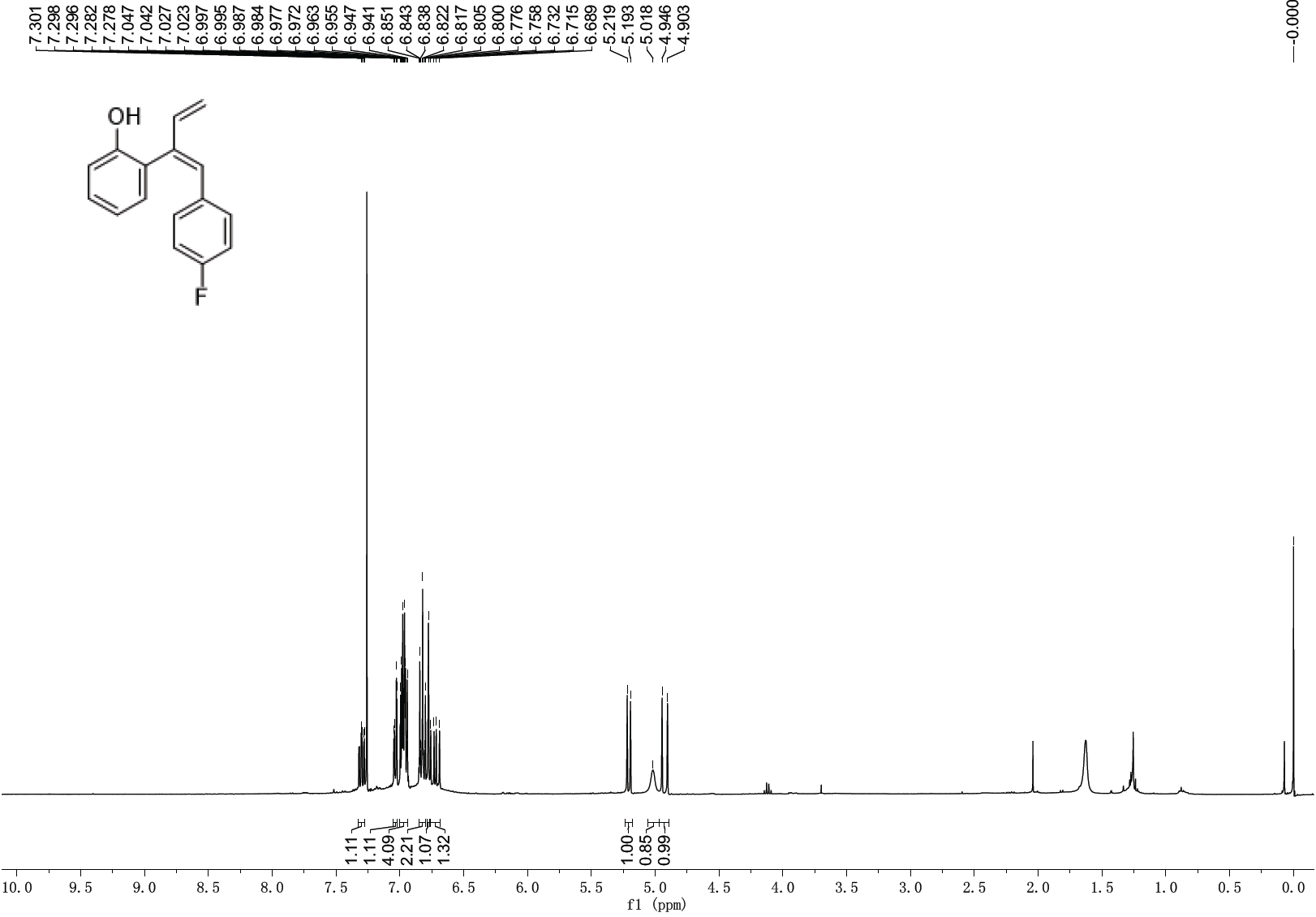
**

**Supplementary Figure 53.** ^1^H-NMR spectrum of **3t**

**
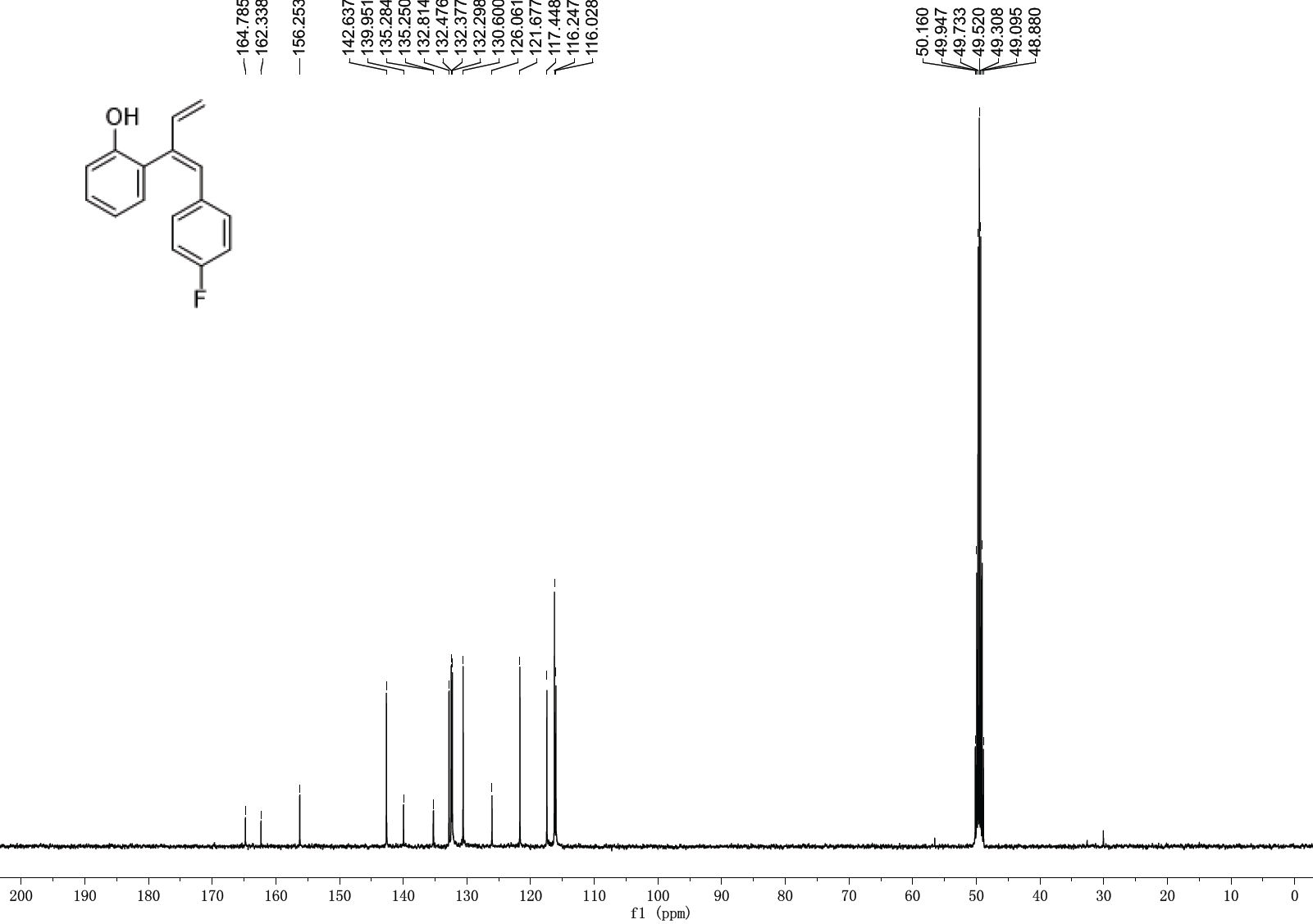
**

**Supplementary Figure 54.** ^13^C-NMR spectrum of **3t**

**
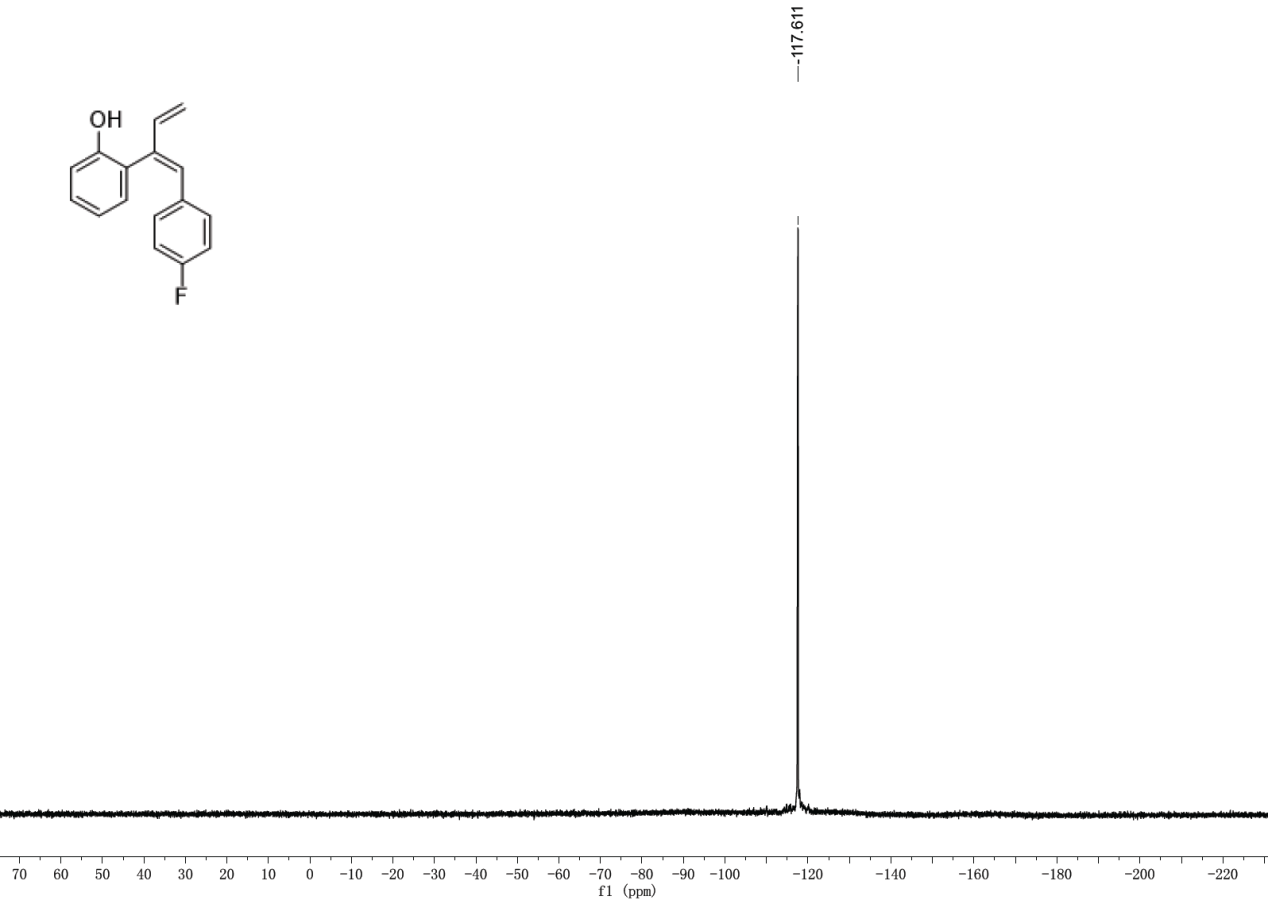
**

**Supplementary Figure 55.** ^19^F-NMR spectrum of **3t**

**3u**

**
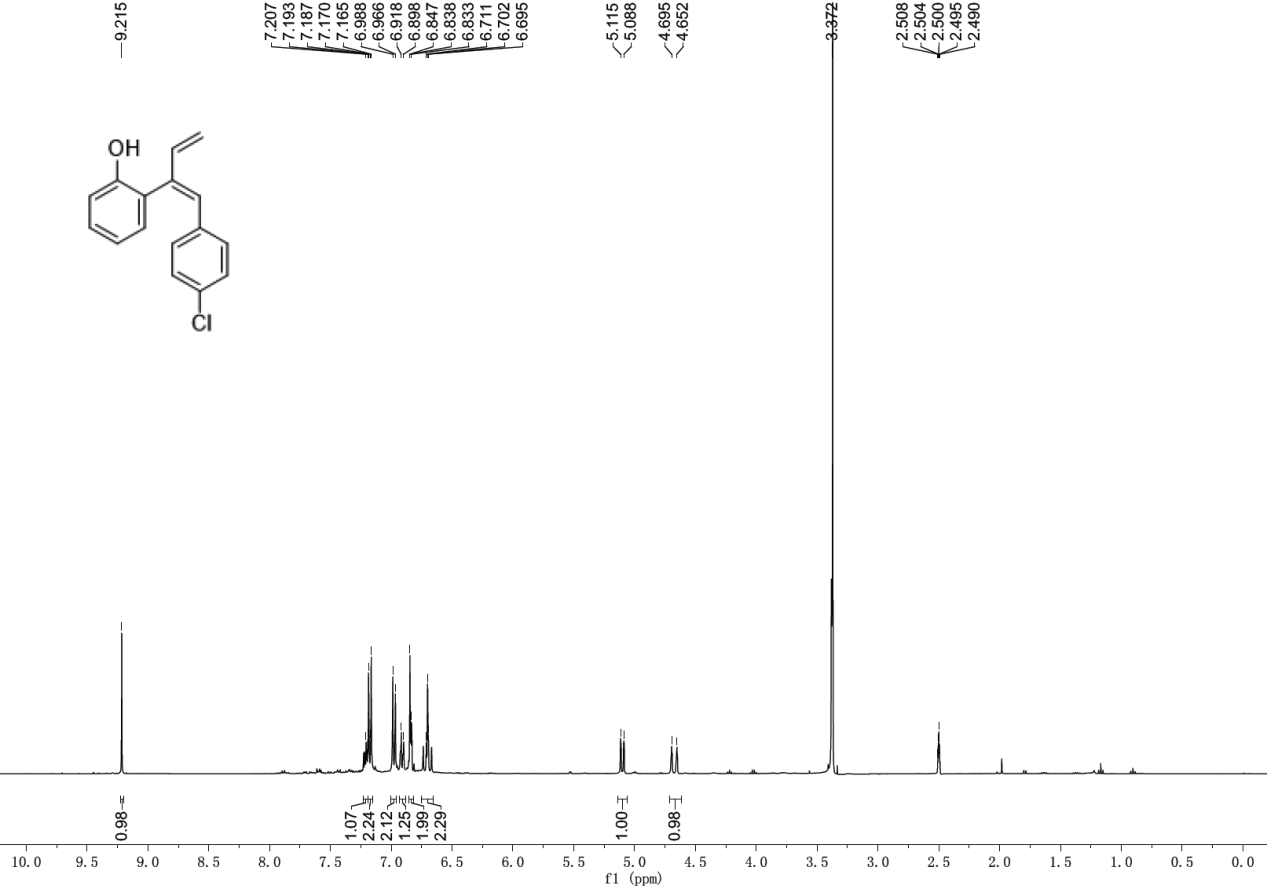
**

**Supplementary Figure 56.** ^1^H-NMR spectrum of **3u**

**
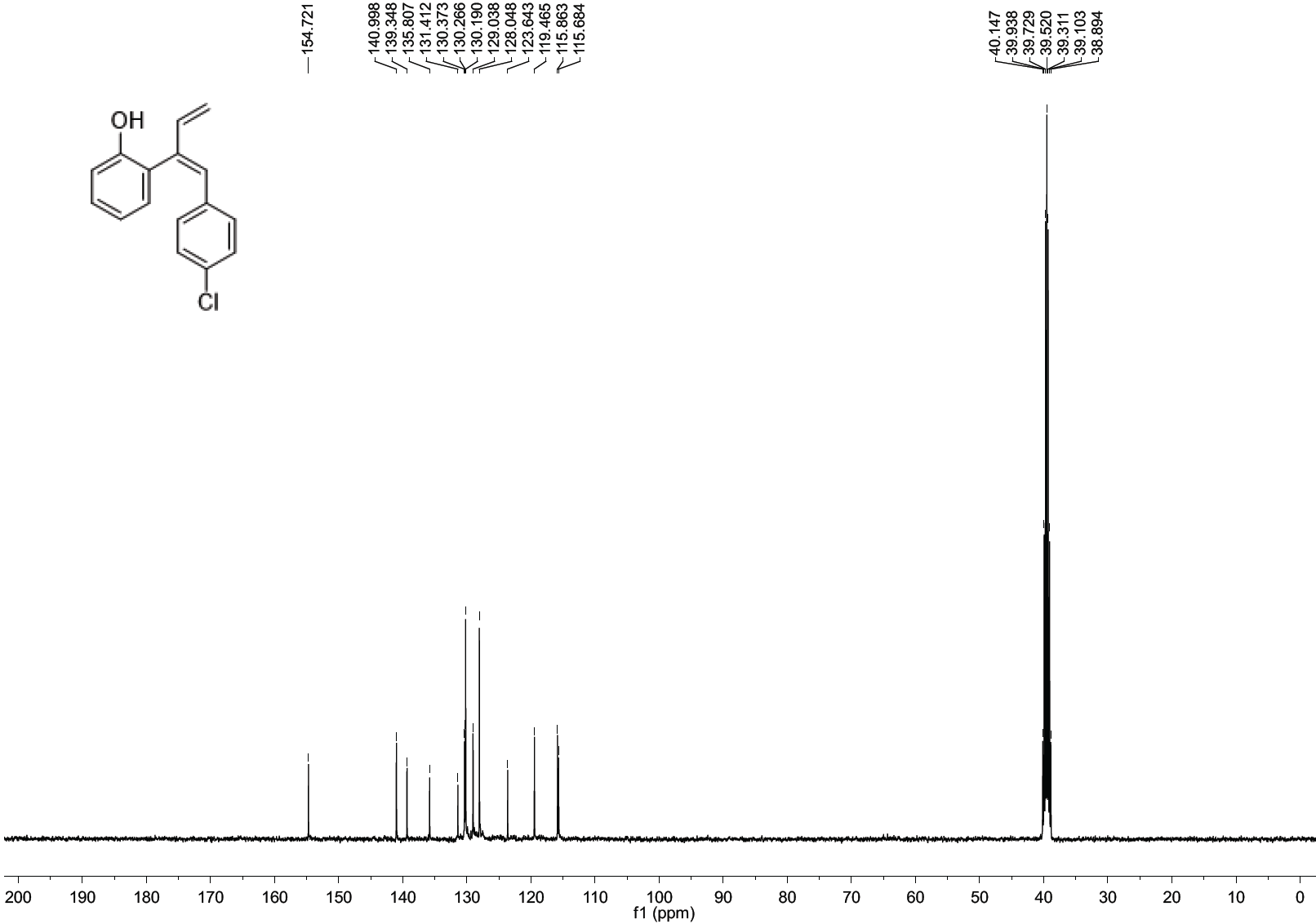
**

**Supplementary Figure 57.** ^13^C-NMR spectrum of **3u**

**3v**

**
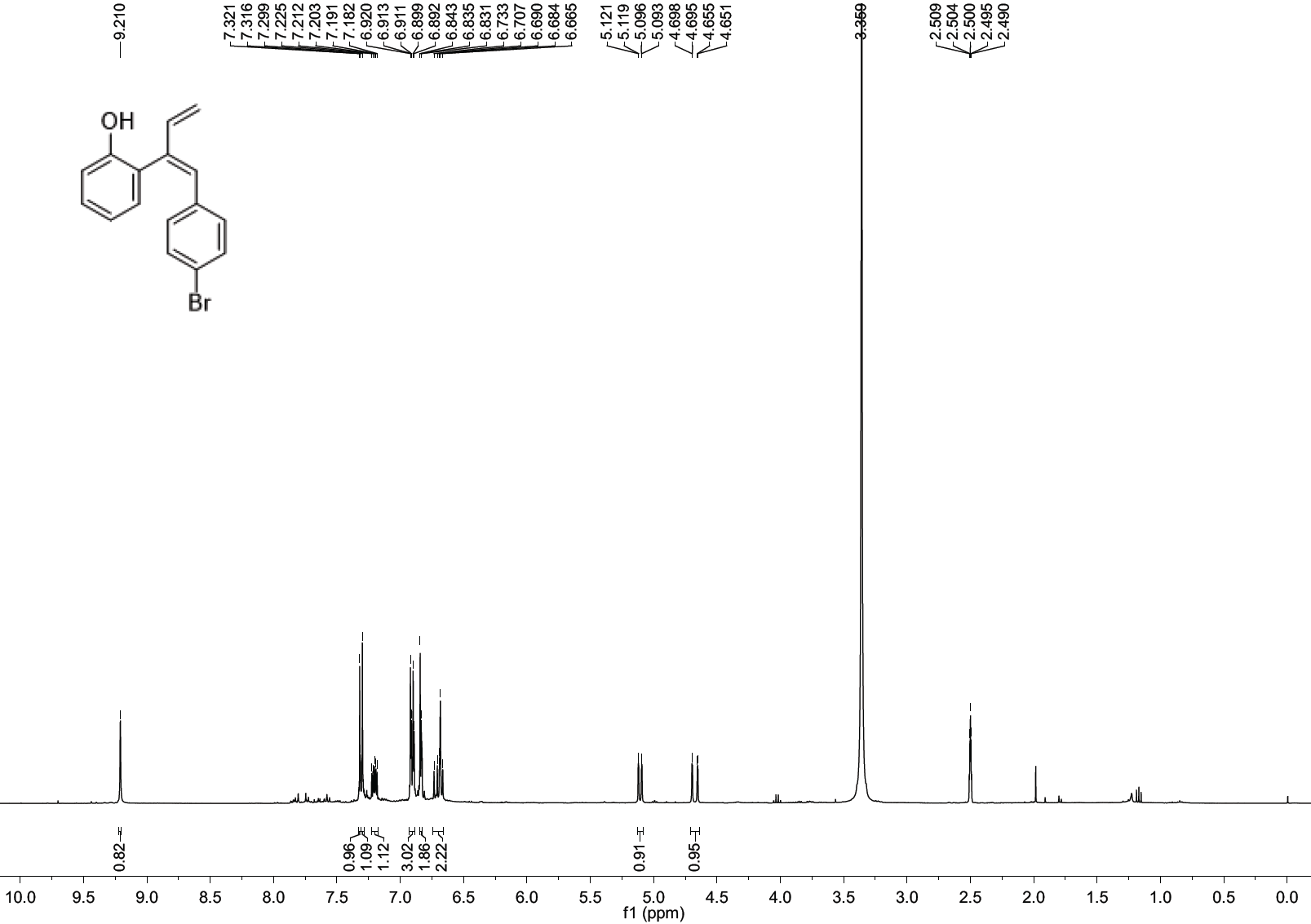
**

**Supplementary Figure 58.** ^1^H-NMR spectrum of **3v**

**
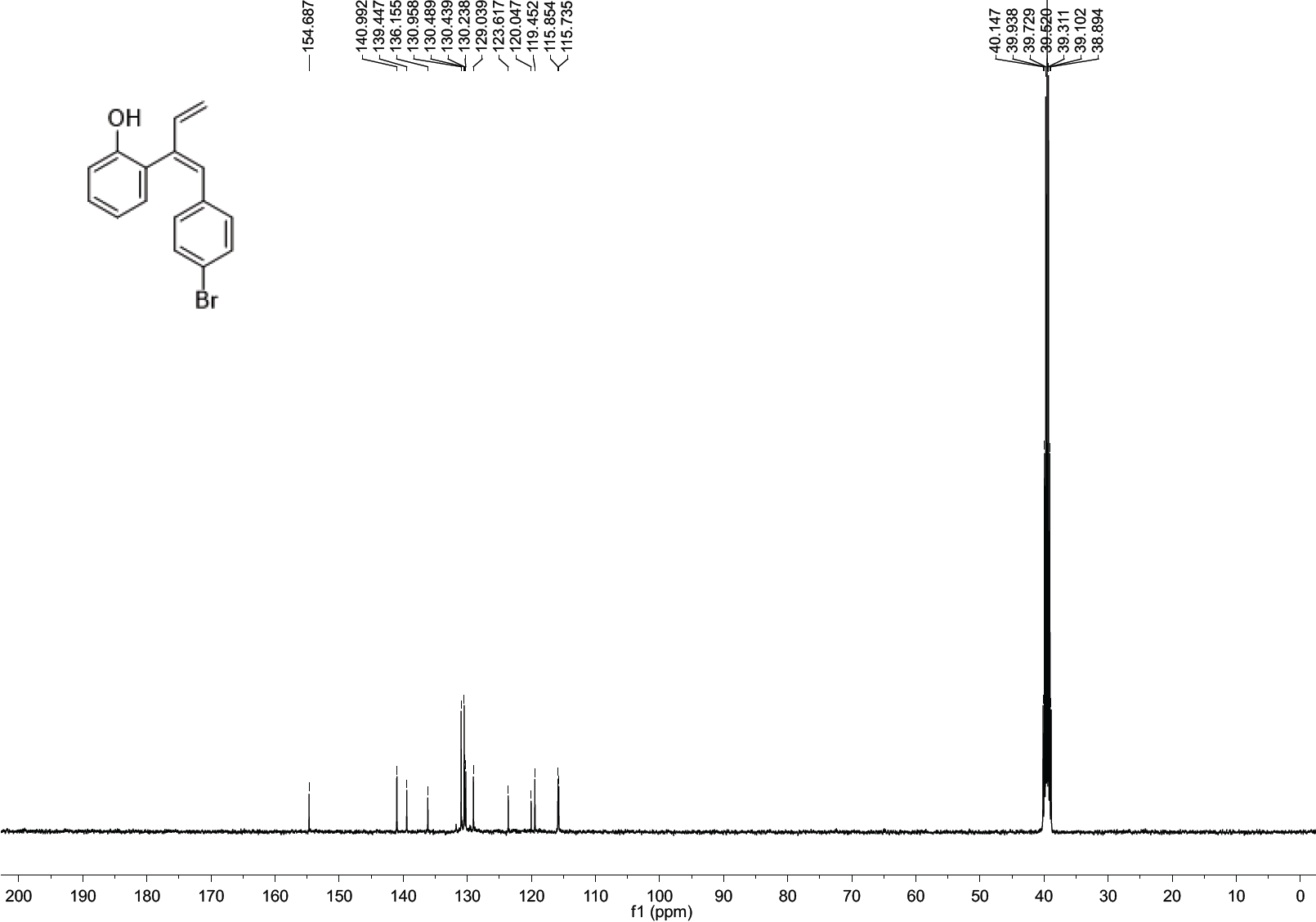
**

**Supplementary Figure 59.** ^13^C-NMR spectrum of **3v**

**3w**

**
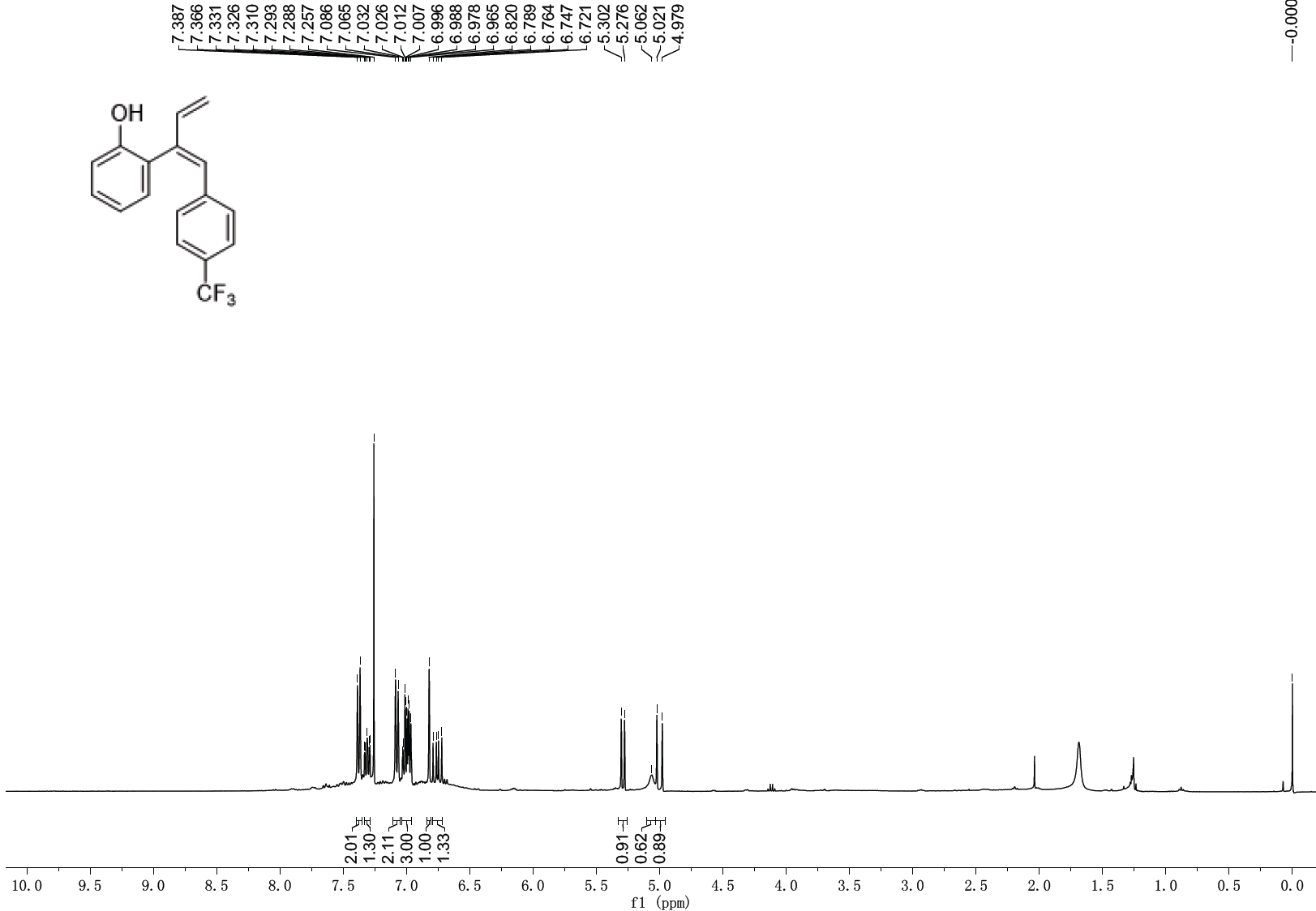
**

**Supplementary Figure 60.** ^1^H-NMR spectrum of **3w**

**
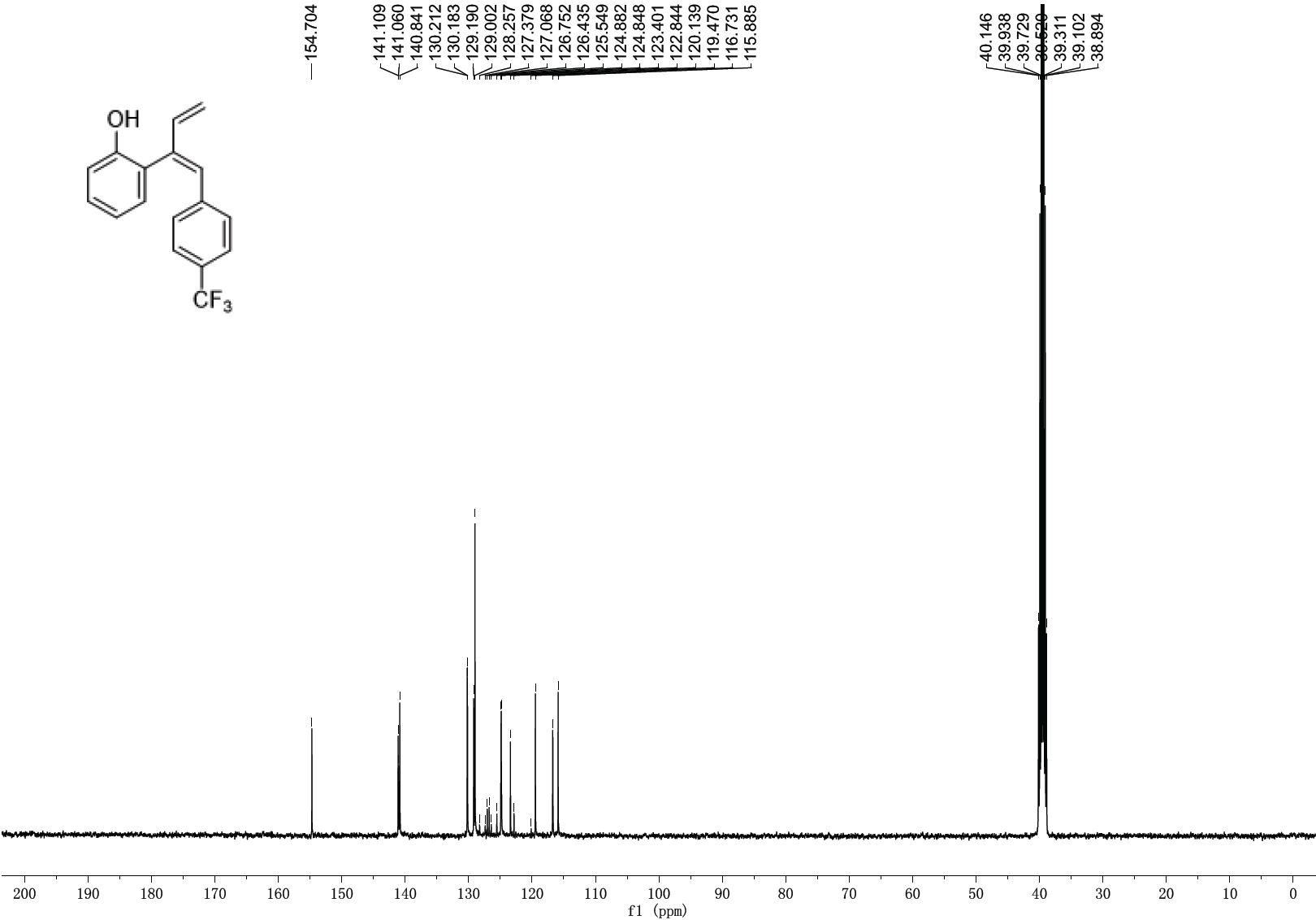
**

**Supplementary Figure 61.** ^13^C-NMR spectrum of **3w**

**
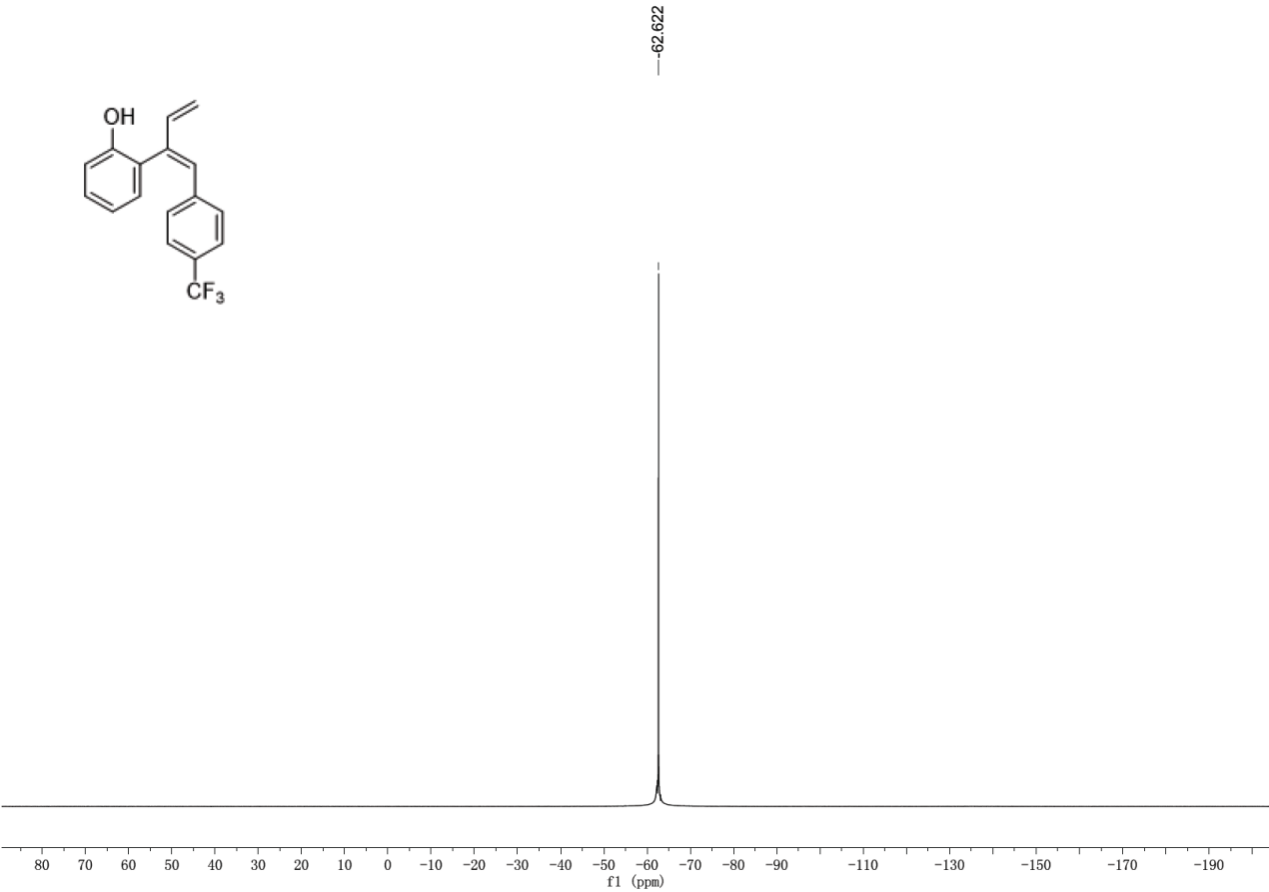
**

**Supplementary Figure 62.** ^19^F-NMR spectrum of **3w**

**3x**

**
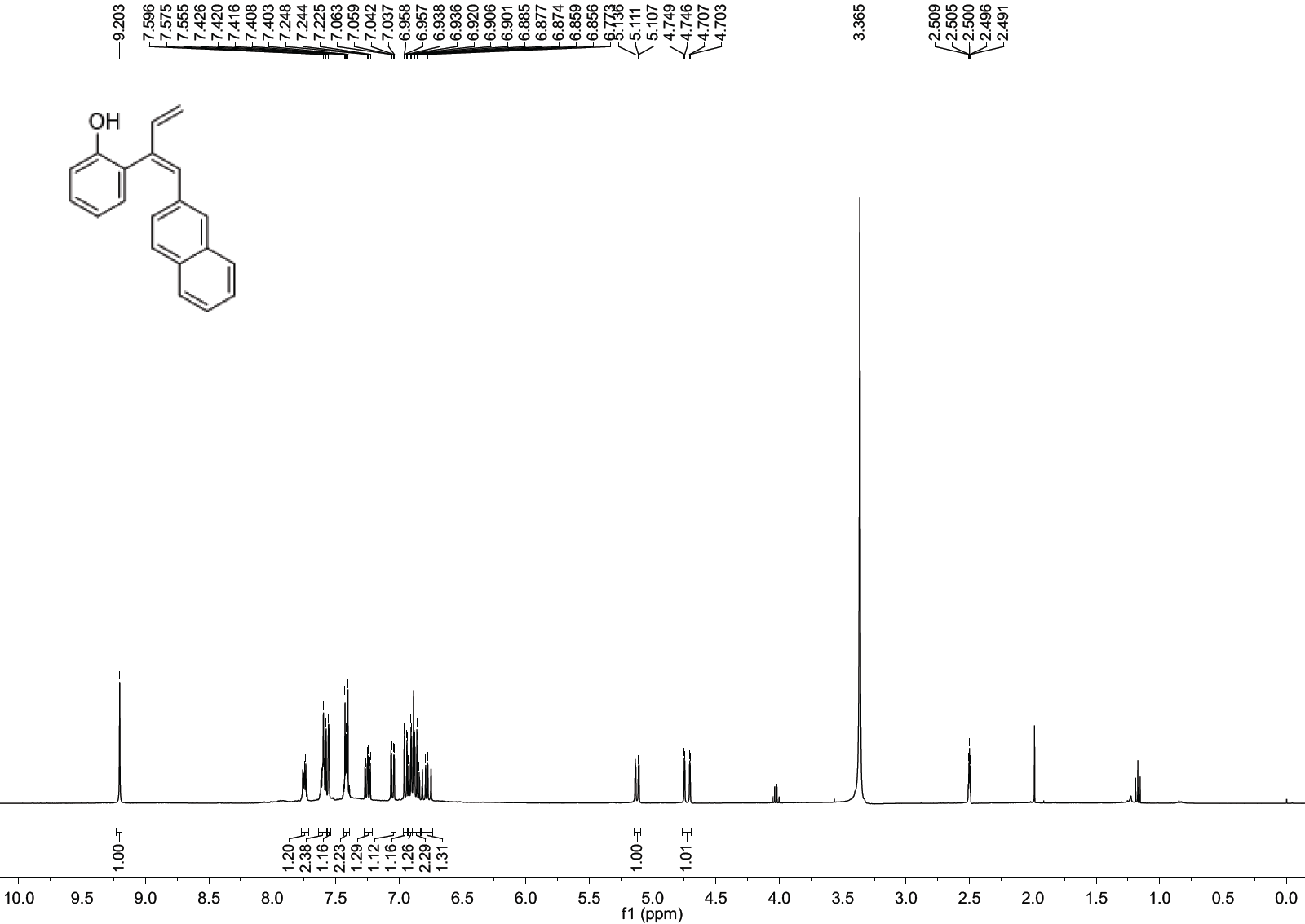
**

**Supplementary Figure 63.** ^1^H-NMR spectrum of **3x**

**
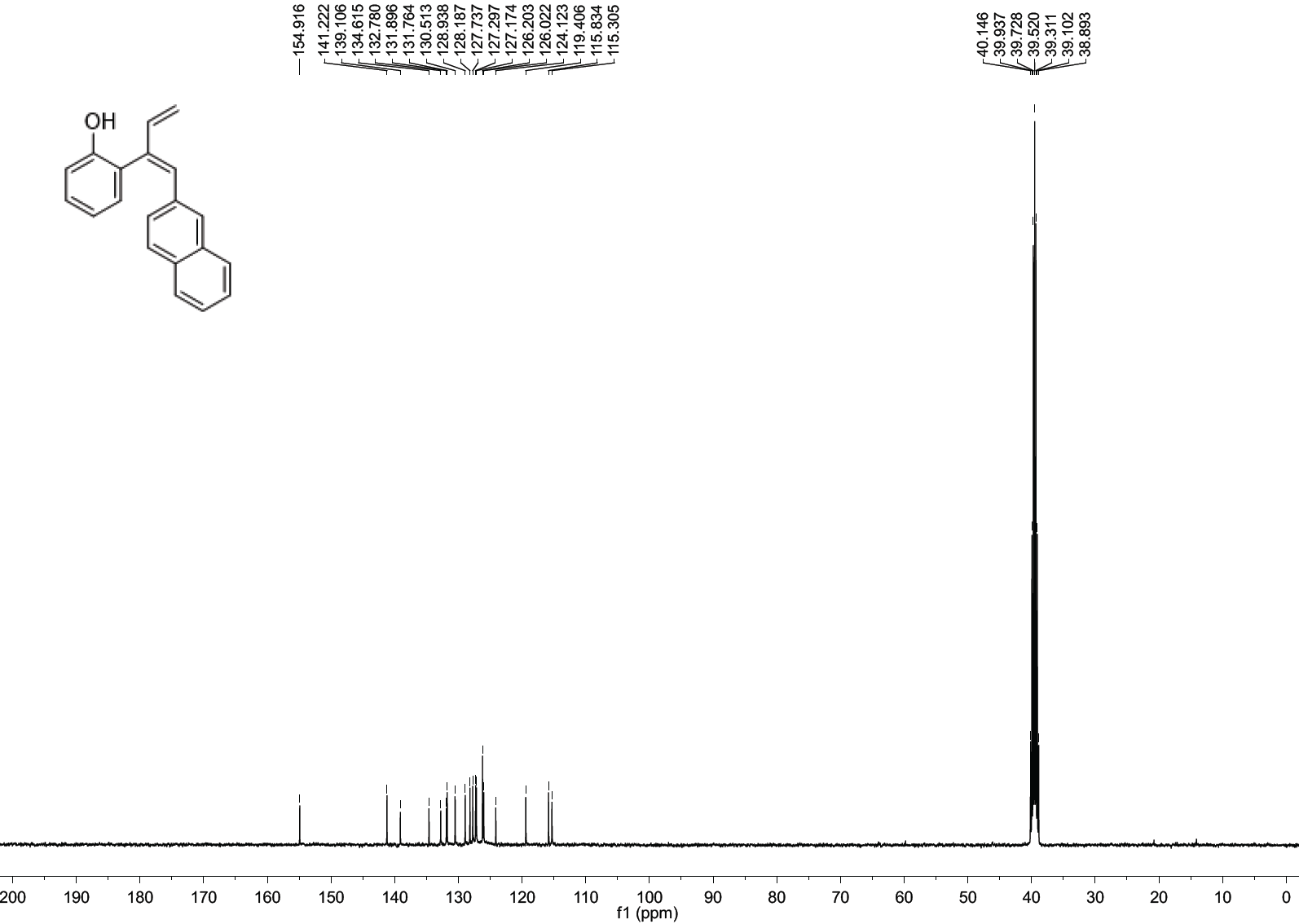
**

**Supplementary Figure 64.** ^13^C-NMR spectrum of **3x**

**3y**

**
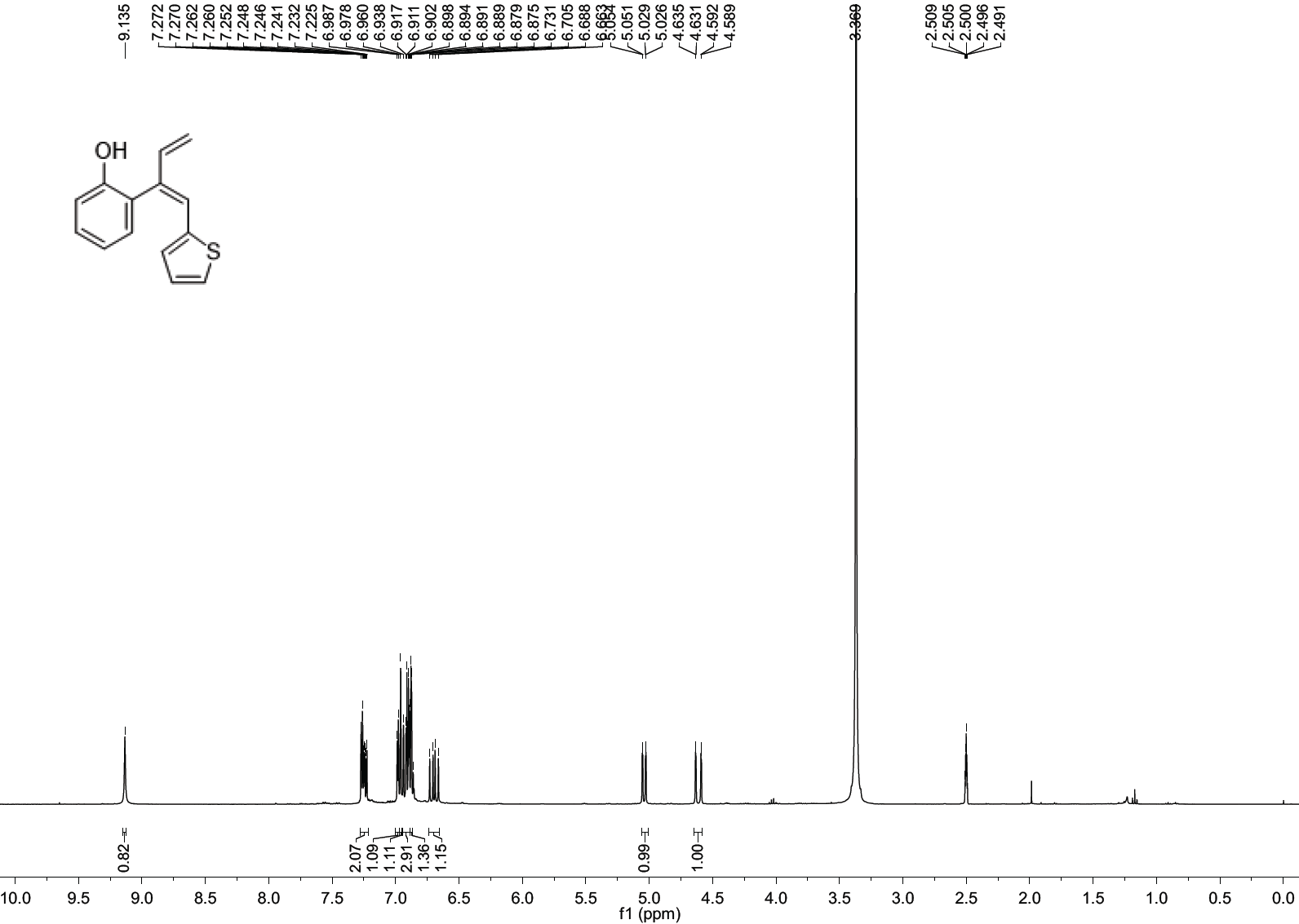
**

**Supplementary Figure 65.** ^1^H-NMR spectrum of **3y**

**
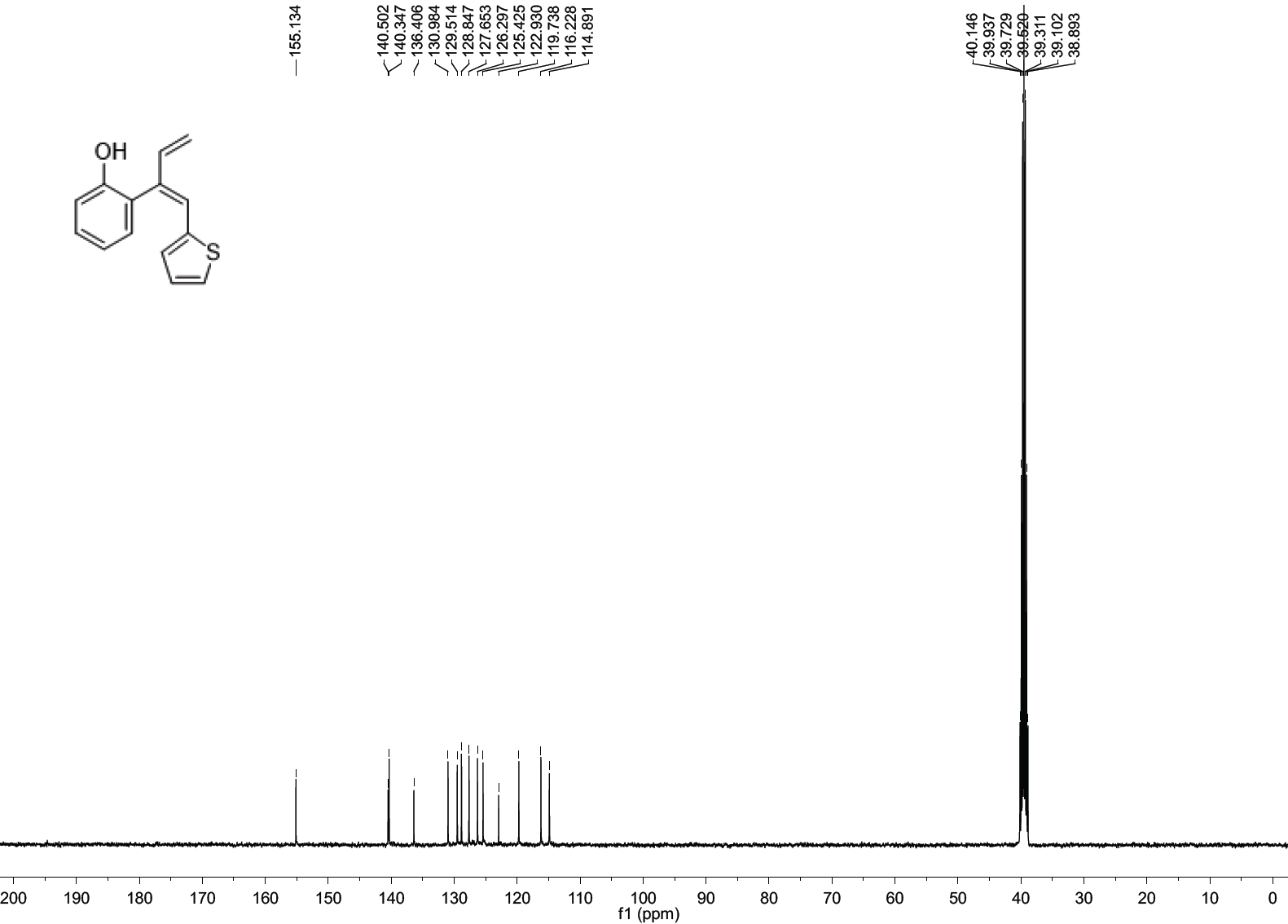
**

**Supplementary Figure 66.** ^13^C-NMR spectrum of **3y**

**3z**


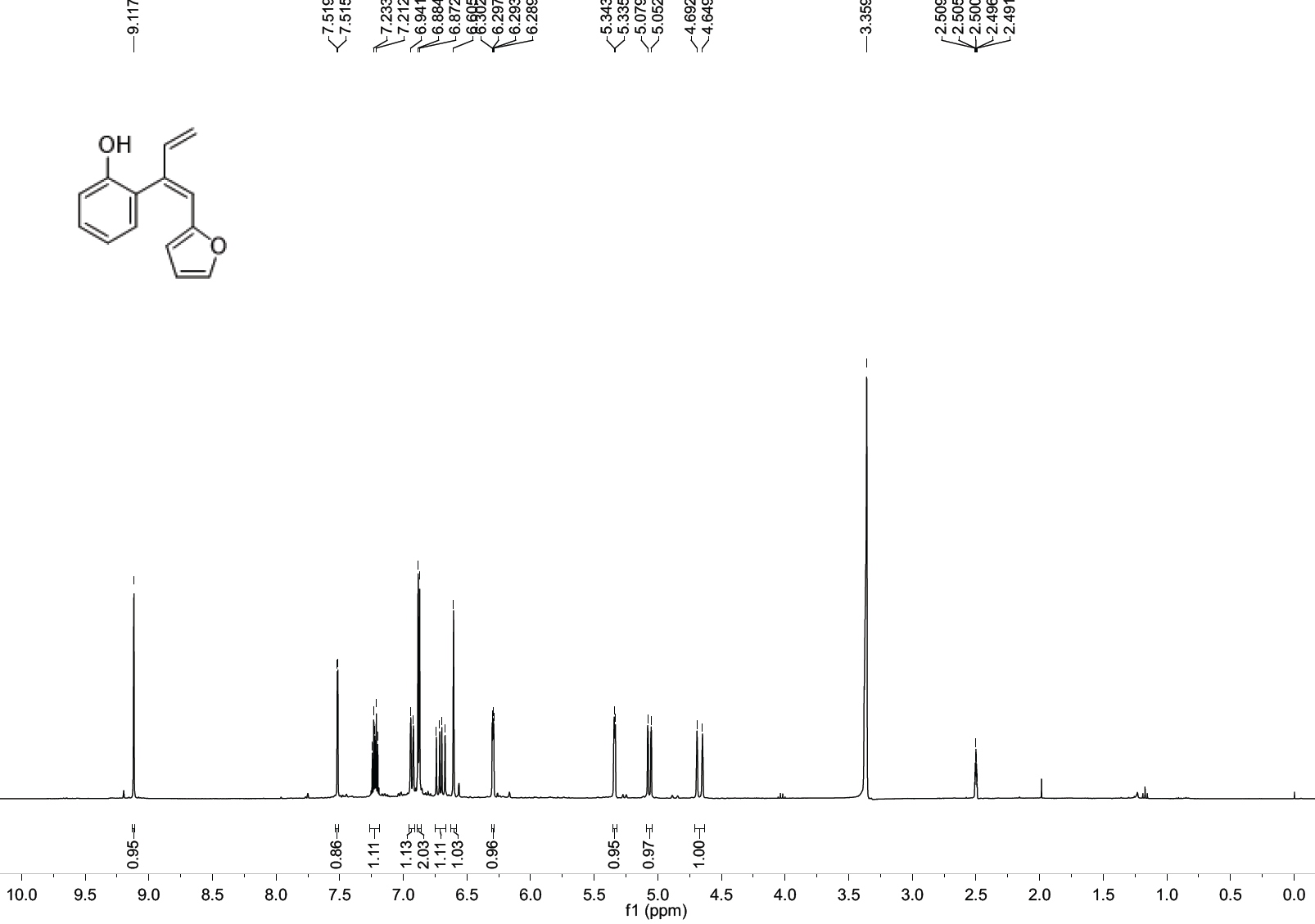


**Supplementary Figure 67.** ^1^H-NMR spectrum of **3z**


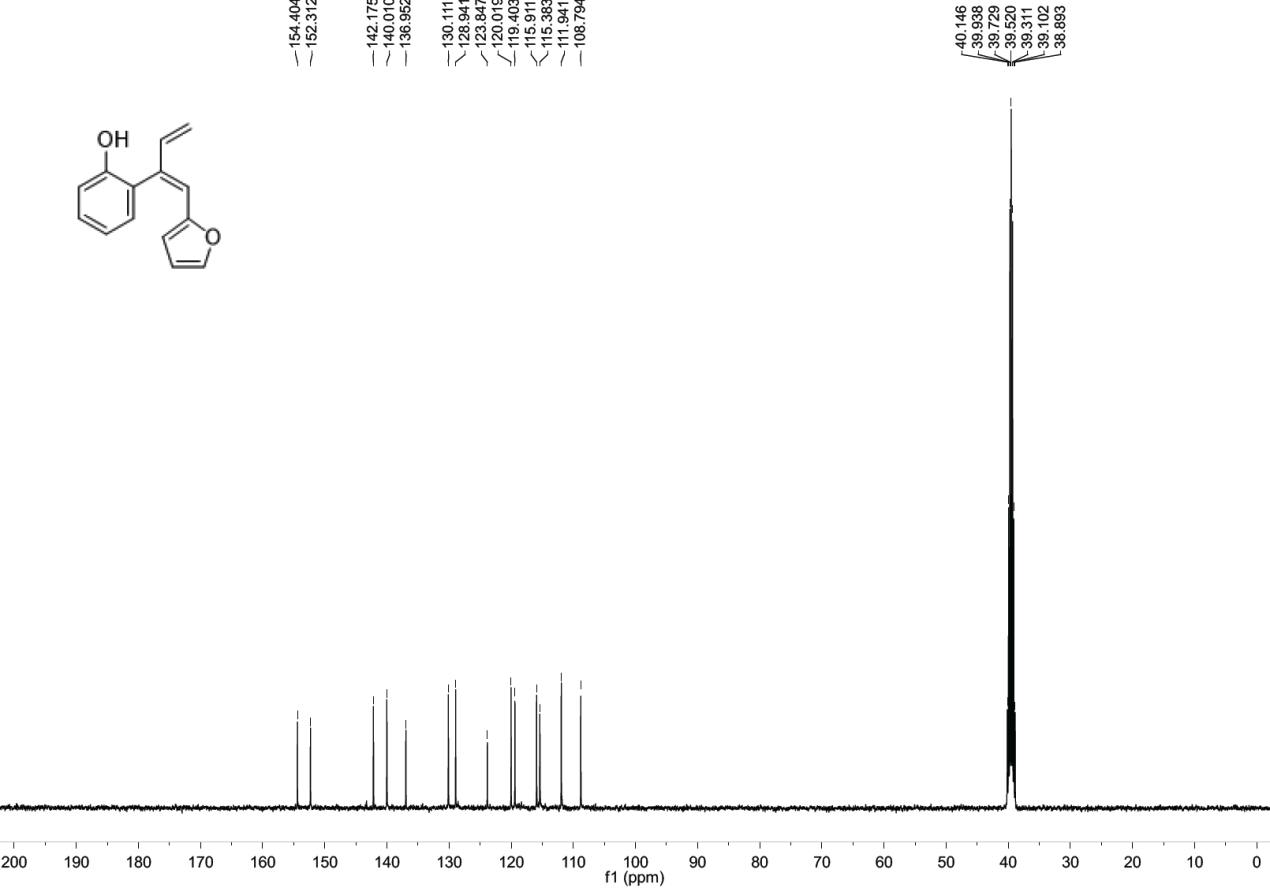


**Supplementary Figure 68.** ^13^C-NMR spectrum of **3z**

**4a**

**
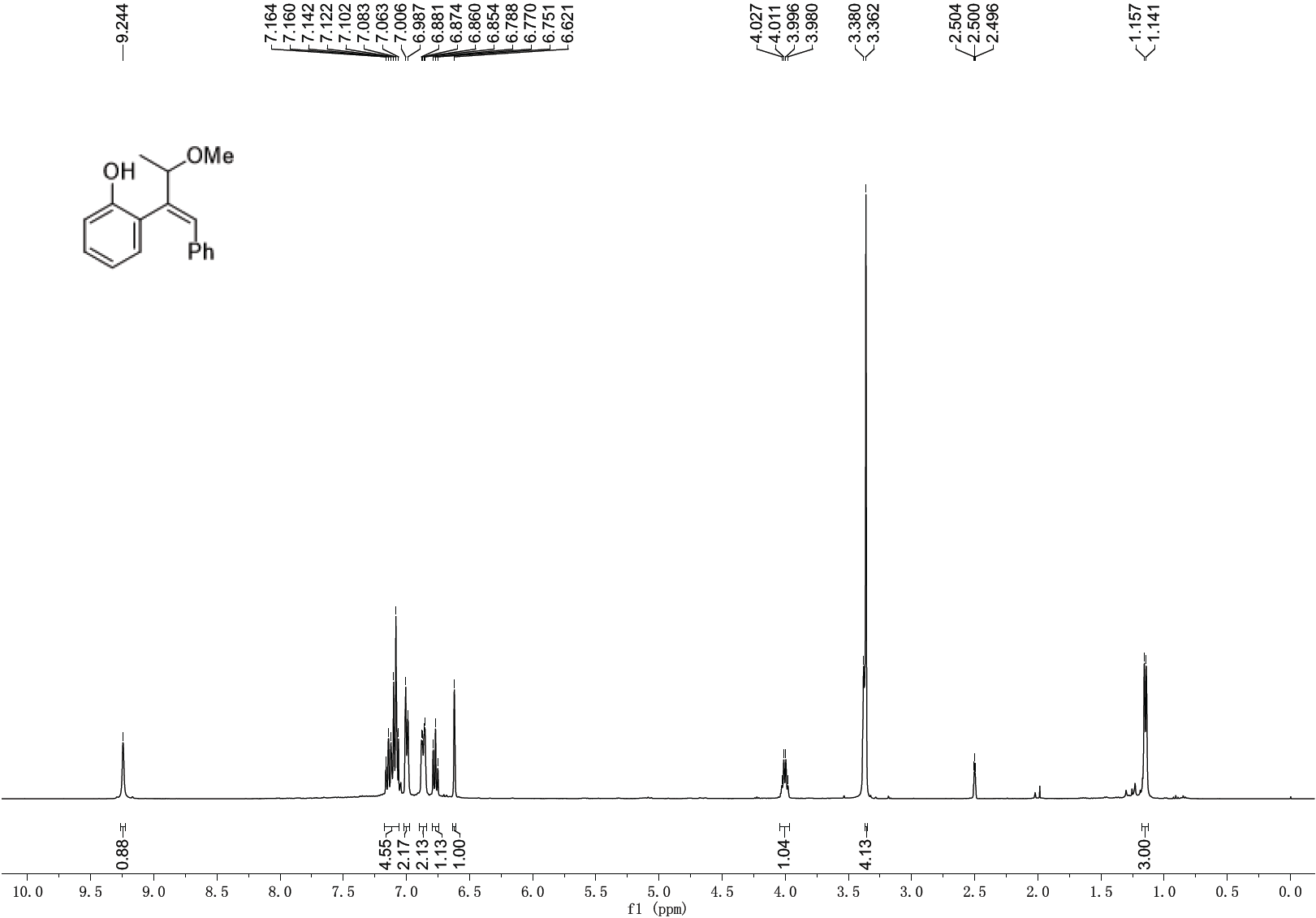
**

**Supplementary Figure 69.** ^1^H-NMR spectrum of **4a**

**
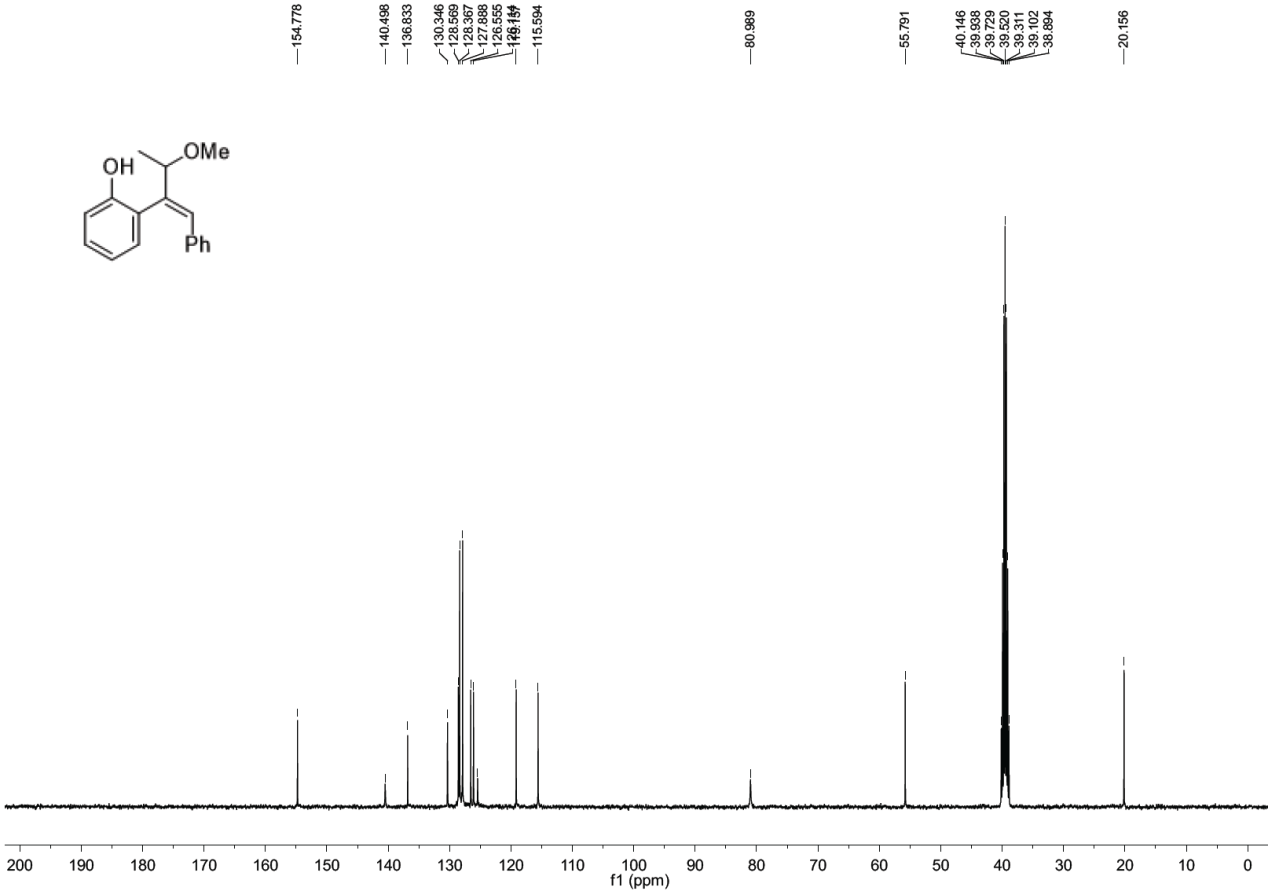
**

**Supplementary Figure 70.** ^13^C-NMR spectrum of **4a**

**4b**

**
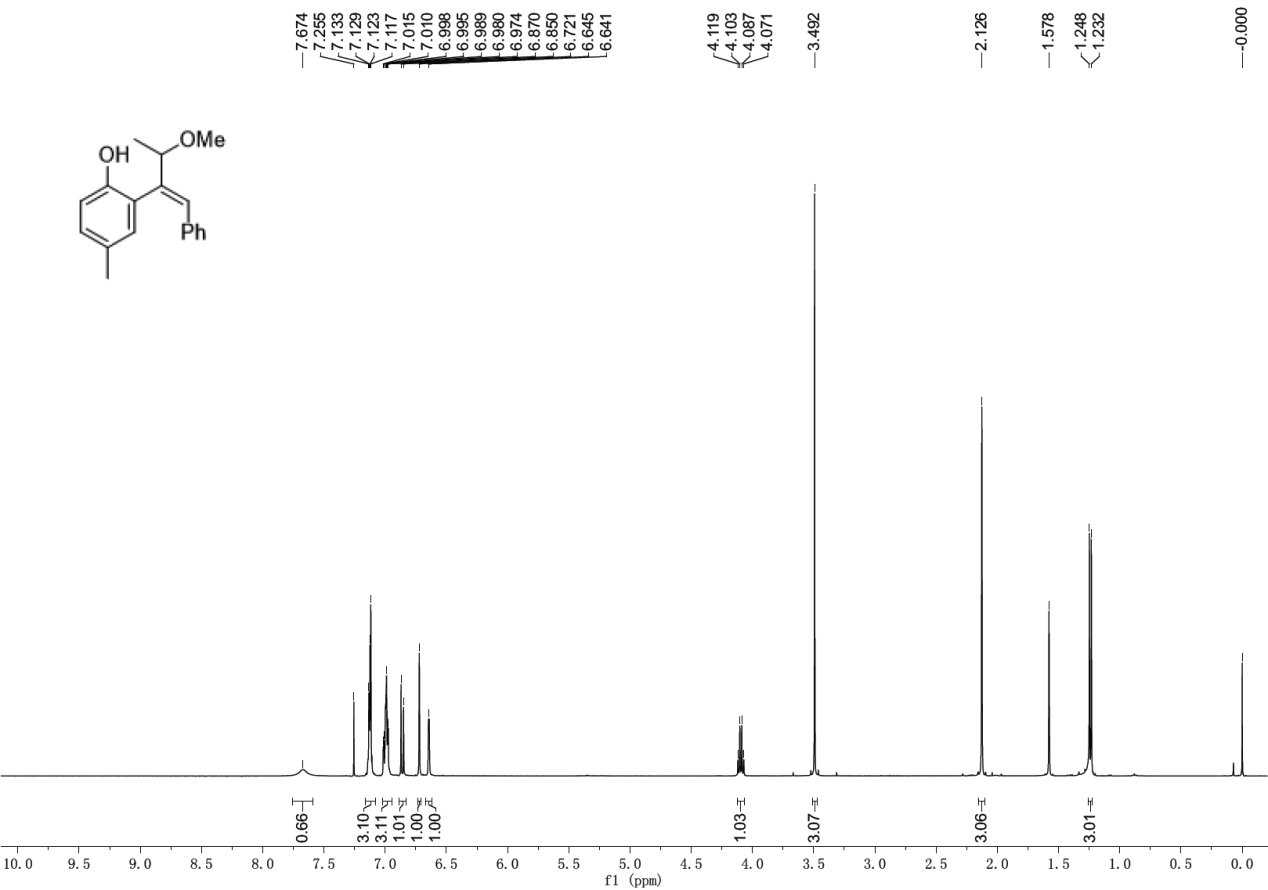
**

**Supplementary Figure 71.** ^1^H-NMR spectrum of **4b**

**
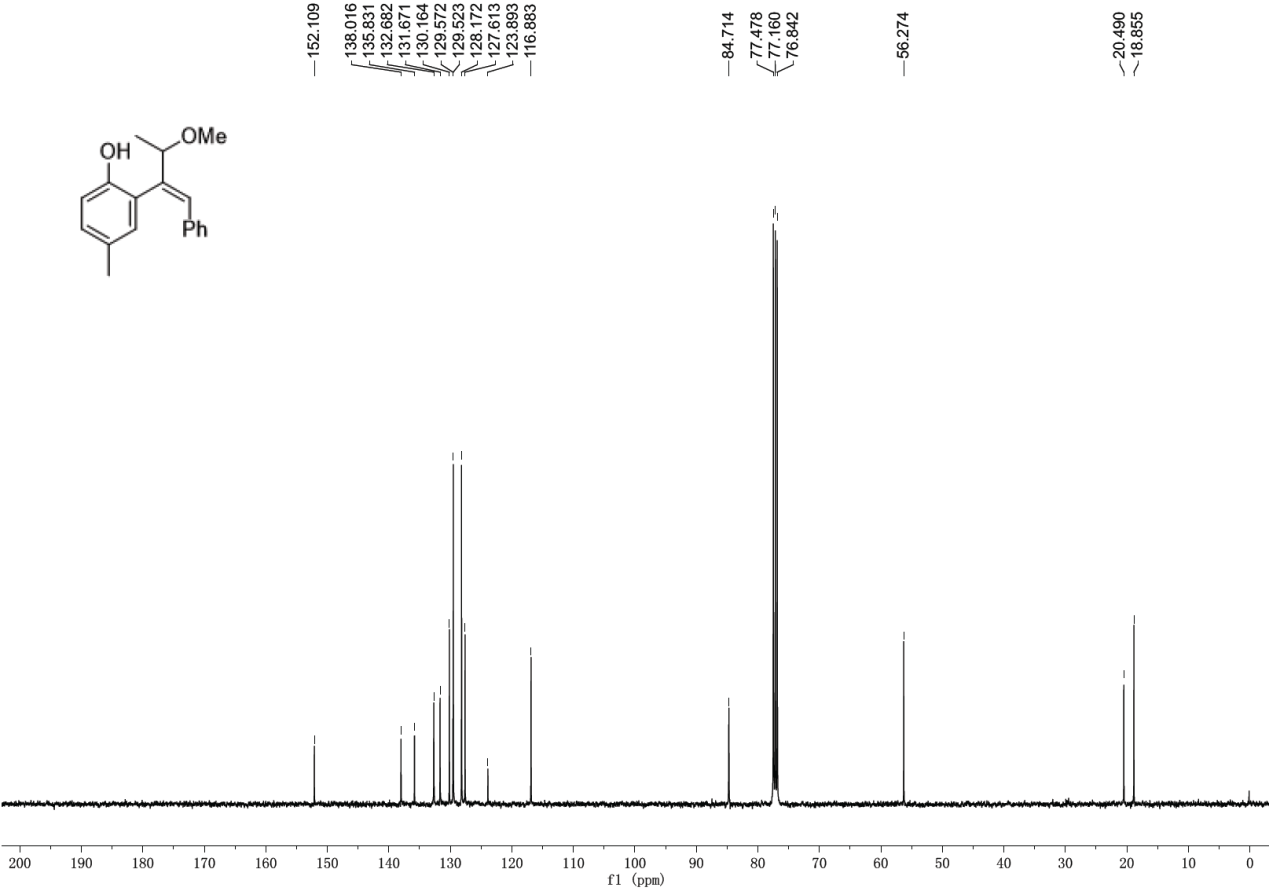
**

**Supplementary Figure 72.** ^13^C-NMR spectrum of **4b**

**4c**

**
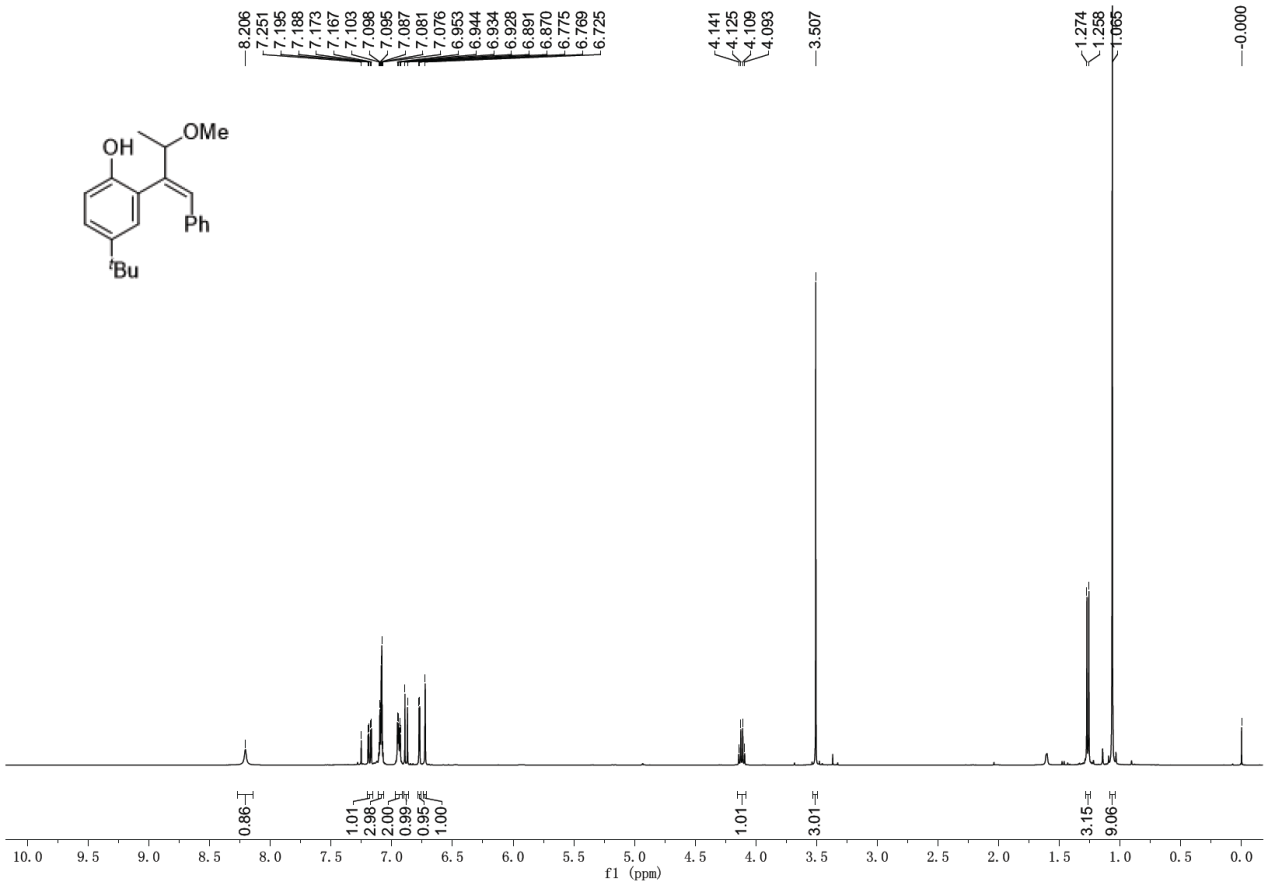
**

**Supplementary Figure 73.** ^1^H-NMR spectrum of **4c**

**
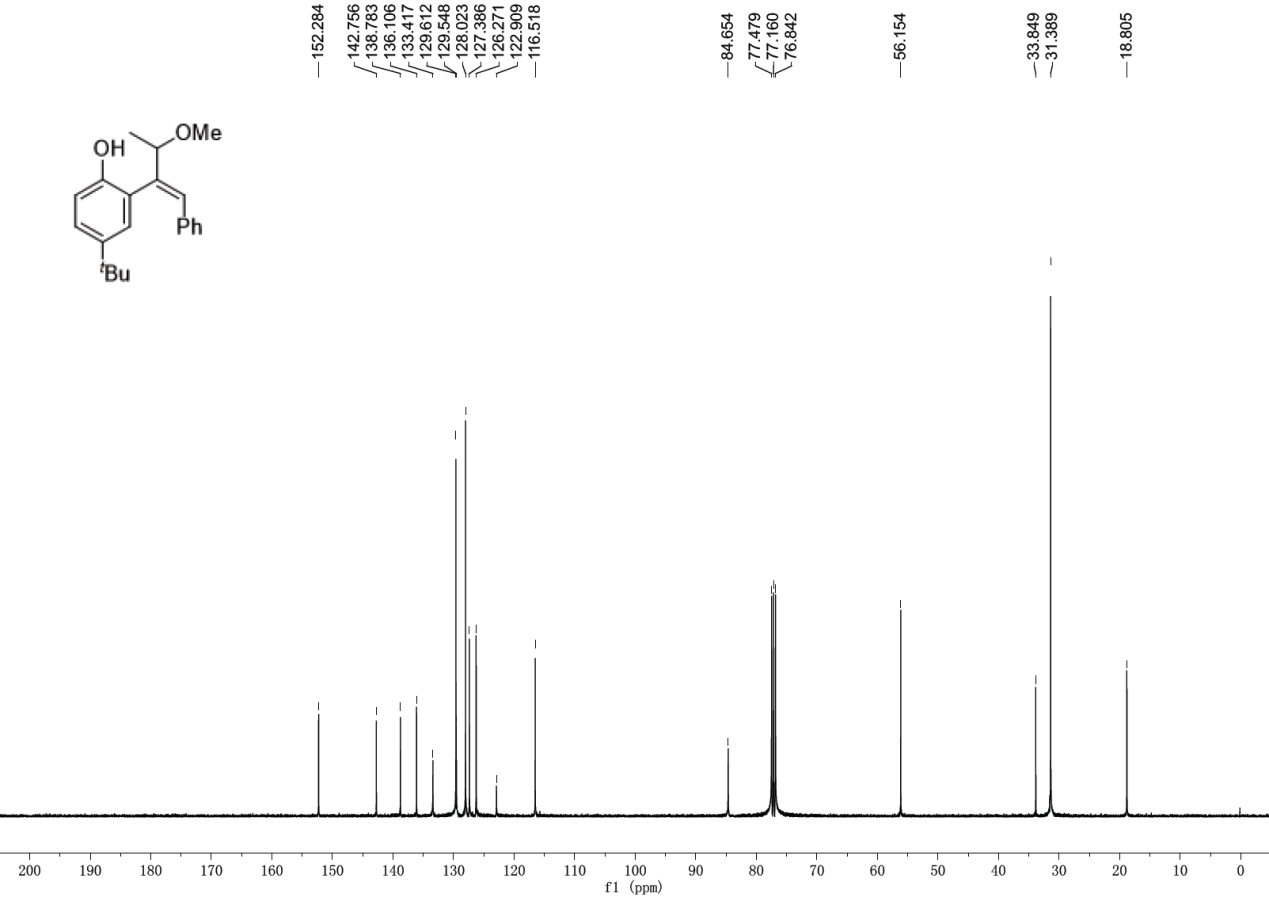
**

**Supplementary Figure 74.** ^13^C-NMR spectrum of **4c**

**4d**

**
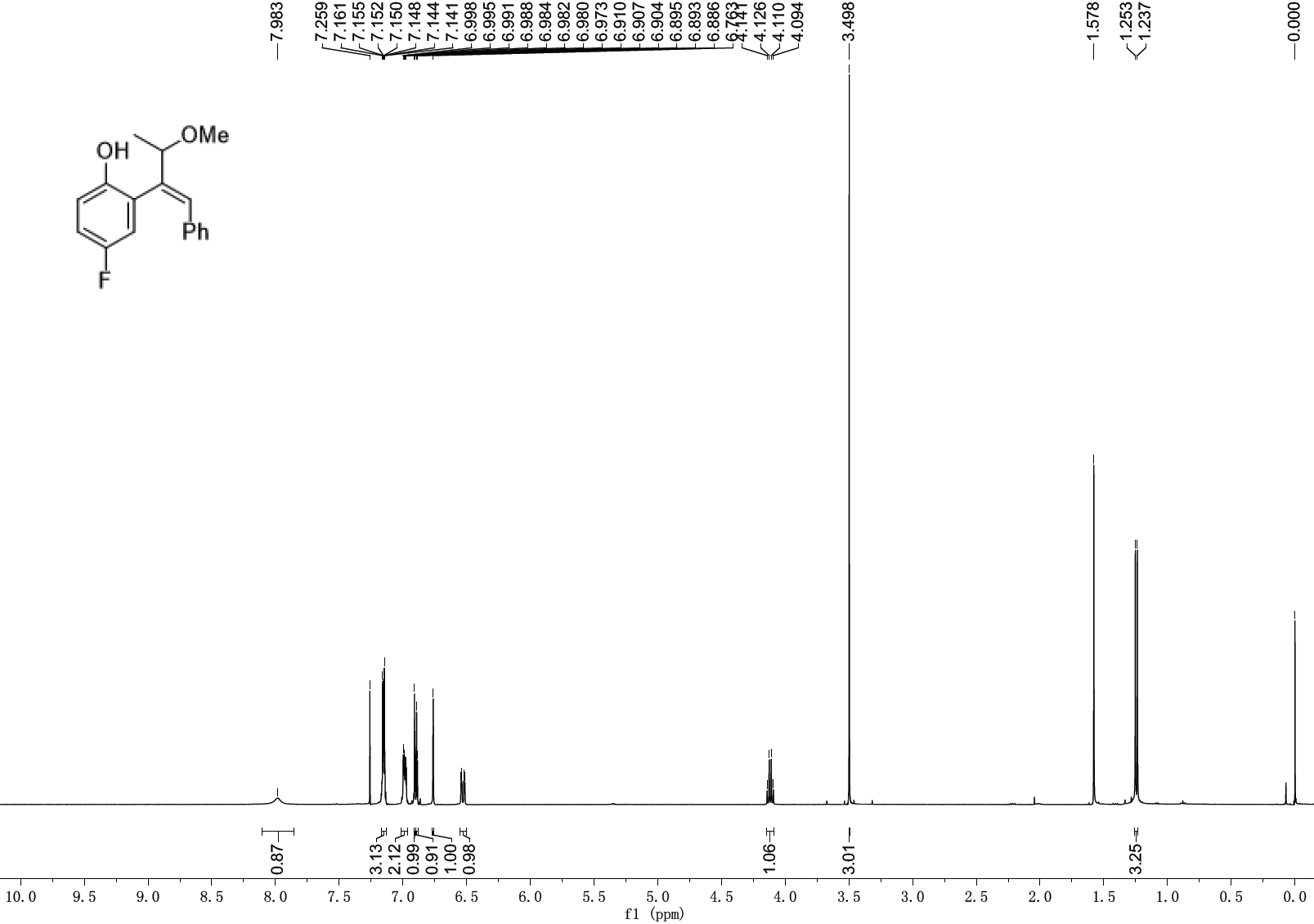
**

**Supplementary Figure 75.** ^1^H-NMR spectrum of **4d**

**
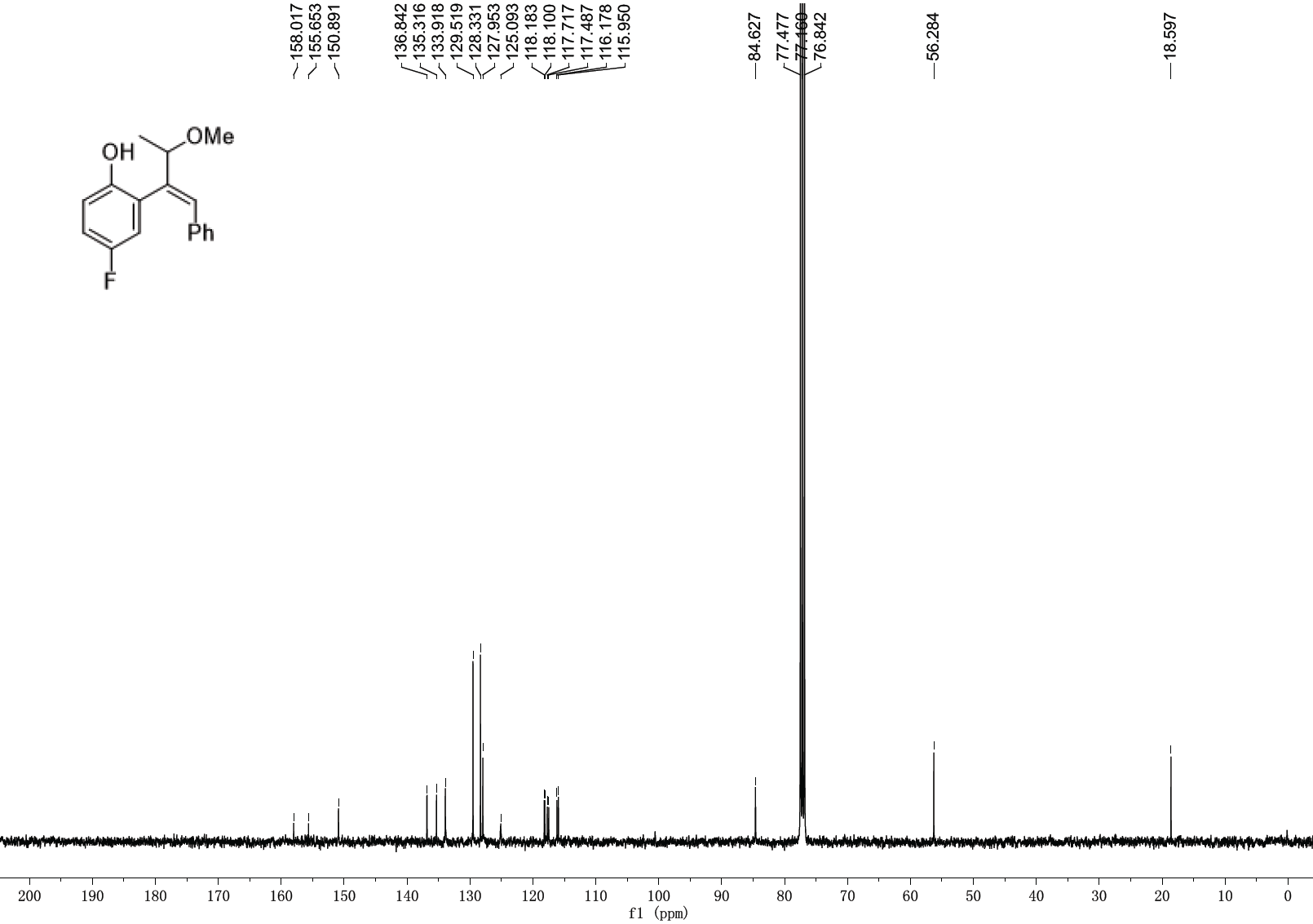
**

**Supplementary Figure 76.** ^13^C-NMR spectrum of **4d**

**
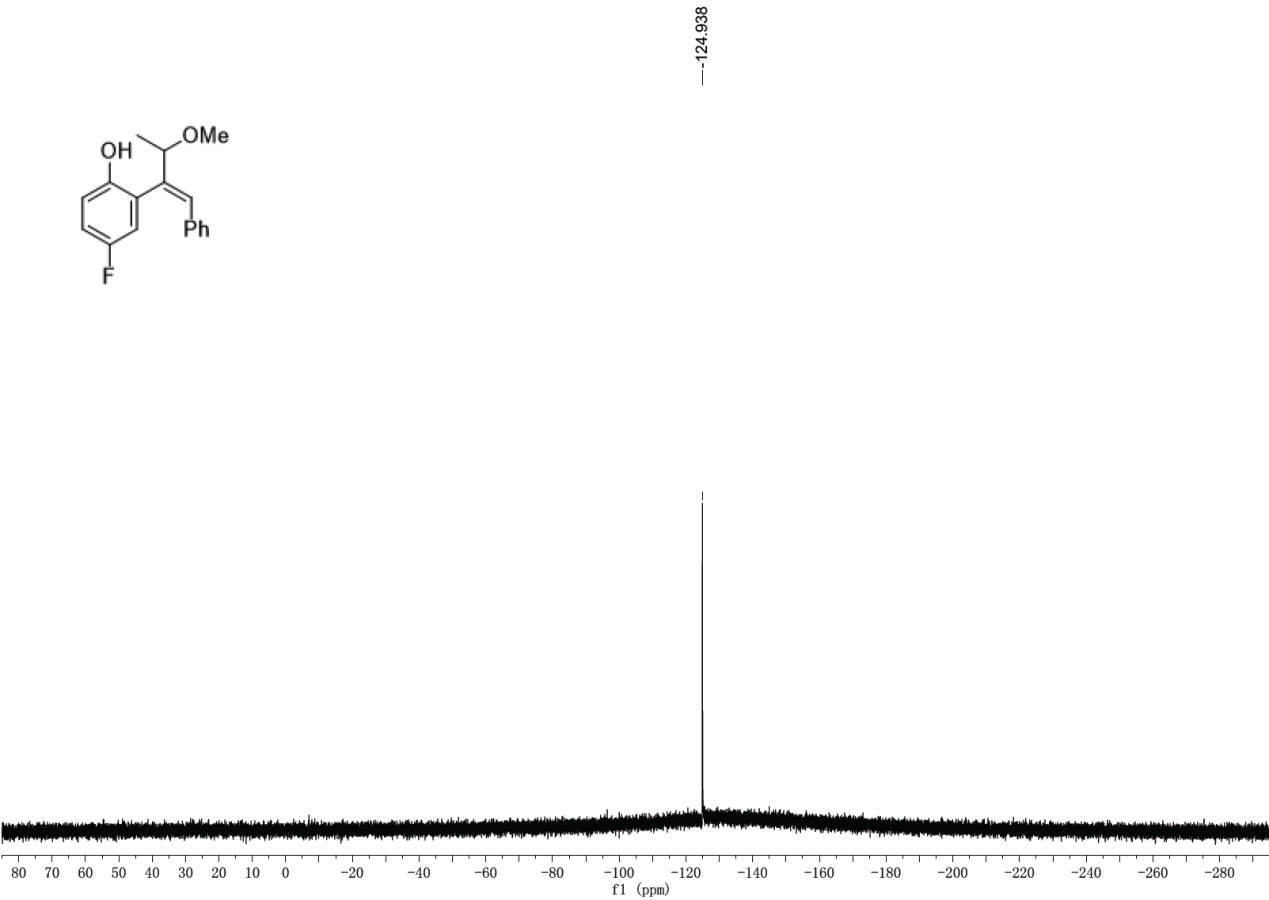
**

**Supplementary Figure 77.** ^19^F-NMR spectrum of **4d**

**4e**

**
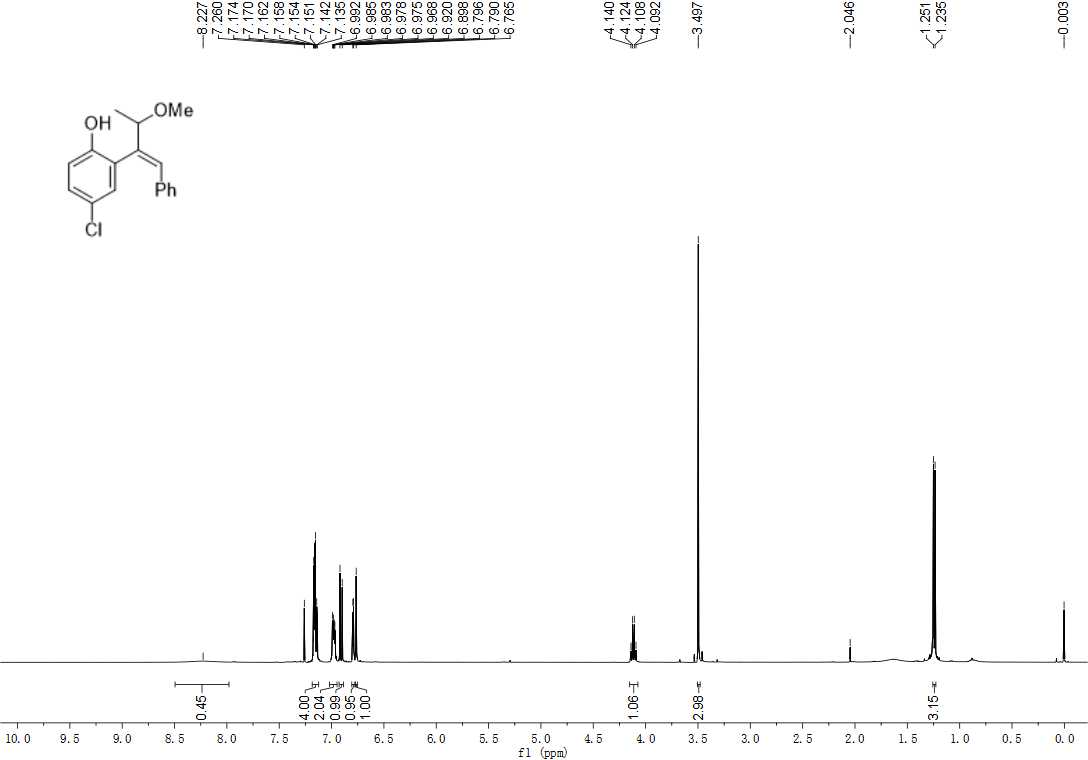
**

**Supplementary Figure 78.** ^1^H-NMR spectrum of **4e**

**
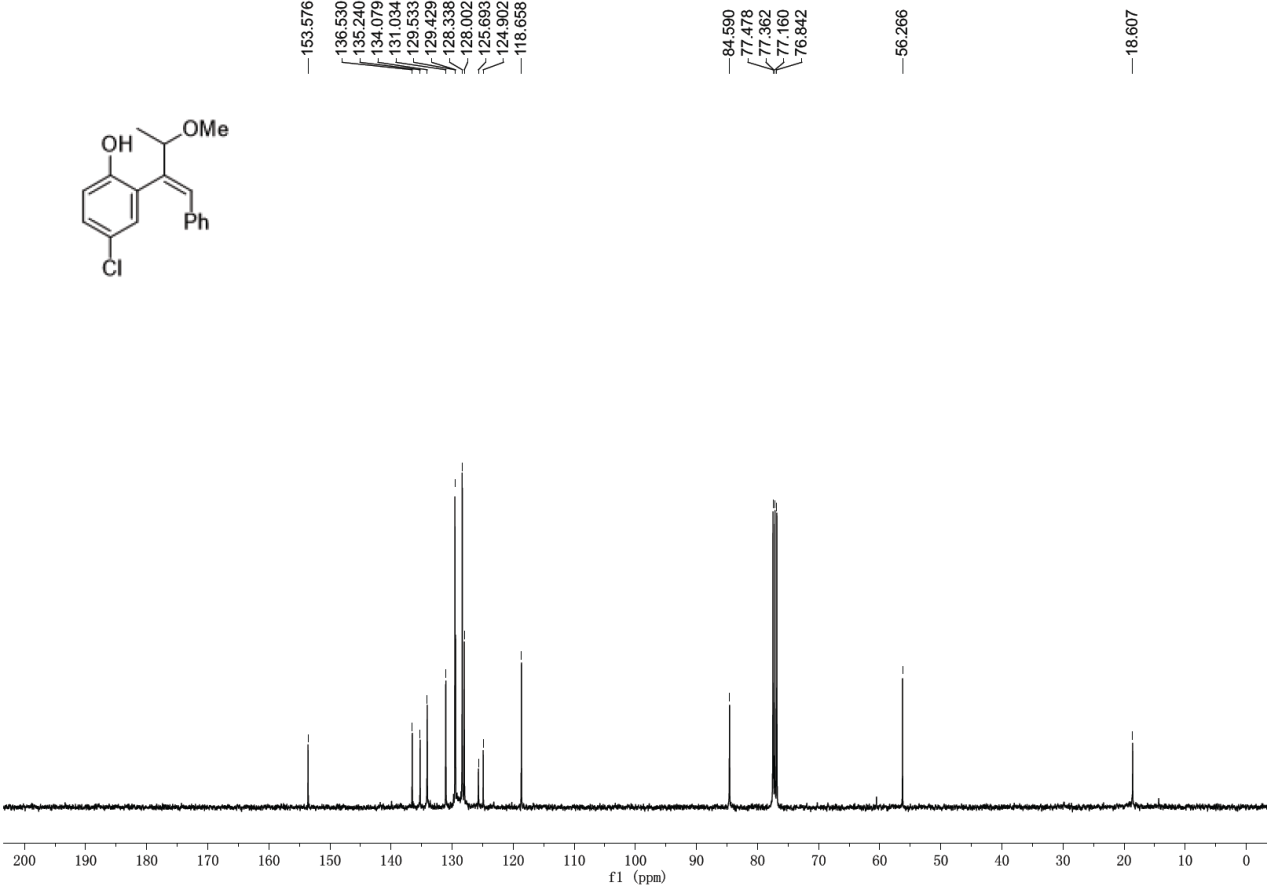
**

**Supplementary Figure 78.** ^13^C-NMR spectrum of **4e**

**4f**

**
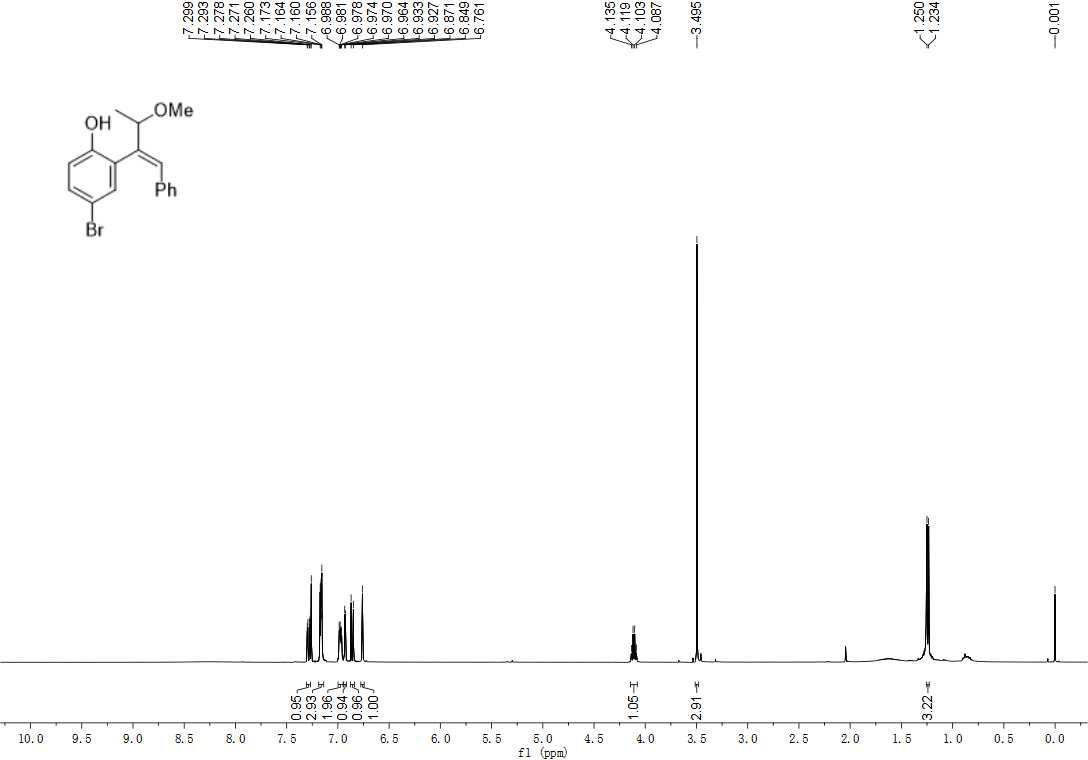
**

**Supplementary Figure 79.** ^1^H-NMR spectrum of **4f**

**
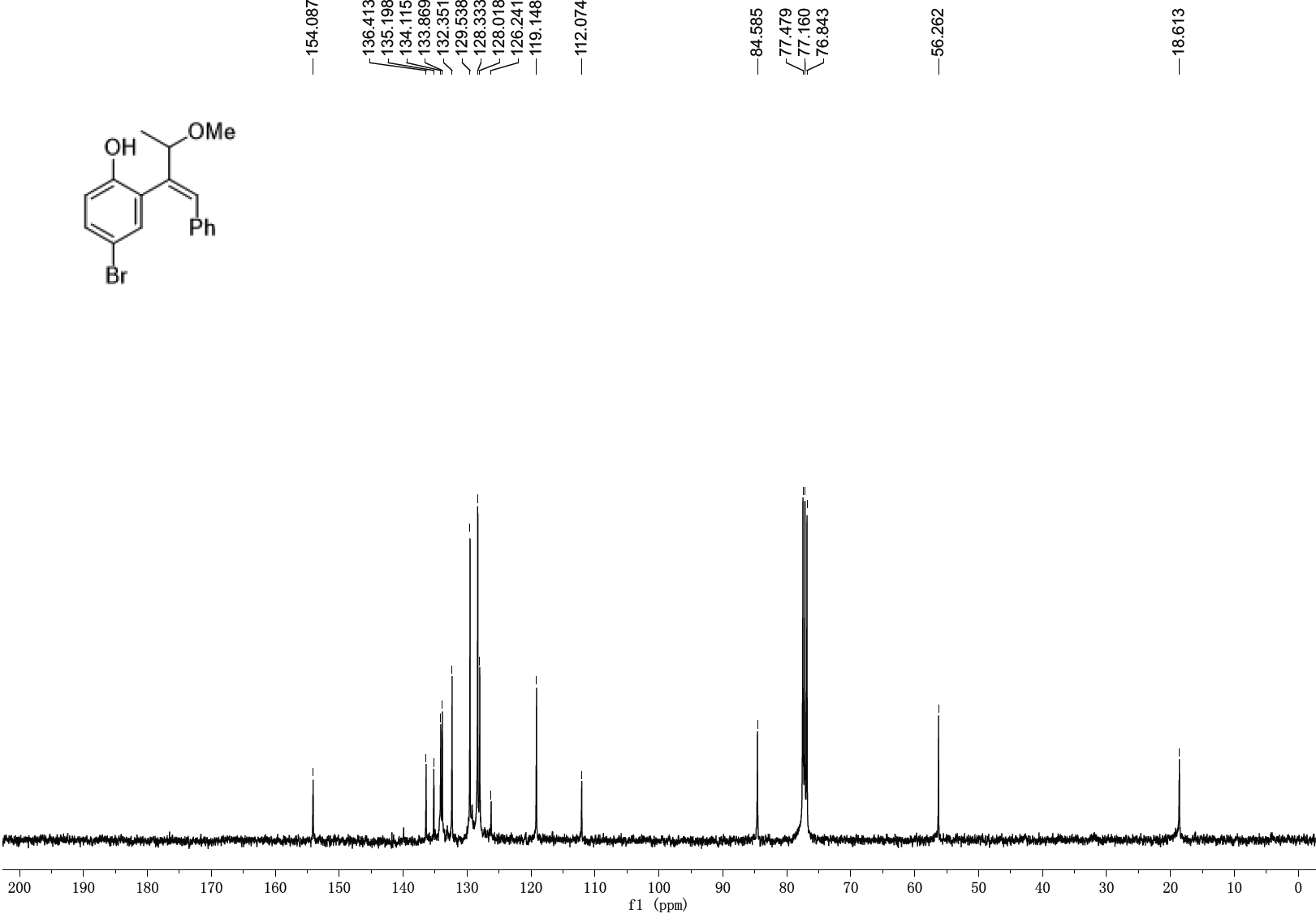
**

**Supplementary Figure 80.** ^13^C-NMR spectrum of **4f**

**4g**

**
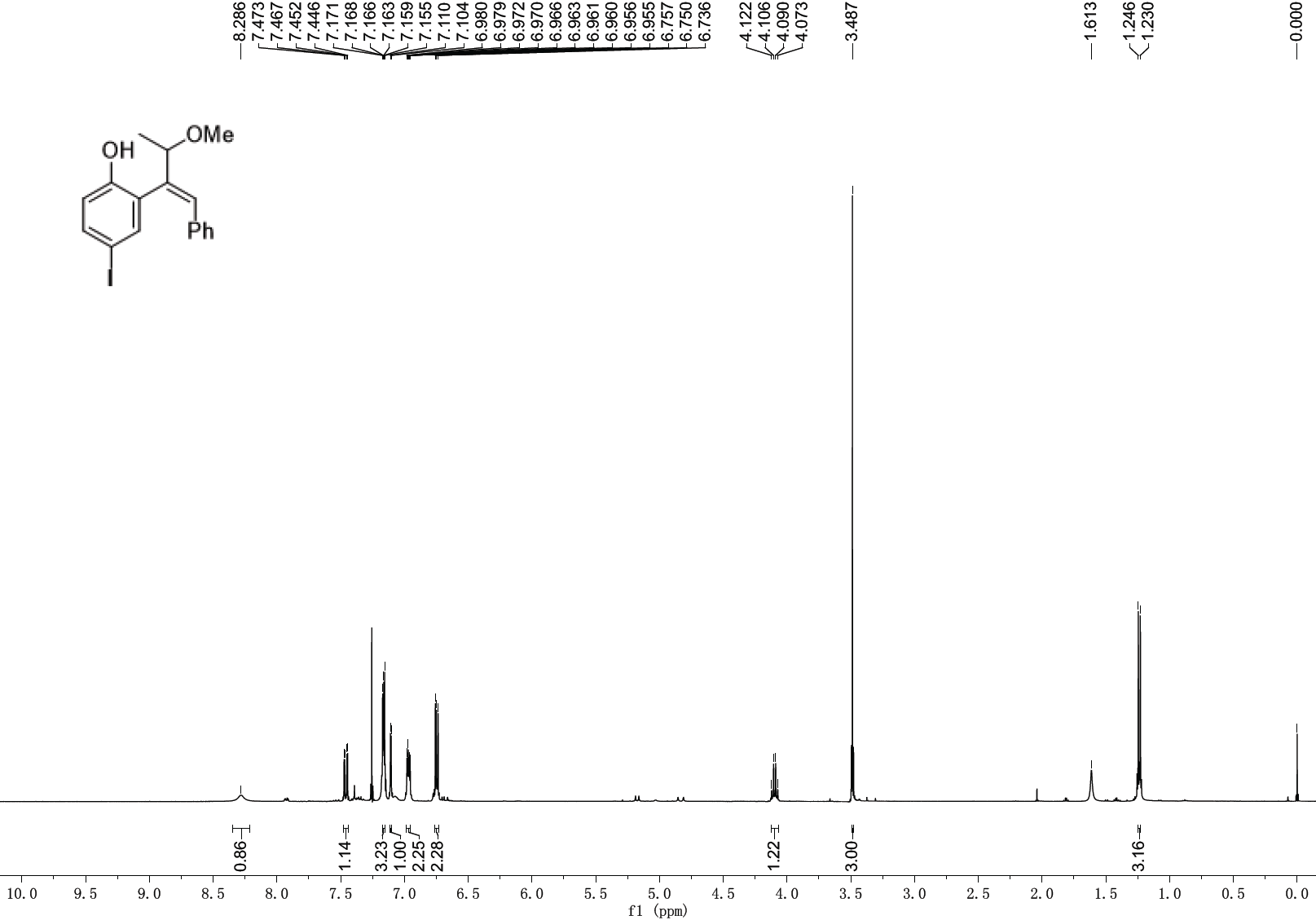
**

**Supplementary Figure 81.** ^1^H-NMR spectrum of **4g**

**
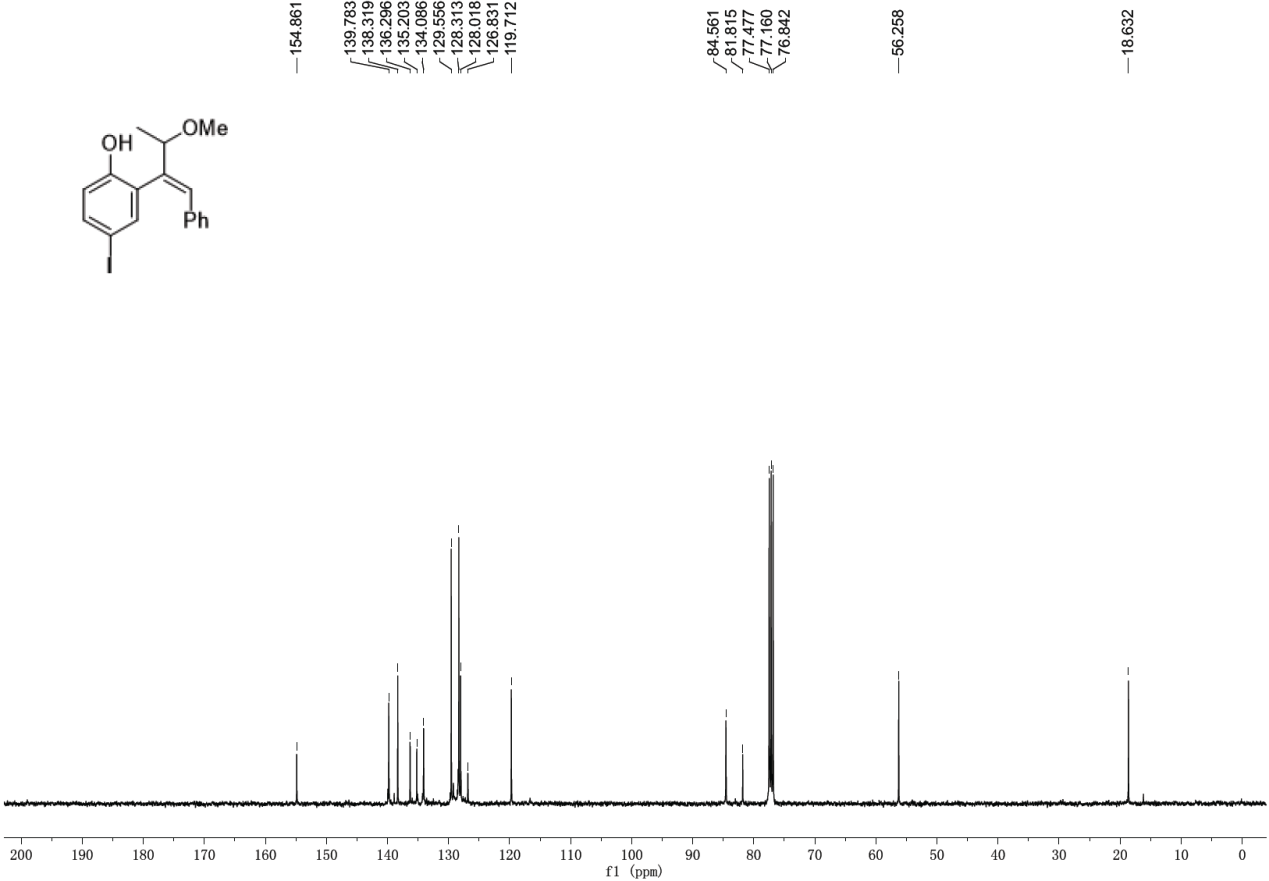
**

**Supplementary Figure 82.** ^13^C-NMR spectrum of **4g**

**4h**

**
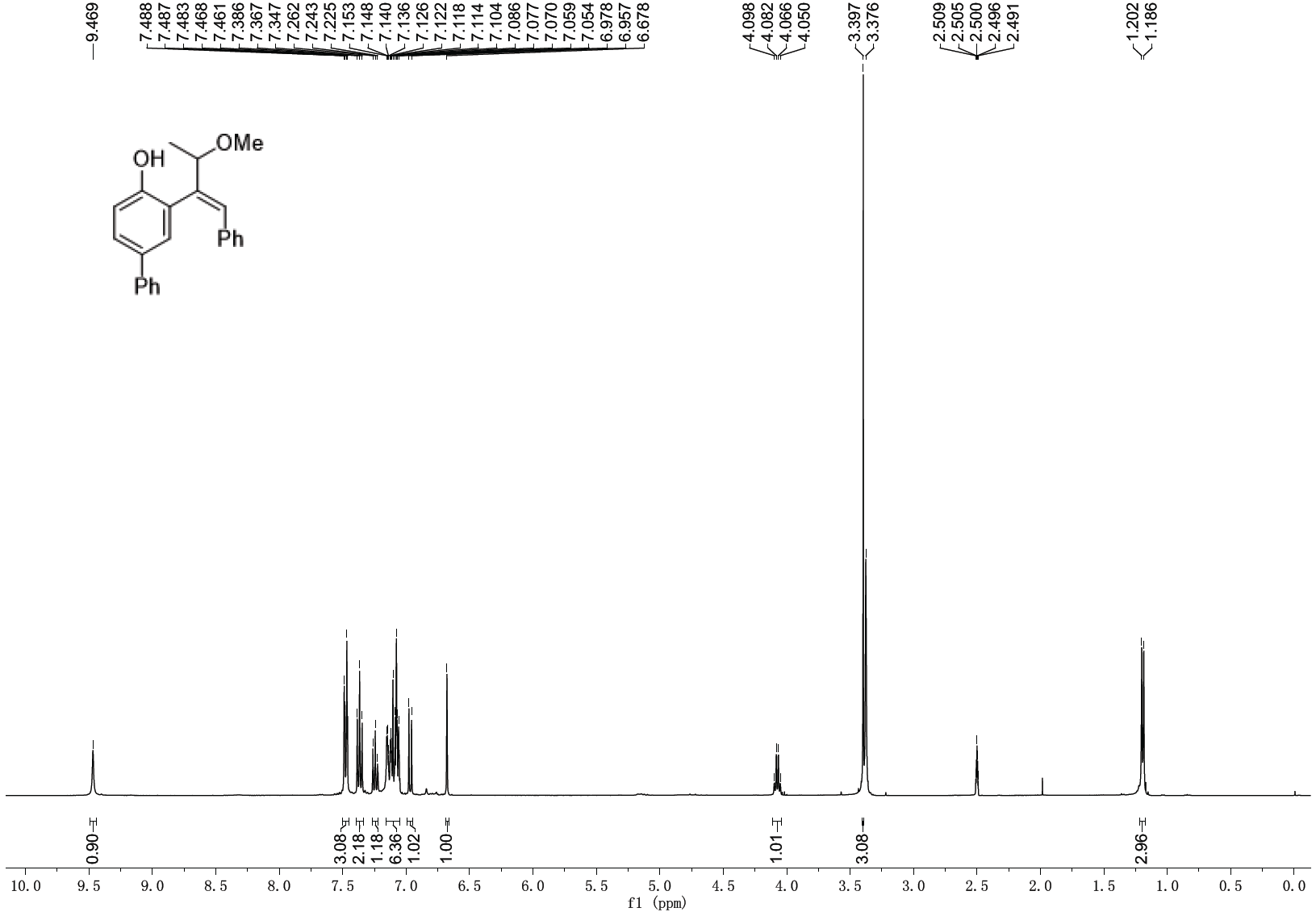
**

**Supplementary Figure 83.** ^1^H-NMR spectrum of **4h**

**
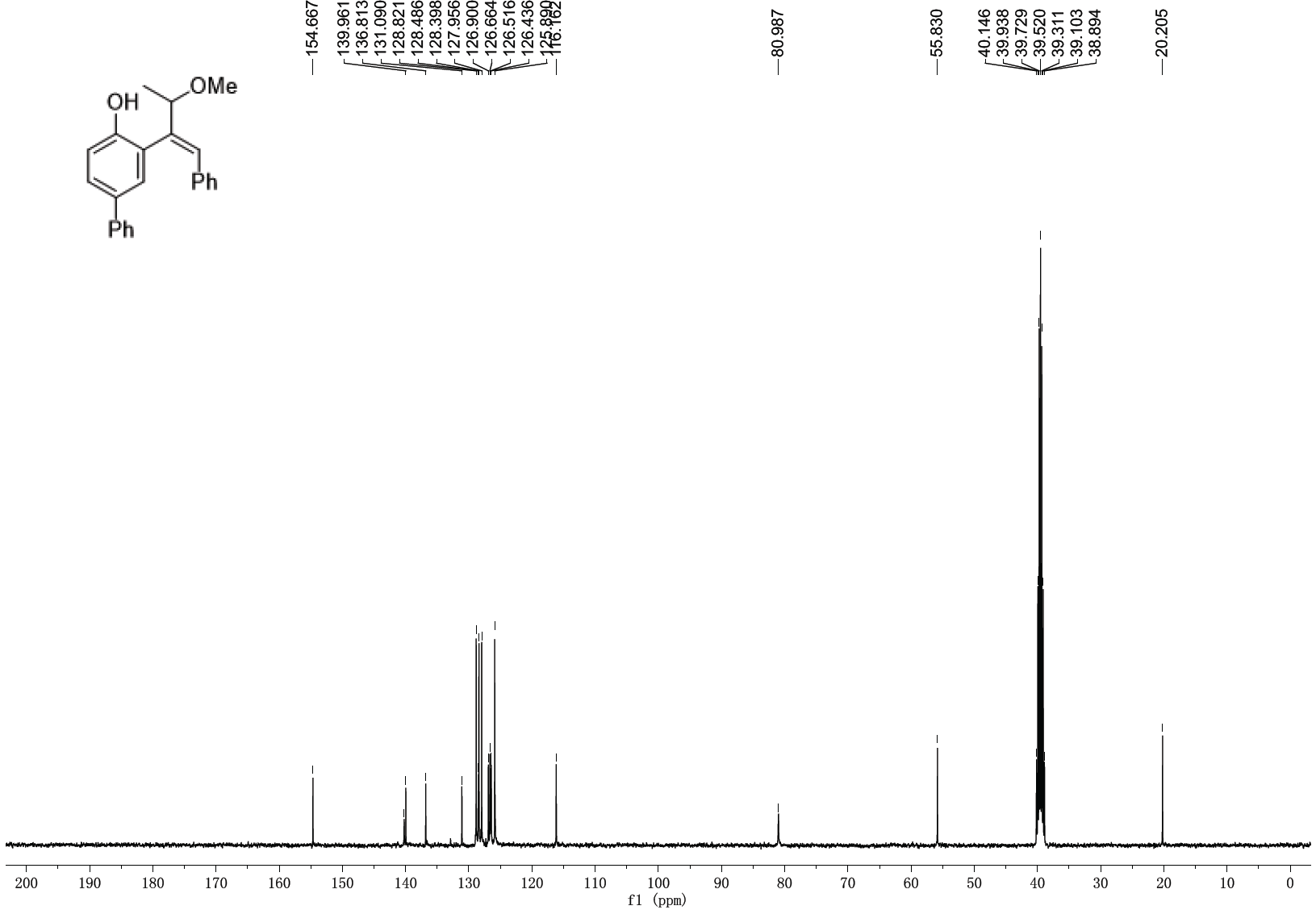
**

**Supplementary Figure 84.** ^13^C-NMR spectrum of **4h**

**4i**

**
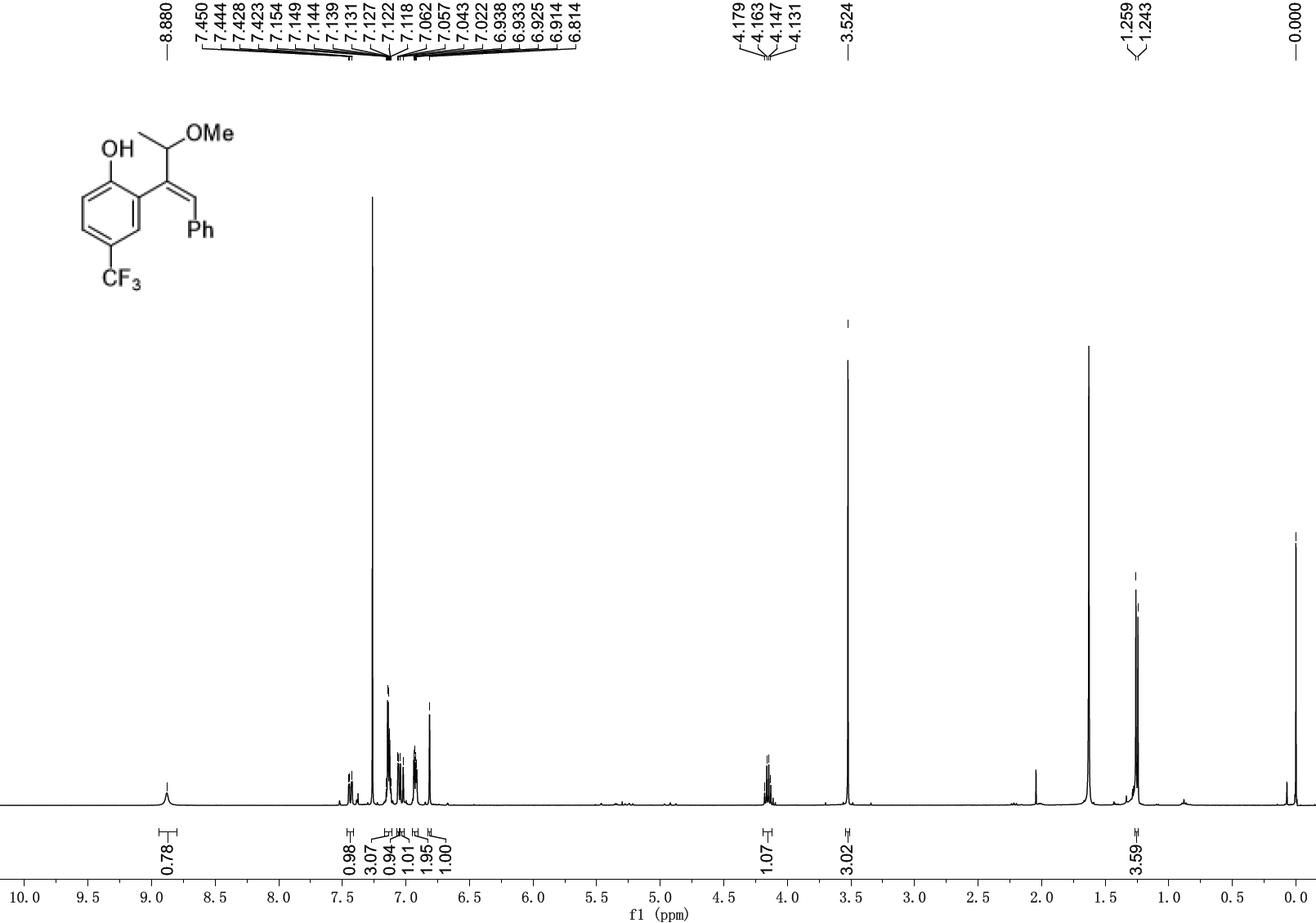
**

**Supplementary Figure 85.** ^1^H-NMR spectrum of **4i**

**
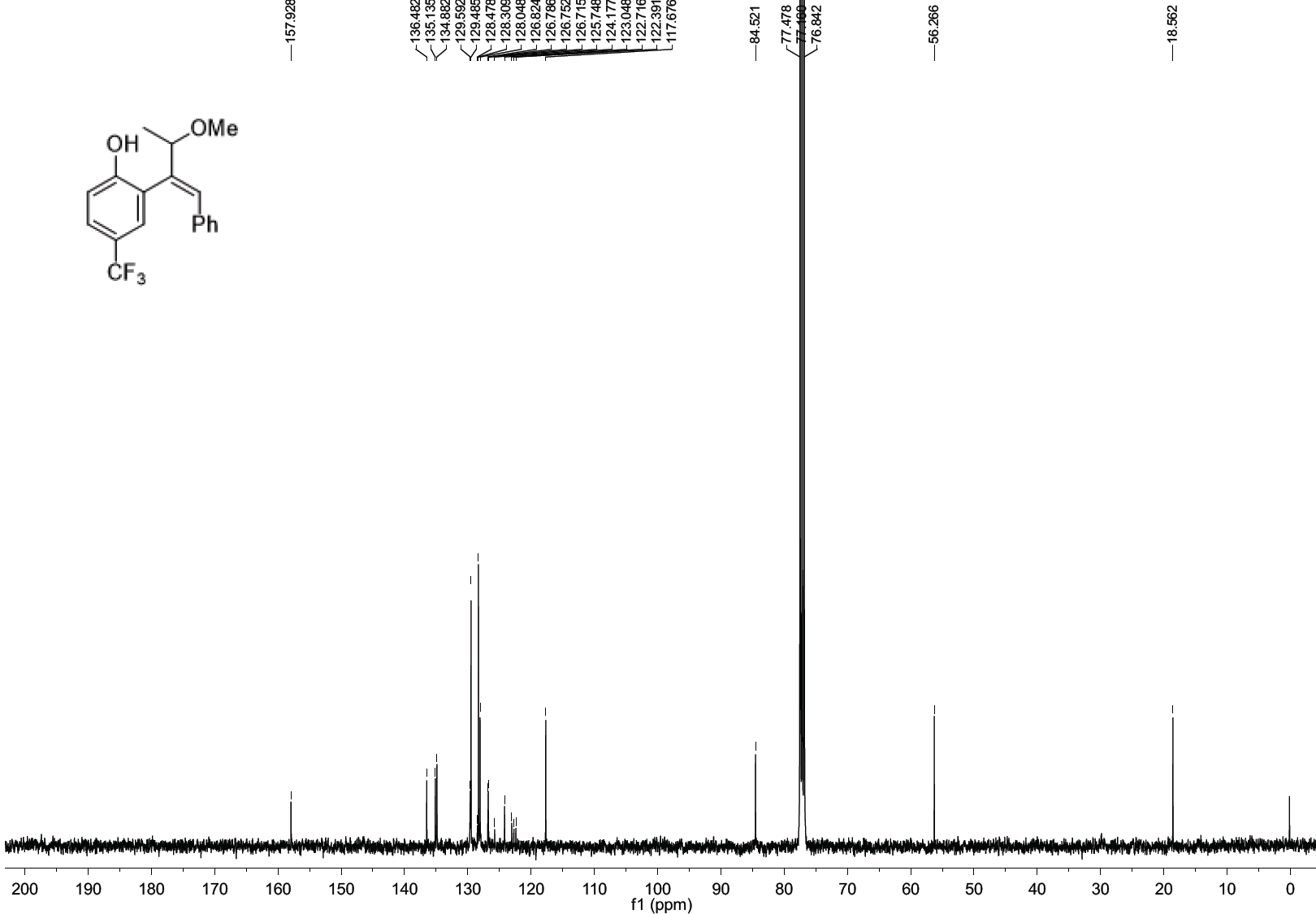
**

**Supplementary Figure 86.** ^13^C-NMR spectrum of **4i**

**
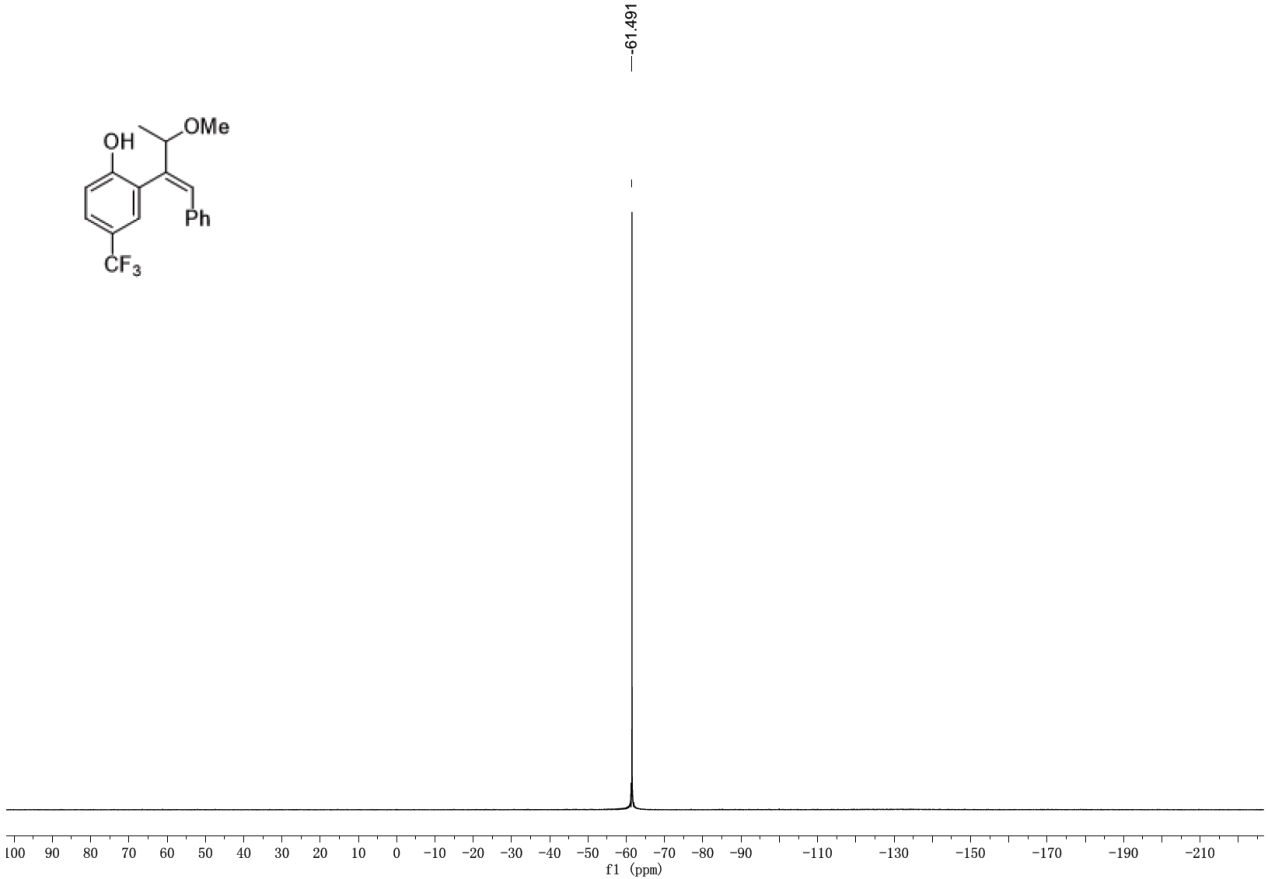
**

**Supplementary Figure 87.** ^19^F-NMR spectrum of **4i**

**4j**

**
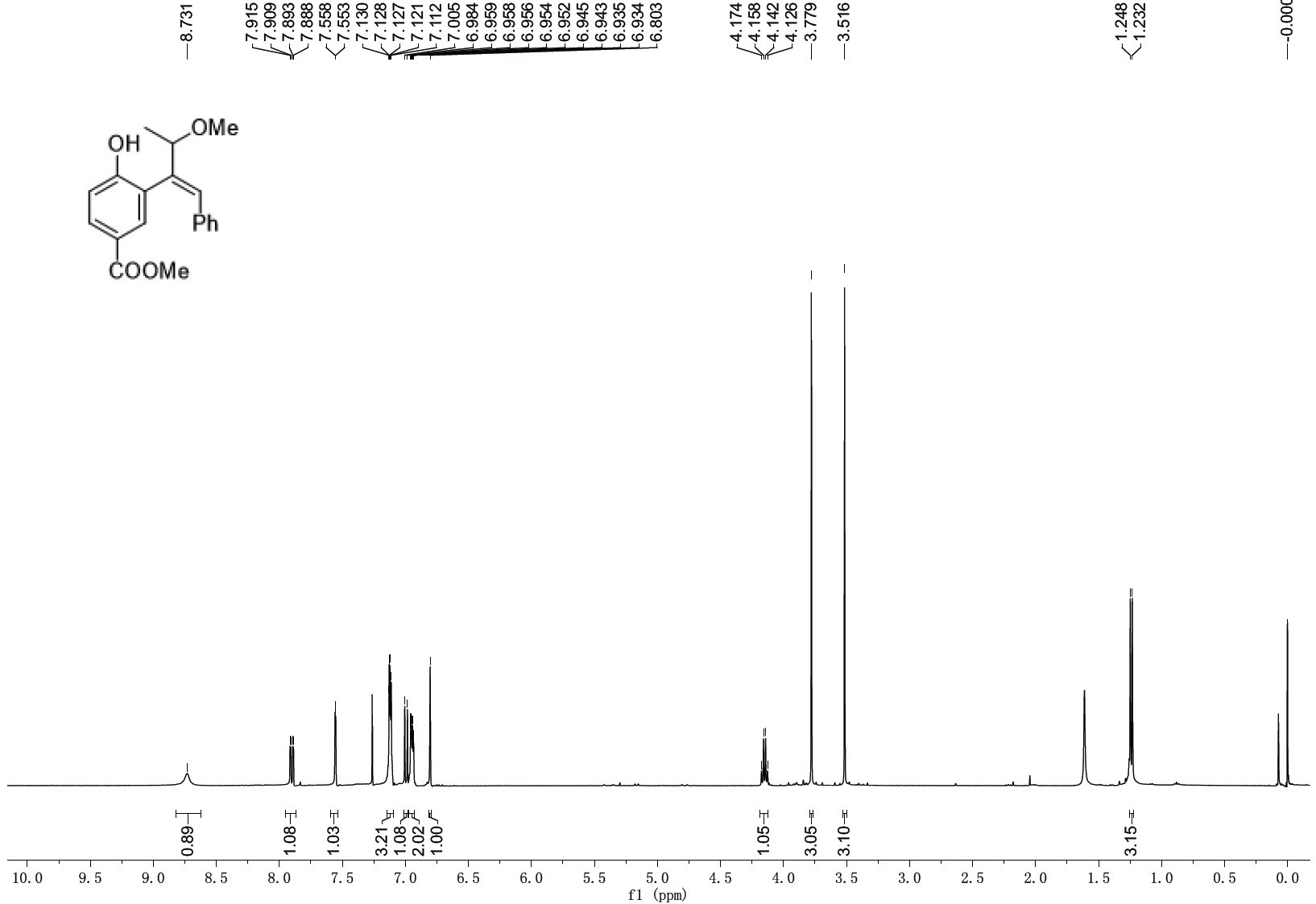
**

**Supplementary Figure 88.** ^1^H-NMR spectrum of **4j**

**
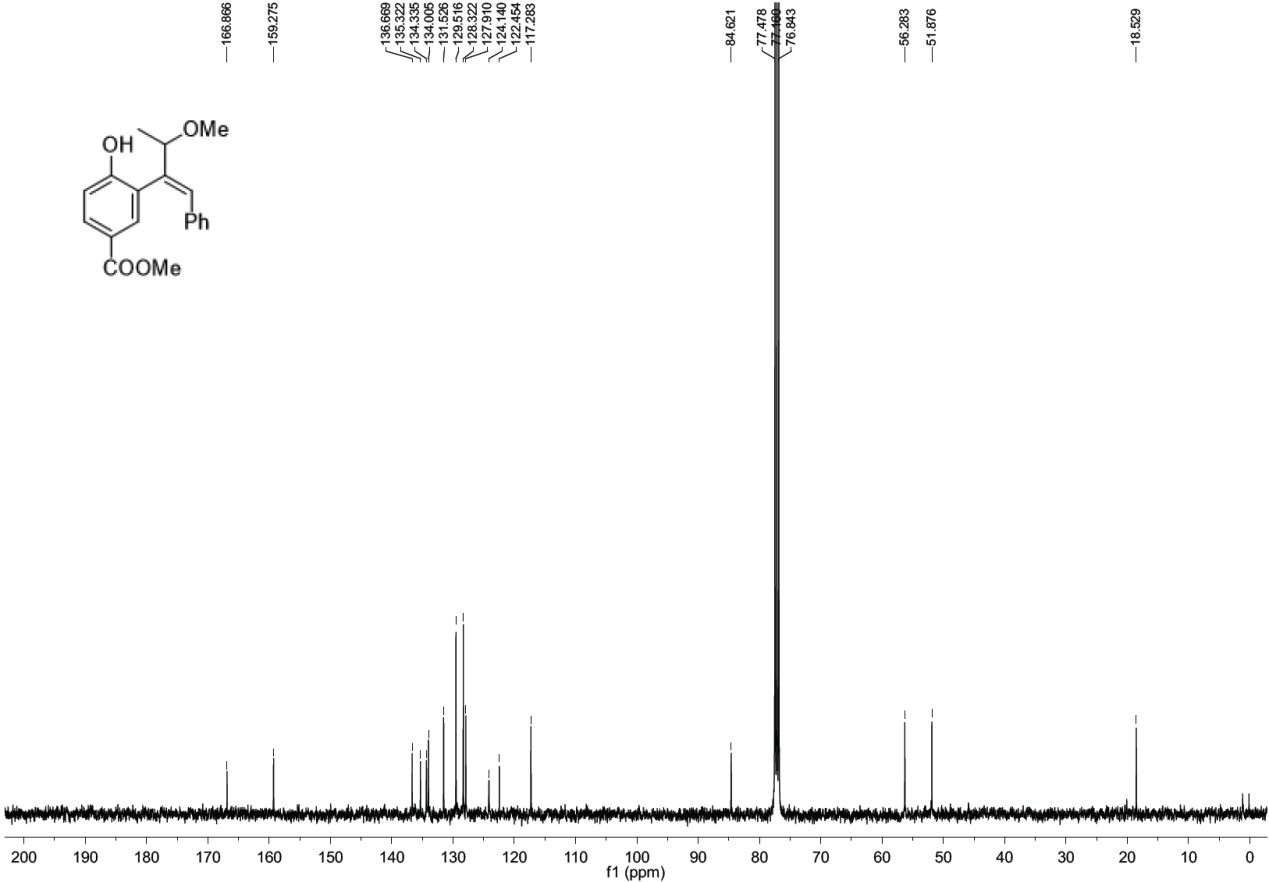
**

**Supplementary Figure 89.** ^13^C-NMR spectrum of **4j**

**4k**

**
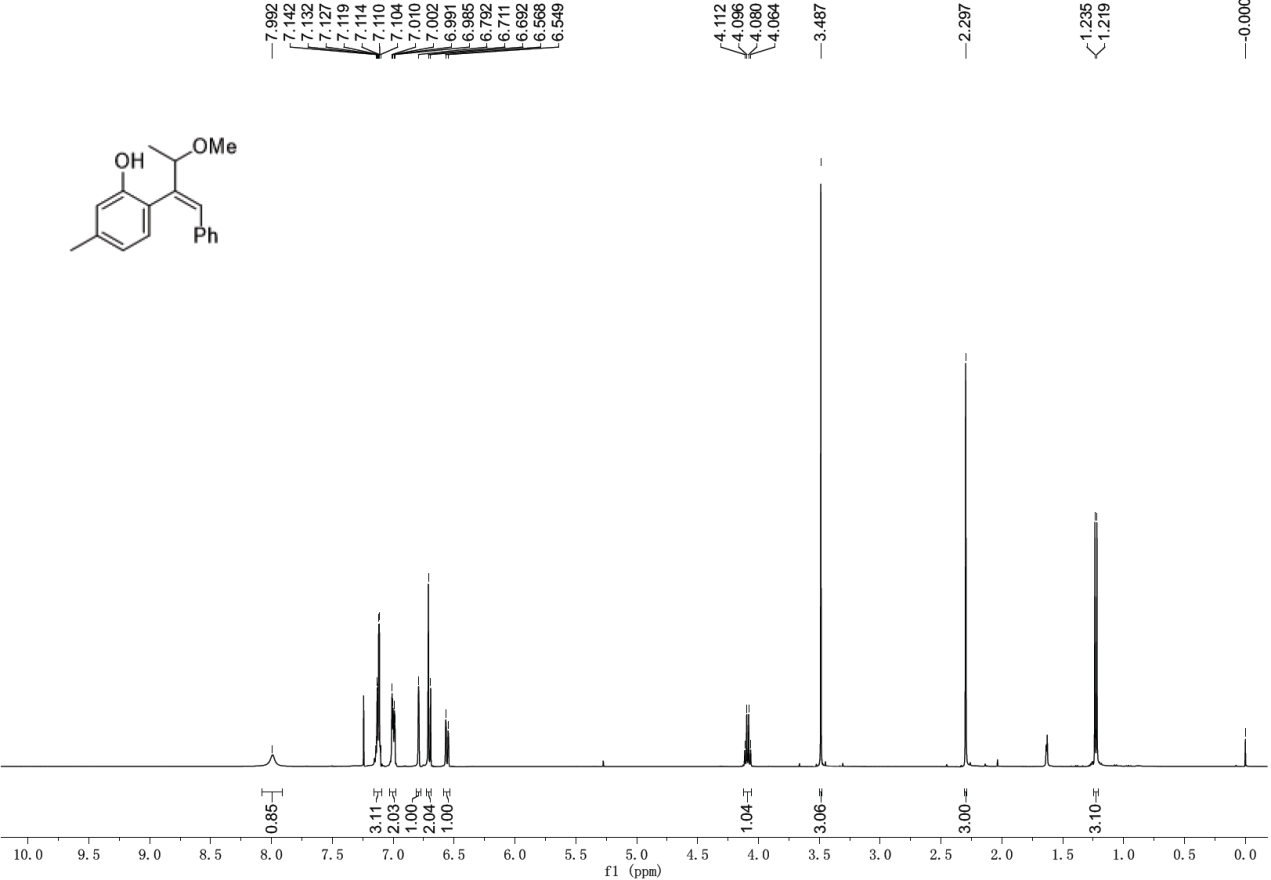
**

**Supplementary Figure 90.** ^1^H-NMR spectrum of **4k**

**
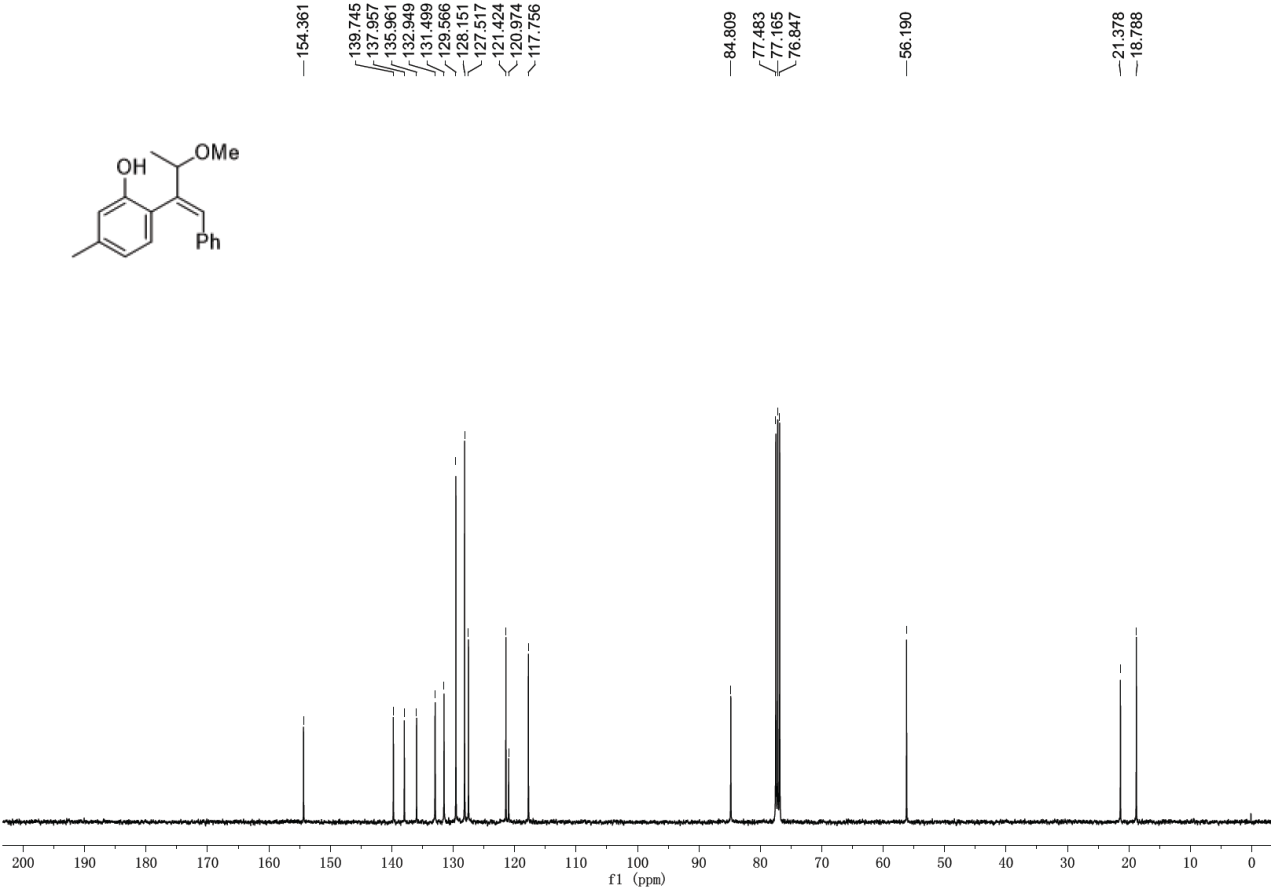
**

**Supplementary Figure 91.** ^13^C-NMR spectrum of **4k**

**4l**

**
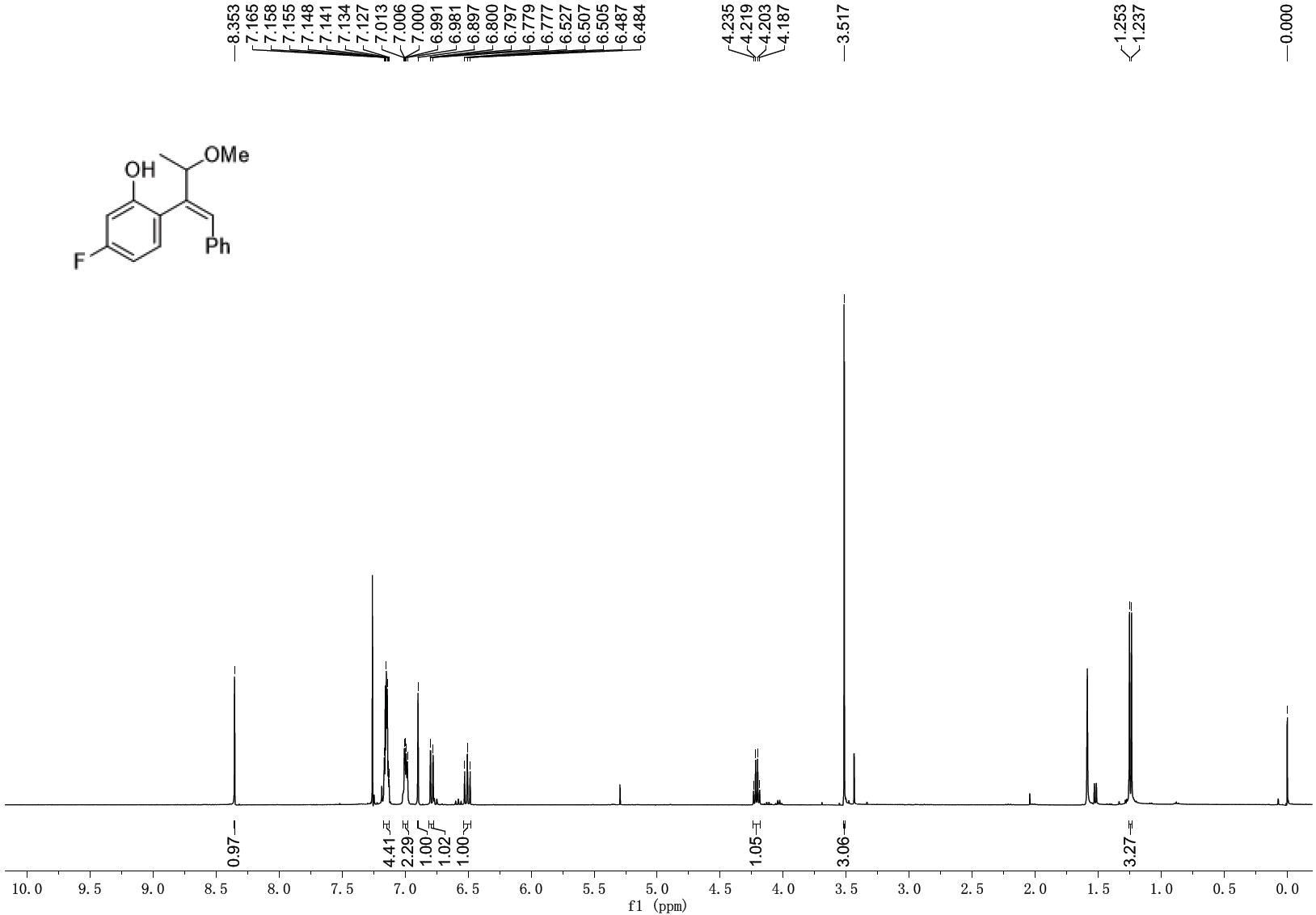
**

**Supplementary Figure 92.** ^1^H-NMR spectrum of **4l**

**
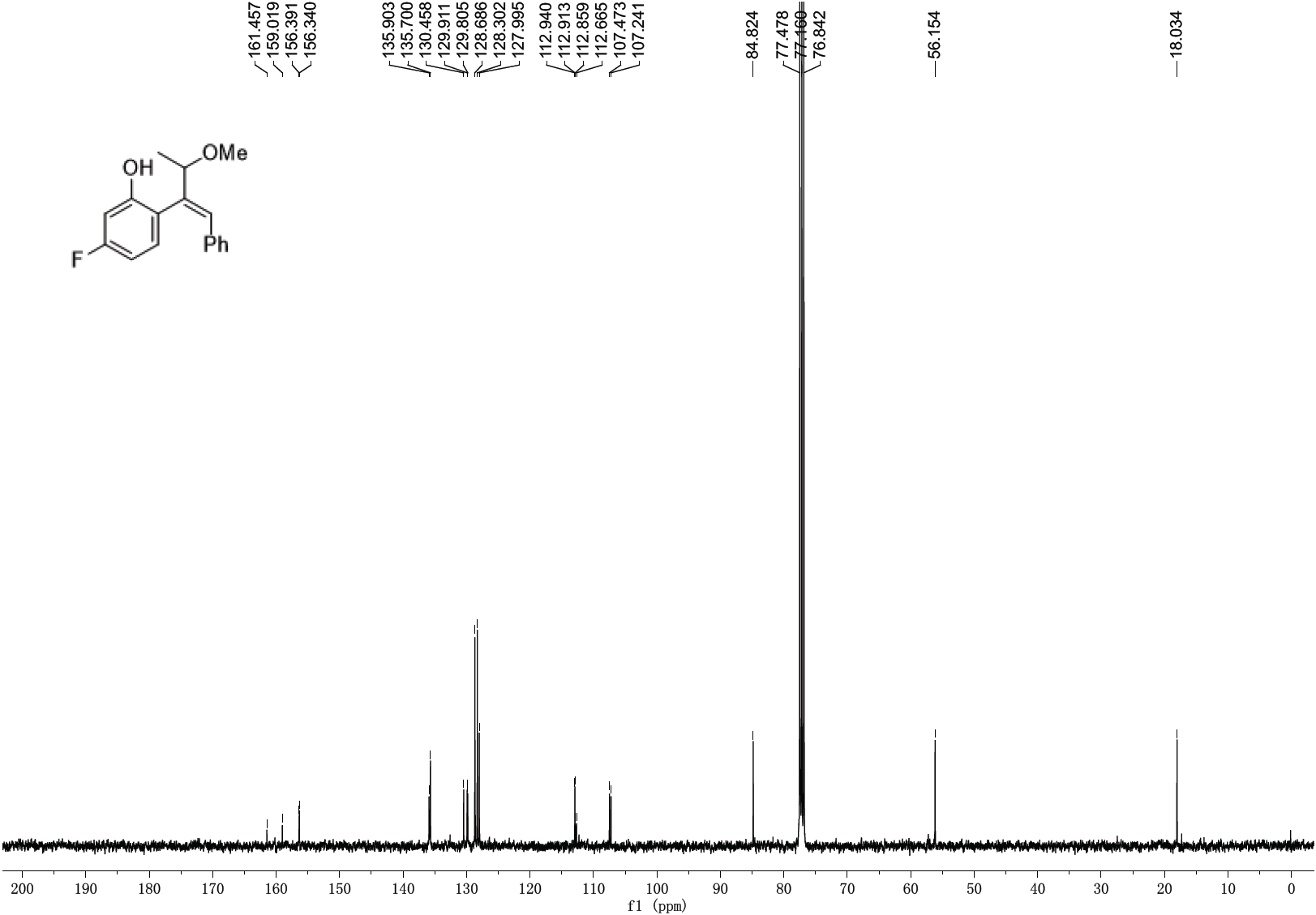
**

**Supplementary Figure 93.** ^13^C-NMR spectrum of **4l**

**
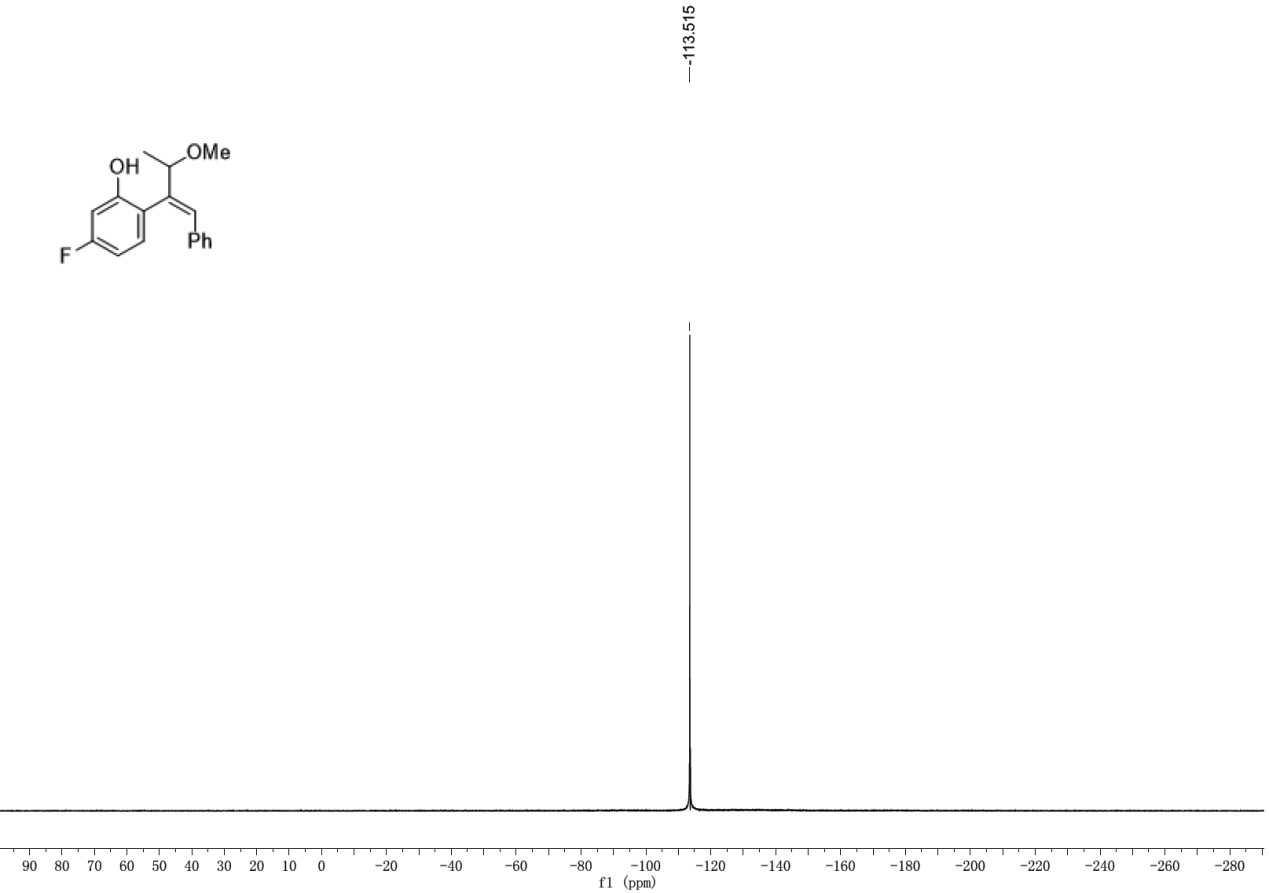
**

**Supplementary Figure 94.** ^19^F-NMR spectrum of **4l**

**4m**

**
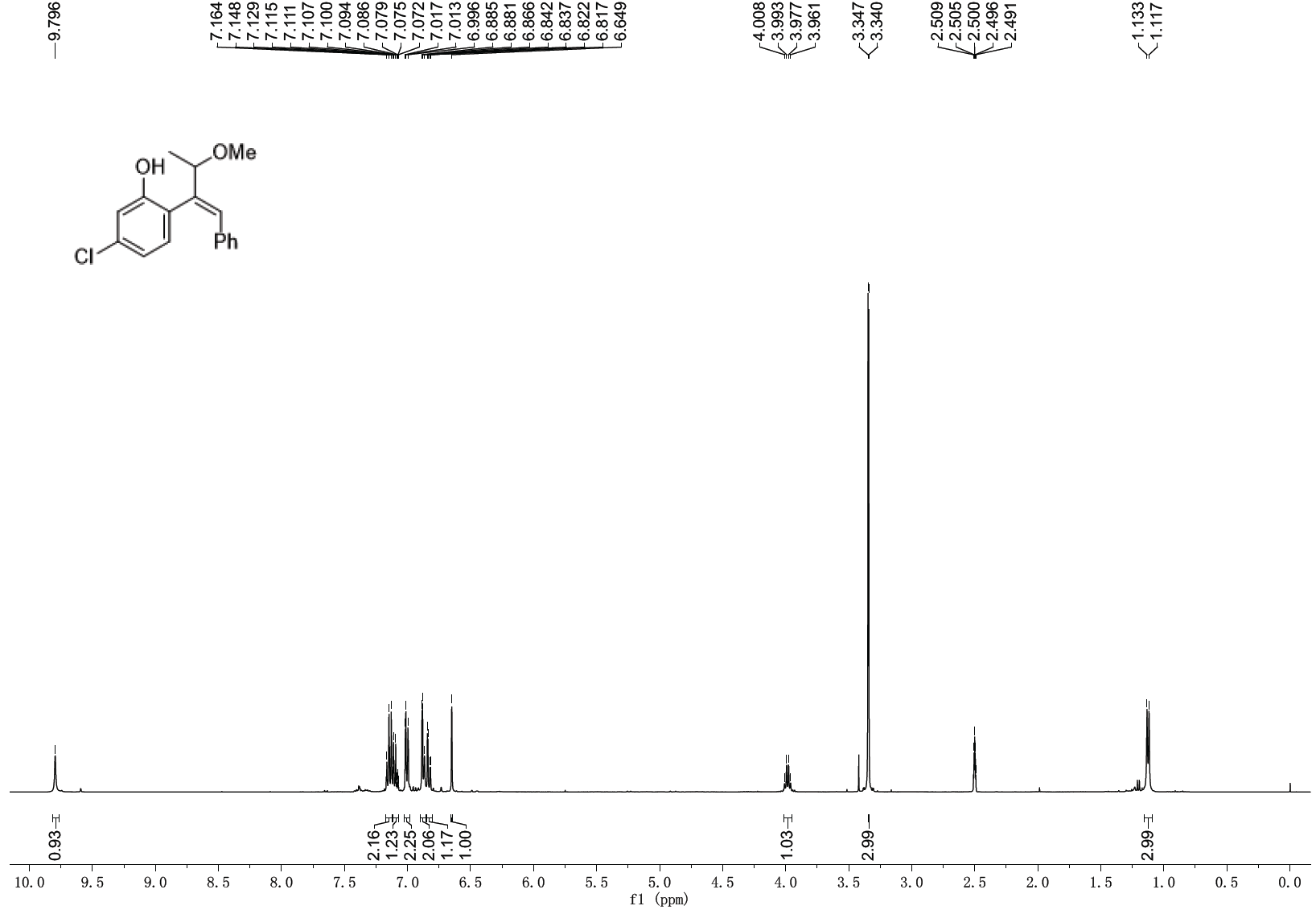
**

**Supplementary Figure 95.** ^1^H-NMR spectrum of **4m**

**
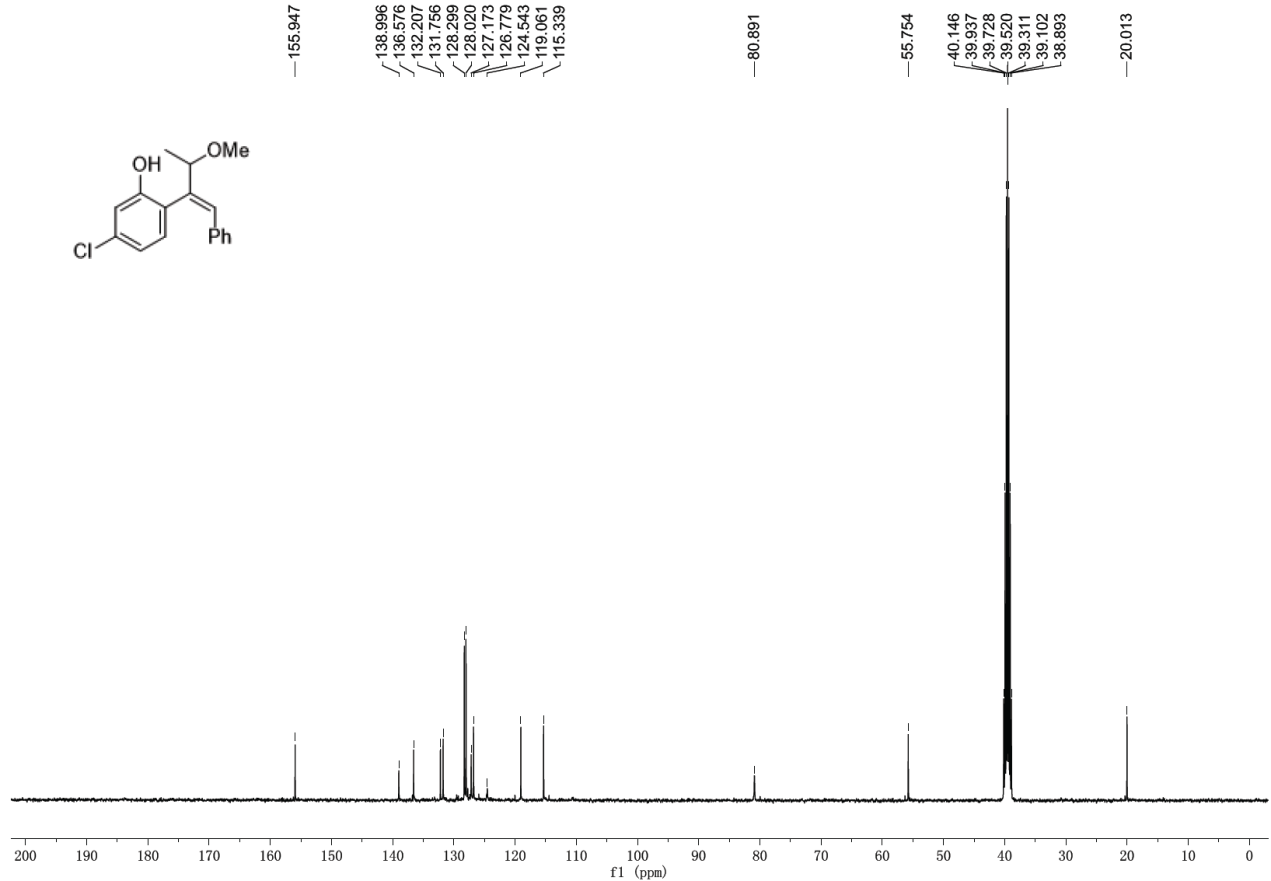
**

**Supplementary Figure 96.** ^13^C-NMR spectrum of **4m**

**4n**

**
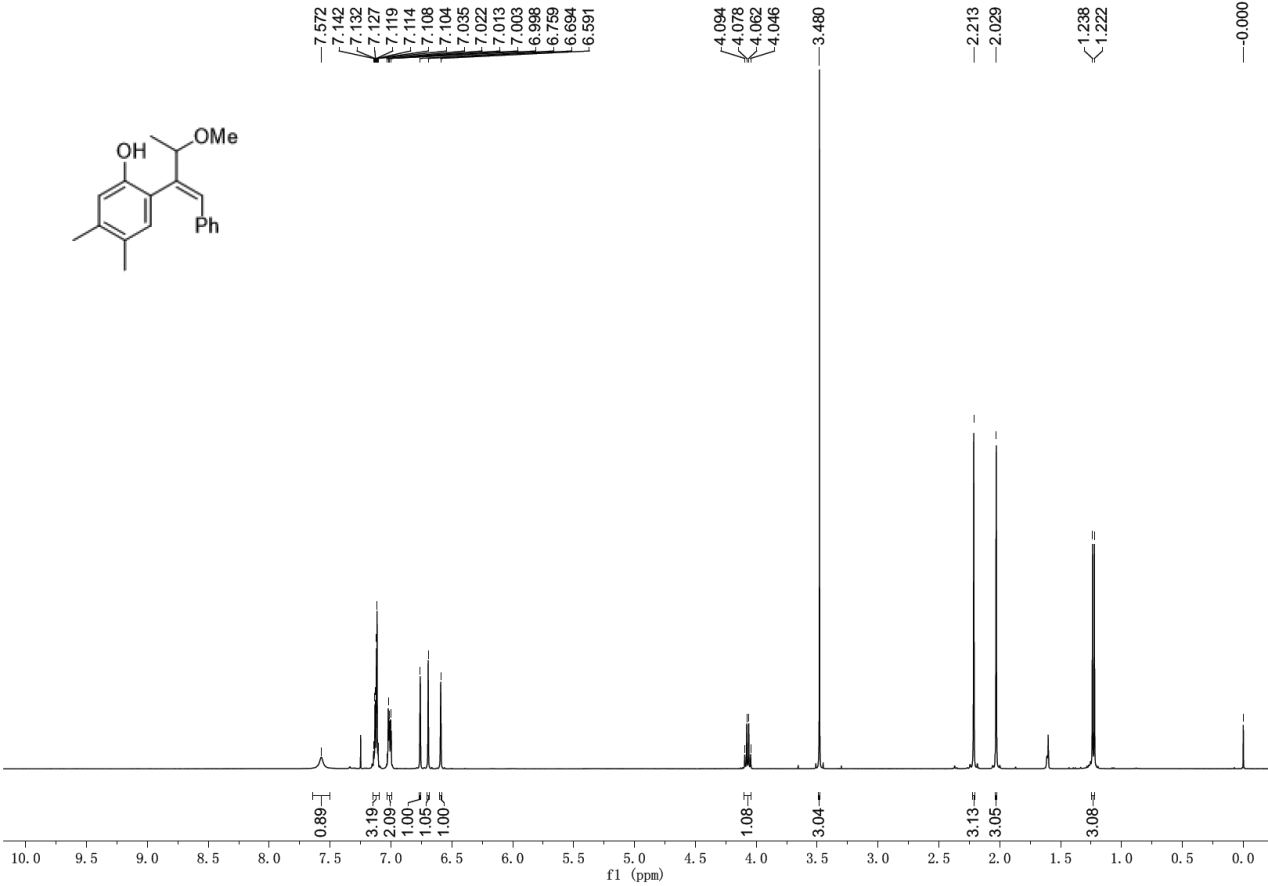
**

**Supplementary Figure 97.** ^1^H-NMR spectrum of **4n**

**
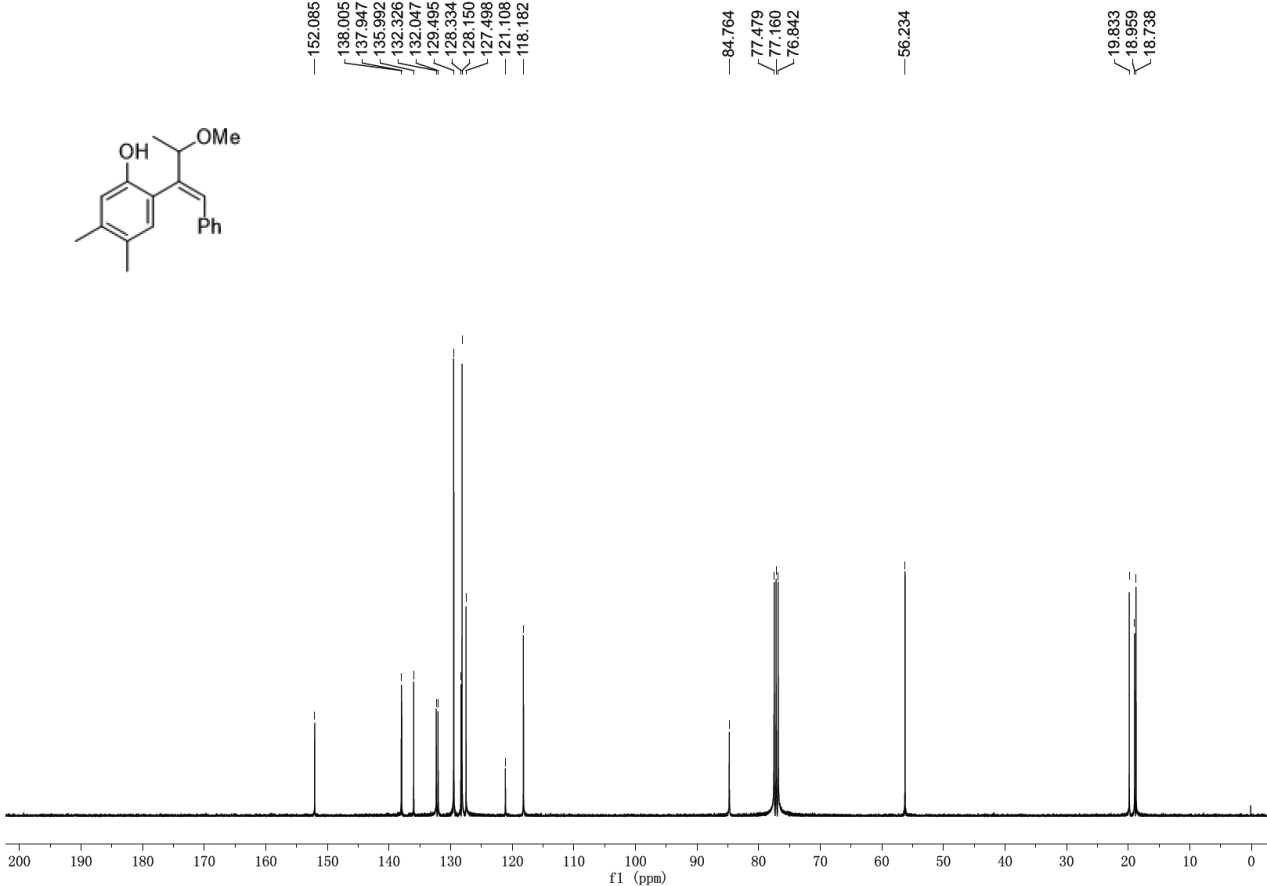
**

**Supplementary Figure 98.** ^13^C-NMR spectrum of **4n**

**4o**

**
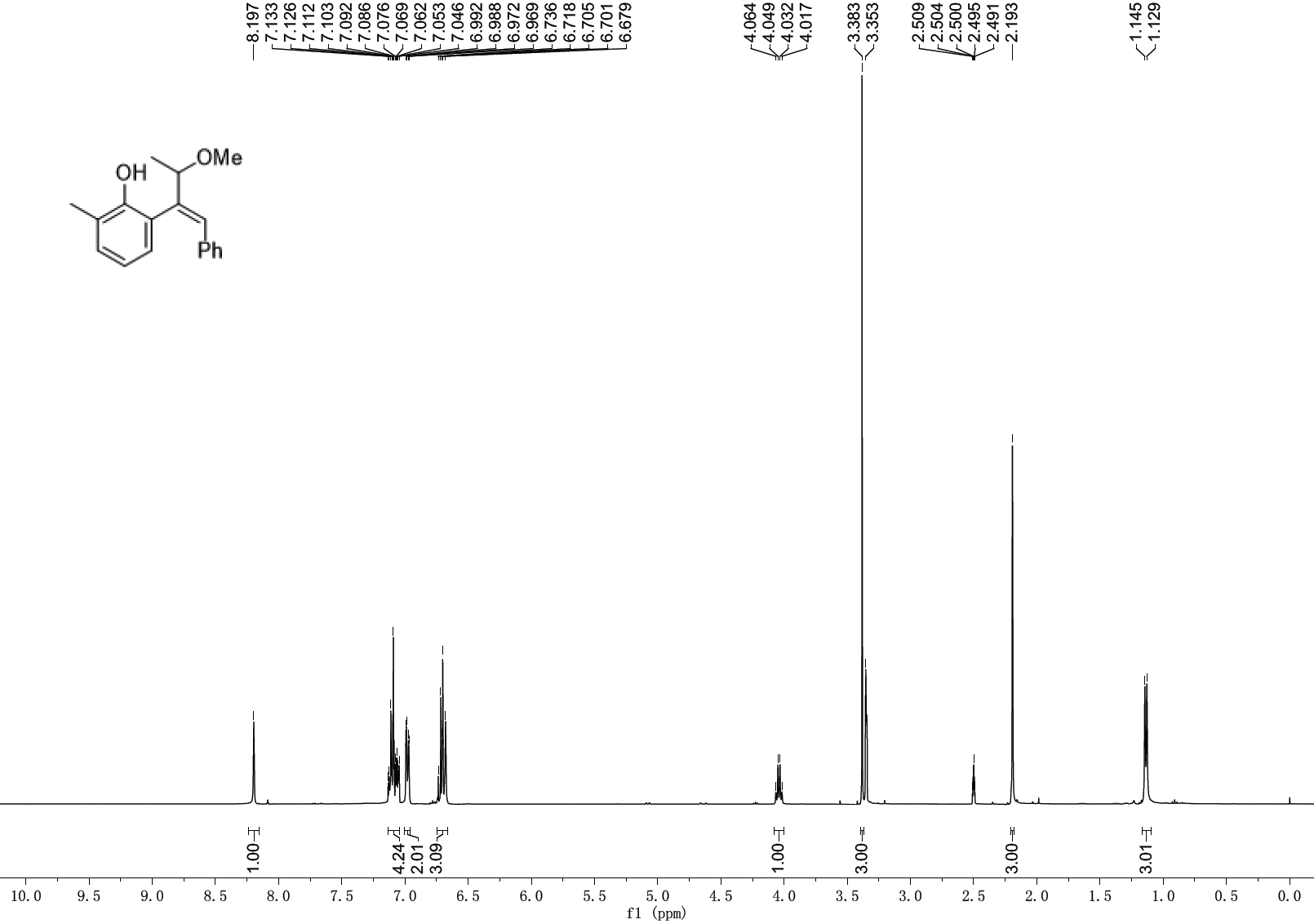
**

**Supplementary Figure 99.** ^1^H-NMR spectrum of **4o**

**
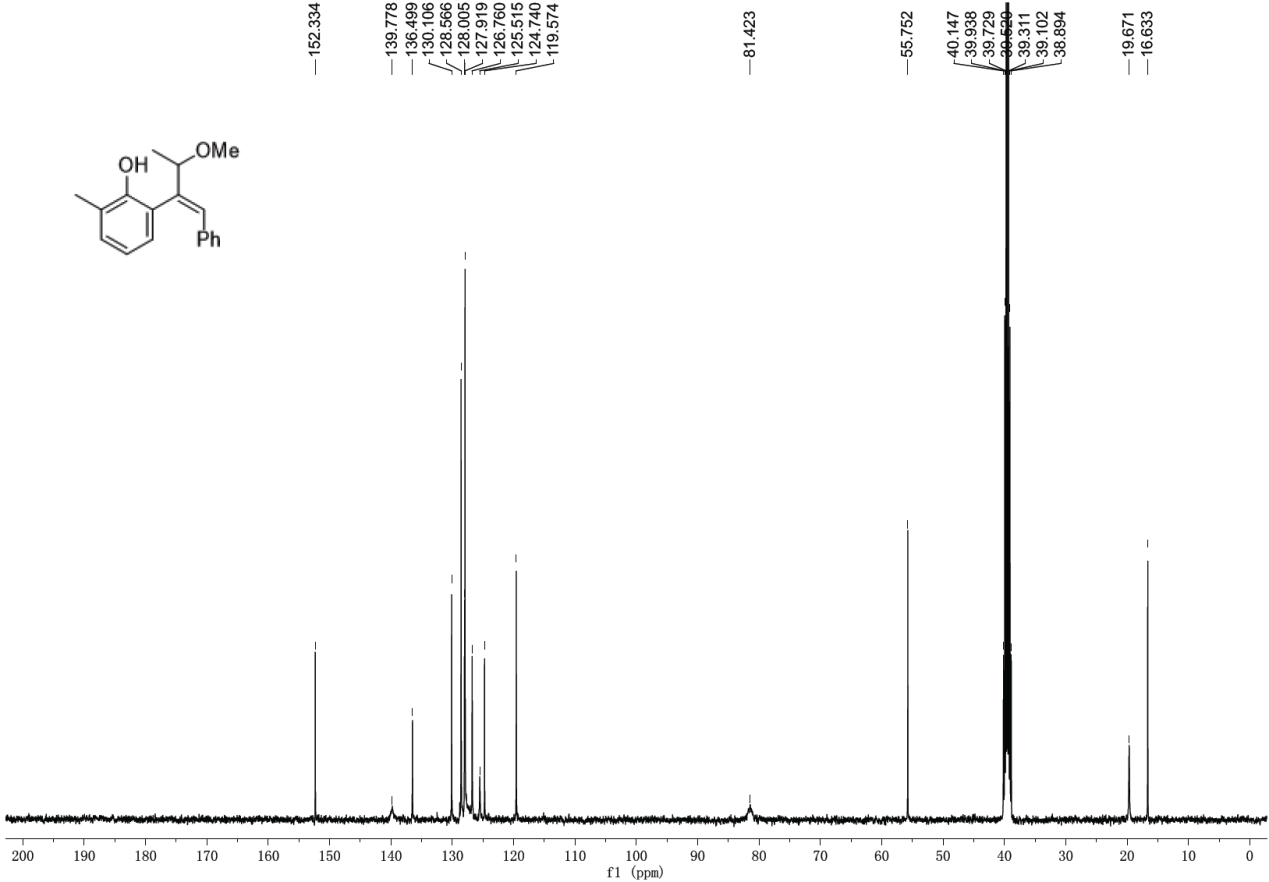
**

**Supplementary Figure 100.** ^13^C-NMR spectrum of **4o**

**4p**

**
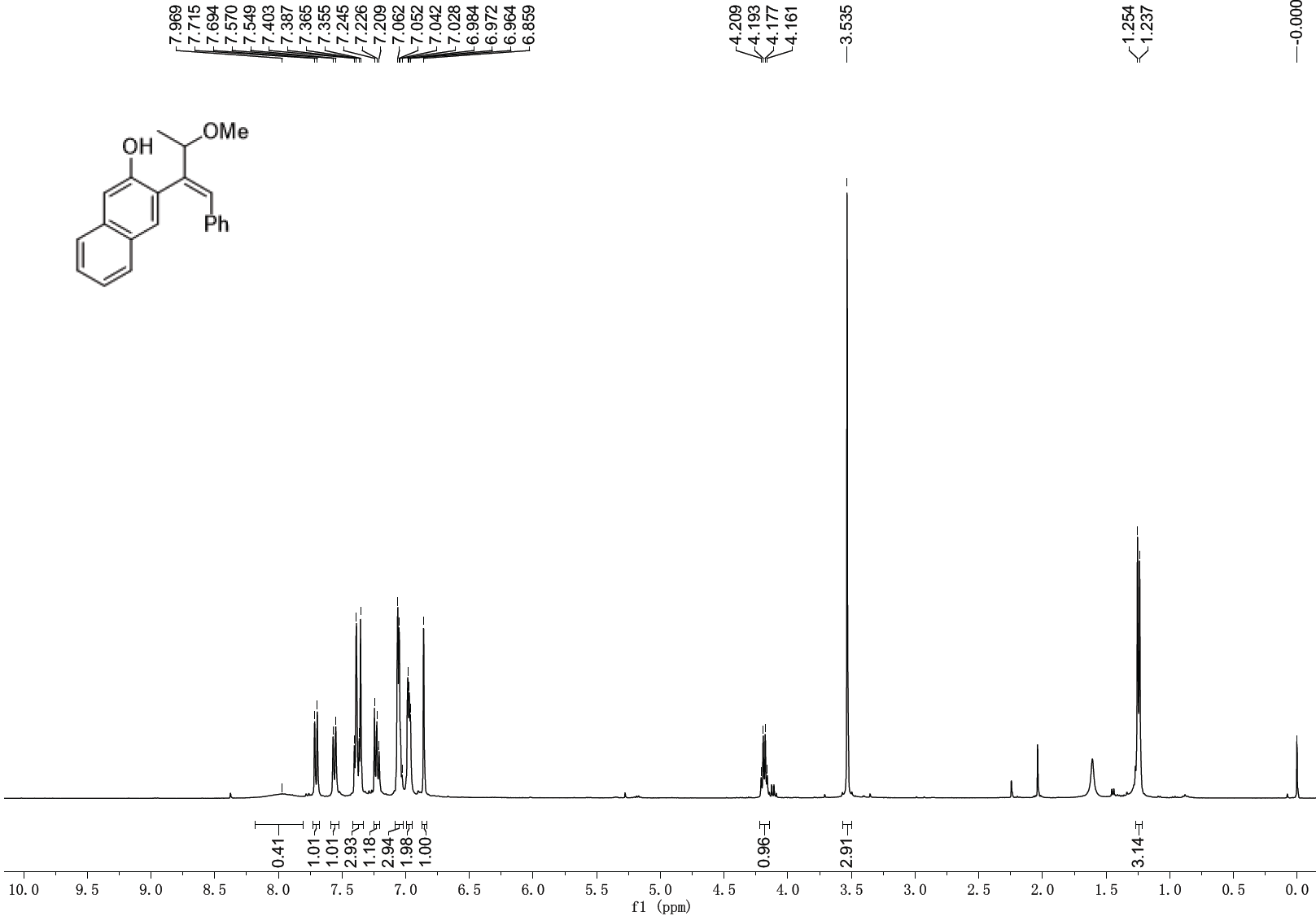
**

**Supplementary Figure 101.** ^1^H-NMR spectrum of **4p**

**
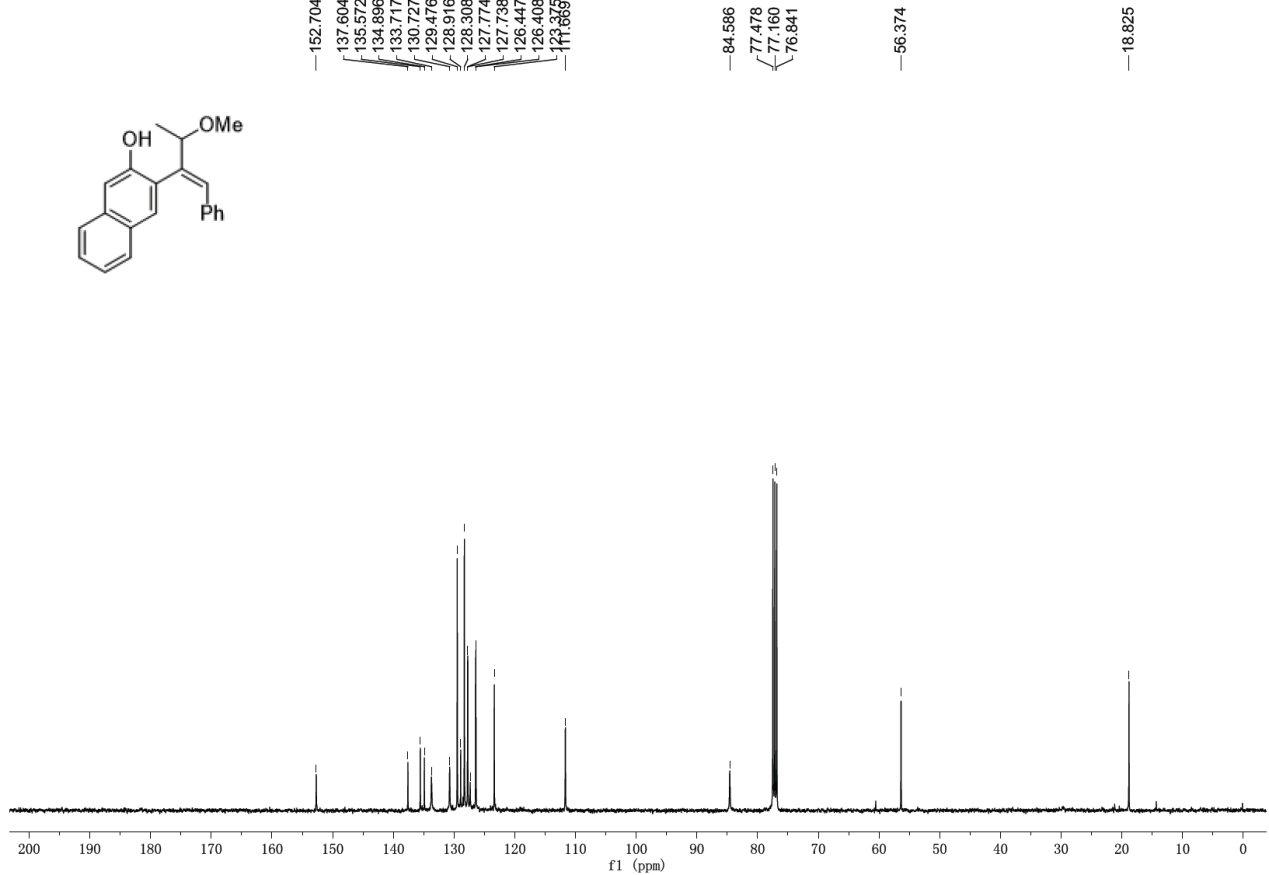
**

**Supplementary Figure 102.** ^13^C-NMR spectrum of **4p**

**4q**

**Supplementary Figure 103.** ^1^H-NMR spectrum of **4q**

**Supplementary Figure 104.** ^13^C-NMR spectrum of **4q**

**4r**

**Supplementary Figure 105.** ^1^H-NMR spectrum of **4r**

**Supplementary Figure 106.** ^13^C-NMR spectrum of **4r**

**4s**

**Supplementary Figure 107.** ^1^H-NMR spectrum of **4s**

**Supplementary Figure 108.** ^13^C-NMR spectrum of **4s**

**4t**

**Supplementary Figure 109.** ^1^H-NMR spectrum of **4t**

**Supplementary Figure 110.** ^13^C-NMR spectrum of **4t**

**Supplementary Figure 111.** ^19^F-NMR spectrum of **4t**

**4u**

**Supplementary Figure 112.** ^1^H-NMR spectrum of **4u**

**Supplementary Figure 113.** ^13^C-NMR spectrum of **4u**

**4v**

**Supplementary Figure 114.** ^1^H-NMR spectrum of **4v**

**Supplementary Figure 115.** ^13^C-NMR spectrum of **4v**

**4w**

**Supplementary Figure 116.** ^1^H-NMR spectrum of **4w**

**Supplementary Figure 117.** ^13^C-NMR spectrum of **4w**

**Supplementary Figure 118.** ^19^F-NMR spectrum of **4w**

**4x**

**Supplementary Figure 119.** ^1^H-NMR spectrum of **4x**

**Supplementary Figure 120.** ^13^C-NMR spectrum of **4x**

**4y**

**Supplementary Figure 121.** ^1^H-NMR spectrum of **4y**

**Supplementary Figure 122.** ^13^C-NMR spectrum of **4y**

**4z**

**Supplementary Figure 123.** ^1^H-NMR spectrum of **4z**

**Supplementary Figure 124.** ^13^C-NMR spectrum of **4z**

**5a**

**Supplementary Figure 125.** ^1^H-NMR spectrum of **5a**

**Supplementary Figure 126.** ^13^C-NMR spectrum of **5a**

**NOESY of 5a**

**Supplementary Figure 127.** ^1^H-^1^H NOESY spectrum of **5a**

**5b**

**Supplementary Figure 128.** ^1^H-NMR spectrum of **5b**

**Supplementary Figure 129.** ^13^C-NMR spectrum of **5b**

**5c**

**Supplementary Figure 130.** ^1^H-NMR spectrum of **5c**

**Supplementary Figure 131.** ^13^C-NMR spectrum of **5c**

**Supplementary Figure 132.** ^19^F-NMR spectrum of **5c**

**NOESY of 5c**

**Supplementary Figure 133.** ^1^H-^1^H NOESY spectrum of **5c**

**5d**

**Supplementary Figure 134.** ^1^H-NMR spectrum of **5d**

**Supplementary Figure 135.** ^13^C-NMR spectrum of **5d**

**5e**

**Supplementary Figure 136.** ^1^H-NMR spectrum of **5e**

**Supplementary Figure 137.** ^13^C-NMR spectrum of **5e**

**5f**

**Supplementary Figure 138.** ^1^H-NMR spectrum of **5f**

**Supplementary Figure 139.** ^13^C-NMR spectrum of **5f**

**5g**

**Supplementary Figure 140.** ^1^H-NMR spectrum of **5g**

**Supplementary Figure 141.** ^13^C-NMR spectrum of **5g**

**5h**

**Supplementary Figure 142.** ^1^H-NMR spectrum of **5h**

**Supplementary Figure 143.** ^13^C-NMR spectrum of **5h**

**5i**

**Supplementary Figure 144.** ^1^H-NMR spectrum of **5i**

**Supplementary Figure 145.** ^13^C-NMR spectrum of **5i**

**5j**

**Supplementary Figure 146.** ^1^H-NMR spectrum of **5j**

**Supplementary Figure 147.** ^13^C-NMR spectrum of **5j**

**Supplementary Figure 148.** ^19^F-NMR spectrum of **5j**

**NOESY of 5j**

**Supplementary Figure 149.** ^1^H-^1^H NOESY spectrum of **5j**

**6a**

**Supplementary Figure 150.** ^1^H-NMR spectrum of **6a**

**Supplementary Figure 151.** ^13^C-NMR spectrum of **6a**

**6b**

**Supplementary Figure 152.** ^1^H-NMR spectrum of **6b**

**Supplementary Figure 153.** ^13^C-NMR spectrum of **6b**

**6c**

**Supplementary Figure 154.** ^1^H-NMR spectrum of **6c**

**Supplementary Figure 155.** ^13^C-NMR spectrum of **6c**

**6d**

**Supplementary Figure 156.** ^1^H-NMR spectrum of **6d**

**Supplementary Figure 157.** ^13^C-NMR spectrum of **6d**

**6e**

**Supplementary Figure 158.** ^1^H-NMR spectrum of **6e**

**Supplementary Figure 159.** ^13^C-NMR spectrum of **6e**

**Supplementary Figure 160.** ^19^F-NMR spectrum of **6e**

**6f**

**Supplementary Figure 161.** ^1^H-NMR spectrum of **6f**

**Supplementary Figure 162.** ^13^C-NMR spectrum of **6f**

**6g**

**Supplementary Figure 163.** ^1^H-NMR spectrum of **6g**

**Supplementary Figure 164.** ^13^C-NMR spectrum of **6g**

**6h**

**Supplementary Figure 165.** ^1^H-NMR spectrum of **6h**

**Supplementary Figure 166.** ^13^C-NMR spectrum of **6h**

**6i**

**Supplementary Figure 167.** ^1^H-NMR spectrum of **6i**

**Supplementary Figure 168.** ^13^C-NMR spectrum of **6i**

**6j**

**Supplementary Figure 169.** ^1^H-NMR spectrum of **6j**

**Supplementary Figure 170.** ^13^C-NMR spectrum of **6j**

**Supplementary Figure 171.** ^19^F-NMR spectrum of **6j**

**6k**

**Supplementary Figure 172.** ^1^H-NMR spectrum of **6k**

**Supplementary Figure 173.** ^13^C-NMR spectrum of **6k**

**6l**

**Supplementary Figure 174.** ^1^H-NMR spectrum of **6l**

**Supplementary Figure 175.** ^13^C-NMR spectrum of **6l**

**6m**

**Supplementary Figure 176.** ^1^H-NMR spectrum of **6m**

**Supplementary Figure 177.** ^13^C-NMR spectrum of **6m**

**6n**

**Supplementary Figure 178.** ^1^H-NMR spectrum of **6n**

**Supplementary Figure 179.** ^13^C-NMR spectrum of **6n**

**Supplementary Figure 180.** ^19^F-NMR spectrum of **6n**

**6o**

**Supplementary Figure 181.** ^1^H-NMR spectrum of **6o**

**Supplementary Figure 182.** ^13^C-NMR spectrum of **6o**

**6p**

**Supplementary Figure 183.** ^1^H-NMR spectrum of **6p**

**Supplementary Figure 184.** ^13^C-NMR spectrum of **6p**

**6q**

**Supplementary Figure 185.** ^1^H-NMR spectrum of **6q**

**Supplementary Figure 186.** ^13^C-NMR spectrum of **6q**

**6r**

**Supplementary Figure 187.** ^1^H-NMR spectrum of **6r**

**Supplementary Figure 188.** ^13^C-NMR spectrum of **6r**

**6s**

**Supplementary Figure 189.** ^1^H-NMR spectrum of **6s**

**Supplementary Figure 190.** ^13^C-NMR spectrum of **6s**

**6t**

**Supplementary Figure 191.** ^1^H-NMR spectrum of **6t**

**Supplementary Figure 192.** ^13^C-NMR spectrum of **6t**

**7**

**Supplementary Figure 193.** ^1^H-NMR spectrum of **7**

**Supplementary Figure 194.** ^13^C-NMR spectrum of **7**

**8**

**Supplementary Figure 195.** ^1^H-NMR spectrum of **8**

**Supplementary Figure 196.** ^13^C-NMR spectrum of **8**

**9**

**Supplementary Figure 197.** ^1^H-NMR spectrum of **9**

**Supplementary Figure 198.** ^13^C-NMR spectrum of **9**

**10**

**Supplementary Figure 199.** ^1^H-NMR spectrum of **10**

**Supplementary Figure 200.** ^13^C-NMR spectrum of **10**

**11**

**Supplementary Figure 201.** ^1^H-NMR spectrum of **11**

**Supplementary Figure 202.** ^13^C-NMR spectrum of **11**

**12**

**Supplementary Figure 203.** ^1^H-NMR spectrum of **12**

**Supplementary Figure 204.** ^13^C-NMR spectrum of **12**

**13**

**Supplementary Figure 205.** ^1^H-NMR spectrum of **13**

**Supplementary Figure 206.** ^13^C-NMR spectrum of **13**

**14**

**Supplementary Figure 207.** ^1^H-NMR spectrum of **14**

**Supplementary Figure 208.** ^13^C-NMR spectrum of **14**

**15**

**Supplementary Figure 209.** ^1^H-NMR spectrum of **15**

**Supplementary Figure 210.** ^13^C-NMR spectrum of **15**

**16**

**Supplementary Figure 211.** ^1^H-NMR spectrum of **16**

**Supplementary Figure 212.** ^13^C-NMR spectrum of **16**

**17**

**Supplementary Figure 213.** ^1^H-NMR spectrum of **17**

**Supplementary Figure 214.** ^13^C-NMR spectrum of **17**

**18**

**Supplementary Figure 215.** ^1^H-NMR spectrum of **18**

**Supplementary Figure 216.** ^13^C-NMR spectrum of **18**

**19**

**Supplementary Figure 217.** ^1^H-NMR spectrum of **19**

**Supplementary Figure 218.** ^13^C-NMR spectrum of **19**

**20**

**Supplementary Figure 219.** ^1^H-NMR spectrum of **20**

**Supplementary Figure 220.** ^13^C-NMR spectrum of **20**

**21**

**Supplementary Figure 221.** ^1^H-NMR spectrum of **21**

**Supplementary Figure 222.** ^13^C-NMR spectrum of **21**

**Supplementary Figure 223.** ^19^F-NMR spectrum of **21**

**22**

**Supplementary Figure 224.** ^1^H-NMR spectrum of **22**

**Supplementary Figure 225.** ^13^C-NMR spectrum of **22**

**23**

**Supplementary Figure 226.** ^1^H-NMR spectrum of **23**

**Supplementary Figure 227.** ^13^C-NMR spectrum of **23**
